# Transcriptional Signatures in Liver Reveal Metabolic Adaptations to Seasons in Migratory Blackheaded Buntings

**DOI:** 10.3389/fphys.2018.01568

**Published:** 2018-11-27

**Authors:** Devraj Singh, Vivek Swarup, Hiep Le, Vinod Kumar

**Affiliations:** ^1^IndoUS Center for Biological Timing, Department of Zoology, University of Delhi, New Delhi, India; ^2^Department of Neurology, University of California, Los Angeles, Los Angeles, CA, United States; ^3^Salk Institute for Biological Studies, La Jolla, CA, United States

**Keywords:** adaptation, birds, gene expression, migration, RNAseq, WGCNA

## Abstract

The molecular underpinnings of metabolic adaptation to seasons are poorly understood in long- distance migrants. We measured changes in physiology and performed *de novo* sequencing of RNA extracted from liver samples collected at 4-h intervals over a period of 24 h from a long-distance avian migrant, the blackheaded bunting (*Emberiza melanocephala*), during two states: photostimulated vernal migratory (M) state and photorefractory non-migratory (nM) state. The M state was differentiated from the nM state based on body fattening and weight gain, as well as on *Zugunruhe*, that is, nocturnal migratory restlessness in caged birds. We found that baseline blood glucose and triglyceride levels were significantly higher in the M state than the nM state; conversely, surface body temperature was higher in the nM state than the M state. In a total of 6 liver samples that were sequenced from each state, 11,246 genes were annotated, including 4448 genes that were cyclic over 24 h. We found 569 differentially expressed genes (DEGs) between the M and the nM state, and the M state showed 131 upregulated and 438 downregulated genes. These DEGs formed core gene hubs associated with specific biological processes in both the states. In addition, weighted gene coexpression network analysis revealed two discrete modules of coexpressed genes, with a significant difference in the expression pattern of metab olism-associated genes between M and nM states. These results demonstrate, for the first time, transcriptome-wide changes in the liver between two distinct physiological states and give molecular insights into seasonal metabolic adaptations in latitudinal migrants.

## Introduction

Bird migration is one of the most salient examples of seasonal ecologyin nature. Annual to-and-fro movements between breeding and non-breeding grounds occur during different seasons and involve significant adaptations to environmental changes in physiology and behavior (Berthold et al., 2003). Following postbreeding molt, avian migrants undergo changes associated with autumn migration (to wintering site) and reverse to a non-migratory state when it overwinters. Later, it reinitiates processes associated with spring migration (to breeding site) and reproduction, which are again reversed at the end of reproduction. These changes are programmed by internal clocks and synchronized to the natural environment by the annual photoperiodic cycle (Gwinner and Helm, 2003; Malik et al., 2014). Increasing photoperiods at the wintering site photostimulate the spring (vernal) migratory state, which is characterized by fat deposition and weight gain; elevated circulating levels of metabolites (e.g., glucose and triglycerides) and metabolic hormones (e.g., insulin and corticosterone); and *Zugunruhe* (migratory restlessness, intense nocturnal activity, and wing whirring in captive birds: Gwinner and Czeschlik, 1978; Ramenofsky and Wingfield, 2007; Trivedi et al., 2014). At the end of the vernal migration, fat depletes, body mass declines, and gonads mature and enlarge in size. Following reproduction, gonads regress as birds enter into photorefractory state during which they remain unresponsive to stimulatory effects of long photoperiod (Malik et al., 2014; Trivedi et al., 2014).

The photostimulated vernal migratory state and the photorefractory non-migratory state strikingly differ in behavior and physiology, particularly in aspects that are linked with night-flight and associated metabolism and energy homeostasis (Bairlein et al., 2015). Both seasonal states are also indicators of changes at multiple levels in response to seasonal changes in the environment. The liver is the major site for seasonal metabolic changes, and it plays crucial roles in the maintenance of global metabolic (energy) homeostasis. In particular, the liver regulates glucose and lipid metabolism: for example, glycogenesis, gluconeogenesis, detoxification, production of plasma protein and bile acids, and synthesis of lipoproteins (Trefts et al., 2015; Kurauti et al., 2016). In avian migrants during the migratory state, the liver actively metabolizes abundant nutritional substrates and stores fuel for flight in the form of triglycerides and also processes fuel substrates for use by other tissues. During the non-migratory state, the liver reverts to its baseline activity (Jenni-Eiermann et al., 2002; Landys et al., 2005; Bairlein et al., 2015). A few recent findings have shown molecular changes in the liver between migratory and non-migratory states in songbird migrants. There were significant differences in the hepatic expression of genes associated with glucose (*SIRT1*, *FOXO1*, *PYGL*, and *GLUT1*) and lipid (*HMGCOA*, *PPAR*s, and *FASN*) metabolism between photoperiod-induced migratory and non-migratory states in migratory blackheaded buntings (*Emberiza melanocephala*; Trivedi et al., 2014). Similarly, there were also differences in 24-h expression patterns of the core clock genes (*BMAL1*, *CLOCK*, *PERIOD2*, and *CRY1*) in the liver of blackheaded buntings (Singh et al., 2015). Furthermore, analyses of the transcriptome of two migratory subspecies of Scandinavian willow warblers (*Phylloscopus trochilus*) showed that differences in their migratory strategies were likely to be governed by only few genes, by temporal differences, or by tissue-specific gene expression patterns (Lundberg et al., 2013; Boss et al., 2016). Similarly, RNA-Seq analyses of blood and pectoral muscle revealed 547 differentially expressed genes (DEGs) between sedentary (*J. h. carolinensis*) and migratory (*J. h. hyemalis*) subspecies of dark eyed juncos (*Junco hyemalis*; Fudickar et al., 2016). There were also 188 DEGs related to the migratory state in the ventral hypothalamus of Swainson’s thrush (*Catharus ustulatus*) as revealed by RNA-Seq study by Johnston et al. (2016).

How the liver meets differential energy requirements of migratory and non-migratory states remains poorly understood at the mechanistic level. In order to address this, we performed RNA-Seq of liver from two groups of blackheaded buntings that were photostimulated into the vernal (spring) migratory state or the photorefractory (postbreeding) non-migratory state. These two states provided a contrasting continuum of the photostimulated induction and cessation of physiological processes associated with photoperiod-induced seasonal states in buntings’ annual life history (Misra et al., 2004; Trivedi et al., 2014; Singh et al., 2015). By comparing the liver transcriptome from these two photostimulated states, we sought to identify DEGs and generate coexpression networks of the gene. We then correlated physiological parameters with gene expression patterns to suggest probable molecular pathways that were possibly associated with seasonal metabolic adaptations in the migratory blackheaded bunting. Thus, the overall goal of this study was to understand the molecular underpinnings of physiological and metabolic adaptations that are a part of the broad adaptability of avian migrants to seasons in the natural environment.

## Materials and Methods

### Animals and Experiment

Adult male blackheaded buntings (*E. melanocephala*) were procured from the overwintering flock in the wild, and following acclimation for a week in an outdoor aviary, they were maintained at a temperature of 22 ± 2°C on short days (SD, 8 h light: 16 h darkness; 8L: 16D) or long days (LD, 16L: 8D) for the following 40 weeks. Under SD, buntings remain unstimulated and photosensitive, which is characterized by normal body mass (no fat deposition), daytime activity, and small reproductively inactive gonads. Under LD, however, buntings are photostimulated. Within the first 2–3 weeks of LD exposure, buntings deposit fat and gain weight, recrudesce testes, and exhibit *Zugunruhe*. These photostimulated changes are reversed after 10–12 weeks of LD: buntings become lean, regress testes, return to daytime activity, and exhibit photorefractoriness (Misra et al., 2004; Rani et al., 2006; Trivedi et al., 2014; Singh et al., 2015). Thus, photostimulated ‘vernal’ migratory (M) and photorefractory ‘postbreeding’ non-migratory (nM) states present a contrast within the continuum of seasonal states in the annual life history of the latitudinal migratory blackheaded buntings.

This experiment lasted for about 4 weeks and used SD photosensitive and LD photorefractory buntings (*n* = 24 each). Short days and LD buntings were in different seasonal physiological states but with similar phenotypes in showing the normal body mass and small testes. At the beginning of the experiment, neither group showed body fat deposits, weighed 23–27 g, and had unstimulated (or regressed) reproductively inactive small testes (0.33–0.52 mm^3^). Birds were singly housed in activity cages (size = 60 cm × 45 cm × 35 cm) that were individually placed in photoperiodic chambers providing programmed lighting (L = 350 ± 10 lux; D = ∼0.4 lux). Short days photosensitive birds were exposed to LD for 18–24 days so that each individual had shown 7 nights of *Zugunruhe.* Long days photorefractory birds were maintained under LD for 24 days.

### Measurement of Changes in Behavior and Physiology

We monitored 24-h activity-rest pattern for changes in behavior. We also measured food intake (FI), body mass, surface body temperature, and blood metabolites in order to determine changes in physiology between photostimulated M and photorefractory nM states. Each bird was handled several times in both light and dark periods to get acclimatized in order to avoid handling or neophobia-induced stress during the experiment. The detail for each measurement has been described in our previous publications (Singh et al., 2010, 2015; Trivedi et al., 2015). Briefly, an infrared sensor mounted on the activity cage continuously monitored general activity of a bird in its cage. Activity data were collected and analyzed using the Chronobiology Kit software program (Stanford Software Systems, CA, United States). We measured FI (g bird^-1^ day^-1^; *n* = 8) and body mass (g; *n* = 12) of randomly selected birds from each group both at the beginning and end of the experiment and recorded surface body temperature (^o^C; *n* = 9 or 10) at 4-h intervals (beginning 1 h after light on, i.e., ZT 1, 5, 9, 13, 17, and 21; ZT, zeitgeber time 0 = light on), a day before the experiment ended.

Food intake was measured consecutively for 2 days. After 24 h from the time food was given on the previous day, food bowl and spillage along with feces were removed. After feces removal, the weight of food collected was subtracted from food given, and the average for 2 days gave FI in g bird^-1^ day-1. From this, the mean (± SE) for the group was calculated. We considered the change in body mass as an index of body fattening, since most, if not all, of the photostimulated fat deposition accounts for weight gain in migratory songbirds including blackheaded buntings (King and Farner, 1959; Misra et al., 2004; Rani et al., 2006; Mishra et al., 2017a). To record body mass, birds were weighed on a top-pan balance with an accuracy of 0.1 g. We recorded surface body temperature using thermoscan (Quick shot Infra-red thermometer; model Exp-01B), which measures body temperature in the range of 32–42^o^C. For this, a gentle air-blow exposed the keel region skin, and the temperature was recorded as an average of 4–5 readings in a quick succession with thermoscan placed at a distance of about 2 cm.

At the same six time points, we collected blood samples from wing vein (*n* = 4 *n* = time point) and measured serum glucose and triglycerides levels by quantitative colorimetric determination using specific kits. A bird was bled only once, 100–150 μl of blood was collected each time by puncturing the wing vein into a capillary tube. The blood sample was first rested at room temperature for 10 min and then was put at 4°C for an hour before it was centrifuged at 1630 g for 15 min at 4^o^C. Serum was harvested and stored at -20°C until assayed for glucose and triglyceride concentrations. We used QuantiChromTM Glucose (Cat. #: DIGL-100) and EnzyChromTM triglycerides (Cat. #: ETGA-200) assay kits for the measurement of glucose and triglycerides levels in 5 and 10 μl serum samples, respectively, and followed the procedure as per the manufacturer’s protocol. Briefly, serum, standards, and reagents were thawed on ice before the assay began. For glucose, standards were diluted in distilled water to final concentrations of 300, 200, 100, 50 mg/dl; distilled water was also taken as blank sample and served as a control. A volume of 5 μl of neat serum and diluted standard aliquots were mixed each with 500 μl of reagent in 1.5 ml tubes. These tubes were then kept in a boiling water bath for 8 min and cooled afterward on a water bath for 4 min. An aliquot of 200 μl of each treated sample and standard was pipetted out into separate wells of a 96-well plate, and optical density (OD) was measured at 630 nm light wavelength. The glucose concentration in each sample was calculated as follows: concentration (mg/dl) = OD sample - OD blank/slope.

For trigyclerides assay, standards were diluted in distilled water to get a concentration of 1, 0.6, 0.3 mmol/l; distilled water was taken as blank sample and served as control. A aliquot of 10 μl of serum diluted five-fold in distilled water was used for the assay. A working reagent was prepared by mixing 100 μl assay buffer, 2 μl enzyme mix, 5 μl lipase, 1 μl ATP, and 1 μl dye in a clean tube. An aliquot of 100 μl of working buffer was added and tap-mixed with sample/standard. This mixture was incubated for 30 min at room temperature, and the OD was measured on a microplate reader at 570 nm light wavelength. Triglycerides concentration of sample was calculated as follows: concentration (mmol/l) = (OD sample-OD water/slope) × N; N is the serum dilution factor.

Next day, birds were sacrificed at the respective time points, and the liver was harvested. Whereas a small piece was processed for hematoxylin-eosin (H-E; Sigma-Aldrich) and transmission electron microscopy (TEM; Tecnai, G20, FEI) histologies, the remaining liver tissue was placed in RNA-later^TM^ (ThermoFisher Scientific, Cat. # AM7020) and stored frozen at -80^o^C until used for RNA-Seq. To examine differences at the histological level, a liver piece was fixed in 4% formaldehyde tissue, cryosectioned at 8-μm-thickness, stained with H-E, and passed through ascending grades of alcohol, and cover-slipped. An AxioCam ICc1 Rev.4 camera attached to the Zeiss Axio Imager M2 microscope digitally imaged the stained sections. Another 2 mm× 2 mm liver piece was prepared for TEM to show a better resolution of differences in fat droplets and vacuoles in the liver between the two physiological states.

Statistical analyses of behavioral and physiological parameters were done using the GraphPad prism (version 5.0) software and the SPSS statistics version-20 software, as appropriate. The effect size (η^2^) of samples was calculated using the SPSS statistics version 20 software.

### Measurement of Gene Expression

#### RNA Extraction and Sequencing

In total, 12 liver samples were sequenced and mapped against the genome of zebra finch. Samples were collected every 4 h over a 24-h period; therefore, there were two samples (one from each state) collected at six time points. We extracted total RNA from all the samples in one batch using Trizol Plus RNA Purification Kit (ThermoFisher Scientific, Cat. # 12183555) and included DNase treatment with Ambion RNase-Free DNase Set and column cleanup with Purelink RNA Mini Kit (Invitrogen, Cat. # 12183018A), as per the manufacturers’ protocol. We used a Ribogreen assay (Invitrogen, R11490) to determine a sample’s RNA concentration and RNA integrity number (RIN; Schroeder et al., 2006) on Agilent 2100 Bioanalyzer. An amount of 1 μg of total RNA was used to generate poly-A selected mRNAs, which were isolated using oligo-dT attached magnetic beads. Complementary DNA (cDNA, 150 – 500 bp with mean size of 250 bp) libraries were generated with TruSeq RNA Sample Preparation Kit v2 (Illumina, San Diego, CA, United States; Cat. # RS-122-2001). Quantified (Quant-iT PicoGreen assay; Life Technologies, P11496) and validated (Agilent 2200 TapeStation system) libraries from all the samples were sequenced in a single lane as 100-bp single-end reads on Illumina HiSeq 2500.

#### Quality Filtering and Mapping

Single end reads of 100 bp with an average depth of 25 million reads were generated for each liver sample (Supplementary Table S1). Raw reads in sff format were converted into fastq format using the processing kit seq_crumbs^1^, and adapters and low quality reads were clipped. Reads were then subjected to quality control using FastQCv0.10^2^ with a passing score threshold Q > 30. Prior to assembly, we eliminated the majority of ribosomal RNA (rRNA) sequences using the short sequence alignment algorithm Bowtie 2 (version 2.0.2). Initial assembly was done using ultrafast universal RNA-Seq aligner STAR (Dobin et al., 2013) and the latest zebra finch (*Taeniopygia guttata*) genome (taeGut build 3.2.4). We performed genome-guided transcript assembly of *T. guttata*, and using Cufflinks (v2.2.1), we conducted fragments per kilobase of transcript per million mapped reads (FPKM) quantification (Trapnell et al., 2012). Finally, we annotated draft assembly using BLASTX with e-value ≤ E^-10^ taking the reference of zebra finch protein database, which is an accessible analytical tool (Lundberg et al., 2013).

#### Differential Gene Expression Analysis

Using the criterion of FPKM value > 0.1 for an expressed gene in 80% of samples, we were able to confidently obtain expression values for a total of 11,246 genes. The log_2_ transformed FPKM values were used for all downstream analyses. We used linear regression model in the R package to perform differential gene expressions (DGEs). False-discovery rate (FDR) < 0.1 and absolute log_2_ fold change > 0.1 were set as thresholds to determine significant DGEs between M and nM states. Based on expression patterns (i.e., condition value), DEGs were categorized as upregulated (positive condition value) or downregulated (negative condition value) in M or nM state. Furthermore, we performed gene ontology (GO) enrichment analyses using the CORNA R package (Wu and Watson, 2009), with Fisher’s exact and hypergeometric tests to assign contigs to a specific GO and/or KEGG term. These analyses enabled us to predict molecular functions and related biological processes from gene expression results. For statistical significance, alpha was set at 0.05.

#### Gene Expression Pattern and Relationships

We used the Jonckheere-Terpstra-Kendall (JTK, R package) analysis to identify cyclic genes and therefore a daily cycle, as shown by RNA-Seq data. The JTK test is a non-parametric test, which uses Kendall’s tau, a measure of rank correlation to measure the association between two measured quantities. The algorithm used in JTK_CYCLE derives optimal combination of period and phase from cosine curves between experimental groups’ time series data. The JTK_CYCLE algorithm R script offers a statistically accurate identification and characterization of cycling transcripts (Hughes et al., 2010). We used data from all six time points over 24 h to compare differences in expression patterns of genes between M and nM states.

#### The Network Analysis

We performed weighted gene coexpression network analysis (WGCNA) in R for an unbiased assessment of transcriptional networks of all 11,246 genes associated with M and nM physiological states (Zhang and Horvath, 2005; Langfelder and Hovarth, 2008). The WGCNA can be considered a step-wise data reduction technique, which (a) starts from the level of thousands of variables (e.g., gene expression profiles), (b) identifies biologically interesting modules based on a node significance measure, (c) represents modules by their centroids (e.g., eigenvectors or intramodular hubs), (d) uses intramodular connectivity (kIM or kME) as quantitative measures of module membership, and (e) combines node significance and module membership measures for identifying significant hub nodes. The module centric analysis alleviates multiple testing problems inherent in high dimensional data, for example, gene expression data. Thus, although similar to other clustering methods in the sense that it also calculates a distance metric between expression patterns of all genes, WGCNA is a better analysis as it takes into account the complexity of the distance metric. The WGCNA starts with simple correlation values (usually Pearson’s) between all pairs of genes. Later, the correlations are transformed into an adjacency matrix by raising the correlations to a soft-thresholding power function β. The parameter β is chosen based on the data set to achieve an approximate scale-free network (Zhang and Horvath, 2005) and favors strong correlations over weak correlations. Adjacencies are next transformed into a topological overlap matrix (Zhao et al., 2010), which as a similarity measure can be subtracted from 1 to give a distance measure. These distance measures are then used in traditional hierarchical clustering to represent the relationships among genes in a familiar dendrogram. The next step in a WGCNA analysis is to break genes into clusters or “modules.” There are many different methods of cutting a dendrogram, and WGCNA suggests a computational approach called Dynamic Branch Cut (Langfelder et al., 2008). Here, we performed WGCNA in the R package (Langfelder and Hovarth, 2008) on the number of annotated transcripts that had a *p*-value < 0.001 in at least one of the experimental contrasts. There are many different parameter choices at each step in the process. After assessing a range of soft-thresholding values, we chose power β = 8. We calculated Pearson correlation coefficients between all pairs of probes in one block on a laptop computer with 64-bit Windows and 4 GB of RAM. We chose to use a signed adjacency and signed topological overlap matrix to preserve differences between positive and negative correlations. Average linkage hierarchical clustering was used and modules were determined using the Dynamic Hybrid method with deepSplit = 2 and a minimum module size = 30. A second Partitioning Around Mediods-like stage of module detection was done with pamRespectsDendro = TRUE. At the end, modules with similar expression patterns were merged at mergeCutHeight = 0.2. Otherwise, default values of blockwise module functions were used. Once modules have been defined, an average expression profile of all genes in the model can be determined by calculating an eigengene value for each treatment group by taking the first principal component of gene expression values in the module. Relatively higher and lower expression values are represented by positive and eigengene values, respectively. The set of eigengene values can be taken as a proxy for an average expression pattern of all genes in a module (Figure 5A). The total number of annotated transcripts × 12 samples data matrix has been now reduced down to six modules × two groups.

The WGCNA analysis defined highly connected intramodular hub genes as module centroids and identified coexpressed gene groups (modules) that were altered between the M state and the subsequent nM state (for details see Supplementary Data Sheet 1). Briefly, bi-weighted mid-correlations were calculated for all gene pairs, and a signed similarity matrix was created, in which gene similarity was reflected by a correlation sign. The signed similarity matrix was raised to power β to emphasize a strong correlation, therefore, reducing the emphasis of a weak correlation, in an exponential scale. The resultant adjacency matrix was then transformed into a topological overlap matrix (Li and Horvath, 2007). We chose a threshold power of nine (smallest threshold that resulted in a scale-free *R*^2^ fit of 0.8) and created consensus networks that calculated component-wise minimum values for topologic overlap (TO), and genes were hierarchically clustered using 1 - TO (dissTOM) as the distance measure. A dynamic tree-cutting algorithm (cutree Hybrid) assigned initial modules using default parameters but with a few modifications (i.e., deepSplit = 4, cutHeight = 0.999, min Module size = 40, dthresh = 0.25, and pamStage = FALSE). These coexpressed gene modules were used to calculate module eigengenes (MEs; or 1st principal component of the module), with reference to a given module of photoperiod-induced M or nM states. A module hub was defined by module membership (kME) values, which are calculated values of Pearson’s correlation coefficient between gene and ME; genes with kME < 0.7 were excluded from the module. The iGraph package was used for network visualization (Csardi and Nepusz, 2006).

## Results

### Differences in Seasonal Phenotypes

**FIGURE 1:**
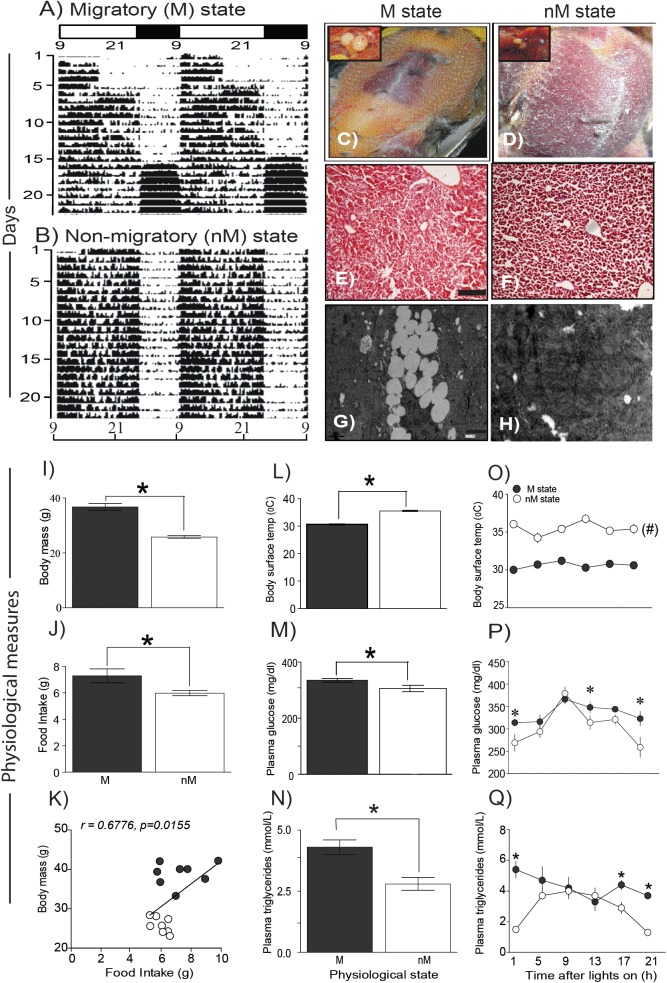
Phenotypic and physiological measures in migratory and non-migratory seasonal states. **(A**,**B)** representative actograms plotted on clock hours (L_on_ 09:00 am; L_off_ 01:00 am) under photostimulated migratory (M) and photorefractory non-migratory (nM) states. **(C**,**D)** show fat deposition in M and nM states, respectively, along with relative testis size in the left top inset. Similarly, histological details in H-E stained simple microscopy, scale bar 200 μm **(E,F)** and transmission electron microscopy of liver sections are shown, scale bar 1 μm **(G**,**H)** under M (left) and nM (right) states. Note, lipid laden liver cells in the M state **(G)**. **(I–K)** mean (±SEM) body mass and daily food intake and correlation of food intake on body mass under M and nM states. **(L–N)** mean (±SEM) basal surface body temperature, blood glucose and triglycerides levels. Also, shown are 24-h changes in surface body temperature **(O)** and blood glucose **(P)** and triglycerides levels in two states **(Q)**. All data points are mean ± SEM for six birds. Student’s *t*-test compared M and nM states, if it involved one time-point. Furthermore, we used one-way ANOVA to determine the significant difference over 24-h, and two-way ANOVA to test if the effect was dependent on the physiological state. Bonferroni post-test was used to compare two states, if two-way ANOVA indicated a significant difference. An asterisk (^∗^) indicates a significant difference at particular time-point(s) and # shows if the difference was significant at all the time points between M and nM states. For significance, alpha was set at 0.05.

Buntings differed in behavior and physiology between the states (Figure 1). In the M state, they exhibited nocturnal *Zugunruhe* and overall increased 24-h activity, and in the nM state, they exhibited only daytime activity and thus lower 24-h activity (Figures 1A,B). In addition, in the M state, there was a significantly higher FI, body mass, blood glucose, and triglycerides, as compared with the same variables in the nM state (*p* < 0.05; Student’s *t*-test; Figures 1I–K,M,N). Conversely, baseline surface body temperature (mean of six temperature measurements over 24 h) was significantly higher in the nM state than in the M state (Figure 1L). Overall, we found a significant positive correlation between FI and body mass (r = 0.68, *p* = 0.016; Figure 1K). Furthermore, there was a difference in liver histology between M and nM states; liver cells were found laden with accumulated fat in the M state (Figures 1E–H).

Interestingly, blood glucose and triglycerides levels were high for most of the day in the M state, whereas surface body temperature was high throughout the 24-h period in the nM state (*p* < 0.05, Bonferroni posttest; Figures 1O–Q). Furthermore, in the M state but not in the nM state, there were significant 24-h variations in blood glucose (*F*_5,23_ = 4.47, *p* = 0.0008; h^2^ = 0.554) and triglyceride levels (*F*_5,23_ = 9.248, *p* = 0.0002, h^2^ = 0.720; 1-way ANOVA) and a significant 24-h rhythm (as shown by cosinor analysis) (Figures 1P,Q). Conversely, surface body temperature showed a significant 24-h variation in the M state only (*F*_5,53_ = 9.248, *p* = 0.0092, h^2^ = 0.266; 1-way RM ANOVA; Figure 1O). Overall, there was a significant effect of the seasonal state on glucose level (*F*_1,36_ = 8.024, *p* = 0.0075; h^2^ = 0.182), triglycerides level (*F*_1,36_ = 19.22, *p* < 0.0001; h^2^ = 0.348), and surface body temperature (*F*_1,102_ = 358.0, *p* < 0.0001; h^2^ = 0.778). Time of day affected only glucose (*F*_5,36_ = 6.158, *p* = 0.0075; h^2^ = 0.461) and triglycerides (*F*_5,36_ = 2.589; *p* = 0.05; h^2^ = 0.241) levels. Furthermore, the effect of the seasonal state was found dependent on the time of day on levels of triglycerides (*F*_5,36_ = 3.61, *p* = 0.0095; h^2^ = 0.334) and body temperature (*F*_5,102_ = 3.188, *p* = 0.0103; h^2^ = 0.135) but not on glucose (*F*_5,36_ = 1.072, *p* = 0.3921; h^2^ = 0.130) levels (2-way ANOVA; Figures 1O–Q).

### Differences in Gene Expression Between Seasonal States

**FIGURE 2:**
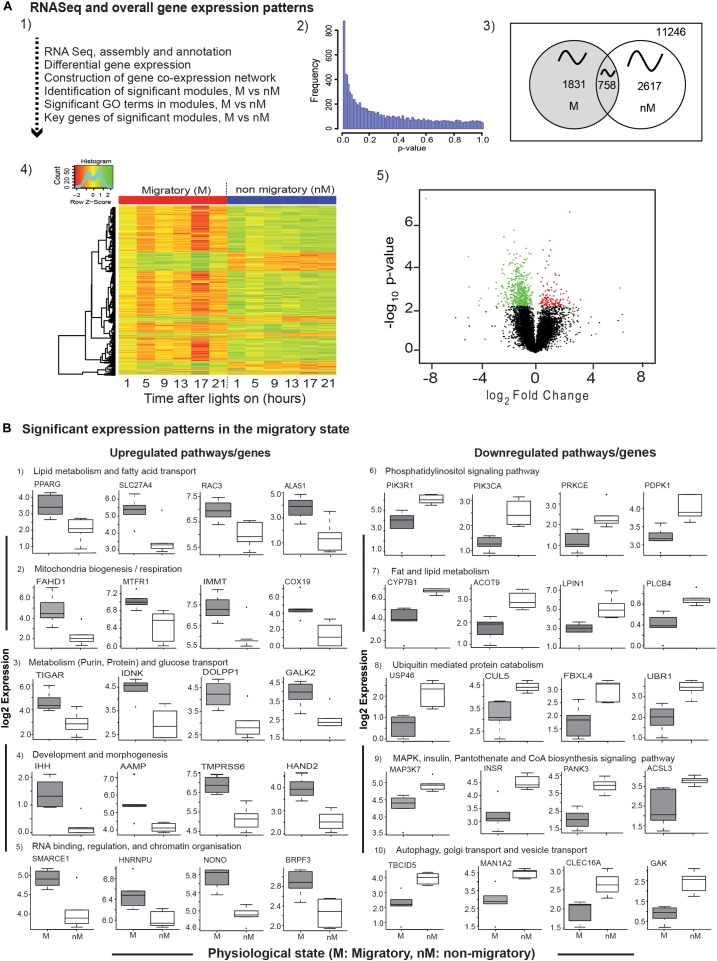
Transcriptome-wide differential gene expression analyses. Top panel (**2A1–A5**): The flow-chart outlines steps of RNA sequencing, differential gene expression (DGE) analysis, and coexpression network analysis **(A1)**; the distribution of nominal p-values from linear regression model employed to detect differential expression between photostimulated migratory (M) and photorefractory non-migratory (nM) states **(A2)**; Venn diagram showing number of cyclic genes in M and nM states, as well as common to both states oscillating with an approximately 24-h period **(A3)**; heatmap of 569 differentially expressed genes in two states (FDR < 0.1), in which scaled expression values are color coded; green- upregulated; red – downregulated **(A4)**, and volcano plot showing the log_2_fold change and negative log_10_ nominal p-value for all the expressed genes, in which green and red circles represent downregulated and upregulated genes in the M state, as compared with the nM state **(A5)**. Bottom panels **(B)**: boxplots of significantly upregulated (left half; **B1–B5**) and downregulated (right half; **B6–B10**) genes included in a specific pathway, in the M state as compared with the nM state. Each boxplot is log_2_FPKM expression value in 5–95% intervals (outliers are shown dots). Wilcox Rank sum test was used to determine the significant change. For significance, alpha was set at 0.05.

A total of 11,246 expressed sequenced tags (ESTs) with *p*-value ≤ 1 were assembled, mapped with reference to zebra finch genome, and analyzed to find DEGs, gene coexpression network, significant GO terms in modules, and key genes consequently (Figures 2A1,A2). These included a total of 4448 genes exhibiting daily oscillation, with 1831 and 2617 genes in M and nM states, respectively; 758 genes were common to both states (Figure 2A3). Furthermore, we found 569 DEGs (FDR < 0.1; Figures 2A4,A5 and Supplementary Table S2) with 131 upregulated and 438 downregulated genes in the M state, as compared with the nM state (FDR < 0.1, Figures 2A4,A5 and Supplementary Table S2). A time-course heat map also shows this and indicates a 24-h variation in DGEs (Figure 2A4).

To evaluate transcriptional signatures in the liver, we identified pathways enriched with genes implicated in various biological processes. We found upregulated pathways enriched with genes associated mainly with fatty acid transport, lipid synthesis and regulation (*PPARG*, *SLC27A4*, *RAC3*, *ALAS1*; Figure 2B1), mitochondrial biogenesis and respiration (*FAHD1*, *MTFR1*, *IMMT*, *COX19*; Figure 2B2), glucose transport, purine-, protein-, and carbohydrate- metabolism (*TIGAR*, *IDNK*, *PPARG*, *GALK2*; Figure 2B3), development, angiogenesis and matrix remodeling pathways (*IHH*, *AAMP*, *TMPRSS6*, *HAND2*; Figure 2B4), RNA binding, regulation, and chromatin organization (*SMARCE1*, *HNRNPU*, *NONO*, *BRPF3*; Figure 2B5). Similarly, downregulated pathways were found to be enriched with genes associated mainly with phosphatidylinositol signaling (*PIK3R1*, *PIK3CA*, *PRKCE*, *PDPK1*; Figure 2B6), lipid and fat metabolism (*CYP7B1*, *ACOT9*, *LIPIN1*, *PLCB4*; Figure 2B7), ubiquitin mediated protein catabolism (*USP46*, *CUL5*, *FBXL4*, *UBR1*; Figure 2B8), MAPK signaling pathway (*MAPK37*, Figure 2B9), pantothenate and CoA biosynthesis signaling pathways (*INSR*, *PANK3*, *ACSL3*; Figure 2B9), autophagy, Golgi transport, and vesicle transport (*TBCID5*, *MAN1A2*, *CLEC16A*, *GAK*; Figure 2B10).

### Coexpression Network Analysis

**FIGURE 3:**
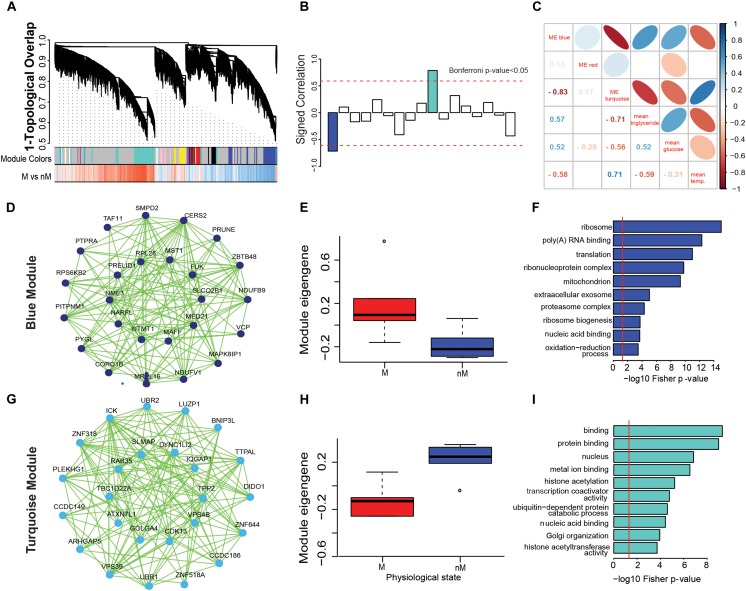
Gene coexpression networks associated with different physiological states. **(A–H)**: Weighted gene coexpression network analysis (WGCNA) to show modular hub genes and pathways. **(A)** the hierarchical clustering of genes based on 1-topological overlap (1-TO) distance plotted with signed coexpressed modules. **(B)** signed correlation of module eigengenes (first principal component of the module) with physiological states, with reference to increased (+ve values) and decreased (-ve values) coexpression patterns in the refractory non-migratory (nM) state. Horizontal dotted red lines indicate threshold significance, as determined by Bonferroni correction. **(C)** pearson’s correlation of module eigengenes with physiological measures (blood glucose and triglycerides levels and surface body temperature). **(D,G)** show the coexpression network of first 25 hub genes that were upregulated in M (migratory: blue module, **D**) and nM (turquoise module, **G**) states. In the inner circle of each module lie genes that showed highest correlation with a module eigengene value. **(E,H)** show boxplots of relative module eigengene expression between M and nM states in blue **(E)** and turquoise **(H)** modules, with significance determined by Wilcox Rank sum test (the outlier values are shown by a dot). **(F,I)** are the gene ontology term enrichment of blue and turquoise modules, respectively. For significance, alpha was set at 0.05.

The WGCNA created gene modules and identified two significant modules from 17 generated modules based on shared similar gene expression patterns (Bonferroni- correction *p* < 0.05; Figures 3A,B). Within each module, a network of the top 25 genes with shared expression patterns (i.e., significantly upregulated in a state) was identified as blue module (M state: *p* = 0.015; Figures 3D,E) or turquoise module (nM state: *p* = 0.0043, Wilcox test; Figures 3G,H). Gene ontology terms highlight biological processes that are enriched in a set of genes in a state (Figures 3F,I). In particular, the blue module was found to be enriched in a diverse number of significant GO terms involved in processes like ribo-nucleoprotein complex, mitochondria, ribosome biogenesis, oxidation reduction process, metabolic process, fatty acid metabolism, and ATP binding (*p* < 0.0001; Fisher test, Supplementary Table S3). Similarly, the turquoise module was found to be enriched in GO terms involved in the ubiquitin-dependent protein catabolic process, Golgi organization, histone acetyltransferase activity, protein phosphorylation, protein kinase activity, and MAPK activity (*p* < 0.0001; Fisher test, Supplementary Table S3).

In order to better understand the physiological relevance of the significant modules, we determined the correlation of physiological parameters (blood glucose and triglycerides levels and surface body temperature) with the eigengene of each module. The eigengene of the blue module (M state) showed a positive correlation with blood glucose (r = 0.5) and triglycerides (r = 0.57) levels and negative correlation (r = -0.58) with surface body temperature (Figure 3C). Conversely, there was a positive correlation between the eignegene of the turquoise module (nM state) with surface body temperature (r = 0.7) and a negative correlation with blood glucose (r = -0.55) and triglycerides (r = -0.7) levels (Figure 3C).

### WGCNA Derived Physiological State Specific Coexpressed Hub Genes

Of the top 25 hub genes that were included in modules, we found significant differences in FPKM log_2_ expression values of 16 genes in the blue module and 21 in the turquoise module (*p* < 0.05; Wilcox test, Supplementary Figure S1). The blue module included genes involved in molecular processes mediated by ribosomal proteins (*RPS6KB2*, *RPL26*; Supplementary Figure S1A), mitochondrial respiration and membrane protein (*NDUFB9*, *PRELID1*, *MRPL16*; Supplementary Figure S1A), cell proliferation, growth and differentiation (*CORO1B*, *PRUNE*; Supplementary Figure S1A), vesicle transport (*VCP*; Supplementary Figure S1A), glycogen storage, glycolipid synthesis, sphingolipid metabolism (*PYGL*, *FUK*, *CERS2*; Supplementary Figure S1A), phosphatidylinositol, MAPK pathway (*PITPNM1*; Supplementary Figure S1A), transcription factor and regulation (*MED21*, *MAFF*, *ZBTB48*; Supplementary Figure S1A), and hepatic growth factor (*MST1*; Supplementary Figure S1A).

The turquoise module hub gene network included genes that were found associated with ubiquitin and proteolytic pathways (*UBR2*, *TPP2*; Supplementary Figure S1B), mitochondrial protein catabolic process (*BNIP3L*; Supplementary Figure S1B), protein transporter and anti-apoptotic activity (TTPAL, ICK; Supplementary Figure S1B), transcriptional regulation (*IZNF318*, *ZNF644*, *ZNF318*, *CDK13*; Supplementary Figure S1B), smad pathway (*VPS39*; Supplementary Figure S1B), retrograde transport at *trans*-Golgi network (*RAB35*, *GOLGA4*, *SLAMP*; Supplementary Figure S1B), Rho GTPase activity (*IQGAPI*, *ARHGAP5*, *TBC1D22A*, *PLEKHG1*; Supplementary Figure S1B), and peripheral protein coding genes (*ATXN7LI*, *CCDC149*, *CCDC186*; Supplementary Figure S1B).

#### Cyclic Transcripts: Circadian Genes and Epigenetic Modifiers

**FIGURE 4:**
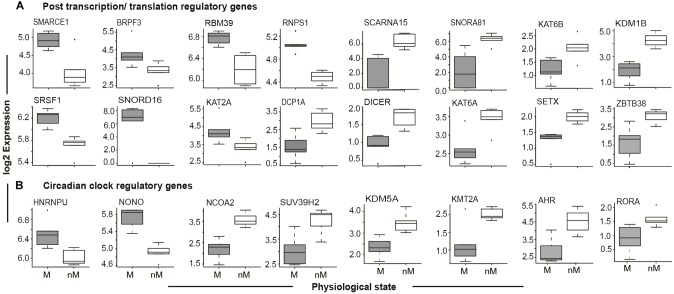
Seasonal state-specific differences in gene expressions. Boxplot of log_2_ FPKM expression values in 5–95% intervals of post transcription/translation regulatory genes (top panel, **A**) and circadian clock regulatory genes (bottom panel, **B**). The outliers are shown as dot in each figure. Wilcox Rank sum test was used to test the significant difference between migratory (M) and non-migratory (nM) states. For significance, alpha was set at 0.05.

Given the role of circadian rhythms in the regulation of photoperiodic seasonal states in blackheaded buntings (Singh et al., 2015; Trivedi et al., 2015; Mishra et al., 2017a,b), we identified genes that showed 24-h oscillations. These cyclic genes included *PER2*, *CRY1*, *CRY2*, *ARNTL* (*BMAL1*), *CLOCK*, and *NPAS2*, which are known to be the core genes of circadian timekeeping (Figure 4B and Supplementary Table S4; JTK analysis). Additional upregulated circadian timekeeping genes included *HNRNPU* (heterogeneous nuclear ribonucleoprotein) *NONO* (Non-POU domain containing Octamer-binding), and *RORA* (retinoid related orphan receptor-alpha) that are involved in the regulation of *ARNTL/BMAL1*. Similarly, *NCOA2*, which acts as a transcriptional coactivator for the CLOCK-ARNTL/BMAL1 heterodimer complex, was upregulated in the nM state (Figure 4B). Upregulated epigenetic modifier proteins in the nM state included histone methyltransferase (*SUV39H2*, *KMT2A*; Figure 4B) and histone demethylase (*KDM5A*), and aryl hydrocarbon receptors (*AHR*s), a ligand-activated helix-loop-helix transcription factor, which functions as a regulator of xenobiotic metabolizing enzymes in the liver (Figure 4B). In the nM state, we also found upregulated genes that encode epigenetic regulators at post-transcription and translation levels and included chromatin modifiers with acetyl transferase and demethylase activities (*SMARCE1*, *BPRPF3*, *KAT2A*, *KAT6B*, *KDM1B*), RNA processing and metabolism (*RBM39*, *RNP81*, *SRSF1*, *DCPIA*, *SETX*), transcriptional repression in a methyl dependent manner, and endonucleases (*ZBTB38*, *ZC3H12C*; Figure 4A and Supplementary Table S2). Additionally, *DICER* and small nucleolar Cajal body specific RNA genes (*SNORD16*, *SNORNA81*, *SCARNA15*), which serve as important post-transcriptional regulators in miRNA biogenesis and non-coding RNA dependent regulation of transcription were upregulated in the nM state (Figure 4A).

#### Key Signaling and Metabolic Pathways

**FIGURE 5:**
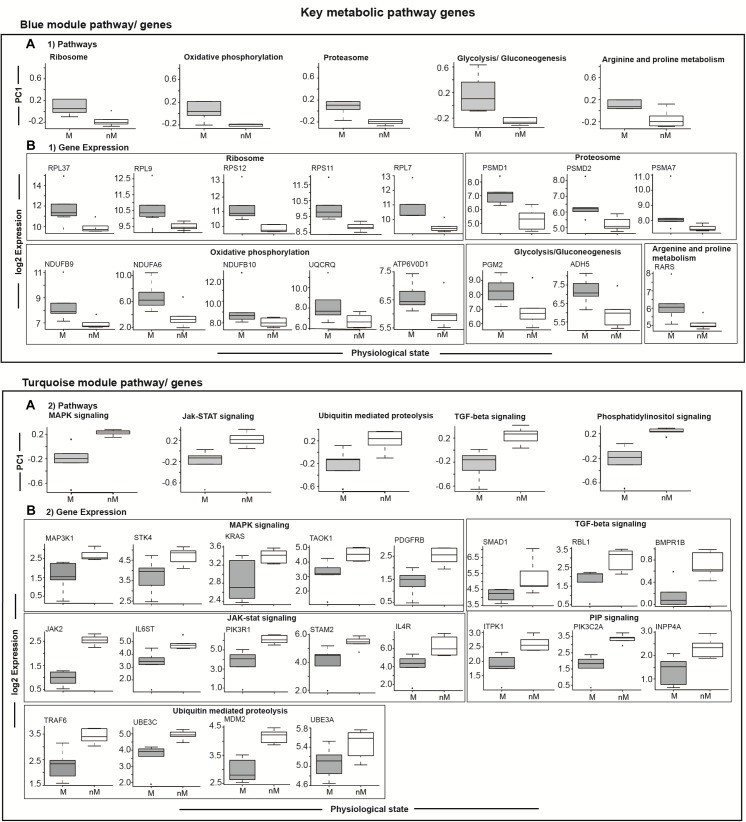
Module specific selected relevant pathways and candidate genes. Top panel: **(A1)** shows differences in gene expressions comprising a particular pathway in the blue module, and in underneath boxed 2 panels **(B1)** are the log_2_ FPKM expression values of coexpressed key upregulated genes enriched in different GO terms showing 5–95% intervals. Bottom panel: Boxplots in the top row show differences in gene expressions comprising a particular pathway in the turquoise module **(A2)**, and in the three panels underneath **(B2)** are the log_2_ FPKM expression values of coexpressed key upregulated genes enriched in different GO terms showing 5–95% intervals. All plots compare migratory (M) and non-migratory (nM) states, and an outlier is shown as dot in each plot. Wilcox Rank sum test was used to determined significant difference between M and nM states. For significance, alpha was set at 0.05.

To highlight differences in the key signaling and metabolic pathways between M and nM states, we plotted module eigengenes (Figure 5). Compared with the nM state, genes with a significantly higher expression in the M state were categorized as contributing to ribosome biogenesis, oxidative phosphorylation, arginine and proline metabolism, proteasome, glycolysis, and gluconeogenesis of the blue modulatory pathways/genes (*p* < 0.05, Fisher test; Figure 5A1). Similarly, genes with a significantly lower expression in the M state were categorized as contributing to various pathways, including MAPK signaling, JAK-STAT signaling, ubiquitin-mediated proteolysis, TGF-beta signaling, and phosphatidylinositol signaling pathways of the turquoise modulatory pathways/genes (*p* < 0.05, Fisher test; Figure 5A2).

#### Transcriptional Signatures of the Seasonal Physiological State

To evaluate transcriptional signatures of seasonal state in the liver, we identified modules with genes associated with various biological processes. Upregulated gene clusters were found enriched in genes associated mainly with ribosome biogenesis (*RPL37*, *RPL9*, *RPS12*, *RPS11*, *RPL7*), oxidative phosphorylation (*NDUFB9*, *NDUFA6*, *NDUFB10*, *UQCRQ*, *ATP6V0D1*), proteosome function (*PSMD1*, *PSMD2*, *PSMA7*), glycolysis and gluconeogenesis (*PGM2*, *ADH5*), and arginine and proline metabolism (RARS) in the pathways/genes of the blue module (Figure 5B1). Similarly, the downregulated gene clusters were found enriched in genes associated mainly with MAPK signaling (*MAP3K1*, *STK4*, *KRAS*, *TAOK1*, *PDGFRB*), JAK-STAT signaling (*JAK2*, *IL6ST*, *PIK3R1*, *STAM2*, *IL4R*), ubiquitin mediated proteolysis (*TRAF6*, *UBE3C*, *MDM2*, *UBE3A*), TGF-beta signaling (*SMAD1*, *RBL1*, *BMPR1B*; Figure 5B2), and PIP signaling pathway (*ITPK1*, *PIK3C2A*, *INPP4A*) in the pathways/genes of the turquoise module (Figure 5B2).

## Discussion

We demonstrate concomitant seasonal changes in behavior and physiology and gene expression in a photoperiodic migrant species. Results from this study reveal differences in physiology, particularly in changes associated with increased energy demands of migration, between photostimulated M and photorefractory nM states in buntings. Consistent with greater FI, fat deposition, and plasma levels of glucose and triglycerides, we found structural changes in the liver between M and nM states. For example, hepatocytes were laden with lipid droplets in significant amounts in the M state but not in the nM state, indicating fat-fuel storage in the liver. This also suggested that the liver was the major site of metabolism and energy homeostasis, and it undergoes drastic changes with transition in photoperiodically regulated seasonal states. Most intriguingly, however, surface body temperature was significantly higher in the nM state than the M state, although buntings exhibited intense nocturnal *Zugunruhe* and had increased overall daily activity in the M state. This, we suggest, was the result of an increased thermogenesis in photorefractory buntings, consistent with reported correlation of thermogenesis with increased liver metabolism in mammals and migratory finches (Swanson and Olmstead, 1999; Williams and Tieleman, 2000; Villarin et al., 2003). The liver contributes to thermogenesis by about 25% from its mitochondria-regulated energy metabolism even when animals are at their basal metabolic rates (Coutre and Hulbert, 1995; Brand et al., 2003; Villarin et al., 2003; Else et al., 2004).

Striking differences between M and nM phenotypes provide strong support to the results of transcriptome-wide gene expressions in blackheaded buntings. Overall, transcriptome data suggest that there were transcriptional and post-transcriptional modifications in the liver for metabolic homeostasis, as required seasonally with the development and cessation of migratory state in blackheaded buntings. We identified candidate genes and the pathways these genes enrich to meet the metabolic needs of M and nM states. In the photostimulated M state, we found 131 upregulated genes associated with mitochondrial aerobic respiration, organization, and fission, ribonucleoprotein complex, glucose transport and carbohydrate metabolism, fatty acid transportation and lipid metabolism, cell cycle and apoptosis, protein ubiquitination and purine metabolism, transcriptional regulation, bone growth, morphogenesis, cardiac morphogenesis, angiogenesis, and platelet aggregation. At the same time, many of the 438 downregulated genes that we found were associated with protein binding, histone acetylation, methyltransferase, transcription coactivator, ubiquitin-dependent protein catabolic process, Golgi organization, protein phosphorylation, and fat, lipid, and energy metabolism. In the photorefractory nM state, on the other hand, we found DEGs associated with protein catabolism, suggesting a metabolic shift from anabolism to catabolism after migration and reproduction have been completed. Particularly, *GNAS* and *ADCY2* genes were highly expressed in the photorefractory nM buntings with higher surface body temperature, consistent with the suggested roles of these two genes in energy metabolism and lipid clearance via cAMP-dependent pathway in mouse (Yu et al., 2000; Chen et al., 2004).

Our results revealed significant differences in cell components, suggesting seasonal changes in liver cell metabolism between the M state and the nM state. For example, genes that enriched ribonucleoprotein complex suggested changes in ribosome biogenesis, which is an indicator of the cellular translational capacity. The other genes that enriched mitochondrial activity suggested an energy-influx resulting from coenzyme A-mediated lipid metabolism (Leonardi et al., 2005). Similarly, genes that enriched folate biosynthesis and estrogen metabolism pathways were downregulated in the M state, and this probably accounted for fat deposition required as flight fuel. These genes might act in the liver by reducing the oxidation of fatty acid, which in turn would lead to the export of triglycerides and the protein catabolism. Alternatively, these genes might act via the modulation of the dietary requirement for choline in the cells of the liver (Zeisel, 2006). The other pathway important in the regulation of lipid metabolism in hepatocytes may be TGF-β/smad signaling interactive pathway, which has been known to influence lipid accumulation and triglyceride levels in hepatocytes (Yang et al., 2014). Similarly, we found suppressed and enhanced expression of genes involved in synthesis and breakdown of lipids in the nM state. In particular, *ACSL3* (acetyl CoA synthetase 3 enzyme) involved in hepatic lipogenesis was downregulated, suggesting an attenuated lipid metabolism. Consistent with this, *ACOT9* (coding for Acyl-CoA thioesterase 9 enzyme) involved in the hydrolysis of acyl-CoA to free fatty acids and coenzyme A, and *PANK3* (coding for pantothenate kinase 3 enzyme) involved in the regulation coenzyme A biosynthesis, were also differentially expressed in the nM state.

Notably, buntings with fatty liver in the M state did not appear to show any symptom of a pathophysiological state, like non-alcoholic fatty liver disease (NAFLD; Corbin and Zeisel, 2012). We speculate that this was due to the genetic defense during the photostimulated M state, as indicated by the module – over representation of most abundant gene expressions. The module shows an increased expression of at least two genes that may be a part of defense in buntings from developing a metabolic syndrome during the migratory state. Both, *SMPD2*, which is potentially responsible for lipidosis (a lipid storage disorder in mammals), and *CERS2*, which encodes a protein associated with cancer growth suppression and involved in sphingolipid synthesis, were increased in expression during the photostimulated M state. In the same way, the enhanced expression of TGF-β signaling genes suggests an increased energy demand for the maintenance of long activity hours in photorefractory nM birds. In fact, TGF-β signaling has been demonstrated as a key regulatory pathway for energy homeostasis in mammals (Yadav et al., 2012). The upregulated ubiquitin pathway may also be important to achieve bulk degradation of skeletal muscle proteins in buntings during the nM state, as reported during fasting and metabolic acidosis in mammals (Lecker et al., 1999). We speculate that the ubiquitin pathway contributes to protein catabolism associated with high surface body temperature in photorefractory buntings. Protein turnover via ubiquitin pathway metabolism could be a physiological adaptation to energy homeostasis, as possibly required during the nM state in blackheaded buntings. Thermoregulatory roles of protein degradation and protein turnover have been suggested in mammals (Ballard, 1977).

There was differential expression of genes that enriched different biological processes, such as miRNA biogenesis, RNA metabolism and stability, and histone demethylation and deacetylation, in buntings between the M state and the nM state. This is shown by differences in the expression patterns of epigenetic modifiers including post-transcriptional regulators (*SNORD16*, *DICER1*), lysine methyltransferases and demethylases (*KMT2A*, *KDM7A*, *KDM5A*), ATP-dependent chromatin remodeling complex (*SMARCE1*), RNA-binding proteins (*HNRNPU*, *NONO*), ligand-activated transcriptional activators (AHR), and nuclear receptor coactivators (*NCOA2*). *SNORD*s (C/D box small nucleolar RNAs) are involved in alternative splicing, cholesterol traffic, production of microRNAs (miRNAs), and lipid toxicity (Falaleeva and Stamm, 2012). Similarly, *DICER1* is the post-transcriptional regulator of specific miRNA-dependent circadian regulation of lipogenic and fatty acid metabolic genes (Fernández-Hernando, 2013). The downregulation of these genes indicated their possible roles in miRNA-mediated gene regulation of seasonal changes in the metabolism of the liver during the nM state. Similarly, buntings in the M state had low expression of *CSNK1D*, which is involved in post-translational regulation in livers of mice (Etchegaray et al., 2009). Differences in the abundance of *DICER 1*, *NCOA2*, *AHR*, *KDM5A*, *CSNK1D*, *KDM7A*, and *KMT2A* might indicate seasonal alterations in circadian oscillations with reduced amplitudes in the M state. The core clock gene 24-h oscillations indeed show changes in amplitude and phase concurrently with photoperiod-induced seasonal transition in behavior and physiology in blackheaded buntings (Singh et al., 2015). We also found in the transcriptome of the liver, 24-h cyclicity of all core clock genes (*PER2*, *CRY1*, *CRY2*, *ARNTL* (*BMAL1*), *CLOCK* and *NPAS2*) and clock-controlled genes (*HNRNPU*, *NONO*, *RORA*, and *NCOA2*) that comprise the transcriptional – translational feedback loop of circadian timekeeping. Although, because of sequencing cost and volume of obtained results for the analyses, we only show results on cyclic genes based on one sample per time point per physiological state, the overall 24-h cyclic expression pattern is consistent with qPCR results of some of these genes from another study on blackheaded buntings (Singh et al., 2015). Furthermore, *SFPQ* gene that encodes a component of the PER complex in mammals was upregulated in bunting liver during the M state; the same gene expression was, however, found to be downregulated in the hypothalamic transcriptome of migratory Swainson’s thrushes (Johnston et al., 2016). Considered together, *SFPQ* gene expression shows changes during different seasonal states but with differences in the expression pattern between peripheral (liver) and central (hypothalamus) tissues. However, it cannot be known from this study how cycling genes play a role in the regulation of seasonal states and associated nutrient metabolism and energy homeostasis in migratory birds. Nonetheless, the circadian regulation of plasma glucose, triglyceride, and many other hormones and the nutritional effect on circadian clock regulated functions has been shown in mammals (Hatori et al., 2012; Eckel-Mahan et al., 2013).

We provide, for the first time, transcriptome-wide evidence for the molecular underpinnings of seasonal metabolic adaptations in a latitudinal migratory songbird. Gene enrichment and network analyses of DEGs revealed a vital role of the liver in meeting the metabolic demands of different seasonal physiological states during the year in migrants. We also suggest seasonal state dependent molecular changes in the liver as the ‘liability’ variable that perhaps triggers and maintains the physiology of birds during migration and postmigration, consistent with the theoretical ‘threshold’ model of Pulido (2011). This model envisages the threshold of a ‘liability’ variable (a protein or hormone, for example) as the determinant for the development of the migratory behavior. In fact, a strong correlation of WGCNA derived specific genes and regulatory pathways with changes in behavior and physiology further highlighted this. Overall, we demonstrate the molecular underpinnings of metabolism and energy homeostasis, which form a significant part of the broader temporal adaptability of species living in the seasonal environment with limited food resources. Furthermore, these results give significant insights into how animals that show metabolic syndrome akin to obesity and diabetes (increased FI, high fat, and become obese with high glucose levels in migratory state and revert to normal levels in the subsequent non-migratory state) for several weeks each year may not develop high-risk diseases, for example diabetes and cardiac failures. Perhaps, future studies on such non-model migratory species could provide vital clues on the molecular switches that regulate metabolism and energy homeostasis in higher vertebrates and humans.

## Data Availability

The sequence data are available in the NCBI Gene Expression Omnibus repository and are accessible through GEO accession number #GSE104748.

## Ethics Statement

The experiments was carried out at the Department of Zoology, University of Lucknow (Lucknow, India), in accordance with the guidelines of the Institutional Animal Ethics Committee (IAEC).

## Author Contributions

VK conceived the idea and designed the experiments. DS carried out the experiments and sampling. HL performed RNA Sequencing on the Illumina platform. DS and VS organized the data and performed analyses. DS and VK wrote the manuscript.

## Conflict of Interest Statement

The authors declare that the research was conducted in the absence of any commercial or financial relationships that could be construed as a potential conflict of interest.

## Footnotes

1 https://bioinf.comav.upv.es/seq_crumbs/

2 http://www.bioinformatics.babraham.ac.uk/projects/fastqc/

## Supplementary Material

Figure S1 Boxplot of log_2_ FPKM expression values in 5–95% intervals of significantly coexpressed module specific hub genes (top panel, **A**) blue and turquoise modules (bottom panel, **B**). The outliers are shown as dot in each figure. Wilcox Rank sum test was used to test the significant difference between migratory (M) and non-migratory (nM) states. For significance, alpha was set at 0.05.

Table S1 Bunting liver sample transcriptome assembly and annotation statistics.

Table S2 List of differentially expressed genes under M and nM states. A gene was categorized as differentially expressed with threshold of false-discovery rate (FDR) of < 0.1 and absolute log_2_ fold change > 0.1. Based on the expression patterns (beta condition value), differentially expressed genes were categorized as upregulated or downregulated in a particular physiological state (-ve condition value: downregulated; +ve condition value: upregulated candidate genes, as compared between M and nM states).

Table S3 List of GO terms in blue and turquoise modules derived from WGCNA analysis.

Table S4 List of cycling genes under migratory and non-migratory states. A gene was characterized as cyclic with threshold of adjusted *P*-value (ADJP < 0.05). The wave characteristics of cyclic genes was categorized as period, peak expression time (Lag), and amplitude.
