# Supplementary figures and images for: Transcriptional Signatures in Liver Reveal Metabolic Adaptations to Seasons in Migratory Blackheaded Buntings
Source: Front Physiol. 2018 Nov 27;9:1568. doi: 10.3389/fphys.2018.01568 (PMC6277527; doi:10.3389/fphys.2018.01568)

Module specific hub genes

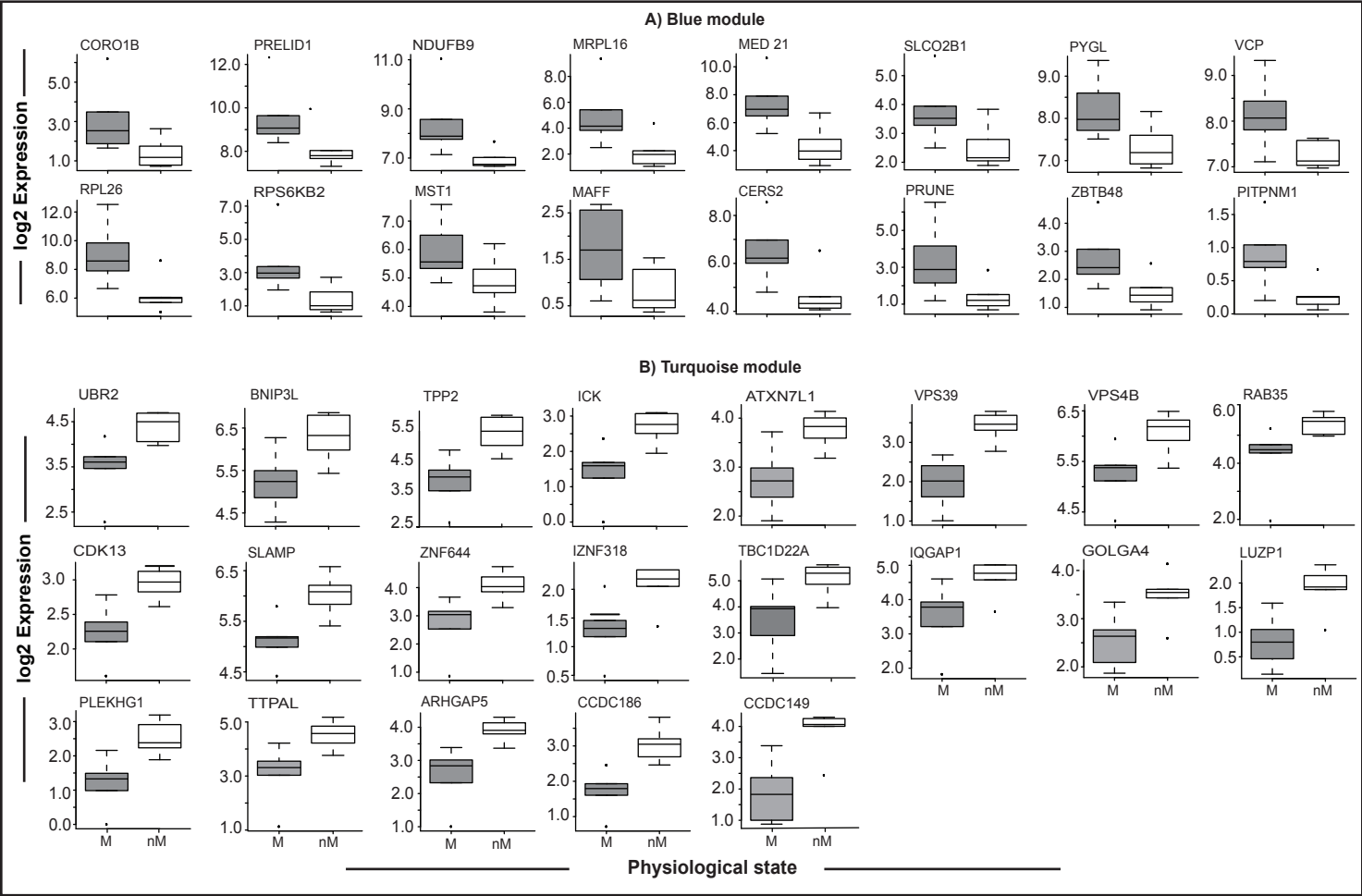

Supplement: Figure S1 — Boxplot of log2 FPKM expression values in 5–95% intervals of significantly coexpressed module specific hub genes (top panel, A) blue and turquoise modules (bottom panel, B). The outliers are shown as dot in each figure. Wilcox Rank sum test was used to test the significant difference between migratory (M) and non-migratory (nM) states. For significance, alpha was set at 0.05. [file Image_1.PDF]
