# Supplementary material for: Transcriptional Signatures in Liver Reveal Metabolic Adaptations to Seasons in Migratory Blackheaded Buntings
Source: Front Physiol. 2018 Nov 27;9:1568. doi: 10.3389/fphys.2018.01568 (PMC6277527; doi:10.3389/fphys.2018.01568)
Supplement: Table S1 — Bunting liver sample transcriptome assembly and annotation statistics. [file Table_1.DOCX]

Table 1 Bunting liver sample transcriptome assembly and annotation statistics

| Lane | Sample  Name | Sample  ID | Index | Yield | #Reads | % One Mismatch Reads (Index) | % of >= Q30 Bases (PF) | Mean Quality Score (PF) |
| --- | --- | --- | --- | --- | --- | --- | --- | --- |
| 5 | M ZT1 Liver | D1-D701-5-ATTACT | ATTACT | 1,342 | 26,323,170 | 0 | 95.33 | 35.58 |
| 5 | M ZT5 Liver | D2-D702-5-TCCGGA | TCCGGA | 1,476 | 28,936,186 | 0 | 95.13 | 35.54 |
| 5 | M ZT9 Liver | D3-D703-5-CGCTCA | CGCTCA | 976 | 19,130,262 | 0 | 79.48 | 32.78 |
| 5 | M ZT13 Liver | D4-D704-5-GAGATT | GAGATT | 840 | 16,478,582 | 0 | 95.13 | 35.54 |
| 5 | M ZT17 Liver | D5-D705-5-ATTCAG | ATTCAG | 745 | 14,606,199 | 0 | 94.47 | 35.38 |
| 5 | M ZT21 Liver | D6-D706-5-GAATTC | GAATTC | 704 | 13,795,889 | 0 | 95.24 | 35.56 |
| 5 | nM ZT1 Liver | D7-D707-5-CTGAAG | CTGAAG | 961 | 18,837,970 | 0 | 95.05 | 35.52 |
| 5 | nM ZT5 Liver | D8-D708-5-TAATGC | TAATGC | 2,175 | 42,651,754 | 0 | 95 | 35.52 |
| 5 | nM ZT9 Liver | D9-D709-5-CGGCTA | CGGCTA | 655 | 12,836,203 | 0 | 95.13 | 35.53 |
| 5 | nM ZT13 Liver | D10-D710-5-TCCGCG | TCCGCG | 351 | 6,874,818 | 0 | 95.21 | 35.55 |
| 5 | nM ZT17 Liver | D11-D711-5-TCTCGC | TCTCGC | 628 | 12,312,214 | 0 | 94.87 | 35.48 |
| 5 | nM ZT21 Liver | D12-D712-5-AGCGAT | AGCGAT | 651 | 12,769,189 | 0 | 95.17 | 35.54 |
