# Supplementary material for: Transcriptional Signatures in Liver Reveal Metabolic Adaptations to Seasons in Migratory Blackheaded Buntings
Source: Front Physiol. 2018 Nov 27;9:1568. doi: 10.3389/fphys.2018.01568 (PMC6277527; doi:10.3389/fphys.2018.01568)
Supplement: Table S2 — List of differentially expressed genes under M and nM states. A gene was categorized as differentially expressed with threshold of false-discovery rate (FDR) of < 0.1 and absolute log2 fold change > 0.1. Based on the expression patterns (beta condition value), differentially expressed genes were categorized as upregulated or downregulated in a particular physiological state (-ve condition value: downregulated; +ve condition value: upregulated candidate genes, as compared between M and nM states). [file Table_2.DOCX]

**Table 2 . List of differentially expressed genes**

Migratory state up-regulated genes

| **Ensembl Gene ID** | beta.condition | SE.condition | pval.condition | FDR_pvalue | gene_short_name | |
| --- | --- | --- | --- | --- | --- | --- |
| ENSTGUG00000002855 | 0.169775 | 0.028372 | 0.000135 | 0.024586 | CACNA1B | |
| ENSTGUG00000011702 | 0.268691 | 0.072734 | 0.004148 | 0.091652 | TMEM200A | |
| ENSTGUG00000000569 | 0.350243 | 0.082062 | 0.001642 | 0.065599 | ENSTGUG00000000569 | |
| ENSTGUG00000008727 | 0.363862 | 0.097364 | 0.003865 | 0.089615 | ENSTGUG00000008727 | |
| ENSTGUG00000011606 | 0.416579 | 0.108494 | 0.003267 | 0.085219 | CDX2 |  |
| ENSTGUG00000010455 | 0.41998 | 0.113166 | 0.004034 | 0.091086 | NOP14 |  |
| ENSTGUG00000000699 | 0.422223 | 0.078892 | 0.000323 | 0.037091 | ENSTGUG00000000699 | |
| ENSTGUG00000003181 | 0.470796 | 0.124962 | 0.003677 | 0.088944 | ENSTGUG00000003181 | |
| ENSTGUG00000007834 | 0.493114 | 0.126042 | 0.002902 | 0.082158 | FIP1L1 |  |
| ENSTGUG00000008309 | 0.494222 | 0.128471 | 0.003228 | 0.084885 | HNRNPU | |
| ENSTGUG00000006563 | 0.499639 | 0.123567 | 0.002348 | 0.076154 | ZNF503 |  |
| ENSTGUG00000008416 | 0.500248 | 0.088817 | 0.000218 | 0.029898 | SRSF1 |  |
| ENSTGUG00000008303 | 0.518848 | 0.132578 | 0.002897 | 0.082158 | ENSTGUG00000008303 | |
| ENSTGUG00000001745 | 0.533186 | 0.121165 | 0.001334 | 0.060741 | ZNF76 |  |
| ENSTGUG00000001218 | 0.535364 | 0.140376 | 0.003408 | 0.086908 | CAP1 |  |
| ENSTGUG00000011257 | 0.549964 | 0.153423 | 0.004973 | 0.098641 | MTFR1 |  |
| ENSTGUG00000003103 | 0.554656 | 0.139877 | 0.002663 | 0.078885 | FKBP6 |  |
| ENSTGUG00000002170 | 0.558984 | 0.129302 | 0.001506 | 0.064249 | RIPK1 |  |
| ENSTGUG00000003237 | 0.567044 | 0.068738 | 8.99E-06 | 0.010222 | RNPS1 |  |
| ENSTGUG00000003048 | 0.580779 | 0.115461 | 0.000514 | 0.044814 | RBM39 |  |
| ENSTGUG00000007233 | 0.585564 | 0.117369 | 0.000546 | 0.045736 | ENSTGUG00000007233 | |
| ENSTGUG00000001612 | 0.611414 | 0.160738 | 0.003464 | 0.087547 | BRPF3 |  |
| ENSTGUG00000012067 | 0.622954 | 0.154928 | 0.002435 | 0.078012 | ENSTGUG00000012067 | |
| ENSTGUG00000007130 | 0.635377 | 0.17385 | 0.004427 | 0.093822 | MYEF2 |  |
| ENSTGUG00000013057 | 0.652641 | 0.181152 | 0.004826 | 0.097077 | TMEM260 | |
| ENSTGUG00000010580 | 0.660852 | 0.181342 | 0.004505 | 0.093822 | F2 |  |
| ENSTGUG00000004842 | 0.668471 | 0.160526 | 0.001936 | 0.071371 | PACS1 |  |
| ENSTGUG00000010453 | 0.715599 | 0.155254 | 0.000966 | 0.053274 | SEC13 |  |
| ENSTGUG00000001880 | 0.72602 | 0.184134 | 0.002762 | 0.080677 | ENSTGUG00000001880 | |
| ENSTGUG00000012025 | 0.729198 | 0.19405 | 0.003736 | 0.088944 | TTLL12 |  |
| ENSTGUG00000007733 | 0.741996 | 0.203554 | 0.004498 | 0.093822 | TNRC6C |  |
| ENSTGUG00000008423 | 0.742659 | 0.182894 | 0.002284 | 0.075337 | RNF7 |  |
| ENSTGUG00000000138 | 0.74304 | 0.114653 | 7.07E-05 | 0.019939 | ENSTGUG00000000138 | |
| ENSTGUG00000010222 | 0.755172 | 0.17626 | 0.0016 | 0.065599 | ALKBH3 |  |
| ENSTGUG00000008642 | 0.756347 | 0.204196 | 0.004081 | 0.091371 | ENSTGUG00000008642 | |
| ENSTGUG00000011473 | 0.772502 | 0.186446 | 0.002001 | 0.071826 | DCAF5 |  |
| ENSTGUG00000000765 | 0.786309 | 0.217444 | 0.00472 | 0.096156 | SRSF4 |  |
| ENSTGUG00000011810 | 0.78823 | 0.192717 | 0.002179 | 0.074042 | DARS |  |
| ENSTGUG00000009164 | 0.789021 | 0.193561 | 0.002227 | 0.07501 | SLC22A18 | |
| ENSTGUG00000000589 | 0.790767 | 0.220077 | 0.004904 | 0.097955 | FLI1 |  |
| ENSTGUG00000011171 | 0.804101 | 0.208734 | 0.0032 | 0.084885 | ENSTGUG00000011171 | |
| ENSTGUG00000010033 | 0.814058 | 0.221846 | 0.004321 | 0.09309 | ENSTGUG00000010033 | |
| ENSTGUG00000007048 | 0.82724 | 0.11644 | 3.28E-05 | 0.01536 | NONO |  |
| ENSTGUG00000001548 | 0.83274 | 0.191439 | 0.001444 | 0.062934 | ENSTGUG00000001548 | |
| ENSTGUG00000010157 | 0.83313 | 0.216755 | 0.003246 | 0.084885 | TRIM8 |  |
| ENSTGUG00000012248 | 0.836221 | 0.209474 | 0.002551 | 0.07825 | ENSTGUG00000012248 | |
| ENSTGUG00000002869 | 0.848106 | 0.236564 | 0.004969 | 0.098641 | HNRNPA2B1 | |
| ENSTGUG00000010538 | 0.850941 | 0.162939 | 0.000388 | 0.040083 | ENSTGUG00000010538 | |
| ENSTGUG00000006867 | 0.851471 | 0.222918 | 0.003375 | 0.086522 | F8 |  |
| ENSTGUG00000012259 | 0.858146 | 0.231756 | 0.00409 | 0.091371 | RAD51AP1 | |
| ENSTGUG00000004221 | 0.870549 | 0.22749 | 0.003336 | 0.086059 | MIS12 |  |
| ENSTGUG00000005825 | 0.870866 | 0.170398 | 0.000457 | 0.042738 | ENSTGUG00000005825 | |
| ENSTGUG00000007914 | 0.872119 | 0.20837 | 0.001871 | 0.070406 | RUVBL1 |  |
| ENSTGUG00000002357 | 0.873843 | 0.210657 | 0.001986 | 0.071808 | SMARCE1 | |
| ENSTGUG00000010128 | 0.87934 | 0.186631 | 0.000827 | 0.050993 | ENSTGUG00000010128 | |
| ENSTGUG00000003339 | 0.921122 | 0.231814 | 0.002628 | 0.078402 | ENSTGUG00000003339 | |
| ENSTGUG00000009857 | 0.923522 | 0.222863 | 0.001999 | 0.071826 | RASSF7 |  |
| ENSTGUG00000011360 | 0.924215 | 0.13683 | 5.01E-05 | 0.016112 | P2RY12 |  |
| ENSTGUG00000003640 | 0.961485 | 0.252746 | 0.003462 | 0.087547 | RAC3 |  |
| ENSTGUG00000010445 | 0.97856 | 0.128202 | 1.77E-05 | 0.012001 | ENSTGUG00000010445 | |
| ENSTGUG00000008899 | 0.982662 | 0.211596 | 0.000916 | 0.052138 | TXNDC12 | |
| ENSTGUG00000003387 | 0.987731 | 0.210655 | 0.000856 | 0.050993 | KPNB1 |  |
| ENSTGUG00000012289 | 0.995681 | 0.272661 | 0.00445 | 0.093822 | PNISR |  |
| ENSTGUG00000009845 | 0.996583 | 0.272345 | 0.004394 | 0.093787 | ENSTGUG00000009845 | |
| ENSTGUG00000007632 | 1.029641 | 0.254526 | 0.002341 | 0.076154 | CCT5 |  |
| ENSTGUG00000011903 | 1.037744 | 0.111817 | 3.14E-06 | 0.006772 | ALG5 |  |
| ENSTGUG00000000866 | 1.053783 | 0.24469 | 0.001545 | 0.064364 | ENSTGUG00000000866 | |
| ENSTGUG00000003940 | 1.099154 | 0.275254 | 0.002546 | 0.07825 | TFAP4 |  |
| ENSTGUG00000010890 | 1.104561 | 0.134064 | 9.09E-06 | 0.010222 | ENSTGUG00000010890 | |
| ENSTGUG00000007007 | 1.117633 | 0.284977 | 0.002858 | 0.08204 | ZDHHC5 |  |
| ENSTGUG00000008442 | 1.138266 | 0.29259 | 0.003008 | 0.082676 | SUN1 |  |
| ENSTGUG00000003381 | 1.155735 | 0.310967 | 0.003998 | 0.09046 | ENSTGUG00000003381 | |
| ENSTGUG00000010595 | 1.162089 | 0.302868 | 0.003281 | 0.085225 | GREM2 |  |
| ENSTGUG00000003289 | 1.173063 | 0.240843 | 0.000651 | 0.048382 | ENSTGUG00000003289 | |
| ENSTGUG00000009204 | 1.175501 | 0.31988 | 0.004283 | 0.092804 | ENSTGUG00000009204 | |
| ENSTGUG00000005704 | 1.181105 | 0.250729 | 0.000828 | 0.050993 | IHH |  |
| ENSTGUG00000002296 | 1.191761 | 0.293286 | 0.002274 | 0.07521 | LGALSL |  |
| ENSTGUG00000001908 | 1.235739 | 0.331415 | 0.003919 | 0.090127 | MAPT |  |
| ENSTGUG00000009062 | 1.249646 | 0.336108 | 0.003988 | 0.09046 | ENSTGUG00000009062 | |
| ENSTGUG00000004356 | 1.254318 | 0.333878 | 0.003742 | 0.088944 | DOLPP1 |  |
| ENSTGUG00000011942 | 1.276089 | 0.320116 | 0.002574 | 0.07825 | ENSTGUG00000011942 | |
| ENSTGUG00000003455 | 1.278489 | 0.350816 | 0.004504 | 0.093822 | SRSF6 |  |
| ENSTGUG00000010535 | 1.315701 | 0.3568 | 0.004194 | 0.09203 | HSPE1 |  |
| ENSTGUG00000012417 | 1.32462 | 0.310361 | 0.001642 | 0.065599 | ZC3H14 |  |
| ENSTGUG00000010776 | 1.341362 | 0.360821 | 0.003991 | 0.09046 | IMMT |  |
| ENSTGUG00000006760 | 1.37028 | 0.25975 | 0.00036 | 0.039041 | ENSTGUG00000006760 | |
| ENSTGUG00000000508 | 1.371862 | 0.300111 | 0.001024 | 0.054086 | HABP4 |  |
| ENSTGUG00000003534 | 1.37997 | 0.30144 | 0.001014 | 0.053782 | ENSTGUG00000003534 | |
| ENSTGUG00000011542 | 1.398414 | 0.373758 | 0.003837 | 0.089527 | EIF2S1 |  |
| ENSTGUG00000005212 | 1.399373 | 0.388183 | 0.004808 | 0.097077 | AAMP |  |
| ENSTGUG00000010223 | 1.405894 | 0.382067 | 0.004248 | 0.092575 | USP14 |  |
| ENSTGUG00000006281 | 1.424664 | 0.238337 | 0.000136 | 0.024586 | HAND2 |  |
| ENSTGUG00000007298 | 1.437348 | 0.382256 | 0.003721 | 0.088944 | GALK2 |  |
| ENSTGUG00000004069 | 1.444659 | 0.397549 | 0.004583 | 0.094607 | PPARG |  |
| ENSTGUG00000005633 | 1.447614 | 0.387619 | 0.003881 | 0.089666 | LMO2 |  |
| ENSTGUG00000005982 | 1.450545 | 0.367732 | 0.002754 | 0.080677 | CCNB2 |  |
| ENSTGUG00000006719 | 1.478655 | 0.251521 | 0.000155 | 0.026206 | ENSTGUG00000006719 | |
| ENSTGUG00000005568 | 1.481222 | 0.3794 | 0.002941 | 0.082321 | SLC2A6 |  |
| ENSTGUG00000012670 | 1.489048 | 0.382568 | 0.002999 | 0.082652 | EEF1A1 |  |
| ENSTGUG00000003199 | 1.494699 | 0.320584 | 0.000891 | 0.051706 | IDNK |  |
| ENSTGUG00000002350 | 1.557312 | 0.303933 | 0.000448 | 0.042738 | ENSTGUG00000002350 | |
| ENSTGUG00000005510 | 1.575912 | 0.393443 | 0.002496 | 0.07825 | ARL6IP4 |  |
| ENSTGUG00000007160 | 1.582322 | 0.415853 | 0.003457 | 0.087547 | TSPAN8 |  |
| ENSTGUG00000010942 | 1.614897 | 0.432452 | 0.003883 | 0.089666 | ENSTGUG00000010942 | |
| ENSTGUG00000000952 | 1.677001 | 0.465331 | 0.004817 | 0.097077 | ENSTGUG00000000952 | |
| ENSTGUG00000012270 | 1.700012 | 0.467853 | 0.004585 | 0.094607 | TIGAR |  |
| ENSTGUG00000003636 | 1.704437 | 0.461059 | 0.00413 | 0.091617 | GPATCH4 | |
| ENSTGUG00000003658 | 1.704729 | 0.450536 | 0.00358 | 0.088478 | SLC27A4 |  |
| ENSTGUG00000010661 | 1.724516 | 0.294057 | 0.000158 | 0.026206 | TMPRSS6 | |
| ENSTGUG00000005032 | 1.737499 | 0.473202 | 0.004304 | 0.093071 | ENSTGUG00000005032 | |
| ENSTGUG00000007896 | 1.750101 | 0.459058 | 0.003416 | 0.086912 | APPBP2 |  |
| ENSTGUG00000008113 | 1.768028 | 0.459968 | 0.003245 | 0.084885 | ENSTGUG00000008113 | |
| ENSTGUG00000009388 | 1.79448 | 0.45543 | 0.002774 | 0.080692 | LSP1 |  |
| ENSTGUG00000004379 | 1.807391 | 0.496361 | 0.004527 | 0.094046 | SULT1E1 | |
| ENSTGUG00000010113 | 1.816679 | 0.451712 | 0.002432 | 0.078012 | FES |  |
| ENSTGUG00000000192 | 1.836889 | 0.508556 | 0.004753 | 0.09665 | LEAP2 |  |
| ENSTGUG00000009697 | 1.846153 | 0.418997 | 0.001322 | 0.06056 | PSMD1 |  |
| ENSTGUG00000009270 | 1.881981 | 0.396862 | 0.00079 | 0.050738 | FHIT |  |
| ENSTGUG00000009601 | 1.896922 | 0.520023 | 0.004479 | 0.093822 | ENSTGUG00000009601 | |
| ENSTGUG00000012178 | 2.022629 | 0.504646 | 0.002486 | 0.07825 | DPYS |  |
| ENSTGUG00000003086 | 2.041698 | 0.569415 | 0.004965 | 0.098641 | ENSTGUG00000003086 | |
| ENSTGUG00000001701 | 2.043299 | 0.460879 | 0.001267 | 0.05988 | ENSTGUG00000001701 | |
| ENSTGUG00000003010 | 2.259862 | 0.49186 | 0.000988 | 0.053473 | ENSTGUG00000003010 | |
| ENSTGUG00000006069 | 2.351518 | 0.591722 | 0.002626 | 0.078402 | ALAS1 |  |
| ENSTGUG00000010926 | 2.387869 | 0.574607 | 0.001962 | 0.071808 | ENSTGUG00000010926 | |
| ENSTGUG00000013009 | 2.460846 | 0.658291 | 0.003858 | 0.089615 | L3HYPDH | |
| ENSTGUG00000011439 | 2.492863 | 0.190383 | 1.28E-07 | 0.00072 | CCDC177 | |
| ENSTGUG00000006763 | 2.517635 | 0.657027 | 0.003309 | 0.085739 | FAHD1 |  |
| ENSTGUG00000001670 | 2.799909 | 0.762761 | 0.004312 | 0.093071 | FKBP5 |  |
| ENSTGUG00000008392 | 3.291826 | 0.823739 | 0.002534 | 0.07825 | COX19 |  |
| ENSTGUG00000018079 | 6.135801 | 1.263192 | 0.000664 | 0.048382 | SNORD16 | |

Migratory state down regulated genes

| **Ensembl Gene ID** | beta.condition | SE.condition | pval.condition | FDR_pvalue | gene_short_name |
| --- | --- | --- | --- | --- | --- |
| ENSTGUG00000010708 | -7.96606 | 0.516153 | 2.66E-08 | 0.000299 | ENSTGUG00000010708 |
| ENSTGUG00000012313 | -4.88859 | 0.646958 | 1.93E-05 | 0.012083 | ENSTGUG00000012313 |
| ENSTGUG00000018714 | -4.84087 | 0.983622 | 0.000604 | 0.046941 | SCARNA15 |
| ENSTGUG00000009671 | -4.11972 | 1.142529 | 0.004802 | 0.097077 | ENSTGUG00000009671 |
| ENSTGUG00000017796 | -4.09219 | 1.048115 | 0.00294 | 0.082321 | SNORA81 |
| ENSTGUG00000017718 | -3.97108 | 0.967807 | 0.002134 | 0.074042 | SNORA63 |
| ENSTGUG00000002325 | -3.41023 | 0.702138 | 0.000664 | 0.048382 | ENSTGUG00000002325 |
| ENSTGUG00000005701 | -2.87556 | 0.60219 | 0.000751 | 0.050283 | ENSTGUG00000005701 |
| ENSTGUG00000011247 | -2.84374 | 0.54194 | 0.000375 | 0.039767 | CYP7B1 |
| ENSTGUG00000010886 | -2.74439 | 0.744355 | 0.004198 | 0.09203 | IFNGR1 |
| ENSTGUG00000012198 | -2.73117 | 0.640865 | 0.001659 | 0.065678 | ENSTGUG00000012198 |
| ENSTGUG00000011640 | -2.69204 | 0.721305 | 0.003896 | 0.089792 | ENPP3 |
| ENSTGUG00000011250 | -2.49541 | 0.565137 | 0.001303 | 0.060304 | ENSTGUG00000011250 |
| ENSTGUG00000002982 | -2.46601 | 0.63764 | 0.003122 | 0.084613 | PIK3R1 |
| ENSTGUG00000007252 | -2.46267 | 0.491639 | 0.00053 | 0.045145 | SECISBP2L |
| ENSTGUG00000004749 | -2.43752 | 0.382844 | 8.17E-05 | 0.020702 | REST |
| ENSTGUG00000007308 | -2.4168 | 0.491262 | 0.000605 | 0.046941 | ACOT9 |
| ENSTGUG00000010057 | -2.39542 | 0.397893 | 0.000129 | 0.024586 | LONRF2 |
| ENSTGUG00000012820 | -2.37105 | 0.488787 | 0.00067 | 0.048382 | RCOR1 |
| ENSTGUG00000004970 | -2.32527 | 0.635629 | 0.004402 | 0.093787 | FAM160A1 |
| ENSTGUG00000006257 | -2.32172 | 0.341551 | 4.75E-05 | 0.016112 | KDM1B |
| ENSTGUG00000011867 | -2.30184 | 0.23618 | 2.01E-06 | 0.005654 | ENSTGUG00000011867 |
| ENSTGUG00000013085 | -2.27995 | 0.55259 | 0.002057 | 0.072992 | LPIN1 |
| ENSTGUG00000009986 | -2.26454 | 0.631216 | 0.004949 | 0.098641 | GALNT2 |
| ENSTGUG00000001666 | -2.24312 | 0.379332 | 0.000148 | 0.025669 | C1GALT1 |
| ENSTGUG00000004788 | -2.21395 | 0.488297 | 0.001085 | 0.056196 | ENSTGUG00000004788 |
| ENSTGUG00000013255 | -2.19681 | 0.504193 | 0.001428 | 0.062898 | ENSTGUG00000013255 |
| ENSTGUG00000012521 | -2.18452 | 0.485868 | 0.00115 | 0.057525 | C14orf159 |
| ENSTGUG00000008151 | -2.15839 | 0.561245 | 0.003235 | 0.084885 | MOCOS |
| ENSTGUG00000000003 | -2.11392 | 0.49542 | 0.001645 | 0.065599 | EGR1 |
| ENSTGUG00000003931 | -2.07985 | 0.503961 | 0.002054 | 0.072992 | ENSTGUG00000003931 |
| ENSTGUG00000002660 | -2.06853 | 0.430166 | 0.000714 | 0.048979 | ENSTGUG00000002660 |
| ENSTGUG00000003429 | -2.04808 | 0.511967 | 0.002517 | 0.07825 | ENSTGUG00000003429 |
| ENSTGUG00000006726 | -2.03857 | 0.538699 | 0.003577 | 0.088478 | TGFBR1 |
| ENSTGUG00000010100 | -2.03531 | 0.246819 | 9.02E-06 | 0.010222 | KIAA0232 |
| ENSTGUG00000009220 | -2.02523 | 0.388847 | 0.000397 | 0.040539 | DENND4A |
| ENSTGUG00000012866 | -2.01762 | 0.359734 | 0.000225 | 0.03012 | BAG5 |
| ENSTGUG00000012310 | -1.98128 | 0.538414 | 0.004247 | 0.092575 | ENSTGUG00000012310 |
| ENSTGUG00000009438 | -1.97145 | 0.486352 | 0.00231 | 0.075971 | CCDC149 |
| ENSTGUG00000005188 | -1.95566 | 0.395999 | 0.000588 | 0.046941 | REEP3 |
| ENSTGUG00000006121 | -1.95455 | 0.392728 | 0.000556 | 0.045736 | ENSTGUG00000006121 |
| ENSTGUG00000011531 | -1.91001 | 0.370352 | 0.000427 | 0.041843 | ENSTGUG00000011531 |
| ENSTGUG00000009545 | -1.90474 | 0.356816 | 0.000329 | 0.037091 | ENSTGUG00000009545 |
| ENSTGUG00000005703 | -1.90142 | 0.281464 | 5.01E-05 | 0.016112 | LDB3 |
| ENSTGUG00000013489 | -1.8931 | 0.242151 | 1.44E-05 | 0.011566 | ZNF654 |
| ENSTGUG00000001510 | -1.87866 | 0.273065 | 4.30E-05 | 0.016112 | PANK3 |
| ENSTGUG00000001806 | -1.83156 | 0.419833 | 0.001415 | 0.062898 | EGFR |
| ENSTGUG00000008271 | -1.82524 | 0.389464 | 0.000859 | 0.050993 | ENSTGUG00000008271 |
| ENSTGUG00000012527 | -1.82457 | 0.493193 | 0.004112 | 0.091386 | KLF12 |
| ENSTGUG00000010032 | -1.80672 | 0.432762 | 0.001903 | 0.070406 | RNPEPL1 |
| ENSTGUG00000004646 | -1.79998 | 0.383247 | 0.000846 | 0.050993 | CPED1 |
| ENSTGUG00000005602 | -1.79957 | 0.25785 | 3.81E-05 | 0.015889 | ENSTGUG00000005602 |
| ENSTGUG00000002596 | -1.79845 | 0.439541 | 0.002173 | 0.074042 | SNX13 |
| ENSTGUG00000009700 | -1.79715 | 0.432622 | 0.001967 | 0.071808 | ENSTGUG00000009700 |
| ENSTGUG00000011453 | -1.79402 | 0.31956 | 0.000223 | 0.03012 | ENSTGUG00000011453 |
| ENSTGUG00000003159 | -1.77765 | 0.377224 | 0.000826 | 0.050993 | TBC1D5 |
| ENSTGUG00000007651 | -1.77481 | 0.452586 | 0.002859 | 0.08204 | SLC25A36 |
| ENSTGUG00000012130 | -1.76719 | 0.404032 | 0.001391 | 0.062383 | ENSTGUG00000012130 |
| ENSTGUG00000002044 | -1.76408 | 0.362959 | 0.000661 | 0.048382 | ENSTGUG00000002044 |
| ENSTGUG00000011840 | -1.75926 | 0.424057 | 0.001984 | 0.071808 | SPOPL |
| ENSTGUG00000002585 | -1.75023 | 0.381402 | 0.000997 | 0.05364 | AHR |
| ENSTGUG00000007006 | -1.74302 | 0.318457 | 0.000272 | 0.032746 | NFATC3 |
| ENSTGUG00000002500 | -1.70701 | 0.285194 | 0.000135 | 0.024586 | ENSTGUG00000002500 |
| ENSTGUG00000008575 | -1.69558 | 0.309377 | 0.000269 | 0.032746 | PIK3C2A |
| ENSTGUG00000002430 | -1.69416 | 0.36491 | 0.000918 | 0.052138 | ENSTGUG00000002430 |
| ENSTGUG00000012536 | -1.69336 | 0.412196 | 0.002117 | 0.073932 | EFR3A |
| ENSTGUG00000006380 | -1.68999 | 0.334181 | 0.000494 | 0.044098 | ZCCHC24 |
| ENSTGUG00000005589 | -1.68851 | 0.447921 | 0.003664 | 0.088944 | ENSTGUG00000005589 |
| ENSTGUG00000004281 | -1.67836 | 0.356291 | 0.000828 | 0.050993 | HACD2 |
| ENSTGUG00000009786 | -1.67549 | 0.328236 | 0.000461 | 0.042738 | CMTM4 |
| ENSTGUG00000011889 | -1.6585 | 0.237758 | 3.83E-05 | 0.015889 | ENSTGUG00000011889 |
| ENSTGUG00000003508 | -1.6535 | 0.351493 | 0.000836 | 0.050993 | ENSTGUG00000003508 |
| ENSTGUG00000005555 | -1.65098 | 0.447548 | 0.004184 | 0.09203 | MTMR10 |
| ENSTGUG00000011465 | -1.65042 | 0.423839 | 0.00299 | 0.082622 | MTMR6 |
| ENSTGUG00000007495 | -1.64875 | 0.334879 | 0.000602 | 0.046941 | ENSTGUG00000007495 |
| ENSTGUG00000003986 | -1.63849 | 0.380053 | 0.001534 | 0.064249 | ANKFY1 |
| ENSTGUG00000005619 | -1.63227 | 0.416243 | 0.00286 | 0.08204 | ENSTGUG00000005619 |
| ENSTGUG00000013350 | -1.62491 | 0.353696 | 0.000989 | 0.053473 | MAN1A2 |
| ENSTGUG00000005414 | -1.6192 | 0.447562 | 0.004707 | 0.096156 | C7orf60 |
| ENSTGUG00000003367 | -1.61156 | 0.366599 | 0.001343 | 0.060922 | GLTSCR1L |
| ENSTGUG00000010569 | -1.60808 | 0.223192 | 2.91E-05 | 0.014225 | LETM1 |
| ENSTGUG00000007282 | -1.60791 | 0.340916 | 0.000821 | 0.050993 | FOXO4 |
| ENSTGUG00000000416 | -1.60588 | 0.317324 | 0.000491 | 0.044098 | ENSTGUG00000000416 |
| ENSTGUG00000010949 | -1.59516 | 0.424367 | 0.003729 | 0.088944 | ENSTGUG00000010949 |
| ENSTGUG00000010105 | -1.59464 | 0.407306 | 0.002889 | 0.082158 | TBC1D14 |
| ENSTGUG00000000154 | -1.59134 | 0.254712 | 9.53E-05 | 0.022624 | GAK |
| ENSTGUG00000012751 | -1.59102 | 0.288335 | 0.000255 | 0.0319 | ENSTGUG00000012751 |
| ENSTGUG00000005043 | -1.57432 | 0.148347 | 9.19E-07 | 0.003447 | JAK2 |
| ENSTGUG00000002601 | -1.57113 | 0.363895 | 0.001519 | 0.064249 | MIER3 |
| ENSTGUG00000000329 | -1.56986 | 0.203024 | 1.58E-05 | 0.011878 | CPEB4 |
| ENSTGUG00000005136 | -1.56831 | 0.36922 | 0.001696 | 0.066653 | MET |
| ENSTGUG00000006135 | -1.5582 | 0.404878 | 0.00322 | 0.084885 | COL4A3BP |
| ENSTGUG00000013359 | -1.54877 | 0.362868 | 0.001642 | 0.065599 | ENSTGUG00000013359 |
| ENSTGUG00000010765 | -1.54668 | 0.30829 | 0.000524 | 0.045145 | ENSTGUG00000010765 |
| ENSTGUG00000008382 | -1.53146 | 0.322536 | 0.000782 | 0.050629 | ENSTGUG00000008382 |
| ENSTGUG00000003235 | -1.52877 | 0.167322 | 3.61E-06 | 0.006772 | TBC1D2B |
| ENSTGUG00000002058 | -1.52823 | 0.382098 | 0.00252 | 0.07825 | ENSTGUG00000002058 |
| ENSTGUG00000012754 | -1.52386 | 0.370758 | 0.00211 | 0.073932 | ENSTGUG00000012754 |
| ENSTGUG00000006758 | -1.51711 | 0.337466 | 0.001151 | 0.057525 | ENSTGUG00000006758 |
| ENSTGUG00000007871 | -1.51695 | 0.302514 | 0.000526 | 0.045145 | USP46 |
| ENSTGUG00000001553 | -1.51162 | 0.318412 | 0.000783 | 0.050629 | HIPK1 |
| ENSTGUG00000002064 | -1.50853 | 0.366372 | 0.002085 | 0.073751 | ENSTGUG00000002064 |
| ENSTGUG00000010859 | -1.50506 | 0.385686 | 0.00295 | 0.082321 | TPP2 |
| ENSTGUG00000005926 | -1.50043 | 0.38894 | 0.003172 | 0.084885 | ENSTGUG00000005926 |
| ENSTGUG00000001285 | -1.4967 | 0.358438 | 0.001901 | 0.070406 | STAM |
| ENSTGUG00000007517 | -1.48353 | 0.349779 | 0.001713 | 0.066653 | CYLD |
| ENSTGUG00000001730 | -1.48335 | 0.304813 | 0.000655 | 0.048382 | ENSTGUG00000001730 |
| ENSTGUG00000007845 | -1.48101 | 0.372368 | 0.002612 | 0.078344 | ACSL3 |
| ENSTGUG00000008657 | -1.47662 | 0.216199 | 4.57E-05 | 0.016112 | ENSTGUG00000008657 |
| ENSTGUG00000010267 | -1.47004 | 0.34947 | 0.00181 | 0.069471 | TRAK2 |
| ENSTGUG00000011533 | -1.46659 | 0.268197 | 0.000274 | 0.032746 | ENSTGUG00000011533 |
| ENSTGUG00000002812 | -1.46175 | 0.385729 | 0.003546 | 0.088442 | SLC30A10 |
| ENSTGUG00000007299 | -1.45735 | 0.324546 | 0.00116 | 0.057731 | TOM1L2 |
| ENSTGUG00000011165 | -1.4546 | 0.284623 | 0.000457 | 0.042738 | UBR1 |
| ENSTGUG00000013398 | -1.44909 | 0.235861 | 0.000109 | 0.023221 | ENSTGUG00000013398 |
| ENSTGUG00000008439 | -1.44736 | 0.279908 | 0.000419 | 0.041843 | WWC3 |
| ENSTGUG00000008376 | -1.44135 | 0.363534 | 0.002665 | 0.078885 | ZBTB38 |
| ENSTGUG00000008171 | -1.43273 | 0.381524 | 0.003751 | 0.088944 | NFXL1 |
| ENSTGUG00000003151 | -1.43173 | 0.229522 | 9.66E-05 | 0.022624 | RALGAPB |
| ENSTGUG00000006820 | -1.42845 | 0.353019 | 0.002337 | 0.076154 | DCP1A |
| ENSTGUG00000005331 | -1.4282 | 0.371313 | 0.003231 | 0.084885 | KIAA0430 |
| ENSTGUG00000007738 | -1.4271 | 0.256136 | 0.000237 | 0.031005 | ENSTGUG00000007738 |
| ENSTGUG00000010101 | -1.42599 | 0.191682 | 2.21E-05 | 0.013093 | FAM117B |
| ENSTGUG00000003013 | -1.4253 | 0.371326 | 0.003274 | 0.085219 | FLVCR1 |
| ENSTGUG00000000637 | -1.42329 | 0.246194 | 0.000177 | 0.026259 | CYFIP2 |
| ENSTGUG00000008437 | -1.41778 | 0.229156 | 0.000103 | 0.023211 | ENSTGUG00000008437 |
| ENSTGUG00000009020 | -1.40347 | 0.225761 | 9.93E-05 | 0.022785 | ZNF507 |
| ENSTGUG00000009607 | -1.40201 | 0.244664 | 0.00019 | 0.027425 | PIKFYVE |
| ENSTGUG00000010290 | -1.40128 | 0.234768 | 0.000138 | 0.024586 | LYST |
| ENSTGUG00000004690 | -1.39349 | 0.313567 | 0.001247 | 0.05988 | AFF2 |
| ENSTGUG00000012623 | -1.39065 | 0.218781 | 8.28E-05 | 0.020702 | BTBD7 |
| ENSTGUG00000007445 | -1.38286 | 0.306637 | 0.001126 | 0.057299 | OSBPL8 |
| ENSTGUG00000008403 | -1.38249 | 0.330712 | 0.001887 | 0.070406 | CNST |
| ENSTGUG00000006543 | -1.37156 | 0.352671 | 0.003014 | 0.082676 | WWC2 |
| ENSTGUG00000011459 | -1.36529 | 0.222159 | 0.000109 | 0.023221 | NCOA2 |
| ENSTGUG00000008720 | -1.36128 | 0.173607 | 1.40E-05 | 0.011566 | LMTK2 |
| ENSTGUG00000011062 | -1.35874 | 0.308454 | 0.001325 | 0.06056 | TMEM181 |
| ENSTGUG00000005229 | -1.35279 | 0.212673 | 8.24E-05 | 0.020702 | ENSTGUG00000005229 |
| ENSTGUG00000003723 | -1.34842 | 0.302705 | 0.001226 | 0.05945 | JMY |
| ENSTGUG00000011900 | -1.33538 | 0.367801 | 0.004607 | 0.094878 | ARHGAP5 |
| ENSTGUG00000008988 | -1.33478 | 0.326259 | 0.002175 | 0.074042 | CPEB3 |
| ENSTGUG00000012639 | -1.33468 | 0.313369 | 0.001665 | 0.065712 | ENSTGUG00000012639 |
| ENSTGUG00000013488 | -1.33289 | 0.260869 | 0.000458 | 0.042738 | ENSTGUG00000013488 |
| ENSTGUG00000010893 | -1.32766 | 0.300326 | 0.001293 | 0.06007 | CCDC186 |
| ENSTGUG00000000708 | -1.32287 | 0.368171 | 0.004904 | 0.097955 | UBE3C |
| ENSTGUG00000008543 | -1.32272 | 0.264313 | 0.000534 | 0.045145 | PRKD3 |
| ENSTGUG00000002523 | -1.32143 | 0.356767 | 0.004082 | 0.091371 | SUPT7L |
| ENSTGUG00000007855 | -1.32064 | 0.203869 | 7.09E-05 | 0.019939 | TMTC2 |
| ENSTGUG00000001482 | -1.32047 | 0.191547 | 4.23E-05 | 0.016112 | TSTD2 |
| ENSTGUG00000000001 | -1.31526 | 0.265703 | 0.000578 | 0.046941 | C2CD2L |
| ENSTGUG00000013626 | -1.3152 | 0.261326 | 0.000512 | 0.044814 | ENSTGUG00000013626 |
| ENSTGUG00000000754 | -1.31253 | 0.362481 | 0.004682 | 0.095913 | ENSTGUG00000000754 |
| ENSTGUG00000007194 | -1.31132 | 0.229485 | 0.000194 | 0.027684 | APPL1 |
| ENSTGUG00000012441 | -1.30991 | 0.205993 | 8.26E-05 | 0.020702 | FOXN3 |
| ENSTGUG00000004589 | -1.30123 | 0.212341 | 0.000112 | 0.023221 | CDYL2 |
| ENSTGUG00000000606 | -1.29805 | 0.33889 | 0.003317 | 0.085758 | ENSTGUG00000000606 |
| ENSTGUG00000009051 | -1.29391 | 0.245675 | 0.000365 | 0.039041 | MBP |
| ENSTGUG00000006395 | -1.29257 | 0.247237 | 0.000385 | 0.040083 | ZMIZ1 |
| ENSTGUG00000012030 | -1.28961 | 0.346346 | 0.003953 | 0.09046 | VPS13B |
| ENSTGUG00000009594 | -1.28847 | 0.279088 | 0.000955 | 0.053038 | WTIP |
| ENSTGUG00000001310 | -1.28774 | 0.209713 | 0.00011 | 0.023221 | ENSTGUG00000001310 |
| ENSTGUG00000005419 | -1.28462 | 0.265634 | 0.000686 | 0.048382 | TMEM168 |
| ENSTGUG00000011318 | -1.28286 | 0.350576 | 0.004394 | 0.093787 | PLEKHG1 |
| ENSTGUG00000000426 | -1.28108 | 0.237948 | 0.000308 | 0.036127 | INSR |
| ENSTGUG00000003069 | -1.28015 | 0.22064 | 0.000172 | 0.026259 | ENSTGUG00000003069 |
| ENSTGUG00000007655 | -1.27972 | 0.312257 | 0.00215 | 0.074042 | SLC7A5 |
| ENSTGUG00000005470 | -1.27817 | 0.340897 | 0.003787 | 0.089035 | KLF13 |
| ENSTGUG00000012387 | -1.278 | 0.32125 | 0.002608 | 0.078344 | SEL1L |
| ENSTGUG00000010277 | -1.27397 | 0.266241 | 0.00074 | 0.050141 | ENSTGUG00000010277 |
| ENSTGUG00000005714 | -1.27215 | 0.265747 | 0.000738 | 0.050141 | ENSTGUG00000005714 |
| ENSTGUG00000005717 | -1.27047 | 0.271622 | 0.000871 | 0.051286 | ZNF366 |
| ENSTGUG00000012650 | -1.26076 | 0.260847 | 0.000688 | 0.048382 | CUL5 |
| ENSTGUG00000005042 | -1.24859 | 0.330751 | 0.003632 | 0.088944 | LIMD1 |
| ENSTGUG00000013379 | -1.24446 | 0.334304 | 0.003959 | 0.09046 | GPR156 |
| ENSTGUG00000005362 | -1.24262 | 0.310265 | 0.002498 | 0.07825 | ASPN |
| ENSTGUG00000006836 | -1.23959 | 0.188861 | 6.36E-05 | 0.018832 | MDM2 |
| ENSTGUG00000003083 | -1.23914 | 0.268482 | 0.000957 | 0.053038 | PHF20 |
| ENSTGUG00000004954 | -1.23722 | 0.326844 | 0.00357 | 0.088478 | QSER1 |
| ENSTGUG00000005922 | -1.23403 | 0.192111 | 7.60E-05 | 0.020702 | USP31 |
| ENSTGUG00000012295 | -1.23194 | 0.27489 | 0.001176 | 0.057761 | FBXL4 |
| ENSTGUG00000002338 | -1.23184 | 0.30554 | 0.002393 | 0.077113 | SUV39H2 |
| ENSTGUG00000011816 | -1.23039 | 0.308421 | 0.002562 | 0.07825 | G2E3 |
| ENSTGUG00000002906 | -1.22927 | 0.284512 | 0.001512 | 0.064249 | HOXA5 |
| ENSTGUG00000002472 | -1.22768 | 0.309481 | 0.002657 | 0.078885 | ENSTGUG00000002472 |
| ENSTGUG00000012378 | -1.21112 | 0.297253 | 0.002234 | 0.07501 | ENSTGUG00000012378 |
| ENSTGUG00000002329 | -1.21106 | 0.274641 | 0.001315 | 0.06056 | DIAPH2 |
| ENSTGUG00000004023 | -1.20254 | 0.217 | 0.000247 | 0.031561 | ENSTGUG00000004023 |
| ENSTGUG00000004554 | -1.2011 | 0.318859 | 0.00368 | 0.088944 | TTC39B |
| ENSTGUG00000010736 | -1.20056 | 0.232657 | 0.000425 | 0.041843 | PIK3CA |
| ENSTGUG00000010407 | -1.19965 | 0.245297 | 0.000632 | 0.048342 | KDELR3 |
| ENSTGUG00000001315 | -1.19965 | 0.304256 | 0.002762 | 0.080677 | FNIP1 |
| ENSTGUG00000010927 | -1.19697 | 0.197909 | 0.000124 | 0.024586 | ABLIM1 |
| ENSTGUG00000007051 | -1.19566 | 0.274647 | 0.001436 | 0.062898 | PTPRB |
| ENSTGUG00000003908 | -1.19481 | 0.287597 | 0.001966 | 0.071808 | PRKCE |
| ENSTGUG00000009752 | -1.19409 | 0.328352 | 0.004562 | 0.094488 | ENSTGUG00000009752 |
| ENSTGUG00000004760 | -1.19218 | 0.312358 | 0.003392 | 0.08669 | DOPEY2 |
| ENSTGUG00000008614 | -1.18954 | 0.301133 | 0.002729 | 0.080348 | SLC39A6 |
| ENSTGUG00000002309 | -1.18496 | 0.248642 | 0.000762 | 0.050406 | RREB1 |
| ENSTGUG00000007476 | -1.1757 | 0.255477 | 0.000977 | 0.053473 | CHD9 |
| ENSTGUG00000007200 | -1.17553 | 0.233657 | 0.000513 | 0.044814 | ENSTGUG00000007200 |
| ENSTGUG00000005477 | -1.17415 | 0.202321 | 0.000172 | 0.026259 | ENSTGUG00000005477 |
| ENSTGUG00000007055 | -1.17303 | 0.258364 | 0.001074 | 0.056195 | NFX1 |
| ENSTGUG00000012023 | -1.16505 | 0.302079 | 0.003177 | 0.084885 | STK3 |
| ENSTGUG00000011626 | -1.16435 | 0.307264 | 0.003547 | 0.088442 | CTGF |
| ENSTGUG00000000314 | -1.16358 | 0.291843 | 0.002572 | 0.07825 | CREBRF |
| ENSTGUG00000003349 | -1.16162 | 0.226453 | 0.000444 | 0.042738 | ENSTGUG00000003349 |
| ENSTGUG00000011305 | -1.1614 | 0.18577 | 9.48E-05 | 0.022624 | VCPIP1 |
| ENSTGUG00000010116 | -1.16 | 0.305782 | 0.003523 | 0.088235 | LDLRAD3 |
| ENSTGUG00000003433 | -1.15586 | 0.199363 | 0.000173 | 0.026259 | VPS13A |
| ENSTGUG00000011863 | -1.15421 | 0.31704 | 0.004533 | 0.094046 | MAN1A1 |
| ENSTGUG00000011149 | -1.14687 | 0.276438 | 0.001984 | 0.071808 | ENSTGUG00000011149 |
| ENSTGUG00000001640 | -1.14653 | 0.309775 | 0.004101 | 0.091371 | ENSTGUG00000001640 |
| ENSTGUG00000006057 | -1.14513 | 0.221983 | 0.000426 | 0.041843 | ENSTGUG00000006057 |
| ENSTGUG00000002468 | -1.13876 | 0.239359 | 0.000771 | 0.050438 | WDFY3 |
| ENSTGUG00000004512 | -1.13859 | 0.256257 | 0.001248 | 0.05988 | ENSTGUG00000004512 |
| ENSTGUG00000011368 | -1.13232 | 0.287623 | 0.002789 | 0.080757 | ENSTGUG00000011368 |
| ENSTGUG00000008675 | -1.13195 | 0.203191 | 0.000237 | 0.031005 | APBB2 |
| ENSTGUG00000009717 | -1.1307 | 0.214523 | 0.000362 | 0.039041 | ENSTGUG00000009717 |
| ENSTGUG00000004282 | -1.12969 | 0.268074 | 0.001788 | 0.068874 | ZBTB41 |
| ENSTGUG00000005064 | -1.12772 | 0.282099 | 0.002528 | 0.07825 | HIPK3 |
| ENSTGUG00000007934 | -1.12741 | 0.251986 | 0.00119 | 0.058178 | TESK2 |
| ENSTGUG00000012633 | -1.12663 | 0.239621 | 0.000839 | 0.050993 | KDM5A |
| ENSTGUG00000010139 | -1.12428 | 0.249902 | 0.001145 | 0.057525 | TRAF6 |
| ENSTGUG00000011408 | -1.11533 | 0.297629 | 0.0038 | 0.089035 | SGCG |
| ENSTGUG00000008211 | -1.11046 | 0.293389 | 0.003573 | 0.088478 | ENSTGUG00000008211 |
| ENSTGUG00000011468 | -1.10975 | 0.14578 | 1.81E-05 | 0.012001 | DENND5B |
| ENSTGUG00000011686 | -1.10933 | 0.232538 | 0.000756 | 0.050336 | KDM7A |
| ENSTGUG00000012619 | -1.10847 | 0.163609 | 4.89E-05 | 0.016112 | MYO6 |
| ENSTGUG00000006889 | -1.10842 | 0.222503 | 0.000552 | 0.045736 | ENSTGUG00000006889 |
| ENSTGUG00000000942 | -1.1074 | 0.262029 | 0.001754 | 0.068024 | PDGFRB |
| ENSTGUG00000002448 | -1.10003 | 0.227947 | 0.000696 | 0.048621 | ARL15 |
| ENSTGUG00000011462 | -1.09527 | 0.291523 | 0.00374 | 0.088944 | ENSTGUG00000011462 |
| ENSTGUG00000012907 | -1.09178 | 0.259701 | 0.001817 | 0.069511 | CEP170B |
| ENSTGUG00000003535 | -1.0853 | 0.268144 | 0.002333 | 0.076154 | ENSTGUG00000003535 |
| ENSTGUG00000011205 | -1.08151 | 0.244326 | 0.001281 | 0.06007 | ELMSAN1 |
| ENSTGUG00000010613 | -1.08009 | 0.163824 | 6.13E-05 | 0.018638 | PGAP1 |
| ENSTGUG00000012136 | -1.07523 | 0.21742 | 0.000583 | 0.046941 | LACE1 |
| ENSTGUG00000012747 | -1.0731 | 0.286297 | 0.003795 | 0.089035 | ENSTGUG00000012747 |
| ENSTGUG00000009770 | -1.07245 | 0.24956 | 0.001568 | 0.064826 | PARD3B |
| ENSTGUG00000002377 | -1.06994 | 0.238455 | 0.001166 | 0.057761 | ENSTGUG00000002377 |
| ENSTGUG00000001438 | -1.06971 | 0.275319 | 0.003032 | 0.082745 | LUZP1 |
| ENSTGUG00000006888 | -1.06834 | 0.283961 | 0.003708 | 0.088944 | TMOD2 |
| ENSTGUG00000002422 | -1.06801 | 0.281442 | 0.003516 | 0.088235 | KIF16B |
| ENSTGUG00000003832 | -1.06654 | 0.267392 | 0.002565 | 0.07825 | PREPL |
| ENSTGUG00000010953 | -1.06162 | 0.23942 | 0.001266 | 0.05988 | SKIL |
| ENSTGUG00000000462 | -1.04837 | 0.203341 | 0.000428 | 0.041843 | ENSTGUG00000000462 |
| ENSTGUG00000001104 | -1.04562 | 0.281249 | 0.00399 | 0.09046 | CEP120 |
| ENSTGUG00000008236 | -1.04514 | 0.252327 | 0.002005 | 0.071826 | ENSTGUG00000008236 |
| ENSTGUG00000008089 | -1.0443 | 0.222078 | 0.000839 | 0.050993 | MAST2 |
| ENSTGUG00000009007 | -1.04348 | 0.266326 | 0.002875 | 0.082158 | PTPRJ |
| ENSTGUG00000005111 | -1.03795 | 0.173891 | 0.000138 | 0.024586 | ENSTGUG00000005111 |
| ENSTGUG00000010919 | -1.03519 | 0.176201 | 0.000156 | 0.026206 | MGA |
| ENSTGUG00000002126 | -1.03253 | 0.243369 | 0.001709 | 0.066653 | RICTOR |
| ENSTGUG00000002680 | -1.0285 | 0.220785 | 0.000897 | 0.051706 | ENSTGUG00000002680 |
| ENSTGUG00000009775 | -1.02759 | 0.269053 | 0.003377 | 0.086522 | ENSTGUG00000009775 |
| ENSTGUG00000009840 | -1.0213 | 0.27193 | 0.003748 | 0.088944 | ENSTGUG00000009840 |
| ENSTGUG00000007083 | -1.02098 | 0.208625 | 0.000629 | 0.048342 | ENSTGUG00000007083 |
| ENSTGUG00000011007 | -1.01479 | 0.210461 | 0.0007 | 0.048621 | C20orf194 |
| ENSTGUG00000012514 | -1.0134 | 0.272059 | 0.003943 | 0.09046 | RPS6KA5 |
| ENSTGUG00000005026 | -1.01323 | 0.189863 | 0.00033 | 0.037091 | MKL2 |
| ENSTGUG00000012376 | -1.01306 | 0.235043 | 0.001537 | 0.064249 | ENSTGUG00000012376 |
| ENSTGUG00000011162 | -1.00994 | 0.174546 | 0.000176 | 0.026259 | HCFC2 |
| ENSTGUG00000011441 | -1.00957 | 0.241584 | 0.001891 | 0.070406 | TMTC1 |
| ENSTGUG00000010530 | -1.00837 | 0.227841 | 0.001283 | 0.06007 | MXD4 |
| ENSTGUG00000009663 | -1.00757 | 0.274894 | 0.004351 | 0.093554 | ENSTGUG00000009663 |
| ENSTGUG00000012458 | -1.00598 | 0.275694 | 0.00447 | 0.093822 | ENSTGUG00000012458 |
| ENSTGUG00000012522 | -1.00511 | 0.233174 | 0.001536 | 0.064249 | ASAP1 |
| ENSTGUG00000011715 | -1.00409 | 0.260377 | 0.003179 | 0.084885 | CCNT2 |
| ENSTGUG00000009822 | -1.00095 | 0.197316 | 0.000483 | 0.04394 | CPEB2 |
| ENSTGUG00000010399 | -0.99807 | 0.26491 | 0.003676 | 0.088944 | ENSTGUG00000010399 |
| ENSTGUG00000010480 | -0.99665 | 0.247979 | 0.002442 | 0.078022 | SATB2 |
| ENSTGUG00000008104 | -0.99471 | 0.188012 | 0.000352 | 0.038832 | NFAT5 |
| ENSTGUG00000005731 | -0.98978 | 0.235988 | 0.001846 | 0.070121 | NRXN1 |
| ENSTGUG00000012926 | -0.98743 | 0.263872 | 0.003834 | 0.089527 | BRF1 |
| ENSTGUG00000008483 | -0.98674 | 0.178482 | 0.000252 | 0.031785 | ATP8A1 |
| ENSTGUG00000000676 | -0.98447 | 0.247233 | 0.002593 | 0.078344 | MEF2C |
| ENSTGUG00000004043 | -0.98103 | 0.234916 | 0.001899 | 0.070406 | HEG1 |
| ENSTGUG00000013179 | -0.97972 | 0.226994 | 0.001523 | 0.064249 | ENSTGUG00000013179 |
| ENSTGUG00000010333 | -0.97372 | 0.249427 | 0.002943 | 0.082321 | FAM126B |
| ENSTGUG00000009761 | -0.9722 | 0.208389 | 0.000887 | 0.051706 | ARMC9 |
| ENSTGUG00000007178 | -0.96579 | 0.18097 | 0.00033 | 0.037091 | ELOVL1 |
| ENSTGUG00000013322 | -0.96357 | 0.256258 | 0.003721 | 0.088944 | KIF13B |
| ENSTGUG00000006226 | -0.96331 | 0.211788 | 0.001061 | 0.055745 | LRRC8C |
| ENSTGUG00000004323 | -0.9614 | 0.144356 | 5.64E-05 | 0.017616 | DENND1B |
| ENSTGUG00000007795 | -0.95382 | 0.223262 | 0.001631 | 0.065599 | BEND2 |
| ENSTGUG00000009239 | -0.95179 | 0.21835 | 0.001423 | 0.062898 | SPAG9 |
| ENSTGUG00000013442 | -0.94318 | 0.161312 | 0.000162 | 0.026259 | ENSTGUG00000013442 |
| ENSTGUG00000012816 | -0.94239 | 0.261189 | 0.004783 | 0.096955 | ENSTGUG00000012816 |
| ENSTGUG00000012375 | -0.9395 | 0.179831 | 0.000387 | 0.040083 | AKAP11 |
| ENSTGUG00000011928 | -0.93578 | 0.195729 | 0.000745 | 0.050145 | KPNA5 |
| ENSTGUG00000001808 | -0.93398 | 0.213573 | 0.001392 | 0.062383 | ENSTGUG00000001808 |
| ENSTGUG00000008498 | -0.92477 | 0.199859 | 0.00094 | 0.052609 | XRN1 |
| ENSTGUG00000008467 | -0.92136 | 0.239404 | 0.00322 | 0.084885 | SHROOM2 |
| ENSTGUG00000008078 | -0.91684 | 0.216106 | 0.00171 | 0.066653 | ENSTGUG00000008078 |
| ENSTGUG00000008164 | -0.91438 | 0.197083 | 0.000923 | 0.052138 | ENSTGUG00000008164 |
| ENSTGUG00000007341 | -0.91081 | 0.240901 | 0.003597 | 0.08871 | SLMAP |
| ENSTGUG00000005848 | -0.90897 | 0.212919 | 0.001639 | 0.065599 | PARG |
| ENSTGUG00000008522 | -0.90444 | 0.24439 | 0.004103 | 0.091371 | SOX6 |
| ENSTGUG00000003376 | -0.90302 | 0.128775 | 3.66E-05 | 0.015889 | ENSTGUG00000003376 |
| ENSTGUG00000003733 | -0.89683 | 0.193705 | 0.000936 | 0.052609 | ENSTGUG00000003733 |
| ENSTGUG00000005797 | -0.89577 | 0.2234 | 0.002479 | 0.07825 | ENSTGUG00000005797 |
| ENSTGUG00000008691 | -0.89156 | 0.243593 | 0.004389 | 0.093787 | STRN |
| ENSTGUG00000002696 | -0.88847 | 0.193283 | 0.000985 | 0.053473 | ENSTGUG00000002696 |
| ENSTGUG00000001581 | -0.88793 | 0.183668 | 0.000687 | 0.048382 | RSBN1 |
| ENSTGUG00000002464 | -0.88294 | 0.240055 | 0.00426 | 0.092575 | DHX29 |
| ENSTGUG00000006295 | -0.8827 | 0.188126 | 0.000852 | 0.050993 | ENSTGUG00000006295 |
| ENSTGUG00000008290 | -0.87611 | 0.230463 | 0.003477 | 0.087674 | PLXNC1 |
| ENSTGUG00000011226 | -0.8742 | 0.202766 | 0.001534 | 0.064249 | ASPH |
| ENSTGUG00000001279 | -0.87293 | 0.164831 | 0.00035 | 0.038832 | KIAA0368 |
| ENSTGUG00000010067 | -0.86798 | 0.176144 | 0.000598 | 0.046941 | PLEKHM3 |
| ENSTGUG00000005092 | -0.86698 | 0.223214 | 0.003039 | 0.082745 | SNX29 |
| ENSTGUG00000000830 | -0.86236 | 0.155055 | 0.00024 | 0.031044 | GDA |
| ENSTGUG00000000686 | -0.85641 | 0.23177 | 0.004142 | 0.091652 | EPS15L1 |
| ENSTGUG00000009145 | -0.85518 | 0.176814 | 0.000685 | 0.048382 | DPP8 |
| ENSTGUG00000002852 | -0.85414 | 0.191526 | 0.001217 | 0.059235 | GPATCH2 |
| ENSTGUG00000000065 | -0.85241 | 0.221138 | 0.003188 | 0.084885 | KMT2A |
| ENSTGUG00000002864 | -0.85101 | 0.23489 | 0.004666 | 0.095762 | SGTB |
| ENSTGUG00000008329 | -0.84802 | 0.187249 | 0.001093 | 0.056196 | PHLPP2 |
| ENSTGUG00000008216 | -0.84653 | 0.147698 | 0.00019 | 0.027425 | ERCC6 |
| ENSTGUG00000012385 | -0.84527 | 0.209068 | 0.00235 | 0.076154 | ENSTGUG00000012385 |
| ENSTGUG00000007060 | -0.84495 | 0.206351 | 0.002163 | 0.074042 | ST6GALNAC3 |
| ENSTGUG00000005284 | -0.8432 | 0.196807 | 0.0016 | 0.065599 | TTL |
| ENSTGUG00000006629 | -0.83844 | 0.231834 | 0.004716 | 0.096156 | KAT6B |
| ENSTGUG00000013637 | -0.83842 | 0.226477 | 0.004095 | 0.091371 | DCBLD2 |
| ENSTGUG00000013611 | -0.83518 | 0.222456 | 0.003757 | 0.088944 | ENSTGUG00000013611 |
| ENSTGUG00000009088 | -0.83414 | 0.188644 | 0.001291 | 0.06007 | ATP13A3 |
| ENSTGUG00000005294 | -0.83194 | 0.213518 | 0.002979 | 0.082622 | BBS9 |
| ENSTGUG00000008732 | -0.83094 | 0.13654 | 0.000118 | 0.024107 | HEATR5B |
| ENSTGUG00000008704 | -0.8243 | 0.201541 | 0.002179 | 0.074042 | SYNM |
| ENSTGUG00000008450 | -0.82236 | 0.198689 | 0.002015 | 0.07195 | TFDP2 |
| ENSTGUG00000005382 | -0.81655 | 0.205198 | 0.002604 | 0.078344 | KAT6A |
| ENSTGUG00000011986 | -0.81014 | 0.224547 | 0.004785 | 0.096955 | RALGAPA1 |
| ENSTGUG00000012658 | -0.81004 | 0.158814 | 0.000464 | 0.042738 | DICER1 |
| ENSTGUG00000002640 | -0.8087 | 0.165975 | 0.000649 | 0.048382 | SLC10A7 |
| ENSTGUG00000008274 | -0.80538 | 0.197919 | 0.002253 | 0.075135 | ZDHHC8 |
| ENSTGUG00000001255 | -0.80487 | 0.11584 | 3.96E-05 | 0.015889 | PCBD2 |
| ENSTGUG00000011879 | -0.80461 | 0.187169 | 0.001564 | 0.064826 | CEP85L |
| ENSTGUG00000011347 | -0.80255 | 0.196911 | 0.00223 | 0.07501 | LATS1 |
| ENSTGUG00000009532 | -0.80249 | 0.171305 | 0.000862 | 0.050993 | PDPK1 |
| ENSTGUG00000005069 | -0.80118 | 0.204843 | 0.002908 | 0.082158 | EXTL2 |
| ENSTGUG00000000733 | -0.79854 | 0.176297 | 0.001092 | 0.056196 | ENSTGUG00000000733 |
| ENSTGUG00000013619 | -0.79393 | 0.169164 | 0.00085 | 0.050993 | ZBTB11 |
| ENSTGUG00000000374 | -0.79321 | 0.108345 | 2.54E-05 | 0.013589 | ENSTGUG00000000374 |
| ENSTGUG00000013193 | -0.79047 | 0.203685 | 0.003055 | 0.08298 | ITSN2 |
| ENSTGUG00000010484 | -0.78821 | 0.210315 | 0.003798 | 0.089035 | MCF2L2 |
| ENSTGUG00000012607 | -0.78351 | 0.197952 | 0.002695 | 0.079545 | ITPK1 |
| ENSTGUG00000001352 | -0.78139 | 0.211867 | 0.00419 | 0.09203 | FAM171A1 |
| ENSTGUG00000005196 | -0.77155 | 0.205179 | 0.00372 | 0.088944 | ENSTGUG00000005196 |
| ENSTGUG00000005577 | -0.77131 | 0.204537 | 0.003655 | 0.088944 | TTLL4 |
| ENSTGUG00000010791 | -0.7634 | 0.182552 | 0.001882 | 0.070406 | ENSTGUG00000010791 |
| ENSTGUG00000009194 | -0.76077 | 0.194302 | 0.002888 | 0.082158 | PLEKHA3 |
| ENSTGUG00000000031 | -0.7607 | 0.208293 | 0.004447 | 0.093822 | GFRA3 |
| ENSTGUG00000008398 | -0.75732 | 0.157475 | 0.000714 | 0.048979 | FGD6 |
| ENSTGUG00000001220 | -0.75524 | 0.17709 | 0.001651 | 0.065599 | ENSTGUG00000001220 |
| ENSTGUG00000002495 | -0.75443 | 0.15278 | 0.000589 | 0.046941 | DAAM2 |
| ENSTGUG00000009266 | -0.75415 | 0.19565 | 0.003188 | 0.084885 | CCDC102B |
| ENSTGUG00000005117 | -0.75346 | 0.161708 | 0.000895 | 0.051706 | SETX |
| ENSTGUG00000008212 | -0.75017 | 0.163864 | 0.001014 | 0.053782 | AKT3 |
| ENSTGUG00000000468 | -0.7461 | 0.171409 | 0.001437 | 0.062898 | ENSTGUG00000000468 |
| ENSTGUG00000009124 | -0.74529 | 0.186879 | 0.002567 | 0.07825 | ZNF407 |
| ENSTGUG00000001556 | -0.74429 | 0.186062 | 0.002517 | 0.07825 | CASD1 |
| ENSTGUG00000011751 | -0.74013 | 0.166838 | 0.001262 | 0.05988 | ETNK1 |
| ENSTGUG00000003310 | -0.73908 | 0.197068 | 0.003781 | 0.089035 | PIGU |
| ENSTGUG00000006371 | -0.73547 | 0.184842 | 0.002605 | 0.078344 | PKD1 |
| ENSTGUG00000010567 | -0.72624 | 0.202669 | 0.004984 | 0.09867 | ANKRD44 |
| ENSTGUG00000011244 | -0.72436 | 0.16159 | 0.001174 | 0.057761 | ZC3H12C |
| ENSTGUG00000010931 | -0.71729 | 0.099528 | 2.90E-05 | 0.014225 | MAPKBP1 |
| ENSTGUG00000001850 | -0.7152 | 0.183577 | 0.002981 | 0.082622 | CDK13 |
| ENSTGUG00000007676 | -0.71427 | 0.122592 | 0.000167 | 0.026259 | FBXL18 |
| ENSTGUG00000006645 | -0.71391 | 0.130843 | 0.000278 | 0.032956 | ENSTGUG00000006645 |
| ENSTGUG00000010994 | -0.7096 | 0.161896 | 0.001371 | 0.061914 | EHD4 |
| ENSTGUG00000010007 | -0.70892 | 0.18418 | 0.003217 | 0.084885 | ENSTGUG00000010007 |
| ENSTGUG00000001723 | -0.70643 | 0.196195 | 0.004843 | 0.097077 | CCNI |
| ENSTGUG00000012785 | -0.69954 | 0.180021 | 0.00303 | 0.082745 | PPP2R5C |
| ENSTGUG00000013598 | -0.69935 | 0.19181 | 0.004491 | 0.093822 | PLCXD2 |
| ENSTGUG00000011910 | -0.69889 | 0.170465 | 0.002145 | 0.074042 | DPY19L4 |
| ENSTGUG00000010295 | -0.69557 | 0.171072 | 0.002265 | 0.075135 | SYT13 |
| ENSTGUG00000007664 | -0.69515 | 0.156755 | 0.001265 | 0.05988 | SEMA5A |
| ENSTGUG00000009747 | -0.69429 | 0.187408 | 0.004077 | 0.091371 | RAPH1 |
| ENSTGUG00000004849 | -0.68976 | 0.164168 | 0.001824 | 0.06954 | CLEC16A |
| ENSTGUG00000004532 | -0.68838 | 0.163183 | 0.001776 | 0.068636 | ANO5 |
| ENSTGUG00000011456 | -0.68595 | 0.179471 | 0.003362 | 0.086522 | CAPRIN2 |
| ENSTGUG00000003427 | -0.6825 | 0.167849 | 0.002264 | 0.075135 | AAR2 |
| ENSTGUG00000011160 | -0.6816 | 0.081242 | 7.74E-06 | 0.010222 | TTBK2 |
| ENSTGUG00000013363 | -0.68039 | 0.14077 | 0.000688 | 0.048382 | ENSTGUG00000013363 |
| ENSTGUG00000012993 | -0.66787 | 0.169529 | 0.002777 | 0.080692 | SIX4 |
| ENSTGUG00000003517 | -0.66538 | 0.163448 | 0.002247 | 0.075135 | THADA |
| ENSTGUG00000005397 | -0.65764 | 0.119593 | 0.000262 | 0.032398 | TMLHE |
| ENSTGUG00000009009 | -0.65419 | 0.182776 | 0.005018 | 0.099187 | BTAF1 |
| ENSTGUG00000004350 | -0.64289 | 0.148357 | 0.001482 | 0.064249 | DENND4C |
| ENSTGUG00000012360 | -0.63561 | 0.17435 | 0.004495 | 0.093822 | MAP3K7 |
| ENSTGUG00000008478 | -0.63486 | 0.154368 | 0.002102 | 0.073932 | GK5 |
| ENSTGUG00000012803 | -0.63209 | 0.111667 | 0.000209 | 0.029438 | ENSTGUG00000012803 |
| ENSTGUG00000011100 | -0.63058 | 0.161897 | 0.002986 | 0.082622 | PLAG1 |
| ENSTGUG00000011912 | -0.62411 | 0.169379 | 0.004213 | 0.092188 | ENSTGUG00000011912 |
| ENSTGUG00000006453 | -0.6138 | 0.150648 | 0.002234 | 0.07501 | SSH2 |
| ENSTGUG00000006542 | -0.61055 | 0.141092 | 0.001496 | 0.064249 | ACAD10 |
| ENSTGUG00000013424 | -0.60564 | 0.160848 | 0.00369 | 0.088944 | SLC35A5 |
| ENSTGUG00000002041 | -0.60211 | 0.131419 | 0.001008 | 0.053782 | ANKRD50 |
| ENSTGUG00000012530 | -0.59869 | 0.166253 | 0.004839 | 0.097077 | TBC1D4 |
| ENSTGUG00000003993 | -0.59479 | 0.139147 | 0.001625 | 0.065599 | TUBGCP6 |
| ENSTGUG00000000459 | -0.58758 | 0.160628 | 0.004403 | 0.093787 | SLC5A3 |
| ENSTGUG00000012442 | -0.58391 | 0.079215 | 2.39E-05 | 0.013462 | FBXO32 |
| ENSTGUG00000006146 | -0.58335 | 0.100181 | 0.000168 | 0.026259 | KIF13A |
| ENSTGUG00000011316 | -0.58192 | 0.144133 | 0.002371 | 0.076629 | SIPA1L1 |
| ENSTGUG00000004827 | -0.5816 | 0.142086 | 0.002168 | 0.074042 | PHKA1 |
| ENSTGUG00000012962 | -0.57478 | 0.127015 | 0.001099 | 0.056196 | PXDN |
| ENSTGUG00000010558 | -0.57256 | 0.156952 | 0.004477 | 0.093822 | WHSC1 |
| ENSTGUG00000007065 | -0.57218 | 0.112846 | 0.000484 | 0.04394 | SEMA6D |
| ENSTGUG00000012448 | -0.56165 | 0.154736 | 0.004615 | 0.094878 | LECT1 |
| ENSTGUG00000003164 | -0.53909 | 0.128662 | 0.001858 | 0.070357 | BMPR1B |
| ENSTGUG00000006245 | -0.52266 | 0.088226 | 0.000146 | 0.025669 | ENSTGUG00000006245 |
| ENSTGUG00000011488 | -0.51624 | 0.138038 | 0.003848 | 0.089587 | AHI1 |
| ENSTGUG00000006214 | -0.50982 | 0.104721 | 0.000653 | 0.048382 | PLCB4 |
| ENSTGUG00000009101 | -0.50032 | 0.107757 | 0.000918 | 0.052138 | ADGRA1 |
| ENSTGUG00000000155 | -0.49391 | 0.123489 | 0.00252 | 0.07825 | PIP5K1C |
| ENSTGUG00000012592 | -0.48568 | 0.061136 | 1.25E-05 | 0.011566 | PHIP |
| ENSTGUG00000009373 | -0.47827 | 0.100449 | 0.000767 | 0.050438 | MBTD1 |
| ENSTGUG00000009850 | -0.47755 | 0.101389 | 0.000829 | 0.050993 | DEPDC5 |
| ENSTGUG00000002729 | -0.47185 | 0.126957 | 0.003997 | 0.09046 | FAM126A |
| ENSTGUG00000006018 | -0.45397 | 0.106441 | 0.00165 | 0.065599 | UNC119 |
| ENSTGUG00000002449 | -0.41968 | 0.106629 | 0.002793 | 0.080757 | SEMA3A |
| ENSTGUG00000001508 | -0.40256 | 0.106027 | 0.003504 | 0.088164 | ENSTGUG00000001508 |
| ENSTGUG00000012717 | -0.3984 | 0.066012 | 0.000126 | 0.024586 | LRP6 |
| ENSTGUG00000013237 | -0.38671 | 0.077725 | 0.000557 | 0.045736 | ENSTGUG00000013237 |
| ENSTGUG00000000276 | -0.37999 | 0.092454 | 0.00211 | 0.073932 | NKTR |
| ENSTGUG00000001978 | -0.37297 | 0.092855 | 0.002451 | 0.078095 | ENSTGUG00000001978 |
| ENSTGUG00000010910 | -0.35925 | 0.045909 | 1.43E-05 | 0.011566 | NHSL1 |
| ENSTGUG00000008597 | -0.34492 | 0.079217 | 0.001434 | 0.062898 | ELAVL4 |
| ENSTGUG00000000530 | -0.34074 | 0.090487 | 0.003688 | 0.088944 | PRDM10 |
| ENSTGUG00000008434 | -0.3024 | 0.077452 | 0.00294 | 0.082321 | PLXNB1 |
| ENSTGUG00000011732 | -0.29134 | 0.079692 | 0.004419 | 0.093822 | LAMA2 |
| ENSTGUG00000001899 | -0.28928 | 0.074827 | 0.00313 | 0.084614 | ENSTGUG00000001899 |
| ENSTGUG00000012778 | -0.27962 | 0.064719 | 0.001512 | 0.064249 | PGR |
| ENSTGUG00000012688 | -0.2793 | 0.049598 | 0.000218 | 0.029898 | KCNQ5 |
| ENSTGUG00000012711 | -0.26979 | 0.059873 | 0.001133 | 0.057371 | BCL11B |
| ENSTGUG00000011400 | -0.25844 | 0.070276 | 0.004264 | 0.092575 | PREX2 |
| ENSTGUG00000001933 | -0.20925 | 0.046226 | 0.001097 | 0.056196 | ADAMTS12 |
