# Supplementary material for: Transcriptional Signatures in Liver Reveal Metabolic Adaptations to Seasons in Migratory Blackheaded Buntings
Source: Front Physiol. 2018 Nov 27;9:1568. doi: 10.3389/fphys.2018.01568 (PMC6277527; doi:10.3389/fphys.2018.01568)
Supplement: Table S3 — List of GO terms in blue and turquoise modules derived from WGCNA analysis. [file Table_3.DOCX]

**Suppl. Table 3.** **List of GO terms derived from blue and turquoise module**

Blue module GO terms

| **Go terms** | **total** | **expectation** | **observation** | **hypergeometric** | **fisher** |
| --- | --- | --- | --- | --- | --- |
| ribosome | 98 | 9 | 39 | 9.57E-16 | 9.57E-16 |
| poly(A) RNA binding | 636 | 60 | 118 | 3.14E-13 | 4.80E-13 |
| translation | 131 | 12 | 40 | 1.00E-11 | 1.00E-11 |
| ribonucleoprotein complex | 103 | 10 | 33 | 1.59E-10 | 1.59E-10 |
| mitochondrion | 738 | 70 | 122 | 3.63E-10 | 4.69E-10 |
| extracellular exosome | 1269 | 121 | 166 | 5.94E-06 | 9.31E-06 |
| proteasome complex | 25 | 2 | 10 | 4.98E-05 | 4.98E-05 |
| ribosome biogenesis | 19 | 2 | 8 | 0.000189 | 0.000189 |
| nucleic acid binding | 538 | 51 | 77 | 0.00015 | 0.000212 |
| oxidation-reduction process | 400 | 38 | 60 | 0.00023 | 0.000341 |
| ATP synthesis coupled proton transport | 12 | 1 | 6 | 0.000406 | 0.000406 |
| metabolic process | 765 | 73 | 100 | 0.000513 | 0.000866 |
| negative regulation of protein transport | 5 | 0 | 3 | 0.007395 | 0.007395 |
| ER membrane protein complex | 5 | 0 | 3 | 0.007395 | 0.007395 |
| positive regulation of phagocytosis | 14 | 1 | 5 | 0.00741 | 0.00741 |
| ATP hydrolysis coupled proton transport | 20 | 2 | 6 | 0.008776 | 0.008776 |
| oxidoreductase activity | 283 | 27 | 40 | 0.006888 | 0.010028 |
| fatty acid metabolic process | 27 | 3 | 7 | 0.011171 | 0.011171 |
| proton transport | 21 | 2 | 6 | 0.011318 | 0.011318 |
| proteolysis involved in cellular protein catabolic process | 21 | 2 | 6 | 0.011318 | 0.011318 |
| negative regulation of glycolytic process | 6 | 1 | 3 | 0.013749 | 0.013749 |
| methylosome | 6 | 1 | 3 | 0.013749 | 0.013749 |
| tricarboxylic acid cycle | 11 | 1 | 4 | 0.015531 | 0.015531 |
| biosynthetic process | 43 | 4 | 9 | 0.017892 | 0.017892 |
| ER to Golgi transport vesicle | 7 | 1 | 3 | 0.022376 | 0.022376 |
| methyltransferase activity | 75 | 7 | 13 | 0.023489 | 0.028346 |
| glycolytic process | 19 | 2 | 5 | 0.028891 | 0.028891 |
| carbohydrate metabolic process | 107 | 10 | 17 | 0.023745 | 0.030517 |
| NAD biosynthetic process | 8 | 1 | 3 | 0.033308 | 0.033308 |
| lipid metabolic process | 88 | 8 | 3 | 0.992177 | 0.045061 |
| regulation of mitophagy | 15 | 1 | 4 | 0.04734 | 0.04734 |
| positive regulation of TOR signaling | 15 | 1 | 4 | 0.04734 | 0.04734 |
| hydrolase activity | 495 | 47 | 60 | 0.028506 | 0.049596 |

Turquoise module GO terms

| **GO terms** | **total** | **expectation** | **observation** | **hypergeometric** | **fisher** |
| --- | --- | --- | --- | --- | --- |
| binding | 275 | 30 | 66 | 4.22E-10 | 4.87E-10 |
| protein binding | 5632 | 618 | 719 | 6.11E-10 | 1.04E-09 |
| nucleus | 1965 | 216 | 284 | 8.50E-08 | 1.46E-07 |
| metal ion binding | 905 | 99 | 148 | 1.99E-07 | 2.96E-07 |
| histone acetylation | 15 | 2 | 9 | 6.06E-06 | 6.06E-06 |
| transcription coactivator activity | 77 | 8 | 22 | 1.70E-05 | 1.70E-05 |
| ubiquitin-dependent protein catabolic process | 99 | 11 | 26 | 1.60E-05 | 2.55E-05 |
| nucleic acid binding | 538 | 59 | 90 | 2.37E-05 | 3.72E-05 |
| Golgi organization | 33 | 4 | 12 | 0.000113 | 0.000113 |
| histone acetyltransferase activity | 21 | 2 | 9 | 0.000193 | 0.000193 |
| chromatin binding | 209 | 23 | 41 | 0.000147 | 0.000193 |
| protein tyrosine kinase activity | 326 | 36 | 58 | 0.000127 | 0.0002 |
| DNA binding | 605 | 66 | 95 | 0.000167 | 0.000287 |
| GTPase activator activity | 88 | 10 | 21 | 0.000431 | 0.000466 |
| endosome to lysosome transport | 19 | 2 | 8 | 0.000511 | 0.000511 |
| histone methyltransferase activity (H3-K4 specific) | 8 | 1 | 5 | 0.000665 | 0.000665 |
| ubiquitinyl hydrolase activity | 42 | 5 | 12 | 0.001388 | 0.001388 |
| cis-Golgi network | 22 | 2 | 8 | 0.0016 | 0.0016 |
| protein phosphorylation | 412 | 45 | 66 | 0.000956 | 0.001645 |
| transferase activity, transferring phosphorus-containing groups | 393 | 43 | 63 | 0.001214 | 0.001751 |
| regulation of transcription, DNA-templated | 461 | 51 | 72 | 0.001173 | 0.001762 |
| protein targeting to Golgi | 6 | 1 | 4 | 0.001803 | 0.001803 |
| pre-autophagosomal structure membrane | 6 | 1 | 4 | 0.001803 | 0.001803 |
| histone binding | 38 | 4 | 11 | 0.001938 | 0.001938 |
| positive regulation of TOR signaling | 15 | 2 | 6 | 0.003596 | 0.003596 |
| methylated histone binding | 20 | 2 | 7 | 0.004021 | 0.004021 |
| acetyltransferase activity | 11 | 1 | 5 | 0.004131 | 0.004131 |
| ubiquitin-protein transferase activity | 129 | 14 | 25 | 0.00322 | 0.004199 |
| vesicle-mediated transport | 96 | 11 | 20 | 0.003393 | 0.004525 |
| protein kinase binding | 189 | 21 | 33 | 0.004593 | 0.006583 |
| retrograde transport, endosome to Golgi | 27 | 3 | 8 | 0.006755 | 0.006755 |
| DNA methylation | 8 | 1 | 4 | 0.00702 | 0.00702 |
| lipid particle | 22 | 2 | 7 | 0.007274 | 0.007274 |
| histone H3 acetylation | 29 | 3 | 8 | 0.010717 | 0.010717 |
| insulin binding | 5 | 1 | 3 | 0.011113 | 0.011113 |
| insulin receptor substrate binding | 5 | 1 | 3 | 0.011113 | 0.011113 |
| Golgi-associated vesicle | 5 | 1 | 3 | 0.011113 | 0.011113 |
| gene silencing by miRNA | 5 | 1 | 3 | 0.011113 | 0.011113 |
| methyl-CpG binding | 5 | 1 | 3 | 0.011113 | 0.011113 |
| regulation of TOR signaling | 6 | 1 | 3 | 0.020422 | 0.020422 |
| MAP kinase kinase kinase activity | 6 | 1 | 3 | 0.020422 | 0.020422 |
| autophagy | 27 | 3 | 7 | 0.023313 | 0.023313 |
| Golgi apparatus | 400 | 44 | 58 | 0.015783 | 0.02742 |
| positive regulation of transcription from RNA polymerase II promoter | 420 | 46 | 60 | 0.018999 | 0.0314 |
| protein O-linked glycosylation | 17 | 2 | 5 | 0.031674 | 0.031674 |
| protein localization to organelle | 7 | 1 | 3 | 0.032856 | 0.032856 |
| glycoprotein binding | 24 | 3 | 6 | 0.041061 | 0.041061 |
| protein stabilization | 43 | 5 | 9 | 0.040808 | 0.047494 |
| pre-autophagosomal structure | 8 | 1 | 3 | 0.048358 | 0.048358 |
| positive regulation of proteasomal ubiquitin-dependent protein catabolic process | 25 | 3 | 6 | 0.049192 | 0.049192 |
