# Supplementary material for: Transcriptional Signatures in Liver Reveal Metabolic Adaptations to Seasons in Migratory Blackheaded Buntings
Source: Front Physiol. 2018 Nov 27;9:1568. doi: 10.3389/fphys.2018.01568 (PMC6277527; doi:10.3389/fphys.2018.01568)
Supplement: Table S4 — List of cycling genes under migratory and non-migratory states. A gene was characterized as cyclic with threshold of adjusted P-value (ADJP < 0.05). The wave characteristics of cyclic genes was categorized as period, peak expression time (Lag), and amplitude. [file Table_4.DOCX]

Table 4. List of cycling genes

Migratory state specific cycling genes

| **EnsemblGeneID** | **GeneSymbol** | **BHQ** | **ADJP** | **PERIOD** | **LAG** | **AMP** |
| --- | --- | --- | --- | --- | --- | --- |
| ENSTGUG00000014743 |  | 0.035 | 0.002 | 26.0 | 18.0 | 1.21 |
| ENSTGUG00000002888 | COG1 | 0.035 | 0.002 | 26.0 | 15.0 | 1.20 |
| ENSTGUG00000008153 | ZNF143 | 0.035 | 0.002 | 26.0 | 2.0 | 1.18 |
| ENSTGUG00000006800 | SRXN1 | 0.035 | 0.002 | 24.0 | 14.0 | 1.18 |
| ENSTGUG00000001662 | LHFPL5 | 0.035 | 0.002 | 24.0 | 14.0 | 1.16 |
| ENSTGUG00000011604 | PDX1 | 0.035 | 0.002 | 24.0 | 2.0 | 1.15 |
| ENSTGUG00000007115 | ZGPAT | 0.035 | 0.002 | 24.0 | 14.0 | 1.15 |
| ENSTGUG00000010278 | TAF5 | 0.035 | 0.002 | 26.0 | 2.0 | 1.12 |
| ENSTGUG00000015960 |  | 0.035 | 0.002 | 24.0 | 5.0 | 1.10 |
| ENSTGUG00000008404 | C3orf19 | 0.035 | 0.002 | 26.0 | 2.0 | 1.09 |
| ENSTGUG00000000702 | NOM1 | 0.035 | 0.002 | 24.0 | 20.0 | 1.08 |
| ENSTGUG00000007505 | YPEL2 | 0.035 | 0.002 | 24.0 | 2.0 | 1.07 |
| ENSTGUG00000002524 | SLC4A1AP | 0.035 | 0.002 | 24.0 | 20.0 | 1.06 |
| ENSTGUG00000006380 | ZCCHC24 | 0.035 | 0.002 | 24.0 | 6.0 | 1.06 |
| ENSTGUG00000009217 | SOCS6 | 0.035 | 0.002 | 24.0 | 2.0 | 1.06 |
| ENSTGUG00000012794 | RAB23 | 0.035 | 0.002 | 25.3 | 4.0 | 1.06 |
| ENSTGUG00000001534 | C1orf109 | 0.035 | 0.002 | 24.0 | 18.0 | 1.05 |
| ENSTGUG00000009959 | BLM | 0.035 | 0.002 | 26.0 | 15.0 | 1.04 |
| ENSTGUG00000006636 | SLCO2A1 | 0.035 | 0.002 | 26.0 | 15.0 | 1.04 |
| ENSTGUG00000013239 | KLHL28 | 0.035 | 0.002 | 24.0 | 14.0 | 1.04 |
| ENSTGUG00000002342 | DCLRE1C | 0.035 | 0.002 | 24.0 | 5.0 | 1.03 |
| ENSTGUG00000011558 | PTPRE | 0.035 | 0.002 | 24.0 | 16.0 | 1.03 |
| ENSTGUG00000007059 | AKAP10 | 0.035 | 0.002 | 26.0 | 2.0 | 1.01 |
| ENSTGUG00000002430 | SETD7 | 0.035 | 0.002 | 25.3 | 4.0 | 1.00 |
| ENSTGUG00000003786 | RAB17 | 0.035 | 0.002 | 25.3 | 16.7 | 0.97 |
| ENSTGUG00000015853 | ME2 | 0.035 | 0.002 | 24.0 | 9.0 | 0.96 |
| ENSTGUG00000004796 | ZNF319 | 0.035 | 0.002 | 24.0 | 8.0 | 0.96 |
| ENSTGUG00000018254 |  | 0.035 | 0.002 | 24.0 | 8.0 | 0.95 |
| ENSTGUG00000002777 | WNK4 | 0.035 | 0.002 | 24.0 | 10.0 | 0.95 |
| ENSTGUG00000013414 | ILDR1 | 0.035 | 0.002 | 24.0 | 14.0 | 0.95 |
| ENSTGUG00000003600 | SH2D3C | 0.035 | 0.002 | 26.0 | 15.0 | 0.94 |
| ENSTGUG00000003314 | MTMR4 | 0.035 | 0.002 | 24.0 | 5.0 | 0.94 |
| ENSTGUG00000013001 | NARS2 | 0.035 | 0.002 | 24.0 | 14.0 | 0.93 |
| ENSTGUG00000009340 | CWC22 | 0.035 | 0.002 | 25.3 | 4.0 | 0.92 |
| ENSTGUG00000006536 | DCTD | 0.035 | 0.002 | 24.0 | 8.0 | 0.90 |
| ENSTGUG00000004032 |  | 0.035 | 0.002 | 24.0 | 6.0 | 0.90 |
| ENSTGUG00000014770 | FMNL3 | 0.035 | 0.002 | 24.0 | 12.0 | 0.89 |
| ENSTGUG00000012958 | WDR89 | 0.035 | 0.002 | 24.0 | 13.0 | 0.89 |
| ENSTGUG00000009370 | HIST1H1A-3 | 0.035 | 0.002 | 26.0 | 15.0 | 0.88 |
| ENSTGUG00000007360 | ANAPC1 | 0.035 | 0.002 | 24.0 | 14.0 | 0.87 |
| ENSTGUG00000009277 | HIST1H1A-1 | 0.035 | 0.002 | 26.0 | 15.0 | 0.86 |
| ENSTGUG00000006351 | TMEM132A | 0.035 | 0.002 | 24.0 | 13.0 | 0.86 |
| ENSTGUG00000006489 |  | 0.035 | 0.002 | 25.3 | 16.7 | 0.86 |
| ENSTGUG00000010311 | FUT8 | 0.035 | 0.002 | 24.0 | 14.0 | 0.86 |
| ENSTGUG00000011447 | HPS3 | 0.035 | 0.002 | 26.0 | 2.0 | 0.84 |
| ENSTGUG00000003830 | ANO10 | 0.035 | 0.002 | 26.0 | 2.0 | 0.84 |
| ENSTGUG00000000326 | FDXACB1 | 0.035 | 0.002 | 24.0 | 18.0 | 0.84 |
| ENSTGUG00000004900 |  | 0.035 | 0.002 | 26.0 | 15.0 | 0.84 |
| ENSTGUG00000003190 | TNFSF15 | 0.035 | 0.002 | 24.0 | 6.0 | 0.84 |
| ENSTGUG00000012937 | ERICH1 | 0.035 | 0.002 | 24.0 | 5.0 | 0.83 |
| ENSTGUG00000011171 | SMC4 | 0.035 | 0.002 | 24.0 | 14.0 | 0.80 |
| ENSTGUG00000000907 | E2F2 | 0.035 | 0.002 | 25.3 | 16.7 | 0.79 |
| ENSTGUG00000002028 | PHLPP | 0.035 | 0.002 | 24.0 | 5.0 | 0.78 |
| ENSTGUG00000008510 | TNKS2 | 0.035 | 0.002 | 24.0 | 2.0 | 0.78 |
| ENSTGUG00000014804 | MAP1S | 0.035 | 0.002 | 24.0 | 12.0 | 0.78 |
| ENSTGUG00000017526 | RAD17 | 0.035 | 0.002 | 24.0 | 20.0 | 0.77 |
| ENSTGUG00000000615 | LSM11 | 0.035 | 0.002 | 24.0 | 18.0 | 0.77 |
| ENSTGUG00000001029 | REEP5 | 0.035 | 0.002 | 24.0 | 16.0 | 0.77 |
| ENSTGUG00000015696 |  | 0.035 | 0.002 | 24.0 | 4.0 | 0.76 |
| ENSTGUG00000008235 | ATP10D | 0.035 | 0.002 | 24.0 | 4.0 | 0.75 |
| ENSTGUG00000001442 | FZD1 | 0.035 | 0.002 | 24.0 | 13.0 | 0.74 |
| ENSTGUG00000018157 |  | 0.035 | 0.002 | 26.0 | 15.0 | 0.74 |
| ENSTGUG00000013617 | CEP97 | 0.035 | 0.002 | 25.3 | 4.0 | 0.69 |
| ENSTGUG00000017496 | DSTYK | 0.035 | 0.002 | 24.0 | 8.0 | 0.69 |
| ENSTGUG00000004788 | SAMD9L | 0.035 | 0.002 | 25.3 | 4.0 | 0.67 |
| ENSTGUG00000000425 |  | 0.035 | 0.002 | 24.0 | 14.0 | 0.66 |
| ENSTGUG00000010257 |  | 0.035 | 0.002 | 24.0 | 5.0 | 0.65 |
| ENSTGUG00000006310 | ZNF280D | 0.035 | 0.002 | 24.0 | 2.0 | 0.65 |
| ENSTGUG00000017283 |  | 0.035 | 0.002 | 24.0 | 14.0 | 0.65 |
| ENSTGUG00000016314 | FLOT1 | 0.035 | 0.002 | 24.0 | 16.0 | 0.64 |
| ENSTGUG00000009376 | PACRG | 0.035 | 0.002 | 25.3 | 16.7 | 0.64 |
| ENSTGUG00000016554 |  | 0.035 | 0.002 | 24.0 | 18.0 | 0.63 |
| ENSTGUG00000007948 |  | 0.035 | 0.002 | 26.0 | 18.0 | 0.63 |
| ENSTGUG00000016356 |  | 0.035 | 0.002 | 26.0 | 2.0 | 0.61 |
| ENSTGUG00000015038 |  | 0.035 | 0.002 | 24.0 | 4.0 | 0.61 |
| ENSTGUG00000003173 | CBLL1 | 0.035 | 0.002 | 24.0 | 22.0 | 0.61 |
| ENSTGUG00000004403 | CADPS2 | 0.035 | 0.002 | 24.0 | 14.0 | 0.59 |
| ENSTGUG00000001475 | GATAD1 | 0.035 | 0.002 | 24.0 | 17.0 | 0.58 |
| ENSTGUG00000011016 | MTERFD3 | 0.035 | 0.002 | 24.0 | 6.0 | 0.57 |
| ENSTGUG00000006054 | JARID2 | 0.035 | 0.002 | 24.0 | 4.0 | 0.56 |
| ENSTGUG00000001071 | YOD1 | 0.035 | 0.002 | 24.0 | 10.0 | 0.55 |
| ENSTGUG00000001919 | TSGA14 | 0.035 | 0.002 | 26.0 | 15.0 | 0.55 |
| ENSTGUG00000015748 | MAP2K6 | 0.035 | 0.002 | 24.0 | 8.0 | 0.54 |
| ENSTGUG00000009791 | FRMD4B | 0.035 | 0.002 | 24.0 | 2.0 | 0.54 |
| ENSTGUG00000000997 | ARID1A | 0.035 | 0.002 | 24.0 | 6.0 | 0.54 |
| ENSTGUG00000001758 | DNAJB5 | 0.035 | 0.002 | 26.0 | 2.0 | 0.53 |
| ENSTGUG00000002511 |  | 0.035 | 0.002 | 24.0 | 6.0 | 0.52 |
| ENSTGUG00000003141 | WDR44 | 0.035 | 0.002 | 24.0 | 2.0 | 0.52 |
| ENSTGUG00000004316 | SH3GLB2 | 0.035 | 0.002 | 24.0 | 12.0 | 0.51 |
| ENSTGUG00000015948 | PPP1R10 | 0.035 | 0.002 | 26.0 | 18.0 | 0.51 |
| ENSTGUG00000006103 | SLC38A1 | 0.035 | 0.002 | 24.0 | 20.0 | 0.49 |
| ENSTGUG00000010596 | RGS7 | 0.035 | 0.002 | 24.0 | 20.0 | 0.49 |
| ENSTGUG00000009291 |  | 0.035 | 0.002 | 25.3 | 16.7 | 0.48 |
| ENSTGUG00000000955 |  | 0.035 | 0.002 | 26.0 | 15.0 | 0.47 |
| ENSTGUG00000015019 |  | 0.035 | 0.002 | 24.0 | 22.0 | 0.47 |
| ENSTGUG00000015488 | SMARCA4 | 0.035 | 0.002 | 24.0 | 14.0 | 0.47 |
| ENSTGUG00000004619 | PATL1 | 0.035 | 0.002 | 25.3 | 16.7 | 0.47 |
| ENSTGUG00000009672 | IQCG | 0.035 | 0.002 | 24.0 | 20.0 | 0.47 |
| ENSTGUG00000002728 | KIAA0562 | 0.035 | 0.002 | 26.0 | 15.0 | 0.46 |
| ENSTGUG00000001589 | DYNC1I1 | 0.035 | 0.002 | 24.0 | 4.0 | 0.46 |
| ENSTGUG00000000781 | UHRF1 | 0.035 | 0.002 | 24.0 | 12.0 | 0.46 |
| ENSTGUG00000011749 | SOX5 | 0.035 | 0.002 | 24.0 | 1.0 | 0.45 |
| ENSTGUG00000008933 | SHCBP1 | 0.035 | 0.002 | 26.0 | 15.0 | 0.45 |
| ENSTGUG00000002134 | GATA3 | 0.035 | 0.002 | 24.0 | 17.0 | 0.45 |
| ENSTGUG00000008969 | CDR2 | 0.035 | 0.002 | 24.0 | 10.0 | 0.44 |
| ENSTGUG00000016568 |  | 0.035 | 0.002 | 26.0 | 2.0 | 0.44 |
| ENSTGUG00000002559 | DCLK2 | 0.035 | 0.002 | 25.3 | 4.0 | 0.44 |
| ENSTGUG00000005368 | RILPL2 | 0.035 | 0.002 | 24.0 | 17.0 | 0.44 |
| ENSTGUG00000005346 | VAMP2 | 0.035 | 0.002 | 24.0 | 16.0 | 0.44 |
| ENSTGUG00000000314 | C5orf41 | 0.035 | 0.002 | 24.0 | 2.0 | 0.44 |
| ENSTGUG00000017090 | DDR2 | 0.035 | 0.002 | 24.0 | 16.0 | 0.44 |
| ENSTGUG00000018231 |  | 0.035 | 0.002 | 24.0 | 2.0 | 0.43 |
| ENSTGUG00000006038 | MTSS1L | 0.035 | 0.002 | 24.0 | 10.0 | 0.43 |
| ENSTGUG00000003797 | LRRFIP1 | 0.035 | 0.002 | 24.0 | 8.0 | 0.42 |
| ENSTGUG00000013328 | LPAR5 | 0.035 | 0.002 | 25.3 | 16.7 | 0.42 |
| ENSTGUG00000010108 |  | 0.035 | 0.002 | 24.0 | 6.0 | 0.42 |
| ENSTGUG00000001497 | DGKQ | 0.035 | 0.002 | 26.0 | 5.0 | 0.42 |
| ENSTGUG00000011271 | SLC33A1 | 0.035 | 0.002 | 24.0 | 12.0 | 0.40 |
| ENSTGUG00000011862 | TMEM67 | 0.035 | 0.002 | 24.0 | 9.0 | 0.40 |
| ENSTGUG00000016143 |  | 0.035 | 0.002 | 24.0 | 14.0 | 0.40 |
| ENSTGUG00000007860 | RHBDF2 | 0.035 | 0.002 | 24.0 | 10.0 | 0.40 |
| ENSTGUG00000008368 | HEATR6 | 0.035 | 0.002 | 24.0 | 12.0 | 0.39 |
| ENSTGUG00000001806 | EGFR | 0.035 | 0.002 | 24.0 | 2.0 | 0.37 |
| ENSTGUG00000003391 | TMPRSS12 | 0.035 | 0.002 | 24.0 | 17.0 | 0.36 |
| ENSTGUG00000004136 | VGLL4 | 0.035 | 0.002 | 24.0 | 10.0 | 0.36 |
| ENSTGUG00000008361 | MRPL46 | 0.035 | 0.002 | 24.0 | 14.0 | 0.35 |
| ENSTGUG00000002165 | GPATCH8 | 0.035 | 0.002 | 24.0 | 10.0 | 0.34 |
| ENSTGUG00000007900 | SCG2 | 0.035 | 0.002 | 24.0 | 8.0 | 0.33 |
| ENSTGUG00000006216 | MFAP3L | 0.035 | 0.002 | 24.0 | 6.0 | 0.33 |
| ENSTGUG00000012594 | ST3GAL1 | 0.035 | 0.002 | 26.0 | 2.0 | 0.32 |
| ENSTGUG00000015941 |  | 0.035 | 0.002 | 24.0 | 14.0 | 0.30 |
| ENSTGUG00000011993 | TUBE1 | 0.035 | 0.002 | 26.0 | 5.0 | 0.30 |
| ENSTGUG00000001296 | ST8SIA6 | 0.035 | 0.002 | 24.0 | 10.0 | 0.29 |
| ENSTGUG00000008921 | LRRC16A | 0.035 | 0.002 | 24.0 | 10.0 | 0.28 |
| ENSTGUG00000016253 | SLC43A3 | 0.035 | 0.002 | 26.0 | 15.0 | 0.27 |
| ENSTGUG00000001709 | ZMYM1 | 0.035 | 0.002 | 24.0 | 2.0 | 0.27 |
| ENSTGUG00000010797 | DTNA | 0.035 | 0.002 | 24.0 | 0.0 | 0.27 |
| ENSTGUG00000005091 | CLSTN2 | 0.035 | 0.002 | 24.0 | 5.0 | 0.27 |
| ENSTGUG00000000087 |  | 0.035 | 0.002 | 24.0 | 14.0 | 0.27 |
| ENSTGUG00000009373 | MBTD1 | 0.035 | 0.002 | 24.0 | 0.0 | 0.26 |
| ENSTGUG00000007093 | CCDC66 | 0.035 | 0.002 | 24.0 | 18.0 | 0.25 |
| ENSTGUG00000002029 | SPATA5 | 0.035 | 0.002 | 24.0 | 14.0 | 0.25 |
| ENSTGUG00000004780 | AMY1A | 0.035 | 0.002 | 24.0 | 2.0 | 0.25 |
| ENSTGUG00000008674 | LGI1 | 0.035 | 0.002 | 24.0 | 9.0 | 0.25 |
| ENSTGUG00000002071 | SLC25A34 | 0.035 | 0.002 | 24.0 | 18.0 | 0.25 |
| ENSTGUG00000008688 | IRS2 | 0.035 | 0.002 | 24.0 | 6.0 | 0.24 |
| ENSTGUG00000013478 | P2RY2 | 0.035 | 0.002 | 24.0 | 6.0 | 0.24 |
| ENSTGUG00000010532 | HORMAD2 | 0.035 | 0.002 | 25.3 | 16.7 | 0.24 |
| ENSTGUG00000007974 | TMEM27 | 0.035 | 0.002 | 24.0 | 8.0 | 0.23 |
| ENSTGUG00000006330 | MNS1 | 0.035 | 0.002 | 26.0 | 18.0 | 0.23 |
| ENSTGUG00000002812 | SLC30A10 | 0.035 | 0.002 | 24.0 | 2.0 | 0.23 |
| ENSTGUG00000000694 | GPR98 | 0.035 | 0.002 | 24.0 | 4.0 | 0.23 |
| ENSTGUG00000016814 |  | 0.035 | 0.002 | 24.0 | 6.0 | 0.22 |
| ENSTGUG00000003913 | KRT5 | 0.035 | 0.002 | 24.0 | 6.0 | 0.22 |
| ENSTGUG00000002114 | EGFLAM | 0.035 | 0.002 | 24.0 | 6.0 | 0.22 |
| ENSTGUG00000005679 | LHCGR | 0.035 | 0.002 | 24.0 | 18.0 | 0.22 |
| ENSTGUG00000011244 | ZC3H12C | 0.035 | 0.002 | 26.0 | 2.0 | 0.21 |
| ENSTGUG00000001894 | CCBE1 | 0.035 | 0.002 | 24.0 | 13.0 | 0.21 |
| ENSTGUG00000001403 |  | 0.035 | 0.002 | 24.0 | 8.0 | 0.21 |
| ENSTGUG00000011973 | LAMA4 | 0.035 | 0.002 | 26.0 | 2.0 | 0.21 |
| ENSTGUG00000017474 | PIK3C2B | 0.035 | 0.002 | 24.0 | 18.0 | 0.21 |
| ENSTGUG00000004560 | EPHA5 | 0.035 | 0.002 | 24.0 | 8.0 | 0.20 |
| ENSTGUG00000009124 | ZNF407 | 0.035 | 0.002 | 26.0 | 2.0 | 0.20 |
| ENSTGUG00000009252 | C3orf59 | 0.035 | 0.002 | 24.0 | 10.0 | 0.20 |
| ENSTGUG00000012818 | AMOTL1 | 0.035 | 0.002 | 24.0 | 2.0 | 0.20 |
| ENSTGUG00000015658 | ZNF493-15 | 0.035 | 0.002 | 25.3 | 16.7 | 0.19 |
| ENSTGUG00000011376 | FNDC1 | 0.035 | 0.002 | 24.0 | 8.0 | 0.18 |
| ENSTGUG00000002902 | HOXA3 | 0.035 | 0.002 | 24.0 | 16.0 | 0.18 |
| ENSTGUG00000012401 | MTBP | 0.035 | 0.002 | 24.0 | 12.0 | 0.18 |
| ENSTGUG00000014663 | HUWE1 | 0.035 | 0.002 | 26.0 | 15.0 | 0.17 |
| ENSTGUG00000001882 | NRF1 | 0.035 | 0.002 | 24.0 | 10.0 | 0.17 |
| ENSTGUG00000007664 | SEMA5A | 0.035 | 0.002 | 24.0 | 16.0 | 0.16 |
| ENSTGUG00000010240 | RPE65 | 0.035 | 0.002 | 24.0 | 10.0 | 0.16 |
| ENSTGUG00000017112 |  | 0.035 | 0.002 | 24.0 | 17.0 | 0.16 |
| ENSTGUG00000011551 | WASF3 | 0.035 | 0.002 | 24.0 | 4.0 | 0.15 |
| ENSTGUG00000000296 | SH3PXD2B | 0.035 | 0.002 | 24.0 | 8.0 | 0.15 |
| ENSTGUG00000004020 |  | 0.035 | 0.002 | 24.0 | 8.0 | 0.15 |
| ENSTGUG00000003312 |  | 0.035 | 0.002 | 24.0 | 16.0 | 0.15 |
| ENSTGUG00000006978 | FIGN | 0.035 | 0.002 | 24.0 | 4.0 | 0.15 |
| ENSTGUG00000009043 | TLR10 | 0.035 | 0.002 | 24.0 | 1.0 | 0.15 |
| ENSTGUG00000005108 | NTNG2 | 0.035 | 0.002 | 24.0 | 14.0 | 0.14 |
| ENSTGUG00000014350 |  | 0.035 | 0.002 | 24.0 | 17.0 | 0.14 |
| ENSTGUG00000007970 | FANCA | 0.035 | 0.002 | 24.0 | 16.0 | 0.13 |
| ENSTGUG00000007358 | CAPS2 | 0.035 | 0.002 | 25.3 | 4.0 | 0.13 |
| ENSTGUG00000011160 | TTBK2 | 0.035 | 0.002 | 24.0 | 20.0 | 0.12 |
| ENSTGUG00000005740 |  | 0.035 | 0.002 | 24.0 | 9.0 | 0.12 |
| ENSTGUG00000016681 | ATP13A2 | 0.035 | 0.002 | 24.0 | 6.0 | 0.12 |
| ENSTGUG00000013270 |  | 0.035 | 0.002 | 26.0 | 15.0 | 0.12 |
| ENSTGUG00000011992 | MATN2 | 0.035 | 0.002 | 24.0 | 6.0 | 0.11 |
| ENSTGUG00000013268 | PKHD1 | 0.035 | 0.002 | 24.0 | 14.0 | 0.10 |
| ENSTGUG00000010519 |  | 0.035 | 0.002 | 26.0 | 15.0 | 0.09 |
| ENSTGUG00000013039 | GDPD4 | 0.035 | 0.002 | 24.0 | 18.0 | 0.09 |
| ENSTGUG00000008910 | SLC13A5 | 0.035 | 0.002 | 24.0 | 20.0 | 0.09 |
| ENSTGUG00000015653 | MYO1A | 0.035 | 0.002 | 24.0 | 13.0 | 0.08 |
| ENSTGUG00000005196 | AVL9 | 0.035 | 0.002 | 24.0 | 9.0 | 0.06 |
| ENSTGUG00000003501 | IQGAP3 | 0.035 | 0.002 | 24.0 | 14.0 | 0.06 |
| ENSTGUG00000007441 |  | 0.035 | 0.002 | 24.0 | 6.0 | 0.04 |
| ENSTGUG00000012717 | LRP6 | 0.035 | 0.002 | 24.0 | 21.0 | 0.03 |
| ENSTGUG00000002049 | INTU | 0.035 | 0.002 | 24.0 | 4.0 | 0.03 |
| ENSTGUG00000013300 | CDCA3 | 0.077 | 0.004 | 28.0 | 16.0 | 22.66 |
| ENSTGUG00000008076 |  | 0.077 | 0.004 | 28.0 | 18.0 | 1.18 |
| ENSTGUG00000013511 | MAEL | 0.077 | 0.004 | 28.0 | 2.0 | 0.84 |
| ENSTGUG00000015351 |  | 0.077 | 0.004 | 28.0 | 16.0 | 0.77 |
| ENSTGUG00000007687 |  | 0.077 | 0.004 | 28.0 | 16.0 | 0.58 |
| ENSTGUG00000003474 | L3MBTL | 0.077 | 0.004 | 28.0 | 16.0 | 0.07 |
| ENSTGUG00000001572 | ALB | 0.130 | 0.009 | 24.0 | 16.0 | 10936.40 |
| ENSTGUG00000008243 | TMSB4X | 0.130 | 0.009 | 28.0 | 15.0 | 514.14 |
| ENSTGUG00000017654 |  | 0.130 | 0.009 | 28.0 | 15.0 | 430.87 |
| ENSTGUG00000007111 | ULK2 | 0.130 | 0.009 | 28.0 | 1.0 | 157.43 |
| ENSTGUG00000010581 | LGALS2 | 0.130 | 0.009 | 28.0 | 15.0 | 144.58 |
| ENSTGUG00000009404 | PI4K2B | 0.130 | 0.009 | 28.0 | 1.0 | 96.24 |
| ENSTGUG00000007670 | ACTG2 | 0.130 | 0.009 | 28.0 | 15.0 | 79.41 |
| ENSTGUG00000013147 | FKBP4 | 0.130 | 0.009 | 28.0 | 1.0 | 68.36 |
| ENSTGUG00000009891 | SLC2A9 | 0.130 | 0.009 | 28.0 | 1.0 | 48.60 |
| ENSTGUG00000014649 |  | 0.130 | 0.009 | 28.0 | 15.0 | 48.58 |
| ENSTGUG00000010650 | RAC2 | 0.130 | 0.009 | 28.0 | 15.0 | 47.31 |
| ENSTGUG00000009210 | ARHGDIB | 0.130 | 0.009 | 28.0 | 15.0 | 43.40 |
| ENSTGUG00000014456 | POLR2G | 0.130 | 0.009 | 28.0 | 15.0 | 43.16 |
| ENSTGUG00000001291 | SKP1A | 0.130 | 0.009 | 28.0 | 1.0 | 42.94 |
| ENSTGUG00000006944 | OGT | 0.130 | 0.009 | 28.0 | 1.0 | 39.60 |
| ENSTGUG00000002550 | SH3BGRL | 0.130 | 0.009 | 28.0 | 1.0 | 30.58 |
| ENSTGUG00000018010 |  | 0.130 | 0.009 | 24.0 | 7.0 | 25.16 |
| ENSTGUG00000008589 | SLC30A9 | 0.130 | 0.009 | 28.0 | 1.0 | 23.45 |
| ENSTGUG00000009527 | B5FXK3_TAEGU | 0.130 | 0.009 | 28.0 | 15.0 | 22.84 |
| ENSTGUG00000002851 |  | 0.130 | 0.009 | 28.0 | 15.0 | 21.32 |
| ENSTGUG00000001529 | MCCC2 | 0.130 | 0.009 | 28.0 | 3.0 | 17.78 |
| ENSTGUG00000013469 | MRPL48 | 0.130 | 0.009 | 28.0 | 15.0 | 14.91 |
| ENSTGUG00000003085 | BAZ1B | 0.130 | 0.009 | 28.0 | 1.0 | 14.52 |
| ENSTGUG00000010081 | EGLN1 | 0.130 | 0.009 | 28.0 | 1.0 | 13.05 |
| ENSTGUG00000007498 |  | 0.130 | 0.009 | 28.0 | 15.0 | 12.07 |
| ENSTGUG00000011712 | HSPH1 | 0.130 | 0.009 | 28.0 | 1.0 | 11.59 |
| ENSTGUG00000018670 |  | 0.130 | 0.009 | 24.0 | 0.0 | 11.21 |
| ENSTGUG00000008735 | HN1 | 0.130 | 0.009 | 28.0 | 15.0 | 11.01 |
| ENSTGUG00000012877 | CHORDC1 | 0.130 | 0.009 | 28.0 | 1.0 | 10.85 |
| ENSTGUG00000007494 | CSRP2 | 0.130 | 0.009 | 28.0 | 15.0 | 10.73 |
| ENSTGUG00000000929 | CNN2 | 0.130 | 0.009 | 28.0 | 15.0 | 9.30 |
| ENSTGUG00000002282 | BEND7 | 0.130 | 0.009 | 28.0 | 1.0 | 8.96 |
| ENSTGUG00000013616 | FAM55C | 0.130 | 0.009 | 28.0 | 1.0 | 8.03 |
| ENSTGUG00000011060 | DYNLT1 | 0.130 | 0.009 | 28.0 | 15.0 | 7.90 |
| ENSTGUG00000006304 | TAOK1 | 0.130 | 0.009 | 28.0 | 1.0 | 7.63 |
| ENSTGUG00000011675 | INPP5A | 0.130 | 0.009 | 28.0 | 1.0 | 7.30 |
| ENSTGUG00000016800 |  | 0.130 | 0.009 | 28.0 | 1.0 | 7.10 |
| ENSTGUG00000015013 | HDAC3 | 0.130 | 0.009 | 28.0 | 15.0 | 6.94 |
| ENSTGUG00000005824 | CD40 | 0.130 | 0.009 | 28.0 | 15.0 | 6.32 |
| ENSTGUG00000005589 |  | 0.130 | 0.009 | 28.0 | 1.0 | 6.00 |
| ENSTGUG00000013344 | KPNA1 | 0.130 | 0.009 | 28.0 | 1.0 | 5.94 |
| ENSTGUG00000004554 | TTC39B | 0.130 | 0.009 | 28.0 | 1.0 | 5.28 |
| ENSTGUG00000002811 | MPP6 | 0.130 | 0.009 | 28.0 | 1.0 | 5.24 |
| ENSTGUG00000003414 | GNAQ | 0.130 | 0.009 | 28.0 | 1.0 | 5.03 |
| ENSTGUG00000013438 | TTF2 | 0.130 | 0.009 | 28.0 | 15.0 | 4.99 |
| ENSTGUG00000002027 | SKP2 | 0.130 | 0.009 | 28.0 | 1.0 | 4.85 |
| ENSTGUG00000016943 |  | 0.130 | 0.009 | 24.0 | 9.0 | 4.67 |
| ENSTGUG00000011183 | C10orf119 | 0.130 | 0.009 | 28.0 | 15.0 | 4.64 |
| ENSTGUG00000003519 | NGLY1 | 0.130 | 0.009 | 28.0 | 1.0 | 4.56 |
| ENSTGUG00000004477 | ALKBH4 | 0.130 | 0.009 | 28.0 | 15.0 | 4.36 |
| ENSTGUG00000016577 | SSU72 | 0.130 | 0.009 | 28.0 | 15.0 | 4.33 |
| ENSTGUG00000015968 | GOLGB1 | 0.130 | 0.009 | 28.0 | 1.0 | 4.16 |
| ENSTGUG00000004438 | ZNF281 | 0.130 | 0.009 | 28.0 | 1.0 | 3.92 |
| ENSTGUG00000017538 | ST7L | 0.130 | 0.009 | 28.0 | 15.0 | 3.85 |
| ENSTGUG00000009606 | PI4K2A | 0.130 | 0.009 | 28.0 | 15.0 | 3.79 |
| ENSTGUG00000018635 |  | 0.130 | 0.009 | 24.0 | 7.0 | 3.77 |
| ENSTGUG00000009515 | ZNF828 | 0.130 | 0.009 | 28.0 | 1.0 | 3.65 |
| ENSTGUG00000000773 | RPUSD4 | 0.130 | 0.009 | 28.0 | 15.0 | 3.53 |
| ENSTGUG00000014820 |  | 0.130 | 0.009 | 28.0 | 1.0 | 3.49 |
| ENSTGUG00000014133 |  | 0.130 | 0.009 | 28.0 | 15.0 | 3.48 |
| ENSTGUG00000003856 | ZBED4 | 0.130 | 0.009 | 28.0 | 1.0 | 3.43 |
| ENSTGUG00000004855 | VPRBP | 0.130 | 0.009 | 28.0 | 1.0 | 3.27 |
| ENSTGUG00000007252 |  | 0.130 | 0.009 | 28.0 | 1.0 | 3.25 |
| ENSTGUG00000012220 | BATF | 0.130 | 0.009 | 28.0 | 15.0 | 3.23 |
| ENSTGUG00000011186 | MTRF1L | 0.130 | 0.009 | 28.0 | 17.0 | 3.18 |
| ENSTGUG00000013694 | PACSIN1 | 0.130 | 0.009 | 28.0 | 15.0 | 3.12 |
| ENSTGUG00000011448 | GALNTL1 | 0.130 | 0.009 | 28.0 | 1.0 | 2.91 |
| ENSTGUG00000013339 | NCAPD2 | 0.130 | 0.009 | 28.0 | 15.0 | 2.77 |
| ENSTGUG00000009308 | TMEM48 | 0.130 | 0.009 | 28.0 | 15.0 | 2.69 |
| ENSTGUG00000007490 | NCKIPSD | 0.130 | 0.009 | 28.0 | 1.0 | 2.63 |
| ENSTGUG00000009808 | PDE6D | 0.130 | 0.009 | 28.0 | 15.0 | 2.38 |
| ENSTGUG00000004954 | QSER1 | 0.130 | 0.009 | 28.0 | 1.0 | 2.37 |
| ENSTGUG00000011705 | SLCO1A2 | 0.130 | 0.009 | 28.0 | 1.0 | 2.35 |
| ENSTGUG00000007335 | NEU2 | 0.130 | 0.009 | 28.0 | 15.0 | 2.27 |
| ENSTGUG00000013320 | UNQ9391 | 0.130 | 0.009 | 24.0 | 6.0 | 2.25 |
| ENSTGUG00000004090 | RC3H1 | 0.130 | 0.009 | 28.0 | 1.0 | 2.23 |
| ENSTGUG00000011946 | NT5DC1 | 0.130 | 0.009 | 28.0 | 17.0 | 2.22 |
| ENSTGUG00000001431 | TMEM39B | 0.130 | 0.009 | 28.0 | 15.0 | 2.15 |
| ENSTGUG00000007327 | SGCZ | 0.130 | 0.009 | 24.0 | 7.0 | 1.96 |
| ENSTGUG00000005516 | OGFOD2 | 0.130 | 0.009 | 28.0 | 15.0 | 1.85 |
| ENSTGUG00000011519 | VTI1B | 0.130 | 0.009 | 28.0 | 15.0 | 1.85 |
| ENSTGUG00000009512 | SIRT4 | 0.130 | 0.009 | 28.0 | 17.0 | 1.72 |
| ENSTGUG00000000123 | MATK | 0.130 | 0.009 | 28.0 | 15.0 | 1.64 |
| ENSTGUG00000002516 | DHX58 | 0.130 | 0.009 | 28.0 | 15.0 | 1.54 |
| ENSTGUG00000002383 | PHF14 | 0.130 | 0.009 | 28.0 | 15.0 | 1.51 |
| ENSTGUG00000012499 | MICAL3 | 0.130 | 0.009 | 28.0 | 1.0 | 1.49 |
| ENSTGUG00000002003 | FBXO42 | 0.130 | 0.009 | 28.0 | 15.0 | 1.45 |
| ENSTGUG00000005062 | BRWD1 | 0.130 | 0.009 | 28.0 | 1.0 | 1.41 |
| ENSTGUG00000011374 | C3orf44 | 0.130 | 0.009 | 28.0 | 15.0 | 1.32 |
| ENSTGUG00000016092 |  | 0.130 | 0.009 | 24.0 | 9.0 | 1.28 |
| ENSTGUG00000013221 | CASP2 | 0.130 | 0.009 | 28.0 | 1.0 | 1.22 |
| ENSTGUG00000008886 | DGCR14 | 0.130 | 0.009 | 28.0 | 15.0 | 1.19 |
| ENSTGUG00000006875 | CPM | 0.130 | 0.009 | 28.0 | 15.0 | 1.13 |
| ENSTGUG00000002712 | RSBN1L | 0.130 | 0.009 | 28.0 | 3.0 | 1.11 |
| ENSTGUG00000002309 | RREB1 | 0.130 | 0.009 | 28.0 | 1.0 | 1.01 |
| ENSTGUG00000003893 | NCF2 | 0.130 | 0.009 | 28.0 | 15.0 | 1.01 |
| ENSTGUG00000011015 | EVI1 | 0.130 | 0.009 | 28.0 | 1.0 | 0.80 |
| ENSTGUG00000016654 | C1orf2 | 0.130 | 0.009 | 24.0 | 13.0 | 0.79 |
| ENSTGUG00000010601 | MFNG | 0.130 | 0.009 | 28.0 | 1.0 | 0.77 |
| ENSTGUG00000017478 |  | 0.130 | 0.009 | 28.0 | 15.0 | 0.75 |
| ENSTGUG00000000288 | UBTD2 | 0.130 | 0.009 | 28.0 | 15.0 | 0.73 |
| ENSTGUG00000007258 |  | 0.130 | 0.009 | 28.0 | 15.0 | 0.72 |
| ENSTGUG00000013894 |  | 0.130 | 0.009 | 24.0 | 0.0 | 0.72 |
| ENSTGUG00000005779 | FIBIN | 0.130 | 0.009 | 24.0 | 9.0 | 0.67 |
| ENSTGUG00000017414 | GON4L | 0.130 | 0.009 | 28.0 | 1.0 | 0.64 |
| ENSTGUG00000009118 | ZNF407 | 0.130 | 0.009 | 28.0 | 1.0 | 0.63 |
| ENSTGUG00000001206 | PTPN7 | 0.130 | 0.009 | 28.0 | 15.0 | 0.59 |
| ENSTGUG00000007367 | NT5M | 0.130 | 0.009 | 28.0 | 15.0 | 0.56 |
| ENSTGUG00000001314 | TOPORS | 0.130 | 0.009 | 28.0 | 1.0 | 0.52 |
| ENSTGUG00000010286 | GNG4 | 0.130 | 0.009 | 28.0 | 17.0 | 0.50 |
| ENSTGUG00000007263 | FAM57A | 0.130 | 0.009 | 24.0 | 9.0 | 0.50 |
| ENSTGUG00000014946 |  | 0.130 | 0.009 | 28.0 | 1.0 | 0.49 |
| ENSTGUG00000016014 |  | 0.130 | 0.009 | 24.0 | 3.0 | 0.48 |
| ENSTGUG00000010484 | MCF2L2 | 0.130 | 0.009 | 28.0 | 1.0 | 0.46 |
| ENSTGUG00000002061 | GJC1 | 0.130 | 0.009 | 28.0 | 15.0 | 0.45 |
| ENSTGUG00000013921 |  | 0.130 | 0.009 | 24.0 | 16.0 | 0.45 |
| ENSTGUG00000015224 |  | 0.130 | 0.009 | 24.0 | 9.0 | 0.43 |
| ENSTGUG00000007077 |  | 0.130 | 0.009 | 24.0 | 6.0 | 0.43 |
| ENSTGUG00000000209 | IL12B | 0.130 | 0.009 | 24.0 | 1.0 | 0.43 |
| ENSTGUG00000000065 | MLL | 0.130 | 0.009 | 28.0 | 1.0 | 0.42 |
| ENSTGUG00000002578 | AGR2 | 0.130 | 0.009 | 24.0 | 10.0 | 0.38 |
| ENSTGUG00000010962 | PRDM4 | 0.130 | 0.009 | 28.0 | 1.0 | 0.38 |
| ENSTGUG00000013973 |  | 0.130 | 0.009 | 24.0 | 16.0 | 0.37 |
| ENSTGUG00000010733 | ASMT | 0.130 | 0.009 | 24.0 | 5.0 | 0.35 |
| ENSTGUG00000014069 |  | 0.130 | 0.009 | 24.0 | 0.0 | 0.33 |
| ENSTGUG00000007688 | HPS4 | 0.130 | 0.009 | 24.0 | 15.0 | 0.33 |
| ENSTGUG00000014087 |  | 0.130 | 0.009 | 24.0 | 4.0 | 0.32 |
| ENSTGUG00000006810 | LRP2BP | 0.130 | 0.009 | 28.0 | 15.0 | 0.30 |
| ENSTGUG00000008346 | GPER | 0.130 | 0.009 | 24.0 | 9.0 | 0.28 |
| ENSTGUG00000014352 |  | 0.130 | 0.009 | 24.0 | 4.0 | 0.26 |
| ENSTGUG00000006767 | GNB5 | 0.130 | 0.009 | 24.0 | 2.0 | 0.23 |
| ENSTGUG00000012051 | POLR2K | 0.130 | 0.009 | 24.0 | 10.0 | 0.22 |
| ENSTGUG00000014391 | ZFYVE26-2 | 0.130 | 0.009 | 24.0 | 0.0 | 0.22 |
| ENSTGUG00000013681 | HSPA12B | 0.130 | 0.009 | 24.0 | 3.0 | 0.22 |
| ENSTGUG00000011238 | GPC5 | 0.130 | 0.009 | 24.0 | 8.0 | 0.22 |
| ENSTGUG00000009867 | IL1RL1 | 0.130 | 0.009 | 28.0 | 3.0 | 0.21 |
| ENSTGUG00000006217 | ANKDD1B | 0.130 | 0.009 | 24.0 | 5.0 | 0.21 |
| ENSTGUG00000013969 |  | 0.130 | 0.009 | 24.0 | 10.0 | 0.20 |
| ENSTGUG00000015923 | MLL2 | 0.130 | 0.009 | 24.0 | 9.0 | 0.20 |
| ENSTGUG00000018387 |  | 0.130 | 0.009 | 24.0 | 10.0 | 0.20 |
| ENSTGUG00000003844 | LHFPL2 | 0.130 | 0.009 | 24.0 | 9.0 | 0.19 |
| ENSTGUG00000003196 | TNFSF8 | 0.130 | 0.009 | 24.0 | 8.0 | 0.19 |
| ENSTGUG00000017062 | UBXN10 | 0.130 | 0.009 | 24.0 | 8.0 | 0.18 |
| ENSTGUG00000008520 | LHFPL4 | 0.130 | 0.009 | 24.0 | 10.0 | 0.18 |
| ENSTGUG00000003217 | ACAP3 | 0.130 | 0.009 | 24.0 | 9.0 | 0.17 |
| ENSTGUG00000000282 | SEC22C | 0.130 | 0.009 | 24.0 | 0.0 | 0.16 |
| ENSTGUG00000003430 | HOXB5 | 0.130 | 0.009 | 24.0 | 8.0 | 0.15 |
| ENSTGUG00000015912 |  | 0.130 | 0.009 | 24.0 | 9.0 | 0.15 |
| ENSTGUG00000015698 |  | 0.130 | 0.009 | 24.0 | 18.0 | 0.15 |
| ENSTGUG00000000017 |  | 0.130 | 0.009 | 24.0 | 0.0 | 0.14 |
| ENSTGUG00000014629 |  | 0.130 | 0.009 | 24.0 | 4.0 | 0.14 |
| ENSTGUG00000018278 |  | 0.130 | 0.009 | 24.0 | 20.0 | 0.14 |
| ENSTGUG00000015983 |  | 0.130 | 0.009 | 24.0 | 8.0 | 0.14 |
| ENSTGUG00000007534 | DHRS7C | 0.130 | 0.009 | 24.0 | 18.0 | 0.13 |
| ENSTGUG00000014734 |  | 0.130 | 0.009 | 24.0 | 0.0 | 0.13 |
| ENSTGUG00000009861 | IL18R1 | 0.130 | 0.009 | 28.0 | 15.0 | 0.13 |
| ENSTGUG00000016374 |  | 0.130 | 0.009 | 24.0 | 9.0 | 0.12 |
| ENSTGUG00000005931 | RDH8 | 0.130 | 0.009 | 24.0 | 10.0 | 0.12 |
| ENSTGUG00000017442 | AIRE | 0.130 | 0.009 | 24.0 | 18.0 | 0.12 |
| ENSTGUG00000012140 |  | 0.130 | 0.009 | 24.0 | 10.0 | 0.12 |
| ENSTGUG00000018244 |  | 0.130 | 0.009 | 24.0 | 3.0 | 0.11 |
| ENSTGUG00000018232 |  | 0.130 | 0.009 | 24.0 | 9.0 | 0.11 |
| ENSTGUG00000009591 | SPIRE1 | 0.130 | 0.009 | 24.0 | 3.0 | 0.11 |
| ENSTGUG00000017172 | TMEM121 | 0.130 | 0.009 | 24.0 | 10.0 | 0.10 |
| ENSTGUG00000004191 | CAMKK1 | 0.130 | 0.009 | 24.0 | 2.0 | 0.10 |
| ENSTGUG00000015602 | RASAL1 | 0.130 | 0.009 | 24.0 | 16.0 | 0.09 |
| ENSTGUG00000005392 | ACCN5 | 0.130 | 0.009 | 24.0 | 2.0 | 0.09 |
| ENSTGUG00000009081 | RASL12 | 0.130 | 0.009 | 28.0 | 15.0 | 0.09 |
| ENSTGUG00000010143 | PCNXL2 | 0.130 | 0.009 | 28.0 | 17.0 | 0.08 |
| ENSTGUG00000016862 | ACVR1C | 0.130 | 0.009 | 24.0 | 6.0 | 0.08 |
| ENSTGUG00000001123 | MASTL | 0.130 | 0.009 | 24.0 | 8.0 | 0.08 |
| ENSTGUG00000002455 | POF1B | 0.130 | 0.009 | 24.0 | 4.0 | 0.08 |
| ENSTGUG00000008270 | WDR69 | 0.130 | 0.009 | 24.0 | 10.0 | 0.07 |
| ENSTGUG00000011049 | STK24 | 0.130 | 0.009 | 24.0 | 9.0 | 0.07 |
| ENSTGUG00000005439 | PDGFC | 0.130 | 0.009 | 24.0 | 6.0 | 0.07 |
| ENSTGUG00000005475 |  | 0.130 | 0.009 | 24.0 | 8.0 | 0.07 |
| ENSTGUG00000006722 | SYT12 | 0.130 | 0.009 | 24.0 | 18.0 | 0.06 |
| ENSTGUG00000007987 | KLHDC6 | 0.130 | 0.009 | 24.0 | 9.0 | 0.06 |
| ENSTGUG00000006964 | SMPD3 | 0.130 | 0.009 | 24.0 | 10.0 | 0.05 |
| ENSTGUG00000017314 | SELE | 0.130 | 0.009 | 24.0 | 0.0 | 0.05 |
| ENSTGUG00000004225 | SLC6A11 | 0.130 | 0.009 | 24.0 | 18.0 | 0.05 |
| ENSTGUG00000018391 |  | 0.130 | 0.009 | 24.0 | 6.0 | 0.04 |
| ENSTGUG00000013485 | ADAMTS5 | 0.130 | 0.009 | 24.0 | 10.0 | 0.03 |
| ENSTGUG00000004321 | SLC24A2 | 0.130 | 0.009 | 24.0 | 6.0 | 0.03 |
| ENSTGUG00000008527 | GABBR2 | 0.130 | 0.009 | 24.0 | 6.0 | 0.03 |
| ENSTGUG00000000464 |  | 0.130 | 0.009 | 24.0 | 10.0 | 0.03 |
| ENSTGUG00000016154 |  | 0.130 | 0.009 | 24.0 | 4.0 | 0.00 |
| ENSTGUG00000002294 |  | 0.234 | 0.018 | 28.0 | 2.0 | 3.69 |
| ENSTGUG00000015137 |  | 0.234 | 0.018 | 28.0 | 2.0 | 0.48 |
| ENSTGUG00000006658 | ADK | 0.234 | 0.032 | 24.0 | 5.0 | 689.20 |
| ENSTGUG00000003313 | PGRMC1 | 0.234 | 0.032 | 24.0 | 5.0 | 526.65 |
| ENSTGUG00000004381 | HBM | 0.234 | 0.032 | 25.3 | 12.7 | 464.38 |
| ENSTGUG00000018223 |  | 0.234 | 0.032 | 25.3 | 9.3 | 362.23 |
| ENSTGUG00000012808 |  | 0.234 | 0.032 | 26.0 | 2.0 | 359.97 |
| ENSTGUG00000003666 | AHCY | 0.234 | 0.032 | 24.0 | 6.0 | 345.47 |
| ENSTGUG00000013056 | PDIA6 | 0.234 | 0.032 | 25.3 | 8.0 | 280.38 |
| ENSTGUG00000002260 | HSD17B11 | 0.234 | 0.032 | 24.0 | 5.0 | 215.54 |
| ENSTGUG00000011393 |  | 0.234 | 0.032 | 24.0 | 21.0 | 210.34 |
| ENSTGUG00000014209 |  | 0.234 | 0.032 | 24.0 | 5.0 | 175.51 |
| ENSTGUG00000003171 |  | 0.234 | 0.032 | 24.0 | 20.0 | 160.72 |
| ENSTGUG00000003404 | C16orf14 | 0.234 | 0.032 | 26.0 | 15.0 | 155.81 |
| ENSTGUG00000012859 | C11orf75 | 0.234 | 0.032 | 24.0 | 5.0 | 155.04 |
| ENSTGUG00000010812 | CHP | 0.234 | 0.032 | 26.0 | 2.0 | 143.01 |
| ENSTGUG00000005930 | SC4MOL | 0.234 | 0.032 | 24.0 | 0.0 | 135.96 |
| ENSTGUG00000006623 | RHOA | 0.234 | 0.032 | 24.0 | 5.0 | 127.26 |
| ENSTGUG00000018598 |  | 0.234 | 0.032 | 24.0 | 18.0 | 125.19 |
| ENSTGUG00000000039 | HYOU1 | 0.234 | 0.032 | 24.0 | 6.0 | 115.71 |
| ENSTGUG00000000797 | STT3A | 0.234 | 0.032 | 25.3 | 12.7 | 106.02 |
| ENSTGUG00000012949 | ACP1 | 0.234 | 0.032 | 24.0 | 2.0 | 91.29 |
| ENSTGUG00000008362 | PANK1 | 0.234 | 0.032 | 25.3 | 9.3 | 84.28 |
| ENSTGUG00000004026 |  | 0.234 | 0.032 | 25.3 | 12.7 | 83.47 |
| ENSTGUG00000004943 | TSC22D3 | 0.234 | 0.032 | 24.0 | 8.0 | 81.42 |
| ENSTGUG00000002193 | C6orf86 | 0.234 | 0.032 | 24.0 | 21.0 | 74.01 |
| ENSTGUG00000007742 | ABCG2 | 0.234 | 0.032 | 25.3 | 9.3 | 71.96 |
| ENSTGUG00000016435 |  | 0.234 | 0.032 | 24.0 | 5.0 | 68.69 |
| ENSTGUG00000011405 | PFN2 | 0.234 | 0.032 | 25.3 | 9.3 | 67.06 |
| ENSTGUG00000006219 | AADAT | 0.234 | 0.032 | 24.0 | 4.0 | 65.44 |
| ENSTGUG00000016881 | TMEM214 | 0.234 | 0.032 | 24.0 | 13.0 | 64.26 |
| ENSTGUG00000003609 | HNRNPH3 | 0.234 | 0.032 | 24.0 | 20.0 | 63.09 |
| ENSTGUG00000017421 | S100A13 | 0.234 | 0.032 | 24.0 | 21.0 | 59.04 |
| ENSTGUG00000013536 | GBE1 | 0.234 | 0.032 | 24.0 | 6.0 | 58.28 |
| ENSTGUG00000015030 |  | 0.234 | 0.032 | 24.0 | 8.0 | 57.15 |
| ENSTGUG00000016471 |  | 0.234 | 0.032 | 25.3 | 8.0 | 56.91 |
| ENSTGUG00000014406 |  | 0.234 | 0.032 | 26.0 | 15.0 | 55.27 |
| ENSTGUG00000003455 | SFRS6 | 0.234 | 0.032 | 24.0 | 12.0 | 54.39 |
| ENSTGUG00000011872 | KYNU | 0.234 | 0.032 | 24.0 | 21.0 | 50.32 |
| ENSTGUG00000010398 | BZW1 | 0.234 | 0.032 | 25.3 | 9.3 | 48.62 |
| ENSTGUG00000008678 | SDF2L1 | 0.234 | 0.032 | 25.3 | 8.0 | 47.87 |
| ENSTGUG00000006156 | C10orf57-1 | 0.234 | 0.032 | 24.0 | 2.0 | 45.56 |
| ENSTGUG00000005781 | FADS1-3 | 0.234 | 0.032 | 25.3 | 16.7 | 44.11 |
| ENSTGUG00000002370 | MUTED-2 | 0.234 | 0.032 | 26.0 | 2.0 | 43.65 |
| ENSTGUG00000007864 | PSMA7 | 0.234 | 0.032 | 26.0 | 15.0 | 42.78 |
| ENSTGUG00000005683 | AARS | 0.234 | 0.032 | 25.3 | 12.7 | 42.62 |
| ENSTGUG00000009238 | TOB1 | 0.234 | 0.032 | 25.3 | 9.3 | 42.30 |
| ENSTGUG00000010935 | TM9SF2 | 0.234 | 0.032 | 24.0 | 1.0 | 41.28 |
| ENSTGUG00000013259 | RBP5 | 0.234 | 0.032 | 24.0 | 14.0 | 40.96 |
| ENSTGUG00000006567 | C5 | 0.234 | 0.032 | 24.0 | 6.0 | 39.61 |
| ENSTGUG00000010842 | HMOX1 | 0.234 | 0.032 | 25.3 | 16.7 | 39.50 |
| ENSTGUG00000004057 |  | 0.234 | 0.032 | 24.0 | 14.0 | 38.68 |
| ENSTGUG00000012904 | AKT1 | 0.234 | 0.032 | 26.0 | 2.0 | 37.85 |
| ENSTGUG00000012669 | CLMN-2 | 0.234 | 0.032 | 24.0 | 5.0 | 37.40 |
| ENSTGUG00000003504 | SLC25A25 | 0.234 | 0.032 | 26.0 | 2.0 | 36.88 |
| ENSTGUG00000008422 | RRAS2 | 0.234 | 0.032 | 26.0 | 2.0 | 36.68 |
| ENSTGUG00000004809 | AIF1L | 0.234 | 0.032 | 24.0 | 8.0 | 34.88 |
| ENSTGUG00000009104 | SLC25A20 | 0.234 | 0.032 | 24.0 | 8.0 | 34.08 |
| ENSTGUG00000009879 | FAM152B | 0.234 | 0.032 | 24.0 | 0.0 | 33.76 |
| ENSTGUG00000006236 | CCBL2 | 0.234 | 0.032 | 24.0 | 1.0 | 33.75 |
| ENSTGUG00000000002 | DPAGT1 | 0.234 | 0.032 | 24.0 | 18.0 | 33.39 |
| ENSTGUG00000006789 | MAPK6 | 0.234 | 0.032 | 24.0 | 2.0 | 32.55 |
| ENSTGUG00000008718 | PDCD6 | 0.234 | 0.032 | 24.0 | 21.0 | 31.34 |
| ENSTGUG00000011700 | HMGB1 | 0.234 | 0.032 | 24.0 | 18.0 | 29.82 |
| ENSTGUG00000010810 | SF3B5 | 0.234 | 0.032 | 25.3 | 12.7 | 29.60 |
| ENSTGUG00000001114 | YME1L1 | 0.234 | 0.032 | 24.0 | 18.0 | 27.72 |
| ENSTGUG00000014696 | TMEM98 | 0.234 | 0.032 | 24.0 | 14.0 | 27.59 |
| ENSTGUG00000001747 | OPRS1 | 0.234 | 0.032 | 25.3 | 12.7 | 27.50 |
| ENSTGUG00000004399 | ADFP | 0.234 | 0.032 | 24.0 | 8.0 | 27.37 |
| ENSTGUG00000009019 | C3orf60 | 0.234 | 0.032 | 25.3 | 12.7 | 27.05 |
| ENSTGUG00000018414 |  | 0.234 | 0.032 | 24.0 | 4.0 | 26.57 |
| ENSTGUG00000009098 |  | 0.234 | 0.032 | 25.3 | 9.3 | 25.83 |
| ENSTGUG00000009413 | BCL6 | 0.234 | 0.032 | 28.0 | 2.0 | 25.60 |
| ENSTGUG00000012677 | GLRX5 | 0.234 | 0.032 | 24.0 | 6.0 | 25.15 |
| ENSTGUG00000009179 | PDHB | 0.234 | 0.032 | 24.0 | 1.0 | 24.48 |
| ENSTGUG00000000417 | NFIL3 | 0.234 | 0.032 | 24.0 | 9.0 | 24.18 |
| ENSTGUG00000000928 | SFRS3 | 0.234 | 0.032 | 25.3 | 16.7 | 23.35 |
| ENSTGUG00000014516 | RAB10 | 0.234 | 0.032 | 24.0 | 18.0 | 22.96 |
| ENSTGUG00000011669 | MKRN1 | 0.234 | 0.032 | 24.0 | 5.0 | 22.48 |
| ENSTGUG00000011788 | TTC38 | 0.234 | 0.032 | 24.0 | 5.0 | 22.45 |
| ENSTGUG00000007372 | C8orf79 | 0.234 | 0.032 | 24.0 | 5.0 | 21.13 |
| ENSTGUG00000005456 | ST6GAL1 | 0.234 | 0.032 | 24.0 | 5.0 | 20.78 |
| ENSTGUG00000012692 | MRPS35 | 0.234 | 0.032 | 26.0 | 15.0 | 20.66 |
| ENSTGUG00000005508 | GLOD5 | 0.234 | 0.032 | 24.0 | 13.0 | 20.47 |
| ENSTGUG00000001393 | C1orf91 | 0.234 | 0.032 | 26.0 | 15.0 | 20.31 |
| ENSTGUG00000000548 | KIAA1191 | 0.234 | 0.032 | 24.0 | 2.0 | 20.04 |
| ENSTGUG00000011541 | MTPN | 0.234 | 0.032 | 26.0 | 2.0 | 19.05 |
| ENSTGUG00000003513 | THOC4 | 0.234 | 0.032 | 26.0 | 15.0 | 18.95 |
| ENSTGUG00000008996 | HNRNPA3 | 0.234 | 0.032 | 24.0 | 22.0 | 18.37 |
| ENSTGUG00000008925 | MRPL27 | 0.234 | 0.032 | 24.0 | 21.0 | 18.13 |
| ENSTGUG00000007096 | ARFRP1 | 0.234 | 0.032 | 26.0 | 15.0 | 18.06 |
| ENSTGUG00000008868 |  | 0.234 | 0.032 | 26.0 | 15.0 | 18.01 |
| ENSTGUG00000004012 | CLK3 | 0.234 | 0.032 | 25.3 | 12.7 | 17.96 |
| ENSTGUG00000004463 | TOR1A | 0.234 | 0.032 | 26.0 | 2.0 | 17.94 |
| ENSTGUG00000001331 | C10orf97 | 0.234 | 0.032 | 25.3 | 9.3 | 17.89 |
| ENSTGUG00000006523 | RASSF3 | 0.234 | 0.032 | 25.3 | 4.0 | 17.87 |
| ENSTGUG00000008435 | COPB1 | 0.234 | 0.032 | 26.0 | 15.0 | 17.76 |
| ENSTGUG00000011782 | HINT3 | 0.234 | 0.032 | 25.3 | 9.3 | 17.54 |
| ENSTGUG00000004133 | EPHX1 | 0.234 | 0.032 | 24.0 | 5.0 | 17.34 |
| ENSTGUG00000011021 | LYPLA1 | 0.234 | 0.032 | 24.0 | 5.0 | 16.70 |
| ENSTGUG00000012113 | SESN1 | 0.234 | 0.032 | 24.0 | 2.0 | 16.69 |
| ENSTGUG00000005161 | TFAM | 0.234 | 0.032 | 24.0 | 2.0 | 16.37 |
| ENSTGUG00000006829 | UFSP2 | 0.234 | 0.032 | 24.0 | 21.0 | 16.37 |
| ENSTGUG00000003901 | EIF4H | 0.234 | 0.032 | 25.3 | 9.3 | 15.92 |
| ENSTGUG00000013859 |  | 0.234 | 0.032 | 25.3 | 9.3 | 15.84 |
| ENSTGUG00000008727 | SUMO4 | 0.234 | 0.032 | 24.0 | 20.0 | 15.68 |
| ENSTGUG00000009568 | COQ5 | 0.234 | 0.032 | 26.0 | 15.0 | 15.53 |
| ENSTGUG00000001466 | SF3A3 | 0.234 | 0.032 | 26.0 | 15.0 | 15.42 |
| ENSTGUG00000002932 | TAX1BP1 | 0.234 | 0.032 | 25.3 | 9.3 | 15.33 |
| ENSTGUG00000009719 | TAPT1 | 0.234 | 0.032 | 24.0 | 2.0 | 15.22 |
| ENSTGUG00000008259 | KIAA0174 | 0.234 | 0.032 | 25.3 | 12.7 | 15.09 |
| ENSTGUG00000003201 |  | 0.234 | 0.032 | 26.0 | 15.0 | 15.05 |
| ENSTGUG00000001105 | RAB18 | 0.234 | 0.032 | 24.0 | 4.0 | 15.01 |
| ENSTGUG00000011894 | RFXAP | 0.234 | 0.032 | 24.0 | 18.0 | 14.79 |
| ENSTGUG00000001213 | PPT1 | 0.234 | 0.032 | 26.0 | 15.0 | 14.65 |
| ENSTGUG00000010086 | WDR12 | 0.234 | 0.032 | 26.0 | 15.0 | 14.49 |
| ENSTGUG00000000388 | IFI30 | 0.234 | 0.032 | 25.3 | 12.7 | 14.29 |
| ENSTGUG00000008237 | GAA | 0.234 | 0.032 | 24.0 | 18.0 | 14.28 |
| ENSTGUG00000013191 | SF3B14 | 0.234 | 0.032 | 24.0 | 18.0 | 14.14 |
| ENSTGUG00000006445 | COMMD7 | 0.234 | 0.032 | 24.0 | 18.0 | 14.13 |
| ENSTGUG00000012400 | MRPL13 | 0.234 | 0.032 | 24.0 | 17.0 | 13.92 |
| ENSTGUG00000007241 |  | 0.234 | 0.032 | 25.3 | 4.0 | 13.35 |
| ENSTGUG00000010853 | HIBCH | 0.234 | 0.032 | 24.0 | 4.0 | 13.12 |
| ENSTGUG00000013492 | PROS1 | 0.234 | 0.032 | 26.0 | 2.0 | 13.04 |
| ENSTGUG00000008318 | LIPA | 0.234 | 0.032 | 25.3 | 9.3 | 13.01 |
| ENSTGUG00000004789 | ZNF207 | 0.234 | 0.032 | 24.0 | 20.0 | 13.01 |
| ENSTGUG00000004083 | LBR | 0.234 | 0.032 | 25.3 | 9.3 | 12.96 |
| ENSTGUG00000005660 | SARDH | 0.234 | 0.032 | 24.0 | 10.0 | 12.76 |
| ENSTGUG00000000460 | GORASP1 | 0.234 | 0.032 | 24.0 | 5.0 | 12.70 |
| ENSTGUG00000011247 | CYP7B1 | 0.234 | 0.032 | 26.0 | 2.0 | 12.62 |
| ENSTGUG00000012671 | AASDHPPT | 0.234 | 0.032 | 24.0 | 1.0 | 12.58 |
| ENSTGUG00000004200 | FXN | 0.234 | 0.032 | 24.0 | 21.0 | 12.38 |
| ENSTGUG00000011047 | KIAA1598 | 0.234 | 0.032 | 24.0 | 5.0 | 12.30 |
| ENSTGUG00000001106 | RASSF5 | 0.234 | 0.032 | 25.3 | 9.3 | 12.29 |
| ENSTGUG00000009508 | KCTD15 | 0.234 | 0.032 | 24.0 | 1.0 | 12.23 |
| ENSTGUG00000005132 | IPMK | 0.234 | 0.032 | 24.0 | 6.0 | 12.12 |
| ENSTGUG00000005239 | RBMX2 | 0.234 | 0.032 | 25.3 | 12.7 | 12.06 |
| ENSTGUG00000010432 | SENP5 | 0.234 | 0.032 | 24.0 | 21.0 | 12.01 |
| ENSTGUG00000004956 | BFAR | 0.234 | 0.032 | 24.0 | 4.0 | 11.96 |
| ENSTGUG00000004969 | C10orf59 | 0.234 | 0.032 | 24.0 | 18.0 | 11.90 |
| ENSTGUG00000006013 | EVI5 | 0.234 | 0.032 | 25.3 | 9.3 | 11.83 |
| ENSTGUG00000014033 |  | 0.234 | 0.032 | 25.3 | 12.7 | 11.80 |
| ENSTGUG00000004333 | SGPL1 | 0.234 | 0.032 | 24.0 | 5.0 | 11.71 |
| ENSTGUG00000010593 | ARHGAP1 | 0.234 | 0.032 | 26.0 | 2.0 | 11.62 |
| ENSTGUG00000011789 | ACTC1 | 0.234 | 0.032 | 24.0 | 18.0 | 11.59 |
| ENSTGUG00000009676 | AP2A2 | 0.234 | 0.032 | 24.0 | 18.0 | 11.49 |
| ENSTGUG00000005686 | GPN3 | 0.234 | 0.032 | 24.0 | 18.0 | 11.21 |
| ENSTGUG00000003590 | TGFBR2 | 0.234 | 0.032 | 26.0 | 2.0 | 10.96 |
| ENSTGUG00000001289 | PTGR1 | 0.234 | 0.032 | 25.3 | 16.7 | 10.93 |
| ENSTGUG00000012290 | COQ3 | 0.234 | 0.032 | 24.0 | 18.0 | 10.82 |
| ENSTGUG00000011308 | SGK3 | 0.234 | 0.032 | 25.3 | 9.3 | 10.79 |
| ENSTGUG00000011401 | EFHA1 | 0.234 | 0.032 | 26.0 | 2.0 | 10.79 |
| ENSTGUG00000001127 | ACBD5 | 0.234 | 0.032 | 24.0 | 2.0 | 10.70 |
| ENSTGUG00000003964 | SH3BP5 | 0.234 | 0.032 | 26.0 | 2.0 | 10.68 |
| ENSTGUG00000009943 | PPP4R2 | 0.234 | 0.032 | 26.0 | 2.0 | 10.50 |
| ENSTGUG00000007649 | TMEM53 | 0.234 | 0.032 | 26.0 | 15.0 | 10.48 |
| ENSTGUG00000004446 | RNF128 | 0.234 | 0.032 | 24.0 | 5.0 | 10.41 |
| ENSTGUG00000003561 | NME3 | 0.234 | 0.032 | 26.0 | 15.0 | 10.38 |
| ENSTGUG00000011114 | IMPAD1 | 0.234 | 0.032 | 24.0 | 18.0 | 10.30 |
| ENSTGUG00000005634 | STARD3NL | 0.234 | 0.032 | 24.0 | 8.0 | 10.30 |
| ENSTGUG00000001258 | C5orf24 | 0.234 | 0.032 | 26.0 | 2.0 | 10.21 |
| ENSTGUG00000000658 |  | 0.234 | 0.032 | 25.3 | 12.7 | 10.03 |
| ENSTGUG00000012415 | DERL1 | 0.234 | 0.032 | 24.0 | 9.0 | 10.01 |
| ENSTGUG00000010062 | FZD5 | 0.234 | 0.032 | 24.0 | 9.0 | 9.95 |
| ENSTGUG00000014189 | SEC11A | 0.234 | 0.032 | 25.3 | 12.7 | 9.94 |
| ENSTGUG00000008073 | NIP7 | 0.234 | 0.032 | 25.3 | 12.7 | 9.93 |
| ENSTGUG00000016954 | RGS2 | 0.234 | 0.032 | 24.0 | 5.0 | 9.92 |
| ENSTGUG00000004252 | GSS | 0.234 | 0.032 | 24.0 | 22.0 | 9.89 |
| ENSTGUG00000002454 | GZMA | 0.234 | 0.032 | 26.0 | 18.0 | 9.53 |
| ENSTGUG00000006447 | NUP62CL | 0.234 | 0.032 | 25.3 | 12.7 | 9.44 |
| ENSTGUG00000015885 | SLC25A44 | 0.234 | 0.032 | 24.0 | 9.0 | 9.25 |
| ENSTGUG00000009133 | YIPF3 | 0.234 | 0.032 | 24.0 | 16.0 | 8.88 |
| ENSTGUG00000005212 | AAMP | 0.234 | 0.032 | 25.3 | 12.7 | 8.85 |
| ENSTGUG00000013130 |  | 0.234 | 0.032 | 24.0 | 1.0 | 8.78 |
| ENSTGUG00000005729 | TNPO1 | 0.234 | 0.032 | 25.3 | 9.3 | 8.75 |
| ENSTGUG00000005870 | SCO1 | 0.234 | 0.032 | 24.0 | 16.0 | 8.75 |
| ENSTGUG00000011532 | RDH10 | 0.234 | 0.032 | 24.0 | 6.0 | 8.74 |
| ENSTGUG00000006922 | FUNDC2 | 0.234 | 0.032 | 24.0 | 9.0 | 8.72 |
| ENSTGUG00000005886 | BCAR3 | 0.234 | 0.032 | 24.0 | 9.0 | 8.59 |
| ENSTGUG00000011698 |  | 0.234 | 0.032 | 24.0 | 2.0 | 8.44 |
| ENSTGUG00000004933 | FAAH2 | 0.234 | 0.032 | 26.0 | 15.0 | 8.44 |
| ENSTGUG00000017106 | MUL1 | 0.234 | 0.032 | 24.0 | 13.0 | 8.37 |
| ENSTGUG00000015883 | ZDHHC13 | 0.234 | 0.032 | 25.3 | 16.7 | 8.35 |
| ENSTGUG00000012549 | CLN5 | 0.234 | 0.032 | 28.0 | 16.0 | 8.35 |
| ENSTGUG00000002930 | HIBADH | 0.234 | 0.032 | 24.0 | 6.0 | 8.30 |
| ENSTGUG00000010595 | GREM2 | 0.234 | 0.032 | 24.0 | 9.0 | 8.09 |
| ENSTGUG00000009242 | PRKAB1 | 0.234 | 0.032 | 24.0 | 9.0 | 8.03 |
| ENSTGUG00000000961 | FBXL17 | 0.234 | 0.032 | 25.3 | 9.3 | 8.01 |
| ENSTGUG00000002261 | PSMD3 | 0.234 | 0.032 | 26.0 | 15.0 | 8.01 |
| ENSTGUG00000002190 | TUBB2A-2 | 0.234 | 0.032 | 24.0 | 10.0 | 7.96 |
| ENSTGUG00000012366 | EPSTI1 | 0.234 | 0.032 | 24.0 | 4.0 | 7.92 |
| ENSTGUG00000002095 | B5FZ53_TAEGU | 0.234 | 0.032 | 25.3 | 12.7 | 7.80 |
| ENSTGUG00000004105 | ADAR | 0.234 | 0.032 | 26.0 | 15.0 | 7.76 |
| ENSTGUG00000005275 | NADSYN1 | 0.234 | 0.032 | 24.0 | 21.0 | 7.76 |
| ENSTGUG00000004400 | SDR42E1 | 0.234 | 0.032 | 24.0 | 14.0 | 7.72 |
| ENSTGUG00000008337 | SLC6A6 | 0.234 | 0.032 | 25.3 | 9.3 | 7.65 |
| ENSTGUG00000001418 | P56LCK | 0.234 | 0.032 | 26.0 | 15.0 | 7.65 |
| ENSTGUG00000002322 | FAM107B | 0.234 | 0.032 | 24.0 | 4.0 | 7.65 |
| ENSTGUG00000016004 |  | 0.234 | 0.032 | 24.0 | 12.0 | 7.62 |
| ENSTGUG00000016617 | SCAMP3 | 0.234 | 0.032 | 25.3 | 12.7 | 7.57 |
| ENSTGUG00000013393 |  | 0.234 | 0.032 | 24.0 | 6.0 | 7.56 |
| ENSTGUG00000008294 | RANBP1 | 0.234 | 0.032 | 25.3 | 9.3 | 7.56 |
| ENSTGUG00000009191 | KCTD6 | 0.234 | 0.032 | 24.0 | 5.0 | 7.55 |
| ENSTGUG00000004795 | RNPC3 | 0.234 | 0.032 | 25.3 | 4.0 | 7.48 |
| ENSTGUG00000013547 | CXADR | 0.234 | 0.032 | 25.3 | 4.0 | 7.38 |
| ENSTGUG00000006174 | ALG13-2 | 0.234 | 0.032 | 24.0 | 17.0 | 7.37 |
| ENSTGUG00000010140 | SLC35D1 | 0.234 | 0.032 | 24.0 | 8.0 | 7.36 |
| ENSTGUG00000013251 | CDC5L | 0.234 | 0.032 | 24.0 | 18.0 | 7.35 |
| ENSTGUG00000000950 |  | 0.234 | 0.032 | 25.3 | 12.7 | 7.35 |
| ENSTGUG00000003658 | SLC27A4 | 0.234 | 0.032 | 24.0 | 14.0 | 7.23 |
| ENSTGUG00000016075 |  | 0.234 | 0.032 | 26.0 | 15.0 | 7.21 |
| ENSTGUG00000014607 |  | 0.234 | 0.032 | 24.0 | 18.0 | 7.12 |
| ENSTGUG00000007799 |  | 0.234 | 0.032 | 24.0 | 9.0 | 7.11 |
| ENSTGUG00000018383 |  | 0.234 | 0.032 | 25.3 | 12.7 | 7.10 |
| ENSTGUG00000013479 | FCHSD2 | 0.234 | 0.032 | 25.3 | 9.3 | 7.10 |
| ENSTGUG00000006417 | RBM5 | 0.234 | 0.032 | 24.0 | 21.0 | 7.07 |
| ENSTGUG00000014327 |  | 0.234 | 0.032 | 24.0 | 14.0 | 7.03 |
| ENSTGUG00000003050 | ATF3 | 0.234 | 0.032 | 25.3 | 4.0 | 7.02 |
| ENSTGUG00000011890 | NUBPL | 0.234 | 0.032 | 24.0 | 4.0 | 7.00 |
| ENSTGUG00000003559 | AK1 | 0.234 | 0.032 | 25.3 | 12.7 | 6.95 |
| ENSTGUG00000001366 | RPP38 | 0.234 | 0.032 | 24.0 | 18.0 | 6.90 |
| ENSTGUG00000004174 | HNF4A-1 | 0.234 | 0.032 | 24.0 | 9.0 | 6.88 |
| ENSTGUG00000005095 | SNRPD1 | 0.234 | 0.032 | 24.0 | 14.0 | 6.86 |
| ENSTGUG00000010516 | TTC39C | 0.234 | 0.032 | 26.0 | 2.0 | 6.81 |
| ENSTGUG00000004787 | C11orf46 | 0.234 | 0.032 | 25.3 | 9.3 | 6.72 |
| ENSTGUG00000002726 | AFF1 | 0.234 | 0.032 | 26.0 | 2.0 | 6.72 |
| ENSTGUG00000008442 | UNC84A | 0.234 | 0.032 | 24.0 | 17.0 | 6.69 |
| ENSTGUG00000009233 | ANKRD40-2 | 0.234 | 0.032 | 24.0 | 14.0 | 6.69 |
| ENSTGUG00000007423 | MPRIP | 0.234 | 0.032 | 25.3 | 4.0 | 6.66 |
| ENSTGUG00000010117 | COMMD9-1 | 0.234 | 0.032 | 24.0 | 21.0 | 6.53 |
| ENSTGUG00000009692 | EXOSC1 | 0.234 | 0.032 | 26.0 | 15.0 | 6.50 |
| ENSTGUG00000004115 | PLXNB2 | 0.234 | 0.032 | 25.3 | 9.3 | 6.46 |
| ENSTGUG00000008891 | VPS35 | 0.234 | 0.032 | 24.0 | 2.0 | 6.44 |
| ENSTGUG00000003720 | SNX33 | 0.234 | 0.032 | 25.3 | 12.7 | 6.43 |
| ENSTGUG00000001232 | FBXL21 | 0.234 | 0.032 | 24.0 | 17.0 | 6.43 |
| ENSTGUG00000016935 | MRTO4 | 0.234 | 0.032 | 26.0 | 15.0 | 6.36 |
| ENSTGUG00000003849 | C9orf16 | 0.234 | 0.032 | 26.0 | 15.0 | 6.34 |
| ENSTGUG00000003553 | POLD2 | 0.234 | 0.032 | 25.3 | 12.7 | 6.33 |
| ENSTGUG00000015702 |  | 0.234 | 0.032 | 26.0 | 15.0 | 6.30 |
| ENSTGUG00000004081 | ATP2C1 | 0.234 | 0.032 | 25.3 | 4.0 | 6.24 |
| ENSTGUG00000010926 |  | 0.234 | 0.032 | 24.0 | 9.0 | 6.24 |
| ENSTGUG00000018197 |  | 0.234 | 0.032 | 24.0 | 2.0 | 6.22 |
| ENSTGUG00000004757 | LEPROTL1 | 0.234 | 0.032 | 24.0 | 18.0 | 6.19 |
| ENSTGUG00000004870 | C16orf75 | 0.234 | 0.032 | 24.0 | 17.0 | 6.09 |
| ENSTGUG00000007724 | MVD | 0.234 | 0.032 | 26.0 | 15.0 | 6.06 |
| ENSTGUG00000017378 | CTSS | 0.234 | 0.032 | 24.0 | 14.0 | 6.06 |
| ENSTGUG00000013754 | FARSA | 0.234 | 0.032 | 26.0 | 15.0 | 6.03 |
| ENSTGUG00000005937 | DTYMK | 0.234 | 0.032 | 26.0 | 15.0 | 5.94 |
| ENSTGUG00000006302 | BMS1 | 0.234 | 0.032 | 24.0 | 17.0 | 5.93 |
| ENSTGUG00000010121 | GRPEL1 | 0.234 | 0.032 | 24.0 | 17.0 | 5.91 |
| ENSTGUG00000007693 | RNFT1 | 0.234 | 0.032 | 24.0 | 4.0 | 5.91 |
| ENSTGUG00000002848 | RUNDC1 | 0.234 | 0.032 | 26.0 | 15.0 | 5.90 |
| ENSTGUG00000016806 |  | 0.234 | 0.032 | 28.0 | 16.0 | 5.89 |
| ENSTGUG00000017360 | DDX6 | 0.234 | 0.032 | 26.0 | 2.0 | 5.86 |
| ENSTGUG00000010005 | ABCB10 | 0.234 | 0.032 | 25.3 | 9.3 | 5.84 |
| ENSTGUG00000014023 |  | 0.234 | 0.032 | 28.0 | 2.0 | 5.80 |
| ENSTGUG00000001075 | FAM54B | 0.234 | 0.032 | 26.0 | 15.0 | 5.80 |
| ENSTGUG00000009363 | MAP2K1 | 0.234 | 0.032 | 26.0 | 15.0 | 5.78 |
| ENSTGUG00000006328 | CDK5RAP1 | 0.234 | 0.032 | 26.0 | 15.0 | 5.75 |
| ENSTGUG00000003869 | SCAMP5 | 0.234 | 0.032 | 26.0 | 15.0 | 5.67 |
| ENSTGUG00000012219 | JDP2 | 0.234 | 0.032 | 25.3 | 12.7 | 5.64 |
| ENSTGUG00000007709 | MED13 | 0.234 | 0.032 | 26.0 | 2.0 | 5.57 |
| ENSTGUG00000006410 | VWCE | 0.234 | 0.032 | 24.0 | 10.0 | 5.55 |
| ENSTGUG00000001433 | PTTG1IP-1 | 0.234 | 0.032 | 26.0 | 2.0 | 5.55 |
| ENSTGUG00000008424 | C7orf20 | 0.234 | 0.032 | 24.0 | 0.0 | 5.46 |
| ENSTGUG00000012062 | SLC22A16 | 0.234 | 0.032 | 26.0 | 2.0 | 5.42 |
| ENSTGUG00000012347 | MAL2 | 0.234 | 0.032 | 25.3 | 9.3 | 5.36 |
| ENSTGUG00000011128 | CYP7A1 | 0.234 | 0.032 | 24.0 | 4.0 | 5.33 |
| ENSTGUG00000009824 | TBP | 0.234 | 0.032 | 24.0 | 21.0 | 5.27 |
| ENSTGUG00000003205 | CANT1 | 0.234 | 0.032 | 25.3 | 12.7 | 5.27 |
| ENSTGUG00000005983 |  | 0.234 | 0.032 | 26.0 | 2.0 | 5.25 |
| ENSTGUG00000010793 | ABHD13 | 0.234 | 0.032 | 24.0 | 21.0 | 5.24 |
| ENSTGUG00000010971 | TRUB1 | 0.234 | 0.032 | 26.0 | 15.0 | 5.19 |
| ENSTGUG00000003409 | ARL16 | 0.234 | 0.032 | 25.3 | 12.7 | 5.15 |
| ENSTGUG00000006390 | PPIF | 0.234 | 0.032 | 25.3 | 4.0 | 5.11 |
| ENSTGUG00000008864 | NCBP2 | 0.234 | 0.032 | 24.0 | 9.0 | 5.05 |
| ENSTGUG00000004436 | CBARA1 | 0.234 | 0.032 | 26.0 | 15.0 | 5.01 |
| ENSTGUG00000009845 | NHP2L1 | 0.234 | 0.032 | 24.0 | 13.0 | 4.98 |
| ENSTGUG00000009518 | PLA2G1B | 0.234 | 0.032 | 24.0 | 18.0 | 4.95 |
| ENSTGUG00000006735 | HSDL1 | 0.234 | 0.032 | 25.3 | 12.7 | 4.93 |
| ENSTGUG00000011568 | JPH1 | 0.234 | 0.032 | 26.0 | 2.0 | 4.92 |
| ENSTGUG00000010996 | ATP6V1H | 0.234 | 0.032 | 24.0 | 21.0 | 4.88 |
| ENSTGUG00000003571 | SPSB3 | 0.234 | 0.032 | 25.3 | 12.7 | 4.76 |
| ENSTGUG00000015716 | STAT6 | 0.234 | 0.032 | 25.3 | 16.7 | 4.74 |
| ENSTGUG00000006195 | VPS33A | 0.234 | 0.032 | 24.0 | 6.0 | 4.71 |
| ENSTGUG00000011167 | INPP5F | 0.234 | 0.032 | 25.3 | 9.3 | 4.64 |
| ENSTGUG00000012270 | C12orf5 | 0.234 | 0.032 | 24.0 | 5.0 | 4.64 |
| ENSTGUG00000009298 | TXNDC10 | 0.234 | 0.032 | 26.0 | 2.0 | 4.62 |
| ENSTGUG00000002040 | SOS1 | 0.234 | 0.032 | 24.0 | 6.0 | 4.62 |
| ENSTGUG00000012231 | TTC35 | 0.234 | 0.032 | 24.0 | 20.0 | 4.61 |
| ENSTGUG00000007019 | B9D1 | 0.234 | 0.032 | 25.3 | 12.7 | 4.57 |
| ENSTGUG00000010958 | PTPRA | 0.234 | 0.032 | 25.3 | 12.7 | 4.56 |
| ENSTGUG00000016965 | PQLC2 | 0.234 | 0.032 | 24.0 | 14.0 | 4.53 |
| ENSTGUG00000007298 | GALK2 | 0.234 | 0.032 | 24.0 | 14.0 | 4.50 |
| ENSTGUG00000012387 | SEL1L | 0.234 | 0.032 | 24.0 | 9.0 | 4.49 |
| ENSTGUG00000008273 | CHCHD4 | 0.234 | 0.032 | 24.0 | 14.0 | 4.47 |
| ENSTGUG00000002623 | PRPF4 | 0.234 | 0.032 | 25.3 | 12.7 | 4.47 |
| ENSTGUG00000015903 | INHBC | 0.234 | 0.032 | 24.0 | 5.0 | 4.46 |
| ENSTGUG00000008103 | MAPK8 | 0.234 | 0.032 | 24.0 | 22.0 | 4.43 |
| ENSTGUG00000006040 | CST7 | 0.234 | 0.032 | 24.0 | 18.0 | 4.43 |
| ENSTGUG00000010230 |  | 0.234 | 0.032 | 25.3 | 12.7 | 4.41 |
| ENSTGUG00000012360 | MAP3K7 | 0.234 | 0.032 | 24.0 | 2.0 | 4.34 |
| ENSTGUG00000008646 | MIF4GD | 0.234 | 0.032 | 25.3 | 12.7 | 4.33 |
| ENSTGUG00000001719 |  | 0.234 | 0.032 | 26.0 | 15.0 | 4.28 |
| ENSTGUG00000006666 |  | 0.234 | 0.032 | 24.0 | 21.0 | 4.28 |
| ENSTGUG00000016368 | A1CF | 0.234 | 0.032 | 24.0 | 9.0 | 4.26 |
| ENSTGUG00000007563 | PTCD1 | 0.234 | 0.032 | 26.0 | 15.0 | 4.25 |
| ENSTGUG00000010587 | SS18 | 0.234 | 0.032 | 26.0 | 15.0 | 4.16 |
| ENSTGUG00000017307 | TIA1 | 0.234 | 0.032 | 24.0 | 8.0 | 4.15 |
| ENSTGUG00000005720 | ARHGAP29 | 0.234 | 0.032 | 26.0 | 2.0 | 4.15 |
| ENSTGUG00000002822 |  | 0.234 | 0.032 | 26.0 | 15.0 | 4.13 |
| ENSTGUG00000014798 | SCAP | 0.234 | 0.032 | 24.0 | 13.0 | 4.11 |
| ENSTGUG00000011248 | RDX | 0.234 | 0.032 | 26.0 | 2.0 | 4.10 |
| ENSTGUG00000003010 |  | 0.234 | 0.032 | 26.0 | 15.0 | 4.08 |
| ENSTGUG00000007403 | TCIRG1 | 0.234 | 0.032 | 25.3 | 12.7 | 4.07 |
| ENSTGUG00000008956 | METTL9 | 0.234 | 0.032 | 24.0 | 1.0 | 4.06 |
| ENSTGUG00000000768 | C9orf41 | 0.234 | 0.032 | 24.0 | 22.0 | 4.05 |
| ENSTGUG00000005337 | IPPK | 0.234 | 0.032 | 24.0 | 9.0 | 4.04 |
| ENSTGUG00000008703 | NUP85 | 0.234 | 0.032 | 26.0 | 15.0 | 3.98 |
| ENSTGUG00000012946 | SH3YL1 | 0.234 | 0.032 | 24.0 | 2.0 | 3.97 |
| ENSTGUG00000004039 |  | 0.234 | 0.032 | 25.3 | 9.3 | 3.97 |
| ENSTGUG00000010113 | FES | 0.234 | 0.032 | 25.3 | 12.7 | 3.96 |
| ENSTGUG00000006187 | SEC16A | 0.234 | 0.032 | 24.0 | 2.0 | 3.96 |
| ENSTGUG00000008709 | TMEM189 | 0.234 | 0.032 | 24.0 | 21.0 | 3.95 |
| ENSTGUG00000007950 |  | 0.234 | 0.032 | 25.3 | 9.3 | 3.95 |
| ENSTGUG00000012945 | FAM110C | 0.234 | 0.032 | 24.0 | 5.0 | 3.94 |
| ENSTGUG00000016658 |  | 0.234 | 0.032 | 24.0 | 9.0 | 3.92 |
| ENSTGUG00000006676 | C18orf22 | 0.234 | 0.032 | 24.0 | 21.0 | 3.87 |
| ENSTGUG00000003570 | ENG | 0.234 | 0.032 | 25.3 | 12.7 | 3.84 |
| ENSTGUG00000003479 | LARP4 | 0.234 | 0.032 | 24.0 | 0.0 | 3.83 |
| ENSTGUG00000004064 | SDF4 | 0.234 | 0.032 | 24.0 | 8.0 | 3.81 |
| ENSTGUG00000012091 | NCALD | 0.234 | 0.032 | 24.0 | 9.0 | 3.76 |
| ENSTGUG00000009872 | FOXP1 | 0.234 | 0.032 | 26.0 | 2.0 | 3.74 |
| ENSTGUG00000015888 | LMNA-2 | 0.234 | 0.032 | 24.0 | 10.0 | 3.73 |
| ENSTGUG00000017245 |  | 0.234 | 0.032 | 24.0 | 9.0 | 3.69 |
| ENSTGUG00000010178 | ALG3 | 0.234 | 0.032 | 24.0 | 14.0 | 3.67 |
| ENSTGUG00000000183 | RNF214 | 0.234 | 0.032 | 25.3 | 12.7 | 3.66 |
| ENSTGUG00000009723 | WBP2NL | 0.234 | 0.032 | 25.3 | 12.7 | 3.66 |
| ENSTGUG00000017150 | RBM41 | 0.234 | 0.032 | 24.0 | 9.0 | 3.64 |
| ENSTGUG00000005921 | STK16 | 0.234 | 0.032 | 24.0 | 14.0 | 3.62 |
| ENSTGUG00000012534 | UCHL3 | 0.234 | 0.032 | 24.0 | 14.0 | 3.60 |
| ENSTGUG00000003410 | HGS | 0.234 | 0.032 | 25.3 | 12.7 | 3.60 |
| ENSTGUG00000010533 | LRP4 | 0.234 | 0.032 | 28.0 | 2.0 | 3.58 |
| ENSTGUG00000016126 | B5FYL7_TAEGU | 0.234 | 0.032 | 26.0 | 15.0 | 3.55 |
| ENSTGUG00000004378 | MPHOSPH6 | 0.234 | 0.032 | 24.0 | 21.0 | 3.54 |
| ENSTGUG00000003253 | IDH3A | 0.234 | 0.032 | 25.3 | 16.7 | 3.53 |
| ENSTGUG00000006609 | IRF10 | 0.234 | 0.032 | 26.0 | 15.0 | 3.50 |
| ENSTGUG00000013133 | FOXJ2 | 0.234 | 0.032 | 24.0 | 6.0 | 3.50 |
| ENSTGUG00000002225 | SRM | 0.234 | 0.032 | 24.0 | 14.0 | 3.48 |
| ENSTGUG00000011977 | PGCP | 0.234 | 0.032 | 24.0 | 6.0 | 3.47 |
| ENSTGUG00000001167 | ARHGAP21 | 0.234 | 0.032 | 24.0 | 2.0 | 3.47 |
| ENSTGUG00000004008 | CDS2 | 0.234 | 0.032 | 25.3 | 16.7 | 3.44 |
| ENSTGUG00000003219 | TMEM204 | 0.234 | 0.032 | 24.0 | 9.0 | 3.43 |
| ENSTGUG00000004167 |  | 0.234 | 0.032 | 24.0 | 13.0 | 3.42 |
| ENSTGUG00000010203 | GNG12 | 0.234 | 0.032 | 25.3 | 12.7 | 3.38 |
| ENSTGUG00000002911 | MLL5 | 0.234 | 0.032 | 26.0 | 2.0 | 3.37 |
| ENSTGUG00000007488 | C17orf71 | 0.234 | 0.032 | 25.3 | 12.7 | 3.36 |
| ENSTGUG00000001796 | C4orf16 | 0.234 | 0.032 | 24.0 | 14.0 | 3.35 |
| ENSTGUG00000012989 | CMPK2 | 0.234 | 0.032 | 24.0 | 8.0 | 3.32 |
| ENSTGUG00000010456 | PICK1 | 0.234 | 0.032 | 24.0 | 10.0 | 3.32 |
| ENSTGUG00000000591 | KIAA0409 | 0.234 | 0.032 | 25.3 | 12.7 | 3.31 |
| ENSTGUG00000001621 | NRG2 | 0.234 | 0.032 | 25.3 | 12.7 | 3.31 |
| ENSTGUG00000004610 | MAF | 0.234 | 0.032 | 24.0 | 9.0 | 3.30 |
| ENSTGUG00000012888 | TMEM14A | 0.234 | 0.032 | 24.0 | 0.0 | 3.26 |
| ENSTGUG00000010038 | XPNPEP3 | 0.234 | 0.032 | 24.0 | 21.0 | 3.23 |
| ENSTGUG00000005280 | NT5C3 | 0.234 | 0.032 | 26.0 | 15.0 | 3.20 |
| ENSTGUG00000013878 |  | 0.234 | 0.032 | 26.0 | 15.0 | 3.19 |
| ENSTGUG00000016979 |  | 0.234 | 0.032 | 25.3 | 16.7 | 3.18 |
| ENSTGUG00000010499 | B3GNT5 | 0.234 | 0.032 | 25.3 | 9.3 | 3.15 |
| ENSTGUG00000005386 | VLDLR | 0.234 | 0.032 | 24.0 | 14.0 | 3.15 |
| ENSTGUG00000012224 | WDSUB1 | 0.234 | 0.032 | 24.0 | 16.0 | 3.14 |
| ENSTGUG00000010657 | ACTL6A | 0.234 | 0.032 | 24.0 | 2.0 | 3.13 |
| ENSTGUG00000002347 | AGTRAP | 0.234 | 0.032 | 24.0 | 9.0 | 3.10 |
| ENSTGUG00000000653 | VPS26B | 0.234 | 0.032 | 24.0 | 9.0 | 3.06 |
| ENSTGUG00000006462 | WDR63 | 0.234 | 0.032 | 24.0 | 21.0 | 3.05 |
| ENSTGUG00000015360 |  | 0.234 | 0.032 | 24.0 | 6.0 | 3.05 |
| ENSTGUG00000009781 | EFCAB4A-2 | 0.234 | 0.032 | 24.0 | 0.0 | 3.04 |
| ENSTGUG00000017546 | COMMD10 | 0.234 | 0.032 | 24.0 | 17.0 | 3.04 |
| ENSTGUG00000007935 | CCDC117 | 0.234 | 0.032 | 26.0 | 2.0 | 3.01 |
| ENSTGUG00000006958 |  | 0.234 | 0.032 | 25.3 | 9.3 | 2.99 |
| ENSTGUG00000009466 |  | 0.234 | 0.032 | 24.0 | 6.0 | 2.99 |
| ENSTGUG00000015516 |  | 0.234 | 0.032 | 24.0 | 8.0 | 2.99 |
| ENSTGUG00000004995 | OCRL | 0.234 | 0.032 | 24.0 | 9.0 | 2.99 |
| ENSTGUG00000005869 | TBC1D7 | 0.234 | 0.032 | 25.3 | 16.7 | 2.99 |
| ENSTGUG00000016770 |  | 0.234 | 0.032 | 24.0 | 1.0 | 2.98 |
| ENSTGUG00000014324 |  | 0.234 | 0.032 | 24.0 | 20.0 | 2.94 |
| ENSTGUG00000007550 | SH3KBP1 | 0.234 | 0.032 | 24.0 | 6.0 | 2.94 |
| ENSTGUG00000010052 | UNC45A | 0.234 | 0.032 | 25.3 | 12.7 | 2.91 |
| ENSTGUG00000001157 | IL28RA | 0.234 | 0.032 | 24.0 | 2.0 | 2.90 |
| ENSTGUG00000006564 | TBC1D30 | 0.234 | 0.032 | 24.0 | 4.0 | 2.89 |
| ENSTGUG00000011590 | LNX2 | 0.234 | 0.032 | 25.3 | 4.0 | 2.88 |
| ENSTGUG00000001318 | RSU1 | 0.234 | 0.032 | 25.3 | 16.7 | 2.87 |
| ENSTGUG00000009590 | SLC25A26 | 0.234 | 0.032 | 24.0 | 18.0 | 2.86 |
| ENSTGUG00000000971 | TMEM222 | 0.234 | 0.032 | 24.0 | 12.0 | 2.86 |
| ENSTGUG00000012810 | WDR20 | 0.234 | 0.032 | 24.0 | 6.0 | 2.85 |
| ENSTGUG00000005574 | HIP1R | 0.234 | 0.032 | 24.0 | 9.0 | 2.84 |
| ENSTGUG00000004710 | GINS3 | 0.234 | 0.032 | 24.0 | 6.0 | 2.82 |
| ENSTGUG00000015594 |  | 0.234 | 0.032 | 24.0 | 20.0 | 2.81 |
| ENSTGUG00000008191 | CHST13 | 0.234 | 0.032 | 25.3 | 12.7 | 2.81 |
| ENSTGUG00000008249 | WBP2 | 0.234 | 0.032 | 24.0 | 8.0 | 2.79 |
| ENSTGUG00000010743 | C6orf103 | 0.234 | 0.032 | 28.0 | 2.0 | 2.78 |
| ENSTGUG00000000796 | DPP9 | 0.234 | 0.032 | 24.0 | 6.0 | 2.77 |
| ENSTGUG00000002856 | C5orf44 | 0.234 | 0.032 | 24.0 | 18.0 | 2.76 |
| ENSTGUG00000000746 | FAM62B | 0.234 | 0.032 | 25.3 | 9.3 | 2.75 |
| ENSTGUG00000004991 | LACTB | 0.234 | 0.032 | 25.3 | 9.3 | 2.73 |
| ENSTGUG00000016104 |  | 0.234 | 0.032 | 24.0 | 2.0 | 2.73 |
| ENSTGUG00000011045 | TMEM68-1 | 0.234 | 0.032 | 25.3 | 9.3 | 2.72 |
| ENSTGUG00000004365 | CDC42BPA | 0.234 | 0.032 | 26.0 | 2.0 | 2.72 |
| ENSTGUG00000004222 | DERL2 | 0.234 | 0.032 | 24.0 | 18.0 | 2.71 |
| ENSTGUG00000011234 | GGH | 0.234 | 0.032 | 26.0 | 2.0 | 2.66 |
| ENSTGUG00000018486 |  | 0.234 | 0.032 | 26.0 | 2.0 | 2.66 |
| ENSTGUG00000011423 | GALNAC4S-6ST | 0.234 | 0.032 | 24.0 | 14.0 | 2.65 |
| ENSTGUG00000001410 | SLU7 | 0.234 | 0.032 | 24.0 | 4.0 | 2.64 |
| ENSTGUG00000012464 |  | 0.234 | 0.032 | 24.0 | 4.0 | 2.63 |
| ENSTGUG00000000494 | CRYZL1 | 0.234 | 0.032 | 26.0 | 15.0 | 2.63 |
| ENSTGUG00000007841 | C12orf26 | 0.234 | 0.032 | 24.0 | 21.0 | 2.60 |
| ENSTGUG00000012815 | CINP | 0.234 | 0.032 | 24.0 | 16.0 | 2.59 |
| ENSTGUG00000001278 | IPO9 | 0.234 | 0.032 | 24.0 | 10.0 | 2.58 |
| ENSTGUG00000008165 | TSPAN1 | 0.234 | 0.032 | 25.3 | 12.7 | 2.58 |
| ENSTGUG00000001595 | TMCO6 | 0.234 | 0.032 | 25.3 | 12.7 | 2.56 |
| ENSTGUG00000014765 | CCDC25 | 0.234 | 0.032 | 24.0 | 14.0 | 2.56 |
| ENSTGUG00000007245 | DRG2 | 0.234 | 0.032 | 24.0 | 21.0 | 2.56 |
| ENSTGUG00000011332 | PCMT1 | 0.234 | 0.032 | 24.0 | 17.0 | 2.56 |
| ENSTGUG00000006448 | FERMT1 | 0.234 | 0.032 | 28.0 | 16.0 | 2.56 |
| ENSTGUG00000002961 | PUS7 | 0.234 | 0.032 | 25.3 | 9.3 | 2.56 |
| ENSTGUG00000002350 | MTHFR | 0.234 | 0.032 | 24.0 | 21.0 | 2.53 |
| ENSTGUG00000001048 | MTPAP | 0.234 | 0.032 | 24.0 | 4.0 | 2.53 |
| ENSTGUG00000006904 | CEP63 | 0.234 | 0.032 | 24.0 | 14.0 | 2.53 |
| ENSTGUG00000012982 | EED | 0.234 | 0.032 | 24.0 | 18.0 | 2.53 |
| ENSTGUG00000008231 | C1orf190 | 0.234 | 0.032 | 24.0 | 10.0 | 2.52 |
| ENSTGUG00000006343 | ANXA11 | 0.234 | 0.032 | 24.0 | 5.0 | 2.51 |
| ENSTGUG00000006791 | NARFL | 0.234 | 0.032 | 26.0 | 15.0 | 2.51 |
| ENSTGUG00000009602 | POP5 | 0.234 | 0.032 | 24.0 | 18.0 | 2.49 |
| ENSTGUG00000006075 | UTX | 0.234 | 0.032 | 26.0 | 2.0 | 2.48 |
| ENSTGUG00000000795 | TICAM1 | 0.234 | 0.032 | 25.3 | 12.7 | 2.48 |
| ENSTGUG00000008552 | ELP2 | 0.234 | 0.032 | 24.0 | 18.0 | 2.46 |
| ENSTGUG00000000202 | MBD3 | 0.234 | 0.032 | 24.0 | 10.0 | 2.45 |
| ENSTGUG00000007679 |  | 0.234 | 0.032 | 26.0 | 2.0 | 2.44 |
| ENSTGUG00000016730 |  | 0.234 | 0.032 | 24.0 | 8.0 | 2.44 |
| ENSTGUG00000002933 | FAM122B | 0.234 | 0.032 | 24.0 | 22.0 | 2.43 |
| ENSTGUG00000007872 | ANUBL1 | 0.234 | 0.032 | 26.0 | 2.0 | 2.39 |
| ENSTGUG00000006474 | EFCAB5 | 0.234 | 0.032 | 24.0 | 16.0 | 2.39 |
| ENSTGUG00000012597 |  | 0.234 | 0.032 | 24.0 | 14.0 | 2.38 |
| ENSTGUG00000018539 |  | 0.234 | 0.032 | 24.0 | 8.0 | 2.37 |
| ENSTGUG00000011535 | STAU2 | 0.234 | 0.032 | 25.3 | 9.3 | 2.34 |
| ENSTGUG00000003491 | C1orf27 | 0.234 | 0.032 | 24.0 | 6.0 | 2.32 |
| ENSTGUG00000009249 | WBP11 | 0.234 | 0.032 | 24.0 | 13.0 | 2.31 |
| ENSTGUG00000008710 | OLA1 | 0.234 | 0.032 | 24.0 | 4.0 | 2.29 |
| ENSTGUG00000009136 | IGDCC4 | 0.234 | 0.032 | 24.0 | 9.0 | 2.29 |
| ENSTGUG00000001822 | TAF1C | 0.234 | 0.032 | 25.3 | 12.7 | 2.28 |
| ENSTGUG00000004841 | MTG1 | 0.234 | 0.032 | 25.3 | 12.7 | 2.27 |
| ENSTGUG00000013714 |  | 0.234 | 0.032 | 26.0 | 15.0 | 2.26 |
| ENSTGUG00000010678 |  | 0.234 | 0.032 | 24.0 | 10.0 | 2.26 |
| ENSTGUG00000008753 |  | 0.234 | 0.032 | 24.0 | 4.0 | 2.26 |
| ENSTGUG00000008002 | ABTB1 | 0.234 | 0.032 | 24.0 | 2.0 | 2.25 |
| ENSTGUG00000008579 | CTNNAL1 | 0.234 | 0.032 | 24.0 | 6.0 | 2.25 |
| ENSTGUG00000016205 | SEC61A2 | 0.234 | 0.032 | 24.0 | 21.0 | 2.22 |
| ENSTGUG00000016967 | RGS4 | 0.234 | 0.032 | 24.0 | 6.0 | 2.22 |
| ENSTGUG00000009084 | ANKRD27 | 0.234 | 0.032 | 24.0 | 1.0 | 2.22 |
| ENSTGUG00000007909 | CDH4 | 0.234 | 0.032 | 24.0 | 12.0 | 2.21 |
| ENSTGUG00000007276 | KLHL15 | 0.234 | 0.032 | 24.0 | 9.0 | 2.20 |
| ENSTGUG00000017035 |  | 0.234 | 0.032 | 26.0 | 15.0 | 2.18 |
| ENSTGUG00000009908 | DRD5 | 0.234 | 0.032 | 24.0 | 5.0 | 2.18 |
| ENSTGUG00000000257 | FAM108A1 | 0.234 | 0.032 | 25.3 | 12.7 | 2.16 |
| ENSTGUG00000003556 | ST6GALNAC6 | 0.234 | 0.032 | 24.0 | 10.0 | 2.13 |
| ENSTGUG00000007615 | ERI3 | 0.234 | 0.032 | 24.0 | 8.0 | 2.12 |
| ENSTGUG00000005993 | RNF34 | 0.234 | 0.032 | 24.0 | 9.0 | 2.11 |
| ENSTGUG00000007016 | DUS2L | 0.234 | 0.032 | 24.0 | 18.0 | 2.10 |
| ENSTGUG00000008180 | NUDT1 | 0.234 | 0.032 | 24.0 | 14.0 | 2.06 |
| ENSTGUG00000000186 | CCDC12 | 0.234 | 0.032 | 24.0 | 16.0 | 2.05 |
| ENSTGUG00000001952 | LIMD2 | 0.234 | 0.032 | 26.0 | 15.0 | 2.04 |
| ENSTGUG00000013129 | NT5C1B | 0.234 | 0.032 | 28.0 | 16.0 | 2.04 |
| ENSTGUG00000009603 | MARVELD1 | 0.234 | 0.032 | 24.0 | 13.0 | 2.04 |
| ENSTGUG00000018130 |  | 0.234 | 0.032 | 24.0 | 12.0 | 2.02 |
| ENSTGUG00000010541 | LGALS1-2 | 0.234 | 0.032 | 24.0 | 21.0 | 2.02 |
| ENSTGUG00000000642 | GRLF1 | 0.234 | 0.032 | 24.0 | 9.0 | 1.99 |
| ENSTGUG00000003971 | CREBBP | 0.234 | 0.032 | 26.0 | 2.0 | 1.98 |
| ENSTGUG00000001953 | SLC35B4 | 0.234 | 0.032 | 24.0 | 13.0 | 1.98 |
| ENSTGUG00000011944 | EPC2 | 0.234 | 0.032 | 26.0 | 2.0 | 1.98 |
| ENSTGUG00000011366 | TTC9 | 0.234 | 0.032 | 24.0 | 14.0 | 1.97 |
| ENSTGUG00000005364 | SNRNP35 | 0.234 | 0.032 | 25.3 | 16.7 | 1.96 |
| ENSTGUG00000008706 | IFT20 | 0.234 | 0.032 | 26.0 | 15.0 | 1.95 |
| ENSTGUG00000004790 | TUBGCP2 | 0.234 | 0.032 | 26.0 | 15.0 | 1.94 |
| ENSTGUG00000010633 | FAM53A | 0.234 | 0.032 | 24.0 | 5.0 | 1.92 |
| ENSTGUG00000004335 | INTS10 | 0.234 | 0.032 | 24.0 | 18.0 | 1.90 |
| ENSTGUG00000009616 | MORN4 | 0.234 | 0.032 | 26.0 | 15.0 | 1.87 |
| ENSTGUG00000000212 | FBXO38 | 0.234 | 0.032 | 24.0 | 2.0 | 1.87 |
| ENSTGUG00000000829 | SGTA | 0.234 | 0.032 | 24.0 | 14.0 | 1.86 |
| ENSTGUG00000009631 | LIMS1 | 0.234 | 0.032 | 24.0 | 1.0 | 1.85 |
| ENSTGUG00000008321 | GALK1 | 0.234 | 0.032 | 24.0 | 14.0 | 1.85 |
| ENSTGUG00000013299 | BLK | 0.234 | 0.032 | 28.0 | 16.0 | 1.85 |
| ENSTGUG00000011228 | FGFR2 | 0.234 | 0.032 | 24.0 | 6.0 | 1.85 |
| ENSTGUG00000012990 | MNAT1 | 0.234 | 0.032 | 26.0 | 15.0 | 1.84 |
| ENSTGUG00000002344 | ABCA2 | 0.234 | 0.032 | 24.0 | 6.0 | 1.83 |
| ENSTGUG00000005918 | STAB1 | 0.234 | 0.032 | 24.0 | 16.0 | 1.83 |
| ENSTGUG00000008496 | PRKAR1B | 0.234 | 0.032 | 24.0 | 6.0 | 1.81 |
| ENSTGUG00000018457 |  | 0.234 | 0.032 | 25.3 | 12.7 | 1.79 |
| ENSTGUG00000015455 | CORO7 | 0.234 | 0.032 | 25.3 | 12.7 | 1.79 |
| ENSTGUG00000008746 |  | 0.234 | 0.032 | 24.0 | 20.0 | 1.79 |
| ENSTGUG00000002180 | MAL | 0.234 | 0.032 | 24.0 | 21.0 | 1.77 |
| ENSTGUG00000005545 | MUSTN1-2 | 0.234 | 0.032 | 24.0 | 18.0 | 1.76 |
| ENSTGUG00000005753 | B5KFM9_TAEGU | 0.234 | 0.032 | 24.0 | 17.0 | 1.76 |
| ENSTGUG00000014667 | FNDC4 | 0.234 | 0.032 | 24.0 | 12.0 | 1.76 |
| ENSTGUG00000007585 | TBCD | 0.234 | 0.032 | 24.0 | 18.0 | 1.76 |
| ENSTGUG00000009783 |  | 0.234 | 0.032 | 25.3 | 9.3 | 1.74 |
| ENSTGUG00000005042 | LIMD1 | 0.234 | 0.032 | 26.0 | 2.0 | 1.74 |
| ENSTGUG00000007145 | MTUS1 | 0.234 | 0.032 | 24.0 | 18.0 | 1.73 |
| ENSTGUG00000011465 | MTMR6 | 0.234 | 0.032 | 24.0 | 2.0 | 1.72 |
| ENSTGUG00000000995 | EPC1 | 0.234 | 0.032 | 26.0 | 2.0 | 1.72 |
| ENSTGUG00000008744 | RPUSD3 | 0.234 | 0.032 | 26.0 | 15.0 | 1.72 |
| ENSTGUG00000003896 | DNAJA3 | 0.234 | 0.032 | 24.0 | 13.0 | 1.72 |
| ENSTGUG00000010819 | TNFSF10-2 | 0.234 | 0.032 | 24.0 | 1.0 | 1.71 |
| ENSTGUG00000004702 | TAF15 | 0.234 | 0.032 | 24.0 | 14.0 | 1.70 |
| ENSTGUG00000004102 | BTBD12 | 0.234 | 0.032 | 24.0 | 16.0 | 1.70 |
| ENSTGUG00000003159 | TBC1D5 | 0.234 | 0.032 | 26.0 | 2.0 | 1.68 |
| ENSTGUG00000005527 | SLC16A9 | 0.234 | 0.032 | 24.0 | 5.0 | 1.67 |
| ENSTGUG00000014775 |  | 0.234 | 0.032 | 24.0 | 14.0 | 1.65 |
| ENSTGUG00000001968 | KIAA1109 | 0.234 | 0.032 | 25.3 | 12.7 | 1.65 |
| ENSTGUG00000007157 | HELLS | 0.234 | 0.032 | 24.0 | 16.0 | 1.65 |
| ENSTGUG00000003962 | ULK3 | 0.234 | 0.032 | 25.3 | 12.7 | 1.64 |
| ENSTGUG00000006473 | RBM41 | 0.234 | 0.032 | 25.3 | 12.7 | 1.63 |
| ENSTGUG00000002946 | DUSP3 | 0.234 | 0.032 | 25.3 | 9.3 | 1.63 |
| ENSTGUG00000008833 | BIRC6 | 0.234 | 0.032 | 24.0 | 2.0 | 1.62 |
| ENSTGUG00000013336 | NOP2 | 0.234 | 0.032 | 26.0 | 15.0 | 1.61 |
| ENSTGUG00000007697 | C17orf101 | 0.234 | 0.032 | 24.0 | 2.0 | 1.60 |
| ENSTGUG00000016901 |  | 0.234 | 0.032 | 24.0 | 10.0 | 1.60 |
| ENSTGUG00000012811 | FAM76B | 0.234 | 0.032 | 25.3 | 9.3 | 1.59 |
| ENSTGUG00000004483 | ASCC1 | 0.234 | 0.032 | 24.0 | 10.0 | 1.58 |
| ENSTGUG00000015076 |  | 0.234 | 0.032 | 24.0 | 6.0 | 1.54 |
| ENSTGUG00000006902 | KIAA0913-1 | 0.234 | 0.032 | 24.0 | 9.0 | 1.53 |
| ENSTGUG00000014874 |  | 0.234 | 0.032 | 24.0 | 6.0 | 1.53 |
| ENSTGUG00000011621 |  | 0.234 | 0.032 | 24.0 | 8.0 | 1.52 |
| ENSTGUG00000001059 | PDIK1L | 0.234 | 0.032 | 24.0 | 6.0 | 1.52 |
| ENSTGUG00000005859 | PIGX | 0.234 | 0.032 | 26.0 | 15.0 | 1.51 |
| ENSTGUG00000014782 |  | 0.234 | 0.032 | 24.0 | 9.0 | 1.51 |
| ENSTGUG00000016661 |  | 0.234 | 0.032 | 24.0 | 22.0 | 1.51 |
| ENSTGUG00000013171 |  | 0.234 | 0.032 | 24.0 | 16.0 | 1.49 |
| ENSTGUG00000011360 | P2RY12 | 0.234 | 0.032 | 25.3 | 12.7 | 1.49 |
| ENSTGUG00000003284 | SENP1 | 0.234 | 0.032 | 24.0 | 16.0 | 1.48 |
| ENSTGUG00000006143 | RAB34 | 0.234 | 0.032 | 25.3 | 12.7 | 1.48 |
| ENSTGUG00000000304 | PSD3-2 | 0.234 | 0.032 | 25.3 | 4.0 | 1.47 |
| ENSTGUG00000002760 | AR | 0.234 | 0.032 | 24.0 | 21.0 | 1.43 |
| ENSTGUG00000004007 | EDC3 | 0.234 | 0.032 | 25.3 | 12.7 | 1.42 |
| ENSTGUG00000002681 | ELOVL7 | 0.234 | 0.032 | 24.0 | 5.0 | 1.40 |
| ENSTGUG00000004305 | DNAJC13 | 0.234 | 0.032 | 26.0 | 2.0 | 1.39 |
| ENSTGUG00000010780 | FBXO30 | 0.234 | 0.032 | 26.0 | 2.0 | 1.38 |
| ENSTGUG00000000187 | AFF4 | 0.234 | 0.032 | 24.0 | 2.0 | 1.36 |
| ENSTGUG00000003507 | IGSF1 | 0.234 | 0.032 | 24.0 | 14.0 | 1.36 |
| ENSTGUG00000014780 |  | 0.234 | 0.032 | 26.0 | 15.0 | 1.35 |
| ENSTGUG00000014110 | NPEPL1 | 0.234 | 0.032 | 24.0 | 9.0 | 1.35 |
| ENSTGUG00000000181 | FZR1 | 0.234 | 0.032 | 24.0 | 10.0 | 1.34 |
| ENSTGUG00000001531 | CDCA8 | 0.234 | 0.032 | 28.0 | 16.0 | 1.34 |
| ENSTGUG00000017497 | FECH | 0.234 | 0.032 | 24.0 | 6.0 | 1.33 |
| ENSTGUG00000003364 | TK1 | 0.234 | 0.032 | 24.0 | 13.0 | 1.33 |
| ENSTGUG00000011472 | C10orf137 | 0.234 | 0.032 | 26.0 | 2.0 | 1.32 |
| ENSTGUG00000008110 | PTPN3-1 | 0.234 | 0.032 | 26.0 | 2.0 | 1.32 |
| ENSTGUG00000001753 | IL11RA-2 | 0.234 | 0.032 | 24.0 | 17.0 | 1.32 |
| ENSTGUG00000012306 | KLHL32 | 0.234 | 0.032 | 26.0 | 2.0 | 1.31 |
| ENSTGUG00000003958 | NOC2L | 0.234 | 0.032 | 26.0 | 15.0 | 1.30 |
| ENSTGUG00000001917 | MAD2L1 | 0.234 | 0.032 | 24.0 | 16.0 | 1.30 |
| ENSTGUG00000009273 | MAP3K4 | 0.234 | 0.032 | 26.0 | 2.0 | 1.30 |
| ENSTGUG00000005331 | KIAA0430 | 0.234 | 0.032 | 25.3 | 9.3 | 1.28 |
| ENSTGUG00000001494 | FAM133A | 0.234 | 0.032 | 24.0 | 21.0 | 1.28 |
| ENSTGUG00000004145 | ATG7 | 0.234 | 0.032 | 24.0 | 17.0 | 1.27 |
| ENSTGUG00000010302 | LRRC40 | 0.234 | 0.032 | 24.0 | 14.0 | 1.27 |
| ENSTGUG00000009774 | RAB31 | 0.234 | 0.032 | 24.0 | 0.0 | 1.26 |
| ENSTGUG00000018161 |  | 0.234 | 0.032 | 25.3 | 12.7 | 1.26 |
| ENSTGUG00000012310 | COG3 | 0.234 | 0.032 | 25.3 | 9.3 | 1.26 |
| ENSTGUG00000015032 |  | 0.234 | 0.032 | 25.3 | 12.7 | 1.25 |
| ENSTGUG00000011295 | PSPC1 | 0.234 | 0.032 | 25.3 | 16.7 | 1.25 |
| ENSTGUG00000004707 |  | 0.234 | 0.032 | 24.0 | 6.0 | 1.25 |
| ENSTGUG00000008575 | PIK3C2A | 0.234 | 0.032 | 26.0 | 2.0 | 1.24 |
| ENSTGUG00000003890 | NMRAL1 | 0.234 | 0.032 | 24.0 | 13.0 | 1.23 |
| ENSTGUG00000008872 | HPS5 | 0.234 | 0.032 | 24.0 | 6.0 | 1.23 |
| ENSTGUG00000004429 | RHBDF1 | 0.234 | 0.032 | 24.0 | 10.0 | 1.22 |
| ENSTGUG00000017448 | TIPARP | 0.234 | 0.032 | 26.0 | 15.0 | 1.21 |
| ENSTGUG00000001959 |  | 0.234 | 0.032 | 24.0 | 6.0 | 1.20 |
| ENSTGUG00000005913 | RAB3IL1 | 0.234 | 0.032 | 24.0 | 9.0 | 1.20 |
| ENSTGUG00000000155 | PIP5K1C | 0.234 | 0.032 | 24.0 | 9.0 | 1.20 |
| ENSTGUG00000002658 | KCNAB2 | 0.234 | 0.032 | 26.0 | 15.0 | 1.20 |
| ENSTGUG00000008268 | UNC13D | 0.234 | 0.032 | 24.0 | 12.0 | 1.19 |
| ENSTGUG00000008670 | PARD6B | 0.234 | 0.032 | 25.3 | 9.3 | 1.18 |
| ENSTGUG00000000207 | MYL3 | 0.234 | 0.032 | 25.3 | 12.7 | 1.17 |
| ENSTGUG00000006813 | MRRF | 0.234 | 0.032 | 24.0 | 18.0 | 1.17 |
| ENSTGUG00000003690 | HMG20A | 0.234 | 0.032 | 26.0 | 2.0 | 1.17 |
| ENSTGUG00000002232 |  | 0.234 | 0.032 | 24.0 | 1.0 | 1.16 |
| ENSTGUG00000000047 | DPYSL3 | 0.234 | 0.032 | 24.0 | 14.0 | 1.16 |
| ENSTGUG00000001912 | CPA1 | 0.234 | 0.032 | 24.0 | 13.0 | 1.15 |
| ENSTGUG00000014883 |  | 0.234 | 0.032 | 24.0 | 9.0 | 1.15 |
| ENSTGUG00000003876 | IQSEC1 | 0.234 | 0.032 | 24.0 | 13.0 | 1.14 |
| ENSTGUG00000013842 |  | 0.234 | 0.032 | 25.3 | 9.3 | 1.14 |
| ENSTGUG00000012782 | THUMPD2 | 0.234 | 0.032 | 24.0 | 10.0 | 1.13 |
| ENSTGUG00000001695 | SHROOM3 | 0.234 | 0.032 | 24.0 | 14.0 | 1.13 |
| ENSTGUG00000000660 | MED16 | 0.234 | 0.032 | 25.3 | 12.7 | 1.12 |
| ENSTGUG00000002781 | CNTD1 | 0.234 | 0.032 | 24.0 | 9.0 | 1.11 |
| ENSTGUG00000002833 | SCD5 | 0.234 | 0.032 | 24.0 | 6.0 | 1.11 |
| ENSTGUG00000018439 |  | 0.234 | 0.032 | 24.0 | 10.0 | 1.10 |
| ENSTGUG00000009135 | PCID2 | 0.234 | 0.032 | 24.0 | 21.0 | 1.10 |
| ENSTGUG00000001809 | RUSC2 | 0.234 | 0.032 | 24.0 | 10.0 | 1.10 |
| ENSTGUG00000009580 | CEP76 | 0.234 | 0.032 | 25.3 | 20.7 | 1.09 |
| ENSTGUG00000001955 | RNASEN | 0.234 | 0.032 | 26.0 | 2.0 | 1.09 |
| ENSTGUG00000016237 |  | 0.234 | 0.032 | 25.3 | 4.0 | 1.09 |
| ENSTGUG00000015778 |  | 0.234 | 0.032 | 25.3 | 9.3 | 1.08 |
| ENSTGUG00000000244 | ZW10 | 0.234 | 0.032 | 24.0 | 17.0 | 1.08 |
| ENSTGUG00000016006 |  | 0.234 | 0.032 | 26.0 | 15.0 | 1.07 |
| ENSTGUG00000003001 | TRA@-1 | 0.234 | 0.032 | 24.0 | 17.0 | 1.07 |
| ENSTGUG00000002436 | RAB33B | 0.234 | 0.032 | 24.0 | 6.0 | 1.06 |
| ENSTGUG00000012992 | RSAD2 | 0.234 | 0.032 | 24.0 | 6.0 | 1.05 |
| ENSTGUG00000000640 | C19orf21 | 0.234 | 0.032 | 24.0 | 8.0 | 1.05 |
| ENSTGUG00000002358 | MUTED-1 | 0.234 | 0.032 | 24.0 | 14.0 | 1.05 |
| ENSTGUG00000003120 | INTS7 | 0.234 | 0.032 | 25.3 | 9.3 | 1.02 |
| ENSTGUG00000003127 | SMARCAD1 | 0.234 | 0.032 | 24.0 | 20.0 | 1.02 |
| ENSTGUG00000000537 | IL10RB | 0.234 | 0.032 | 24.0 | 17.0 | 1.01 |
| ENSTGUG00000005350 | CCND1 | 0.234 | 0.032 | 24.0 | 16.0 | 1.00 |
| ENSTGUG00000012384 | RERGL | 0.234 | 0.032 | 26.0 | 2.0 | 0.99 |
| ENSTGUG00000002359 |  | 0.234 | 0.032 | 24.0 | 16.0 | 0.99 |
| ENSTGUG00000001543 | IL8-2 | 0.234 | 0.032 | 24.0 | 4.0 | 0.99 |
| ENSTGUG00000001765 | WRN | 0.234 | 0.032 | 24.0 | 6.0 | 0.98 |
| ENSTGUG00000004862 | LETM2 | 0.234 | 0.032 | 24.0 | 14.0 | 0.97 |
| ENSTGUG00000000669 | TMEM161B | 0.234 | 0.032 | 25.3 | 9.3 | 0.96 |
| ENSTGUG00000001236 | ARL5B | 0.234 | 0.032 | 24.0 | 6.0 | 0.96 |
| ENSTGUG00000002104 | PHF17 | 0.234 | 0.032 | 24.0 | 0.0 | 0.96 |
| ENSTGUG00000010863 | HMGXB4 | 0.234 | 0.032 | 24.0 | 21.0 | 0.96 |
| ENSTGUG00000014658 | KCNN1 | 0.234 | 0.032 | 25.3 | 12.7 | 0.95 |
| ENSTGUG00000013021 | KCNK16 | 0.234 | 0.032 | 24.0 | 6.0 | 0.95 |
| ENSTGUG00000004227 | CCDC14 | 0.234 | 0.032 | 24.0 | 9.0 | 0.95 |
| ENSTGUG00000010079 | SNRPD3 | 0.234 | 0.032 | 24.0 | 17.0 | 0.94 |
| ENSTGUG00000001266 | PABPC4 | 0.234 | 0.032 | 24.0 | 18.0 | 0.94 |
| ENSTGUG00000002079 | SERPINB2 | 0.234 | 0.032 | 24.0 | 8.0 | 0.94 |
| ENSTGUG00000009887 |  | 0.234 | 0.032 | 24.0 | 5.0 | 0.93 |
| ENSTGUG00000002948 | MPP3 | 0.234 | 0.032 | 24.0 | 16.0 | 0.93 |
| ENSTGUG00000004934 |  | 0.234 | 0.032 | 24.0 | 8.0 | 0.93 |
| ENSTGUG00000013902 | CWF19L2 | 0.234 | 0.032 | 24.0 | 20.0 | 0.91 |
| ENSTGUG00000017288 | CHMP7 | 0.234 | 0.032 | 24.0 | 14.0 | 0.90 |
| ENSTGUG00000000283 |  | 0.234 | 0.032 | 25.3 | 9.3 | 0.90 |
| ENSTGUG00000003855 | PPCDC | 0.234 | 0.032 | 24.0 | 16.0 | 0.90 |
| ENSTGUG00000006356 | RFX7 | 0.234 | 0.032 | 26.0 | 2.0 | 0.90 |
| ENSTGUG00000015292 | MTF2 | 0.234 | 0.032 | 25.3 | 16.7 | 0.89 |
| ENSTGUG00000010987 | FASTKD5 | 0.234 | 0.032 | 26.0 | 15.0 | 0.88 |
| ENSTGUG00000009487 | PPARGC1A | 0.234 | 0.032 | 26.0 | 2.0 | 0.88 |
| ENSTGUG00000009325 |  | 0.234 | 0.032 | 24.0 | 14.0 | 0.85 |
| ENSTGUG00000000751 | WDR60 | 0.234 | 0.032 | 24.0 | 20.0 | 0.85 |
| ENSTGUG00000013407 | KIAA1407 | 0.234 | 0.032 | 24.0 | 21.0 | 0.85 |
| ENSTGUG00000017076 |  | 0.234 | 0.032 | 24.0 | 18.0 | 0.83 |
| ENSTGUG00000002338 | SUV39H2 | 0.234 | 0.032 | 24.0 | 2.0 | 0.83 |
| ENSTGUG00000005168 |  | 0.234 | 0.032 | 24.0 | 20.0 | 0.83 |
| ENSTGUG00000008115 | NOC4L | 0.234 | 0.032 | 24.0 | 14.0 | 0.82 |
| ENSTGUG00000001491 | EFCAB1 | 0.234 | 0.032 | 26.0 | 15.0 | 0.82 |
| ENSTGUG00000005011 | TMEM169 | 0.234 | 0.032 | 24.0 | 4.0 | 0.82 |
| ENSTGUG00000000396 | GRK6 | 0.234 | 0.032 | 24.0 | 6.0 | 0.81 |
| ENSTGUG00000017543 |  | 0.234 | 0.032 | 24.0 | 1.0 | 0.81 |
| ENSTGUG00000016708 |  | 0.234 | 0.032 | 24.0 | 9.0 | 0.80 |
| ENSTGUG00000001596 | CD14 | 0.234 | 0.032 | 24.0 | 14.0 | 0.80 |
| ENSTGUG00000007879 | GATA2 | 0.234 | 0.032 | 24.0 | 14.0 | 0.79 |
| ENSTGUG00000016207 |  | 0.234 | 0.032 | 24.0 | 4.0 | 0.78 |
| ENSTGUG00000015272 |  | 0.234 | 0.032 | 24.0 | 10.0 | 0.78 |
| ENSTGUG00000002126 |  | 0.234 | 0.032 | 24.0 | 6.0 | 0.78 |
| ENSTGUG00000018279 |  | 0.234 | 0.032 | 24.0 | 6.0 | 0.78 |
| ENSTGUG00000005245 | RTKN2 | 0.234 | 0.032 | 24.0 | 9.0 | 0.77 |
| ENSTGUG00000005355 | CENPP | 0.234 | 0.032 | 24.0 | 18.0 | 0.77 |
| ENSTGUG00000003104 | SLC28A3 | 0.234 | 0.032 | 24.0 | 20.0 | 0.77 |
| ENSTGUG00000010893 | C10orf118 | 0.234 | 0.032 | 25.3 | 9.3 | 0.77 |
| ENSTGUG00000003283 | BZRAP1 | 0.234 | 0.032 | 24.0 | 6.0 | 0.77 |
| ENSTGUG00000013074 | FBXO34 | 0.234 | 0.032 | 24.0 | 13.0 | 0.76 |
| ENSTGUG00000007251 |  | 0.234 | 0.032 | 24.0 | 12.0 | 0.75 |
| ENSTGUG00000007435 | TCHP | 0.234 | 0.032 | 25.3 | 16.7 | 0.75 |
| ENSTGUG00000016941 | POLR3C | 0.234 | 0.032 | 26.0 | 15.0 | 0.75 |
| ENSTGUG00000003079 | SLC26A11 | 0.234 | 0.032 | 24.0 | 13.0 | 0.75 |
| ENSTGUG00000012469 | ZNF292 | 0.234 | 0.032 | 25.3 | 9.3 | 0.74 |
| ENSTGUG00000008216 | ERCC6 | 0.234 | 0.032 | 26.0 | 2.0 | 0.73 |
| ENSTGUG00000015231 |  | 0.234 | 0.032 | 24.0 | 2.0 | 0.73 |
| ENSTGUG00000006787 | FN3KRP | 0.234 | 0.032 | 24.0 | 20.0 | 0.72 |
| ENSTGUG00000009063 | ZYG11A | 0.234 | 0.032 | 26.0 | 2.0 | 0.72 |
| ENSTGUG00000000346 | MYO1F | 0.234 | 0.032 | 24.0 | 14.0 | 0.72 |
| ENSTGUG00000009015 | RASGRP3 | 0.234 | 0.032 | 24.0 | 6.0 | 0.71 |
| ENSTGUG00000011385 | C2orf67 | 0.234 | 0.032 | 26.0 | 2.0 | 0.71 |
| ENSTGUG00000017156 | CASP9 | 0.234 | 0.032 | 24.0 | 17.0 | 0.71 |
| ENSTGUG00000012809 | CEP57 | 0.234 | 0.032 | 24.0 | 21.0 | 0.70 |
| ENSTGUG00000014633 | ZNF493-6 | 0.234 | 0.032 | 25.3 | 12.7 | 0.70 |
| ENSTGUG00000006123 | C20orf94 | 0.234 | 0.032 | 24.0 | 5.0 | 0.70 |
| ENSTGUG00000004467 | ORAI2 | 0.234 | 0.032 | 24.0 | 9.0 | 0.69 |
| ENSTGUG00000006525 | PLAGL2 | 0.234 | 0.032 | 24.0 | 6.0 | 0.69 |
| ENSTGUG00000013349 | CD86 | 0.234 | 0.032 | 26.0 | 15.0 | 0.68 |
| ENSTGUG00000007143 | STAG1 | 0.234 | 0.032 | 24.0 | 18.0 | 0.68 |
| ENSTGUG00000003744 |  | 0.234 | 0.032 | 24.0 | 6.0 | 0.68 |
| ENSTGUG00000002758 | EZH1 | 0.234 | 0.032 | 24.0 | 16.0 | 0.67 |
| ENSTGUG00000010647 | DGKZ | 0.234 | 0.032 | 24.0 | 8.0 | 0.67 |
| ENSTGUG00000011055 | SYTL3 | 0.234 | 0.032 | 28.0 | 16.0 | 0.66 |
| ENSTGUG00000006711 | MLF1IP | 0.234 | 0.032 | 25.3 | 12.7 | 0.66 |
| ENSTGUG00000010625 |  | 0.234 | 0.032 | 24.0 | 6.0 | 0.66 |
| ENSTGUG00000009007 | PTPRJ | 0.234 | 0.032 | 25.3 | 9.3 | 0.66 |
| ENSTGUG00000007501 | SPTB | 0.234 | 0.032 | 25.3 | 12.7 | 0.65 |
| ENSTGUG00000000030 | KIAA0427 | 0.234 | 0.032 | 26.0 | 2.0 | 0.65 |
| ENSTGUG00000010409 | AOX1 | 0.234 | 0.032 | 24.0 | 6.0 | 0.64 |
| ENSTGUG00000008262 | LRRC41 | 0.234 | 0.032 | 24.0 | 18.0 | 0.64 |
| ENSTGUG00000006913 | INCENP-1 | 0.234 | 0.032 | 24.0 | 14.0 | 0.64 |
| ENSTGUG00000002476 | TTC25 | 0.234 | 0.032 | 24.0 | 18.0 | 0.64 |
| ENSTGUG00000000588 | FAM160A2 | 0.234 | 0.032 | 24.0 | 14.0 | 0.64 |
| ENSTGUG00000001006 | C5orf13 | 0.234 | 0.032 | 24.0 | 2.0 | 0.63 |
| ENSTGUG00000014651 |  | 0.234 | 0.032 | 25.3 | 12.7 | 0.63 |
| ENSTGUG00000014818 |  | 0.234 | 0.032 | 25.3 | 4.0 | 0.63 |
| ENSTGUG00000003197 | HDAC4 | 0.234 | 0.032 | 24.0 | 0.0 | 0.62 |
| ENSTGUG00000010890 |  | 0.234 | 0.032 | 24.0 | 9.0 | 0.62 |
| ENSTGUG00000006369 | CD5 | 0.234 | 0.032 | 26.0 | 15.0 | 0.62 |
| ENSTGUG00000002853 | ZC4H2 | 0.234 | 0.032 | 25.3 | 12.7 | 0.62 |
| ENSTGUG00000006471 | DLG5 | 0.234 | 0.032 | 26.0 | 2.0 | 0.61 |
| ENSTGUG00000010290 | LYST | 0.234 | 0.032 | 26.0 | 2.0 | 0.61 |
| ENSTGUG00000005219 |  | 0.234 | 0.032 | 24.0 | 18.0 | 0.60 |
| ENSTGUG00000006086 | GLMN | 0.234 | 0.032 | 24.0 | 6.0 | 0.59 |
| ENSTGUG00000007120 | ANKRD34B | 0.234 | 0.032 | 24.0 | 9.0 | 0.59 |
| ENSTGUG00000000871 | NDST1 | 0.234 | 0.032 | 24.0 | 13.0 | 0.59 |
| ENSTGUG00000010431 | MAFF | 0.234 | 0.032 | 24.0 | 12.0 | 0.58 |
| ENSTGUG00000018523 |  | 0.234 | 0.032 | 25.3 | 16.7 | 0.58 |
| ENSTGUG00000008335 | AEN | 0.234 | 0.032 | 24.0 | 12.0 | 0.57 |
| ENSTGUG00000009200 | C19orf40 | 0.234 | 0.032 | 24.0 | 17.0 | 0.57 |
| ENSTGUG00000018506 |  | 0.234 | 0.032 | 26.0 | 18.0 | 0.56 |
| ENSTGUG00000002648 | INPPL1 | 0.234 | 0.032 | 25.3 | 12.7 | 0.56 |
| ENSTGUG00000011340 | SYCP3 | 0.234 | 0.032 | 24.0 | 16.0 | 0.55 |
| ENSTGUG00000015669 |  | 0.234 | 0.032 | 24.0 | 8.0 | 0.55 |
| ENSTGUG00000011800 | KL | 0.234 | 0.032 | 24.0 | 9.0 | 0.54 |
| ENSTGUG00000005757 | ABCB6 | 0.234 | 0.032 | 24.0 | 14.0 | 0.54 |
| ENSTGUG00000013627 | ABI3BP | 0.234 | 0.032 | 24.0 | 4.0 | 0.54 |
| ENSTGUG00000001713 | SMPD2 | 0.234 | 0.032 | 24.0 | 14.0 | 0.54 |
| ENSTGUG00000004499 | WDR47 | 0.234 | 0.032 | 26.0 | 2.0 | 0.54 |
| ENSTGUG00000006866 | C1QTNF8 | 0.234 | 0.032 | 25.3 | 12.7 | 0.53 |
| ENSTGUG00000008393 | TERF2IP | 0.234 | 0.032 | 24.0 | 21.0 | 0.53 |
| ENSTGUG00000014790 |  | 0.234 | 0.032 | 28.0 | 0.0 | 0.52 |
| ENSTGUG00000018238 |  | 0.234 | 0.032 | 25.3 | 16.7 | 0.52 |
| ENSTGUG00000011074 | TFPI | 0.234 | 0.032 | 24.0 | 18.0 | 0.52 |
| ENSTGUG00000012430 | AKR1B10-1 | 0.234 | 0.032 | 24.0 | 5.0 | 0.52 |
| ENSTGUG00000003656 | OSBPL10 | 0.234 | 0.032 | 24.0 | 5.0 | 0.51 |
| ENSTGUG00000000748 | PCSK5 | 0.234 | 0.032 | 24.0 | 5.0 | 0.51 |
| ENSTGUG00000007264 | TLL2 | 0.234 | 0.032 | 25.3 | 12.7 | 0.51 |
| ENSTGUG00000002402 | THSD7A | 0.234 | 0.032 | 24.0 | 5.0 | 0.50 |
| ENSTGUG00000004722 | RBPMS2 | 0.234 | 0.032 | 24.0 | 4.0 | 0.50 |
| ENSTGUG00000015701 |  | 0.234 | 0.032 | 24.0 | 5.0 | 0.50 |
| ENSTGUG00000011928 | KPNA5 | 0.234 | 0.032 | 24.0 | 2.0 | 0.50 |
| ENSTGUG00000005596 | PRKAG3 | 0.234 | 0.032 | 24.0 | 8.0 | 0.49 |
| ENSTGUG00000010854 | MAP3K5 | 0.234 | 0.032 | 24.0 | 6.0 | 0.49 |
| ENSTGUG00000013901 |  | 0.234 | 0.032 | 25.3 | 12.7 | 0.49 |
| ENSTGUG00000005502 | MSH2 | 0.234 | 0.032 | 24.0 | 4.0 | 0.49 |
| ENSTGUG00000004299 | MLLT3 | 0.234 | 0.032 | 24.0 | 4.0 | 0.49 |
| ENSTGUG00000005784 | WDR33 | 0.234 | 0.032 | 24.0 | 17.0 | 0.49 |
| ENSTGUG00000011468 | DENND5B | 0.234 | 0.032 | 24.0 | 10.0 | 0.49 |
| ENSTGUG00000014566 | ZNF638 | 0.234 | 0.032 | 24.0 | 9.0 | 0.48 |
| ENSTGUG00000001756 | ANKS1A | 0.234 | 0.032 | 24.0 | 5.0 | 0.47 |
| ENSTGUG00000013041 | KLF11 | 0.234 | 0.032 | 24.0 | 13.0 | 0.47 |
| ENSTGUG00000009066 | ZNF318 | 0.234 | 0.032 | 25.3 | 9.3 | 0.47 |
| ENSTGUG00000001137 | SNCAIP | 0.234 | 0.032 | 24.0 | 9.0 | 0.46 |
| ENSTGUG00000001502 | PCDH18 | 0.234 | 0.032 | 24.0 | 8.0 | 0.46 |
| ENSTGUG00000003517 | THADA | 0.234 | 0.032 | 26.0 | 2.0 | 0.45 |
| ENSTGUG00000001267 | SUSD1 | 0.234 | 0.032 | 26.0 | 2.0 | 0.45 |
| ENSTGUG00000002563 | NOXA1 | 0.234 | 0.032 | 24.0 | 6.0 | 0.45 |
| ENSTGUG00000015304 |  | 0.234 | 0.032 | 24.0 | 16.0 | 0.44 |
| ENSTGUG00000008492 | CYP2R1 | 0.234 | 0.032 | 24.0 | 9.0 | 0.44 |
| ENSTGUG00000011885 | ARHGEF5 | 0.234 | 0.032 | 24.0 | 6.0 | 0.44 |
| ENSTGUG00000009356 | RASA3 | 0.234 | 0.032 | 25.3 | 9.3 | 0.43 |
| ENSTGUG00000017440 | C6orf49 | 0.234 | 0.032 | 24.0 | 14.0 | 0.43 |
| ENSTGUG00000016210 |  | 0.234 | 0.032 | 26.0 | 2.0 | 0.43 |
| ENSTGUG00000004822 | NUP214 | 0.234 | 0.032 | 25.3 | 12.7 | 0.43 |
| ENSTGUG00000004221 | MIS12 | 0.234 | 0.032 | 24.0 | 14.0 | 0.42 |
| ENSTGUG00000010001 | CRMP1 | 0.234 | 0.032 | 24.0 | 14.0 | 0.41 |
| ENSTGUG00000008409 |  | 0.234 | 0.032 | 24.0 | 2.0 | 0.41 |
| ENSTGUG00000010103 | SIPA1L2 | 0.234 | 0.032 | 25.3 | 9.3 | 0.41 |
| ENSTGUG00000000201 | MEX3D | 0.234 | 0.032 | 24.0 | 13.0 | 0.41 |
| ENSTGUG00000002462 | GPX8 | 0.234 | 0.032 | 24.0 | 5.0 | 0.41 |
| ENSTGUG00000005627 | SFRP4 | 0.234 | 0.032 | 24.0 | 1.0 | 0.41 |
| ENSTGUG00000009005 | TEKT1 | 0.234 | 0.032 | 24.0 | 6.0 | 0.40 |
| ENSTGUG00000013519 | ADPRH | 0.234 | 0.032 | 24.0 | 12.0 | 0.39 |
| ENSTGUG00000008592 |  | 0.234 | 0.032 | 24.0 | 10.0 | 0.39 |
| ENSTGUG00000001973 | ACE | 0.234 | 0.032 | 24.0 | 21.0 | 0.39 |
| ENSTGUG00000004387 | NAT2 | 0.234 | 0.032 | 25.3 | 9.3 | 0.38 |
| ENSTGUG00000014529 |  | 0.234 | 0.032 | 24.0 | 10.0 | 0.38 |
| ENSTGUG00000003118 | POM121 | 0.234 | 0.032 | 24.0 | 12.0 | 0.38 |
| ENSTGUG00000006060 | GFI1 | 0.234 | 0.032 | 28.0 | 16.0 | 0.38 |
| ENSTGUG00000014662 |  | 0.234 | 0.032 | 24.0 | 14.0 | 0.38 |
| ENSTGUG00000007939 | C15orf26 | 0.234 | 0.032 | 24.0 | 21.0 | 0.37 |
| ENSTGUG00000007188 | ZBTB5 | 0.234 | 0.032 | 24.0 | 9.0 | 0.37 |
| ENSTGUG00000012603 | ZFAT | 0.234 | 0.032 | 24.0 | 6.0 | 0.37 |
| ENSTGUG00000014079 |  | 0.234 | 0.032 | 24.0 | 14.0 | 0.37 |
| ENSTGUG00000015262 | DNM2 | 0.234 | 0.032 | 26.0 | 15.0 | 0.37 |
| ENSTGUG00000001447 | RLF | 0.234 | 0.032 | 24.0 | 2.0 | 0.37 |
| ENSTGUG00000009493 | UPF3A | 0.234 | 0.032 | 24.0 | 5.0 | 0.36 |
| ENSTGUG00000009287 | TSPAN32 | 0.234 | 0.032 | 24.0 | 6.0 | 0.36 |
| ENSTGUG00000003074 | FAM129B | 0.234 | 0.032 | 24.0 | 8.0 | 0.35 |
| ENSTGUG00000010645 | POLE | 0.234 | 0.032 | 24.0 | 13.0 | 0.33 |
| ENSTGUG00000003622 | FAM83C | 0.234 | 0.032 | 25.3 | 4.0 | 0.33 |
| ENSTGUG00000004461 | MKI67IP | 0.234 | 0.032 | 25.3 | 12.7 | 0.33 |
| ENSTGUG00000000983 | FLT4 | 0.234 | 0.032 | 26.0 | 2.0 | 0.33 |
| ENSTGUG00000009975 | EVC2 | 0.234 | 0.032 | 24.0 | 8.0 | 0.32 |
| ENSTGUG00000000174 | NBEAL1 | 0.234 | 0.032 | 25.3 | 12.7 | 0.32 |
| ENSTGUG00000011309 | ZMYM2 | 0.234 | 0.032 | 26.0 | 2.0 | 0.31 |
| ENSTGUG00000014518 |  | 0.234 | 0.032 | 24.0 | 4.0 | 0.31 |
| ENSTGUG00000016355 |  | 0.234 | 0.032 | 24.0 | 22.0 | 0.31 |
| ENSTGUG00000014797 |  | 0.234 | 0.032 | 24.0 | 2.0 | 0.31 |
| ENSTGUG00000004103 | H2AFY2 | 0.234 | 0.032 | 24.0 | 16.0 | 0.31 |
| ENSTGUG00000013705 |  | 0.234 | 0.032 | 24.0 | 5.0 | 0.30 |
| ENSTGUG00000002702 | EFNB1 | 0.234 | 0.032 | 24.0 | 13.0 | 0.30 |
| ENSTGUG00000006872 | FAP | 0.234 | 0.032 | 24.0 | 4.0 | 0.30 |
| ENSTGUG00000005609 | ADAMTSL2 | 0.234 | 0.032 | 24.0 | 8.0 | 0.30 |
| ENSTGUG00000011633 | HEY1 | 0.234 | 0.032 | 24.0 | 10.0 | 0.29 |
| ENSTGUG00000001755 | FBXL2 | 0.234 | 0.032 | 24.0 | 5.0 | 0.29 |
| ENSTGUG00000004760 | DOPEY2 | 0.234 | 0.032 | 26.0 | 2.0 | 0.29 |
| ENSTGUG00000006190 | NEK8 | 0.234 | 0.032 | 24.0 | 12.0 | 0.28 |
| ENSTGUG00000008736 | GAD1-2 | 0.234 | 0.032 | 24.0 | 18.0 | 0.27 |
| ENSTGUG00000005838 | PHACTR1 | 0.234 | 0.032 | 24.0 | 20.0 | 0.27 |
| ENSTGUG00000003863 | ODF2 | 0.234 | 0.032 | 26.0 | 15.0 | 0.27 |
| ENSTGUG00000005471 | SFMBT1 | 0.234 | 0.032 | 24.0 | 10.0 | 0.27 |
| ENSTGUG00000007242 | IL2RG | 0.234 | 0.032 | 24.0 | 14.0 | 0.27 |
| ENSTGUG00000003733 | GRAMD4 | 0.234 | 0.032 | 24.0 | 1.0 | 0.27 |
| ENSTGUG00000005193 | C9orf98 | 0.234 | 0.032 | 24.0 | 9.0 | 0.26 |
| ENSTGUG00000001112 | SRGAP2 | 0.234 | 0.032 | 24.0 | 13.0 | 0.26 |
| ENSTGUG00000000051 | LMNB2 | 0.234 | 0.032 | 24.0 | 6.0 | 0.26 |
| ENSTGUG00000009905 | SLC5A1 | 0.234 | 0.032 | 24.0 | 5.0 | 0.26 |
| ENSTGUG00000003623 |  | 0.234 | 0.032 | 24.0 | 1.0 | 0.26 |
| ENSTGUG00000001434 | PFTK1 | 0.234 | 0.032 | 26.0 | 2.0 | 0.26 |
| ENSTGUG00000016241 |  | 0.234 | 0.032 | 24.0 | 12.0 | 0.26 |
| ENSTGUG00000013355 | TBX15 | 0.234 | 0.032 | 24.0 | 20.0 | 0.26 |
| ENSTGUG00000012956 | DLG2 | 0.234 | 0.032 | 25.3 | 9.3 | 0.26 |
| ENSTGUG00000002280 | TRMT12 | 0.234 | 0.032 | 24.0 | 12.0 | 0.26 |
| ENSTGUG00000013456 | PGM2L1 | 0.234 | 0.032 | 24.0 | 2.0 | 0.25 |
| ENSTGUG00000008212 | AKT3 | 0.234 | 0.032 | 26.0 | 2.0 | 0.25 |
| ENSTGUG00000013529 | PDZK1 | 0.234 | 0.032 | 24.0 | 5.0 | 0.24 |
| ENSTGUG00000009430 | CNNM1 | 0.234 | 0.032 | 24.0 | 6.0 | 0.24 |
| ENSTGUG00000002819 | EHMT1 | 0.234 | 0.032 | 24.0 | 9.0 | 0.24 |
| ENSTGUG00000010436 | ACTN2 | 0.234 | 0.032 | 24.0 | 8.0 | 0.23 |
| ENSTGUG00000017309 | C1orf112 | 0.234 | 0.032 | 24.0 | 6.0 | 0.23 |
| ENSTGUG00000012102 | FMNL2 | 0.234 | 0.032 | 24.0 | 9.0 | 0.23 |
| ENSTGUG00000011120 | UBXN2B | 0.234 | 0.032 | 24.0 | 6.0 | 0.23 |
| ENSTGUG00000003550 | FAM102A | 0.234 | 0.032 | 24.0 | 2.0 | 0.23 |
| ENSTGUG00000007795 | BEND2 | 0.234 | 0.032 | 26.0 | 2.0 | 0.22 |
| ENSTGUG00000011796 | SLC7A7 | 0.234 | 0.032 | 25.3 | 9.3 | 0.22 |
| ENSTGUG00000003952 | SAMD11 | 0.234 | 0.032 | 24.0 | 6.0 | 0.22 |
| ENSTGUG00000002170 | RIPK1 | 0.234 | 0.032 | 24.0 | 16.0 | 0.21 |
| ENSTGUG00000001072 | DDX49 | 0.234 | 0.032 | 24.0 | 12.0 | 0.21 |
| ENSTGUG00000008724 | SNAI1 | 0.234 | 0.032 | 24.0 | 6.0 | 0.20 |
| ENSTGUG00000009735 | FHL2 | 0.234 | 0.032 | 24.0 | 13.0 | 0.20 |
| ENSTGUG00000003258 | TBC1D24 | 0.234 | 0.032 | 24.0 | 6.0 | 0.20 |
| ENSTGUG00000005624 | RLTPR | 0.234 | 0.032 | 24.0 | 9.0 | 0.20 |
| ENSTGUG00000008618 | LIMCH1 | 0.234 | 0.032 | 25.3 | 9.3 | 0.20 |
| ENSTGUG00000008359 |  | 0.234 | 0.032 | 24.0 | 21.0 | 0.20 |
| ENSTGUG00000015604 |  | 0.234 | 0.032 | 24.0 | 18.0 | 0.20 |
| ENSTGUG00000010272 | CNGA3 | 0.234 | 0.032 | 24.0 | 9.0 | 0.20 |
| ENSTGUG00000003278 | CXorf39 | 0.234 | 0.032 | 26.0 | 2.0 | 0.19 |
| ENSTGUG00000012609 | RAD52 | 0.234 | 0.032 | 24.0 | 16.0 | 0.19 |
| ENSTGUG00000009367 | PARK2 | 0.234 | 0.032 | 24.0 | 6.0 | 0.19 |
| ENSTGUG00000001340 | TRIM36-1 | 0.234 | 0.032 | 25.3 | 4.0 | 0.18 |
| ENSTGUG00000005840 | TMEM171 | 0.234 | 0.032 | 24.0 | 16.0 | 0.18 |
| ENSTGUG00000000757 | ROBO4 | 0.234 | 0.032 | 24.0 | 16.0 | 0.18 |
| ENSTGUG00000012830 | BMP5 | 0.234 | 0.032 | 24.0 | 16.0 | 0.17 |
| ENSTGUG00000006563 | ZNF503 | 0.234 | 0.032 | 24.0 | 13.0 | 0.17 |
| ENSTGUG00000003506 | PPL | 0.234 | 0.032 | 24.0 | 8.0 | 0.17 |
| ENSTGUG00000009830 |  | 0.234 | 0.032 | 24.0 | 5.0 | 0.17 |
| ENSTGUG00000008437 | AHCTF1 | 0.234 | 0.032 | 26.0 | 2.0 | 0.17 |
| ENSTGUG00000018292 |  | 0.234 | 0.032 | 24.0 | 6.0 | 0.16 |
| ENSTGUG00000010667 | PDCD4 | 0.234 | 0.032 | 24.0 | 9.0 | 0.15 |
| ENSTGUG00000014130 |  | 0.234 | 0.032 | 24.0 | 9.0 | 0.15 |
| ENSTGUG00000002972 | AZI1 | 0.234 | 0.032 | 24.0 | 17.0 | 0.15 |
| ENSTGUG00000006632 | HELB | 0.234 | 0.032 | 24.0 | 4.0 | 0.15 |
| ENSTGUG00000004821 | DUSP4 | 0.234 | 0.032 | 24.0 | 17.0 | 0.14 |
| ENSTGUG00000001817 | HECW1 | 0.234 | 0.032 | 28.0 | 2.0 | 0.14 |
| ENSTGUG00000005935 | CCDC18 | 0.234 | 0.032 | 24.0 | 9.0 | 0.14 |
| ENSTGUG00000008998 | MYCBPAP | 0.234 | 0.032 | 24.0 | 22.0 | 0.14 |
| ENSTGUG00000000891 | SLC16A4 | 0.234 | 0.032 | 24.0 | 12.0 | 0.14 |
| ENSTGUG00000001996 | IL7R | 0.234 | 0.032 | 24.0 | 9.0 | 0.14 |
| ENSTGUG00000003935 | LMLN | 0.234 | 0.032 | 24.0 | 13.0 | 0.14 |
| ENSTGUG00000007806 | REPS2 | 0.234 | 0.032 | 24.0 | 13.0 | 0.13 |
| ENSTGUG00000003580 | CEP250 | 0.234 | 0.032 | 24.0 | 18.0 | 0.13 |
| ENSTGUG00000003398 | COL27A1 | 0.234 | 0.032 | 24.0 | 14.0 | 0.13 |
| ENSTGUG00000004882 | BIN1 | 0.234 | 0.032 | 24.0 | 5.0 | 0.13 |
| ENSTGUG00000006998 | SSH1 | 0.234 | 0.032 | 24.0 | 18.0 | 0.13 |
| ENSTGUG00000003801 | ANKS3 | 0.234 | 0.032 | 24.0 | 4.0 | 0.13 |
| ENSTGUG00000013666 |  | 0.234 | 0.032 | 24.0 | 18.0 | 0.13 |
| ENSTGUG00000011356 | MAP3K9 | 0.234 | 0.032 | 24.0 | 5.0 | 0.13 |
| ENSTGUG00000012325 | SLCO1C1 | 0.234 | 0.032 | 24.0 | 21.0 | 0.13 |
| ENSTGUG00000005035 | MST1R | 0.234 | 0.032 | 24.0 | 6.0 | 0.12 |
| ENSTGUG00000013805 |  | 0.234 | 0.032 | 24.0 | 5.0 | 0.12 |
| ENSTGUG00000011137 | STARD9-2 | 0.234 | 0.032 | 24.0 | 2.0 | 0.12 |
| ENSTGUG00000002059 |  | 0.234 | 0.032 | 24.0 | 20.0 | 0.12 |
| ENSTGUG00000016996 |  | 0.234 | 0.032 | 24.0 | 4.0 | 0.12 |
| ENSTGUG00000000896 | C19orf26 | 0.234 | 0.032 | 24.0 | 18.0 | 0.12 |
| ENSTGUG00000003249 | MPO | 0.234 | 0.032 | 28.0 | 0.0 | 0.12 |
| ENSTGUG00000000530 | PRDM10 | 0.234 | 0.032 | 24.0 | 2.0 | 0.11 |
| ENSTGUG00000016761 | RIF1 | 0.234 | 0.032 | 24.0 | 9.0 | 0.11 |
| ENSTGUG00000001978 | TANC2 | 0.234 | 0.032 | 26.0 | 18.0 | 0.11 |
| ENSTGUG00000003651 | WDR90 | 0.234 | 0.032 | 24.0 | 16.0 | 0.11 |
| ENSTGUG00000005159 | ZMAT4 | 0.234 | 0.032 | 24.0 | 16.0 | 0.11 |
| ENSTGUG00000006645 | STOX2 | 0.234 | 0.032 | 24.0 | 17.0 | 0.10 |
| ENSTGUG00000014059 |  | 0.234 | 0.032 | 24.0 | 4.0 | 0.10 |
| ENSTGUG00000007877 | TMC3 | 0.234 | 0.032 | 24.0 | 2.0 | 0.10 |
| ENSTGUG00000008565 | ATP9A | 0.234 | 0.032 | 24.0 | 4.0 | 0.10 |
| ENSTGUG00000003181 | SLC26A3 | 0.234 | 0.032 | 24.0 | 1.0 | 0.10 |
| ENSTGUG00000017462 | ZNF493-26 | 0.234 | 0.032 | 25.3 | 12.7 | 0.10 |
| ENSTGUG00000006252 | SLC4A3 | 0.234 | 0.032 | 24.0 | 16.0 | 0.09 |
| ENSTGUG00000003921 | ATP8B2-1 | 0.234 | 0.032 | 24.0 | 9.0 | 0.09 |
| ENSTGUG00000000276 | NKTR | 0.234 | 0.032 | 24.0 | 16.0 | 0.09 |
| ENSTGUG00000009705 | THBS2 | 0.234 | 0.032 | 24.0 | 12.0 | 0.09 |
| ENSTGUG00000003084 | MMRN1 | 0.234 | 0.032 | 24.0 | 16.0 | 0.08 |
| ENSTGUG00000001583 | PHTF1 | 0.234 | 0.032 | 24.0 | 6.0 | 0.08 |
| ENSTGUG00000013363 | KIAA2018 | 0.234 | 0.032 | 24.0 | 2.0 | 0.08 |
| ENSTGUG00000008003 | GUCY1B2 | 0.234 | 0.032 | 24.0 | 17.0 | 0.08 |
| ENSTGUG00000003207 | DNAH17 | 0.234 | 0.032 | 24.0 | 6.0 | 0.08 |
| ENSTGUG00000006206 | CAPN6 | 0.234 | 0.032 | 25.3 | 9.3 | 0.07 |
| ENSTGUG00000005736 |  | 0.234 | 0.032 | 24.0 | 18.0 | 0.07 |
| ENSTGUG00000014697 |  | 0.234 | 0.032 | 24.0 | 4.0 | 0.07 |
| ENSTGUG00000006227 | LRIG3 | 0.234 | 0.032 | 24.0 | 6.0 | 0.06 |
| ENSTGUG00000002591 | ZNF618 | 0.234 | 0.032 | 24.0 | 20.0 | 0.06 |
| ENSTGUG00000009037 | SLC5A8 | 0.234 | 0.032 | 24.0 | 4.0 | 0.05 |
| ENSTGUG00000001113 | PTCHD3 | 0.234 | 0.032 | 24.0 | 17.0 | 0.05 |
| ENSTGUG00000002501 | ZNF385C | 0.234 | 0.032 | 24.0 | 5.0 | 0.05 |
| ENSTGUG00000001508 |  | 0.234 | 0.032 | 24.0 | 18.0 | 0.05 |
| ENSTGUG00000002821 | DFNA5 | 0.234 | 0.032 | 24.0 | 6.0 | 0.04 |
| ENSTGUG00000012915 | C14orf79 | 0.234 | 0.032 | 24.0 | 18.0 | 0.04 |
| ENSTGUG00000000532 | PTCH1 | 0.234 | 0.032 | 24.0 | 1.0 | 0.04 |
| ENSTGUG00000011419 | SACS | 0.234 | 0.032 | 24.0 | 6.0 | 0.04 |
| ENSTGUG00000012496 | C13orf34 | 0.234 | 0.032 | 24.0 | 16.0 | 0.03 |
| ENSTGUG00000003210 | LPO | 0.234 | 0.032 | 28.0 | 16.0 | 0.03 |
| ENSTGUG00000014916 | GABRP | 0.234 | 0.032 | 24.0 | 18.0 | 0.03 |
| ENSTGUG00000009575 | SLIT2 | 0.234 | 0.032 | 24.0 | 16.0 | 0.03 |
| ENSTGUG00000010340 | RNFT2 | 0.234 | 0.032 | 24.0 | 8.0 | 0.02 |
| ENSTGUG00000015078 |  | 0.234 | 0.032 | 24.0 | 4.0 | 0.02 |
| ENSTGUG00000004184 | CDH23 | 0.234 | 0.032 | 28.0 | 16.0 | 0.02 |
| ENSTGUG00000010908 | CTNNA2 | 0.234 | 0.032 | 24.0 | 18.0 | 0.01 |
| ENSTGUG00000017542 | AP3S1 | 0.003 | 0.000 | 24.0 | 18.0 | 13.63 |
| ENSTGUG00000010054 | UPB1 | 0.003 | 0.000 | 24.0 | 6.0 | 237.10 |
| ENSTGUG00000001974 | AGXT2 | 0.003 | 0.000 | 24.0 | 4.0 | 136.75 |
| ENSTGUG00000000800 | ALDH1A1 | 0.003 | 0.000 | 24.0 | 6.0 | 134.65 |
| ENSTGUG00000001054 | CANX | 0.003 | 0.000 | 24.0 | 6.0 | 114.63 |
| ENSTGUG00000011323 | IYD | 0.003 | 0.000 | 24.0 | 6.0 | 77.18 |
| ENSTGUG00000008312 | H3F3B | 0.003 | 0.000 | 24.0 | 18.0 | 71.49 |
| ENSTGUG00000010443 | ACADM | 0.003 | 0.000 | 24.0 | 4.0 | 69.16 |
| ENSTGUG00000000418 | AUH | 0.003 | 0.000 | 24.0 | 6.0 | 61.79 |
| ENSTGUG00000009196 | NUDT7 | 0.003 | 0.000 | 24.0 | 16.0 | 45.91 |
| ENSTGUG00000010549 | COQ10B | 0.003 | 0.000 | 24.0 | 2.0 | 41.74 |
| ENSTGUG00000004281 | PTPLB | 0.003 | 0.000 | 24.0 | 2.0 | 40.26 |
| ENSTGUG00000014474 |  | 0.003 | 0.000 | 24.0 | 18.0 | 34.58 |
| ENSTGUG00000016944 |  | 0.003 | 0.000 | 24.0 | 2.0 | 29.80 |
| ENSTGUG00000009402 | SNAPC5 | 0.003 | 0.000 | 24.0 | 6.0 | 26.18 |
| ENSTGUG00000016040 |  | 0.003 | 0.000 | 24.0 | 22.0 | 24.41 |
| ENSTGUG00000010307 | ARL8B | 0.003 | 0.000 | 24.0 | 6.0 | 23.71 |
| ENSTGUG00000001254 | HSDL2 | 0.003 | 0.000 | 24.0 | 4.0 | 22.46 |
| ENSTGUG00000012846 | TINAG | 0.003 | 0.000 | 24.0 | 4.0 | 18.41 |
| ENSTGUG00000009876 | WDR1 | 0.003 | 0.000 | 24.0 | 0.0 | 17.81 |
| ENSTGUG00000012239 | SUCLA2 | 0.003 | 0.000 | 24.0 | 22.0 | 15.77 |
| ENSTGUG00000008391 | CHST4 | 0.003 | 0.000 | 24.0 | 6.0 | 15.61 |
| ENSTGUG00000012178 | DPYS | 0.003 | 0.000 | 24.0 | 2.0 | 14.53 |
| ENSTGUG00000004234 | TMEM194A | 0.003 | 0.000 | 24.0 | 12.0 | 14.26 |
| ENSTGUG00000001372 | ZBTB8OS | 0.003 | 0.000 | 24.0 | 4.0 | 12.40 |
| ENSTGUG00000009139 | PODN | 0.003 | 0.000 | 24.0 | 4.0 | 11.42 |
| ENSTGUG00000002135 | RBMX | 0.003 | 0.000 | 24.0 | 18.0 | 10.93 |
| ENSTGUG00000010585 | CDC42EP1 | 0.003 | 0.000 | 24.0 | 6.0 | 10.91 |
| ENSTGUG00000005401 | FRRS1 | 0.003 | 0.000 | 24.0 | 6.0 | 10.50 |
| ENSTGUG00000003954 | METTL6 | 0.003 | 0.000 | 24.0 | 18.0 | 10.04 |
| ENSTGUG00000005819 | ABCB7 | 0.003 | 0.000 | 24.0 | 4.0 | 9.28 |
| ENSTGUG00000004981 | UCK1 | 0.003 | 0.000 | 24.0 | 6.0 | 9.22 |
| ENSTGUG00000006035 | DTNBP1 | 0.003 | 0.000 | 24.0 | 0.0 | 7.18 |
| ENSTGUG00000012434 | TTC8 | 0.003 | 0.000 | 24.0 | 22.0 | 7.05 |
| ENSTGUG00000000103 | TRIM14 | 0.003 | 0.000 | 24.0 | 2.0 | 6.73 |
| ENSTGUG00000007048 | NONO | 0.003 | 0.000 | 24.0 | 18.0 | 6.61 |
| ENSTGUG00000005104 | TOMM34 | 0.003 | 0.000 | 24.0 | 12.0 | 6.48 |
| ENSTGUG00000012405 | SNTB1 | 0.003 | 0.000 | 24.0 | 4.0 | 6.27 |
| ENSTGUG00000014081 |  | 0.003 | 0.000 | 24.0 | 14.0 | 6.27 |
| ENSTGUG00000014951 |  | 0.003 | 0.000 | 24.0 | 6.0 | 5.18 |
| ENSTGUG00000012200 | ATG5 | 0.003 | 0.000 | 24.0 | 20.0 | 4.93 |
| ENSTGUG00000004596 | C16orf68 | 0.003 | 0.000 | 24.0 | 4.0 | 4.60 |
| ENSTGUG00000015256 | PEF1 | 0.003 | 0.000 | 24.0 | 16.0 | 4.32 |
| ENSTGUG00000006114 |  | 0.003 | 0.000 | 24.0 | 18.0 | 4.03 |
| ENSTGUG00000005413 | CAB39 | 0.003 | 0.000 | 24.0 | 0.0 | 3.99 |
| ENSTGUG00000013701 | HFE2 | 0.003 | 0.000 | 24.0 | 6.0 | 3.62 |
| ENSTGUG00000004724 | CISH | 0.003 | 0.000 | 24.0 | 18.0 | 3.21 |
| ENSTGUG00000008426 | SAP30BP | 0.003 | 0.000 | 24.0 | 16.0 | 3.21 |
| ENSTGUG00000006853 | TRPV2 | 0.003 | 0.000 | 24.0 | 10.0 | 3.03 |
| ENSTGUG00000014228 |  | 0.003 | 0.000 | 24.0 | 4.0 | 2.84 |
| ENSTGUG00000006776 | BRD9 | 0.003 | 0.000 | 24.0 | 18.0 | 2.73 |
| ENSTGUG00000011744 | FAM98B | 0.003 | 0.000 | 24.0 | 16.0 | 2.70 |
| ENSTGUG00000002297 | MSL1 | 0.003 | 0.000 | 24.0 | 22.0 | 2.68 |
| ENSTGUG00000004486 | C9orf78 | 0.003 | 0.000 | 24.0 | 16.0 | 2.52 |
| ENSTGUG00000011619 | IL7 | 0.003 | 0.000 | 24.0 | 4.0 | 2.25 |
| ENSTGUG00000010770 | FAM59A | 0.003 | 0.000 | 24.0 | 4.0 | 2.21 |
| ENSTGUG00000002055 |  | 0.003 | 0.000 | 24.0 | 6.0 | 2.21 |
| ENSTGUG00000011650 | ADCK2 | 0.003 | 0.000 | 24.0 | 0.0 | 2.01 |
| ENSTGUG00000015434 | SMYD5 | 0.003 | 0.000 | 24.0 | 12.0 | 1.88 |
| ENSTGUG00000000360 | MLLT1 | 0.003 | 0.000 | 24.0 | 4.0 | 1.87 |
| ENSTGUG00000005118 | CPNE2 | 0.003 | 0.000 | 24.0 | 16.0 | 1.85 |
| ENSTGUG00000010636 |  | 0.003 | 0.000 | 24.0 | 18.0 | 1.82 |
| ENSTGUG00000008113 |  | 0.003 | 0.000 | 24.0 | 16.0 | 1.64 |
| ENSTGUG00000012039 | SIP1 | 0.003 | 0.000 | 24.0 | 22.0 | 1.62 |
| ENSTGUG00000005968 | COG7 | 0.003 | 0.000 | 24.0 | 16.0 | 1.42 |
| ENSTGUG00000008328 | ST5 | 0.003 | 0.000 | 24.0 | 6.0 | 1.39 |
| ENSTGUG00000015142 | BTK | 0.003 | 0.000 | 24.0 | 18.0 | 1.37 |
| ENSTGUG00000009211 | DCUN1D3 | 0.003 | 0.000 | 24.0 | 6.0 | 1.29 |
| ENSTGUG00000010474 | C2orf60 | 0.003 | 0.000 | 24.0 | 16.0 | 1.29 |
| ENSTGUG00000003564 | MAFG | 0.003 | 0.000 | 24.0 | 14.0 | 1.28 |
| ENSTGUG00000006301 | MBOAT1 | 0.003 | 0.000 | 24.0 | 16.0 | 1.26 |
| ENSTGUG00000013526 | GJA5 | 0.003 | 0.000 | 24.0 | 6.0 | 1.21 |
| ENSTGUG00000001987 |  | 0.003 | 0.000 | 24.0 | 8.0 | 1.15 |
| ENSTGUG00000004614 | WWOX | 0.003 | 0.000 | 24.0 | 8.0 | 1.11 |
| ENSTGUG00000008683 | PAQR9 | 0.003 | 0.000 | 24.0 | 8.0 | 1.05 |
| ENSTGUG00000007121 | FAM151B | 0.003 | 0.000 | 24.0 | 20.0 | 1.04 |
| ENSTGUG00000011216 | STAB2 | 0.003 | 0.000 | 24.0 | 16.0 | 1.00 |
| ENSTGUG00000010083 | MKL1 | 0.003 | 0.000 | 24.0 | 12.0 | 0.99 |
| ENSTGUG00000006653 | ENPP6 | 0.003 | 0.000 | 24.0 | 8.0 | 0.98 |
| ENSTGUG00000010781 | NAALADL2-1 | 0.003 | 0.000 | 24.0 | 22.0 | 0.96 |
| ENSTGUG00000000936 | PI16 | 0.003 | 0.000 | 24.0 | 4.0 | 0.95 |
| ENSTGUG00000011685 | SNX16 | 0.003 | 0.000 | 24.0 | 4.0 | 0.91 |
| ENSTGUG00000006044 | PLTP | 0.003 | 0.000 | 24.0 | 16.0 | 0.91 |
| ENSTGUG00000001871 | AHCYL2 | 0.003 | 0.000 | 24.0 | 12.0 | 0.91 |
| ENSTGUG00000009082 | ABCC10 | 0.003 | 0.000 | 24.0 | 16.0 | 0.90 |
| ENSTGUG00000013057 | C14orf101 | 0.003 | 0.000 | 24.0 | 16.0 | 0.86 |
| ENSTGUG00000006012 | ADRBK1 | 0.003 | 0.000 | 24.0 | 12.0 | 0.86 |
| ENSTGUG00000011358 | PSTK | 0.003 | 0.000 | 24.0 | 18.0 | 0.85 |
| ENSTGUG00000004500 | TBC1D8B | 0.003 | 0.000 | 24.0 | 4.0 | 0.83 |
| ENSTGUG00000010404 | HEATR1 | 0.003 | 0.000 | 24.0 | 18.0 | 0.83 |
| ENSTGUG00000012250 | C14orf118 | 0.003 | 0.000 | 24.0 | 6.0 | 0.80 |
| ENSTGUG00000002607 | GPBP1 | 0.003 | 0.000 | 24.0 | 18.0 | 0.74 |
| ENSTGUG00000005387 | C7orf42 | 0.003 | 0.000 | 24.0 | 4.0 | 0.66 |
| ENSTGUG00000004616 | STYXL1 | 0.003 | 0.000 | 24.0 | 18.0 | 0.62 |
| ENSTGUG00000015670 |  | 0.003 | 0.000 | 24.0 | 8.0 | 0.62 |
| ENSTGUG00000008339 | DET1 | 0.003 | 0.000 | 24.0 | 14.0 | 0.60 |
| ENSTGUG00000010972 |  | 0.003 | 0.000 | 24.0 | 18.0 | 0.60 |
| ENSTGUG00000009789 | KANK4 | 0.003 | 0.000 | 24.0 | 0.0 | 0.60 |
| ENSTGUG00000016188 | PFKM-3 | 0.003 | 0.000 | 24.0 | 8.0 | 0.60 |
| ENSTGUG00000007192 | MED8 | 0.003 | 0.000 | 24.0 | 16.0 | 0.56 |
| ENSTGUG00000001653 | RUFY3 | 0.003 | 0.000 | 24.0 | 14.0 | 0.55 |
| ENSTGUG00000010626 | SLBP | 0.003 | 0.000 | 24.0 | 14.0 | 0.53 |
| ENSTGUG00000010527 | C11orf49 | 0.003 | 0.000 | 24.0 | 18.0 | 0.46 |
| ENSTGUG00000011156 | B3GALNT1 | 0.003 | 0.000 | 24.0 | 18.0 | 0.44 |
| ENSTGUG00000008759 | LYSMD4 | 0.003 | 0.000 | 24.0 | 14.0 | 0.43 |
| ENSTGUG00000013885 | GATAD2A | 0.003 | 0.000 | 24.0 | 12.0 | 0.43 |
| ENSTGUG00000005559 | MSH6 | 0.003 | 0.000 | 24.0 | 16.0 | 0.41 |
| ENSTGUG00000000462 | SORL1 | 0.003 | 0.000 | 24.0 | 8.0 | 0.40 |
| ENSTGUG00000003275 | ANKFN1 | 0.003 | 0.000 | 24.0 | 6.0 | 0.37 |
| ENSTGUG00000011690 | KATNAL1 | 0.003 | 0.000 | 24.0 | 6.0 | 0.37 |
| ENSTGUG00000006943 | ATG10 | 0.003 | 0.000 | 24.0 | 16.0 | 0.34 |
| ENSTGUG00000003290 | CCNF | 0.003 | 0.000 | 24.0 | 14.0 | 0.24 |
| ENSTGUG00000013999 |  | 0.003 | 0.000 | 24.0 | 16.0 | 0.17 |
| ENSTGUG00000005922 | USP31 | 0.003 | 0.000 | 24.0 | 0.0 | 0.16 |
| ENSTGUG00000001174 | KIF21B | 0.003 | 0.000 | 24.0 | 18.0 | 0.12 |
| ENSTGUG00000004369 | DOCK5 | 0.003 | 0.000 | 24.0 | 18.0 | 0.09 |
| ENSTGUG00000002598 | TAS1R1 | 0.003 | 0.000 | 24.0 | 18.0 | 0.06 |
| ENSTGUG00000008841 | TPH1 | 0.003 | 0.000 | 24.0 | 18.0 | 0.03 |
| ENSTGUG00000002235 |  | 0.029 | 0.000 | 24.0 | 6.0 | 20.88 |
| ENSTGUG00000004468 |  | 0.029 | 0.000 | 24.0 | 2.0 | 5.01 |
| ENSTGUG00000004368 | HSD3B7 | 0.029 | 0.000 | 24.0 | 0.0 | 0.99 |
| ENSTGUG00000003194 | TWIST2 | 0.029 | 0.000 | 24.0 | 8.0 | 0.87 |
| ENSTGUG00000015275 |  | 0.029 | 0.000 | 24.0 | 8.0 | 0.82 |
| ENSTGUG00000017021 |  | 0.029 | 0.000 | 24.0 | 8.0 | 0.76 |
| ENSTGUG00000009529 | 10-Sep | 0.029 | 0.000 | 24.0 | 2.0 | 0.63 |
| ENSTGUG00000002896 | LPHN3 | 0.029 | 0.000 | 24.0 | 8.0 | 0.50 |
| ENSTGUG00000014319 |  | 0.029 | 0.000 | 24.0 | 16.0 | 0.45 |
| ENSTGUG00000002542 | MEOX2 | 0.029 | 0.000 | 24.0 | 6.0 | 0.36 |
| ENSTGUG00000015851 |  | 0.029 | 0.000 | 24.0 | 16.0 | 0.33 |
| ENSTGUG00000011613 | PKIA | 0.029 | 0.000 | 24.0 | 8.0 | 0.25 |
| ENSTGUG00000013488 | ZNF654 | 0.029 | 0.000 | 24.0 | 2.0 | 0.25 |
| ENSTGUG00000015584 |  | 0.029 | 0.000 | 24.0 | 2.0 | 0.24 |
| ENSTGUG00000012330 | ISM2 | 0.029 | 0.000 | 24.0 | 6.0 | 0.22 |
| ENSTGUG00000010313 |  | 0.029 | 0.000 | 24.0 | 8.0 | 0.19 |
| ENSTGUG00000011959 |  | 0.029 | 0.000 | 24.0 | 6.0 | 0.18 |
| ENSTGUG00000008867 | PIGZ | 0.029 | 0.000 | 24.0 | 18.0 | 0.18 |
| ENSTGUG00000014824 | C10orf137 | 0.029 | 0.000 | 24.0 | 2.0 | 0.16 |
| ENSTGUG00000014120 |  | 0.029 | 0.000 | 24.0 | 2.0 | 0.15 |
| ENSTGUG00000013808 |  | 0.029 | 0.000 | 24.0 | 18.0 | 0.14 |
| ENSTGUG00000011225 | VEPH1 | 0.029 | 0.000 | 24.0 | 18.0 | 0.13 |
| ENSTGUG00000003752 | WNT7A | 0.029 | 0.000 | 24.0 | 2.0 | 0.13 |
| ENSTGUG00000018412 |  | 0.035 | 0.002 | 26.0 | 2.0 | 2188.98 |
| ENSTGUG00000004240 | ELOVL6 | 0.035 | 0.002 | 26.0 | 2.0 | 1419.24 |
| ENSTGUG00000017273 | MR1 | 0.035 | 0.002 | 26.0 | 15.0 | 1362.90 |
| ENSTGUG00000006120 | LIPC | 0.035 | 0.002 | 26.0 | 2.0 | 892.56 |
| ENSTGUG00000007772 | CYP2C8-2 | 0.035 | 0.002 | 24.0 | 2.0 | 555.89 |
| ENSTGUG00000002713 | MOSC1 | 0.035 | 0.002 | 26.0 | 2.0 | 403.14 |
| ENSTGUG00000004140 | PSAP-1 | 0.035 | 0.002 | 26.0 | 2.0 | 317.55 |
| ENSTGUG00000010742 | ACSL5 | 0.035 | 0.002 | 24.0 | 6.0 | 282.81 |
| ENSTGUG00000012936 | MTHFD1 | 0.035 | 0.002 | 24.0 | 6.0 | 282.43 |
| ENSTGUG00000002252 | UGP2 | 0.035 | 0.002 | 25.3 | 4.0 | 252.36 |
| ENSTGUG00000008791 | SERPIND1 | 0.035 | 0.002 | 24.0 | 5.0 | 246.68 |
| ENSTGUG00000011435 | CP | 0.035 | 0.002 | 24.0 | 8.0 | 181.78 |
| ENSTGUG00000009671 |  | 0.035 | 0.002 | 24.0 | 2.0 | 148.71 |
| ENSTGUG00000013291 | CTSB | 0.035 | 0.002 | 26.0 | 15.0 | 144.81 |
| ENSTGUG00000003229 | DLD | 0.035 | 0.002 | 24.0 | 2.0 | 143.97 |
| ENSTGUG00000004911 | PPP1R3B | 0.035 | 0.002 | 26.0 | 2.0 | 142.71 |
| ENSTGUG00000008091 | MTHFS | 0.035 | 0.002 | 26.0 | 2.0 | 133.38 |
| ENSTGUG00000003183 |  | 0.035 | 0.002 | 26.0 | 15.0 | 125.26 |
| ENSTGUG00000000598 | CTSL1 | 0.035 | 0.002 | 24.0 | 18.0 | 122.50 |
| ENSTGUG00000013229 | PLA2G7 | 0.035 | 0.002 | 26.0 | 2.0 | 115.23 |
| ENSTGUG00000017729 |  | 0.035 | 0.002 | 24.0 | 6.0 | 111.75 |
| ENSTGUG00000010913 | A2LD1 | 0.035 | 0.002 | 24.0 | 6.0 | 109.28 |
| ENSTGUG00000009617 | C8B | 0.035 | 0.002 | 24.0 | 10.0 | 106.61 |
| ENSTGUG00000002619 | CD36 | 0.035 | 0.002 | 26.0 | 2.0 | 90.21 |
| ENSTGUG00000008939 | IGLL1 | 0.035 | 0.002 | 26.0 | 15.0 | 89.36 |
| ENSTGUG00000008207 | BTG1 | 0.035 | 0.002 | 24.0 | 8.0 | 82.29 |
| ENSTGUG00000012724 | CYP46A1 | 0.035 | 0.002 | 26.0 | 2.0 | 77.89 |
| ENSTGUG00000013087 | A2M-3 | 0.035 | 0.002 | 26.0 | 5.0 | 76.15 |
| ENSTGUG00000008513 | PDK1 | 0.035 | 0.002 | 26.0 | 2.0 | 72.44 |
| ENSTGUG00000003040 | HACL1 | 0.035 | 0.002 | 26.0 | 2.0 | 71.17 |
| ENSTGUG00000017237 | C1QB | 0.035 | 0.002 | 26.0 | 15.0 | 69.40 |
| ENSTGUG00000009653 | CIDEA | 0.035 | 0.002 | 26.0 | 2.0 | 63.37 |
| ENSTGUG00000000766 | C9orf95 | 0.035 | 0.002 | 26.0 | 2.0 | 63.26 |
| ENSTGUG00000014758 |  | 0.035 | 0.002 | 24.0 | 21.0 | 62.99 |
| ENSTGUG00000009841 | GNPAT | 0.035 | 0.002 | 25.3 | 4.0 | 58.28 |
| ENSTGUG00000002627 | MCFD2 | 0.035 | 0.002 | 24.0 | 6.0 | 55.17 |
| ENSTGUG00000003716 | ABCG8 | 0.035 | 0.002 | 24.0 | 1.0 | 52.58 |
| ENSTGUG00000005950 | FAM8A1 | 0.035 | 0.002 | 24.0 | 2.0 | 51.81 |
| ENSTGUG00000014114 | ALDH6A1 | 0.035 | 0.002 | 25.3 | 4.0 | 49.22 |
| ENSTGUG00000004398 | ACAD11-1 | 0.035 | 0.002 | 24.0 | 1.0 | 49.13 |
| ENSTGUG00000001437 | SARS | 0.035 | 0.002 | 24.0 | 14.0 | 44.68 |
| ENSTGUG00000013632 | NIT2 | 0.035 | 0.002 | 26.0 | 2.0 | 43.92 |
| ENSTGUG00000001661 | IGJ | 0.035 | 0.002 | 26.0 | 15.0 | 43.91 |
| ENSTGUG00000008960 |  | 0.035 | 0.002 | 24.0 | 14.0 | 42.91 |
| ENSTGUG00000011969 | SDC2 | 0.035 | 0.002 | 24.0 | 2.0 | 41.38 |
| ENSTGUG00000008167 | ACOX1 | 0.035 | 0.002 | 24.0 | 6.0 | 37.46 |
| ENSTGUG00000018072 |  | 0.035 | 0.002 | 24.0 | 6.0 | 36.89 |
| ENSTGUG00000006107 | STK25 | 0.035 | 0.002 | 24.0 | 8.0 | 35.76 |
| ENSTGUG00000003063 | PPP2R5A | 0.035 | 0.002 | 24.0 | 22.0 | 35.65 |
| ENSTGUG00000003551 |  | 0.035 | 0.002 | 25.3 | 16.7 | 35.22 |
| ENSTGUG00000016380 |  | 0.035 | 0.002 | 24.0 | 2.0 | 32.77 |
| ENSTGUG00000014435 | SERPINA12-3 | 0.035 | 0.002 | 24.0 | 2.0 | 32.74 |
| ENSTGUG00000018695 |  | 0.035 | 0.002 | 26.0 | 2.0 | 32.71 |
| ENSTGUG00000002691 | ZMYND19 | 0.035 | 0.002 | 24.0 | 13.0 | 32.27 |
| ENSTGUG00000017768 |  | 0.035 | 0.002 | 24.0 | 14.0 | 30.45 |
| ENSTGUG00000011445 | ERH | 0.035 | 0.002 | 26.0 | 15.0 | 28.37 |
| ENSTGUG00000007243 | EFHD1 | 0.035 | 0.002 | 24.0 | 6.0 | 28.26 |
| ENSTGUG00000013958 |  | 0.035 | 0.002 | 24.0 | 14.0 | 27.31 |
| ENSTGUG00000008505 | UFD1L | 0.035 | 0.002 | 24.0 | 4.0 | 26.92 |
| ENSTGUG00000009941 | TOB2 | 0.035 | 0.002 | 25.3 | 4.0 | 26.59 |
| ENSTGUG00000000402 | ACAA1 | 0.035 | 0.002 | 24.0 | 8.0 | 26.10 |
| ENSTGUG00000015205 |  | 0.035 | 0.002 | 24.0 | 5.0 | 25.56 |
| ENSTGUG00000003051 | FAM13A1 | 0.035 | 0.002 | 24.0 | 2.0 | 24.95 |
| ENSTGUG00000000084 | RAB11B | 0.035 | 0.002 | 24.0 | 6.0 | 24.66 |
| ENSTGUG00000008222 | RAB9A | 0.035 | 0.002 | 24.0 | 2.0 | 23.42 |
| ENSTGUG00000018089 |  | 0.035 | 0.002 | 26.0 | 15.0 | 23.24 |
| ENSTGUG00000009169 | CPN1 | 0.035 | 0.002 | 26.0 | 15.0 | 23.02 |
| ENSTGUG00000007363 | RASD1 | 0.035 | 0.002 | 24.0 | 14.0 | 22.93 |
| ENSTGUG00000010329 | LRPAP1 | 0.035 | 0.002 | 24.0 | 5.0 | 22.91 |
| ENSTGUG00000010316 | EDEM1 | 0.035 | 0.002 | 24.0 | 8.0 | 22.19 |
| ENSTGUG00000008728 | RNF114 | 0.035 | 0.002 | 25.3 | 16.7 | 21.92 |
| ENSTGUG00000013103 | PSMC6 | 0.035 | 0.002 | 24.0 | 20.0 | 21.91 |
| ENSTGUG00000012128 | AZIN1 | 0.035 | 0.002 | 26.0 | 2.0 | 21.59 |
| ENSTGUG00000014660 |  | 0.035 | 0.002 | 24.0 | 8.0 | 21.37 |
| ENSTGUG00000005585 | HCCS | 0.035 | 0.002 | 24.0 | 18.0 | 20.74 |
| ENSTGUG00000012035 | MCAT | 0.035 | 0.002 | 24.0 | 5.0 | 20.12 |
| ENSTGUG00000011176 | CA8 | 0.035 | 0.002 | 24.0 | 2.0 | 20.09 |
| ENSTGUG00000010156 | API5 | 0.035 | 0.002 | 26.0 | 2.0 | 19.22 |
| ENSTGUG00000005684 | SNRPB | 0.035 | 0.002 | 26.0 | 15.0 | 18.95 |
| ENSTGUG00000004652 | PMM2 | 0.035 | 0.002 | 24.0 | 5.0 | 18.21 |
| ENSTGUG00000006667 | PQLC1 | 0.035 | 0.002 | 26.0 | 2.0 | 17.65 |
| ENSTGUG00000001518 |  | 0.035 | 0.002 | 24.0 | 8.0 | 17.51 |
| ENSTGUG00000011494 | ZFP36L1 | 0.035 | 0.002 | 24.0 | 10.0 | 16.97 |
| ENSTGUG00000012106 | GYS2 | 0.035 | 0.002 | 24.0 | 2.0 | 16.93 |
| ENSTGUG00000001269 | SEC24A | 0.035 | 0.002 | 24.0 | 22.0 | 16.16 |
| ENSTGUG00000008006 | FAM108C1 | 0.035 | 0.002 | 24.0 | 6.0 | 15.99 |
| ENSTGUG00000003599 | LZIC | 0.035 | 0.002 | 24.0 | 18.0 | 15.73 |
| ENSTGUG00000004272 | ACSS2 | 0.035 | 0.002 | 24.0 | 6.0 | 15.67 |
| ENSTGUG00000012916 | FZD4 | 0.035 | 0.002 | 24.0 | 5.0 | 15.55 |
| ENSTGUG00000007404 | ANKH | 0.035 | 0.002 | 24.0 | 14.0 | 15.27 |
| ENSTGUG00000001456 | RAP1A | 0.035 | 0.002 | 24.0 | 4.0 | 14.69 |
| ENSTGUG00000008667 | LONP2 | 0.035 | 0.002 | 24.0 | 2.0 | 14.18 |
| ENSTGUG00000005595 | DNAJB9 | 0.035 | 0.002 | 26.0 | 2.0 | 14.14 |
| ENSTGUG00000013013 | KCTD21 | 0.035 | 0.002 | 24.0 | 2.0 | 13.92 |
| ENSTGUG00000010110 | TMEM180 | 0.035 | 0.002 | 24.0 | 6.0 | 13.89 |
| ENSTGUG00000009176 | CPT2 | 0.035 | 0.002 | 24.0 | 4.0 | 13.84 |
| ENSTGUG00000004208 | TJP2 | 0.035 | 0.002 | 26.0 | 2.0 | 13.77 |
| ENSTGUG00000003632 | STT3B | 0.035 | 0.002 | 24.0 | 6.0 | 13.71 |
| ENSTGUG00000008137 | VEGFA | 0.035 | 0.002 | 24.0 | 6.0 | 13.39 |
| ENSTGUG00000002761 | ARHGAP24 | 0.035 | 0.002 | 24.0 | 2.0 | 13.19 |
| ENSTGUG00000005150 |  | 0.035 | 0.002 | 24.0 | 20.0 | 13.15 |
| ENSTGUG00000006220 | ST3GAL2 | 0.035 | 0.002 | 24.0 | 8.0 | 13.05 |
| ENSTGUG00000010838 | RMND5A | 0.035 | 0.002 | 24.0 | 0.0 | 13.03 |
| ENSTGUG00000013269 | LPCAT3 | 0.035 | 0.002 | 24.0 | 20.0 | 12.79 |
| ENSTGUG00000006908 | PNPT1 | 0.035 | 0.002 | 24.0 | 2.0 | 12.70 |
| ENSTGUG00000005284 | TTL | 0.035 | 0.002 | 26.0 | 2.0 | 12.59 |
| ENSTGUG00000016091 |  | 0.035 | 0.002 | 24.0 | 14.0 | 12.56 |
| ENSTGUG00000012746 | EVL | 0.035 | 0.002 | 25.3 | 16.7 | 12.45 |
| ENSTGUG00000006428 | CYR61 | 0.035 | 0.002 | 26.0 | 15.0 | 12.40 |
| ENSTGUG00000004801 | HDAC8 | 0.035 | 0.002 | 26.0 | 15.0 | 12.36 |
| ENSTGUG00000007259 | PPP6C | 0.035 | 0.002 | 26.0 | 15.0 | 12.23 |
| ENSTGUG00000013795 |  | 0.035 | 0.002 | 24.0 | 10.0 | 12.21 |
| ENSTGUG00000005261 | TMBIM1 | 0.035 | 0.002 | 24.0 | 14.0 | 11.75 |
| ENSTGUG00000005611 | ALG14 | 0.035 | 0.002 | 24.0 | 20.0 | 11.17 |
| ENSTGUG00000012398 | KIAA0564 | 0.035 | 0.002 | 24.0 | 2.0 | 11.14 |
| ENSTGUG00000002086 | CCDC103 | 0.035 | 0.002 | 26.0 | 15.0 | 11.03 |
| ENSTGUG00000010724 | MYO1B | 0.035 | 0.002 | 24.0 | 2.0 | 10.97 |
| ENSTGUG00000008783 | ICT1 | 0.035 | 0.002 | 24.0 | 18.0 | 10.89 |
| ENSTGUG00000005495 | DPYD | 0.035 | 0.002 | 24.0 | 4.0 | 10.52 |
| ENSTGUG00000013789 | NT5C3L | 0.035 | 0.002 | 24.0 | 16.0 | 10.39 |
| ENSTGUG00000011668 | IMPA1 | 0.035 | 0.002 | 24.0 | 2.0 | 10.32 |
| ENSTGUG00000000761 | OSTF1 | 0.035 | 0.002 | 24.0 | 2.0 | 10.21 |
| ENSTGUG00000016851 |  | 0.035 | 0.002 | 26.0 | 2.0 | 10.19 |
| ENSTGUG00000010754 | RAB32 | 0.035 | 0.002 | 24.0 | 2.0 | 10.13 |
| ENSTGUG00000003005 | NFS1 | 0.035 | 0.002 | 24.0 | 2.0 | 10.06 |
| ENSTGUG00000007976 | COBL | 0.035 | 0.002 | 24.0 | 2.0 | 10.00 |
| ENSTGUG00000009722 | CYP2J2-1 | 0.035 | 0.002 | 24.0 | 21.0 | 9.95 |
| ENSTGUG00000006956 | CORO1C | 0.035 | 0.002 | 26.0 | 15.0 | 9.93 |
| ENSTGUG00000002295 | DPP7 | 0.035 | 0.002 | 24.0 | 18.0 | 9.91 |
| ENSTGUG00000015876 | TUBB2A-5-pseudogene | 0.035 | 0.002 | 24.0 | 10.0 | 9.84 |
| ENSTGUG00000017327 | B5G325_TAEGU | 0.035 | 0.002 | 26.0 | 15.0 | 9.68 |
| ENSTGUG00000004185 | GAR1 | 0.035 | 0.002 | 24.0 | 18.0 | 9.40 |
| ENSTGUG00000000113 | NANS | 0.035 | 0.002 | 26.0 | 2.0 | 9.35 |
| ENSTGUG00000003058 | BCL7B | 0.035 | 0.002 | 24.0 | 14.0 | 9.34 |
| ENSTGUG00000004021 | AGGF1 | 0.035 | 0.002 | 25.3 | 4.0 | 9.28 |
| ENSTGUG00000007810 | NLRP12 | 0.035 | 0.002 | 25.3 | 16.7 | 9.14 |
| ENSTGUG00000000278 | MKNK2 | 0.035 | 0.002 | 25.3 | 4.0 | 9.13 |
| ENSTGUG00000009728 | ABI2 | 0.035 | 0.002 | 26.0 | 15.0 | 9.13 |
| ENSTGUG00000002740 | KLHL7 | 0.035 | 0.002 | 24.0 | 2.0 | 9.03 |
| ENSTGUG00000017313 | PHPT1-2 | 0.035 | 0.002 | 24.0 | 18.0 | 8.94 |
| ENSTGUG00000013608 | HHLA2 | 0.035 | 0.002 | 24.0 | 10.0 | 8.82 |
| ENSTGUG00000006807 | DPP4 | 0.035 | 0.002 | 24.0 | 6.0 | 8.63 |
| ENSTGUG00000011022 | LTV1 | 0.035 | 0.002 | 24.0 | 18.0 | 8.50 |
| ENSTGUG00000007537 |  | 0.035 | 0.002 | 24.0 | 6.0 | 8.44 |
| ENSTGUG00000000134 | YIPF5 | 0.035 | 0.002 | 24.0 | 12.0 | 8.39 |
| ENSTGUG00000012034 | BXDC1 | 0.035 | 0.002 | 26.0 | 15.0 | 8.38 |
| ENSTGUG00000002608 | POLE3 | 0.035 | 0.002 | 24.0 | 20.0 | 8.35 |
| ENSTGUG00000009767 | CENPM | 0.035 | 0.002 | 24.0 | 8.0 | 8.31 |
| ENSTGUG00000014589 |  | 0.035 | 0.002 | 24.0 | 6.0 | 8.16 |
| ENSTGUG00000017034 | DHPS | 0.035 | 0.002 | 26.0 | 15.0 | 8.07 |
| ENSTGUG00000001517 | GNG11 | 0.035 | 0.002 | 24.0 | 14.0 | 7.92 |
| ENSTGUG00000013592 | C3orf52 | 0.035 | 0.002 | 24.0 | 2.0 | 7.85 |
| ENSTGUG00000010427 | NPC1 | 0.035 | 0.002 | 24.0 | 1.0 | 7.83 |
| ENSTGUG00000003228 | IL13RA1 | 0.035 | 0.002 | 24.0 | 2.0 | 7.79 |
| ENSTGUG00000011089 | C10orf46 | 0.035 | 0.002 | 26.0 | 2.0 | 7.76 |
| ENSTGUG00000008226 | DYNC1I2 | 0.035 | 0.002 | 24.0 | 21.0 | 7.75 |
| ENSTGUG00000007821 | TALDO1 | 0.035 | 0.002 | 25.3 | 16.7 | 7.65 |
| ENSTGUG00000012651 |  | 0.035 | 0.002 | 24.0 | 20.0 | 7.59 |
| ENSTGUG00000008570 | TBC1D12 | 0.035 | 0.002 | 26.0 | 2.0 | 7.38 |
| ENSTGUG00000003731 | SLC25A16 | 0.035 | 0.002 | 25.3 | 16.7 | 7.37 |
| ENSTGUG00000011978 |  | 0.035 | 0.002 | 24.0 | 6.0 | 7.33 |
| ENSTGUG00000001860 | VPS41 | 0.035 | 0.002 | 24.0 | 2.0 | 7.32 |
| ENSTGUG00000004736 | LIN7C | 0.035 | 0.002 | 24.0 | 18.0 | 7.31 |
| ENSTGUG00000008036 | C18orf21 | 0.035 | 0.002 | 24.0 | 18.0 | 7.27 |
| ENSTGUG00000004415 | OIT3 | 0.035 | 0.002 | 24.0 | 13.0 | 7.22 |
| ENSTGUG00000006642 | KIAA1370 | 0.035 | 0.002 | 24.0 | 1.0 | 7.21 |
| ENSTGUG00000007610 | PTRH2 | 0.035 | 0.002 | 24.0 | 14.0 | 7.16 |
| ENSTGUG00000002187 | FBXL20 | 0.035 | 0.002 | 24.0 | 8.0 | 7.09 |
| ENSTGUG00000003813 | EDEM2 | 0.035 | 0.002 | 24.0 | 8.0 | 7.07 |
| ENSTGUG00000003894 | GFPT1 | 0.035 | 0.002 | 24.0 | 12.0 | 7.05 |
| ENSTGUG00000014642 | ZNF512 | 0.035 | 0.002 | 25.3 | 4.0 | 6.98 |
| ENSTGUG00000005128 | KIAA0922 | 0.035 | 0.002 | 24.0 | 0.0 | 6.91 |
| ENSTGUG00000008851 | ING1 | 0.035 | 0.002 | 26.0 | 2.0 | 6.90 |
| ENSTGUG00000016564 |  | 0.035 | 0.002 | 24.0 | 4.0 | 6.89 |
| ENSTGUG00000000446 | CKS2 | 0.035 | 0.002 | 24.0 | 14.0 | 6.74 |
| ENSTGUG00000010255 | STRADB | 0.035 | 0.002 | 26.0 | 2.0 | 6.64 |
| ENSTGUG00000002313 | MAN1B1 | 0.035 | 0.002 | 24.0 | 6.0 | 6.59 |
| ENSTGUG00000009065 |  | 0.035 | 0.002 | 24.0 | 2.0 | 6.57 |
| ENSTGUG00000005952 | SARM1 | 0.035 | 0.002 | 24.0 | 2.0 | 6.47 |
| ENSTGUG00000001024 | HMGN2 | 0.035 | 0.002 | 24.0 | 17.0 | 6.47 |
| ENSTGUG00000011639 | AVEN | 0.035 | 0.002 | 24.0 | 18.0 | 6.45 |
| ENSTGUG00000007886 | FAM21C | 0.035 | 0.002 | 24.0 | 2.0 | 6.39 |
| ENSTGUG00000006561 | SLC9A3R2 | 0.035 | 0.002 | 24.0 | 9.0 | 6.38 |
| ENSTGUG00000003755 | YTHDC2 | 0.035 | 0.002 | 24.0 | 2.0 | 6.33 |
| ENSTGUG00000009385 | DDX47 | 0.035 | 0.002 | 24.0 | 18.0 | 6.32 |
| ENSTGUG00000012817 | CWC15 | 0.035 | 0.002 | 24.0 | 20.0 | 6.31 |
| ENSTGUG00000002329 | DIAPH2 | 0.035 | 0.002 | 26.0 | 2.0 | 6.31 |
| ENSTGUG00000010047 | CHST10 | 0.035 | 0.002 | 24.0 | 4.0 | 6.25 |
| ENSTGUG00000001603 | STK40 | 0.035 | 0.002 | 24.0 | 6.0 | 6.08 |
| ENSTGUG00000003092 | NDRG3 | 0.035 | 0.002 | 24.0 | 6.0 | 5.94 |
| ENSTGUG00000008534 | MRPL40 | 0.035 | 0.002 | 24.0 | 18.0 | 5.92 |
| ENSTGUG00000011923 | SNX6 | 0.035 | 0.002 | 24.0 | 0.0 | 5.91 |
| ENSTGUG00000005887 | PAK1IP1 | 0.035 | 0.002 | 24.0 | 18.0 | 5.87 |
| ENSTGUG00000008402 | ADAP1 | 0.035 | 0.002 | 24.0 | 8.0 | 5.79 |
| ENSTGUG00000000675 | MED7 | 0.035 | 0.002 | 26.0 | 15.0 | 5.60 |
| ENSTGUG00000016622 | QPCTL | 0.035 | 0.002 | 24.0 | 14.0 | 5.58 |
| ENSTGUG00000015928 |  | 0.035 | 0.002 | 24.0 | 5.0 | 5.52 |
| ENSTGUG00000002146 | FAM175A | 0.035 | 0.002 | 24.0 | 18.0 | 5.46 |
| ENSTGUG00000011869 | PPM2C | 0.035 | 0.002 | 26.0 | 2.0 | 5.38 |
| ENSTGUG00000010877 | EIF2AK3 | 0.035 | 0.002 | 24.0 | 4.0 | 5.37 |
| ENSTGUG00000004244 | ENPEP | 0.035 | 0.002 | 24.0 | 4.0 | 5.36 |
| ENSTGUG00000015459 | SFXN5 | 0.035 | 0.002 | 24.0 | 14.0 | 5.27 |
| ENSTGUG00000008726 | PDS5A | 0.035 | 0.002 | 26.0 | 2.0 | 5.25 |
| ENSTGUG00000000564 | NUB1 | 0.035 | 0.002 | 25.3 | 16.7 | 5.21 |
| ENSTGUG00000013585 | BACH1 | 0.035 | 0.002 | 24.0 | 2.0 | 5.15 |
| ENSTGUG00000017235 |  | 0.035 | 0.002 | 24.0 | 8.0 | 4.80 |
| ENSTGUG00000009481 | C18orf1 | 0.035 | 0.002 | 25.3 | 16.7 | 4.79 |
| ENSTGUG00000016379 |  | 0.035 | 0.002 | 26.0 | 2.0 | 4.75 |
| ENSTGUG00000000379 | MCAM | 0.035 | 0.002 | 24.0 | 13.0 | 4.73 |
| ENSTGUG00000003806 | SLC3A1 | 0.035 | 0.002 | 26.0 | 15.0 | 4.58 |
| ENSTGUG00000008940 | SULF2 | 0.035 | 0.002 | 24.0 | 1.0 | 4.55 |
| ENSTGUG00000006043 | FAM63B | 0.035 | 0.002 | 26.0 | 2.0 | 4.50 |
| ENSTGUG00000012826 | CDC42BPB | 0.035 | 0.002 | 26.0 | 2.0 | 4.49 |
| ENSTGUG00000016219 |  | 0.035 | 0.002 | 24.0 | 6.0 | 4.46 |
| ENSTGUG00000003938 |  | 0.035 | 0.002 | 24.0 | 9.0 | 4.34 |
| ENSTGUG00000006242 | CGNL1 | 0.035 | 0.002 | 26.0 | 2.0 | 4.27 |
| ENSTGUG00000016884 |  | 0.035 | 0.002 | 24.0 | 22.0 | 4.26 |
| ENSTGUG00000007721 | CYBA | 0.035 | 0.002 | 26.0 | 15.0 | 4.25 |
| ENSTGUG00000014269 |  | 0.035 | 0.002 | 24.0 | 20.0 | 4.25 |
| ENSTGUG00000006439 | PRRG3 | 0.035 | 0.002 | 24.0 | 18.0 | 4.24 |
| ENSTGUG00000006260 | TGM2 | 0.035 | 0.002 | 24.0 | 14.0 | 4.22 |
| ENSTGUG00000002448 | ARL15 | 0.035 | 0.002 | 24.0 | 18.0 | 4.22 |
| ENSTGUG00000008117 | ASB9 | 0.035 | 0.002 | 24.0 | 4.0 | 4.21 |
| ENSTGUG00000009768 | BST1 | 0.035 | 0.002 | 24.0 | 4.0 | 4.20 |
| ENSTGUG00000010077 | SEMA4G | 0.035 | 0.002 | 24.0 | 8.0 | 4.12 |
| ENSTGUG00000000413 | DDX41 | 0.035 | 0.002 | 24.0 | 14.0 | 4.11 |
| ENSTGUG00000000692 | LYSMD3 | 0.035 | 0.002 | 26.0 | 2.0 | 4.11 |
| ENSTGUG00000009560 | MAGI1 | 0.035 | 0.002 | 24.0 | 2.0 | 4.11 |
| ENSTGUG00000007341 | SLMAP | 0.035 | 0.002 | 24.0 | 2.0 | 4.10 |
| ENSTGUG00000006560 | CPD | 0.035 | 0.002 | 24.0 | 6.0 | 4.09 |
| ENSTGUG00000005858 | P2RX4 | 0.035 | 0.002 | 24.0 | 13.0 | 4.09 |
| ENSTGUG00000012802 | DST | 0.035 | 0.002 | 26.0 | 2.0 | 3.96 |
| ENSTGUG00000009915 | CHKA | 0.035 | 0.002 | 24.0 | 22.0 | 3.95 |
| ENSTGUG00000007440 | TRPM7-2 | 0.035 | 0.002 | 24.0 | 2.0 | 3.93 |
| ENSTGUG00000002720 | C1orf174 | 0.035 | 0.002 | 26.0 | 18.0 | 3.91 |
| ENSTGUG00000017168 |  | 0.035 | 0.002 | 24.0 | 16.0 | 3.84 |
| ENSTGUG00000013077 | LGALS3 | 0.035 | 0.002 | 24.0 | 20.0 | 3.81 |
| ENSTGUG00000014619 |  | 0.035 | 0.002 | 24.0 | 6.0 | 3.80 |
| ENSTGUG00000010719 | RABL4 | 0.035 | 0.002 | 24.0 | 18.0 | 3.73 |
| ENSTGUG00000006209 | C4orf27 | 0.035 | 0.002 | 24.0 | 16.0 | 3.72 |
| ENSTGUG00000004538 | STXBP3 | 0.035 | 0.002 | 24.0 | 1.0 | 3.70 |
| ENSTGUG00000002208 | PRPF4B | 0.035 | 0.002 | 26.0 | 2.0 | 3.67 |
| ENSTGUG00000016783 |  | 0.035 | 0.002 | 24.0 | 6.0 | 3.54 |
| ENSTGUG00000010381 | THPO | 0.035 | 0.002 | 24.0 | 12.0 | 3.53 |
| ENSTGUG00000013412 | CLDND1 | 0.035 | 0.002 | 24.0 | 13.0 | 3.52 |
| ENSTGUG00000005361 | NDE1 | 0.035 | 0.002 | 24.0 | 6.0 | 3.51 |
| ENSTGUG00000006181 | DIABLO | 0.035 | 0.002 | 24.0 | 14.0 | 3.49 |
| ENSTGUG00000013132 | GNG2 | 0.035 | 0.002 | 25.3 | 16.7 | 3.45 |
| ENSTGUG00000009642 | MMS19 | 0.035 | 0.002 | 26.0 | 15.0 | 3.44 |
| ENSTGUG00000000287 | LPL | 0.035 | 0.002 | 24.0 | 21.0 | 3.43 |
| ENSTGUG00000006874 | SLC7A6 | 0.035 | 0.002 | 24.0 | 2.0 | 3.39 |
| ENSTGUG00000005216 | PLRG1 | 0.035 | 0.002 | 24.0 | 20.0 | 3.37 |
| ENSTGUG00000006095 | WDR82 | 0.035 | 0.002 | 24.0 | 13.0 | 3.36 |
| ENSTGUG00000001052 | PFKFB2 | 0.035 | 0.002 | 24.0 | 6.0 | 3.35 |
| ENSTGUG00000012589 | HMGN3 | 0.035 | 0.002 | 24.0 | 20.0 | 3.28 |
| ENSTGUG00000006847 | TMOD3 | 0.035 | 0.002 | 26.0 | 2.0 | 3.28 |
| ENSTGUG00000004961 | EXOSC7 | 0.035 | 0.002 | 26.0 | 15.0 | 3.28 |
| ENSTGUG00000008857 | ARFGEF2 | 0.035 | 0.002 | 24.0 | 2.0 | 3.25 |
| ENSTGUG00000013250 | SUPT3H | 0.035 | 0.002 | 26.0 | 2.0 | 3.23 |
| ENSTGUG00000012539 | LMO7 | 0.035 | 0.002 | 26.0 | 2.0 | 3.17 |
| ENSTGUG00000005711 | ANAPC7 | 0.035 | 0.002 | 26.0 | 15.0 | 3.12 |
| ENSTGUG00000007641 | KLHDC4 | 0.035 | 0.002 | 24.0 | 14.0 | 3.06 |
| ENSTGUG00000004363 | NUDT13 | 0.035 | 0.002 | 24.0 | 17.0 | 3.04 |
| ENSTGUG00000016177 |  | 0.035 | 0.002 | 25.3 | 4.0 | 2.99 |
| ENSTGUG00000002184 | TUBB2A-1 | 0.035 | 0.002 | 24.0 | 9.0 | 2.95 |
| ENSTGUG00000012435 | ATAD2 | 0.035 | 0.002 | 24.0 | 5.0 | 2.93 |
| ENSTGUG00000006936 | MTCP1 | 0.035 | 0.002 | 24.0 | 8.0 | 2.93 |
| ENSTGUG00000000480 | TMEM38A | 0.035 | 0.002 | 24.0 | 8.0 | 2.93 |
| ENSTGUG00000009445 | ACOT11 | 0.035 | 0.002 | 24.0 | 14.0 | 2.87 |
| ENSTGUG00000000866 | RPA2 | 0.035 | 0.002 | 24.0 | 17.0 | 2.82 |
| ENSTGUG00000004338 | AXIN1 | 0.035 | 0.002 | 24.0 | 6.0 | 2.81 |
| ENSTGUG00000007373 | GLIPR1-1 | 0.035 | 0.002 | 26.0 | 18.0 | 2.80 |
| ENSTGUG00000004161 | NAT15 | 0.035 | 0.002 | 24.0 | 18.0 | 2.78 |
| ENSTGUG00000015021 |  | 0.035 | 0.002 | 25.3 | 16.7 | 2.75 |
| ENSTGUG00000012305 | C14orf133 | 0.035 | 0.002 | 24.0 | 2.0 | 2.75 |
| ENSTGUG00000003237 | RNPS1 | 0.035 | 0.002 | 26.0 | 15.0 | 2.73 |
| ENSTGUG00000007901 | CHMP1A | 0.035 | 0.002 | 24.0 | 14.0 | 2.71 |
| ENSTGUG00000005267 | ENOX2 | 0.035 | 0.002 | 25.3 | 4.0 | 2.66 |
| ENSTGUG00000002870 | NLN | 0.035 | 0.002 | 25.3 | 4.0 | 2.62 |
| ENSTGUG00000012076 | ZNF410 | 0.035 | 0.002 | 24.0 | 14.0 | 2.61 |
| ENSTGUG00000005568 | SLC2A6 | 0.035 | 0.002 | 26.0 | 15.0 | 2.59 |
| ENSTGUG00000000807 | PUM1 | 0.035 | 0.002 | 24.0 | 14.0 | 2.58 |
| ENSTGUG00000014634 |  | 0.035 | 0.002 | 25.3 | 16.7 | 2.52 |
| ENSTGUG00000007728 |  | 0.035 | 0.002 | 25.3 | 16.7 | 2.51 |
| ENSTGUG00000004402 | C16orf35 | 0.035 | 0.002 | 24.0 | 16.0 | 2.49 |
| ENSTGUG00000010074 | LPIN2 | 0.035 | 0.002 | 26.0 | 2.0 | 2.47 |
| ENSTGUG00000009650 | GCC2 | 0.035 | 0.002 | 26.0 | 2.0 | 2.47 |
| ENSTGUG00000011126 | GRK5 | 0.035 | 0.002 | 24.0 | 2.0 | 2.44 |
| ENSTGUG00000002765 | ARMC10 | 0.035 | 0.002 | 24.0 | 0.0 | 2.43 |
| ENSTGUG00000002271 | MED24 | 0.035 | 0.002 | 24.0 | 10.0 | 2.43 |
| ENSTGUG00000012149 | SLC25A32 | 0.035 | 0.002 | 24.0 | 2.0 | 2.42 |
| ENSTGUG00000012917 | CDCA4 | 0.035 | 0.002 | 24.0 | 2.0 | 2.41 |
| ENSTGUG00000014065 | IFRD2 | 0.035 | 0.002 | 24.0 | 13.0 | 2.40 |
| ENSTGUG00000001748 | GALT | 0.035 | 0.002 | 24.0 | 14.0 | 2.40 |
| ENSTGUG00000011510 | YARS2 | 0.035 | 0.002 | 24.0 | 18.0 | 2.38 |
| ENSTGUG00000000245 | HOOK3 | 0.035 | 0.002 | 24.0 | 20.0 | 2.38 |
| ENSTGUG00000005762 | SMYD4 | 0.035 | 0.002 | 26.0 | 15.0 | 2.38 |
| ENSTGUG00000011482 | C12orf35 | 0.035 | 0.002 | 26.0 | 2.0 | 2.37 |
| ENSTGUG00000005873 | FBXO45 | 0.035 | 0.002 | 26.0 | 2.0 | 2.37 |
| ENSTGUG00000014501 |  | 0.035 | 0.002 | 24.0 | 12.0 | 2.34 |
| ENSTGUG00000008453 | GUF1 | 0.035 | 0.002 | 24.0 | 20.0 | 2.34 |
| ENSTGUG00000002387 | FBXO48 | 0.035 | 0.002 | 24.0 | 20.0 | 2.33 |
| ENSTGUG00000014636 | DNAJC18 | 0.035 | 0.002 | 24.0 | 8.0 | 2.32 |
| ENSTGUG00000016289 | REEP4 | 0.035 | 0.002 | 26.0 | 15.0 | 2.31 |
| ENSTGUG00000007013 | MAP3K7IP3 | 0.035 | 0.002 | 24.0 | 2.0 | 2.31 |
| ENSTGUG00000014495 | ERBB2 | 0.035 | 0.002 | 24.0 | 18.0 | 2.29 |
| ENSTGUG00000002969 | WIPF3 | 0.035 | 0.002 | 24.0 | 14.0 | 2.28 |
| ENSTGUG00000009744 | TSPAN4 | 0.035 | 0.002 | 24.0 | 13.0 | 2.24 |
| ENSTGUG00000012271 | C14orf4 | 0.035 | 0.002 | 24.0 | 12.0 | 2.21 |
| ENSTGUG00000011672 | ZFAND1 | 0.035 | 0.002 | 24.0 | 18.0 | 2.20 |
| ENSTGUG00000002010 | CETN1 | 0.035 | 0.002 | 26.0 | 2.0 | 2.17 |
| ENSTGUG00000017532 | ZFYVE16 | 0.035 | 0.002 | 24.0 | 5.0 | 2.16 |
| ENSTGUG00000010852 | MFSD6 | 0.035 | 0.002 | 24.0 | 4.0 | 2.16 |
| ENSTGUG00000009904 | ITGB3BP | 0.035 | 0.002 | 26.0 | 15.0 | 2.14 |
| ENSTGUG00000003192 | CPSF3L | 0.035 | 0.002 | 24.0 | 14.0 | 2.12 |
| ENSTGUG00000003250 | TRERF1 | 0.035 | 0.002 | 26.0 | 15.0 | 2.08 |
| ENSTGUG00000010000 | L3MBTL2 | 0.035 | 0.002 | 26.0 | 15.0 | 2.08 |
| ENSTGUG00000005412 | BNIP2 | 0.035 | 0.002 | 24.0 | 2.0 | 2.07 |
| ENSTGUG00000012892 | ANGPT2 | 0.035 | 0.002 | 24.0 | 6.0 | 2.03 |
| ENSTGUG00000012959 | PPP2R5E | 0.035 | 0.002 | 24.0 | 2.0 | 2.02 |
| ENSTGUG00000015747 | LRP1 | 0.035 | 0.002 | 24.0 | 8.0 | 2.00 |
| ENSTGUG00000005201 | MYH10 | 0.035 | 0.002 | 24.0 | 6.0 | 2.00 |
| ENSTGUG00000001633 | MAPK14 | 0.035 | 0.002 | 24.0 | 5.0 | 1.99 |
| ENSTGUG00000002108 | CD40LG | 0.035 | 0.002 | 26.0 | 15.0 | 1.90 |
| ENSTGUG00000017492 | RBBP5 | 0.035 | 0.002 | 24.0 | 9.0 | 1.88 |
| ENSTGUG00000012947 | SYNE2-1 | 0.035 | 0.002 | 26.0 | 2.0 | 1.87 |
| ENSTGUG00000015807 |  | 0.035 | 0.002 | 24.0 | 18.0 | 1.87 |
| ENSTGUG00000010421 |  | 0.035 | 0.002 | 24.0 | 8.0 | 1.86 |
| ENSTGUG00000010897 | MCM4 | 0.035 | 0.002 | 24.0 | 12.0 | 1.83 |
| ENSTGUG00000005400 | SLC2A10 | 0.035 | 0.002 | 24.0 | 14.0 | 1.82 |
| ENSTGUG00000008412 | 5-Sep | 0.035 | 0.002 | 24.0 | 22.0 | 1.82 |
| ENSTGUG00000003963 | TRABD | 0.035 | 0.002 | 26.0 | 2.0 | 1.80 |
| ENSTGUG00000008657 | ZAK | 0.035 | 0.002 | 24.0 | 22.0 | 1.79 |
| ENSTGUG00000007962 | NFKBIE | 0.035 | 0.002 | 24.0 | 14.0 | 1.78 |
| ENSTGUG00000002622 | RAB6B | 0.035 | 0.002 | 24.0 | 5.0 | 1.78 |
| ENSTGUG00000004417 | FAM29A | 0.035 | 0.002 | 24.0 | 6.0 | 1.77 |
| ENSTGUG00000016515 |  | 0.035 | 0.002 | 24.0 | 22.0 | 1.76 |
| ENSTGUG00000007760 | KIAA0415 | 0.035 | 0.002 | 24.0 | 16.0 | 1.76 |
| ENSTGUG00000009776 | EFCAB4A-1 | 0.035 | 0.002 | 24.0 | 2.0 | 1.75 |
| ENSTGUG00000012279 | TRIM24 | 0.035 | 0.002 | 26.0 | 2.0 | 1.74 |
| ENSTGUG00000009153 | B5G2Y0_TAEGU | 0.035 | 0.002 | 24.0 | 18.0 | 1.74 |
| ENSTGUG00000004689 |  | 0.035 | 0.002 | 26.0 | 2.0 | 1.72 |
| ENSTGUG00000010642 | GTPBP6 | 0.035 | 0.002 | 24.0 | 1.0 | 1.68 |
| ENSTGUG00000000833 | DCPS | 0.035 | 0.002 | 25.3 | 16.7 | 1.67 |
| ENSTGUG00000002415 | SNRPB2 | 0.035 | 0.002 | 24.0 | 18.0 | 1.64 |
| ENSTGUG00000003405 | TOP2B | 0.035 | 0.002 | 26.0 | 2.0 | 1.63 |
| ENSTGUG00000006549 | ATP9B | 0.035 | 0.002 | 26.0 | 2.0 | 1.61 |
| ENSTGUG00000015991 |  | 0.035 | 0.002 | 24.0 | 4.0 | 1.57 |
| ENSTGUG00000014055 |  | 0.035 | 0.002 | 26.0 | 15.0 | 1.56 |
| ENSTGUG00000003738 | PTPN9 | 0.035 | 0.002 | 24.0 | 12.0 | 1.54 |
| ENSTGUG00000001687 | KIAA0319L | 0.035 | 0.002 | 26.0 | 2.0 | 1.54 |
| ENSTGUG00000005348 | TMPRSS2 | 0.035 | 0.002 | 24.0 | 6.0 | 1.54 |
| ENSTGUG00000008090 | TERF2 | 0.035 | 0.002 | 24.0 | 10.0 | 1.54 |
| ENSTGUG00000004679 |  | 0.035 | 0.002 | 24.0 | 5.0 | 1.53 |
| ENSTGUG00000011026 | PLAGL1 | 0.035 | 0.002 | 24.0 | 0.0 | 1.52 |
| ENSTGUG00000008656 | B5G1M9_TAEGU | 0.035 | 0.002 | 24.0 | 16.0 | 1.50 |
| ENSTGUG00000007717 | ZC3H18 | 0.035 | 0.002 | 26.0 | 15.0 | 1.47 |
| ENSTGUG00000006890 | RIC8A | 0.035 | 0.002 | 24.0 | 12.0 | 1.46 |
| ENSTGUG00000007169 | POLR1E | 0.035 | 0.002 | 24.0 | 14.0 | 1.42 |
| ENSTGUG00000009610 | KIAA0226 | 0.035 | 0.002 | 24.0 | 6.0 | 1.41 |
| ENSTGUG00000010455 | NOP14 | 0.035 | 0.002 | 24.0 | 17.0 | 1.41 |
| ENSTGUG00000002915 | PLCH2 | 0.035 | 0.002 | 24.0 | 5.0 | 1.40 |
| ENSTGUG00000007491 | NFKB2 | 0.035 | 0.002 | 24.0 | 14.0 | 1.39 |
| ENSTGUG00000010808 | RBM9 | 0.035 | 0.002 | 24.0 | 16.0 | 1.39 |
| ENSTGUG00000010306 | CASP10 | 0.035 | 0.002 | 24.0 | 18.0 | 1.36 |
| ENSTGUG00000011490 | FGD4 | 0.035 | 0.002 | 25.3 | 4.0 | 1.31 |
| ENSTGUG00000009449 | SMG1 | 0.035 | 0.002 | 26.0 | 2.0 | 1.28 |
| ENSTGUG00000003815 | C1orf21 | 0.035 | 0.002 | 25.3 | 16.7 | 1.28 |
| ENSTGUG00000001868 | SMO | 0.035 | 0.002 | 24.0 | 12.0 | 1.27 |
| ENSTGUG00000000097 | CDC25A | 0.035 | 0.002 | 26.0 | 15.0 | 1.25 |
| ENSTGUG00000011159 | ADARB1 | 0.035 | 0.002 | 24.0 | 6.0 | 1.25 |
| ENSTGUG00000003143 | TBC1D16 | 0.035 | 0.002 | 24.0 | 14.0 | 1.25 |
| ENSTGUG00000012569 | CPSF2 | 0.035 | 0.002 | 26.0 | 2.0 | 1.24 |
| ENSTGUG00000012747 | ZC3H3-2 | 0.035 | 0.002 | 24.0 | 2.0 | 1.24 |
| ENSTGUG00000000102 | POLE4 | 0.035 | 0.002 | 26.0 | 15.0 | 1.23 |
|  |  |  |  |  |  |  |
|  |  |  |  |  |  |  |

non- migratory state specific cycling genes

| **EnsemblGeneID** | **GeneSymbol** | **BHQ** | **ADJP** | **PERIOD** | **LAG** | **AMP** |
| --- | --- | --- | --- | --- | --- | --- |
| ENSTGUG00000008254 | PCK1 | 0.002 | 0.000 | 24.0 | 22.0 | 2672.9 |
| ENSTGUG00000005949 | FTH1 | 0.002 | 0.000 | 24.0 | 2.0 | 1291.2 |
| ENSTGUG00000003390 | NT5DC4 | 0.002 | 0.000 | 24.0 | 0.0 | 646.5 |
| ENSTGUG00000007938 | XBP1 | 0.002 | 0.000 | 24.0 | 2.0 | 249.6 |
| ENSTGUG00000010111 | ADSL | 0.002 | 0.000 | 24.0 | 4.0 | 228.1 |
| ENSTGUG00000006189 | HDLBP | 0.002 | 0.000 | 24.0 | 4.0 | 181.4 |
| ENSTGUG00000013431 | ATP1B1 | 0.002 | 0.000 | 24.0 | 2.0 | 158.7 |
| ENSTGUG00000007510 | PROC | 0.002 | 0.000 | 24.0 | 2.0 | 104.9 |
| ENSTGUG00000007332 | PRDX4 | 0.002 | 0.000 | 24.0 | 10.0 | 96.9 |
| ENSTGUG00000003450 | SLC25A10 | 0.002 | 0.000 | 24.0 | 6.0 | 85.7 |
| ENSTGUG00000011520 | ALDH8A1 | 0.002 | 0.000 | 24.0 | 2.0 | 63.9 |
| ENSTGUG00000007601 | MAP1LC3B | 0.002 | 0.000 | 24.0 | 2.0 | 59.8 |
| ENSTGUG00000017580 |  | 0.002 | 0.000 | 24.0 | 14.0 | 57.9 |
| ENSTGUG00000000865 | RPS15 | 0.002 | 0.000 | 24.0 | 10.0 | 57.0 |
| ENSTGUG00000016340 | IDH2 | 0.002 | 0.000 | 24.0 | 4.0 | 53.4 |
| ENSTGUG00000006083 | HMGCR | 0.002 | 0.000 | 24.0 | 0.0 | 52.7 |
| ENSTGUG00000002139 | PSMD11 | 0.002 | 0.000 | 24.0 | 18.0 | 48.3 |
| ENSTGUG00000003498 | ARHGDIA | 0.002 | 0.000 | 24.0 | 2.0 | 36.3 |
| ENSTGUG00000003325 | RPL15 | 0.002 | 0.000 | 24.0 | 6.0 | 33.9 |
| ENSTGUG00000009263 |  | 0.002 | 0.000 | 24.0 | 22.0 | 29.6 |
| ENSTGUG00000007796 | PITPNB | 0.002 | 0.000 | 24.0 | 12.0 | 27.7 |
| ENSTGUG00000015635 |  | 0.002 | 0.000 | 24.0 | 6.0 | 23.6 |
| ENSTGUG00000009826 | SLC25A22 | 0.002 | 0.000 | 24.0 | 0.0 | 22.2 |
| ENSTGUG00000008877 | ACTR6 | 0.002 | 0.000 | 24.0 | 16.0 | 21.7 |
| ENSTGUG00000010731 | ZNF639 | 0.002 | 0.000 | 24.0 | 16.0 | 20.8 |
| ENSTGUG00000002230 | PECI | 0.002 | 0.000 | 24.0 | 22.0 | 20.4 |
| ENSTGUG00000003296 | EIF4E | 0.002 | 0.000 | 24.0 | 16.0 | 18.0 |
| ENSTGUG00000009509 | IL4R | 0.002 | 0.000 | 24.0 | 0.0 | 15.7 |
| ENSTGUG00000011557 | UBE2W | 0.002 | 0.000 | 24.0 | 18.0 | 15.7 |
| ENSTGUG00000007125 |  | 0.002 | 0.000 | 24.0 | 0.0 | 15.4 |
| ENSTGUG00000010185 | SERBP1 | 0.002 | 0.000 | 24.0 | 0.0 | 15.1 |
| ENSTGUG00000005944 | TWF1 | 0.002 | 0.000 | 24.0 | 14.0 | 14.4 |
| ENSTGUG00000010776 | IMMT | 0.002 | 0.000 | 24.0 | 4.0 | 14.0 |
| ENSTGUG00000008267 | PGAM5 | 0.002 | 0.000 | 24.0 | 0.0 | 13.7 |
| ENSTGUG00000015721 |  | 0.002 | 0.000 | 24.0 | 2.0 | 13.6 |
| ENSTGUG00000000770 | SPARC | 0.002 | 0.000 | 24.0 | 8.0 | 11.4 |
| ENSTGUG00000008344 | NDUFA12 | 0.002 | 0.000 | 24.0 | 4.0 | 11.3 |
| ENSTGUG00000006747 | C16orf73 | 0.002 | 0.000 | 24.0 | 22.0 | 11.2 |
| ENSTGUG00000008327 | LSM3 | 0.002 | 0.000 | 24.0 | 16.0 | 10.8 |
| ENSTGUG00000011485 | BCCIP | 0.002 | 0.000 | 24.0 | 16.0 | 10.6 |
| ENSTGUG00000012410 | RRAGD | 0.002 | 0.000 | 24.0 | 14.0 | 10.6 |
| ENSTGUG00000008197 | CHMP5 | 0.002 | 0.000 | 24.0 | 16.0 | 10.6 |
| ENSTGUG00000010234 | RBM34 | 0.002 | 0.000 | 24.0 | 4.0 | 10.4 |
| ENSTGUG00000004035 | KRT7 | 0.002 | 0.000 | 24.0 | 2.0 | 10.3 |
| ENSTGUG00000003276 | TSPAN5 | 0.002 | 0.000 | 24.0 | 14.0 | 10.2 |
| ENSTGUG00000005637 | FAM168B | 0.002 | 0.000 | 24.0 | 6.0 | 10.1 |
| ENSTGUG00000005948 | HEXB | 0.002 | 0.000 | 24.0 | 14.0 | 9.7 |
| ENSTGUG00000012756 | NAPRT1 | 0.002 | 0.000 | 24.0 | 4.0 | 9.5 |
| ENSTGUG00000013025 | IAH1 | 0.002 | 0.000 | 24.0 | 12.0 | 9.3 |
| ENSTGUG00000016698 |  | 0.002 | 0.000 | 24.0 | 8.0 | 9.0 |
| ENSTGUG00000016717 |  | 0.002 | 0.000 | 24.0 | 0.0 | 8.7 |
| ENSTGUG00000001057 | UPF1 | 0.002 | 0.000 | 24.0 | 4.0 | 8.7 |
| ENSTGUG00000002035 | NMT1 | 0.002 | 0.000 | 24.0 | 0.0 | 8.2 |
| ENSTGUG00000010269 | EIF4G1 | 0.002 | 0.000 | 24.0 | 0.0 | 8.2 |
| ENSTGUG00000007588 | PDAP1 | 0.002 | 0.000 | 24.0 | 20.0 | 8.0 |
| ENSTGUG00000005311 | MDFIC | 0.002 | 0.000 | 24.0 | 2.0 | 7.4 |
| ENSTGUG00000001216 | TGFBI | 0.002 | 0.000 | 24.0 | 8.0 | 6.8 |
| ENSTGUG00000005648 | PRPF8 | 0.002 | 0.000 | 24.0 | 0.0 | 6.8 |
| ENSTGUG00000003482 | MAPKSP1 | 0.002 | 0.000 | 24.0 | 14.0 | 6.5 |
| ENSTGUG00000004097 | ENAH | 0.002 | 0.000 | 24.0 | 12.0 | 6.4 |
| ENSTGUG00000011814 | RFC3 | 0.002 | 0.000 | 24.0 | 16.0 | 5.8 |
| ENSTGUG00000012443 | TDP1 | 0.002 | 0.000 | 24.0 | 14.0 | 5.6 |
| ENSTGUG00000014807 | SAE1 | 0.002 | 0.000 | 24.0 | 8.0 | 5.6 |
| ENSTGUG00000002547 | PER3 | 0.002 | 0.000 | 24.0 | 2.0 | 5.6 |
| ENSTGUG00000013582 | NUMA1 | 0.002 | 0.000 | 24.0 | 2.0 | 5.5 |
| ENSTGUG00000011282 | AKAP12-1 | 0.002 | 0.000 | 24.0 | 8.0 | 5.2 |
| ENSTGUG00000006336 | TMEM38B | 0.002 | 0.000 | 24.0 | 20.0 | 5.2 |
| ENSTGUG00000006266 | PPP1R7 | 0.002 | 0.000 | 24.0 | 18.0 | 5.1 |
| ENSTGUG00000017285 | VAMP4 | 0.002 | 0.000 | 24.0 | 18.0 | 4.9 |
| ENSTGUG00000016429 |  | 0.002 | 0.000 | 24.0 | 14.0 | 4.8 |
| ENSTGUG00000001336 | CCDC112 | 0.002 | 0.000 | 24.0 | 0.0 | 4.8 |
| ENSTGUG00000010176 | UNC50 | 0.002 | 0.000 | 24.0 | 18.0 | 4.7 |
| ENSTGUG00000004520 | FYCO1 | 0.002 | 0.000 | 24.0 | 16.0 | 4.6 |
| ENSTGUG00000013026 | C11orf67 | 0.002 | 0.000 | 24.0 | 18.0 | 4.6 |
| ENSTGUG00000002960 | SLC38A10 | 0.002 | 0.000 | 24.0 | 2.0 | 4.6 |
| ENSTGUG00000010816 | MAPRE2 | 0.002 | 0.000 | 24.0 | 10.0 | 4.5 |
| ENSTGUG00000011222 | PCNT | 0.002 | 0.000 | 24.0 | 14.0 | 4.5 |
| ENSTGUG00000012725 | DCUN1D5 | 0.002 | 0.000 | 24.0 | 16.0 | 4.5 |
| ENSTGUG00000003537 |  | 0.002 | 0.000 | 24.0 | 16.0 | 4.5 |
| ENSTGUG00000005551 | PITPNA | 0.002 | 0.000 | 24.0 | 12.0 | 4.4 |
| ENSTGUG00000015636 |  | 0.002 | 0.000 | 24.0 | 4.0 | 4.4 |
| ENSTGUG00000016185 |  | 0.002 | 0.000 | 24.0 | 6.0 | 4.4 |
| ENSTGUG00000003247 | AMDHD2 | 0.002 | 0.000 | 24.0 | 2.0 | 4.3 |
| ENSTGUG00000013271 | EMG1 | 0.002 | 0.000 | 24.0 | 20.0 | 4.2 |
| ENSTGUG00000000315 | DIXDC1 | 0.002 | 0.000 | 24.0 | 12.0 | 4.2 |
| ENSTGUG00000013076 | E2F6 | 0.002 | 0.000 | 24.0 | 14.0 | 4.1 |
| ENSTGUG00000002764 | IARS2 | 0.002 | 0.000 | 24.0 | 22.0 | 4.0 |
| ENSTGUG00000004036 | PCNA | 0.002 | 0.000 | 24.0 | 14.0 | 4.0 |
| ENSTGUG00000012276 | CCND2 | 0.002 | 0.000 | 24.0 | 8.0 | 4.0 |
| ENSTGUG00000007281 | EFHA2 | 0.002 | 0.000 | 24.0 | 16.0 | 3.8 |
| ENSTGUG00000002603 | TAF9B | 0.002 | 0.000 | 24.0 | 4.0 | 3.5 |
| ENSTGUG00000009247 | THUMPD1 | 0.002 | 0.000 | 24.0 | 0.0 | 3.5 |
| ENSTGUG00000008692 | NLK | 0.002 | 0.000 | 24.0 | 12.0 | 3.4 |
| ENSTGUG00000013421 | GTPBP8 | 0.002 | 0.000 | 24.0 | 16.0 | 3.3 |
| ENSTGUG00000008784 | STAU1 | 0.002 | 0.000 | 24.0 | 20.0 | 3.3 |
| ENSTGUG00000008823 | CSE1L | 0.002 | 0.000 | 24.0 | 14.0 | 3.2 |
| ENSTGUG00000008905 | DGCR2 | 0.002 | 0.000 | 24.0 | 12.0 | 3.1 |
| ENSTGUG00000007701 | C15orf40 | 0.002 | 0.000 | 24.0 | 18.0 | 3.1 |
| ENSTGUG00000004319 | HGSNAT | 0.002 | 0.000 | 24.0 | 18.0 | 3.1 |
| ENSTGUG00000010471 | ADD1 | 0.002 | 0.000 | 24.0 | 14.0 | 3.0 |
| ENSTGUG00000005742 | NDUFV3 | 0.002 | 0.000 | 24.0 | 14.0 | 2.9 |
| ENSTGUG00000012378 | GTF2A1 | 0.002 | 0.000 | 24.0 | 0.0 | 2.9 |
| ENSTGUG00000003095 | ARL4C | 0.002 | 0.000 | 24.0 | 10.0 | 2.8 |
| ENSTGUG00000008310 | ARVCF | 0.002 | 0.000 | 24.0 | 6.0 | 2.8 |
| ENSTGUG00000006324 | HS2ST1 | 0.002 | 0.000 | 24.0 | 12.0 | 2.7 |
| ENSTGUG00000003677 | GPS1 | 0.002 | 0.000 | 24.0 | 16.0 | 2.7 |
| ENSTGUG00000012561 |  | 0.002 | 0.000 | 24.0 | 0.0 | 2.7 |
| ENSTGUG00000010836 | NAB1 | 0.002 | 0.000 | 24.0 | 6.0 | 2.7 |
| ENSTGUG00000012721 | FAM135A | 0.002 | 0.000 | 24.0 | 14.0 | 2.4 |
| ENSTGUG00000006994 | ZCCHC9 | 0.002 | 0.000 | 24.0 | 2.0 | 2.3 |
| ENSTGUG00000009023 | TMEM156 | 0.002 | 0.000 | 24.0 | 12.0 | 2.3 |
| ENSTGUG00000005097 | ENTPD6 | 0.002 | 0.000 | 24.0 | 14.0 | 2.3 |
| ENSTGUG00000011867 | HECTD1 | 0.002 | 0.000 | 24.0 | 22.0 | 2.2 |
| ENSTGUG00000006740 | MBTPS1 | 0.002 | 0.000 | 24.0 | 8.0 | 2.2 |
| ENSTGUG00000007730 | GLP1R | 0.002 | 0.000 | 24.0 | 6.0 | 2.2 |
| ENSTGUG00000008497 | SETD5 | 0.002 | 0.000 | 24.0 | 6.0 | 2.1 |
| ENSTGUG00000003124 | DUS4L | 0.002 | 0.000 | 24.0 | 18.0 | 2.1 |
| ENSTGUG00000009330 | IGF2 | 0.002 | 0.000 | 24.0 | 0.0 | 2.1 |
| ENSTGUG00000015872 | PHF12 | 0.002 | 0.000 | 24.0 | 2.0 | 2.0 |
| ENSTGUG00000008729 |  | 0.002 | 0.000 | 24.0 | 12.0 | 1.9 |
| ENSTGUG00000015556 |  | 0.002 | 0.000 | 24.0 | 8.0 | 1.9 |
| ENSTGUG00000005529 | YEATS2 | 0.002 | 0.000 | 24.0 | 6.0 | 1.9 |
| ENSTGUG00000002701 | PNPLA6 | 0.002 | 0.000 | 24.0 | 6.0 | 1.9 |
| ENSTGUG00000013505 | RCSD1 | 0.002 | 0.000 | 24.0 | 10.0 | 1.8 |
| ENSTGUG00000012418 | NARG1L | 0.002 | 0.000 | 24.0 | 8.0 | 1.7 |
| ENSTGUG00000008676 | CDCA7 | 0.002 | 0.000 | 24.0 | 12.0 | 1.7 |
| ENSTGUG00000008190 | ZFHX3 | 0.002 | 0.000 | 24.0 | 2.0 | 1.7 |
| ENSTGUG00000002520 | SLC45A1 | 0.002 | 0.000 | 24.0 | 0.0 | 1.6 |
| ENSTGUG00000005126 | TNS1 | 0.002 | 0.000 | 24.0 | 2.0 | 1.6 |
| ENSTGUG00000003765 | CCAR1 | 0.002 | 0.000 | 24.0 | 20.0 | 1.6 |
| ENSTGUG00000002258 | RPP40 | 0.002 | 0.000 | 24.0 | 2.0 | 1.6 |
| ENSTGUG00000010408 | TYW3 | 0.002 | 0.000 | 24.0 | 16.0 | 1.6 |
| ENSTGUG00000005004 | DBNDD2 | 0.002 | 0.000 | 24.0 | 6.0 | 1.5 |
| ENSTGUG00000017417 | RFWD2 | 0.002 | 0.000 | 24.0 | 8.0 | 1.5 |
| ENSTGUG00000015096 |  | 0.002 | 0.000 | 24.0 | 12.0 | 1.5 |
| ENSTGUG00000003198 | SATB1 | 0.002 | 0.000 | 24.0 | 14.0 | 1.4 |
| ENSTGUG00000007497 | GARNL3 | 0.002 | 0.000 | 24.0 | 14.0 | 1.4 |
| ENSTGUG00000016754 |  | 0.002 | 0.000 | 24.0 | 2.0 | 1.3 |
| ENSTGUG00000004511 | CCR1-2 | 0.002 | 0.000 | 24.0 | 12.0 | 1.3 |
| ENSTGUG00000002434 |  | 0.002 | 0.000 | 24.0 | 16.0 | 1.2 |
| ENSTGUG00000007557 | KDR | 0.002 | 0.000 | 24.0 | 8.0 | 1.1 |
| ENSTGUG00000012445 | KLHL38 | 0.002 | 0.000 | 24.0 | 14.0 | 1.1 |
| ENSTGUG00000010552 | TUBGCP5 | 0.002 | 0.000 | 24.0 | 14.0 | 1.1 |
| ENSTGUG00000009604 | LRIG1 | 0.002 | 0.000 | 24.0 | 4.0 | 1.1 |
| ENSTGUG00000009504 | IQCH | 0.002 | 0.000 | 24.0 | 12.0 | 1.1 |
| ENSTGUG00000008855 |  | 0.002 | 0.000 | 24.0 | 8.0 | 1.1 |
| ENSTGUG00000004507 | CCR1-1 | 0.002 | 0.000 | 24.0 | 14.0 | 1.0 |
| ENSTGUG00000006405 | PARP3 | 0.002 | 0.000 | 24.0 | 0.0 | 1.0 |
| ENSTGUG00000002142 | USP6NL-1 | 0.002 | 0.000 | 24.0 | 14.0 | 1.0 |
| ENSTGUG00000017290 | BAT2D1 | 0.002 | 0.000 | 24.0 | 2.0 | 1.0 |
| ENSTGUG00000004579 | CENPN | 0.002 | 0.000 | 24.0 | 14.0 | 0.9 |
| ENSTGUG00000007314 | LPCAT2 | 0.002 | 0.000 | 24.0 | 10.0 | 0.9 |
| ENSTGUG00000006788 | CYBB | 0.002 | 0.000 | 24.0 | 6.0 | 0.9 |
| ENSTGUG00000016031 |  | 0.002 | 0.000 | 24.0 | 20.0 | 0.9 |
| ENSTGUG00000007478 | ANGPTL2 | 0.002 | 0.000 | 24.0 | 2.0 | 0.8 |
| ENSTGUG00000016038 | DPF2 | 0.002 | 0.000 | 24.0 | 2.0 | 0.8 |
| ENSTGUG00000003887 |  | 0.002 | 0.000 | 24.0 | 22.0 | 0.8 |
| ENSTGUG00000014072 |  | 0.002 | 0.000 | 24.0 | 12.0 | 0.8 |
| ENSTGUG00000012788 |  | 0.002 | 0.000 | 24.0 | 4.0 | 0.8 |
| ENSTGUG00000012203 | MLH3 | 0.002 | 0.000 | 24.0 | 14.0 | 0.8 |
| ENSTGUG00000004849 | CLEC16A | 0.002 | 0.000 | 24.0 | 22.0 | 0.7 |
| ENSTGUG00000002890 | ERBB2IP | 0.002 | 0.000 | 24.0 | 8.0 | 0.7 |
| ENSTGUG00000000520 | RNF44 | 0.002 | 0.000 | 24.0 | 8.0 | 0.7 |
| ENSTGUG00000013061 |  | 0.002 | 0.000 | 24.0 | 6.0 | 0.7 |
| ENSTGUG00000001163 | THNSL1 | 0.002 | 0.000 | 24.0 | 20.0 | 0.7 |
| ENSTGUG00000004635 | VAV3 | 0.002 | 0.000 | 24.0 | 14.0 | 0.7 |
| ENSTGUG00000006079 | JAG1 | 0.002 | 0.000 | 24.0 | 8.0 | 0.6 |
| ENSTGUG00000005240 | LRRC39 | 0.002 | 0.000 | 24.0 | 0.0 | 0.6 |
| ENSTGUG00000007227 | SCLY | 0.002 | 0.000 | 24.0 | 0.0 | 0.6 |
| ENSTGUG00000007194 | APPL1 | 0.002 | 0.000 | 24.0 | 12.0 | 0.6 |
| ENSTGUG00000004852 | PAOX | 0.002 | 0.000 | 24.0 | 4.0 | 0.6 |
| ENSTGUG00000005914 |  | 0.002 | 0.000 | 24.0 | 10.0 | 0.6 |
| ENSTGUG00000011143 | CDAN1 | 0.002 | 0.000 | 24.0 | 14.0 | 0.6 |
| ENSTGUG00000008483 | ATP8A1 | 0.002 | 0.000 | 24.0 | 2.0 | 0.6 |
| ENSTGUG00000006675 | TTC19 | 0.002 | 0.000 | 24.0 | 22.0 | 0.6 |
| ENSTGUG00000008533 | ATR | 0.002 | 0.000 | 24.0 | 18.0 | 0.6 |
| ENSTGUG00000011871 | CDH17 | 0.002 | 0.000 | 24.0 | 12.0 | 0.5 |
| ENSTGUG00000008506 | RNF215 | 0.002 | 0.000 | 24.0 | 22.0 | 0.5 |
| ENSTGUG00000010792 |  | 0.002 | 0.000 | 24.0 | 14.0 | 0.5 |
| ENSTGUG00000011550 |  | 0.002 | 0.000 | 24.0 | 8.0 | 0.5 |
| ENSTGUG00000002566 | CAMTA1 | 0.002 | 0.000 | 24.0 | 0.0 | 0.5 |
| ENSTGUG00000003548 | BICD2 | 0.002 | 0.000 | 24.0 | 6.0 | 0.5 |
| ENSTGUG00000008842 | AGTR1 | 0.002 | 0.000 | 24.0 | 12.0 | 0.5 |
| ENSTGUG00000005111 | SPON1 | 0.002 | 0.000 | 24.0 | 6.0 | 0.5 |
| ENSTGUG00000016506 |  | 0.002 | 0.000 | 24.0 | 8.0 | 0.4 |
| ENSTGUG00000009221 | RTTN | 0.002 | 0.000 | 24.0 | 16.0 | 0.4 |
| ENSTGUG00000018540 |  | 0.002 | 0.000 | 24.0 | 2.0 | 0.4 |
| ENSTGUG00000002567 | BZW2 | 0.002 | 0.000 | 24.0 | 18.0 | 0.4 |
| ENSTGUG00000003342 | TLR4 | 0.002 | 0.000 | 24.0 | 8.0 | 0.4 |
| ENSTGUG00000001145 | LOXL1 | 0.002 | 0.000 | 24.0 | 4.0 | 0.4 |
| ENSTGUG00000011059 |  | 0.002 | 0.000 | 24.0 | 10.0 | 0.4 |
| ENSTGUG00000016120 |  | 0.002 | 0.000 | 24.0 | 14.0 | 0.4 |
| ENSTGUG00000004879 | ALPK3 | 0.002 | 0.000 | 24.0 | 14.0 | 0.4 |
| ENSTGUG00000016732 | CD28 | 0.002 | 0.000 | 24.0 | 10.0 | 0.4 |
| ENSTGUG00000005144 | B3GALT5 | 0.002 | 0.000 | 24.0 | 8.0 | 0.4 |
| ENSTGUG00000003948 | GPD1-2 | 0.002 | 0.000 | 24.0 | 10.0 | 0.4 |
| ENSTGUG00000009893 | IL1R2 | 0.002 | 0.000 | 24.0 | 2.0 | 0.3 |
| ENSTGUG00000014475 | ASCC3L1 | 0.002 | 0.000 | 24.0 | 2.0 | 0.3 |
| ENSTGUG00000003179 | CHD6 | 0.002 | 0.000 | 24.0 | 0.0 | 0.3 |
| ENSTGUG00000002058 | C5orf42 | 0.002 | 0.000 | 24.0 | 12.0 | 0.3 |
| ENSTGUG00000014292 |  | 0.002 | 0.000 | 24.0 | 8.0 | 0.3 |
| ENSTGUG00000010242 | GSTT1 | 0.002 | 0.000 | 24.0 | 8.0 | 0.3 |
| ENSTGUG00000012553 | DOPEY1 | 0.002 | 0.000 | 24.0 | 14.0 | 0.3 |
| ENSTGUG00000010933 | ADRA1D | 0.002 | 0.000 | 24.0 | 8.0 | 0.2 |
| ENSTGUG00000012385 | CASP8AP2 | 0.002 | 0.000 | 24.0 | 16.0 | 0.2 |
| ENSTGUG00000012186 | RPS6KL1 | 0.002 | 0.000 | 24.0 | 12.0 | 0.2 |
| ENSTGUG00000004901 | PPAPDC3 | 0.002 | 0.000 | 24.0 | 12.0 | 0.2 |
| ENSTGUG00000004647 | ARNT | 0.002 | 0.000 | 24.0 | 2.0 | 0.2 |
| ENSTGUG00000013699 |  | 0.002 | 0.000 | 24.0 | 2.0 | 0.2 |
| ENSTGUG00000007956 |  | 0.002 | 0.000 | 24.0 | 14.0 | 0.2 |
| ENSTGUG00000006320 | KNTC1 | 0.002 | 0.000 | 24.0 | 12.0 | 0.1 |
| ENSTGUG00000007509 | CELSR3 | 0.002 | 0.000 | 24.0 | 2.0 | 0.1 |
| ENSTGUG00000007691 | SLC29A4 | 0.002 | 0.000 | 24.0 | 12.0 | 0.1 |
| ENSTGUG00000013126 | KCNS3 | 0.002 | 0.000 | 24.0 | 12.0 | 0.1 |
| ENSTGUG00000008434 | PLXNB1 | 0.002 | 0.000 | 24.0 | 12.0 | 0.1 |
| ENSTGUG00000018415 |  | 0.002 | 0.000 | 24.0 | 12.0 | 0.1 |
| ENSTGUG00000012625 | KIAA1409 | 0.002 | 0.000 | 24.0 | 12.0 | 0.0 |
| ENSTGUG00000015454 | RAB11FIP5 | 0.018 | 0.000 | 24.0 | 4.0 | 1.0 |
| ENSTGUG00000010294 | KIAA1772 | 0.018 | 0.000 | 24.0 | 22.0 | 1.0 |
| ENSTGUG00000005054 | CAMKV | 0.018 | 0.000 | 24.0 | 12.0 | 0.9 |
| ENSTGUG00000008991 | APOD | 0.018 | 0.000 | 24.0 | 2.0 | 0.8 |
| ENSTGUG00000000034 |  | 0.018 | 0.000 | 24.0 | 4.0 | 0.6 |
| ENSTGUG00000004609 | FREQ | 0.018 | 0.000 | 24.0 | 8.0 | 0.6 |
| ENSTGUG00000006858 | P2RX5 | 0.018 | 0.000 | 24.0 | 12.0 | 0.5 |
| ENSTGUG00000014958 |  | 0.018 | 0.000 | 24.0 | 12.0 | 0.5 |
| ENSTGUG00000001608 | CXXC5 | 0.018 | 0.000 | 24.0 | 10.0 | 0.5 |
| ENSTGUG00000010058 | PPP2R2C | 0.018 | 0.000 | 24.0 | 8.0 | 0.4 |
| ENSTGUG00000018132 |  | 0.018 | 0.000 | 24.0 | 4.0 | 0.4 |
| ENSTGUG00000005830 | 1-Mar | 0.018 | 0.000 | 24.0 | 10.0 | 0.4 |
| ENSTGUG00000009205 | CBLN2 | 0.018 | 0.000 | 24.0 | 12.0 | 0.3 |
| ENSTGUG00000002893 |  | 0.018 | 0.000 | 24.0 | 12.0 | 0.3 |
| ENSTGUG00000009638 | ITGA11 | 0.018 | 0.000 | 24.0 | 8.0 | 0.3 |
| ENSTGUG00000006278 | MS4A8B | 0.018 | 0.000 | 24.0 | 12.0 | 0.3 |
| ENSTGUG00000008378 | TFAP2C | 0.018 | 0.000 | 24.0 | 0.0 | 0.2 |
| ENSTGUG00000005864 | C20orf12 | 0.018 | 0.000 | 24.0 | 12.0 | 0.2 |
| ENSTGUG00000012298 | TRPS1 | 0.018 | 0.000 | 24.0 | 22.0 | 0.1 |
| ENSTGUG00000016403 |  | 0.018 | 0.000 | 24.0 | 12.0 | 0.0 |
| ENSTGUG00000001743 | FRAS1 | 0.018 | 0.000 | 24.0 | 2.0 | 0.0 |
| ENSTGUG00000013287 | GAL10 | 0.025 | 0.002 | 26.0 | 5.0 | 19284.5 |
| ENSTGUG00000018669 |  | 0.025 | 0.002 | 24.0 | 12.0 | 7016.0 |
| ENSTGUG00000007683 | SCD | 0.025 | 0.002 | 24.0 | 8.0 | 2085.1 |
| ENSTGUG00000008257 | PRPS2 | 0.025 | 0.002 | 24.0 | 4.0 | 1657.6 |
| ENSTGUG00000010437 | AHSG | 0.025 | 0.002 | 24.0 | 8.0 | 1648.4 |
| ENSTGUG00000006192 |  | 0.025 | 0.002 | 24.0 | 4.0 | 1554.6 |
| ENSTGUG00000017965 |  | 0.025 | 0.002 | 24.0 | 13.0 | 740.8 |
| ENSTGUG00000014576 |  | 0.025 | 0.002 | 24.0 | 10.0 | 702.4 |
| ENSTGUG00000012881 | GSTA4-2 | 0.025 | 0.002 | 26.0 | 2.0 | 608.4 |
| ENSTGUG00000010184 | SERF2 | 0.025 | 0.002 | 24.0 | 2.0 | 487.6 |
| ENSTGUG00000012670 | EEF1A1 | 0.025 | 0.002 | 24.0 | 2.0 | 463.7 |
| ENSTGUG00000010815 | HABP2 | 0.025 | 0.002 | 26.0 | 15.0 | 452.3 |
| ENSTGUG00000017345 | GLUL | 0.025 | 0.002 | 24.0 | 1.0 | 441.1 |
| ENSTGUG00000010582 |  | 0.025 | 0.002 | 24.0 | 10.0 | 409.4 |
| ENSTGUG00000011826 | FABP7 | 0.025 | 0.002 | 24.0 | 21.0 | 390.2 |
| ENSTGUG00000012864 | ELOVL5 | 0.025 | 0.002 | 24.0 | 14.0 | 345.1 |
| ENSTGUG00000007082 | SHMT1 | 0.025 | 0.002 | 26.0 | 2.0 | 294.2 |
| ENSTGUG00000018763 |  | 0.025 | 0.002 | 24.0 | 22.0 | 289.6 |
| ENSTGUG00000017558 |  | 0.025 | 0.002 | 24.0 | 13.0 | 242.7 |
| ENSTGUG00000016179 |  | 0.025 | 0.002 | 24.0 | 14.0 | 237.5 |
| ENSTGUG00000000895 | B5FZC5_TAEGU | 0.025 | 0.002 | 24.0 | 4.0 | 222.2 |
| ENSTGUG00000007127 | GJB1 | 0.025 | 0.002 | 24.0 | 0.0 | 208.1 |
| ENSTGUG00000001282 |  | 0.025 | 0.002 | 24.0 | 1.0 | 207.8 |
| ENSTGUG00000003977 | CYP1A2 | 0.025 | 0.002 | 24.0 | 2.0 | 203.5 |
| ENSTGUG00000012323 | SLCO1B1 | 0.025 | 0.002 | 26.0 | 18.0 | 179.6 |
| ENSTGUG00000009259 | CD81 | 0.025 | 0.002 | 24.0 | 2.0 | 167.4 |
| ENSTGUG00000010709 | GPAM | 0.025 | 0.002 | 24.0 | 12.0 | 148.7 |
| ENSTGUG00000001699 | LGALS1-1 | 0.025 | 0.002 | 24.0 | 9.0 | 143.7 |
| ENSTGUG00000013240 | CYP39A1 | 0.025 | 0.002 | 24.0 | 0.0 | 116.9 |
| ENSTGUG00000009678 | CES3-1 | 0.025 | 0.002 | 24.0 | 12.0 | 111.0 |
| ENSTGUG00000006186 | TLCD1 | 0.025 | 0.002 | 26.0 | 5.0 | 110.1 |
| ENSTGUG00000011797 | PPARA | 0.025 | 0.002 | 26.0 | 15.0 | 110.0 |
| ENSTGUG00000008832 | GPT2 | 0.025 | 0.002 | 24.0 | 4.0 | 106.2 |
| ENSTGUG00000000178 | ATP5I | 0.025 | 0.002 | 24.0 | 4.0 | 104.8 |
| ENSTGUG00000013386 | ATP1A1 | 0.025 | 0.002 | 26.0 | 2.0 | 98.1 |
| ENSTGUG00000008351 | GPR146 | 0.025 | 0.002 | 24.0 | 0.0 | 94.3 |
| ENSTGUG00000013480 | RPS3 | 0.025 | 0.002 | 26.0 | 5.0 | 93.7 |
| ENSTGUG00000002404 | EIF1 | 0.025 | 0.002 | 24.0 | 4.0 | 92.5 |
| ENSTGUG00000012247 | ARFGAP3 | 0.025 | 0.002 | 24.0 | 12.0 | 90.6 |
| ENSTGUG00000010695 | ATOH8 | 0.025 | 0.002 | 24.0 | 21.0 | 89.3 |
| ENSTGUG00000018459 |  | 0.025 | 0.002 | 26.0 | 5.0 | 83.6 |
| ENSTGUG00000006517 | SLC38A3 | 0.025 | 0.002 | 24.0 | 1.0 | 77.8 |
| ENSTGUG00000003552 | DPM2 | 0.025 | 0.002 | 24.0 | 1.0 | 74.7 |
| ENSTGUG00000014285 |  | 0.025 | 0.002 | 26.0 | 15.0 | 74.5 |
| ENSTGUG00000013310 | MLF2 | 0.025 | 0.002 | 24.0 | 10.0 | 74.3 |
| ENSTGUG00000010250 | MIF | 0.025 | 0.002 | 25.3 | 4.0 | 74.2 |
| ENSTGUG00000003152 | UGT1A8 | 0.025 | 0.002 | 24.0 | 22.0 | 74.1 |
| ENSTGUG00000010251 | NT5C2 | 0.025 | 0.002 | 24.0 | 2.0 | 66.2 |
| ENSTGUG00000009033 | CLPX | 0.025 | 0.002 | 24.0 | 14.0 | 62.5 |
| ENSTGUG00000002676 | RPL12 | 0.025 | 0.002 | 26.0 | 5.0 | 60.7 |
| ENSTGUG00000005900 | POLDIP2 | 0.025 | 0.002 | 26.0 | 5.0 | 60.6 |
| ENSTGUG00000009418 | GOT1 | 0.025 | 0.002 | 24.0 | 4.0 | 59.7 |
| ENSTGUG00000001332 | AK2 | 0.025 | 0.002 | 24.0 | 2.0 | 58.7 |
| ENSTGUG00000009884 | PMM1 | 0.025 | 0.002 | 24.0 | 22.0 | 58.5 |
| ENSTGUG00000016312 | FAM165A | 0.025 | 0.002 | 24.0 | 0.0 | 57.5 |
| ENSTGUG00000005910 | NDUFAB1 | 0.025 | 0.002 | 24.0 | 2.0 | 52.8 |
| ENSTGUG00000006108 | CTSA | 0.025 | 0.002 | 24.0 | 4.0 | 51.3 |
| ENSTGUG00000008229 | FH | 0.025 | 0.002 | 24.0 | 4.0 | 50.4 |
| ENSTGUG00000013279 | C12orf57 | 0.025 | 0.002 | 26.0 | 5.0 | 47.4 |
| ENSTGUG00000000044 | RPL17 | 0.025 | 0.002 | 24.0 | 4.0 | 45.6 |
| ENSTGUG00000011260 | LSS | 0.025 | 0.002 | 24.0 | 4.0 | 44.8 |
| ENSTGUG00000004330 | PCBD1-1 | 0.025 | 0.002 | 25.3 | 4.0 | 44.2 |
| ENSTGUG00000010220 | PDIA3 | 0.025 | 0.002 | 24.0 | 0.0 | 43.1 |
| ENSTGUG00000004657 | POR | 0.025 | 0.002 | 24.0 | 1.0 | 42.3 |
| ENSTGUG00000010472 | RPL32 | 0.025 | 0.002 | 24.0 | 4.0 | 42.0 |
| ENSTGUG00000007885 | SEPP1 | 0.025 | 0.002 | 25.3 | 4.0 | 40.4 |
| ENSTGUG00000007231 | ACYP2 | 0.025 | 0.002 | 24.0 | 20.0 | 39.5 |
| ENSTGUG00000007574 | SLC6A9 | 0.025 | 0.002 | 26.0 | 2.0 | 39.0 |
| ENSTGUG00000004480 | SGK2 | 0.025 | 0.002 | 24.0 | 21.0 | 38.3 |
| ENSTGUG00000014940 |  | 0.025 | 0.002 | 24.0 | 4.0 | 38.2 |
| ENSTGUG00000016631 | SDHB | 0.025 | 0.002 | 25.3 | 4.0 | 38.2 |
| ENSTGUG00000004376 | PKM2 | 0.025 | 0.002 | 24.0 | 10.0 | 37.8 |
| ENSTGUG00000013934 |  | 0.025 | 0.002 | 24.0 | 20.0 | 37.8 |
| ENSTGUG00000009785 | PNPLA2 | 0.025 | 0.002 | 24.0 | 0.0 | 36.8 |
| ENSTGUG00000000967 | SIKE | 0.025 | 0.002 | 24.0 | 1.0 | 36.1 |
| ENSTGUG00000014433 | HADHB | 0.025 | 0.002 | 24.0 | 4.0 | 35.8 |
| ENSTGUG00000001982 | MKLN1 | 0.025 | 0.002 | 24.0 | 21.0 | 35.7 |
| ENSTGUG00000003795 | PPA2 | 0.025 | 0.002 | 26.0 | 18.0 | 35.5 |
| ENSTGUG00000011088 | BCHE | 0.025 | 0.002 | 24.0 | 0.0 | 34.4 |
| ENSTGUG00000009957 | AGT | 0.025 | 0.002 | 26.0 | 5.0 | 33.5 |
| ENSTGUG00000001470 | CCNG1 | 0.025 | 0.002 | 24.0 | 13.0 | 33.1 |
| ENSTGUG00000010394 | LGALS8 | 0.025 | 0.002 | 24.0 | 8.0 | 32.9 |
| ENSTGUG00000012467 | C6orf162 | 0.025 | 0.002 | 24.0 | 18.0 | 32.4 |
| ENSTGUG00000003576 | MAP1LC3A | 0.025 | 0.002 | 24.0 | 0.0 | 32.0 |
| ENSTGUG00000000931 | HISPPD1 | 0.025 | 0.002 | 24.0 | 0.0 | 31.4 |
| ENSTGUG00000006028 | DUSP7 | 0.025 | 0.002 | 24.0 | 9.0 | 31.2 |
| ENSTGUG00000011414 | MRPL18 | 0.025 | 0.002 | 24.0 | 9.0 | 30.9 |
| ENSTGUG00000001814 | BLVRA | 0.025 | 0.002 | 24.0 | 4.0 | 30.8 |
| ENSTGUG00000009494 |  | 0.025 | 0.002 | 24.0 | 13.0 | 30.0 |
| ENSTGUG00000003236 | RAB5A | 0.025 | 0.002 | 24.0 | 16.0 | 29.2 |
| ENSTGUG00000002538 | MRPL33 | 0.025 | 0.002 | 24.0 | 20.0 | 29.1 |
| ENSTGUG00000009388 | LSP1 | 0.025 | 0.002 | 24.0 | 10.0 | 28.8 |
| ENSTGUG00000010073 | RHOU | 0.025 | 0.002 | 26.0 | 2.0 | 27.6 |
| ENSTGUG00000012757 | SLC25A29 | 0.025 | 0.002 | 24.0 | 0.0 | 27.6 |
| ENSTGUG00000001201 | C1orf128 | 0.025 | 0.002 | 24.0 | 6.0 | 27.5 |
| ENSTGUG00000012700 | PTP4A3 | 0.025 | 0.002 | 24.0 | 2.0 | 27.5 |
| ENSTGUG00000007699 | FAM103A1 | 0.025 | 0.002 | 24.0 | 12.0 | 26.6 |
| ENSTGUG00000008967 | BDH1 | 0.025 | 0.002 | 24.0 | 0.0 | 26.5 |
| ENSTGUG00000012067 | PABPC1 | 0.025 | 0.002 | 24.0 | 6.0 | 26.4 |
| ENSTGUG00000004080 | FAM20A | 0.025 | 0.002 | 26.0 | 5.0 | 26.4 |
| ENSTGUG00000008702 | TMEM97 | 0.025 | 0.002 | 24.0 | 8.0 | 25.8 |
| ENSTGUG00000002198 | NUDT5 | 0.025 | 0.002 | 24.0 | 13.0 | 25.7 |
| ENSTGUG00000002797 | B5FZN7_TAEGU | 0.025 | 0.002 | 26.0 | 18.0 | 25.4 |
| ENSTGUG00000008977 | NR1H4 | 0.025 | 0.002 | 24.0 | 1.0 | 25.2 |
| ENSTGUG00000002325 | TBC1D9 | 0.025 | 0.002 | 24.0 | 1.0 | 24.9 |
| ENSTGUG00000003974 | UBE2G1 | 0.025 | 0.002 | 24.0 | 13.0 | 24.4 |
| ENSTGUG00000008175 | LUM | 0.025 | 0.002 | 24.0 | 9.0 | 24.2 |
| ENSTGUG00000002562 | VAMP3 | 0.025 | 0.002 | 24.0 | 4.0 | 24.1 |
| ENSTGUG00000005554 | KIAA0895L-2 | 0.025 | 0.002 | 24.0 | 4.0 | 24.1 |
| ENSTGUG00000005961 | AGPAT3 | 0.025 | 0.002 | 24.0 | 17.0 | 24.0 |
| ENSTGUG00000010141 | SUMO1 | 0.025 | 0.002 | 24.0 | 17.0 | 23.8 |
| ENSTGUG00000007553 | B4GALT2 | 0.025 | 0.002 | 24.0 | 8.0 | 23.4 |
| ENSTGUG00000000663 | C19orf22 | 0.025 | 0.002 | 26.0 | 5.0 | 23.3 |
| ENSTGUG00000011499 | C14orf83 | 0.025 | 0.002 | 24.0 | 6.0 | 23.1 |
| ENSTGUG00000000873 | GAMT | 0.025 | 0.002 | 24.0 | 8.0 | 22.5 |
| ENSTGUG00000005218 | B5FXI1_TAEGU | 0.025 | 0.002 | 24.0 | 2.0 | 22.3 |
| ENSTGUG00000011583 |  | 0.025 | 0.002 | 26.0 | 2.0 | 21.8 |
| ENSTGUG00000009555 | SCPEP1 | 0.025 | 0.002 | 24.0 | 10.0 | 21.8 |
| ENSTGUG00000008458 | GABARAPL2 | 0.025 | 0.002 | 24.0 | 20.0 | 21.4 |
| ENSTGUG00000011863 | MAN1A1 | 0.025 | 0.002 | 24.0 | 1.0 | 21.2 |
| ENSTGUG00000011645 | C15orf24 | 0.025 | 0.002 | 24.0 | 21.0 | 20.8 |
| ENSTGUG00000016310 |  | 0.025 | 0.002 | 24.0 | 9.0 | 20.7 |
| ENSTGUG00000013204 | MRPL19 | 0.025 | 0.002 | 24.0 | 14.0 | 20.5 |
| ENSTGUG00000001409 |  | 0.025 | 0.002 | 26.0 | 15.0 | 19.6 |
| ENSTGUG00000007297 | IVD | 0.025 | 0.002 | 24.0 | 4.0 | 19.1 |
| ENSTGUG00000009655 | MED28 | 0.025 | 0.002 | 24.0 | 13.0 | 18.9 |
| ENSTGUG00000001496 | HSD11B1 | 0.025 | 0.002 | 24.0 | 12.0 | 18.3 |
| ENSTGUG00000001274 | UGCG | 0.025 | 0.002 | 24.0 | 14.0 | 18.2 |
| ENSTGUG00000012819 | ANKRD9 | 0.025 | 0.002 | 24.0 | 8.0 | 18.1 |
| ENSTGUG00000011960 | NDUFA9 | 0.025 | 0.002 | 24.0 | 20.0 | 17.9 |
| ENSTGUG00000012342 | COLEC10 | 0.025 | 0.002 | 24.0 | 8.0 | 17.7 |
| ENSTGUG00000008170 | PSMD7 | 0.025 | 0.002 | 24.0 | 21.0 | 17.6 |
| ENSTGUG00000005889 | ARL6IP1 | 0.025 | 0.002 | 26.0 | 18.0 | 17.4 |
| ENSTGUG00000005549 | KANK1 | 0.025 | 0.002 | 24.0 | 0.0 | 17.2 |
| ENSTGUG00000013036 | PSMA3 | 0.025 | 0.002 | 24.0 | 20.0 | 17.0 |
| ENSTGUG00000006982 | RAB3IP | 0.025 | 0.002 | 24.0 | 14.0 | 16.8 |
| ENSTGUG00000000874 |  | 0.025 | 0.002 | 24.0 | 4.0 | 16.8 |
| ENSTGUG00000002030 | C5orf33 | 0.025 | 0.002 | 24.0 | 6.0 | 16.3 |
| ENSTGUG00000002255 | ORMDL3 | 0.025 | 0.002 | 26.0 | 2.0 | 16.2 |
| ENSTGUG00000006687 | HM13 | 0.025 | 0.002 | 24.0 | 2.0 | 16.1 |
| ENSTGUG00000006309 | BPI | 0.025 | 0.002 | 24.0 | 4.0 | 15.9 |
| ENSTGUG00000000411 | ARRDC2 | 0.025 | 0.002 | 24.0 | 18.0 | 15.8 |
| ENSTGUG00000006957 | SELK | 0.025 | 0.002 | 24.0 | 20.0 | 15.8 |
| ENSTGUG00000006031 | AGPAT2 | 0.025 | 0.002 | 24.0 | 13.0 | 15.8 |
| ENSTGUG00000002839 |  | 0.025 | 0.002 | 24.0 | 2.0 | 15.4 |
| ENSTGUG00000002663 | LSM6 | 0.025 | 0.002 | 24.0 | 16.0 | 15.3 |
| ENSTGUG00000007284 | SNX12 | 0.025 | 0.002 | 24.0 | 4.0 | 15.3 |
| ENSTGUG00000012482 | SNX14 | 0.025 | 0.002 | 26.0 | 18.0 | 14.8 |
| ENSTGUG00000013895 |  | 0.025 | 0.002 | 26.0 | 5.0 | 14.7 |
| ENSTGUG00000009757 | POLR2L | 0.025 | 0.002 | 24.0 | 16.0 | 14.7 |
| ENSTGUG00000002575 | DNAJC11 | 0.025 | 0.002 | 24.0 | 0.0 | 14.6 |
| ENSTGUG00000011640 | ENPP3 | 0.025 | 0.002 | 24.0 | 18.0 | 14.5 |
| ENSTGUG00000000534 | IFNGR2 | 0.025 | 0.002 | 24.0 | 22.0 | 14.3 |
| ENSTGUG00000007396 | ITGB1BP3 | 0.025 | 0.002 | 24.0 | 13.0 | 14.3 |
| ENSTGUG00000004158 | NUDT16L1 | 0.025 | 0.002 | 24.0 | 1.0 | 14.2 |
| ENSTGUG00000010886 | IFNGR1 | 0.025 | 0.002 | 24.0 | 4.0 | 14.2 |
| ENSTGUG00000012952 | TMEM18 | 0.025 | 0.002 | 24.0 | 14.0 | 14.1 |
| ENSTGUG00000014407 |  | 0.025 | 0.002 | 24.0 | 2.0 | 14.0 |
| ENSTGUG00000016288 |  | 0.025 | 0.002 | 24.0 | 9.0 | 14.0 |
| ENSTGUG00000009383 | QKI | 0.025 | 0.002 | 26.0 | 2.0 | 13.8 |
| ENSTGUG00000008934 | MED31 | 0.025 | 0.002 | 24.0 | 14.0 | 13.7 |
| ENSTGUG00000017539 | LSM5 | 0.025 | 0.002 | 24.0 | 16.0 | 13.6 |
| ENSTGUG00000001723 | CCNI | 0.025 | 0.002 | 24.0 | 4.0 | 13.5 |
| ENSTGUG00000012141 | SNX3 | 0.025 | 0.002 | 24.0 | 14.0 | 13.3 |
| ENSTGUG00000014979 | RAD23A | 0.025 | 0.002 | 24.0 | 6.0 | 13.2 |
| ENSTGUG00000011375 | SELT | 0.025 | 0.002 | 24.0 | 14.0 | 13.1 |
| ENSTGUG00000009478 | HLF | 0.025 | 0.002 | 25.3 | 4.0 | 12.9 |
| ENSTGUG00000006414 | DDB1 | 0.025 | 0.002 | 24.0 | 2.0 | 12.9 |
| ENSTGUG00000008441 | GNPDA2 | 0.025 | 0.002 | 24.0 | 13.0 | 12.5 |
| ENSTGUG00000013086 | SAMD4A | 0.025 | 0.002 | 24.0 | 8.0 | 12.4 |
| ENSTGUG00000010915 | PCCA | 0.025 | 0.002 | 24.0 | 2.0 | 12.4 |
| ENSTGUG00000001616 | TRAPPC3 | 0.025 | 0.002 | 24.0 | 13.0 | 12.3 |
| ENSTGUG00000004721 | EIF4EBP1 | 0.025 | 0.002 | 24.0 | 4.0 | 12.3 |
| ENSTGUG00000010036 | PDCL3 | 0.025 | 0.002 | 24.0 | 14.0 | 12.2 |
| ENSTGUG00000000824 | ZFAND5 | 0.025 | 0.002 | 24.0 | 13.0 | 12.1 |
| ENSTGUG00000000018 | VPS11 | 0.025 | 0.002 | 24.0 | 20.0 | 12.1 |
| ENSTGUG00000001519 | BET1 | 0.025 | 0.002 | 24.0 | 17.0 | 12.1 |
| ENSTGUG00000009577 | UBE2I | 0.025 | 0.002 | 24.0 | 16.0 | 12.0 |
| ENSTGUG00000002305 | LY86 | 0.025 | 0.002 | 24.0 | 10.0 | 11.9 |
| ENSTGUG00000004902 | KLHDC3 | 0.025 | 0.002 | 24.0 | 8.0 | 11.9 |
| ENSTGUG00000012066 | ALG11 | 0.025 | 0.002 | 24.0 | 18.0 | 11.9 |
| ENSTGUG00000005298 |  | 0.025 | 0.002 | 24.0 | 2.0 | 11.9 |
| ENSTGUG00000008600 | C9orf6 | 0.025 | 0.002 | 26.0 | 18.0 | 11.9 |
| ENSTGUG00000006601 | PDRG1 | 0.025 | 0.002 | 24.0 | 2.0 | 11.9 |
| ENSTGUG00000016336 |  | 0.025 | 0.002 | 24.0 | 12.0 | 11.8 |
| ENSTGUG00000008861 | UHRF1BP1L | 0.025 | 0.002 | 24.0 | 14.0 | 11.8 |
| ENSTGUG00000003394 | C20orf24 | 0.025 | 0.002 | 24.0 | 13.0 | 11.6 |
| ENSTGUG00000000820 | GTPBP4 | 0.025 | 0.002 | 24.0 | 21.0 | 11.6 |
| ENSTGUG00000010049 | FAM119A | 0.025 | 0.002 | 24.0 | 4.0 | 11.4 |
| ENSTGUG00000008757 | ATP5H | 0.025 | 0.002 | 24.0 | 2.0 | 11.4 |
| ENSTGUG00000011057 | GOLIM4 | 0.025 | 0.002 | 24.0 | 20.0 | 11.3 |
| ENSTGUG00000001453 | UTP11L | 0.025 | 0.002 | 24.0 | 0.0 | 11.2 |
| ENSTGUG00000005765 | WDR5 | 0.025 | 0.002 | 24.0 | 16.0 | 11.0 |
| ENSTGUG00000002958 | NPLOC4 | 0.025 | 0.002 | 24.0 | 4.0 | 10.9 |
| ENSTGUG00000011957 | FRK | 0.025 | 0.002 | 24.0 | 22.0 | 10.8 |
| ENSTGUG00000008482 | DPY30 | 0.025 | 0.002 | 24.0 | 14.0 | 10.8 |
| ENSTGUG00000012474 | RNF139 | 0.025 | 0.002 | 24.0 | 13.0 | 10.8 |
| ENSTGUG00000001407 | SHROOM1 | 0.025 | 0.002 | 24.0 | 14.0 | 10.6 |
| ENSTGUG00000002364 | C7orf23 | 0.025 | 0.002 | 24.0 | 14.0 | 10.4 |
| ENSTGUG00000005617 | PNPLA8 | 0.025 | 0.002 | 24.0 | 20.0 | 10.3 |
| ENSTGUG00000007479 | IP6K2 | 0.025 | 0.002 | 24.0 | 22.0 | 10.2 |
| ENSTGUG00000011601 | STX7 | 0.025 | 0.002 | 24.0 | 0.0 | 10.1 |
| ENSTGUG00000006530 | C15orf15 | 0.025 | 0.002 | 24.0 | 17.0 | 10.1 |
| ENSTGUG00000014267 |  | 0.025 | 0.002 | 25.3 | 4.0 | 10.0 |
| ENSTGUG00000014999 |  | 0.025 | 0.002 | 26.0 | 15.0 | 10.0 |
| ENSTGUG00000005402 | LBH | 0.025 | 0.002 | 24.0 | 8.0 | 10.0 |
| ENSTGUG00000005800 | RXRA | 0.025 | 0.002 | 26.0 | 5.0 | 9.9 |
| ENSTGUG00000000799 | ANXA6 | 0.025 | 0.002 | 24.0 | 2.0 | 9.8 |
| ENSTGUG00000005693 | ELMO2 | 0.025 | 0.002 | 24.0 | 10.0 | 9.6 |
| ENSTGUG00000011917 | EGLN3 | 0.025 | 0.002 | 25.3 | 4.0 | 9.6 |
| ENSTGUG00000010270 | CD82 | 0.025 | 0.002 | 24.0 | 12.0 | 9.5 |
| ENSTGUG00000012479 | AKR1B10-3 | 0.025 | 0.002 | 24.0 | 10.0 | 9.4 |
| ENSTGUG00000005845 | TIMM23B | 0.025 | 0.002 | 24.0 | 0.0 | 9.4 |
| ENSTGUG00000001722 | SFPQ | 0.025 | 0.002 | 24.0 | 20.0 | 9.4 |
| ENSTGUG00000000953 | NUDT12 | 0.025 | 0.002 | 24.0 | 1.0 | 9.3 |
| ENSTGUG00000017485 | SEC11C | 0.025 | 0.002 | 24.0 | 22.0 | 9.3 |
| ENSTGUG00000000313 | ATP6V0E1 | 0.025 | 0.002 | 24.0 | 13.0 | 9.2 |
| ENSTGUG00000004572 | EHD3 | 0.025 | 0.002 | 26.0 | 5.0 | 9.1 |
| ENSTGUG00000001690 | SCARB2 | 0.025 | 0.002 | 24.0 | 13.0 | 9.1 |
| ENSTGUG00000001302 | C5orf15 | 0.025 | 0.002 | 24.0 | 20.0 | 9.0 |
| ENSTGUG00000006651 | B5G1C5_TAEGU | 0.025 | 0.002 | 24.0 | 18.0 | 8.9 |
| ENSTGUG00000012455 | AKR1B10-2 | 0.025 | 0.002 | 24.0 | 0.0 | 8.8 |
| ENSTGUG00000006744 | RAP1B | 0.025 | 0.002 | 24.0 | 16.0 | 8.7 |
| ENSTGUG00000003330 | MRPL45 | 0.025 | 0.002 | 24.0 | 2.0 | 8.7 |
| ENSTGUG00000015749 |  | 0.025 | 0.002 | 24.0 | 9.0 | 8.7 |
| ENSTGUG00000001303 |  | 0.025 | 0.002 | 24.0 | 20.0 | 8.6 |
| ENSTGUG00000010771 |  | 0.025 | 0.002 | 24.0 | 12.0 | 8.5 |
| ENSTGUG00000001077 | SSNA1 | 0.025 | 0.002 | 24.0 | 14.0 | 8.5 |
| ENSTGUG00000001120 | AMPD2 | 0.025 | 0.002 | 24.0 | 2.0 | 8.4 |
| ENSTGUG00000006029 | UBFD1 | 0.025 | 0.002 | 24.0 | 20.0 | 8.4 |
| ENSTGUG00000002286 | PRPF18 | 0.025 | 0.002 | 24.0 | 22.0 | 8.3 |
| ENSTGUG00000001683 | UBE2R2 | 0.025 | 0.002 | 24.0 | 13.0 | 8.3 |
| ENSTGUG00000000641 | PTBP1 | 0.025 | 0.002 | 26.0 | 5.0 | 8.2 |
| ENSTGUG00000012182 | ACVR1 | 0.025 | 0.002 | 26.0 | 2.0 | 8.0 |
| ENSTGUG00000008460 | MEMO1 | 0.025 | 0.002 | 24.0 | 13.0 | 8.0 |
| ENSTGUG00000003713 | XIAP | 0.025 | 0.002 | 24.0 | 13.0 | 8.0 |
| ENSTGUG00000000302 | PLEKHJ1 | 0.025 | 0.002 | 24.0 | 8.0 | 7.8 |
| ENSTGUG00000000985 | SLC25A46 | 0.025 | 0.002 | 26.0 | 18.0 | 7.7 |
| ENSTGUG00000007067 | CTNND1 | 0.025 | 0.002 | 26.0 | 2.0 | 7.7 |
| ENSTGUG00000001553 | HIPK1 | 0.025 | 0.002 | 25.3 | 4.0 | 7.7 |
| ENSTGUG00000002182 |  | 0.025 | 0.002 | 24.0 | 21.0 | 7.6 |
| ENSTGUG00000011104 | MBNL2 | 0.025 | 0.002 | 24.0 | 4.0 | 7.6 |
| ENSTGUG00000010992 | PEX3 | 0.025 | 0.002 | 24.0 | 14.0 | 7.6 |
| ENSTGUG00000010488 | EIF3L | 0.025 | 0.002 | 24.0 | 0.0 | 7.6 |
| ENSTGUG00000010231 | PSMD2 | 0.025 | 0.002 | 24.0 | 0.0 | 7.6 |
| ENSTGUG00000000407 | OAF | 0.025 | 0.002 | 26.0 | 5.0 | 7.6 |
| ENSTGUG00000003100 | HBP1 | 0.025 | 0.002 | 24.0 | 20.0 | 7.6 |
| ENSTGUG00000012085 | COQ6 | 0.025 | 0.002 | 24.0 | 0.0 | 7.6 |
| ENSTGUG00000007369 | PLDN | 0.025 | 0.002 | 24.0 | 14.0 | 7.4 |
| ENSTGUG00000010004 | FASTKD2 | 0.025 | 0.002 | 24.0 | 16.0 | 7.4 |
| ENSTGUG00000011243 | YTHDF3 | 0.025 | 0.002 | 24.0 | 0.0 | 7.4 |
| ENSTGUG00000010932 | C6orf115 | 0.025 | 0.002 | 24.0 | 14.0 | 7.3 |
| ENSTGUG00000003158 | PDLIM5 | 0.025 | 0.002 | 24.0 | 4.0 | 7.3 |
| ENSTGUG00000001816 | STK17A | 0.025 | 0.002 | 24.0 | 21.0 | 7.3 |
| ENSTGUG00000012705 | CREBL2 | 0.025 | 0.002 | 24.0 | 22.0 | 7.3 |
| ENSTGUG00000007873 | SYAP1 | 0.025 | 0.002 | 24.0 | 14.0 | 7.2 |
| ENSTGUG00000011052 | TCP11L2 | 0.025 | 0.002 | 24.0 | 20.0 | 6.9 |
| ENSTGUG00000012031 | SLC16A10 | 0.025 | 0.002 | 24.0 | 22.0 | 6.9 |
| ENSTGUG00000010744 | DHRSX | 0.025 | 0.002 | 24.0 | 4.0 | 6.9 |
| ENSTGUG00000002290 | C5orf28 | 0.025 | 0.002 | 24.0 | 21.0 | 6.9 |
| ENSTGUG00000003726 | SLC25A33 | 0.025 | 0.002 | 24.0 | 14.0 | 6.8 |
| ENSTGUG00000007893 | DPEP1 | 0.025 | 0.002 | 24.0 | 5.0 | 6.7 |
| ENSTGUG00000007903 | CYP3A80 | 0.025 | 0.002 | 24.0 | 0.0 | 6.7 |
| ENSTGUG00000015433 |  | 0.025 | 0.002 | 24.0 | 1.0 | 6.7 |
| ENSTGUG00000012285 | USP45 | 0.025 | 0.002 | 26.0 | 15.0 | 6.6 |
| ENSTGUG00000007843 | SPG7 | 0.025 | 0.002 | 24.0 | 0.0 | 6.5 |
| ENSTGUG00000003710 | NHEDC1 | 0.025 | 0.002 | 24.0 | 13.0 | 6.5 |
| ENSTGUG00000002803 | CCDC126 | 0.025 | 0.002 | 24.0 | 13.0 | 6.5 |
| ENSTGUG00000004416 | GRAMD2 | 0.025 | 0.002 | 24.0 | 10.0 | 6.4 |
| ENSTGUG00000000965 | CSF1R | 0.025 | 0.002 | 24.0 | 8.0 | 6.4 |
| ENSTGUG00000006508 | RAB27A | 0.025 | 0.002 | 24.0 | 0.0 | 6.3 |
| ENSTGUG00000013433 | XCL1 | 0.025 | 0.002 | 24.0 | 12.0 | 6.3 |
| ENSTGUG00000003611 | MTX3 | 0.025 | 0.002 | 24.0 | 18.0 | 6.2 |
| ENSTGUG00000010564 | TMCC1 | 0.025 | 0.002 | 24.0 | 6.0 | 6.1 |
| ENSTGUG00000004289 | SLC20A2 | 0.025 | 0.002 | 24.0 | 14.0 | 6.1 |
| ENSTGUG00000009619 | PDCD2L | 0.025 | 0.002 | 24.0 | 12.0 | 6.0 |
| ENSTGUG00000011670 | SLC35F5 | 0.025 | 0.002 | 24.0 | 12.0 | 6.0 |
| ENSTGUG00000008432 | ATP1B3 | 0.025 | 0.002 | 24.0 | 6.0 | 5.9 |
| ENSTGUG00000008622 |  | 0.025 | 0.002 | 24.0 | 20.0 | 5.9 |
| ENSTGUG00000017343 | PTP4A2 | 0.025 | 0.002 | 24.0 | 12.0 | 5.9 |
| ENSTGUG00000007797 |  | 0.025 | 0.002 | 24.0 | 8.0 | 5.8 |
| ENSTGUG00000010384 | ERO1LB | 0.025 | 0.002 | 24.0 | 2.0 | 5.8 |
| ENSTGUG00000000724 | TPM2 | 0.025 | 0.002 | 24.0 | 2.0 | 5.7 |
| ENSTGUG00000016660 |  | 0.025 | 0.002 | 24.0 | 1.0 | 5.6 |
| ENSTGUG00000004587 | C16orf61 | 0.025 | 0.002 | 24.0 | 16.0 | 5.5 |
| ENSTGUG00000012229 | SSPN | 0.025 | 0.002 | 24.0 | 8.0 | 5.5 |
| ENSTGUG00000009270 | FHIT | 0.025 | 0.002 | 24.0 | 2.0 | 5.5 |
| ENSTGUG00000002929 | POLR2B | 0.025 | 0.002 | 24.0 | 18.0 | 5.5 |
| ENSTGUG00000017315 | RXRG | 0.025 | 0.002 | 24.0 | 10.0 | 5.5 |
| ENSTGUG00000006271 | DEK | 0.025 | 0.002 | 26.0 | 18.0 | 5.4 |
| ENSTGUG00000004306 | RABEP1 | 0.025 | 0.002 | 24.0 | 12.0 | 5.4 |
| ENSTGUG00000007088 | NLE1 | 0.025 | 0.002 | 26.0 | 5.0 | 5.3 |
| ENSTGUG00000011451 | METTL10 | 0.025 | 0.002 | 25.3 | 16.7 | 5.3 |
| ENSTGUG00000007518 | ARFGAP1 | 0.025 | 0.002 | 24.0 | 20.0 | 5.3 |
| ENSTGUG00000014568 | MATR3 | 0.025 | 0.002 | 24.0 | 13.0 | 5.2 |
| ENSTGUG00000005680 | ACD | 0.025 | 0.002 | 24.0 | 21.0 | 5.2 |
| ENSTGUG00000008252 | ADSS | 0.025 | 0.002 | 24.0 | 20.0 | 5.1 |
| ENSTGUG00000003101 | FZD9 | 0.025 | 0.002 | 24.0 | 8.0 | 5.1 |
| ENSTGUG00000010859 | TPP2 | 0.025 | 0.002 | 24.0 | 20.0 | 5.1 |
| ENSTGUG00000006969 | PLA2G15 | 0.025 | 0.002 | 25.3 | 4.0 | 5.1 |
| ENSTGUG00000003302 | PGD | 0.025 | 0.002 | 24.0 | 20.0 | 5.0 |
| ENSTGUG00000013213 | CD2AP | 0.025 | 0.002 | 26.0 | 15.0 | 5.0 |
| ENSTGUG00000005705 | PTCD2 | 0.025 | 0.002 | 26.0 | 18.0 | 5.0 |
| ENSTGUG00000002228 | EHBP1 | 0.025 | 0.002 | 26.0 | 2.0 | 5.0 |
| ENSTGUG00000000683 | CETN3 | 0.025 | 0.002 | 24.0 | 16.0 | 5.0 |
| ENSTGUG00000013362 | NAT13 | 0.025 | 0.002 | 24.0 | 13.0 | 5.0 |
| ENSTGUG00000001298 | VIM | 0.025 | 0.002 | 24.0 | 10.0 | 4.9 |
| ENSTGUG00000007737 | C5orf49 | 0.025 | 0.002 | 24.0 | 8.0 | 4.9 |
| ENSTGUG00000011904 | ZEB2 | 0.025 | 0.002 | 24.0 | 8.0 | 4.9 |
| ENSTGUG00000002880 | PANK4 | 0.025 | 0.002 | 24.0 | 14.0 | 4.9 |
| ENSTGUG00000008840 | LIAS | 0.025 | 0.002 | 24.0 | 20.0 | 4.8 |
| ENSTGUG00000009193 | TBC1D19 | 0.025 | 0.002 | 26.0 | 18.0 | 4.8 |
| ENSTGUG00000011545 | CHRM2 | 0.025 | 0.002 | 24.0 | 22.0 | 4.8 |
| ENSTGUG00000011083 | KIAA1033 | 0.025 | 0.002 | 24.0 | 14.0 | 4.8 |
| ENSTGUG00000011851 | FAM118A | 0.025 | 0.002 | 26.0 | 15.0 | 4.7 |
| ENSTGUG00000009519 | IL21R | 0.025 | 0.002 | 24.0 | 0.0 | 4.6 |
| ENSTGUG00000015812 | AKAP8 | 0.025 | 0.002 | 24.0 | 12.0 | 4.6 |
| ENSTGUG00000011211 | MLF1 | 0.025 | 0.002 | 24.0 | 13.0 | 4.6 |
| ENSTGUG00000004925 | CLIC2 | 0.025 | 0.002 | 24.0 | 8.0 | 4.6 |
| ENSTGUG00000012942 | ESR2 | 0.025 | 0.002 | 24.0 | 13.0 | 4.6 |
| ENSTGUG00000001010 | DAPK3 | 0.025 | 0.002 | 24.0 | 1.0 | 4.5 |
| ENSTGUG00000011713 | E2F5 | 0.025 | 0.002 | 24.0 | 16.0 | 4.5 |
| ENSTGUG00000001360 | C9orf82 | 0.025 | 0.002 | 24.0 | 20.0 | 4.5 |
| ENSTGUG00000013556 | GABPA | 0.025 | 0.002 | 24.0 | 20.0 | 4.4 |
| ENSTGUG00000005875 | ANKRA2 | 0.025 | 0.002 | 25.3 | 16.7 | 4.4 |
| ENSTGUG00000009913 | TMEM128 | 0.025 | 0.002 | 26.0 | 18.0 | 4.4 |
| ENSTGUG00000006931 | SLC12A7 | 0.025 | 0.002 | 26.0 | 18.0 | 4.4 |
| ENSTGUG00000015581 | AP1B1 | 0.025 | 0.002 | 24.0 | 2.0 | 4.3 |
| ENSTGUG00000009318 | C16orf62 | 0.025 | 0.002 | 26.0 | 18.0 | 4.3 |
| ENSTGUG00000000143 | ZNF259 | 0.025 | 0.002 | 26.0 | 5.0 | 4.2 |
| ENSTGUG00000002862 | ESRRG | 0.025 | 0.002 | 24.0 | 14.0 | 4.2 |
| ENSTGUG00000001030 | PGPEP1 | 0.025 | 0.002 | 24.0 | 12.0 | 4.2 |
| ENSTGUG00000012053 | CDC2L6 | 0.025 | 0.002 | 24.0 | 14.0 | 4.2 |
| ENSTGUG00000000398 | THY1 | 0.025 | 0.002 | 24.0 | 9.0 | 4.2 |
| ENSTGUG00000018151 |  | 0.025 | 0.002 | 24.0 | 0.0 | 4.1 |
| ENSTGUG00000012002 | BRMS1L | 0.025 | 0.002 | 24.0 | 14.0 | 4.1 |
| ENSTGUG00000011438 | KIAA0247 | 0.025 | 0.002 | 26.0 | 5.0 | 4.1 |
| ENSTGUG00000003471 | DNAJC12 | 0.025 | 0.002 | 24.0 | 22.0 | 4.1 |
| ENSTGUG00000012334 | GTF2F2 | 0.025 | 0.002 | 26.0 | 18.0 | 4.1 |
| ENSTGUG00000008185 | METTL8 | 0.025 | 0.002 | 24.0 | 16.0 | 4.1 |
| ENSTGUG00000007238 | GOLGA1 | 0.025 | 0.002 | 26.0 | 18.0 | 4.1 |
| ENSTGUG00000009450 | RNMT | 0.025 | 0.002 | 24.0 | 20.0 | 4.1 |
| ENSTGUG00000004646 | C7orf58 | 0.025 | 0.002 | 24.0 | 0.0 | 4.1 |
| ENSTGUG00000011594 | VNN2 | 0.025 | 0.002 | 24.0 | 2.0 | 4.1 |
| ENSTGUG00000004153 | ZER1 | 0.025 | 0.002 | 24.0 | 0.0 | 4.1 |
| ENSTGUG00000000501 | CDC14B | 0.025 | 0.002 | 24.0 | 14.0 | 4.1 |
| ENSTGUG00000008500 | TBL1X | 0.025 | 0.002 | 24.0 | 10.0 | 4.0 |
| ENSTGUG00000000171 | RNF14 | 0.025 | 0.002 | 24.0 | 8.0 | 4.0 |
| ENSTGUG00000012236 | NUDT15 | 0.025 | 0.002 | 26.0 | 2.0 | 4.0 |
| ENSTGUG00000007331 | DTWD1 | 0.025 | 0.002 | 24.0 | 20.0 | 4.0 |
| ENSTGUG00000009245 | RBPJ | 0.025 | 0.002 | 24.0 | 0.0 | 3.9 |
| ENSTGUG00000008677 | KCNJ11 | 0.025 | 0.002 | 26.0 | 15.0 | 3.9 |
| ENSTGUG00000004833 | ZNF511 | 0.025 | 0.002 | 24.0 | 0.0 | 3.9 |
| ENSTGUG00000010155 | MGAT4A | 0.025 | 0.002 | 26.0 | 2.0 | 3.9 |
| ENSTGUG00000009099 | CARS | 0.025 | 0.002 | 24.0 | 21.0 | 3.9 |
| ENSTGUG00000009155 | CUL4A | 0.025 | 0.002 | 24.0 | 18.0 | 3.8 |
| ENSTGUG00000000846 | FAM40A | 0.025 | 0.002 | 24.0 | 2.0 | 3.8 |
| ENSTGUG00000004137 | SLC16A6 | 0.025 | 0.002 | 24.0 | 16.0 | 3.8 |
| ENSTGUG00000000814 | FOXRED1 | 0.025 | 0.002 | 24.0 | 2.0 | 3.8 |
| ENSTGUG00000013134 | FRMD6 | 0.025 | 0.002 | 24.0 | 12.0 | 3.8 |
| ENSTGUG00000005109 | VCAM1 | 0.025 | 0.002 | 24.0 | 10.0 | 3.8 |
| ENSTGUG00000004889 | SNN | 0.025 | 0.002 | 24.0 | 0.0 | 3.7 |
| ENSTGUG00000010795 | LIG4 | 0.025 | 0.002 | 24.0 | 20.0 | 3.7 |
| ENSTGUG00000000892 | FAM174A | 0.025 | 0.002 | 24.0 | 21.0 | 3.7 |
| ENSTGUG00000006897 | FAM149A | 0.025 | 0.002 | 26.0 | 2.0 | 3.7 |
| ENSTGUG00000008848 | CHN1 | 0.025 | 0.002 | 24.0 | 16.0 | 3.7 |
| ENSTGUG00000004963 | PIGT | 0.025 | 0.002 | 24.0 | 0.0 | 3.6 |
| ENSTGUG00000003488 | DNAJB14 | 0.025 | 0.002 | 24.0 | 16.0 | 3.6 |
| ENSTGUG00000014504 | GINS2 | 0.025 | 0.002 | 24.0 | 12.0 | 3.6 |
| ENSTGUG00000007677 | GPR64 | 0.025 | 0.002 | 26.0 | 15.0 | 3.6 |
| ENSTGUG00000003722 | STAG2 | 0.025 | 0.002 | 24.0 | 10.0 | 3.6 |
| ENSTGUG00000007428 | RALGPS1 | 0.025 | 0.002 | 24.0 | 13.0 | 3.6 |
| ENSTGUG00000004447 | B5FZD1_TAEGU | 0.025 | 0.002 | 24.0 | 2.0 | 3.6 |
| ENSTGUG00000002111 | ARHGEF6 | 0.025 | 0.002 | 24.0 | 10.0 | 3.6 |
| ENSTGUG00000010869 | CASP7 | 0.025 | 0.002 | 24.0 | 16.0 | 3.5 |
| ENSTGUG00000004130 | MGRN1 | 0.025 | 0.002 | 24.0 | 0.0 | 3.5 |
| ENSTGUG00000004454 | IFT52 | 0.025 | 0.002 | 24.0 | 12.0 | 3.5 |
| ENSTGUG00000009871 | HS3ST1 | 0.025 | 0.002 | 24.0 | 2.0 | 3.5 |
| ENSTGUG00000001281 | LZTR1 | 0.025 | 0.002 | 25.3 | 16.7 | 3.5 |
| ENSTGUG00000008540 | CHD2 | 0.025 | 0.002 | 24.0 | 6.0 | 3.5 |
| ENSTGUG00000013246 | CLIC5 | 0.025 | 0.002 | 24.0 | 13.0 | 3.5 |
| ENSTGUG00000002090 | C4orf29 | 0.025 | 0.002 | 24.0 | 12.0 | 3.4 |
| ENSTGUG00000004006 | RAF1 | 0.025 | 0.002 | 24.0 | 1.0 | 3.4 |
| ENSTGUG00000010363 | JOSD1 | 0.025 | 0.002 | 24.0 | 8.0 | 3.4 |
| ENSTGUG00000007274 | RABEPK | 0.025 | 0.002 | 24.0 | 4.0 | 3.4 |
| ENSTGUG00000008017 | RPRD1A | 0.025 | 0.002 | 24.0 | 12.0 | 3.4 |
| ENSTGUG00000007400 |  | 0.025 | 0.002 | 24.0 | 14.0 | 3.3 |
| ENSTGUG00000014872 |  | 0.025 | 0.002 | 26.0 | 5.0 | 3.3 |
| ENSTGUG00000004317 |  | 0.025 | 0.002 | 24.0 | 13.0 | 3.3 |
| ENSTGUG00000008577 | HDHD1A | 0.025 | 0.002 | 24.0 | 13.0 | 3.3 |
| ENSTGUG00000000760 | ESAM | 0.025 | 0.002 | 24.0 | 5.0 | 3.2 |
| ENSTGUG00000004011 | VBP1 | 0.025 | 0.002 | 24.0 | 18.0 | 3.2 |
| ENSTGUG00000010934 | HECA | 0.025 | 0.002 | 24.0 | 12.0 | 3.2 |
| ENSTGUG00000014183 | CSNK1G2 | 0.025 | 0.002 | 26.0 | 5.0 | 3.2 |
| ENSTGUG00000012163 | ARNTL2 | 0.025 | 0.002 | 25.3 | 16.7 | 3.2 |
| ENSTGUG00000001510 | PANK3 | 0.025 | 0.002 | 24.0 | 0.0 | 3.2 |
| ENSTGUG00000006704 | SENP2 | 0.025 | 0.002 | 25.3 | 16.7 | 3.2 |
| ENSTGUG00000014103 |  | 0.025 | 0.002 | 24.0 | 0.0 | 3.2 |
| ENSTGUG00000011983 | NFKBIA | 0.025 | 0.002 | 24.0 | 14.0 | 3.1 |
| ENSTGUG00000000001 | C2CD2L | 0.025 | 0.002 | 24.0 | 2.0 | 3.1 |
| ENSTGUG00000002544 | ENTPD8 | 0.025 | 0.002 | 24.0 | 2.0 | 3.1 |
| ENSTGUG00000000624 | ZCCHC6 | 0.025 | 0.002 | 24.0 | 20.0 | 3.1 |
| ENSTGUG00000012249 | ENY2 | 0.025 | 0.002 | 24.0 | 16.0 | 3.1 |
| ENSTGUG00000004952 | ZDHHC3 | 0.025 | 0.002 | 24.0 | 14.0 | 3.1 |
| ENSTGUG00000004230 | LIN9 | 0.025 | 0.002 | 24.0 | 10.0 | 3.1 |
| ENSTGUG00000010518 | RNF4 | 0.025 | 0.002 | 24.0 | 17.0 | 3.1 |
| ENSTGUG00000004867 | SOCS1 | 0.025 | 0.002 | 24.0 | 4.0 | 3.1 |
| ENSTGUG00000004699 | CCDC34 | 0.025 | 0.002 | 24.0 | 18.0 | 3.1 |
| ENSTGUG00000011949 | C8orf38 | 0.025 | 0.002 | 24.0 | 21.0 | 3.1 |
| ENSTGUG00000007193 | OLFML2A | 0.025 | 0.002 | 24.0 | 2.0 | 3.0 |
| ENSTGUG00000007171 | FLII | 0.025 | 0.002 | 24.0 | 2.0 | 3.0 |
| ENSTGUG00000016519 | DOCK1 | 0.025 | 0.002 | 24.0 | 1.0 | 3.0 |
| ENSTGUG00000017294 | FMO4 | 0.025 | 0.002 | 24.0 | 12.0 | 2.9 |
| ENSTGUG00000008108 |  | 0.025 | 0.002 | 24.0 | 14.0 | 2.9 |
| ENSTGUG00000014355 |  | 0.025 | 0.002 | 24.0 | 6.0 | 2.9 |
| ENSTGUG00000016987 |  | 0.025 | 0.002 | 24.0 | 13.0 | 2.9 |
| ENSTGUG00000010820 | VPS24 | 0.025 | 0.002 | 24.0 | 6.0 | 2.8 |
| ENSTGUG00000000913 | CCNY | 0.025 | 0.002 | 24.0 | 14.0 | 2.8 |
| ENSTGUG00000006876 | PSMF1 | 0.025 | 0.002 | 24.0 | 6.0 | 2.8 |
| ENSTGUG00000005957 | SLC38A7 | 0.025 | 0.002 | 24.0 | 1.0 | 2.7 |
| ENSTGUG00000003560 | CMC1 | 0.025 | 0.002 | 24.0 | 14.0 | 2.7 |
| ENSTGUG00000000146 | PIGG | 0.025 | 0.002 | 24.0 | 16.0 | 2.7 |
| ENSTGUG00000005514 | POLB | 0.025 | 0.002 | 24.0 | 14.0 | 2.7 |
| ENSTGUG00000010133 | MRPL30 | 0.025 | 0.002 | 24.0 | 20.0 | 2.6 |
| ENSTGUG00000007559 | ADRBK2 | 0.025 | 0.002 | 24.0 | 12.0 | 2.6 |
| ENSTGUG00000004286 | NXT2 | 0.025 | 0.002 | 24.0 | 18.0 | 2.6 |
| ENSTGUG00000009258 | TSSC4 | 0.025 | 0.002 | 24.0 | 20.0 | 2.6 |
| ENSTGUG00000014248 | CHN2 | 0.025 | 0.002 | 24.0 | 2.0 | 2.6 |
| ENSTGUG00000003701 |  | 0.025 | 0.002 | 24.0 | 9.0 | 2.6 |
| ENSTGUG00000010354 | OBFC1 | 0.025 | 0.002 | 24.0 | 14.0 | 2.6 |
| ENSTGUG00000015937 |  | 0.025 | 0.002 | 24.0 | 10.0 | 2.6 |
| ENSTGUG00000013622 | TXNL4B | 0.025 | 0.002 | 24.0 | 14.0 | 2.6 |
| ENSTGUG00000006886 | SIRT3 | 0.025 | 0.002 | 24.0 | 5.0 | 2.6 |
| ENSTGUG00000000889 | PITRM1 | 0.025 | 0.002 | 26.0 | 18.0 | 2.6 |
| ENSTGUG00000005436 | CDK2AP1 | 0.025 | 0.002 | 24.0 | 13.0 | 2.5 |
| ENSTGUG00000005631 | CBWD3 | 0.025 | 0.002 | 25.3 | 16.7 | 2.5 |
| ENSTGUG00000003748 | SMG5 | 0.025 | 0.002 | 24.0 | 8.0 | 2.5 |
| ENSTGUG00000001191 | PIP4K2A | 0.025 | 0.002 | 24.0 | 10.0 | 2.5 |
| ENSTGUG00000010216 | C10orf26 | 0.025 | 0.002 | 24.0 | 8.0 | 2.5 |
| ENSTGUG00000006144 |  | 0.025 | 0.002 | 24.0 | 14.0 | 2.5 |
| ENSTGUG00000009665 | NFAM1 | 0.025 | 0.002 | 24.0 | 12.0 | 2.5 |
| ENSTGUG00000011508 | PIGH | 0.025 | 0.002 | 24.0 | 21.0 | 2.5 |
| ENSTGUG00000004645 | HYAL2 | 0.025 | 0.002 | 24.0 | 4.0 | 2.5 |
| ENSTGUG00000015286 |  | 0.025 | 0.002 | 24.0 | 12.0 | 2.5 |
| ENSTGUG00000007850 | EFTUD1 | 0.025 | 0.002 | 24.0 | 18.0 | 2.5 |
| ENSTGUG00000008122 | PIK3R3 | 0.025 | 0.002 | 24.0 | 12.0 | 2.4 |
| ENSTGUG00000005182 | ATP6V0A2 | 0.025 | 0.002 | 24.0 | 18.0 | 2.4 |
| ENSTGUG00000010023 | CREB1 | 0.025 | 0.002 | 26.0 | 2.0 | 2.4 |
| ENSTGUG00000012242 | TMEM74 | 0.025 | 0.002 | 24.0 | 10.0 | 2.4 |
| ENSTGUG00000005329 | GTF3C5 | 0.025 | 0.002 | 24.0 | 1.0 | 2.4 |
| ENSTGUG00000005780 | FCHO2 | 0.025 | 0.002 | 24.0 | 10.0 | 2.3 |
| ENSTGUG00000001606 | THRAP3 | 0.025 | 0.002 | 24.0 | 0.0 | 2.3 |
| ENSTGUG00000012381 | DEPDC6-2 | 0.025 | 0.002 | 24.0 | 10.0 | 2.3 |
| ENSTGUG00000008953 | POP4 | 0.025 | 0.002 | 26.0 | 18.0 | 2.3 |
| ENSTGUG00000013544 | NRIP1 | 0.025 | 0.002 | 24.0 | 14.0 | 2.3 |
| ENSTGUG00000008701 | PHKB | 0.025 | 0.002 | 24.0 | 20.0 | 2.3 |
| ENSTGUG00000002064 | SPEN | 0.025 | 0.002 | 26.0 | 2.0 | 2.3 |
| ENSTGUG00000008523 | LLGL2 | 0.025 | 0.002 | 24.0 | 13.0 | 2.3 |
| ENSTGUG00000006360 | NEDD4-1 | 0.025 | 0.002 | 24.0 | 13.0 | 2.2 |
| ENSTGUG00000014061 |  | 0.025 | 0.002 | 24.0 | 22.0 | 2.2 |
| ENSTGUG00000001938 | EXOSC9 | 0.025 | 0.002 | 24.0 | 18.0 | 2.2 |
| ENSTGUG00000002923 | C4orf14 | 0.025 | 0.002 | 24.0 | 17.0 | 2.2 |
| ENSTGUG00000003387 | KPNB1 | 0.025 | 0.002 | 26.0 | 2.0 | 2.2 |
| ENSTGUG00000012008 | FOXO1A | 0.025 | 0.002 | 24.0 | 10.0 | 2.2 |
| ENSTGUG00000018153 |  | 0.025 | 0.002 | 26.0 | 5.0 | 2.2 |
| ENSTGUG00000011708 | USPL1 | 0.025 | 0.002 | 24.0 | 18.0 | 2.2 |
| ENSTGUG00000001963 | WDR68 | 0.025 | 0.002 | 24.0 | 0.0 | 2.2 |
| ENSTGUG00000001614 |  | 0.025 | 0.002 | 24.0 | 4.0 | 2.1 |
| ENSTGUG00000014898 | KCNMB1 | 0.025 | 0.002 | 24.0 | 12.0 | 2.1 |
| ENSTGUG00000016584 | DCTN1 | 0.025 | 0.002 | 24.0 | 0.0 | 2.1 |
| ENSTGUG00000009573 | PACRGL | 0.025 | 0.002 | 24.0 | 18.0 | 2.1 |
| ENSTGUG00000013110 | SMC6 | 0.025 | 0.002 | 24.0 | 14.0 | 2.1 |
| ENSTGUG00000001988 | PIGN | 0.025 | 0.002 | 24.0 | 2.0 | 2.1 |
| ENSTGUG00000006914 | IFIH1 | 0.025 | 0.002 | 24.0 | 14.0 | 2.0 |
| ENSTGUG00000006457 | SYDE2 | 0.025 | 0.002 | 24.0 | 22.0 | 2.0 |
| ENSTGUG00000003639 | RCN2 | 0.025 | 0.002 | 24.0 | 21.0 | 2.0 |
| ENSTGUG00000011582 | CREB3L2 | 0.025 | 0.002 | 26.0 | 2.0 | 2.0 |
| ENSTGUG00000005079 | SMARCAL1 | 0.025 | 0.002 | 24.0 | 14.0 | 2.0 |
| ENSTGUG00000003233 | CXorf57 | 0.025 | 0.002 | 24.0 | 1.0 | 2.0 |
| ENSTGUG00000003186 | RCOR3 | 0.025 | 0.002 | 24.0 | 13.0 | 2.0 |
| ENSTGUG00000002045 | NIPBL-2 | 0.025 | 0.002 | 24.0 | 22.0 | 2.0 |
| ENSTGUG00000010698 |  | 0.025 | 0.002 | 24.0 | 17.0 | 2.0 |
| ENSTGUG00000008168 | SNX8 | 0.025 | 0.002 | 24.0 | 0.0 | 2.0 |
| ENSTGUG00000015804 |  | 0.025 | 0.002 | 24.0 | 4.0 | 2.0 |
| ENSTGUG00000015561 |  | 0.025 | 0.002 | 24.0 | 0.0 | 2.0 |
| ENSTGUG00000002730 | KIF2A | 0.025 | 0.002 | 24.0 | 6.0 | 1.9 |
| ENSTGUG00000009459 | CERKL | 0.025 | 0.002 | 24.0 | 20.0 | 1.9 |
| ENSTGUG00000005820 | FHOD1 | 0.025 | 0.002 | 24.0 | 2.0 | 1.9 |
| ENSTGUG00000010907 | TMTC4 | 0.025 | 0.002 | 24.0 | 14.0 | 1.9 |
| ENSTGUG00000007066 | MSH3-1 | 0.025 | 0.002 | 26.0 | 18.0 | 1.9 |
| ENSTGUG00000012973 | TSSC1 | 0.025 | 0.002 | 24.0 | 14.0 | 1.9 |
| ENSTGUG00000008684 | EXOC3 | 0.025 | 0.002 | 24.0 | 13.0 | 1.9 |
| ENSTGUG00000007672 | WHDC1 | 0.025 | 0.002 | 24.0 | 20.0 | 1.8 |
| ENSTGUG00000005849 | GLB1L | 0.025 | 0.002 | 26.0 | 5.0 | 1.8 |
| ENSTGUG00000000175 | TAGLN | 0.025 | 0.002 | 26.0 | 5.0 | 1.8 |
| ENSTGUG00000017434 |  | 0.025 | 0.002 | 24.0 | 10.0 | 1.8 |
| ENSTGUG00000006348 | TEX9 | 0.025 | 0.002 | 24.0 | 20.0 | 1.8 |
| ENSTGUG00000007087 |  | 0.025 | 0.002 | 24.0 | 21.0 | 1.8 |
| ENSTGUG00000008029 | TMTC3 | 0.025 | 0.002 | 24.0 | 20.0 | 1.8 |
| ENSTGUG00000002062 |  | 0.025 | 0.002 | 26.0 | 2.0 | 1.8 |
| ENSTGUG00000016452 |  | 0.025 | 0.002 | 24.0 | 10.0 | 1.8 |
| ENSTGUG00000002708 | HLX | 0.025 | 0.002 | 24.0 | 2.0 | 1.7 |
| ENSTGUG00000003022 | GOLM1 | 0.025 | 0.002 | 24.0 | 16.0 | 1.7 |
| ENSTGUG00000009177 | STIM2 | 0.025 | 0.002 | 26.0 | 2.0 | 1.7 |
| ENSTGUG00000000469 | HICE1 | 0.025 | 0.002 | 24.0 | 16.0 | 1.7 |
| ENSTGUG00000011523 | RDH11 | 0.025 | 0.002 | 24.0 | 0.0 | 1.7 |
| ENSTGUG00000004773 |  | 0.025 | 0.002 | 24.0 | 16.0 | 1.7 |
| ENSTGUG00000007135 | TPD52L2 | 0.025 | 0.002 | 24.0 | 18.0 | 1.7 |
| ENSTGUG00000011584 | GTF3A | 0.025 | 0.002 | 24.0 | 6.0 | 1.7 |
| ENSTGUG00000005149 | UBE2D1 | 0.025 | 0.002 | 24.0 | 13.0 | 1.7 |
| ENSTGUG00000005775 | RPA1 | 0.025 | 0.002 | 24.0 | 14.0 | 1.7 |
| ENSTGUG00000004256 | DOLK | 0.025 | 0.002 | 24.0 | 16.0 | 1.7 |
| ENSTGUG00000006749 | MYO5C | 0.025 | 0.002 | 24.0 | 18.0 | 1.7 |
| ENSTGUG00000001459 | KRIT1 | 0.025 | 0.002 | 24.0 | 4.0 | 1.7 |
| ENSTGUG00000009239 | SPAG9 | 0.025 | 0.002 | 24.0 | 16.0 | 1.7 |
| ENSTGUG00000007708 | ZFPM1 | 0.025 | 0.002 | 24.0 | 8.0 | 1.7 |
| ENSTGUG00000007437 | FAM105A | 0.025 | 0.002 | 24.0 | 12.0 | 1.7 |
| ENSTGUG00000008047 | TH1L | 0.025 | 0.002 | 24.0 | 13.0 | 1.6 |
| ENSTGUG00000009797 | NRP2 | 0.025 | 0.002 | 24.0 | 6.0 | 1.6 |
| ENSTGUG00000004516 | XCR1 | 0.025 | 0.002 | 24.0 | 12.0 | 1.6 |
| ENSTGUG00000006988 | TAF1 | 0.025 | 0.002 | 24.0 | 20.0 | 1.6 |
| ENSTGUG00000001586 | PSTPIP2 | 0.025 | 0.002 | 24.0 | 9.0 | 1.6 |
| ENSTGUG00000008865 |  | 0.025 | 0.002 | 24.0 | 4.0 | 1.6 |
| ENSTGUG00000005014 | XRCC5 | 0.025 | 0.002 | 24.0 | 21.0 | 1.6 |
| ENSTGUG00000006841 | FUBP1 | 0.025 | 0.002 | 24.0 | 0.0 | 1.6 |
| ENSTGUG00000009733 | UBA3 | 0.025 | 0.002 | 24.0 | 14.0 | 1.6 |
| ENSTGUG00000010112 | DVL3 | 0.025 | 0.002 | 24.0 | 2.0 | 1.6 |
| ENSTGUG00000011566 | LY96 | 0.025 | 0.002 | 24.0 | 8.0 | 1.6 |
| ENSTGUG00000002510 | RPS6KA6 | 0.025 | 0.002 | 24.0 | 1.0 | 1.6 |
| ENSTGUG00000008681 | PTPN1 | 0.025 | 0.002 | 24.0 | 2.0 | 1.6 |
| ENSTGUG00000001027 | LSM4 | 0.025 | 0.002 | 24.0 | 10.0 | 1.5 |
| ENSTGUG00000000164 | SETD2 | 0.025 | 0.002 | 24.0 | 2.0 | 1.5 |
| ENSTGUG00000004484 | LRWD1 | 0.025 | 0.002 | 24.0 | 20.0 | 1.5 |
| ENSTGUG00000015964 |  | 0.025 | 0.002 | 24.0 | 0.0 | 1.5 |
| ENSTGUG00000001066 | APBA1 | 0.025 | 0.002 | 24.0 | 1.0 | 1.5 |
| ENSTGUG00000001579 | CCDC5 | 0.025 | 0.002 | 24.0 | 12.0 | 1.5 |
| ENSTGUG00000001985 | NECAP2 | 0.025 | 0.002 | 24.0 | 12.0 | 1.5 |
| ENSTGUG00000003828 | CIZ1 | 0.025 | 0.002 | 24.0 | 1.0 | 1.5 |
| ENSTGUG00000007321 | UBE3B | 0.025 | 0.002 | 24.0 | 8.0 | 1.5 |
| ENSTGUG00000010623 | TMEM129 | 0.025 | 0.002 | 24.0 | 13.0 | 1.5 |
| ENSTGUG00000003503 | GCNT1 | 0.025 | 0.002 | 26.0 | 15.0 | 1.5 |
| ENSTGUG00000008280 | TMEM43 | 0.025 | 0.002 | 24.0 | 13.0 | 1.5 |
| ENSTGUG00000012978 | HIF1A | 0.025 | 0.002 | 24.0 | 0.0 | 1.5 |
| ENSTGUG00000003412 | DIP2B | 0.025 | 0.002 | 26.0 | 5.0 | 1.4 |
| ENSTGUG00000008901 | RFC1 | 0.025 | 0.002 | 24.0 | 12.0 | 1.4 |
| ENSTGUG00000006124 | NEK1 | 0.025 | 0.002 | 24.0 | 16.0 | 1.4 |
| ENSTGUG00000015952 |  | 0.025 | 0.002 | 24.0 | 22.0 | 1.4 |
| ENSTGUG00000016131 | USP47 | 0.025 | 0.002 | 24.0 | 1.0 | 1.4 |
| ENSTGUG00000009711 | RRP12 | 0.025 | 0.002 | 24.0 | 0.0 | 1.4 |
| ENSTGUG00000015396 |  | 0.025 | 0.002 | 24.0 | 10.0 | 1.4 |
| ENSTGUG00000013189 | C2orf44 | 0.025 | 0.002 | 26.0 | 15.0 | 1.4 |
| ENSTGUG00000001909 | ZFR | 0.025 | 0.002 | 24.0 | 1.0 | 1.4 |
| ENSTGUG00000010777 | DLL4 | 0.025 | 0.002 | 24.0 | 9.0 | 1.4 |
| ENSTGUG00000008619 | ELK3 | 0.025 | 0.002 | 24.0 | 9.0 | 1.4 |
| ENSTGUG00000014169 |  | 0.025 | 0.002 | 24.0 | 2.0 | 1.4 |
| ENSTGUG00000000274 | BTBD2 | 0.025 | 0.002 | 24.0 | 4.0 | 1.3 |
| ENSTGUG00000007222 | CEP152 | 0.025 | 0.002 | 26.0 | 15.0 | 1.3 |
| ENSTGUG00000012585 | RBM26 | 0.025 | 0.002 | 24.0 | 6.0 | 1.3 |
| ENSTGUG00000002837 |  | 0.025 | 0.002 | 24.0 | 20.0 | 1.3 |
| ENSTGUG00000004958 | CRLF3 | 0.025 | 0.002 | 24.0 | 12.0 | 1.3 |
| ENSTGUG00000005318 | SLC35A3 | 0.025 | 0.002 | 24.0 | 9.0 | 1.3 |
| ENSTGUG00000010759 | ARSD-1 | 0.025 | 0.002 | 24.0 | 22.0 | 1.3 |
| ENSTGUG00000009086 | TPCN1 | 0.025 | 0.002 | 24.0 | 9.0 | 1.3 |
| ENSTGUG00000001522 | COL1A2 | 0.025 | 0.002 | 24.0 | 8.0 | 1.3 |
| ENSTGUG00000012227 | HACE1 | 0.025 | 0.002 | 26.0 | 18.0 | 1.3 |
| ENSTGUG00000002037 | PRKCQ | 0.025 | 0.002 | 24.0 | 12.0 | 1.3 |
| ENSTGUG00000011984 | MMADHC | 0.025 | 0.002 | 24.0 | 6.0 | 1.3 |
| ENSTGUG00000007473 | ANKRD13A | 0.025 | 0.002 | 24.0 | 12.0 | 1.3 |
| ENSTGUG00000004965 | SHF | 0.025 | 0.002 | 24.0 | 8.0 | 1.3 |
| ENSTGUG00000014462 |  | 0.025 | 0.002 | 24.0 | 22.0 | 1.3 |
| ENSTGUG00000001922 | MYL4 | 0.025 | 0.002 | 24.0 | 8.0 | 1.3 |
| ENSTGUG00000007204 | C1orf84 | 0.025 | 0.002 | 24.0 | 1.0 | 1.2 |
| ENSTGUG00000013903 | ATP13A1 | 0.025 | 0.002 | 24.0 | 0.0 | 1.2 |
| ENSTGUG00000000119 | SMARCC1 | 0.025 | 0.002 | 26.0 | 5.0 | 1.2 |
| ENSTGUG00000012260 | VASH1 | 0.025 | 0.002 | 24.0 | 8.0 | 1.2 |
| ENSTGUG00000003878 | SRBD1 | 0.025 | 0.002 | 24.0 | 20.0 | 1.2 |
| ENSTGUG00000009220 | DENND4A | 0.025 | 0.002 | 24.0 | 18.0 | 1.2 |
| ENSTGUG00000011529 | RNF6 | 0.025 | 0.002 | 24.0 | 18.0 | 1.2 |
| ENSTGUG00000012264 | ANGEL1 | 0.025 | 0.002 | 26.0 | 15.0 | 1.2 |
| ENSTGUG00000006499 | PIGB | 0.025 | 0.002 | 24.0 | 14.0 | 1.2 |
| ENSTGUG00000007006 | NFATC3 | 0.025 | 0.002 | 24.0 | 2.0 | 1.2 |
| ENSTGUG00000007455 | USP22 | 0.025 | 0.002 | 24.0 | 6.0 | 1.2 |
| ENSTGUG00000010807 | GLS | 0.025 | 0.002 | 24.0 | 12.0 | 1.2 |
| ENSTGUG00000015147 |  | 0.025 | 0.002 | 24.0 | 14.0 | 1.2 |
| ENSTGUG00000000976 | SLC9A1 | 0.025 | 0.002 | 24.0 | 4.0 | 1.2 |
| ENSTGUG00000003834 | UBAP2L | 0.025 | 0.002 | 24.0 | 1.0 | 1.2 |
| ENSTGUG00000003659 | PSTPIP1 | 0.025 | 0.002 | 24.0 | 13.0 | 1.2 |
| ENSTGUG00000011943 | DSE | 0.025 | 0.002 | 24.0 | 6.0 | 1.2 |
| ENSTGUG00000013875 |  | 0.025 | 0.002 | 24.0 | 2.0 | 1.2 |
| ENSTGUG00000011748 | MEIS2 | 0.025 | 0.002 | 24.0 | 9.0 | 1.2 |
| ENSTGUG00000004016 | PLEKHN1 | 0.025 | 0.002 | 25.3 | 16.7 | 1.2 |
| ENSTGUG00000006437 | TSC2 | 0.025 | 0.002 | 24.0 | 5.0 | 1.2 |
| ENSTGUG00000011718 |  | 0.025 | 0.002 | 24.0 | 18.0 | 1.2 |
| ENSTGUG00000008974 | FAM65B | 0.025 | 0.002 | 24.0 | 12.0 | 1.1 |
| ENSTGUG00000010717 | INPP5J | 0.025 | 0.002 | 24.0 | 4.0 | 1.1 |
| ENSTGUG00000013953 | SF4 | 0.025 | 0.002 | 24.0 | 1.0 | 1.1 |
| ENSTGUG00000000938 | HNRNPM | 0.025 | 0.002 | 24.0 | 1.0 | 1.1 |
| ENSTGUG00000013379 | GPR156 | 0.025 | 0.002 | 26.0 | 5.0 | 1.1 |
| ENSTGUG00000008104 | NFAT5 | 0.025 | 0.002 | 25.3 | 4.0 | 1.1 |
| ENSTGUG00000000079 | TCERG1 | 0.025 | 0.002 | 24.0 | 4.0 | 1.1 |
| ENSTGUG00000010277 | TSPAN18 | 0.025 | 0.002 | 24.0 | 8.0 | 1.1 |
| ENSTGUG00000001656 | ANKHD1 | 0.025 | 0.002 | 26.0 | 2.0 | 1.1 |
| ENSTGUG00000005394 | TNFSF10-1 | 0.025 | 0.002 | 25.3 | 16.7 | 1.1 |
| ENSTGUG00000000648 | ACAD8 | 0.025 | 0.002 | 24.0 | 0.0 | 1.1 |
| ENSTGUG00000006505 | PSMD5 | 0.025 | 0.002 | 24.0 | 0.0 | 1.1 |
| ENSTGUG00000005209 | GINS4 | 0.025 | 0.002 | 24.0 | 22.0 | 1.1 |
| ENSTGUG00000010856 | C13orf27 | 0.025 | 0.002 | 24.0 | 20.0 | 1.1 |
| ENSTGUG00000002671 | MMAA | 0.025 | 0.002 | 24.0 | 21.0 | 1.1 |
| ENSTGUG00000002601 | MIER3 | 0.025 | 0.002 | 24.0 | 20.0 | 1.1 |
| ENSTGUG00000001146 | RCAN3 | 0.025 | 0.002 | 26.0 | 2.0 | 1.1 |
| ENSTGUG00000011424 | TNFRSF19 | 0.025 | 0.002 | 24.0 | 8.0 | 1.1 |
| ENSTGUG00000006355 |  | 0.025 | 0.002 | 24.0 | 6.0 | 1.1 |
| ENSTGUG00000003366 |  | 0.025 | 0.002 | 24.0 | 4.0 | 1.0 |
| ENSTGUG00000004874 | SHROOM4 | 0.025 | 0.002 | 24.0 | 12.0 | 1.0 |
| ENSTGUG00000005739 | BRD3 | 0.025 | 0.002 | 24.0 | 22.0 | 1.0 |
| ENSTGUG00000002099 | HTATSF1 | 0.025 | 0.002 | 24.0 | 22.0 | 1.0 |
| ENSTGUG00000010741 | SLC35C1 | 0.025 | 0.002 | 24.0 | 18.0 | 1.0 |
| ENSTGUG00000005241 | SLC25A14 | 0.025 | 0.002 | 26.0 | 2.0 | 1.0 |
| ENSTGUG00000002053 |  | 0.025 | 0.002 | 24.0 | 10.0 | 1.0 |
| ENSTGUG00000006558 | HCK | 0.025 | 0.002 | 24.0 | 8.0 | 1.0 |
| ENSTGUG00000003908 | PRKCE | 0.025 | 0.002 | 24.0 | 2.0 | 1.0 |
| ENSTGUG00000006076 | RPAP2 | 0.025 | 0.002 | 24.0 | 20.0 | 1.0 |
| ENSTGUG00000018509 |  | 0.025 | 0.002 | 24.0 | 5.0 | 1.0 |
| ENSTGUG00000015467 |  | 0.025 | 0.002 | 24.0 | 22.0 | 1.0 |
| ENSTGUG00000011939 | MBD5 | 0.025 | 0.002 | 24.0 | 2.0 | 1.0 |
| ENSTGUG00000009477 | JMJD5 | 0.025 | 0.002 | 24.0 | 6.0 | 1.0 |
| ENSTGUG00000005153 | RBP1 | 0.025 | 0.002 | 24.0 | 13.0 | 0.9 |
| ENSTGUG00000013886 |  | 0.025 | 0.002 | 24.0 | 16.0 | 0.9 |
| ENSTGUG00000000926 | GIN1 | 0.025 | 0.002 | 24.0 | 14.0 | 0.9 |
| ENSTGUG00000003695 | SEMA4A | 0.025 | 0.002 | 24.0 | 8.0 | 0.9 |
| ENSTGUG00000001015 | APC | 0.025 | 0.002 | 24.0 | 12.0 | 0.9 |
| ENSTGUG00000006952 | TRPV1 | 0.025 | 0.002 | 24.0 | 14.0 | 0.9 |
| ENSTGUG00000000088 |  | 0.025 | 0.002 | 24.0 | 8.0 | 0.9 |
| ENSTGUG00000013575 |  | 0.025 | 0.002 | 24.0 | 1.0 | 0.9 |
| ENSTGUG00000000819 | CHAF1A | 0.025 | 0.002 | 24.0 | 13.0 | 0.9 |
| ENSTGUG00000010840 | PRKDC | 0.025 | 0.002 | 24.0 | 14.0 | 0.9 |
| ENSTGUG00000015647 |  | 0.025 | 0.002 | 24.0 | 12.0 | 0.9 |
| ENSTGUG00000015241 | MAT2A | 0.025 | 0.002 | 24.0 | 6.0 | 0.9 |
| ENSTGUG00000007472 |  | 0.025 | 0.002 | 24.0 | 5.0 | 0.9 |
| ENSTGUG00000004323 | DENND1B | 0.025 | 0.002 | 24.0 | 8.0 | 0.9 |
| ENSTGUG00000012176 | TM7SF4 | 0.025 | 0.002 | 24.0 | 13.0 | 0.8 |
| ENSTGUG00000001927 | C4orf31 | 0.025 | 0.002 | 24.0 | 10.0 | 0.8 |
| ENSTGUG00000010786 | INO80 | 0.025 | 0.002 | 24.0 | 21.0 | 0.8 |
| ENSTGUG00000014557 |  | 0.025 | 0.002 | 26.0 | 2.0 | 0.8 |
| ENSTGUG00000002110 | MYO1D | 0.025 | 0.002 | 24.0 | 1.0 | 0.8 |
| ENSTGUG00000008306 |  | 0.025 | 0.002 | 24.0 | 12.0 | 0.8 |
| ENSTGUG00000015423 |  | 0.025 | 0.002 | 24.0 | 22.0 | 0.8 |
| ENSTGUG00000010168 | ALS2 | 0.025 | 0.002 | 24.0 | 20.0 | 0.8 |
| ENSTGUG00000003718 |  | 0.025 | 0.002 | 24.0 | 13.0 | 0.8 |
| ENSTGUG00000011766 | KIAA0528 | 0.025 | 0.002 | 24.0 | 12.0 | 0.8 |
| ENSTGUG00000005270 | ARID5B | 0.025 | 0.002 | 24.0 | 22.0 | 0.8 |
| ENSTGUG00000013957 |  | 0.025 | 0.002 | 24.0 | 22.0 | 0.8 |
| ENSTGUG00000007374 | DLG3 | 0.025 | 0.002 | 24.0 | 1.0 | 0.8 |
| ENSTGUG00000011320 | FRZB | 0.025 | 0.002 | 24.0 | 5.0 | 0.8 |
| ENSTGUG00000008792 |  | 0.025 | 0.002 | 24.0 | 8.0 | 0.8 |
| ENSTGUG00000014674 |  | 0.025 | 0.002 | 24.0 | 22.0 | 0.8 |
| ENSTGUG00000001096 | RFESD | 0.025 | 0.002 | 24.0 | 16.0 | 0.8 |
| ENSTGUG00000005817 | YAF2 | 0.025 | 0.002 | 24.0 | 22.0 | 0.8 |
| ENSTGUG00000007961 | ZNF276 | 0.025 | 0.002 | 24.0 | 16.0 | 0.8 |
| ENSTGUG00000012855 |  | 0.025 | 0.002 | 24.0 | 16.0 | 0.8 |
| ENSTGUG00000002983 | FKBP14 | 0.025 | 0.002 | 24.0 | 1.0 | 0.8 |
| ENSTGUG00000002977 | RPS6KC1 | 0.025 | 0.002 | 24.0 | 1.0 | 0.8 |
| ENSTGUG00000007438 | GIT2 | 0.025 | 0.002 | 24.0 | 8.0 | 0.8 |
| ENSTGUG00000001479 | MAT2B | 0.025 | 0.002 | 24.0 | 21.0 | 0.8 |
| ENSTGUG00000001527 | CCDC99 | 0.025 | 0.002 | 24.0 | 12.0 | 0.8 |
| ENSTGUG00000005512 | KIAA0895L-1 | 0.025 | 0.002 | 26.0 | 2.0 | 0.8 |
| ENSTGUG00000009752 | TGFBRAP1 | 0.025 | 0.002 | 24.0 | 0.0 | 0.8 |
| ENSTGUG00000008691 | STRN | 0.025 | 0.002 | 24.0 | 12.0 | 0.8 |
| ENSTGUG00000003751 | RAB11FIP3 | 0.025 | 0.002 | 24.0 | 8.0 | 0.8 |
| ENSTGUG00000000784 |  | 0.025 | 0.002 | 24.0 | 4.0 | 0.8 |
| ENSTGUG00000009058 | ZNF518A | 0.025 | 0.002 | 25.3 | 16.7 | 0.8 |
| ENSTGUG00000001821 | CAMK2D | 0.025 | 0.002 | 24.0 | 10.0 | 0.8 |
| ENSTGUG00000003105 | GAA | 0.025 | 0.002 | 26.0 | 5.0 | 0.8 |
| ENSTGUG00000012632 | ASB2 | 0.025 | 0.002 | 24.0 | 13.0 | 0.7 |
| ENSTGUG00000007009 | CRB2 | 0.025 | 0.002 | 24.0 | 8.0 | 0.7 |
| ENSTGUG00000000375 |  | 0.025 | 0.002 | 24.0 | 9.0 | 0.7 |
| ENSTGUG00000011441 | TMTC1 | 0.025 | 0.002 | 24.0 | 8.0 | 0.7 |
| ENSTGUG00000008766 |  | 0.025 | 0.002 | 24.0 | 8.0 | 0.7 |
| ENSTGUG00000008964 | MED13L | 0.025 | 0.002 | 26.0 | 2.0 | 0.7 |
| ENSTGUG00000004408 |  | 0.025 | 0.002 | 24.0 | 8.0 | 0.7 |
| ENSTGUG00000010896 | TYRO3 | 0.025 | 0.002 | 24.0 | 10.0 | 0.7 |
| ENSTGUG00000004107 | RASSF2 | 0.025 | 0.002 | 24.0 | 10.0 | 0.7 |
| ENSTGUG00000002940 | SRPK2 | 0.025 | 0.002 | 25.3 | 16.7 | 0.7 |
| ENSTGUG00000005892 | SNHG8 | 0.025 | 0.002 | 24.0 | 12.0 | 0.7 |
| ENSTGUG00000004949 | GPR56 | 0.025 | 0.002 | 24.0 | 2.0 | 0.7 |
| ENSTGUG00000006938 | FAM101B | 0.025 | 0.002 | 24.0 | 12.0 | 0.7 |
| ENSTGUG00000017222 |  | 0.025 | 0.002 | 24.0 | 16.0 | 0.7 |
| ENSTGUG00000009393 | TMC5-1 | 0.025 | 0.002 | 24.0 | 12.0 | 0.7 |
| ENSTGUG00000008741 | SPATA2 | 0.025 | 0.002 | 25.3 | 4.0 | 0.7 |
| ENSTGUG00000011408 | SGCG | 0.025 | 0.002 | 24.0 | 12.0 | 0.7 |
| ENSTGUG00000009754 | C6orf70 | 0.025 | 0.002 | 24.0 | 21.0 | 0.7 |
| ENSTGUG00000015717 |  | 0.025 | 0.002 | 24.0 | 12.0 | 0.7 |
| ENSTGUG00000007954 | ZNRF3 | 0.025 | 0.002 | 24.0 | 1.0 | 0.7 |
| ENSTGUG00000004540 | KIAA0556 | 0.025 | 0.002 | 24.0 | 14.0 | 0.7 |
| ENSTGUG00000001233 | AKAP2 | 0.025 | 0.002 | 24.0 | 8.0 | 0.6 |
| ENSTGUG00000009489 | PARS2 | 0.025 | 0.002 | 26.0 | 5.0 | 0.6 |
| ENSTGUG00000012874 |  | 0.025 | 0.002 | 24.0 | 10.0 | 0.6 |
| ENSTGUG00000002588 | LPAR4 | 0.025 | 0.002 | 26.0 | 5.0 | 0.6 |
| ENSTGUG00000002456 | TASP1 | 0.025 | 0.002 | 24.0 | 14.0 | 0.6 |
| ENSTGUG00000017547 | SEMA6A | 0.025 | 0.002 | 24.0 | 2.0 | 0.6 |
| ENSTGUG00000008236 | DENND5A | 0.025 | 0.002 | 24.0 | 4.0 | 0.6 |
| ENSTGUG00000017274 | KIAA0859 | 0.025 | 0.002 | 24.0 | 0.0 | 0.6 |
| ENSTGUG00000011709 | L3MBTL3 | 0.025 | 0.002 | 24.0 | 9.0 | 0.6 |
| ENSTGUG00000009300 | IL1RAP | 0.025 | 0.002 | 26.0 | 2.0 | 0.6 |
| ENSTGUG00000013991 |  | 0.025 | 0.002 | 24.0 | 12.0 | 0.6 |
| ENSTGUG00000017530 | PPM1J | 0.025 | 0.002 | 24.0 | 13.0 | 0.6 |
| ENSTGUG00000015881 | R3HDM2 | 0.025 | 0.002 | 24.0 | 10.0 | 0.6 |
| ENSTGUG00000008583 | KIAA0195 | 0.025 | 0.002 | 24.0 | 0.0 | 0.6 |
| ENSTGUG00000011044 | EZR | 0.025 | 0.002 | 24.0 | 17.0 | 0.6 |
| ENSTGUG00000009880 | C10orf76 | 0.025 | 0.002 | 24.0 | 16.0 | 0.6 |
| ENSTGUG00000009041 | TLR1B | 0.025 | 0.002 | 24.0 | 22.0 | 0.6 |
| ENSTGUG00000000752 | CCDC15 | 0.025 | 0.002 | 25.3 | 16.7 | 0.6 |
| ENSTGUG00000003831 | AAK1 | 0.025 | 0.002 | 24.0 | 0.0 | 0.5 |
| ENSTGUG00000013554 | JAM2 | 0.025 | 0.002 | 24.0 | 10.0 | 0.5 |
| ENSTGUG00000002657 |  | 0.025 | 0.002 | 24.0 | 13.0 | 0.5 |
| ENSTGUG00000001344 | KCNN2 | 0.025 | 0.002 | 26.0 | 15.0 | 0.5 |
| ENSTGUG00000013779 | ARHGAP11A | 0.025 | 0.002 | 24.0 | 14.0 | 0.5 |
| ENSTGUG00000005581 | EPB42 | 0.025 | 0.002 | 24.0 | 16.0 | 0.5 |
| ENSTGUG00000006249 | INPP5E | 0.025 | 0.002 | 24.0 | 16.0 | 0.5 |
| ENSTGUG00000005297 | SLC11A1 | 0.025 | 0.002 | 24.0 | 4.0 | 0.5 |
| ENSTGUG00000011205 | C14orf43 | 0.025 | 0.002 | 24.0 | 22.0 | 0.5 |
| ENSTGUG00000012863 | SLC36A4 | 0.025 | 0.002 | 24.0 | 14.0 | 0.5 |
| ENSTGUG00000009668 | ZDHHC16 | 0.025 | 0.002 | 24.0 | 22.0 | 0.5 |
| ENSTGUG00000002083 | PLK4 | 0.025 | 0.002 | 24.0 | 14.0 | 0.5 |
| ENSTGUG00000012607 | ITPK1 | 0.025 | 0.002 | 24.0 | 8.0 | 0.5 |
| ENSTGUG00000013187 | EPHB6 | 0.025 | 0.002 | 24.0 | 8.0 | 0.5 |
| ENSTGUG00000003215 |  | 0.025 | 0.002 | 24.0 | 6.0 | 0.5 |
| ENSTGUG00000006796 | TPPP | 0.025 | 0.002 | 24.0 | 12.0 | 0.5 |
| ENSTGUG00000016228 | FAM116B | 0.025 | 0.002 | 24.0 | 2.0 | 0.5 |
| ENSTGUG00000006294 | PLCB1 | 0.025 | 0.002 | 24.0 | 12.0 | 0.5 |
| ENSTGUG00000001539 | SELL | 0.025 | 0.002 | 24.0 | 9.0 | 0.5 |
| ENSTGUG00000013053 | ATP6V1C2 | 0.025 | 0.002 | 24.0 | 22.0 | 0.5 |
| ENSTGUG00000007049 | DPEP3 | 0.025 | 0.002 | 24.0 | 1.0 | 0.5 |
| ENSTGUG00000010919 | MGA | 0.025 | 0.002 | 24.0 | 16.0 | 0.5 |
| ENSTGUG00000003703 | LINGO1 | 0.025 | 0.002 | 24.0 | 4.0 | 0.5 |
| ENSTGUG00000012851 | TAF1D | 0.025 | 0.002 | 24.0 | 16.0 | 0.4 |
| ENSTGUG00000007609 | MYO18B-2 | 0.025 | 0.002 | 24.0 | 12.0 | 0.4 |
| ENSTGUG00000016156 |  | 0.025 | 0.002 | 24.0 | 2.0 | 0.4 |
| ENSTGUG00000005483 | KIAA0895 | 0.025 | 0.002 | 25.3 | 16.7 | 0.4 |
| ENSTGUG00000007464 | DGKD | 0.025 | 0.002 | 24.0 | 2.0 | 0.4 |
| ENSTGUG00000011213 | DNAL1 | 0.025 | 0.002 | 24.0 | 14.0 | 0.4 |
| ENSTGUG00000002793 | HEPH | 0.025 | 0.002 | 24.0 | 9.0 | 0.4 |
| ENSTGUG00000003093 |  | 0.025 | 0.002 | 24.0 | 13.0 | 0.4 |
| ENSTGUG00000010235 | MGAT3 | 0.025 | 0.002 | 24.0 | 12.0 | 0.4 |
| ENSTGUG00000011808 | PRKD1 | 0.025 | 0.002 | 24.0 | 10.0 | 0.4 |
| ENSTGUG00000005951 | TLL1 | 0.025 | 0.002 | 24.0 | 2.0 | 0.4 |
| ENSTGUG00000007862 | ZSWIM5 | 0.025 | 0.002 | 24.0 | 0.0 | 0.4 |
| ENSTGUG00000010040 | EHF | 0.025 | 0.002 | 24.0 | 10.0 | 0.4 |
| ENSTGUG00000006921 | SCG3 | 0.025 | 0.002 | 24.0 | 17.0 | 0.4 |
| ENSTGUG00000006997 | TNKS1BP1 | 0.025 | 0.002 | 24.0 | 1.0 | 0.4 |
| ENSTGUG00000002634 | CHD5 | 0.025 | 0.002 | 24.0 | 4.0 | 0.4 |
| ENSTGUG00000017147 |  | 0.025 | 0.002 | 24.0 | 22.0 | 0.4 |
| ENSTGUG00000001979 | PRLR | 0.025 | 0.002 | 26.0 | 18.0 | 0.4 |
| ENSTGUG00000009106 | ZNF516 | 0.025 | 0.002 | 24.0 | 10.0 | 0.4 |
| ENSTGUG00000003154 | PLCL2 | 0.025 | 0.002 | 24.0 | 12.0 | 0.4 |
| ENSTGUG00000003303 |  | 0.025 | 0.002 | 24.0 | 10.0 | 0.4 |
| ENSTGUG00000012408 | PTPN21 | 0.025 | 0.002 | 24.0 | 20.0 | 0.4 |
| ENSTGUG00000011506 |  | 0.025 | 0.002 | 25.3 | 16.7 | 0.4 |
| ENSTGUG00000017182 |  | 0.025 | 0.002 | 24.0 | 12.0 | 0.4 |
| ENSTGUG00000002213 | PKD2 | 0.025 | 0.002 | 24.0 | 9.0 | 0.4 |
| ENSTGUG00000003307 | SCN8A | 0.025 | 0.002 | 24.0 | 6.0 | 0.4 |
| ENSTGUG00000015771 |  | 0.025 | 0.002 | 24.0 | 12.0 | 0.4 |
| ENSTGUG00000006844 | XK | 0.025 | 0.002 | 24.0 | 10.0 | 0.4 |
| ENSTGUG00000012122 | ARMC2 | 0.025 | 0.002 | 24.0 | 18.0 | 0.4 |
| ENSTGUG00000015686 |  | 0.025 | 0.002 | 24.0 | 12.0 | 0.4 |
| ENSTGUG00000004073 | B3GALT6 | 0.025 | 0.002 | 24.0 | 0.0 | 0.4 |
| ENSTGUG00000008387 | GNB1L | 0.025 | 0.002 | 24.0 | 20.0 | 0.4 |
| ENSTGUG00000010694 | NCF4 | 0.025 | 0.002 | 24.0 | 12.0 | 0.4 |
| ENSTGUG00000002345 | HCN1 | 0.025 | 0.002 | 24.0 | 21.0 | 0.4 |
| ENSTGUG00000013442 | URB1 | 0.025 | 0.002 | 24.0 | 0.0 | 0.4 |
| ENSTGUG00000016874 | ALOX12B | 0.025 | 0.002 | 24.0 | 10.0 | 0.3 |
| ENSTGUG00000012963 | SYTL2 | 0.025 | 0.002 | 24.0 | 14.0 | 0.3 |
| ENSTGUG00000001746 | ARID3C | 0.025 | 0.002 | 26.0 | 5.0 | 0.3 |
| ENSTGUG00000006825 | ATHL1 | 0.025 | 0.002 | 24.0 | 9.0 | 0.3 |
| ENSTGUG00000010199 | CABIN1 | 0.025 | 0.002 | 24.0 | 14.0 | 0.3 |
| ENSTGUG00000009479 | ATF7IP | 0.025 | 0.002 | 24.0 | 10.0 | 0.3 |
| ENSTGUG00000008636 | IGF1R | 0.025 | 0.002 | 24.0 | 20.0 | 0.3 |
| ENSTGUG00000011117 | FAM110B | 0.025 | 0.002 | 24.0 | 10.0 | 0.3 |
| ENSTGUG00000013159 |  | 0.025 | 0.002 | 24.0 | 13.0 | 0.3 |
| ENSTGUG00000000100 |  | 0.025 | 0.002 | 24.0 | 16.0 | 0.3 |
| ENSTGUG00000008559 | EVI2A | 0.025 | 0.002 | 24.0 | 13.0 | 0.3 |
| ENSTGUG00000004366 | SEMA5B | 0.025 | 0.002 | 24.0 | 0.0 | 0.3 |
| ENSTGUG00000011581 | RASL11A | 0.025 | 0.002 | 24.0 | 9.0 | 0.3 |
| ENSTGUG00000014661 |  | 0.025 | 0.002 | 24.0 | 12.0 | 0.3 |
| ENSTGUG00000010965 | GPR183 | 0.025 | 0.002 | 24.0 | 12.0 | 0.3 |
| ENSTGUG00000009151 | ABCC3 | 0.025 | 0.002 | 24.0 | 10.0 | 0.3 |
| ENSTGUG00000001837 | FOXP4 | 0.025 | 0.002 | 24.0 | 0.0 | 0.3 |
| ENSTGUG00000007084 | PPP2R3A | 0.025 | 0.002 | 24.0 | 9.0 | 0.3 |
| ENSTGUG00000014410 | FAM38A | 0.025 | 0.002 | 24.0 | 2.0 | 0.3 |
| ENSTGUG00000012077 | GPR6 | 0.025 | 0.002 | 24.0 | 0.0 | 0.3 |
| ENSTGUG00000008856 | MYLK3 | 0.025 | 0.002 | 24.0 | 22.0 | 0.3 |
| ENSTGUG00000007686 | KIT | 0.025 | 0.002 | 24.0 | 13.0 | 0.3 |
| ENSTGUG00000012221 | FLVCR2 | 0.025 | 0.002 | 24.0 | 10.0 | 0.3 |
| ENSTGUG00000005422 | RFT1 | 0.025 | 0.002 | 24.0 | 12.0 | 0.3 |
| ENSTGUG00000001966 | PLXNA4-1 | 0.025 | 0.002 | 24.0 | 0.0 | 0.3 |
| ENSTGUG00000001977 |  | 0.025 | 0.002 | 24.0 | 8.0 | 0.3 |
| ENSTGUG00000006226 | LRRC8C | 0.025 | 0.002 | 24.0 | 12.0 | 0.3 |
| ENSTGUG00000017286 | LOXL2 | 0.025 | 0.002 | 24.0 | 0.0 | 0.3 |
| ENSTGUG00000012821 | TRAF3 | 0.025 | 0.002 | 24.0 | 16.0 | 0.3 |
| ENSTGUG00000012523 | SNAP91 | 0.025 | 0.002 | 24.0 | 12.0 | 0.2 |
| ENSTGUG00000008965 |  | 0.025 | 0.002 | 24.0 | 12.0 | 0.2 |
| ENSTGUG00000004907 |  | 0.025 | 0.002 | 24.0 | 8.0 | 0.2 |
| ENSTGUG00000018303 |  | 0.025 | 0.002 | 24.0 | 13.0 | 0.2 |
| ENSTGUG00000012190 | ZFPM2 | 0.025 | 0.002 | 24.0 | 9.0 | 0.2 |
| ENSTGUG00000002407 | SEMA3D | 0.025 | 0.002 | 24.0 | 13.0 | 0.2 |
| ENSTGUG00000005640 | DBH | 0.025 | 0.002 | 26.0 | 2.0 | 0.2 |
| ENSTGUG00000002397 |  | 0.025 | 0.002 | 24.0 | 14.0 | 0.2 |
| ENSTGUG00000010310 | ATP10A | 0.025 | 0.002 | 24.0 | 12.0 | 0.2 |
| ENSTGUG00000008398 | FGD6 | 0.025 | 0.002 | 24.0 | 8.0 | 0.2 |
| ENSTGUG00000000031 | GFRA3 | 0.025 | 0.002 | 24.0 | 13.0 | 0.2 |
| ENSTGUG00000011735 | FRY | 0.025 | 0.002 | 24.0 | 10.0 | 0.2 |
| ENSTGUG00000007590 | C20orf74 | 0.025 | 0.002 | 24.0 | 10.0 | 0.2 |
| ENSTGUG00000000232 | PPARGC1B | 0.025 | 0.002 | 24.0 | 18.0 | 0.2 |
| ENSTGUG00000014151 |  | 0.025 | 0.002 | 24.0 | 12.0 | 0.2 |
| ENSTGUG00000001883 | SYNPO2 | 0.025 | 0.002 | 26.0 | 5.0 | 0.2 |
| ENSTGUG00000004265 | UNC5B | 0.025 | 0.002 | 24.0 | 4.0 | 0.2 |
| ENSTGUG00000003764 | RNF2 | 0.025 | 0.002 | 24.0 | 8.0 | 0.2 |
| ENSTGUG00000010875 | TNIK | 0.025 | 0.002 | 24.0 | 12.0 | 0.2 |
| ENSTGUG00000002459 | TNFRSF8-1 | 0.025 | 0.002 | 26.0 | 18.0 | 0.2 |
| ENSTGUG00000009614 |  | 0.025 | 0.002 | 26.0 | 5.0 | 0.2 |
| ENSTGUG00000001215 |  | 0.025 | 0.002 | 24.0 | 6.0 | 0.2 |
| ENSTGUG00000007933 | MGAT4C | 0.025 | 0.002 | 24.0 | 10.0 | 0.2 |
| ENSTGUG00000000321 | C19orf35 | 0.025 | 0.002 | 24.0 | 10.0 | 0.2 |
| ENSTGUG00000008166 | GPM6B | 0.025 | 0.002 | 24.0 | 10.0 | 0.2 |
| ENSTGUG00000006224 | C5orf37 | 0.025 | 0.002 | 24.0 | 16.0 | 0.2 |
| ENSTGUG00000000556 | NUP98 | 0.025 | 0.002 | 24.0 | 1.0 | 0.2 |
| ENSTGUG00000012019 | TPTE | 0.025 | 0.002 | 24.0 | 12.0 | 0.2 |
| ENSTGUG00000002453 | ESM1 | 0.025 | 0.002 | 24.0 | 14.0 | 0.2 |
| ENSTGUG00000007955 | MRVI1 | 0.025 | 0.002 | 24.0 | 10.0 | 0.2 |
| ENSTGUG00000010492 | PLCL1 | 0.025 | 0.002 | 24.0 | 10.0 | 0.2 |
| ENSTGUG00000014841 |  | 0.025 | 0.002 | 24.0 | 12.0 | 0.2 |
| ENSTGUG00000004621 | ZDHHC21 | 0.025 | 0.002 | 24.0 | 10.0 | 0.2 |
| ENSTGUG00000012556 | MYCBP2 | 0.025 | 0.002 | 24.0 | 13.0 | 0.1 |
| ENSTGUG00000000138 |  | 0.025 | 0.002 | 24.0 | 12.0 | 0.1 |
| ENSTGUG00000006089 | SPEG | 0.025 | 0.002 | 24.0 | 12.0 | 0.1 |
| ENSTGUG00000001199 | SPAG6 | 0.025 | 0.002 | 24.0 | 13.0 | 0.1 |
| ENSTGUG00000006408 | CAMSAP1 | 0.025 | 0.002 | 26.0 | 15.0 | 0.1 |
| ENSTGUG00000013359 | FAM46C | 0.025 | 0.002 | 24.0 | 12.0 | 0.1 |
| ENSTGUG00000004116 | AIFM2 | 0.025 | 0.002 | 24.0 | 20.0 | 0.1 |
| ENSTGUG00000004703 | CACNA2D2 | 0.025 | 0.002 | 24.0 | 9.0 | 0.1 |
| ENSTGUG00000005553 | C4orf18 | 0.025 | 0.002 | 24.0 | 9.0 | 0.1 |
| ENSTGUG00000002632 | ARR3 | 0.025 | 0.002 | 24.0 | 12.0 | 0.1 |
| ENSTGUG00000010126 | NDC80 | 0.025 | 0.002 | 24.0 | 20.0 | 0.1 |
| ENSTGUG00000002327 | FBXO44 | 0.025 | 0.002 | 24.0 | 0.0 | 0.1 |
| ENSTGUG00000003919 | HAX1 | 0.025 | 0.002 | 24.0 | 12.0 | 0.1 |
| ENSTGUG00000009658 | DACT2 | 0.025 | 0.002 | 24.0 | 6.0 | 0.1 |
| ENSTGUG00000000451 | HK3 | 0.025 | 0.002 | 24.0 | 8.0 | 0.1 |
| ENSTGUG00000010195 | COLEC12 | 0.025 | 0.002 | 24.0 | 12.0 | 0.1 |
| ENSTGUG00000016625 | ADAMTS14 | 0.025 | 0.002 | 26.0 | 5.0 | 0.1 |
| ENSTGUG00000006621 | NFATC1 | 0.025 | 0.002 | 24.0 | 8.0 | 0.1 |
| ENSTGUG00000012652 | EIF2C2 | 0.025 | 0.002 | 24.0 | 17.0 | 0.1 |
| ENSTGUG00000010152 | C11orf74 | 0.025 | 0.002 | 24.0 | 2.0 | 0.1 |
| ENSTGUG00000005096 | BSN | 0.025 | 0.002 | 24.0 | 8.0 | 0.1 |
| ENSTGUG00000012740 | COL19A1 | 0.025 | 0.002 | 25.3 | 16.7 | 0.1 |
| ENSTGUG00000002955 | MAST4-1 | 0.025 | 0.002 | 24.0 | 13.0 | 0.1 |
| ENSTGUG00000001847 |  | 0.025 | 0.002 | 24.0 | 21.0 | 0.1 |
| ENSTGUG00000012688 | KCNQ5 | 0.025 | 0.002 | 24.0 | 10.0 | 0.1 |
| ENSTGUG00000004671 | GPR124 | 0.025 | 0.002 | 24.0 | 4.0 | 0.1 |
| ENSTGUG00000007628 | C20orf26 | 0.025 | 0.002 | 24.0 | 14.0 | 0.1 |
| ENSTGUG00000011397 | CPXM2 | 0.025 | 0.002 | 24.0 | 4.0 | 0.1 |
| ENSTGUG00000009778 | ADAMTS7 | 0.025 | 0.002 | 24.0 | 12.0 | 0.1 |
| ENSTGUG00000001933 | ADAMTS12 | 0.025 | 0.002 | 24.0 | 8.0 | 0.1 |
| ENSTGUG00000010782 | TNFSF13B | 0.025 | 0.002 | 24.0 | 10.0 | 0.1 |
| ENSTGUG00000003870 | NLRC3 | 0.025 | 0.002 | 24.0 | 9.0 | 0.1 |
| ENSTGUG00000008031 | MCM2 | 0.025 | 0.002 | 24.0 | 10.0 | 0.1 |
| ENSTGUG00000004269 | ATP2C2 | 0.025 | 0.002 | 24.0 | 12.0 | 0.1 |
| ENSTGUG00000009049 | CACNA1G | 0.025 | 0.002 | 24.0 | 1.0 | 0.1 |
| ENSTGUG00000004593 | MMP17 | 0.025 | 0.002 | 24.0 | 14.0 | 0.1 |
| ENSTGUG00000017331 | HIVEP3-2 | 0.025 | 0.002 | 24.0 | 8.0 | 0.0 |
| ENSTGUG00000015609 | ARID5A | 0.056 | 0.004 | 28.0 | 0.0 | 18.8 |
| ENSTGUG00000014487 | COG8 | 0.056 | 0.004 | 28.0 | 0.0 | 3.9 |
| ENSTGUG00000013835 | DOCK7 | 0.056 | 0.004 | 28.0 | 0.0 | 1.3 |
| ENSTGUG00000006491 | SH2B3 | 0.056 | 0.004 | 28.0 | 0.0 | 0.8 |
| ENSTGUG00000014755 |  | 0.056 | 0.004 | 28.0 | 0.0 | 0.4 |
| ENSTGUG00000016257 |  | 0.056 | 0.004 | 28.0 | 0.0 | 0.3 |
| ENSTGUG00000016651 |  | 0.056 | 0.004 | 28.0 | 0.0 | 0.3 |
| ENSTGUG00000017310 | PPAPDC1B | 0.056 | 0.004 | 28.0 | 0.0 | 0.3 |
| ENSTGUG00000011261 | C6orf97 | 0.056 | 0.004 | 28.0 | 0.0 | 0.3 |
| ENSTGUG00000016574 |  | 0.056 | 0.004 | 28.0 | 0.0 | 0.2 |
| ENSTGUG00000001567 |  | 0.056 | 0.004 | 28.0 | 0.0 | 0.1 |
| ENSTGUG00000010663 | RBM20-1 | 0.056 | 0.004 | 28.0 | 0.0 | 0.1 |
| ENSTGUG00000010426 | MYBPC3 | 0.056 | 0.004 | 28.0 | 0.0 | 0.0 |
| ENSTGUG00000017540 | CDO1 | 0.102 | 0.009 | 28.0 | 1.0 | 2639.8 |
| ENSTGUG00000017838 |  | 0.102 | 0.009 | 24.0 | 2.0 | 351.3 |
| ENSTGUG00000016860 | C3 | 0.102 | 0.009 | 28.0 | 3.0 | 268.9 |
| ENSTGUG00000003882 | ENO1 | 0.102 | 0.009 | 28.0 | 3.0 | 210.7 |
| ENSTGUG00000004926 | RPS3A | 0.102 | 0.009 | 28.0 | 3.0 | 161.6 |
| ENSTGUG00000001073 | FCAMR | 0.102 | 0.009 | 28.0 | 3.0 | 140.3 |
| ENSTGUG00000007196 | RPL35 | 0.102 | 0.009 | 28.0 | 3.0 | 77.8 |
| ENSTGUG00000013142 | PYGL | 0.102 | 0.009 | 28.0 | 3.0 | 64.0 |
| ENSTGUG00000003059 | PLS3 | 0.102 | 0.009 | 28.0 | 1.0 | 43.9 |
| ENSTGUG00000005094 |  | 0.102 | 0.009 | 28.0 | 3.0 | 41.0 |
| ENSTGUG00000004883 | LITAF | 0.102 | 0.009 | 28.0 | 3.0 | 31.0 |
| ENSTGUG00000014970 | NPM1 | 0.102 | 0.009 | 28.0 | 17.0 | 29.5 |
| ENSTGUG00000011079 | PDCD10 | 0.102 | 0.009 | 28.0 | 17.0 | 28.8 |
| ENSTGUG00000003880 |  | 0.102 | 0.009 | 28.0 | 3.0 | 26.2 |
| ENSTGUG00000013481 | SERPINH1 | 0.102 | 0.009 | 28.0 | 3.0 | 23.7 |
| ENSTGUG00000007703 | NDUFV1 | 0.102 | 0.009 | 28.0 | 3.0 | 16.5 |
| ENSTGUG00000017739 |  | 0.102 | 0.009 | 24.0 | 0.0 | 14.5 |
| ENSTGUG00000003432 | NDUFA1 | 0.102 | 0.009 | 28.0 | 3.0 | 13.7 |
| ENSTGUG00000001463 | B4GALT1 | 0.102 | 0.009 | 28.0 | 1.0 | 13.4 |
| ENSTGUG00000011964 | HDAC2 | 0.102 | 0.009 | 28.0 | 17.0 | 12.7 |
| ENSTGUG00000012480 | NDUFB9 | 0.102 | 0.009 | 28.0 | 3.0 | 12.1 |
| ENSTGUG00000000518 | SLC35D2 | 0.102 | 0.009 | 28.0 | 15.0 | 10.5 |
| ENSTGUG00000015279 |  | 0.102 | 0.009 | 24.0 | 6.0 | 9.8 |
| ENSTGUG00000012618 | UBR7 | 0.102 | 0.009 | 28.0 | 15.0 | 9.1 |
| ENSTGUG00000004665 | CARHSP1 | 0.102 | 0.009 | 28.0 | 3.0 | 8.5 |
| ENSTGUG00000009057 |  | 0.102 | 0.009 | 28.0 | 3.0 | 8.3 |
| ENSTGUG00000008201 | C2orf37 | 0.102 | 0.009 | 28.0 | 17.0 | 7.7 |
| ENSTGUG00000006984 | STRBP | 0.102 | 0.009 | 28.0 | 15.0 | 7.6 |
| ENSTGUG00000012710 | ASRGL1 | 0.102 | 0.009 | 28.0 | 17.0 | 7.2 |
| ENSTGUG00000013169 | PARP11 | 0.102 | 0.009 | 28.0 | 17.0 | 6.2 |
| ENSTGUG00000009736 | NAPG | 0.102 | 0.009 | 28.0 | 17.0 | 6.2 |
| ENSTGUG00000005643 | RILP | 0.102 | 0.009 | 28.0 | 15.0 | 5.9 |
| ENSTGUG00000002971 | ERGIC3 | 0.102 | 0.009 | 28.0 | 17.0 | 5.7 |
| ENSTGUG00000010752 | KIAA1012 | 0.102 | 0.009 | 28.0 | 15.0 | 5.4 |
| ENSTGUG00000004986 | PET112L | 0.102 | 0.009 | 28.0 | 17.0 | 5.4 |
| ENSTGUG00000008852 | ANKRD10 | 0.102 | 0.009 | 28.0 | 1.0 | 5.1 |
| ENSTGUG00000007712 | CIDEC | 0.102 | 0.009 | 28.0 | 3.0 | 5.0 |
| ENSTGUG00000002118 | CDS1 | 0.102 | 0.009 | 28.0 | 15.0 | 4.8 |
| ENSTGUG00000006085 | CBR4 | 0.102 | 0.009 | 28.0 | 17.0 | 4.7 |
| ENSTGUG00000017408 | KIAA0907 | 0.102 | 0.009 | 28.0 | 1.0 | 4.3 |
| ENSTGUG00000011548 | TCF21 | 0.102 | 0.009 | 28.0 | 3.0 | 4.2 |
| ENSTGUG00000005061 | XPNPEP2 | 0.102 | 0.009 | 28.0 | 3.0 | 4.1 |
| ENSTGUG00000012615 | WNK1 | 0.102 | 0.009 | 28.0 | 3.0 | 3.8 |
| ENSTGUG00000017500 | KLHDC8A | 0.102 | 0.009 | 24.0 | 9.0 | 3.8 |
| ENSTGUG00000018198 |  | 0.102 | 0.009 | 24.0 | 10.0 | 3.8 |
| ENSTGUG00000003119 | ROGDI | 0.102 | 0.009 | 28.0 | 17.0 | 3.7 |
| ENSTGUG00000005868 | C3orf34 | 0.102 | 0.009 | 28.0 | 17.0 | 3.5 |
| ENSTGUG00000012079 | FIG4 | 0.102 | 0.009 | 28.0 | 17.0 | 3.4 |
| ENSTGUG00000011080 | C10orf84 | 0.102 | 0.009 | 28.0 | 15.0 | 3.4 |
| ENSTGUG00000007580 | METRNL | 0.102 | 0.009 | 28.0 | 15.0 | 2.9 |
| ENSTGUG00000006307 | FSD1L | 0.102 | 0.009 | 28.0 | 1.0 | 2.8 |
| ENSTGUG00000013512 | ILDR2 | 0.102 | 0.009 | 28.0 | 1.0 | 2.7 |
| ENSTGUG00000018160 |  | 0.102 | 0.009 | 28.0 | 17.0 | 2.6 |
| ENSTGUG00000010039 | SFXN3 | 0.102 | 0.009 | 28.0 | 1.0 | 2.4 |
| ENSTGUG00000002363 | ETAA1 | 0.102 | 0.009 | 28.0 | 15.0 | 2.4 |
| ENSTGUG00000014012 | G0S2 | 0.102 | 0.009 | 24.0 | 9.0 | 2.3 |
| ENSTGUG00000017933 |  | 0.102 | 0.009 | 24.0 | 12.0 | 2.3 |
| ENSTGUG00000016106 |  | 0.102 | 0.009 | 24.0 | 1.0 | 2.2 |
| ENSTGUG00000007527 | MAP7D2 | 0.102 | 0.009 | 28.0 | 17.0 | 2.1 |
| ENSTGUG00000013639 |  | 0.102 | 0.009 | 28.0 | 17.0 | 2.1 |
| ENSTGUG00000009836 | IRF7 | 0.102 | 0.009 | 24.0 | 1.0 | 2.0 |
| ENSTGUG00000001923 | COPG2 | 0.102 | 0.009 | 28.0 | 3.0 | 1.9 |
| ENSTGUG00000008588 | PLS1 | 0.102 | 0.009 | 28.0 | 15.0 | 1.9 |
| ENSTGUG00000015709 |  | 0.102 | 0.009 | 24.0 | 13.0 | 1.9 |
| ENSTGUG00000009003 | EEF2K | 0.102 | 0.009 | 28.0 | 17.0 | 1.9 |
| ENSTGUG00000017127 |  | 0.102 | 0.009 | 24.0 | 2.0 | 1.9 |
| ENSTGUG00000008494 | SPAST | 0.102 | 0.009 | 28.0 | 17.0 | 1.9 |
| ENSTGUG00000003980 | RAB43 | 0.102 | 0.009 | 28.0 | 3.0 | 1.8 |
| ENSTGUG00000017375 | MRPL24 | 0.102 | 0.009 | 28.0 | 3.0 | 1.6 |
| ENSTGUG00000001565 | AP4B1 | 0.102 | 0.009 | 28.0 | 1.0 | 1.5 |
| ENSTGUG00000003516 | ITCH | 0.102 | 0.009 | 28.0 | 17.0 | 1.3 |
| ENSTGUG00000014389 |  | 0.102 | 0.009 | 28.0 | 3.0 | 1.1 |
| ENSTGUG00000016015 |  | 0.102 | 0.009 | 28.0 | 15.0 | 1.0 |
| ENSTGUG00000005563 | TMEM144 | 0.102 | 0.009 | 28.0 | 17.0 | 1.0 |
| ENSTGUG00000010769 | SHPRH | 0.102 | 0.009 | 28.0 | 15.0 | 1.0 |
| ENSTGUG00000012068 | ABCC9 | 0.102 | 0.009 | 28.0 | 1.0 | 0.9 |
| ENSTGUG00000009345 | LEPREL1 | 0.102 | 0.009 | 28.0 | 3.0 | 0.9 |
| ENSTGUG00000011077 | CALCRL | 0.102 | 0.009 | 28.0 | 3.0 | 0.9 |
| ENSTGUG00000013910 |  | 0.102 | 0.009 | 24.0 | 9.0 | 0.8 |
| ENSTGUG00000014849 |  | 0.102 | 0.009 | 24.0 | 14.0 | 0.7 |
| ENSTGUG00000018477 |  | 0.102 | 0.009 | 24.0 | 9.0 | 0.6 |
| ENSTGUG00000014738 |  | 0.102 | 0.009 | 24.0 | 13.0 | 0.6 |
| ENSTGUG00000005160 | RBP2 | 0.102 | 0.009 | 24.0 | 4.0 | 0.6 |
| ENSTGUG00000008164 | CEP170 | 0.102 | 0.009 | 28.0 | 1.0 | 0.6 |
| ENSTGUG00000012095 |  | 0.102 | 0.009 | 24.0 | 21.0 | 0.6 |
| ENSTGUG00000007906 | VWC2 | 0.102 | 0.009 | 24.0 | 4.0 | 0.6 |
| ENSTGUG00000015182 |  | 0.102 | 0.009 | 24.0 | 12.0 | 0.6 |
| ENSTGUG00000014727 |  | 0.102 | 0.009 | 24.0 | 9.0 | 0.5 |
| ENSTGUG00000012630 | PRIMA1 | 0.102 | 0.009 | 24.0 | 0.0 | 0.5 |
| ENSTGUG00000005127 |  | 0.102 | 0.009 | 24.0 | 6.0 | 0.5 |
| ENSTGUG00000006164 | SNX21-2 | 0.102 | 0.009 | 28.0 | 3.0 | 0.5 |
| ENSTGUG00000017201 |  | 0.102 | 0.009 | 24.0 | 9.0 | 0.5 |
| ENSTGUG00000015509 |  | 0.102 | 0.009 | 28.0 | 3.0 | 0.5 |
| ENSTGUG00000002997 | SDR39U1 | 0.102 | 0.009 | 28.0 | 3.0 | 0.4 |
| ENSTGUG00000015284 |  | 0.102 | 0.009 | 24.0 | 4.0 | 0.4 |
| ENSTGUG00000007794 | RAI2 | 0.102 | 0.009 | 24.0 | 2.0 | 0.4 |
| ENSTGUG00000000391 | PLCD1 | 0.102 | 0.009 | 28.0 | 1.0 | 0.4 |
| ENSTGUG00000012919 | GPR132 | 0.102 | 0.009 | 24.0 | 4.0 | 0.4 |
| ENSTGUG00000002782 | EDA2R | 0.102 | 0.009 | 24.0 | 9.0 | 0.4 |
| ENSTGUG00000007287 | C15orf52 | 0.102 | 0.009 | 24.0 | 11.0 | 0.4 |
| ENSTGUG00000000568 | TMEM45B | 0.102 | 0.009 | 24.0 | 1.0 | 0.3 |
| ENSTGUG00000004513 | RASA4B | 0.102 | 0.009 | 28.0 | 3.0 | 0.3 |
| ENSTGUG00000015473 |  | 0.102 | 0.009 | 24.0 | 20.0 | 0.3 |
| ENSTGUG00000013691 | ZFP1-1 | 0.102 | 0.009 | 24.0 | 0.0 | 0.3 |
| ENSTGUG00000013860 |  | 0.102 | 0.009 | 24.0 | 4.0 | 0.3 |
| ENSTGUG00000015864 |  | 0.102 | 0.009 | 28.0 | 15.0 | 0.3 |
| ENSTGUG00000008433 | CUEDC1 | 0.102 | 0.009 | 24.0 | 1.0 | 0.3 |
| ENSTGUG00000004440 |  | 0.102 | 0.009 | 24.0 | 13.0 | 0.3 |
| ENSTGUG00000002745 | PTPN13 | 0.102 | 0.009 | 28.0 | 1.0 | 0.3 |
| ENSTGUG00000015546 | CACNB3 | 0.102 | 0.009 | 24.0 | 5.0 | 0.3 |
| ENSTGUG00000015751 |  | 0.102 | 0.009 | 24.0 | 11.0 | 0.3 |
| ENSTGUG00000010881 | IL20RA | 0.102 | 0.009 | 24.0 | 13.0 | 0.3 |
| ENSTGUG00000013459 | GRIK1 | 0.102 | 0.009 | 28.0 | 3.0 | 0.2 |
| ENSTGUG00000016982 |  | 0.102 | 0.009 | 24.0 | 9.0 | 0.2 |
| ENSTGUG00000007863 | RASL11B | 0.102 | 0.009 | 24.0 | 6.0 | 0.2 |
| ENSTGUG00000018305 |  | 0.102 | 0.009 | 24.0 | 6.0 | 0.2 |
| ENSTGUG00000001720 | DNAI1 | 0.102 | 0.009 | 24.0 | 13.0 | 0.2 |
| ENSTGUG00000007707 | PKD2L1 | 0.102 | 0.009 | 28.0 | 15.0 | 0.2 |
| ENSTGUG00000012092 | C6orf199 | 0.102 | 0.009 | 24.0 | 20.0 | 0.2 |
| ENSTGUG00000000958 | NGFB | 0.102 | 0.009 | 24.0 | 6.0 | 0.2 |
| ENSTGUG00000015075 |  | 0.102 | 0.009 | 24.0 | 0.0 | 0.2 |
| ENSTGUG00000009582 | CRTAC1 | 0.102 | 0.009 | 24.0 | 6.0 | 0.2 |
| ENSTGUG00000008348 | BMP7 | 0.102 | 0.009 | 28.0 | 3.0 | 0.2 |
| ENSTGUG00000004013 | LEF1 | 0.102 | 0.009 | 24.0 | 13.0 | 0.2 |
| ENSTGUG00000011322 | MAP2 | 0.102 | 0.009 | 24.0 | 2.0 | 0.2 |
| ENSTGUG00000013075 | DLGAP5 | 0.102 | 0.009 | 24.0 | 17.0 | 0.2 |
| ENSTGUG00000001593 | ADAMTS3 | 0.102 | 0.009 | 28.0 | 3.0 | 0.2 |
| ENSTGUG00000000195 | C1QTNF2 | 0.102 | 0.009 | 24.0 | 0.0 | 0.1 |
| ENSTGUG00000007459 | FBLN7-2 | 0.102 | 0.009 | 24.0 | 10.0 | 0.1 |
| ENSTGUG00000003133 | SLC26A4 | 0.102 | 0.009 | 24.0 | 11.0 | 0.1 |
| ENSTGUG00000014968 |  | 0.102 | 0.009 | 24.0 | 2.0 | 0.1 |
| ENSTGUG00000006388 | WDR17 | 0.102 | 0.009 | 24.0 | 7.0 | 0.1 |
| ENSTGUG00000003108 | VWA1 | 0.102 | 0.009 | 24.0 | 2.0 | 0.1 |
| ENSTGUG00000006074 | ANKRD31-1 | 0.102 | 0.009 | 24.0 | 22.0 | 0.1 |
| ENSTGUG00000006175 |  | 0.102 | 0.009 | 24.0 | 12.0 | 0.1 |
| ENSTGUG00000002354 | RNF150 | 0.102 | 0.009 | 24.0 | 2.0 | 0.1 |
| ENSTGUG00000010102 | CNTN4 | 0.102 | 0.009 | 24.0 | 11.0 | 0.1 |
| ENSTGUG00000010366 | CHRD-1 | 0.102 | 0.009 | 24.0 | 2.0 | 0.1 |
| ENSTGUG00000008928 |  | 0.102 | 0.009 | 24.0 | 21.0 | 0.1 |
| ENSTGUG00000001550 | EREG | 0.102 | 0.009 | 24.0 | 0.0 | 0.1 |
| ENSTGUG00000005816 | GPRIN2 | 0.102 | 0.009 | 24.0 | 4.0 | 0.1 |
| ENSTGUG00000016914 |  | 0.102 | 0.009 | 24.0 | 2.0 | 0.1 |
| ENSTGUG00000000365 | ADAMTS19 | 0.102 | 0.009 | 24.0 | 1.0 | 0.1 |
| ENSTGUG00000010763 | ARSD-2 | 0.102 | 0.009 | 24.0 | 4.0 | 0.1 |
| ENSTGUG00000017135 | JAK3 | 0.102 | 0.009 | 24.0 | 12.0 | 0.1 |
| ENSTGUG00000007262 | CSRNP3 | 0.102 | 0.009 | 24.0 | 21.0 | 0.1 |
| ENSTGUG00000000013 | KATNAL2 | 0.102 | 0.009 | 24.0 | 12.0 | 0.1 |
| ENSTGUG00000011058 | SLC18A2 | 0.102 | 0.009 | 24.0 | 6.0 | 0.1 |
| ENSTGUG00000008016 | PIGW | 0.102 | 0.009 | 24.0 | 0.0 | 0.1 |
| ENSTGUG00000013607 | MYH15 | 0.102 | 0.009 | 24.0 | 13.0 | 0.1 |
| ENSTGUG00000016383 | SIGLEC1 | 0.102 | 0.009 | 24.0 | 6.0 | 0.1 |
| ENSTGUG00000009749 | PATZ1 | 0.102 | 0.009 | 24.0 | 6.0 | 0.1 |
| ENSTGUG00000012912 | MYOM2 | 0.102 | 0.009 | 24.0 | 15.0 | 0.1 |
| ENSTGUG00000008311 | DNER | 0.102 | 0.009 | 24.0 | 6.0 | 0.1 |
| ENSTGUG00000012786 | KHDRBS2 | 0.102 | 0.009 | 24.0 | 0.0 | 0.0 |
| ENSTGUG00000007744 | PTCH2 | 0.102 | 0.009 | 24.0 | 6.0 | 0.0 |
| ENSTGUG00000004482 | BNC2 | 0.102 | 0.009 | 24.0 | 2.0 | 0.0 |
| ENSTGUG00000002254 | MCM10 | 0.102 | 0.009 | 24.0 | 10.0 | 0.0 |
| ENSTGUG00000009008 | ANO4-2 | 0.102 | 0.009 | 24.0 | 6.0 | 0.0 |
| ENSTGUG00000013372 | STXBP5L | 0.102 | 0.009 | 24.0 | 12.0 | 0.0 |
| ENSTGUG00000012532 | ADCY8 | 0.102 | 0.009 | 24.0 | 6.0 | 0.0 |
| ENSTGUG00000011783 | PKDREJ-1 | 0.102 | 0.009 | 24.0 | 0.0 | 0.0 |
| ENSTGUG00000007680 |  | 0.102 | 0.009 | 24.0 | 13.0 | 0.0 |
| ENSTGUG00000000562 |  | 0.179 | 0.032 | 28.0 | 2.0 | 15753.2 |
| ENSTGUG00000008768 | RBP4 | 0.179 | 0.032 | 25.3 | 8.0 | 5431.1 |
| ENSTGUG00000007418 | C20orf149 | 0.179 | 0.032 | 24.0 | 5.0 | 4473.4 |
| ENSTGUG00000005279 | FGG | 0.179 | 0.032 | 24.0 | 1.0 | 1924.3 |
| ENSTGUG00000018753 |  | 0.179 | 0.032 | 24.0 | 5.0 | 1609.9 |
| ENSTGUG00000016325 | PTMS | 0.179 | 0.032 | 24.0 | 9.0 | 1448.0 |
| ENSTGUG00000006576 | LTF | 0.179 | 0.032 | 24.0 | 8.0 | 1425.3 |
| ENSTGUG00000018764 |  | 0.179 | 0.032 | 24.0 | 4.0 | 1080.8 |
| ENSTGUG00000002279 | HMGCS1 | 0.179 | 0.032 | 24.0 | 22.0 | 1058.4 |
| ENSTGUG00000013338 | GAPDH | 0.179 | 0.032 | 25.3 | 8.0 | 1057.2 |
| ENSTGUG00000010708 | TTR-1 | 0.179 | 0.032 | 24.0 | 8.0 | 1044.7 |
| ENSTGUG00000005933 | VTN | 0.179 | 0.032 | 24.0 | 4.0 | 1010.4 |
| ENSTGUG00000012540 | ME1 | 0.179 | 0.032 | 24.0 | 12.0 | 857.4 |
| ENSTGUG00000003884 | SPP2 | 0.179 | 0.032 | 24.0 | 12.0 | 854.7 |
| ENSTGUG00000012088 | LDHB | 0.179 | 0.032 | 25.3 | 9.3 | 678.5 |
| ENSTGUG00000005086 | PSPH-1 | 0.179 | 0.032 | 24.0 | 4.0 | 614.9 |
| ENSTGUG00000013017 | THRSP | 0.179 | 0.032 | 25.3 | 8.0 | 586.9 |
| ENSTGUG00000003783 | BHMT2 | 0.179 | 0.032 | 24.0 | 4.0 | 559.1 |
| ENSTGUG00000018724 |  | 0.179 | 0.032 | 25.3 | 4.0 | 407.6 |
| ENSTGUG00000005501 | CLDN15 | 0.179 | 0.032 | 24.0 | 12.0 | 305.2 |
| ENSTGUG00000003254 | B5G3G3_TAEGU | 0.179 | 0.032 | 26.0 | 5.0 | 304.9 |
| ENSTGUG00000013463 | SOD1 | 0.179 | 0.032 | 26.0 | 5.0 | 263.9 |
| ENSTGUG00000006111 | HPD | 0.179 | 0.032 | 24.0 | 1.0 | 256.2 |
| ENSTGUG00000010464 | NIPSNAP1 | 0.179 | 0.032 | 26.0 | 5.0 | 249.2 |
| ENSTGUG00000012576 | NDRG1 | 0.179 | 0.032 | 26.0 | 15.0 | 240.5 |
| ENSTGUG00000016242 | THRSPB | 0.179 | 0.032 | 25.3 | 8.0 | 239.2 |
| ENSTGUG00000006659 | GCHFR | 0.179 | 0.032 | 28.0 | 2.0 | 216.1 |
| ENSTGUG00000011097 | RPS20 | 0.179 | 0.032 | 26.0 | 5.0 | 213.4 |
| ENSTGUG00000017523 |  | 0.179 | 0.032 | 24.0 | 4.0 | 208.5 |
| ENSTGUG00000015517 |  | 0.179 | 0.032 | 24.0 | 1.0 | 200.0 |
| ENSTGUG00000012004 | RPL30 | 0.179 | 0.032 | 24.0 | 16.0 | 184.4 |
| ENSTGUG00000004885 | GLDC | 0.179 | 0.032 | 24.0 | 2.0 | 181.4 |
| ENSTGUG00000007413 | PPAT | 0.179 | 0.032 | 26.0 | 2.0 | 167.4 |
| ENSTGUG00000018735 |  | 0.179 | 0.032 | 24.0 | 17.0 | 165.9 |
| ENSTGUG00000002186 | RPL37 | 0.179 | 0.032 | 24.0 | 10.0 | 165.8 |
| ENSTGUG00000011404 | ACAT2 | 0.179 | 0.032 | 24.0 | 1.0 | 162.4 |
| ENSTGUG00000005737 | SERPINF2 | 0.179 | 0.032 | 24.0 | 0.0 | 149.8 |
| ENSTGUG00000009458 | RPLP0 | 0.179 | 0.032 | 26.0 | 5.0 | 149.5 |
| ENSTGUG00000008414 | SCCPDH | 0.179 | 0.032 | 26.0 | 15.0 | 144.9 |
| ENSTGUG00000015799 | TIMP2 | 0.179 | 0.032 | 24.0 | 2.0 | 144.6 |
| ENSTGUG00000004450 | XDH | 0.179 | 0.032 | 24.0 | 21.0 | 141.9 |
| ENSTGUG00000006052 | PGK1 | 0.179 | 0.032 | 24.0 | 10.0 | 138.8 |
| ENSTGUG00000015321 | RPS5 | 0.179 | 0.032 | 24.0 | 2.0 | 138.2 |
| ENSTGUG00000012287 | AKR1D1 | 0.179 | 0.032 | 24.0 | 0.0 | 136.0 |
| ENSTGUG00000003591 | C12orf62 | 0.179 | 0.032 | 26.0 | 5.0 | 135.0 |
| ENSTGUG00000004334 | RPS6 | 0.179 | 0.032 | 24.0 | 4.0 | 132.5 |
| ENSTGUG00000004584 | RPSA | 0.179 | 0.032 | 26.0 | 5.0 | 129.3 |
| ENSTGUG00000013578 |  | 0.179 | 0.032 | 26.0 | 5.0 | 121.8 |
| ENSTGUG00000006540 | MT4 | 0.179 | 0.032 | 24.0 | 0.0 | 117.6 |
| ENSTGUG00000004224 | CFHR5 | 0.179 | 0.032 | 25.3 | 4.0 | 113.2 |
| ENSTGUG00000011974 |  | 0.179 | 0.032 | 24.0 | 9.0 | 111.4 |
| ENSTGUG00000004571 | MDH2 | 0.179 | 0.032 | 24.0 | 4.0 | 110.9 |
| ENSTGUG00000008663 | BRI3 | 0.179 | 0.032 | 24.0 | 10.0 | 108.2 |
| ENSTGUG00000013618 | RPL24 | 0.179 | 0.032 | 24.0 | 2.0 | 107.7 |
| ENSTGUG00000007086 | RPS27A | 0.179 | 0.032 | 24.0 | 1.0 | 107.6 |
| ENSTGUG00000003102 | UGT1A9 | 0.179 | 0.032 | 24.0 | 1.0 | 99.1 |
| ENSTGUG00000004109 | COX4I1 | 0.179 | 0.032 | 24.0 | 4.0 | 99.1 |
| ENSTGUG00000015659 | FTL | 0.179 | 0.032 | 26.0 | 5.0 | 96.4 |
| ENSTGUG00000002866 | CYCS | 0.179 | 0.032 | 24.0 | 21.0 | 95.6 |
| ENSTGUG00000003983 | CYP2U1 | 0.179 | 0.032 | 26.0 | 15.0 | 92.3 |
| ENSTGUG00000006577 | VDAC2 | 0.179 | 0.032 | 24.0 | 20.0 | 91.6 |
| ENSTGUG00000008253 | NUDT4 | 0.179 | 0.032 | 26.0 | 15.0 | 91.4 |
| ENSTGUG00000016993 | C1orf151 | 0.179 | 0.032 | 24.0 | 22.0 | 91.1 |
| ENSTGUG00000005988 | RPL5 | 0.179 | 0.032 | 24.0 | 2.0 | 90.6 |
| ENSTGUG00000004539 | PPA1 | 0.179 | 0.032 | 25.3 | 4.0 | 89.0 |
| ENSTGUG00000012105 | CD164 | 0.179 | 0.032 | 26.0 | 2.0 | 83.2 |
| ENSTGUG00000013382 | COX17 | 0.179 | 0.032 | 28.0 | 2.0 | 80.0 |
| ENSTGUG00000002446 | EDF1 | 0.179 | 0.032 | 24.0 | 21.0 | 78.1 |
| ENSTGUG00000008947 | UQCRFS1 | 0.179 | 0.032 | 24.0 | 4.0 | 77.9 |
| ENSTGUG00000005641 | CYP27A1 | 0.179 | 0.032 | 26.0 | 5.0 | 77.7 |
| ENSTGUG00000000192 | LEAP2 | 0.179 | 0.032 | 24.0 | 8.0 | 76.2 |
| ENSTGUG00000003631 |  | 0.179 | 0.032 | 24.0 | 4.0 | 73.0 |
| ENSTGUG00000005659 | ABCD3 | 0.179 | 0.032 | 24.0 | 20.0 | 72.8 |
| ENSTGUG00000007790 | CYP3A5 | 0.179 | 0.032 | 25.3 | 4.0 | 72.2 |
| ENSTGUG00000017554 | RPS23 | 0.179 | 0.032 | 26.0 | 5.0 | 71.4 |
| ENSTGUG00000011395 | MRP63 | 0.179 | 0.032 | 24.0 | 12.0 | 71.2 |
| ENSTGUG00000004709 | ERLIN1 | 0.179 | 0.032 | 24.0 | 12.0 | 71.0 |
| ENSTGUG00000002528 | PPAP2A | 0.179 | 0.032 | 28.0 | 2.0 | 69.6 |
| ENSTGUG00000017584 |  | 0.179 | 0.032 | 24.0 | 13.0 | 66.9 |
| ENSTGUG00000007133 | B5G207_TAEGU | 0.179 | 0.032 | 26.0 | 5.0 | 66.6 |
| ENSTGUG00000010248 | RPL3 | 0.179 | 0.032 | 28.0 | 2.0 | 65.4 |
| ENSTGUG00000008946 | ACSF2 | 0.179 | 0.032 | 24.0 | 1.0 | 62.2 |
| ENSTGUG00000006444 | RPL18A | 0.179 | 0.032 | 24.0 | 4.0 | 61.9 |
| ENSTGUG00000001930 | ANXA5 | 0.179 | 0.032 | 24.0 | 0.0 | 61.9 |
| ENSTGUG00000003340 | ADH5 | 0.179 | 0.032 | 24.0 | 1.0 | 60.9 |
| ENSTGUG00000002178 | BPHL | 0.179 | 0.032 | 24.0 | 14.0 | 60.7 |
| ENSTGUG00000003472 | P4HB | 0.179 | 0.032 | 24.0 | 6.0 | 57.9 |
| ENSTGUG00000000431 | PRELID1 | 0.179 | 0.032 | 24.0 | 4.0 | 57.5 |
| ENSTGUG00000012983 | RPS7 | 0.179 | 0.032 | 24.0 | 16.0 | 56.5 |
| ENSTGUG00000015631 | NACA | 0.179 | 0.032 | 24.0 | 2.0 | 55.5 |
| ENSTGUG00000012275 |  | 0.179 | 0.032 | 24.0 | 10.0 | 55.4 |
| ENSTGUG00000009428 | CTSD | 0.179 | 0.032 | 24.0 | 6.0 | 55.4 |
| ENSTGUG00000016721 | ALDH4A1 | 0.179 | 0.032 | 24.0 | 4.0 | 54.9 |
| ENSTGUG00000011654 | POMP | 0.179 | 0.032 | 24.0 | 17.0 | 54.8 |
| ENSTGUG00000014295 | ANPEP | 0.179 | 0.032 | 28.0 | 2.0 | 54.4 |
| ENSTGUG00000013573 | STARD10 | 0.179 | 0.032 | 24.0 | 4.0 | 54.1 |
| ENSTGUG00000000542 | HIGD2A | 0.179 | 0.032 | 24.0 | 4.0 | 53.4 |
| ENSTGUG00000011643 | NDUFB2 | 0.179 | 0.032 | 24.0 | 21.0 | 52.3 |
| ENSTGUG00000001004 | EEF2 | 0.179 | 0.032 | 24.0 | 1.0 | 52.2 |
| ENSTGUG00000002508 | MOCS1 | 0.179 | 0.032 | 24.0 | 22.0 | 51.6 |
| ENSTGUG00000013836 | B5G1C7_TAEGU | 0.179 | 0.032 | 24.0 | 4.0 | 47.3 |
| ENSTGUG00000000076 | ATP5L | 0.179 | 0.032 | 26.0 | 5.0 | 47.1 |
| ENSTGUG00000009224 |  | 0.179 | 0.032 | 25.3 | 4.0 | 44.7 |
| ENSTGUG00000005194 | CHCHD2 | 0.179 | 0.032 | 24.0 | 6.0 | 44.4 |
| ENSTGUG00000016990 | RGS5 | 0.179 | 0.032 | 26.0 | 5.0 | 44.4 |
| ENSTGUG00000008178 | DCN | 0.179 | 0.032 | 24.0 | 9.0 | 44.3 |
| ENSTGUG00000003429 | ABCD2 | 0.179 | 0.032 | 24.0 | 13.0 | 42.6 |
| ENSTGUG00000014477 | HADHA | 0.179 | 0.032 | 24.0 | 4.0 | 42.4 |
| ENSTGUG00000001964 | CHCHD3 | 0.179 | 0.032 | 24.0 | 4.0 | 41.7 |
| ENSTGUG00000010576 | PRODH | 0.179 | 0.032 | 24.0 | 1.0 | 41.6 |
| ENSTGUG00000001043 | FKBP8 | 0.179 | 0.032 | 24.0 | 4.0 | 40.4 |
| ENSTGUG00000004261 | COTL1 | 0.179 | 0.032 | 24.0 | 13.0 | 40.0 |
| ENSTGUG00000008447 | PSMA1 | 0.179 | 0.032 | 26.0 | 18.0 | 38.9 |
| ENSTGUG00000001937 | GDI2 | 0.179 | 0.032 | 24.0 | 4.0 | 38.7 |
| ENSTGUG00000000904 | ID3 | 0.179 | 0.032 | 24.0 | 2.0 | 38.0 |
| ENSTGUG00000003502 | H2AFZ | 0.179 | 0.032 | 24.0 | 13.0 | 37.2 |
| ENSTGUG00000016331 |  | 0.179 | 0.032 | 28.0 | 2.0 | 36.2 |
| ENSTGUG00000017176 | CRIP1 | 0.179 | 0.032 | 25.3 | 8.0 | 35.9 |
| ENSTGUG00000001451 | CYP51A1 | 0.179 | 0.032 | 24.0 | 4.0 | 35.7 |
| ENSTGUG00000012882 | GSTA2 | 0.179 | 0.032 | 24.0 | 8.0 | 35.1 |
| ENSTGUG00000012391 | MGST1 | 0.179 | 0.032 | 24.0 | 12.0 | 35.1 |
| ENSTGUG00000012181 | MED21 | 0.179 | 0.032 | 24.0 | 4.0 | 34.1 |
| ENSTGUG00000014424 | SLC25A11 | 0.179 | 0.032 | 24.0 | 4.0 | 33.6 |
| ENSTGUG00000009675 |  | 0.179 | 0.032 | 24.0 | 0.0 | 31.8 |
| ENSTGUG00000005943 | PDXK | 0.179 | 0.032 | 24.0 | 14.0 | 31.4 |
| ENSTGUG00000007973 | OCIAD1 | 0.179 | 0.032 | 24.0 | 14.0 | 31.1 |
| ENSTGUG00000008739 | SLC25A3 | 0.179 | 0.032 | 24.0 | 20.0 | 31.1 |
| ENSTGUG00000005087 | CETP | 0.179 | 0.032 | 24.0 | 20.0 | 30.9 |
| ENSTGUG00000011392 | ACADL | 0.179 | 0.032 | 24.0 | 4.0 | 30.5 |
| ENSTGUG00000003273 | CACYBP | 0.179 | 0.032 | 24.0 | 18.0 | 30.5 |
| ENSTGUG00000012317 | SLC25A30 | 0.179 | 0.032 | 24.0 | 1.0 | 30.2 |
| ENSTGUG00000018480 |  | 0.179 | 0.032 | 24.0 | 13.0 | 29.6 |
| ENSTGUG00000008218 | SEDLP | 0.179 | 0.032 | 28.0 | 16.0 | 29.4 |
| ENSTGUG00000012668 | CLMN-1 | 0.179 | 0.032 | 24.0 | 13.0 | 28.2 |
| ENSTGUG00000000991 | CREB3L3 | 0.179 | 0.032 | 24.0 | 12.0 | 28.2 |
| ENSTGUG00000009630 |  | 0.179 | 0.032 | 24.0 | 13.0 | 28.0 |
| ENSTGUG00000008034 | CDH1 | 0.179 | 0.032 | 24.0 | 2.0 | 27.9 |
| ENSTGUG00000009863 | ANGPTL3-1 | 0.179 | 0.032 | 24.0 | 13.0 | 27.9 |
| ENSTGUG00000012313 |  | 0.179 | 0.032 | 24.0 | 9.0 | 27.7 |
| ENSTGUG00000012465 | CALM1 | 0.179 | 0.032 | 24.0 | 13.0 | 27.1 |
| ENSTGUG00000004672 | RANP1 | 0.179 | 0.032 | 25.3 | 20.7 | 26.7 |
| ENSTGUG00000007042 | TXNDC14 | 0.179 | 0.032 | 24.0 | 1.0 | 26.3 |
| ENSTGUG00000015842 |  | 0.179 | 0.032 | 24.0 | 22.0 | 26.2 |
| ENSTGUG00000004764 | MMP15 | 0.179 | 0.032 | 25.3 | 8.0 | 25.9 |
| ENSTGUG00000009806 | PSMB1 | 0.179 | 0.032 | 24.0 | 20.0 | 25.9 |
| ENSTGUG00000004207 | DCI | 0.179 | 0.032 | 24.0 | 1.0 | 25.5 |
| ENSTGUG00000002582 | ITM2A | 0.179 | 0.032 | 24.0 | 2.0 | 25.4 |
| ENSTGUG00000015913 | TUBA1C | 0.179 | 0.032 | 26.0 | 5.0 | 25.3 |
| ENSTGUG00000010367 | TOMM22 | 0.179 | 0.032 | 24.0 | 21.0 | 25.2 |
| ENSTGUG00000009158 | PEBP1 | 0.179 | 0.032 | 24.0 | 4.0 | 24.7 |
| ENSTGUG00000016030 | PARVA | 0.179 | 0.032 | 24.0 | 8.0 | 24.0 |
| ENSTGUG00000012870 | FBXO9 | 0.179 | 0.032 | 26.0 | 18.0 | 23.9 |
| ENSTGUG00000002876 | CBX3 | 0.179 | 0.032 | 26.0 | 18.0 | 23.8 |
| ENSTGUG00000003898 | B5G047_TAEGU | 0.179 | 0.032 | 24.0 | 4.0 | 23.5 |
| ENSTGUG00000011659 | ACTR3 | 0.179 | 0.032 | 24.0 | 13.0 | 23.5 |
| ENSTGUG00000018276 |  | 0.179 | 0.032 | 24.0 | 4.0 | 23.2 |
| ENSTGUG00000005385 | ANO1 | 0.179 | 0.032 | 24.0 | 0.0 | 22.7 |
| ENSTGUG00000016508 | ADRM1 | 0.179 | 0.032 | 25.3 | 20.7 | 22.3 |
| ENSTGUG00000010360 |  | 0.179 | 0.032 | 24.0 | 12.0 | 22.1 |
| ENSTGUG00000006851 | PSMD13 | 0.179 | 0.032 | 24.0 | 4.0 | 21.9 |
| ENSTGUG00000010400 | PSMC3 | 0.179 | 0.032 | 24.0 | 21.0 | 21.4 |
| ENSTGUG00000003070 | MLXIPL | 0.179 | 0.032 | 25.3 | 8.0 | 21.2 |
| ENSTGUG00000003757 |  | 0.179 | 0.032 | 26.0 | 5.0 | 21.0 |
| ENSTGUG00000000930 | TCEA3 | 0.179 | 0.032 | 26.0 | 5.0 | 20.7 |
| ENSTGUG00000015708 |  | 0.179 | 0.032 | 24.0 | 1.0 | 20.7 |
| ENSTGUG00000002299 | PAIP1 | 0.179 | 0.032 | 26.0 | 18.0 | 19.8 |
| ENSTGUG00000006372 | RAD23B | 0.179 | 0.032 | 24.0 | 1.0 | 19.8 |
| ENSTGUG00000001364 | PLAA | 0.179 | 0.032 | 26.0 | 18.0 | 19.8 |
| ENSTGUG00000010463 | RABGGTB | 0.179 | 0.032 | 24.0 | 16.0 | 19.5 |
| ENSTGUG00000005273 | GUSB | 0.179 | 0.032 | 24.0 | 6.0 | 19.3 |
| ENSTGUG00000001696 | ZMAT2 | 0.179 | 0.032 | 26.0 | 18.0 | 19.2 |
| ENSTGUG00000012998 | ID2 | 0.179 | 0.032 | 24.0 | 4.0 | 18.4 |
| ENSTGUG00000010046 | FAM10A4 | 0.179 | 0.032 | 24.0 | 2.0 | 18.4 |
| ENSTGUG00000018526 |  | 0.179 | 0.032 | 24.0 | 4.0 | 18.4 |
| ENSTGUG00000007525 | ATP6V0B | 0.179 | 0.032 | 24.0 | 4.0 | 18.2 |
| ENSTGUG00000008508 | PPP1R3C | 0.179 | 0.032 | 24.0 | 13.0 | 18.2 |
| ENSTGUG00000005425 | EDNRB2 | 0.179 | 0.032 | 26.0 | 5.0 | 18.1 |
| ENSTGUG00000001002 | RPS6KA1 | 0.179 | 0.032 | 24.0 | 12.0 | 18.1 |
| ENSTGUG00000003641 | STUB1 | 0.179 | 0.032 | 24.0 | 1.0 | 18.0 |
| ENSTGUG00000002138 |  | 0.179 | 0.032 | 26.0 | 18.0 | 18.0 |
| ENSTGUG00000005184 | NICN1 | 0.179 | 0.032 | 24.0 | 4.0 | 17.9 |
| ENSTGUG00000009598 | C8A | 0.179 | 0.032 | 24.0 | 21.0 | 17.9 |
| ENSTGUG00000003886 | VPS26A | 0.179 | 0.032 | 26.0 | 15.0 | 17.8 |
| ENSTGUG00000008876 | RAC1 | 0.179 | 0.032 | 25.3 | 4.0 | 17.6 |
| ENSTGUG00000013570 | ARRB1 | 0.179 | 0.032 | 24.0 | 5.0 | 17.5 |
| ENSTGUG00000005254 | DHCR7 | 0.179 | 0.032 | 24.0 | 0.0 | 17.5 |
| ENSTGUG00000015436 | CES3-2 | 0.179 | 0.032 | 24.0 | 6.0 | 17.3 |
| ENSTGUG00000000510 | HSD11B1L | 0.179 | 0.032 | 24.0 | 13.0 | 17.1 |
| ENSTGUG00000013236 | B5FXG6_TAEGU | 0.179 | 0.032 | 24.0 | 21.0 | 17.0 |
| ENSTGUG00000006218 | XRCC6BP1 | 0.179 | 0.032 | 24.0 | 14.0 | 16.4 |
| ENSTGUG00000004067 | AGXT2L1 | 0.179 | 0.032 | 24.0 | 9.0 | 16.4 |
| ENSTGUG00000010223 | USP14 | 0.179 | 0.032 | 26.0 | 18.0 | 16.0 |
| ENSTGUG00000017005 | SLC27A2 | 0.179 | 0.032 | 28.0 | 2.0 | 15.6 |
| ENSTGUG00000010538 | HSPD1 | 0.179 | 0.032 | 24.0 | 6.0 | 15.6 |
| ENSTGUG00000005985 | GFM2 | 0.179 | 0.032 | 24.0 | 16.0 | 15.5 |
| ENSTGUG00000001415 | ADIPOR1 | 0.179 | 0.032 | 24.0 | 4.0 | 15.4 |
| ENSTGUG00000004380 | SULT2B1 | 0.179 | 0.032 | 24.0 | 4.0 | 15.2 |
| ENSTGUG00000000949 |  | 0.179 | 0.032 | 24.0 | 8.0 | 15.1 |
| ENSTGUG00000006811 | CENPV | 0.179 | 0.032 | 26.0 | 15.0 | 15.0 |
| ENSTGUG00000001879 | CA9 | 0.179 | 0.032 | 24.0 | 13.0 | 15.0 |
| ENSTGUG00000003968 | TBCA | 0.179 | 0.032 | 24.0 | 16.0 | 15.0 |
| ENSTGUG00000016161 |  | 0.179 | 0.032 | 24.0 | 1.0 | 14.6 |
| ENSTGUG00000003989 | CRIPT | 0.179 | 0.032 | 26.0 | 18.0 | 14.6 |
| ENSTGUG00000005048 | MINPP1 | 0.179 | 0.032 | 24.0 | 6.0 | 14.6 |
| ENSTGUG00000011415 | COMMD2 | 0.179 | 0.032 | 24.0 | 20.0 | 14.5 |
| ENSTGUG00000009782 | NCL | 0.179 | 0.032 | 24.0 | 0.0 | 14.5 |
| ENSTGUG00000006633 | USP4 | 0.179 | 0.032 | 25.3 | 8.0 | 14.2 |
| ENSTGUG00000005974 | NELL2 | 0.179 | 0.032 | 24.0 | 5.0 | 14.2 |
| ENSTGUG00000000121 | REXO2 | 0.179 | 0.032 | 24.0 | 2.0 | 14.0 |
| ENSTGUG00000008299 | FAM36A | 0.179 | 0.032 | 24.0 | 14.0 | 13.7 |
| ENSTGUG00000000815 | SRPR | 0.179 | 0.032 | 24.0 | 2.0 | 13.5 |
| ENSTGUG00000011630 | MRPS33 | 0.179 | 0.032 | 24.0 | 22.0 | 13.5 |
| ENSTGUG00000008503 | HAL | 0.179 | 0.032 | 24.0 | 22.0 | 13.5 |
| ENSTGUG00000012986 | C11orf73 | 0.179 | 0.032 | 25.3 | 20.7 | 13.4 |
| ENSTGUG00000008804 | STAG3L3 | 0.179 | 0.032 | 24.0 | 17.0 | 13.3 |
| ENSTGUG00000010118 | SMPD4 | 0.179 | 0.032 | 24.0 | 0.0 | 13.3 |
| ENSTGUG00000013374 | RABL3 | 0.179 | 0.032 | 24.0 | 16.0 | 13.2 |
| ENSTGUG00000010266 | CTH | 0.179 | 0.032 | 24.0 | 1.0 | 13.2 |
| ENSTGUG00000002212 | PPCS | 0.179 | 0.032 | 24.0 | 0.0 | 13.2 |
| ENSTGUG00000007248 | GLOD4 | 0.179 | 0.032 | 24.0 | 4.0 | 13.2 |
| ENSTGUG00000003960 |  | 0.179 | 0.032 | 24.0 | 12.0 | 13.1 |
| ENSTGUG00000004588 | PFKFB4 | 0.179 | 0.032 | 26.0 | 18.0 | 12.8 |
| ENSTGUG00000000543 | NOP16 | 0.179 | 0.032 | 25.3 | 20.7 | 12.7 |
| ENSTGUG00000012395 | STRAP | 0.179 | 0.032 | 26.0 | 18.0 | 12.7 |
| ENSTGUG00000006045 | C21orf33 | 0.179 | 0.032 | 24.0 | 2.0 | 12.6 |
| ENSTGUG00000001611 | SLC4A4 | 0.179 | 0.032 | 24.0 | 0.0 | 12.5 |
| ENSTGUG00000000826 | AHCYL1 | 0.179 | 0.032 | 24.0 | 1.0 | 12.5 |
| ENSTGUG00000007378 | COPS3 | 0.179 | 0.032 | 25.3 | 20.7 | 12.3 |
| ENSTGUG00000001009 | RNF130 | 0.179 | 0.032 | 24.0 | 12.0 | 12.3 |
| ENSTGUG00000000535 | CLTB | 0.179 | 0.032 | 24.0 | 4.0 | 12.2 |
| ENSTGUG00000007831 | MXRA7 | 0.179 | 0.032 | 24.0 | 10.0 | 12.2 |
| ENSTGUG00000005018 | COQ9 | 0.179 | 0.032 | 24.0 | 1.0 | 12.2 |
| ENSTGUG00000005881 | ATP6V0D1 | 0.179 | 0.032 | 24.0 | 1.0 | 12.1 |
| ENSTGUG00000005410 | YPEL5 | 0.179 | 0.032 | 24.0 | 0.0 | 12.0 |
| ENSTGUG00000014720 |  | 0.179 | 0.032 | 24.0 | 2.0 | 11.7 |
| ENSTGUG00000004912 | EIF3M | 0.179 | 0.032 | 24.0 | 21.0 | 11.7 |
| ENSTGUG00000008309 | HNRNPU | 0.179 | 0.032 | 25.3 | 16.7 | 11.7 |
| ENSTGUG00000017267 | PPIH | 0.179 | 0.032 | 24.0 | 18.0 | 11.6 |
| ENSTGUG00000002204 | CDC123 | 0.179 | 0.032 | 24.0 | 20.0 | 11.5 |
| ENSTGUG00000011147 | RASSF6 | 0.179 | 0.032 | 28.0 | 16.0 | 11.5 |
| ENSTGUG00000008209 | TMEM184A | 0.179 | 0.032 | 26.0 | 15.0 | 11.4 |
| ENSTGUG00000010583 | FGFR3 | 0.179 | 0.032 | 24.0 | 10.0 | 11.4 |
| ENSTGUG00000010898 | RPIA | 0.179 | 0.032 | 24.0 | 12.0 | 11.4 |
| ENSTGUG00000011369 | MED6 | 0.179 | 0.032 | 24.0 | 17.0 | 11.4 |
| ENSTGUG00000011556 | ATP6V1D | 0.179 | 0.032 | 24.0 | 2.0 | 11.3 |
| ENSTGUG00000003586 | PBLD | 0.179 | 0.032 | 24.0 | 14.0 | 11.3 |
| ENSTGUG00000004957 |  | 0.179 | 0.032 | 24.0 | 18.0 | 11.2 |
| ENSTGUG00000000804 | EI24 | 0.179 | 0.032 | 24.0 | 5.0 | 11.2 |
| ENSTGUG00000002820 | LYPLAL1 | 0.179 | 0.032 | 24.0 | 21.0 | 11.2 |
| ENSTGUG00000011437 | MIPEP | 0.179 | 0.032 | 24.0 | 12.0 | 11.2 |
| ENSTGUG00000002674 | MIA3 | 0.179 | 0.032 | 24.0 | 21.0 | 11.2 |
| ENSTGUG00000010912 | CCDC28A | 0.179 | 0.032 | 24.0 | 20.0 | 11.2 |
| ENSTGUG00000006737 | PTDSS2 | 0.179 | 0.032 | 24.0 | 5.0 | 11.1 |
| ENSTGUG00000000630 | ISCA1 | 0.179 | 0.032 | 24.0 | 16.0 | 11.1 |
| ENSTGUG00000016769 | NOL9 | 0.179 | 0.032 | 24.0 | 0.0 | 11.1 |
| ENSTGUG00000011285 | ADHFE1 | 0.179 | 0.032 | 24.0 | 16.0 | 11.1 |
| ENSTGUG00000009570 | PSMG2 | 0.179 | 0.032 | 24.0 | 21.0 | 11.0 |
| ENSTGUG00000011567 | STEAP3 | 0.179 | 0.032 | 25.3 | 8.0 | 10.9 |
| ENSTGUG00000009262 | CCDC50 | 0.179 | 0.032 | 24.0 | 5.0 | 10.8 |
| ENSTGUG00000005274 | MRPS22 | 0.179 | 0.032 | 24.0 | 17.0 | 10.8 |
| ENSTGUG00000000592 | PPAP2C | 0.179 | 0.032 | 26.0 | 5.0 | 10.7 |
| ENSTGUG00000001068 | WAC | 0.179 | 0.032 | 24.0 | 10.0 | 10.7 |
| ENSTGUG00000010422 | SPI1 | 0.179 | 0.032 | 24.0 | 8.0 | 10.6 |
| ENSTGUG00000003700 | DUS1L | 0.179 | 0.032 | 24.0 | 16.0 | 10.5 |
| ENSTGUG00000012616 | C14orf109 | 0.179 | 0.032 | 24.0 | 12.0 | 10.5 |
| ENSTGUG00000004139 | KALRN | 0.179 | 0.032 | 25.3 | 9.3 | 10.5 |
| ENSTGUG00000001797 | IP6K3 | 0.179 | 0.032 | 24.0 | 0.0 | 10.3 |
| ENSTGUG00000010758 | MEP1B | 0.179 | 0.032 | 24.0 | 12.0 | 10.3 |
| ENSTGUG00000009902 | YWHAH | 0.179 | 0.032 | 24.0 | 12.0 | 10.1 |
| ENSTGUG00000006526 | C11orf79 | 0.179 | 0.032 | 24.0 | 4.0 | 10.1 |
| ENSTGUG00000012284 | LCP1 | 0.179 | 0.032 | 24.0 | 13.0 | 10.1 |
| ENSTGUG00000001632 | PFDN1 | 0.179 | 0.032 | 24.0 | 4.0 | 10.0 |
| ENSTGUG00000007957 | MESDC2 | 0.179 | 0.032 | 24.0 | 14.0 | 9.9 |
| ENSTGUG00000004977 | PTEN | 0.179 | 0.032 | 24.0 | 10.0 | 9.8 |
| ENSTGUG00000015989 |  | 0.179 | 0.032 | 25.3 | 20.7 | 9.4 |
| ENSTGUG00000009809 | SPATA2L | 0.179 | 0.032 | 26.0 | 2.0 | 9.3 |
| ENSTGUG00000002914 | FAM104A | 0.179 | 0.032 | 24.0 | 17.0 | 9.3 |
| ENSTGUG00000000803 | C19orf10 | 0.179 | 0.032 | 24.0 | 2.0 | 9.3 |
| ENSTGUG00000009976 | CTSH | 0.179 | 0.032 | 24.0 | 5.0 | 9.2 |
| ENSTGUG00000010213 | C1orf31 | 0.179 | 0.032 | 24.0 | 20.0 | 9.2 |
| ENSTGUG00000000801 | LAPTM5 | 0.179 | 0.032 | 24.0 | 5.0 | 9.2 |
| ENSTGUG00000005031 |  | 0.179 | 0.032 | 24.0 | 21.0 | 9.2 |
| ENSTGUG00000006274 | MAOA | 0.179 | 0.032 | 24.0 | 21.0 | 9.2 |
| ENSTGUG00000004063 | SET | 0.179 | 0.032 | 25.3 | 20.7 | 9.1 |
| ENSTGUG00000000681 | C5orf4 | 0.179 | 0.032 | 24.0 | 14.0 | 9.1 |
| ENSTGUG00000000400 | CDC42SE2 | 0.179 | 0.032 | 24.0 | 16.0 | 9.0 |
| ENSTGUG00000009948 | NPM3 | 0.179 | 0.032 | 28.0 | 2.0 | 9.0 |
| ENSTGUG00000009201 | TMEM140 | 0.179 | 0.032 | 24.0 | 1.0 | 9.0 |
| ENSTGUG00000010217 | RPS19BP1 | 0.179 | 0.032 | 24.0 | 17.0 | 8.9 |
| ENSTGUG00000001813 |  | 0.179 | 0.032 | 24.0 | 9.0 | 8.8 |
| ENSTGUG00000004662 | FUBP3 | 0.179 | 0.032 | 25.3 | 20.7 | 8.7 |
| ENSTGUG00000004694 | TEX2 | 0.179 | 0.032 | 24.0 | 9.0 | 8.7 |
| ENSTGUG00000008599 | NR2F2 | 0.179 | 0.032 | 24.0 | 10.0 | 8.7 |
| ENSTGUG00000003132 | OXNAD1 | 0.179 | 0.032 | 24.0 | 16.0 | 8.7 |
| ENSTGUG00000009062 | BLNK | 0.179 | 0.032 | 24.0 | 14.0 | 8.7 |
| ENSTGUG00000003130 | HIP1 | 0.179 | 0.032 | 25.3 | 8.0 | 8.7 |
| ENSTGUG00000007370 | CCDC107 | 0.179 | 0.032 | 24.0 | 22.0 | 8.7 |
| ENSTGUG00000010323 | CLCN2 | 0.179 | 0.032 | 28.0 | 2.0 | 8.5 |
| ENSTGUG00000007105 | EIF3F | 0.179 | 0.032 | 24.0 | 2.0 | 8.5 |
| ENSTGUG00000011341 | MBNL1 | 0.179 | 0.032 | 24.0 | 5.0 | 8.5 |
| ENSTGUG00000004804 | DDHD2 | 0.179 | 0.032 | 28.0 | 2.0 | 8.5 |
| ENSTGUG00000011306 |  | 0.179 | 0.032 | 26.0 | 2.0 | 8.5 |
| ENSTGUG00000003829 | EIF4B | 0.179 | 0.032 | 24.0 | 1.0 | 8.4 |
| ENSTGUG00000007942 | SSB | 0.179 | 0.032 | 24.0 | 20.0 | 8.4 |
| ENSTGUG00000003633 | TOR2A | 0.179 | 0.032 | 24.0 | 22.0 | 8.3 |
| ENSTGUG00000005818 | NCOA4 | 0.179 | 0.032 | 24.0 | 1.0 | 8.3 |
| ENSTGUG00000008612 | GRB2 | 0.179 | 0.032 | 24.0 | 2.0 | 8.3 |
| ENSTGUG00000006033 | TINP1 | 0.179 | 0.032 | 24.0 | 22.0 | 8.3 |
| ENSTGUG00000000438 | FGFR4 | 0.179 | 0.032 | 24.0 | 4.0 | 8.3 |
| ENSTGUG00000010755 | RNF138 | 0.179 | 0.032 | 25.3 | 12.7 | 8.3 |
| ENSTGUG00000017473 | PPP1R15B | 0.179 | 0.032 | 26.0 | 2.0 | 8.2 |
| ENSTGUG00000006568 | KLHL30 | 0.179 | 0.032 | 25.3 | 8.0 | 8.2 |
| ENSTGUG00000007945 | C12orf29 | 0.179 | 0.032 | 24.0 | 14.0 | 8.0 |
| ENSTGUG00000007191 | GRHPR | 0.179 | 0.032 | 26.0 | 5.0 | 7.9 |
| ENSTGUG00000008471 | BRD7 | 0.179 | 0.032 | 24.0 | 14.0 | 7.9 |
| ENSTGUG00000002098 | LARP2 | 0.179 | 0.032 | 24.0 | 20.0 | 7.9 |
| ENSTGUG00000012185 | LRP12 | 0.179 | 0.032 | 24.0 | 1.0 | 7.8 |
| ENSTGUG00000006339 | FA2H | 0.179 | 0.032 | 24.0 | 5.0 | 7.8 |
| ENSTGUG00000003598 | ASPSCR1 | 0.179 | 0.032 | 24.0 | 10.0 | 7.8 |
| ENSTGUG00000011656 | DENND2A | 0.179 | 0.032 | 24.0 | 9.0 | 7.7 |
| ENSTGUG00000003308 | WDR61 | 0.179 | 0.032 | 25.3 | 16.7 | 7.6 |
| ENSTGUG00000011231 | CCNL1 | 0.179 | 0.032 | 28.0 | 2.0 | 7.6 |
| ENSTGUG00000017165 |  | 0.179 | 0.032 | 24.0 | 17.0 | 7.5 |
| ENSTGUG00000008687 |  | 0.179 | 0.032 | 24.0 | 13.0 | 7.5 |
| ENSTGUG00000002751 | PSMC2 | 0.179 | 0.032 | 24.0 | 22.0 | 7.4 |
| ENSTGUG00000013553 | MRPL39 | 0.179 | 0.032 | 24.0 | 18.0 | 7.4 |
| ENSTGUG00000014811 |  | 0.179 | 0.032 | 24.0 | 1.0 | 7.4 |
| ENSTGUG00000007436 | NDUFS8 | 0.179 | 0.032 | 24.0 | 4.0 | 7.4 |
| ENSTGUG00000000010 | HMBS | 0.179 | 0.032 | 24.0 | 9.0 | 7.4 |
| ENSTGUG00000009467 | SMAD6 | 0.179 | 0.032 | 24.0 | 4.0 | 7.4 |
| ENSTGUG00000004362 | CRAT | 0.179 | 0.032 | 24.0 | 22.0 | 7.3 |
| ENSTGUG00000009599 | CLN6 | 0.179 | 0.032 | 24.0 | 8.0 | 7.3 |
| ENSTGUG00000011483 | LACTB2 | 0.179 | 0.032 | 28.0 | 16.0 | 7.3 |
| ENSTGUG00000013321 | MSRA | 0.179 | 0.032 | 24.0 | 0.0 | 7.3 |
| ENSTGUG00000004591 | EIF4EBP2 | 0.179 | 0.032 | 24.0 | 4.0 | 7.2 |
| ENSTGUG00000005122 |  | 0.179 | 0.032 | 28.0 | 2.0 | 7.2 |
| ENSTGUG00000007119 | PSMB7 | 0.179 | 0.032 | 25.3 | 4.0 | 7.1 |
| ENSTGUG00000009010 | EPN3 | 0.179 | 0.032 | 26.0 | 5.0 | 7.1 |
| ENSTGUG00000008316 | TRIP12 | 0.179 | 0.032 | 24.0 | 18.0 | 7.1 |
| ENSTGUG00000009682 | SMOC2 | 0.179 | 0.032 | 24.0 | 9.0 | 7.1 |
| ENSTGUG00000004792 | C16orf57 | 0.179 | 0.032 | 24.0 | 5.0 | 7.0 |
| ENSTGUG00000011932 | RWDD1 | 0.179 | 0.032 | 24.0 | 14.0 | 7.0 |
| ENSTGUG00000002759 | IPO11 | 0.179 | 0.032 | 26.0 | 18.0 | 7.0 |
| ENSTGUG00000000980 | NUDC | 0.179 | 0.032 | 24.0 | 1.0 | 7.0 |
| ENSTGUG00000013801 | FAM50B | 0.179 | 0.032 | 24.0 | 2.0 | 7.0 |
| ENSTGUG00000011795 | STXBP6 | 0.179 | 0.032 | 24.0 | 13.0 | 7.0 |
| ENSTGUG00000004888 | RAD54L2 | 0.179 | 0.032 | 24.0 | 12.0 | 6.9 |
| ENSTGUG00000005433 | GTF2A2 | 0.179 | 0.032 | 25.3 | 16.7 | 6.9 |
| ENSTGUG00000013591 | CD200 | 0.179 | 0.032 | 24.0 | 0.0 | 6.8 |
| ENSTGUG00000010712 | MAPK8IP1 | 0.179 | 0.032 | 26.0 | 5.0 | 6.8 |
| ENSTGUG00000000067 | LSM7 | 0.179 | 0.032 | 24.0 | 20.0 | 6.8 |
| ENSTGUG00000002442 | NARG1 | 0.179 | 0.032 | 26.0 | 18.0 | 6.8 |
| ENSTGUG00000004211 | CLCN7 | 0.179 | 0.032 | 24.0 | 1.0 | 6.8 |
| ENSTGUG00000016284 | GRINA | 0.179 | 0.032 | 24.0 | 2.0 | 6.7 |
| ENSTGUG00000002727 | TUBG1 | 0.179 | 0.032 | 24.0 | 2.0 | 6.6 |
| ENSTGUG00000010746 | MYH9 | 0.179 | 0.032 | 28.0 | 2.0 | 6.6 |
| ENSTGUG00000010343 |  | 0.179 | 0.032 | 24.0 | 21.0 | 6.6 |
| ENSTGUG00000002778 | PMPCB | 0.179 | 0.032 | 26.0 | 18.0 | 6.5 |
| ENSTGUG00000004149 | B5FZA9_TAEGU | 0.179 | 0.032 | 24.0 | 9.0 | 6.5 |
| ENSTGUG00000005056 | GBAS | 0.179 | 0.032 | 26.0 | 2.0 | 6.5 |
| ENSTGUG00000010022 | LEPROT | 0.179 | 0.032 | 24.0 | 8.0 | 6.5 |
| ENSTGUG00000017296 | ATP6V1B2 | 0.179 | 0.032 | 24.0 | 5.0 | 6.4 |
| ENSTGUG00000012451 | PSMC1 | 0.179 | 0.032 | 24.0 | 22.0 | 6.3 |
| ENSTGUG00000011931 | CFL2 | 0.179 | 0.032 | 24.0 | 21.0 | 6.3 |
| ENSTGUG00000013798 |  | 0.179 | 0.032 | 24.0 | 1.0 | 6.3 |
| ENSTGUG00000001773 | SNRPC | 0.179 | 0.032 | 24.0 | 2.0 | 6.3 |
| ENSTGUG00000007095 | NEK6 | 0.179 | 0.032 | 24.0 | 12.0 | 6.3 |
| ENSTGUG00000001335 | FEM1C | 0.179 | 0.032 | 24.0 | 1.0 | 6.3 |
| ENSTGUG00000013436 | TIPRL | 0.179 | 0.032 | 24.0 | 17.0 | 6.2 |
| ENSTGUG00000002982 | PIK3R1 | 0.179 | 0.032 | 24.0 | 22.0 | 6.2 |
| ENSTGUG00000014293 |  | 0.179 | 0.032 | 26.0 | 18.0 | 6.2 |
| ENSTGUG00000008963 | IGSF6 | 0.179 | 0.032 | 24.0 | 9.0 | 6.2 |
| ENSTGUG00000011754 | C15orf41 | 0.179 | 0.032 | 24.0 | 6.0 | 6.2 |
| ENSTGUG00000004108 | UCHL5 | 0.179 | 0.032 | 24.0 | 20.0 | 6.2 |
| ENSTGUG00000000850 | RBM22 | 0.179 | 0.032 | 24.0 | 2.0 | 6.2 |
| ENSTGUG00000011263 | C6orf211 | 0.179 | 0.032 | 24.0 | 0.0 | 6.1 |
| ENSTGUG00000013233 |  | 0.179 | 0.032 | 24.0 | 12.0 | 6.1 |
| ENSTGUG00000000083 | UBE4A | 0.179 | 0.032 | 24.0 | 1.0 | 6.1 |
| ENSTGUG00000012833 |  | 0.179 | 0.032 | 24.0 | 6.0 | 6.1 |
| ENSTGUG00000006941 | SHPK | 0.179 | 0.032 | 24.0 | 12.0 | 6.0 |
| ENSTGUG00000001132 | CLIC4 | 0.179 | 0.032 | 28.0 | 2.0 | 6.0 |
| ENSTGUG00000007714 | 9-Sep | 0.179 | 0.032 | 24.0 | 2.0 | 6.0 |
| ENSTGUG00000002166 | MRPS5 | 0.179 | 0.032 | 26.0 | 18.0 | 6.0 |
| ENSTGUG00000017490 | NEDD4L | 0.179 | 0.032 | 24.0 | 12.0 | 5.9 |
| ENSTGUG00000001546 | SERF1B | 0.179 | 0.032 | 24.0 | 0.0 | 5.9 |
| ENSTGUG00000009080 | ARIH2 | 0.179 | 0.032 | 24.0 | 9.0 | 5.9 |
| ENSTGUG00000009561 | GATC | 0.179 | 0.032 | 24.0 | 14.0 | 5.9 |
| ENSTGUG00000006604 | C20orf57 | 0.179 | 0.032 | 24.0 | 2.0 | 5.8 |
| ENSTGUG00000001398 | CEPT1 | 0.179 | 0.032 | 24.0 | 0.0 | 5.8 |
| ENSTGUG00000005170 | ARPC2 | 0.179 | 0.032 | 24.0 | 2.0 | 5.7 |
| ENSTGUG00000012644 | TMEM30A | 0.179 | 0.032 | 24.0 | 12.0 | 5.7 |
| ENSTGUG00000014923 |  | 0.179 | 0.032 | 24.0 | 2.0 | 5.7 |
| ENSTGUG00000015833 | USP36 | 0.179 | 0.032 | 24.0 | 0.0 | 5.7 |
| ENSTGUG00000005028 |  | 0.179 | 0.032 | 24.0 | 2.0 | 5.7 |
| ENSTGUG00000009090 | MTFMT | 0.179 | 0.032 | 26.0 | 18.0 | 5.6 |
| ENSTGUG00000016507 | RUSC1 | 0.179 | 0.032 | 24.0 | 1.0 | 5.6 |
| ENSTGUG00000004938 | PPP2R5D | 0.179 | 0.032 | 24.0 | 9.0 | 5.6 |
| ENSTGUG00000005493 | RQCD1 | 0.179 | 0.032 | 24.0 | 1.0 | 5.6 |
| ENSTGUG00000004613 |  | 0.179 | 0.032 | 24.0 | 14.0 | 5.5 |
| ENSTGUG00000001295 | TCF7 | 0.179 | 0.032 | 24.0 | 4.0 | 5.5 |
| ENSTGUG00000005019 | PAPSS2 | 0.179 | 0.032 | 24.0 | 4.0 | 5.4 |
| ENSTGUG00000005283 | EIF2B1 | 0.179 | 0.032 | 24.0 | 18.0 | 5.4 |
| ENSTGUG00000004223 | BNIP3L | 0.179 | 0.032 | 24.0 | 20.0 | 5.4 |
| ENSTGUG00000007064 | MTIF2 | 0.179 | 0.032 | 26.0 | 15.0 | 5.4 |
| ENSTGUG00000006265 | PMPCA | 0.179 | 0.032 | 24.0 | 22.0 | 5.4 |
| ENSTGUG00000005773 | POLR2D | 0.179 | 0.032 | 24.0 | 10.0 | 5.3 |
| ENSTGUG00000000732 | TTC37 | 0.179 | 0.032 | 26.0 | 18.0 | 5.3 |
| ENSTGUG00000008449 | ADAT1 | 0.179 | 0.032 | 24.0 | 13.0 | 5.3 |
| ENSTGUG00000005082 | TACC1 | 0.179 | 0.032 | 26.0 | 5.0 | 5.3 |
| ENSTGUG00000010662 | CTBP1 | 0.179 | 0.032 | 24.0 | 5.0 | 5.3 |
| ENSTGUG00000007411 | SMS | 0.179 | 0.032 | 24.0 | 13.0 | 5.3 |
| ENSTGUG00000007309 | TUSC3 | 0.179 | 0.032 | 24.0 | 1.0 | 5.2 |
| ENSTGUG00000002046 |  | 0.179 | 0.032 | 24.0 | 21.0 | 5.2 |
| ENSTGUG00000008271 | ABHD2 | 0.179 | 0.032 | 24.0 | 20.0 | 5.2 |
| ENSTGUG00000014251 | DUT | 0.179 | 0.032 | 24.0 | 17.0 | 5.2 |
| ENSTGUG00000012944 | PCF11 | 0.179 | 0.032 | 24.0 | 22.0 | 5.2 |
| ENSTGUG00000009771 | TM2D1 | 0.179 | 0.032 | 24.0 | 13.0 | 5.2 |
| ENSTGUG00000004120 | FAM100A | 0.179 | 0.032 | 24.0 | 0.0 | 5.2 |
| ENSTGUG00000016248 | SAPS2 | 0.179 | 0.032 | 26.0 | 5.0 | 5.2 |
| ENSTGUG00000005002 | TPM1 | 0.179 | 0.032 | 24.0 | 9.0 | 5.2 |
| ENSTGUG00000012099 | UBR5 | 0.179 | 0.032 | 24.0 | 8.0 | 5.2 |
| ENSTGUG00000004360 | AMMECR1 | 0.179 | 0.032 | 24.0 | 9.0 | 5.1 |
| ENSTGUG00000013837 |  | 0.179 | 0.032 | 28.0 | 16.0 | 5.1 |
| ENSTGUG00000009903 | FBXW4 | 0.179 | 0.032 | 24.0 | 5.0 | 5.0 |
| ENSTGUG00000012555 | FBXL3 | 0.179 | 0.032 | 24.0 | 21.0 | 5.0 |
| ENSTGUG00000011798 | HDDC2 | 0.179 | 0.032 | 24.0 | 16.0 | 5.0 |
| ENSTGUG00000002814 | VSIG4 | 0.179 | 0.032 | 24.0 | 8.0 | 4.9 |
| ENSTGUG00000000527 | FAF2 | 0.179 | 0.032 | 24.0 | 0.0 | 4.9 |
| ENSTGUG00000004545 | FNBP1 | 0.179 | 0.032 | 24.0 | 8.0 | 4.9 |
| ENSTGUG00000017261 | TADA1L | 0.179 | 0.032 | 24.0 | 0.0 | 4.9 |
| ENSTGUG00000005518 | INPP5K | 0.179 | 0.032 | 24.0 | 8.0 | 4.9 |
| ENSTGUG00000002148 | ECE1 | 0.179 | 0.032 | 24.0 | 0.0 | 4.8 |
| ENSTGUG00000001613 |  | 0.179 | 0.032 | 26.0 | 5.0 | 4.8 |
| ENSTGUG00000002085 | DDOST | 0.179 | 0.032 | 24.0 | 2.0 | 4.8 |
| ENSTGUG00000011460 | SLC22A2 | 0.179 | 0.032 | 24.0 | 22.0 | 4.8 |
| ENSTGUG00000005940 | ATG4B | 0.179 | 0.032 | 26.0 | 5.0 | 4.8 |
| ENSTGUG00000005603 | CTCF | 0.179 | 0.032 | 24.0 | 22.0 | 4.8 |
| ENSTGUG00000012243 |  | 0.179 | 0.032 | 24.0 | 9.0 | 4.7 |
| ENSTGUG00000008566 | CASKIN2 | 0.179 | 0.032 | 24.0 | 5.0 | 4.7 |
| ENSTGUG00000008897 | KLB | 0.179 | 0.032 | 24.0 | 2.0 | 4.7 |
| ENSTGUG00000000073 | SLC4A1-1 | 0.179 | 0.032 | 24.0 | 2.0 | 4.7 |
| ENSTGUG00000002450 | ESF1 | 0.179 | 0.032 | 26.0 | 18.0 | 4.7 |
| ENSTGUG00000015263 |  | 0.179 | 0.032 | 24.0 | 9.0 | 4.7 |
| ENSTGUG00000006110 | RANBP9 | 0.179 | 0.032 | 24.0 | 21.0 | 4.7 |
| ENSTGUG00000011093 | EIF3A | 0.179 | 0.032 | 26.0 | 2.0 | 4.6 |
| ENSTGUG00000008988 | CPEB3 | 0.179 | 0.032 | 24.0 | 2.0 | 4.6 |
| ENSTGUG00000003485 | UBE2Q2 | 0.179 | 0.032 | 24.0 | 13.0 | 4.6 |
| ENSTGUG00000003163 | UBQLN1 | 0.179 | 0.032 | 24.0 | 16.0 | 4.6 |
| ENSTGUG00000002389 | USP38 | 0.179 | 0.032 | 24.0 | 14.0 | 4.6 |
| ENSTGUG00000002796 | EPRS | 0.179 | 0.032 | 24.0 | 12.0 | 4.5 |
| ENSTGUG00000003044 | NSL1 | 0.179 | 0.032 | 24.0 | 17.0 | 4.5 |
| ENSTGUG00000002360 | ZNF330 | 0.179 | 0.032 | 24.0 | 20.0 | 4.5 |
| ENSTGUG00000003224 | TELO2 | 0.179 | 0.032 | 25.3 | 20.7 | 4.5 |
| ENSTGUG00000007859 | 8-Mar | 0.179 | 0.032 | 24.0 | 9.0 | 4.4 |
| ENSTGUG00000010865 | PMS1 | 0.179 | 0.032 | 28.0 | 16.0 | 4.4 |
| ENSTGUG00000007139 | RAMP3 | 0.179 | 0.032 | 24.0 | 9.0 | 4.4 |
| ENSTGUG00000003209 | CCL5 | 0.179 | 0.032 | 24.0 | 14.0 | 4.4 |
| ENSTGUG00000000166 | GNPDA1 | 0.179 | 0.032 | 24.0 | 5.0 | 4.4 |
| ENSTGUG00000008176 | VAPB | 0.179 | 0.032 | 24.0 | 20.0 | 4.3 |
| ENSTGUG00000009272 | TFDP1 | 0.179 | 0.032 | 24.0 | 13.0 | 4.3 |
| ENSTGUG00000004626 |  | 0.179 | 0.032 | 24.0 | 13.0 | 4.3 |
| ENSTGUG00000003862 | ALG12 | 0.179 | 0.032 | 24.0 | 17.0 | 4.3 |
| ENSTGUG00000009265 |  | 0.179 | 0.032 | 24.0 | 22.0 | 4.3 |
| ENSTGUG00000007480 | TMEM150 | 0.179 | 0.032 | 28.0 | 2.0 | 4.2 |
| ENSTGUG00000014470 | VPS4A | 0.179 | 0.032 | 26.0 | 5.0 | 4.2 |
| ENSTGUG00000013777 |  | 0.179 | 0.032 | 25.3 | 20.7 | 4.2 |
| ENSTGUG00000009469 | SSFA2 | 0.179 | 0.032 | 24.0 | 0.0 | 4.2 |
| ENSTGUG00000001657 | ASNS | 0.179 | 0.032 | 24.0 | 10.0 | 4.2 |
| ENSTGUG00000006579 | WIF1 | 0.179 | 0.032 | 24.0 | 12.0 | 4.2 |
| ENSTGUG00000003246 | KAT2B | 0.179 | 0.032 | 24.0 | 12.0 | 4.2 |
| ENSTGUG00000017445 | MED20 | 0.179 | 0.032 | 24.0 | 4.0 | 4.2 |
| ENSTGUG00000010800 | ARGLU1 | 0.179 | 0.032 | 24.0 | 13.0 | 4.2 |
| ENSTGUG00000001097 | RUFY1 | 0.179 | 0.032 | 24.0 | 20.0 | 4.1 |
| ENSTGUG00000000906 | KLF6 | 0.179 | 0.032 | 24.0 | 8.0 | 4.1 |
| ENSTGUG00000005710 | TLCD2 | 0.179 | 0.032 | 24.0 | 4.0 | 4.1 |
| ENSTGUG00000006011 | PCIF1 | 0.179 | 0.032 | 26.0 | 2.0 | 4.1 |
| ENSTGUG00000006436 | BCAR1 | 0.179 | 0.032 | 24.0 | 2.0 | 4.1 |
| ENSTGUG00000008289 | FAM152A | 0.179 | 0.032 | 24.0 | 20.0 | 4.1 |
| ENSTGUG00000011293 |  | 0.179 | 0.032 | 25.3 | 12.7 | 4.1 |
| ENSTGUG00000001626 | PIK3C3 | 0.179 | 0.032 | 24.0 | 21.0 | 4.1 |
| ENSTGUG00000005237 | POLR1B | 0.179 | 0.032 | 26.0 | 5.0 | 4.1 |
| ENSTGUG00000001729 | DCTN3 | 0.179 | 0.032 | 24.0 | 4.0 | 4.0 |
| ENSTGUG00000013977 |  | 0.179 | 0.032 | 24.0 | 14.0 | 4.0 |
| ENSTGUG00000012559 | TRIP11 | 0.179 | 0.032 | 24.0 | 21.0 | 4.0 |
| ENSTGUG00000010568 | ZFYVE20 | 0.179 | 0.032 | 24.0 | 14.0 | 4.0 |
| ENSTGUG00000002015 | LMBRD2 | 0.179 | 0.032 | 26.0 | 18.0 | 4.0 |
| ENSTGUG00000015245 | ATRN | 0.179 | 0.032 | 24.0 | 20.0 | 4.0 |
| ENSTGUG00000008281 | SOCS2 | 0.179 | 0.032 | 24.0 | 1.0 | 4.0 |
| ENSTGUG00000002901 | DDX26B | 0.179 | 0.032 | 24.0 | 22.0 | 3.9 |
| ENSTGUG00000000058 | ACAA2 | 0.179 | 0.032 | 24.0 | 4.0 | 3.9 |
| ENSTGUG00000016736 |  | 0.179 | 0.032 | 24.0 | 20.0 | 3.9 |
| ENSTGUG00000012567 | SLA | 0.179 | 0.032 | 24.0 | 13.0 | 3.9 |
| ENSTGUG00000012980 | RNASEH1 | 0.179 | 0.032 | 24.0 | 17.0 | 3.9 |
| ENSTGUG00000007952 | MESDC1 | 0.179 | 0.032 | 25.3 | 8.0 | 3.8 |
| ENSTGUG00000010465 | NAT5 | 0.179 | 0.032 | 24.0 | 4.0 | 3.8 |
| ENSTGUG00000011136 | TFB1M | 0.179 | 0.032 | 24.0 | 14.0 | 3.8 |
| ENSTGUG00000012063 | CMAS | 0.179 | 0.032 | 25.3 | 20.7 | 3.8 |
| ENSTGUG00000001208 | COMMD3 | 0.179 | 0.032 | 24.0 | 14.0 | 3.8 |
| ENSTGUG00000011245 | SSR3 | 0.179 | 0.032 | 24.0 | 21.0 | 3.7 |
| ENSTGUG00000001604 | UBE2D2 | 0.179 | 0.032 | 24.0 | 13.0 | 3.7 |
| ENSTGUG00000012687 | PAPOLA | 0.179 | 0.032 | 24.0 | 14.0 | 3.7 |
| ENSTGUG00000005583 | RWDD3 | 0.179 | 0.032 | 26.0 | 18.0 | 3.7 |
| ENSTGUG00000004771 | AP2B1 | 0.179 | 0.032 | 24.0 | 1.0 | 3.7 |
| ENSTGUG00000011637 | PPP2R2D | 0.179 | 0.032 | 25.3 | 9.3 | 3.7 |
| ENSTGUG00000013030 | CLNS1A | 0.179 | 0.032 | 24.0 | 16.0 | 3.7 |
| ENSTGUG00000011140 | NMD3 | 0.179 | 0.032 | 24.0 | 22.0 | 3.6 |
| ENSTGUG00000001556 | CASD1 | 0.179 | 0.032 | 26.0 | 15.0 | 3.6 |
| ENSTGUG00000007359 | MMAB | 0.179 | 0.032 | 24.0 | 18.0 | 3.5 |
| ENSTGUG00000003773 | CERK | 0.179 | 0.032 | 24.0 | 12.0 | 3.5 |
| ENSTGUG00000006775 | PFKM-2 | 0.179 | 0.032 | 24.0 | 2.0 | 3.5 |
| ENSTGUG00000009855 | LDB1 | 0.179 | 0.032 | 24.0 | 2.0 | 3.5 |
| ENSTGUG00000008942 | TXNDC17 | 0.179 | 0.032 | 24.0 | 20.0 | 3.5 |
| ENSTGUG00000001005 | DENND2C | 0.179 | 0.032 | 24.0 | 9.0 | 3.5 |
| ENSTGUG00000011989 | NHLRC3 | 0.179 | 0.032 | 24.0 | 17.0 | 3.5 |
| ENSTGUG00000014655 |  | 0.179 | 0.032 | 28.0 | 2.0 | 3.4 |
| ENSTGUG00000013253 | CLSTN3 | 0.179 | 0.032 | 24.0 | 9.0 | 3.4 |
| ENSTGUG00000003643 | RHOT2 | 0.179 | 0.032 | 26.0 | 2.0 | 3.4 |
| ENSTGUG00000005796 | ADAL | 0.179 | 0.032 | 24.0 | 12.0 | 3.4 |
| ENSTGUG00000001844 |  | 0.179 | 0.032 | 24.0 | 14.0 | 3.4 |
| ENSTGUG00000005036 | CIAPIN1 | 0.179 | 0.032 | 24.0 | 22.0 | 3.4 |
| ENSTGUG00000013515 | PTGFRN | 0.179 | 0.032 | 26.0 | 2.0 | 3.4 |
| ENSTGUG00000009440 | CDC16 | 0.179 | 0.032 | 24.0 | 16.0 | 3.4 |
| ENSTGUG00000008234 | AGFG1 | 0.179 | 0.032 | 24.0 | 12.0 | 3.3 |
| ENSTGUG00000004389 | PTPRC | 0.179 | 0.032 | 24.0 | 10.0 | 3.3 |
| ENSTGUG00000000843 | CITED4 | 0.179 | 0.032 | 26.0 | 2.0 | 3.3 |
| ENSTGUG00000001211 | MLLT10 | 0.179 | 0.032 | 24.0 | 20.0 | 3.3 |
| ENSTGUG00000002610 | ALAD | 0.179 | 0.032 | 24.0 | 1.0 | 3.3 |
| ENSTGUG00000002572 | STAT5A | 0.179 | 0.032 | 24.0 | 2.0 | 3.2 |
| ENSTGUG00000001673 |  | 0.179 | 0.032 | 24.0 | 18.0 | 3.2 |
| ENSTGUG00000009368 | COX15 | 0.179 | 0.032 | 24.0 | 1.0 | 3.2 |
| ENSTGUG00000004356 | DOLPP1 | 0.179 | 0.032 | 24.0 | 5.0 | 3.2 |
| ENSTGUG00000012584 | SH3BGRL2 | 0.179 | 0.032 | 24.0 | 8.0 | 3.2 |
| ENSTGUG00000014272 |  | 0.179 | 0.032 | 28.0 | 16.0 | 3.2 |
| ENSTGUG00000001268 | CAMLG | 0.179 | 0.032 | 28.0 | 16.0 | 3.2 |
| ENSTGUG00000013168 | MAP4K5 | 0.179 | 0.032 | 24.0 | 12.0 | 3.1 |
| ENSTGUG00000006264 | BAP1 | 0.179 | 0.032 | 24.0 | 8.0 | 3.1 |
| ENSTGUG00000005262 | RANBP10 | 0.179 | 0.032 | 24.0 | 17.0 | 3.1 |
| ENSTGUG00000001465 | WDR77 | 0.179 | 0.032 | 24.0 | 0.0 | 3.1 |
| ENSTGUG00000002974 | PROX1 | 0.179 | 0.032 | 24.0 | 2.0 | 3.1 |
| ENSTGUG00000000536 | IFNAR1 | 0.179 | 0.032 | 24.0 | 0.0 | 3.1 |
| ENSTGUG00000006802 | RBM18 | 0.179 | 0.032 | 25.3 | 8.0 | 3.0 |
| ENSTGUG00000004214 | ACBD3 | 0.179 | 0.032 | 24.0 | 21.0 | 3.0 |
| ENSTGUG00000000721 |  | 0.179 | 0.032 | 25.3 | 8.0 | 3.0 |
| ENSTGUG00000006081 | ADAM10 | 0.179 | 0.032 | 24.0 | 1.0 | 3.0 |
| ENSTGUG00000000463 | WDR48 | 0.179 | 0.032 | 28.0 | 16.0 | 3.0 |
| ENSTGUG00000008567 | PPIL2 | 0.179 | 0.032 | 24.0 | 18.0 | 3.0 |
| ENSTGUG00000011223 |  | 0.179 | 0.032 | 24.0 | 16.0 | 3.0 |
| ENSTGUG00000009035 | TTRAP | 0.179 | 0.032 | 24.0 | 20.0 | 3.0 |
| ENSTGUG00000006532 | TM9SF4 | 0.179 | 0.032 | 24.0 | 1.0 | 2.9 |
| ENSTGUG00000009815 | COPS7B | 0.179 | 0.032 | 24.0 | 14.0 | 2.9 |
| ENSTGUG00000003721 | GOLGA2 | 0.179 | 0.032 | 26.0 | 2.0 | 2.9 |
| ENSTGUG00000010104 | GGT5 | 0.179 | 0.032 | 24.0 | 6.0 | 2.9 |
| ENSTGUG00000009565 | DNAJC10 | 0.179 | 0.032 | 24.0 | 22.0 | 2.9 |
| ENSTGUG00000013049 | NAT12 | 0.179 | 0.032 | 24.0 | 5.0 | 2.9 |
| ENSTGUG00000008195 | RAB22A | 0.179 | 0.032 | 24.0 | 4.0 | 2.9 |
| ENSTGUG00000009645 | FYTTD1 | 0.179 | 0.032 | 24.0 | 17.0 | 2.9 |
| ENSTGUG00000016268 |  | 0.179 | 0.032 | 24.0 | 12.0 | 2.9 |
| ENSTGUG00000008751 | B5G4P1_TAEGU | 0.179 | 0.032 | 24.0 | 2.0 | 2.9 |
| ENSTGUG00000011965 | PPP2R3C | 0.179 | 0.032 | 24.0 | 9.0 | 2.9 |
| ENSTGUG00000008082 | GNAS | 0.179 | 0.032 | 24.0 | 12.0 | 2.9 |
| ENSTGUG00000011899 | CD9 | 0.179 | 0.032 | 24.0 | 5.0 | 2.8 |
| ENSTGUG00000001382 | TEK | 0.179 | 0.032 | 24.0 | 6.0 | 2.8 |
| ENSTGUG00000006693 | MRPL21 | 0.179 | 0.032 | 24.0 | 12.0 | 2.8 |
| ENSTGUG00000005398 | TP53RK | 0.179 | 0.032 | 24.0 | 14.0 | 2.8 |
| ENSTGUG00000000426 | INSR | 0.179 | 0.032 | 24.0 | 4.0 | 2.8 |
| ENSTGUG00000007224 | PTPRF | 0.179 | 0.032 | 24.0 | 4.0 | 2.8 |
| ENSTGUG00000017279 | ITPA | 0.179 | 0.032 | 24.0 | 4.0 | 2.8 |
| ENSTGUG00000007091 | HUS1 | 0.179 | 0.032 | 24.0 | 14.0 | 2.8 |
| ENSTGUG00000012541 | SMEK1 | 0.179 | 0.032 | 24.0 | 9.0 | 2.8 |
| ENSTGUG00000013429 | BLZF1 | 0.179 | 0.032 | 26.0 | 18.0 | 2.7 |
| ENSTGUG00000009562 | KCTD5 | 0.179 | 0.032 | 24.0 | 17.0 | 2.7 |
| ENSTGUG00000005572 | FBXO11 | 0.179 | 0.032 | 24.0 | 13.0 | 2.7 |
| ENSTGUG00000017265 | C1orf9 | 0.179 | 0.032 | 24.0 | 0.0 | 2.7 |
| ENSTGUG00000000182 | NFIC | 0.179 | 0.032 | 24.0 | 0.0 | 2.7 |
| ENSTGUG00000003724 | SNUPN | 0.179 | 0.032 | 24.0 | 1.0 | 2.7 |
| ENSTGUG00000003899 | SMARCA1 | 0.179 | 0.032 | 24.0 | 13.0 | 2.7 |
| ENSTGUG00000000522 | C9orf102-1 | 0.179 | 0.032 | 24.0 | 13.0 | 2.7 |
| ENSTGUG00000010551 | IMPACT | 0.179 | 0.032 | 24.0 | 17.0 | 2.7 |
| ENSTGUG00000010628 | AMBRA1 | 0.179 | 0.032 | 25.3 | 8.0 | 2.7 |
| ENSTGUG00000018219 |  | 0.179 | 0.032 | 24.0 | 6.0 | 2.6 |
| ENSTGUG00000008291 | COMMD8 | 0.179 | 0.032 | 24.0 | 16.0 | 2.6 |
| ENSTGUG00000004084 | F2RL1 | 0.179 | 0.032 | 24.0 | 21.0 | 2.6 |
| ENSTGUG00000014190 | CDK7 | 0.179 | 0.032 | 24.0 | 17.0 | 2.6 |
| ENSTGUG00000011170 | C21orf70 | 0.179 | 0.032 | 24.0 | 2.0 | 2.6 |
| ENSTGUG00000004758 | GAS2L2 | 0.179 | 0.032 | 26.0 | 5.0 | 2.6 |
| ENSTGUG00000014753 | PPM1G | 0.179 | 0.032 | 24.0 | 4.0 | 2.6 |
| ENSTGUG00000012273 | KIAA1737 | 0.179 | 0.032 | 24.0 | 13.0 | 2.6 |
| ENSTGUG00000011292 | MPHOSPH8 | 0.179 | 0.032 | 24.0 | 14.0 | 2.6 |
| ENSTGUG00000002668 | AIDA | 0.179 | 0.032 | 24.0 | 9.0 | 2.6 |
| ENSTGUG00000011019 | RIC8B | 0.179 | 0.032 | 26.0 | 15.0 | 2.6 |
| ENSTGUG00000006613 | LLPH | 0.179 | 0.032 | 24.0 | 0.0 | 2.6 |
| ENSTGUG00000012439 | ORC3L | 0.179 | 0.032 | 24.0 | 20.0 | 2.6 |
| ENSTGUG00000012012 | SLC25A15 | 0.179 | 0.032 | 24.0 | 12.0 | 2.6 |
| ENSTGUG00000011359 |  | 0.179 | 0.032 | 24.0 | 12.0 | 2.5 |
| ENSTGUG00000011478 | C12orf72 | 0.179 | 0.032 | 24.0 | 14.0 | 2.5 |
| ENSTGUG00000007269 | BASP1 | 0.179 | 0.032 | 24.0 | 8.0 | 2.5 |
| ENSTGUG00000003244 | NDUFA10 | 0.179 | 0.032 | 24.0 | 6.0 | 2.5 |
| ENSTGUG00000006637 | FAM82A2 | 0.179 | 0.032 | 24.0 | 1.0 | 2.5 |
| ENSTGUG00000008609 | TMEM188 | 0.179 | 0.032 | 24.0 | 14.0 | 2.5 |
| ENSTGUG00000014139 |  | 0.179 | 0.032 | 25.3 | 8.0 | 2.5 |
| ENSTGUG00000007426 | RBL2 | 0.179 | 0.032 | 24.0 | 21.0 | 2.5 |
| ENSTGUG00000010167 | KLHL22 | 0.179 | 0.032 | 24.0 | 20.0 | 2.5 |
| ENSTGUG00000006061 | PALLD | 0.179 | 0.032 | 24.0 | 1.0 | 2.5 |
| ENSTGUG00000016099 |  | 0.179 | 0.032 | 24.0 | 13.0 | 2.5 |
| ENSTGUG00000005312 | EDC4 | 0.179 | 0.032 | 24.0 | 2.0 | 2.5 |
| ENSTGUG00000001942 |  | 0.179 | 0.032 | 24.0 | 1.0 | 2.5 |
| ENSTGUG00000015333 |  | 0.179 | 0.032 | 24.0 | 9.0 | 2.5 |
| ENSTGUG00000012206 | PREP | 0.179 | 0.032 | 24.0 | 21.0 | 2.5 |
| ENSTGUG00000007896 | APPBP2 | 0.179 | 0.032 | 24.0 | 18.0 | 2.5 |
| ENSTGUG00000012558 | PHF20L1 | 0.179 | 0.032 | 24.0 | 20.0 | 2.5 |
| ENSTGUG00000005167 | AIFM1 | 0.179 | 0.032 | 24.0 | 1.0 | 2.5 |
| ENSTGUG00000000409 |  | 0.179 | 0.032 | 26.0 | 2.0 | 2.4 |
| ENSTGUG00000003061 | TBL2 | 0.179 | 0.032 | 24.0 | 22.0 | 2.4 |
| ENSTGUG00000008196 | DHX38 | 0.179 | 0.032 | 24.0 | 1.0 | 2.4 |
| ENSTGUG00000005335 |  | 0.179 | 0.032 | 24.0 | 17.0 | 2.4 |
| ENSTGUG00000007253 | CNOT7 | 0.179 | 0.032 | 24.0 | 14.0 | 2.4 |
| ENSTGUG00000003841 | CLUAP1 | 0.179 | 0.032 | 25.3 | 16.7 | 2.4 |
| ENSTGUG00000003846 | SCAMP1 | 0.179 | 0.032 | 24.0 | 16.0 | 2.4 |
| ENSTGUG00000009578 | MLLT4 | 0.179 | 0.032 | 24.0 | 4.0 | 2.4 |
| ENSTGUG00000011647 | ZBTB10 | 0.179 | 0.032 | 24.0 | 1.0 | 2.4 |
| ENSTGUG00000001808 |  | 0.179 | 0.032 | 24.0 | 6.0 | 2.4 |
| ENSTGUG00000006647 | TANK | 0.179 | 0.032 | 24.0 | 18.0 | 2.3 |
| ENSTGUG00000009083 |  | 0.179 | 0.032 | 24.0 | 17.0 | 2.3 |
| ENSTGUG00000017332 | SMAP2 | 0.179 | 0.032 | 24.0 | 12.0 | 2.3 |
| ENSTGUG00000010246 | ARID4B | 0.179 | 0.032 | 24.0 | 21.0 | 2.3 |
| ENSTGUG00000008305 | RAE1 | 0.179 | 0.032 | 24.0 | 0.0 | 2.3 |
| ENSTGUG00000007351 | SLC30A4 | 0.179 | 0.032 | 24.0 | 16.0 | 2.3 |
| ENSTGUG00000013633 | TBC1D23 | 0.179 | 0.032 | 24.0 | 20.0 | 2.3 |
| ENSTGUG00000001036 | MCC | 0.179 | 0.032 | 24.0 | 2.0 | 2.3 |
| ENSTGUG00000013361 | ATP6V1A | 0.179 | 0.032 | 24.0 | 21.0 | 2.3 |
| ENSTGUG00000005966 | SLC46A1 | 0.179 | 0.032 | 24.0 | 8.0 | 2.3 |
| ENSTGUG00000014964 | SLC5A6 | 0.179 | 0.032 | 24.0 | 4.0 | 2.3 |
| ENSTGUG00000001587 | KIAA1632 | 0.179 | 0.032 | 24.0 | 14.0 | 2.3 |
| ENSTGUG00000013211 | POLE2 | 0.179 | 0.032 | 24.0 | 14.0 | 2.3 |
| ENSTGUG00000005317 | SHISA5 | 0.179 | 0.032 | 25.3 | 12.7 | 2.3 |
| ENSTGUG00000011673 | RAB19 | 0.179 | 0.032 | 24.0 | 10.0 | 2.3 |
| ENSTGUG00000013590 | RNF160 | 0.179 | 0.032 | 26.0 | 18.0 | 2.2 |
| ENSTGUG00000005428 | ELMO3 | 0.179 | 0.032 | 24.0 | 2.0 | 2.2 |
| ENSTGUG00000000124 | LARS | 0.179 | 0.032 | 24.0 | 0.0 | 2.2 |
| ENSTGUG00000002834 | PPWD1 | 0.179 | 0.032 | 25.3 | 20.7 | 2.2 |
| ENSTGUG00000011062 | TMEM181 | 0.179 | 0.032 | 24.0 | 22.0 | 2.2 |
| ENSTGUG00000006427 | PHAX | 0.179 | 0.032 | 24.0 | 18.0 | 2.2 |
| ENSTGUG00000001083 | SEPN1 | 0.179 | 0.032 | 26.0 | 2.0 | 2.2 |
| ENSTGUG00000011303 | NCKAP1 | 0.179 | 0.032 | 24.0 | 16.0 | 2.2 |
| ENSTGUG00000004586 | MTMR1 | 0.179 | 0.032 | 24.0 | 14.0 | 2.2 |
| ENSTGUG00000008121 | LFNG | 0.179 | 0.032 | 24.0 | 8.0 | 2.2 |
| ENSTGUG00000015463 |  | 0.179 | 0.032 | 24.0 | 22.0 | 2.2 |
| ENSTGUG00000011684 | EPB41L2 | 0.179 | 0.032 | 24.0 | 13.0 | 2.2 |
| ENSTGUG00000002895 | KCTD3 | 0.179 | 0.032 | 24.0 | 13.0 | 2.1 |
| ENSTGUG00000001144 | C1orf130 | 0.179 | 0.032 | 24.0 | 18.0 | 2.1 |
| ENSTGUG00000009790 | EIF4ENIF1 | 0.179 | 0.032 | 24.0 | 0.0 | 2.1 |
| ENSTGUG00000002287 | CLGN | 0.179 | 0.032 | 24.0 | 13.0 | 2.1 |
| ENSTGUG00000002690 | TAF1A | 0.179 | 0.032 | 24.0 | 17.0 | 2.1 |
| ENSTGUG00000017418 | DAP3 | 0.179 | 0.032 | 24.0 | 6.0 | 2.1 |
| ENSTGUG00000016261 |  | 0.179 | 0.032 | 24.0 | 1.0 | 2.1 |
| ENSTGUG00000011317 | DHX36 | 0.179 | 0.032 | 24.0 | 14.0 | 2.1 |
| ENSTGUG00000012020 | REV3L | 0.179 | 0.032 | 28.0 | 2.0 | 2.1 |
| ENSTGUG00000005629 | SLC44A3 | 0.179 | 0.032 | 24.0 | 13.0 | 2.1 |
| ENSTGUG00000007657 | RPS6KB1 | 0.179 | 0.032 | 24.0 | 1.0 | 2.0 |
| ENSTGUG00000013067 | ROCK2 | 0.179 | 0.032 | 24.0 | 12.0 | 2.0 |
| ENSTGUG00000015811 | USP36 | 0.179 | 0.032 | 24.0 | 0.0 | 2.0 |
| ENSTGUG00000007420 | MERTK | 0.179 | 0.032 | 24.0 | 8.0 | 2.0 |
| ENSTGUG00000014099 |  | 0.179 | 0.032 | 24.0 | 0.0 | 2.0 |
| ENSTGUG00000008822 | DASRAA | 0.179 | 0.032 | 24.0 | 8.0 | 2.0 |
| ENSTGUG00000016361 |  | 0.179 | 0.032 | 24.0 | 1.0 | 2.0 |
| ENSTGUG00000007040 | ARMC8 | 0.179 | 0.032 | 24.0 | 9.0 | 2.0 |
| ENSTGUG00000005419 | TMEM168 | 0.179 | 0.032 | 25.3 | 20.7 | 2.0 |
| ENSTGUG00000006204 | C17orf63 | 0.179 | 0.032 | 28.0 | 2.0 | 2.0 |
| ENSTGUG00000007523 | FOXA2 | 0.179 | 0.032 | 24.0 | 2.0 | 2.0 |
| ENSTGUG00000005558 | PTBP2 | 0.179 | 0.032 | 24.0 | 13.0 | 2.0 |
| ENSTGUG00000011402 |  | 0.179 | 0.032 | 26.0 | 15.0 | 2.0 |
| ENSTGUG00000009946 | ZC3H7B | 0.179 | 0.032 | 24.0 | 2.0 | 2.0 |
| ENSTGUG00000015587 |  | 0.179 | 0.032 | 26.0 | 5.0 | 2.0 |
| ENSTGUG00000008360 |  | 0.179 | 0.032 | 24.0 | 22.0 | 2.0 |
| ENSTGUG00000007540 | CLTC | 0.179 | 0.032 | 25.3 | 9.3 | 2.0 |
| ENSTGUG00000009971 | SAPS3 | 0.179 | 0.032 | 24.0 | 20.0 | 2.0 |
| ENSTGUG00000001557 | DNALI1 | 0.179 | 0.032 | 24.0 | 21.0 | 2.0 |
| ENSTGUG00000008958 |  | 0.179 | 0.032 | 24.0 | 14.0 | 1.9 |
| ENSTGUG00000005893 | NT5DC2 | 0.179 | 0.032 | 24.0 | 13.0 | 1.9 |
| ENSTGUG00000005455 | FADD | 0.179 | 0.032 | 24.0 | 2.0 | 1.9 |
| ENSTGUG00000008923 | LTBP1 | 0.179 | 0.032 | 24.0 | 6.0 | 1.9 |
| ENSTGUG00000010414 | CSNK1E | 0.179 | 0.032 | 24.0 | 10.0 | 1.9 |
| ENSTGUG00000007278 | ATPAF2 | 0.179 | 0.032 | 24.0 | 1.0 | 1.9 |
| ENSTGUG00000008887 | QARS | 0.179 | 0.032 | 24.0 | 0.0 | 1.9 |
| ENSTGUG00000001119 | CNOT6 | 0.179 | 0.032 | 24.0 | 14.0 | 1.9 |
| ENSTGUG00000004121 | C3orf31 | 0.179 | 0.032 | 24.0 | 1.0 | 1.9 |
| ENSTGUG00000001202 | ARL8A | 0.179 | 0.032 | 24.0 | 12.0 | 1.9 |
| ENSTGUG00000002339 | TMEM141 | 0.179 | 0.032 | 24.0 | 6.0 | 1.9 |
| ENSTGUG00000003956 | ADCY9 | 0.179 | 0.032 | 24.0 | 0.0 | 1.9 |
| ENSTGUG00000013199 | KLHDC2 | 0.179 | 0.032 | 24.0 | 8.0 | 1.9 |
| ENSTGUG00000014372 |  | 0.179 | 0.032 | 24.0 | 1.0 | 1.9 |
| ENSTGUG00000008198 | FANCI | 0.179 | 0.032 | 28.0 | 16.0 | 1.9 |
| ENSTGUG00000006891 | TLR3 | 0.179 | 0.032 | 26.0 | 18.0 | 1.9 |
| ENSTGUG00000011368 |  | 0.179 | 0.032 | 26.0 | 18.0 | 1.9 |
| ENSTGUG00000013486 | N6AMT1 | 0.179 | 0.032 | 25.3 | 20.7 | 1.9 |
| ENSTGUG00000008574 | C2orf56 | 0.179 | 0.032 | 24.0 | 14.0 | 1.9 |
| ENSTGUG00000010291 | PRDM11 | 0.179 | 0.032 | 28.0 | 16.0 | 1.9 |
| ENSTGUG00000009760 | CD151 | 0.179 | 0.032 | 24.0 | 2.0 | 1.9 |
| ENSTGUG00000010227 |  | 0.179 | 0.032 | 24.0 | 22.0 | 1.9 |
| ENSTGUG00000006373 | VPS37C | 0.179 | 0.032 | 24.0 | 10.0 | 1.9 |
| ENSTGUG00000010957 | GPR126 | 0.179 | 0.032 | 24.0 | 10.0 | 1.9 |
| ENSTGUG00000011386 | EIF2A | 0.179 | 0.032 | 24.0 | 0.0 | 1.9 |
| ENSTGUG00000016930 | INTS3 | 0.179 | 0.032 | 24.0 | 2.0 | 1.9 |
| ENSTGUG00000016932 | KIAA0090 | 0.179 | 0.032 | 24.0 | 8.0 | 1.8 |
| ENSTGUG00000015450 |  | 0.179 | 0.032 | 24.0 | 12.0 | 1.8 |
| ENSTGUG00000014676 |  | 0.179 | 0.032 | 24.0 | 22.0 | 1.8 |
| ENSTGUG00000009739 | PHF10 | 0.179 | 0.032 | 26.0 | 18.0 | 1.8 |
| ENSTGUG00000009624 | C10orf65 | 0.179 | 0.032 | 24.0 | 2.0 | 1.8 |
| ENSTGUG00000012104 | RNASEH2B | 0.179 | 0.032 | 24.0 | 17.0 | 1.8 |
| ENSTGUG00000018128 |  | 0.179 | 0.032 | 24.0 | 4.0 | 1.8 |
| ENSTGUG00000002137 | FYB | 0.179 | 0.032 | 24.0 | 5.0 | 1.8 |
| ENSTGUG00000003508 | LAMP2 | 0.179 | 0.032 | 24.0 | 0.0 | 1.8 |
| ENSTGUG00000007829 | BCAS3 | 0.179 | 0.032 | 24.0 | 21.0 | 1.8 |
| ENSTGUG00000012748 |  | 0.179 | 0.032 | 24.0 | 5.0 | 1.8 |
| ENSTGUG00000015167 |  | 0.179 | 0.032 | 24.0 | 8.0 | 1.8 |
| ENSTGUG00000002722 | MARK1 | 0.179 | 0.032 | 24.0 | 12.0 | 1.8 |
| ENSTGUG00000013193 | ITSN2 | 0.179 | 0.032 | 24.0 | 2.0 | 1.8 |
| ENSTGUG00000005718 |  | 0.179 | 0.032 | 24.0 | 10.0 | 1.8 |
| ENSTGUG00000001337 | RNF19B | 0.179 | 0.032 | 24.0 | 0.0 | 1.8 |
| ENSTGUG00000012262 | ASCC3 | 0.179 | 0.032 | 25.3 | 20.7 | 1.8 |
| ENSTGUG00000010462 | LOC26010 | 0.179 | 0.032 | 24.0 | 5.0 | 1.7 |
| ENSTGUG00000004903 | BAT2L | 0.179 | 0.032 | 24.0 | 2.0 | 1.7 |
| ENSTGUG00000003843 | C2orf34 | 0.179 | 0.032 | 24.0 | 21.0 | 1.7 |
| ENSTGUG00000007134 | FGL1 | 0.179 | 0.032 | 24.0 | 9.0 | 1.7 |
| ENSTGUG00000004865 | MALT1-1 | 0.179 | 0.032 | 24.0 | 12.0 | 1.7 |
| ENSTGUG00000012064 | ANKRD46 | 0.179 | 0.032 | 24.0 | 20.0 | 1.7 |
| ENSTGUG00000005304 | ASL | 0.179 | 0.032 | 24.0 | 1.0 | 1.7 |
| ENSTGUG00000000094 |  | 0.179 | 0.032 | 24.0 | 1.0 | 1.7 |
| ENSTGUG00000006464 | SEMA3F | 0.179 | 0.032 | 24.0 | 4.0 | 1.7 |
| ENSTGUG00000010335 | VPREB3 | 0.179 | 0.032 | 28.0 | 0.0 | 1.7 |
| ENSTGUG00000003543 | SERINC5 | 0.179 | 0.032 | 24.0 | 17.0 | 1.7 |
| ENSTGUG00000001449 | RRAGC | 0.179 | 0.032 | 24.0 | 14.0 | 1.7 |
| ENSTGUG00000013549 | C21orf91 | 0.179 | 0.032 | 25.3 | 20.7 | 1.7 |
| ENSTGUG00000002826 | MSN | 0.179 | 0.032 | 24.0 | 5.0 | 1.7 |
| ENSTGUG00000013296 | GATA4 | 0.179 | 0.032 | 25.3 | 8.0 | 1.7 |
| ENSTGUG00000002897 | MMGT1 | 0.179 | 0.032 | 24.0 | 17.0 | 1.7 |
| ENSTGUG00000001032 | DHDDS | 0.179 | 0.032 | 24.0 | 2.0 | 1.7 |
| ENSTGUG00000010210 |  | 0.179 | 0.032 | 24.0 | 2.0 | 1.7 |
| ENSTGUG00000009547 | FGFR1OP | 0.179 | 0.032 | 28.0 | 16.0 | 1.7 |
| ENSTGUG00000017321 | MGST3 | 0.179 | 0.032 | 24.0 | 6.0 | 1.7 |
| ENSTGUG00000003674 | PAPD4 | 0.179 | 0.032 | 24.0 | 20.0 | 1.7 |
| ENSTGUG00000012957 | SGPP1 | 0.179 | 0.032 | 24.0 | 10.0 | 1.7 |
| ENSTGUG00000017550 | DTWD2 | 0.179 | 0.032 | 24.0 | 20.0 | 1.7 |
| ENSTGUG00000006719 | IGF2BP2 | 0.179 | 0.032 | 24.0 | 5.0 | 1.7 |
| ENSTGUG00000016412 |  | 0.179 | 0.032 | 25.3 | 12.7 | 1.6 |
| ENSTGUG00000001471 | ANKIB1 | 0.179 | 0.032 | 24.0 | 2.0 | 1.6 |
| ENSTGUG00000006202 | CTNNBL1 | 0.179 | 0.032 | 24.0 | 0.0 | 1.6 |
| ENSTGUG00000008752 |  | 0.179 | 0.032 | 28.0 | 16.0 | 1.6 |
| ENSTGUG00000010680 | PHF21A | 0.179 | 0.032 | 26.0 | 2.0 | 1.6 |
| ENSTGUG00000000671 | HAVCR2 | 0.179 | 0.032 | 24.0 | 9.0 | 1.6 |
| ENSTGUG00000010656 | CSF2RB | 0.179 | 0.032 | 24.0 | 8.0 | 1.6 |
| ENSTGUG00000001858 | C7orf36 | 0.179 | 0.032 | 24.0 | 18.0 | 1.6 |
| ENSTGUG00000008146 | STX16 | 0.179 | 0.032 | 24.0 | 12.0 | 1.6 |
| ENSTGUG00000007326 | MAPKAP1 | 0.179 | 0.032 | 26.0 | 5.0 | 1.6 |
| ENSTGUG00000014941 | MAPRE3 | 0.179 | 0.032 | 24.0 | 4.0 | 1.6 |
| ENSTGUG00000004552 | PRPF3 | 0.179 | 0.032 | 24.0 | 2.0 | 1.6 |
| ENSTGUG00000008601 | BRPF1 | 0.179 | 0.032 | 24.0 | 4.0 | 1.6 |
| ENSTGUG00000001369 | SLC22A5 | 0.179 | 0.032 | 24.0 | 20.0 | 1.6 |
| ENSTGUG00000000944 | FGD2 | 0.179 | 0.032 | 24.0 | 9.0 | 1.6 |
| ENSTGUG00000006900 |  | 0.179 | 0.032 | 24.0 | 9.0 | 1.6 |
| ENSTGUG00000003155 | C9orf64 | 0.179 | 0.032 | 24.0 | 10.0 | 1.6 |
| ENSTGUG00000012246 | C14orf179 | 0.179 | 0.032 | 24.0 | 22.0 | 1.6 |
| ENSTGUG00000010229 | SMCR7L | 0.179 | 0.032 | 24.0 | 10.0 | 1.6 |
| ENSTGUG00000010207 | GPR177 | 0.179 | 0.032 | 24.0 | 8.0 | 1.6 |
| ENSTGUG00000011565 | TMEM70 | 0.179 | 0.032 | 24.0 | 20.0 | 1.6 |
| ENSTGUG00000000793 | ANXA1 | 0.179 | 0.032 | 24.0 | 9.0 | 1.5 |
| ENSTGUG00000001000 | PIAS4 | 0.179 | 0.032 | 24.0 | 2.0 | 1.5 |
| ENSTGUG00000013758 | GRN | 0.179 | 0.032 | 24.0 | 2.0 | 1.5 |
| ENSTGUG00000002089 | DCAKD | 0.179 | 0.032 | 24.0 | 0.0 | 1.5 |
| ENSTGUG00000011770 | AQR | 0.179 | 0.032 | 24.0 | 14.0 | 1.5 |
| ENSTGUG00000010635 | CHRM4 | 0.179 | 0.032 | 24.0 | 6.0 | 1.5 |
| ENSTGUG00000003684 | CMTM8 | 0.179 | 0.032 | 26.0 | 15.0 | 1.5 |
| ENSTGUG00000009485 | HPS1 | 0.179 | 0.032 | 24.0 | 5.0 | 1.5 |
| ENSTGUG00000001241 | NSUN6 | 0.179 | 0.032 | 24.0 | 17.0 | 1.5 |
| ENSTGUG00000013088 | CGRRF1 | 0.179 | 0.032 | 24.0 | 12.0 | 1.5 |
| ENSTGUG00000003924 | RERE | 0.179 | 0.032 | 24.0 | 2.0 | 1.5 |
| ENSTGUG00000009056 | TBC1D1 | 0.179 | 0.032 | 24.0 | 13.0 | 1.5 |
| ENSTGUG00000003769 | CSAD | 0.179 | 0.032 | 24.0 | 21.0 | 1.5 |
| ENSTGUG00000004960 | DYRK1A | 0.179 | 0.032 | 24.0 | 2.0 | 1.5 |
| ENSTGUG00000012623 | BTBD7 | 0.179 | 0.032 | 24.0 | 14.0 | 1.5 |
| ENSTGUG00000013015 | ITGB1BP1 | 0.179 | 0.032 | 24.0 | 21.0 | 1.5 |
| ENSTGUG00000001853 | TLN1 | 0.179 | 0.032 | 24.0 | 2.0 | 1.5 |
| ENSTGUG00000009116 | RBM45 | 0.179 | 0.032 | 24.0 | 14.0 | 1.5 |
| ENSTGUG00000011210 | NT5DC3 | 0.179 | 0.032 | 24.0 | 14.0 | 1.5 |
| ENSTGUG00000002999 | MBNL3 | 0.179 | 0.032 | 24.0 | 16.0 | 1.5 |
| ENSTGUG00000015825 |  | 0.179 | 0.032 | 24.0 | 20.0 | 1.4 |
| ENSTGUG00000001650 | ACN9 | 0.179 | 0.032 | 24.0 | 14.0 | 1.4 |
| ENSTGUG00000003112 | METTL2A | 0.179 | 0.032 | 24.0 | 13.0 | 1.4 |
| ENSTGUG00000008159 |  | 0.179 | 0.032 | 24.0 | 9.0 | 1.4 |
| ENSTGUG00000015597 |  | 0.179 | 0.032 | 24.0 | 1.0 | 1.4 |
| ENSTGUG00000007604 | FBXL15 | 0.179 | 0.032 | 24.0 | 10.0 | 1.4 |
| ENSTGUG00000005302 | DDX55 | 0.179 | 0.032 | 24.0 | 18.0 | 1.4 |
| ENSTGUG00000011056 | LYN | 0.179 | 0.032 | 24.0 | 9.0 | 1.4 |
| ENSTGUG00000015332 |  | 0.179 | 0.032 | 24.0 | 17.0 | 1.4 |
| ENSTGUG00000006433 | GPR34 | 0.179 | 0.032 | 24.0 | 5.0 | 1.4 |
| ENSTGUG00000000637 | CYFIP2 | 0.179 | 0.032 | 24.0 | 12.0 | 1.4 |
| ENSTGUG00000007130 | MYEF2 | 0.179 | 0.032 | 25.3 | 16.7 | 1.4 |
| ENSTGUG00000009397 | ZCCHC4 | 0.179 | 0.032 | 24.0 | 14.0 | 1.4 |
| ENSTGUG00000013050 | MUDENG | 0.179 | 0.032 | 24.0 | 22.0 | 1.4 |
| ENSTGUG00000001049 | MAML1 | 0.179 | 0.032 | 24.0 | 12.0 | 1.4 |
| ENSTGUG00000006507 | CYBASC3 | 0.179 | 0.032 | 24.0 | 4.0 | 1.4 |
| ENSTGUG00000003808 | GATSL1 | 0.179 | 0.032 | 24.0 | 9.0 | 1.4 |
| ENSTGUG00000010542 | TBC1D10A | 0.179 | 0.032 | 24.0 | 2.0 | 1.4 |
| ENSTGUG00000010967 | DOCK9 | 0.179 | 0.032 | 24.0 | 13.0 | 1.4 |
| ENSTGUG00000013184 | SOS2 | 0.179 | 0.032 | 24.0 | 22.0 | 1.4 |
| ENSTGUG00000004772 | METT5D1 | 0.179 | 0.032 | 24.0 | 18.0 | 1.4 |
| ENSTGUG00000013637 | DCBLD2 | 0.179 | 0.032 | 24.0 | 13.0 | 1.3 |
| ENSTGUG00000010065 | PDE4B | 0.179 | 0.032 | 24.0 | 2.0 | 1.3 |
| ENSTGUG00000014206 |  | 0.179 | 0.032 | 24.0 | 21.0 | 1.3 |
| ENSTGUG00000004812 | FZD10 | 0.179 | 0.032 | 24.0 | 0.0 | 1.3 |
| ENSTGUG00000008961 | WDR19 | 0.179 | 0.032 | 26.0 | 15.0 | 1.3 |
| ENSTGUG00000008733 | MEF2A | 0.179 | 0.032 | 24.0 | 22.0 | 1.3 |
| ENSTGUG00000005404 |  | 0.179 | 0.032 | 24.0 | 10.0 | 1.3 |
| ENSTGUG00000006596 | SAMD8 | 0.179 | 0.032 | 24.0 | 1.0 | 1.3 |
| ENSTGUG00000006501 | SLC6A4 | 0.179 | 0.032 | 25.3 | 8.0 | 1.3 |
| ENSTGUG00000017471 | PLEKHA6 | 0.179 | 0.032 | 24.0 | 2.0 | 1.3 |
| ENSTGUG00000010843 | TMEM194B | 0.179 | 0.032 | 24.0 | 9.0 | 1.3 |
| ENSTGUG00000010094 | FAM178A | 0.179 | 0.032 | 24.0 | 17.0 | 1.3 |
| ENSTGUG00000017529 |  | 0.179 | 0.032 | 24.0 | 1.0 | 1.3 |
| ENSTGUG00000006273 |  | 0.179 | 0.032 | 24.0 | 13.0 | 1.3 |
| ENSTGUG00000005434 | AP3M2 | 0.179 | 0.032 | 24.0 | 12.0 | 1.3 |
| ENSTGUG00000013376 | NDUFB4-1 | 0.179 | 0.032 | 24.0 | 12.0 | 1.3 |
| ENSTGUG00000013660 | DDX20 | 0.179 | 0.032 | 25.3 | 16.7 | 1.3 |
| ENSTGUG00000001640 | MOBKL1A | 0.179 | 0.032 | 24.0 | 10.0 | 1.3 |
| ENSTGUG00000013947 |  | 0.179 | 0.032 | 28.0 | 2.0 | 1.3 |
| ENSTGUG00000006062 | DES | 0.179 | 0.032 | 26.0 | 5.0 | 1.3 |
| ENSTGUG00000006888 | TMOD2 | 0.179 | 0.032 | 26.0 | 15.0 | 1.3 |
| ENSTGUG00000009738 | C6orf120 | 0.179 | 0.032 | 24.0 | 20.0 | 1.3 |
| ENSTGUG00000006446 | CLDN2 | 0.179 | 0.032 | 24.0 | 5.0 | 1.3 |
| ENSTGUG00000003304 | KIF1B | 0.179 | 0.032 | 24.0 | 8.0 | 1.3 |
| ENSTGUG00000007056 | PPP3CB | 0.179 | 0.032 | 24.0 | 20.0 | 1.3 |
| ENSTGUG00000009829 | PISD | 0.179 | 0.032 | 25.3 | 16.7 | 1.3 |
| ENSTGUG00000005644 | PGM5 | 0.179 | 0.032 | 24.0 | 10.0 | 1.3 |
| ENSTGUG00000006488 | PLA2R1 | 0.179 | 0.032 | 26.0 | 2.0 | 1.2 |
| ENSTGUG00000003403 | CEP78 | 0.179 | 0.032 | 24.0 | 18.0 | 1.2 |
| ENSTGUG00000013624 | SENP7 | 0.179 | 0.032 | 26.0 | 18.0 | 1.2 |
| ENSTGUG00000013106 | FAM49A | 0.179 | 0.032 | 24.0 | 10.0 | 1.2 |
| ENSTGUG00000001492 | TDRD7 | 0.179 | 0.032 | 24.0 | 21.0 | 1.2 |
| ENSTGUG00000006000 | OLFM1 | 0.179 | 0.032 | 24.0 | 21.0 | 1.2 |
| ENSTGUG00000016406 |  | 0.179 | 0.032 | 24.0 | 2.0 | 1.2 |
| ENSTGUG00000010122 |  | 0.179 | 0.032 | 26.0 | 2.0 | 1.2 |
| ENSTGUG00000005874 | WDR53 | 0.179 | 0.032 | 24.0 | 9.0 | 1.2 |
| ENSTGUG00000003588 | NOTUM | 0.179 | 0.032 | 24.0 | 9.0 | 1.2 |
| ENSTGUG00000007277 | VPS53 | 0.179 | 0.032 | 24.0 | 21.0 | 1.2 |
| ENSTGUG00000015043 |  | 0.179 | 0.032 | 26.0 | 15.0 | 1.2 |
| ENSTGUG00000005735 | SAP130 | 0.179 | 0.032 | 24.0 | 0.0 | 1.2 |
| ENSTGUG00000008193 | OFD1 | 0.179 | 0.032 | 24.0 | 13.0 | 1.2 |
| ENSTGUG00000003758 | FBLN2 | 0.179 | 0.032 | 24.0 | 1.0 | 1.2 |
| ENSTGUG00000001234 | KLHL12 | 0.179 | 0.032 | 25.3 | 8.0 | 1.2 |
| ENSTGUG00000000612 | CDC34 | 0.179 | 0.032 | 24.0 | 12.0 | 1.2 |
| ENSTGUG00000001493 | TRAF3IP3 | 0.179 | 0.032 | 24.0 | 8.0 | 1.2 |
| ENSTGUG00000003535 |  | 0.179 | 0.032 | 24.0 | 6.0 | 1.2 |
| ENSTGUG00000004519 | PSIP1 | 0.179 | 0.032 | 24.0 | 20.0 | 1.2 |
| ENSTGUG00000003178 |  | 0.179 | 0.032 | 24.0 | 14.0 | 1.2 |
| ENSTGUG00000009028 | SMTNL2 | 0.179 | 0.032 | 24.0 | 9.0 | 1.2 |
| ENSTGUG00000011990 | LHFP | 0.179 | 0.032 | 24.0 | 9.0 | 1.2 |
| ENSTGUG00000006686 | C16orf70 | 0.179 | 0.032 | 24.0 | 20.0 | 1.2 |
| ENSTGUG00000006222 | PATL1 | 0.179 | 0.032 | 28.0 | 2.0 | 1.2 |
| ENSTGUG00000012703 | CDKN1B | 0.179 | 0.032 | 24.0 | 0.0 | 1.2 |
| ENSTGUG00000010469 | RFC4 | 0.179 | 0.032 | 24.0 | 14.0 | 1.2 |
| ENSTGUG00000014336 |  | 0.179 | 0.032 | 26.0 | 18.0 | 1.2 |
| ENSTGUG00000011377 | SAP18 | 0.179 | 0.032 | 24.0 | 8.0 | 1.2 |
| ENSTGUG00000010941 | CITED2 | 0.179 | 0.032 | 24.0 | 0.0 | 1.2 |
| ENSTGUG00000008666 |  | 0.179 | 0.032 | 24.0 | 12.0 | 1.2 |
| ENSTGUG00000001355 | KIAA1522-2 | 0.179 | 0.032 | 24.0 | 2.0 | 1.2 |
| ENSTGUG00000008669 | C10orf4 | 0.179 | 0.032 | 24.0 | 16.0 | 1.2 |
| ENSTGUG00000006201 | RPAP3 | 0.179 | 0.032 | 24.0 | 13.0 | 1.1 |
| ENSTGUG00000002195 | TRMT6 | 0.179 | 0.032 | 24.0 | 16.0 | 1.1 |
| ENSTGUG00000012686 | AK7 | 0.179 | 0.032 | 28.0 | 16.0 | 1.1 |
| ENSTGUG00000003959 | OSBPL11 | 0.179 | 0.032 | 24.0 | 9.0 | 1.1 |
| ENSTGUG00000011651 | PAG1 | 0.179 | 0.032 | 24.0 | 0.0 | 1.1 |
| ENSTGUG00000004735 | FMR1 | 0.179 | 0.032 | 24.0 | 12.0 | 1.1 |
| ENSTGUG00000007997 | MYO19 | 0.179 | 0.032 | 24.0 | 13.0 | 1.1 |
| ENSTGUG00000010998 | MYNN | 0.179 | 0.032 | 24.0 | 20.0 | 1.1 |
| ENSTGUG00000010670 | MAP3K7IP2 | 0.179 | 0.032 | 24.0 | 12.0 | 1.1 |
| ENSTGUG00000013579 | C21orf66 | 0.179 | 0.032 | 25.3 | 20.7 | 1.1 |
| ENSTGUG00000010775 | VPS18 | 0.179 | 0.032 | 24.0 | 0.0 | 1.1 |
| ENSTGUG00000008317 | CH25H | 0.179 | 0.032 | 24.0 | 2.0 | 1.1 |
| ENSTGUG00000003445 | MTA3 | 0.179 | 0.032 | 24.0 | 4.0 | 1.1 |
| ENSTGUG00000011113 | ARID1B | 0.179 | 0.032 | 24.0 | 22.0 | 1.1 |
| ENSTGUG00000004559 | HSPB1 | 0.179 | 0.032 | 24.0 | 6.0 | 1.1 |
| ENSTGUG00000014318 |  | 0.179 | 0.032 | 24.0 | 13.0 | 1.1 |
| ENSTGUG00000015243 | CCT7 | 0.179 | 0.032 | 24.0 | 2.0 | 1.1 |
| ENSTGUG00000008549 | CPNE9 | 0.179 | 0.032 | 24.0 | 9.0 | 1.1 |
| ENSTGUG00000016036 |  | 0.179 | 0.032 | 24.0 | 0.0 | 1.1 |
| ENSTGUG00000003349 | THRB | 0.179 | 0.032 | 26.0 | 2.0 | 1.1 |
| ENSTGUG00000017399 | TUFT1 | 0.179 | 0.032 | 24.0 | 0.0 | 1.1 |
| ENSTGUG00000006050 | EGFL7 | 0.179 | 0.032 | 28.0 | 2.0 | 1.1 |
| ENSTGUG00000002917 | HOXA7 | 0.179 | 0.032 | 24.0 | 9.0 | 1.1 |
| ENSTGUG00000003988 | TUBGCP6-1 | 0.179 | 0.032 | 24.0 | 17.0 | 1.1 |
| ENSTGUG00000014680 | HTRA2 | 0.179 | 0.032 | 24.0 | 6.0 | 1.1 |
| ENSTGUG00000016128 |  | 0.179 | 0.032 | 24.0 | 0.0 | 1.1 |
| ENSTGUG00000010736 | PIK3CA | 0.179 | 0.032 | 24.0 | 22.0 | 1.1 |
| ENSTGUG00000006091 | SFRS2IP | 0.179 | 0.032 | 24.0 | 14.0 | 1.1 |
| ENSTGUG00000007069 | UNG | 0.179 | 0.032 | 28.0 | 0.0 | 1.1 |
| ENSTGUG00000009017 | RBM19 | 0.179 | 0.032 | 24.0 | 0.0 | 1.1 |
| ENSTGUG00000002631 | SOCS5 | 0.179 | 0.032 | 26.0 | 5.0 | 1.1 |
| ENSTGUG00000008649 | CAMK1 | 0.179 | 0.032 | 24.0 | 8.0 | 1.1 |
| ENSTGUG00000018201 |  | 0.179 | 0.032 | 24.0 | 10.0 | 1.1 |
| ENSTGUG00000006002 | FTSJD1 | 0.179 | 0.032 | 24.0 | 20.0 | 1.1 |
| ENSTGUG00000002885 | SLC39A11 | 0.179 | 0.032 | 24.0 | 6.0 | 1.1 |
| ENSTGUG00000010080 | C1orf124 | 0.179 | 0.032 | 24.0 | 0.0 | 1.0 |
| ENSTGUG00000015904 |  | 0.179 | 0.032 | 24.0 | 0.0 | 1.0 |
| ENSTGUG00000012315 | FHL5 | 0.179 | 0.032 | 24.0 | 8.0 | 1.0 |
| ENSTGUG00000014691 | SLC22A4 | 0.179 | 0.032 | 24.0 | 22.0 | 1.0 |
| ENSTGUG00000003006 | VASH2 | 0.179 | 0.032 | 25.3 | 8.0 | 1.0 |
| ENSTGUG00000005043 | JAK2 | 0.179 | 0.032 | 24.0 | 8.0 | 1.0 |
| ENSTGUG00000001292 | PTPLA | 0.179 | 0.032 | 24.0 | 13.0 | 1.0 |
| ENSTGUG00000010280 | B3GALNT2 | 0.179 | 0.032 | 24.0 | 13.0 | 1.0 |
| ENSTGUG00000012208 | ANGPT1 | 0.179 | 0.032 | 24.0 | 5.0 | 1.0 |
| ENSTGUG00000002109 | HP1BP3 | 0.179 | 0.032 | 24.0 | 20.0 | 1.0 |
| ENSTGUG00000009103 | PARP16 | 0.179 | 0.032 | 24.0 | 12.0 | 1.0 |
| ENSTGUG00000015146 |  | 0.179 | 0.032 | 25.3 | 16.7 | 1.0 |
| ENSTGUG00000000421 | ARHGEF12 | 0.179 | 0.032 | 24.0 | 4.0 | 1.0 |
| ENSTGUG00000005446 | CLIP4 | 0.179 | 0.032 | 24.0 | 16.0 | 1.0 |
| ENSTGUG00000008628 | PPM1F | 0.179 | 0.032 | 24.0 | 21.0 | 1.0 |
| ENSTGUG00000002742 | FRMPD2-1 | 0.179 | 0.032 | 24.0 | 6.0 | 1.0 |
| ENSTGUG00000002939 | TTYH2 | 0.179 | 0.032 | 25.3 | 8.0 | 1.0 |
| ENSTGUG00000000617 | HCN2-2 | 0.179 | 0.032 | 24.0 | 4.0 | 1.0 |
| ENSTGUG00000001421 | IKBKAP | 0.179 | 0.032 | 24.0 | 2.0 | 1.0 |
| ENSTGUG00000013985 |  | 0.179 | 0.032 | 24.0 | 8.0 | 1.0 |
| ENSTGUG00000006661 | DNAJC17 | 0.179 | 0.032 | 24.0 | 1.0 | 1.0 |
| ENSTGUG00000000750 | SLC37A2 | 0.179 | 0.032 | 24.0 | 8.0 | 1.0 |
| ENSTGUG00000002500 |  | 0.179 | 0.032 | 26.0 | 2.0 | 1.0 |
| ENSTGUG00000009934 | LRP5 | 0.179 | 0.032 | 24.0 | 0.0 | 0.9 |
| ENSTGUG00000016819 |  | 0.179 | 0.032 | 24.0 | 14.0 | 0.9 |
| ENSTGUG00000017406 | SEC16B | 0.179 | 0.032 | 24.0 | 5.0 | 0.9 |
| ENSTGUG00000018227 |  | 0.179 | 0.032 | 24.0 | 12.0 | 0.9 |
| ENSTGUG00000013125 | SLC2A3 | 0.179 | 0.032 | 26.0 | 2.0 | 0.9 |
| ENSTGUG00000006196 | RASGEF1A | 0.179 | 0.032 | 24.0 | 12.0 | 0.9 |
| ENSTGUG00000009853 | RAB28 | 0.179 | 0.032 | 25.3 | 16.7 | 0.9 |
| ENSTGUG00000006820 | DCP1A | 0.179 | 0.032 | 24.0 | 22.0 | 0.9 |
| ENSTGUG00000010157 | TRIM8 | 0.179 | 0.032 | 24.0 | 8.0 | 0.9 |
| ENSTGUG00000018183 |  | 0.179 | 0.032 | 24.0 | 14.0 | 0.9 |
| ENSTGUG00000009731 | NCK2 | 0.179 | 0.032 | 24.0 | 9.0 | 0.9 |
| ENSTGUG00000012150 | KIAA0317 | 0.179 | 0.032 | 24.0 | 1.0 | 0.9 |
| ENSTGUG00000010828 | FAM54A | 0.179 | 0.032 | 24.0 | 10.0 | 0.9 |
| ENSTGUG00000017447 | CCND3 | 0.179 | 0.032 | 24.0 | 8.0 | 0.9 |
| ENSTGUG00000015066 |  | 0.179 | 0.032 | 24.0 | 4.0 | 0.9 |
| ENSTGUG00000015045 |  | 0.179 | 0.032 | 24.0 | 12.0 | 0.9 |
| ENSTGUG00000006316 | LMO4 | 0.179 | 0.032 | 24.0 | 2.0 | 0.9 |
| ENSTGUG00000013718 |  | 0.179 | 0.032 | 24.0 | 2.0 | 0.9 |
| ENSTGUG00000000070 | LASS4 | 0.179 | 0.032 | 24.0 | 14.0 | 0.9 |
| ENSTGUG00000001784 |  | 0.179 | 0.032 | 28.0 | 0.0 | 0.9 |
| ENSTGUG00000016779 | RBM4B | 0.179 | 0.032 | 26.0 | 5.0 | 0.8 |
| ENSTGUG00000018403 |  | 0.179 | 0.032 | 24.0 | 1.0 | 0.8 |
| ENSTGUG00000015003 |  | 0.179 | 0.032 | 24.0 | 2.0 | 0.8 |
| ENSTGUG00000006142 | TNRC6A | 0.179 | 0.032 | 24.0 | 5.0 | 0.8 |
| ENSTGUG00000000696 | RBM33 | 0.179 | 0.032 | 24.0 | 5.0 | 0.8 |
| ENSTGUG00000008450 | TFDP2 | 0.179 | 0.032 | 24.0 | 12.0 | 0.8 |
| ENSTGUG00000002794 | LIN54 | 0.179 | 0.032 | 24.0 | 14.0 | 0.8 |
| ENSTGUG00000002506 |  | 0.179 | 0.032 | 24.0 | 9.0 | 0.8 |
| ENSTGUG00000014708 |  | 0.179 | 0.032 | 24.0 | 16.0 | 0.8 |
| ENSTGUG00000002376 | CNRIP1 | 0.179 | 0.032 | 24.0 | 5.0 | 0.8 |
| ENSTGUG00000015694 |  | 0.179 | 0.032 | 24.0 | 12.0 | 0.8 |
| ENSTGUG00000013423 | CCDC80 | 0.179 | 0.032 | 24.0 | 9.0 | 0.8 |
| ENSTGUG00000004755 | PTK7 | 0.179 | 0.032 | 26.0 | 2.0 | 0.8 |
| ENSTGUG00000003139 | RFTN1 | 0.179 | 0.032 | 24.0 | 8.0 | 0.8 |
| ENSTGUG00000015705 | B5FXE6_TAEGU | 0.179 | 0.032 | 24.0 | 12.0 | 0.8 |
| ENSTGUG00000013304 | CD4 | 0.179 | 0.032 | 24.0 | 12.0 | 0.8 |
| ENSTGUG00000000414 | ROR2 | 0.179 | 0.032 | 24.0 | 13.0 | 0.8 |
| ENSTGUG00000008526 | PDGFA | 0.179 | 0.032 | 24.0 | 6.0 | 0.8 |
| ENSTGUG00000002116 | LIFR | 0.179 | 0.032 | 28.0 | 2.0 | 0.8 |
| ENSTGUG00000004653 | RASSF1 | 0.179 | 0.032 | 24.0 | 8.0 | 0.8 |
| ENSTGUG00000000063 |  | 0.179 | 0.032 | 24.0 | 20.0 | 0.8 |
| ENSTGUG00000006016 | KIAA0649 | 0.179 | 0.032 | 24.0 | 14.0 | 0.8 |
| ENSTGUG00000014871 |  | 0.179 | 0.032 | 24.0 | 22.0 | 0.8 |
| ENSTGUG00000008116 |  | 0.179 | 0.032 | 24.0 | 8.0 | 0.8 |
| ENSTGUG00000006972 |  | 0.179 | 0.032 | 26.0 | 5.0 | 0.8 |
| ENSTGUG00000001667 | AQP3 | 0.179 | 0.032 | 25.3 | 12.7 | 0.8 |
| ENSTGUG00000011886 | C14orf126 | 0.179 | 0.032 | 24.0 | 20.0 | 0.8 |
| ENSTGUG00000009897 | PTPRM | 0.179 | 0.032 | 24.0 | 2.0 | 0.8 |
| ENSTGUG00000006690 | NCOR1 | 0.179 | 0.032 | 24.0 | 9.0 | 0.8 |
| ENSTGUG00000010679 |  | 0.179 | 0.032 | 24.0 | 12.0 | 0.8 |
| ENSTGUG00000002355 | PARP8 | 0.179 | 0.032 | 24.0 | 9.0 | 0.8 |
| ENSTGUG00000005593 | FAM123B-1 | 0.179 | 0.032 | 24.0 | 12.0 | 0.7 |
| ENSTGUG00000012828 | PANX1 | 0.179 | 0.032 | 28.0 | 0.0 | 0.7 |
| ENSTGUG00000008365 | CLCN4 | 0.179 | 0.032 | 24.0 | 21.0 | 0.7 |
| ENSTGUG00000009649 | LRCH3 | 0.179 | 0.032 | 26.0 | 5.0 | 0.7 |
| ENSTGUG00000003746 | H6PD | 0.179 | 0.032 | 24.0 | 2.0 | 0.7 |
| ENSTGUG00000002191 | CHGB | 0.179 | 0.032 | 28.0 | 2.0 | 0.7 |
| ENSTGUG00000002124 | GMDS | 0.179 | 0.032 | 24.0 | 9.0 | 0.7 |
| ENSTGUG00000017525 | LRIG2 | 0.179 | 0.032 | 24.0 | 1.0 | 0.7 |
| ENSTGUG00000017402 | ETV3 | 0.179 | 0.032 | 24.0 | 6.0 | 0.7 |
| ENSTGUG00000010362 | RBBP8 | 0.179 | 0.032 | 24.0 | 13.0 | 0.7 |
| ENSTGUG00000009011 | ZCCHC11 | 0.179 | 0.032 | 24.0 | 14.0 | 0.7 |
| ENSTGUG00000003582 | B5FXW3_TAEGU | 0.179 | 0.032 | 24.0 | 2.0 | 0.7 |
| ENSTGUG00000007658 | RNF216 | 0.179 | 0.032 | 24.0 | 6.0 | 0.7 |
| ENSTGUG00000004216 | CCDC46 | 0.179 | 0.032 | 24.0 | 10.0 | 0.7 |
| ENSTGUG00000016704 |  | 0.179 | 0.032 | 24.0 | 0.0 | 0.7 |
| ENSTGUG00000010558 | WHSC1 | 0.179 | 0.032 | 24.0 | 10.0 | 0.7 |
| ENSTGUG00000004122 | DUSP11 | 0.179 | 0.032 | 24.0 | 0.0 | 0.7 |
| ENSTGUG00000013440 | ST3GAL6 | 0.179 | 0.032 | 24.0 | 9.0 | 0.7 |
| ENSTGUG00000007185 | DEAF1 | 0.179 | 0.032 | 24.0 | 4.0 | 0.7 |
| ENSTGUG00000001293 | MACF1 | 0.179 | 0.032 | 24.0 | 2.0 | 0.7 |
| ENSTGUG00000005342 | C20orf111 | 0.179 | 0.032 | 24.0 | 13.0 | 0.7 |
| ENSTGUG00000002816 |  | 0.179 | 0.032 | 24.0 | 2.0 | 0.7 |
| ENSTGUG00000016439 |  | 0.179 | 0.032 | 24.0 | 8.0 | 0.7 |
| ENSTGUG00000007578 |  | 0.179 | 0.032 | 24.0 | 4.0 | 0.7 |
| ENSTGUG00000011215 | ACOT1 | 0.179 | 0.032 | 24.0 | 14.0 | 0.7 |
| ENSTGUG00000007328 |  | 0.179 | 0.032 | 24.0 | 8.0 | 0.7 |
| ENSTGUG00000007172 | PDGFRL | 0.179 | 0.032 | 24.0 | 10.0 | 0.7 |
| ENSTGUG00000016214 | IGSF9 | 0.179 | 0.032 | 24.0 | 4.0 | 0.7 |
| ENSTGUG00000000828 | SLC39A3 | 0.179 | 0.032 | 24.0 | 5.0 | 0.7 |
| ENSTGUG00000008394 | ATPAF1 | 0.179 | 0.032 | 24.0 | 6.0 | 0.7 |
| ENSTGUG00000007417 | JMJD2A | 0.179 | 0.032 | 24.0 | 0.0 | 0.7 |
| ENSTGUG00000006586 | OGFOD1 | 0.179 | 0.032 | 24.0 | 16.0 | 0.7 |
| ENSTGUG00000010513 |  | 0.179 | 0.032 | 24.0 | 4.0 | 0.7 |
| ENSTGUG00000009821 | SFI1 | 0.179 | 0.032 | 24.0 | 21.0 | 0.7 |
| ENSTGUG00000010433 | PLA2G6 | 0.179 | 0.032 | 24.0 | 2.0 | 0.7 |
| ENSTGUG00000002659 | PTRF | 0.179 | 0.032 | 24.0 | 2.0 | 0.7 |
| ENSTGUG00000014403 |  | 0.179 | 0.032 | 24.0 | 5.0 | 0.7 |
| ENSTGUG00000007342 | INPP5D | 0.179 | 0.032 | 24.0 | 9.0 | 0.7 |
| ENSTGUG00000006509 | FAM161A | 0.179 | 0.032 | 24.0 | 13.0 | 0.7 |
| ENSTGUG00000005814 | DPH1 | 0.179 | 0.032 | 24.0 | 2.0 | 0.7 |
| ENSTGUG00000015425 | C2orf7 | 0.179 | 0.032 | 24.0 | 2.0 | 0.7 |
| ENSTGUG00000008717 | RHOH | 0.179 | 0.032 | 24.0 | 9.0 | 0.7 |
| ENSTGUG00000015471 | B5G0X9_TAEGU | 0.179 | 0.032 | 24.0 | 12.0 | 0.7 |
| ENSTGUG00000013042 | RRM2 | 0.179 | 0.032 | 24.0 | 13.0 | 0.7 |
| ENSTGUG00000009677 |  | 0.179 | 0.032 | 24.0 | 21.0 | 0.7 |
| ENSTGUG00000016388 |  | 0.179 | 0.032 | 24.0 | 2.0 | 0.7 |
| ENSTGUG00000012082 | CACNB4 | 0.179 | 0.032 | 24.0 | 13.0 | 0.7 |
| ENSTGUG00000009920 | ZNF509 | 0.179 | 0.032 | 24.0 | 18.0 | 0.7 |
| ENSTGUG00000008053 | DUSP6 | 0.179 | 0.032 | 24.0 | 8.0 | 0.6 |
| ENSTGUG00000017268 | DNM3 | 0.179 | 0.032 | 24.0 | 4.0 | 0.6 |
| ENSTGUG00000004357 | CDH13 | 0.179 | 0.032 | 28.0 | 2.0 | 0.6 |
| ENSTGUG00000011265 | ARHGAP20 | 0.179 | 0.032 | 24.0 | 9.0 | 0.6 |
| ENSTGUG00000010169 | FAM83F | 0.179 | 0.032 | 24.0 | 21.0 | 0.6 |
| ENSTGUG00000015558 |  | 0.179 | 0.032 | 24.0 | 4.0 | 0.6 |
| ENSTGUG00000009966 | TBC1D8 | 0.179 | 0.032 | 24.0 | 6.0 | 0.6 |
| ENSTGUG00000012675 | KIAA1826 | 0.179 | 0.032 | 24.0 | 12.0 | 0.6 |
| ENSTGUG00000003970 |  | 0.179 | 0.032 | 24.0 | 8.0 | 0.6 |
| ENSTGUG00000013483 | ADAMTS1 | 0.179 | 0.032 | 24.0 | 8.0 | 0.6 |
| ENSTGUG00000002852 | GPATCH2 | 0.179 | 0.032 | 24.0 | 20.0 | 0.6 |
| ENSTGUG00000012948 | ANKRD42 | 0.179 | 0.032 | 24.0 | 1.0 | 0.6 |
| ENSTGUG00000007202 | C9orf126 | 0.179 | 0.032 | 24.0 | 12.0 | 0.6 |
| ENSTGUG00000009446 | EMP1 | 0.179 | 0.032 | 24.0 | 12.0 | 0.6 |
| ENSTGUG00000010512 | RFTN2 | 0.179 | 0.032 | 25.3 | 8.0 | 0.6 |
| ENSTGUG00000006396 | ANKRD13B | 0.179 | 0.032 | 24.0 | 5.0 | 0.6 |
| ENSTGUG00000009847 | MITF | 0.179 | 0.032 | 24.0 | 8.0 | 0.6 |
| ENSTGUG00000004364 | P4HA1 | 0.179 | 0.032 | 24.0 | 9.0 | 0.6 |
| ENSTGUG00000013141 |  | 0.179 | 0.032 | 24.0 | 9.0 | 0.6 |
| ENSTGUG00000003371 | THRB | 0.179 | 0.032 | 24.0 | 4.0 | 0.6 |
| ENSTGUG00000008127 | WEE1 | 0.179 | 0.032 | 24.0 | 13.0 | 0.6 |
| ENSTGUG00000001631 | EIF2C4 | 0.179 | 0.032 | 24.0 | 6.0 | 0.6 |
| ENSTGUG00000004577 | SLC25A38 | 0.179 | 0.032 | 24.0 | 5.0 | 0.6 |
| ENSTGUG00000010116 | LDLRAD3 | 0.179 | 0.032 | 25.3 | 12.7 | 0.6 |
| ENSTGUG00000002489 | ETV1 | 0.179 | 0.032 | 24.0 | 8.0 | 0.6 |
| ENSTGUG00000002263 | FRAP1 | 0.179 | 0.032 | 24.0 | 17.0 | 0.6 |
| ENSTGUG00000005562 | BCS1L | 0.179 | 0.032 | 24.0 | 2.0 | 0.6 |
| ENSTGUG00000014761 |  | 0.179 | 0.032 | 24.0 | 21.0 | 0.6 |
| ENSTGUG00000000452 |  | 0.179 | 0.032 | 28.0 | 18.0 | 0.6 |
| ENSTGUG00000005083 | SNX29 | 0.179 | 0.032 | 24.0 | 21.0 | 0.6 |
| ENSTGUG00000008487 | NF1 | 0.179 | 0.032 | 24.0 | 13.0 | 0.6 |
| ENSTGUG00000002686 | COASY | 0.179 | 0.032 | 24.0 | 4.0 | 0.6 |
| ENSTGUG00000007733 | TNRC6C | 0.179 | 0.032 | 24.0 | 0.0 | 0.6 |
| ENSTGUG00000012514 | RPS6KA5 | 0.179 | 0.032 | 24.0 | 21.0 | 0.6 |
| ENSTGUG00000006318 | MON2 | 0.179 | 0.032 | 24.0 | 14.0 | 0.6 |
| ENSTGUG00000002640 | SLC10A7 | 0.179 | 0.032 | 24.0 | 18.0 | 0.6 |
| ENSTGUG00000011264 | PDE7A | 0.179 | 0.032 | 24.0 | 10.0 | 0.6 |
| ENSTGUG00000015363 |  | 0.179 | 0.032 | 26.0 | 18.0 | 0.6 |
| ENSTGUG00000012397 | GALC | 0.179 | 0.032 | 24.0 | 10.0 | 0.6 |
| ENSTGUG00000007075 | CXorf21 | 0.179 | 0.032 | 24.0 | 17.0 | 0.6 |
| ENSTGUG00000007503 | GZF1 | 0.179 | 0.032 | 24.0 | 1.0 | 0.5 |
| ENSTGUG00000012382 | STON2 | 0.179 | 0.032 | 24.0 | 21.0 | 0.5 |
| ENSTGUG00000001559 | MARVELD2 | 0.179 | 0.032 | 24.0 | 22.0 | 0.5 |
| ENSTGUG00000011596 | MKI67-5 | 0.179 | 0.032 | 24.0 | 13.0 | 0.5 |
| ENSTGUG00000003016 | RAP2C | 0.179 | 0.032 | 24.0 | 2.0 | 0.5 |
| ENSTGUG00000015040 | DDX24 | 0.179 | 0.032 | 24.0 | 22.0 | 0.5 |
| ENSTGUG00000002944 | SKI | 0.179 | 0.032 | 24.0 | 22.0 | 0.5 |
| ENSTGUG00000007430 | TRIM37 | 0.179 | 0.032 | 24.0 | 9.0 | 0.5 |
| ENSTGUG00000005877 | GFOD1 | 0.179 | 0.032 | 24.0 | 9.0 | 0.5 |
| ENSTGUG00000011514 | DOCK1 | 0.179 | 0.032 | 24.0 | 22.0 | 0.5 |
| ENSTGUG00000012145 | FZD6 | 0.179 | 0.032 | 24.0 | 14.0 | 0.5 |
| ENSTGUG00000012267 | LRCH1 | 0.179 | 0.032 | 24.0 | 2.0 | 0.5 |
| ENSTGUG00000004421 | ADAMTSL1-1 | 0.179 | 0.032 | 24.0 | 17.0 | 0.5 |
| ENSTGUG00000001545 | MTHFD2L | 0.179 | 0.032 | 24.0 | 0.0 | 0.5 |
| ENSTGUG00000006290 | SNED1 | 0.179 | 0.032 | 24.0 | 1.0 | 0.5 |
| ENSTGUG00000012048 | NEK3 | 0.179 | 0.032 | 24.0 | 17.0 | 0.5 |
| ENSTGUG00000004887 | RAD51L3 | 0.179 | 0.032 | 24.0 | 2.0 | 0.5 |
| ENSTGUG00000001738 | DEF6 | 0.179 | 0.032 | 26.0 | 18.0 | 0.5 |
| ENSTGUG00000001376 | BSDC1 | 0.179 | 0.032 | 24.0 | 2.0 | 0.5 |
| ENSTGUG00000003267 |  | 0.179 | 0.032 | 24.0 | 4.0 | 0.5 |
| ENSTGUG00000009849 | CDH11 | 0.179 | 0.032 | 24.0 | 12.0 | 0.5 |
| ENSTGUG00000005647 | F3 | 0.179 | 0.032 | 24.0 | 9.0 | 0.5 |
| ENSTGUG00000010128 | TNRC6B | 0.179 | 0.032 | 24.0 | 1.0 | 0.5 |
| ENSTGUG00000003150 | MAPK8IP3 | 0.179 | 0.032 | 26.0 | 5.0 | 0.5 |
| ENSTGUG00000005867 | C17orf48 | 0.179 | 0.032 | 24.0 | 0.0 | 0.5 |
| ENSTGUG00000010924 | COL5A2 | 0.179 | 0.032 | 24.0 | 8.0 | 0.5 |
| ENSTGUG00000000771 | MECR | 0.179 | 0.032 | 24.0 | 0.0 | 0.5 |
| ENSTGUG00000001828 | NPR2 | 0.179 | 0.032 | 24.0 | 4.0 | 0.5 |
| ENSTGUG00000012345 | TSC22D1 | 0.179 | 0.032 | 24.0 | 5.0 | 0.5 |
| ENSTGUG00000015985 | HCLS1 | 0.179 | 0.032 | 24.0 | 10.0 | 0.5 |
| ENSTGUG00000011347 | LATS1 | 0.179 | 0.032 | 24.0 | 1.0 | 0.5 |
| ENSTGUG00000012074 | FBXO33 | 0.179 | 0.032 | 24.0 | 20.0 | 0.5 |
| ENSTGUG00000003805 | SNTA1 | 0.179 | 0.032 | 24.0 | 12.0 | 0.5 |
| ENSTGUG00000000041 |  | 0.179 | 0.032 | 24.0 | 17.0 | 0.5 |
| ENSTGUG00000007968 | SUSD2 | 0.179 | 0.032 | 25.3 | 9.3 | 0.4 |
| ENSTGUG00000006243 | GALNT7 | 0.179 | 0.032 | 24.0 | 9.0 | 0.4 |
| ENSTGUG00000007250 | MELK | 0.179 | 0.032 | 24.0 | 14.0 | 0.4 |
| ENSTGUG00000008732 | HEATR5B | 0.179 | 0.032 | 24.0 | 12.0 | 0.4 |
| ENSTGUG00000007736 | PPEF1 | 0.179 | 0.032 | 24.0 | 22.0 | 0.4 |
| ENSTGUG00000002048 | EPHA2 | 0.179 | 0.032 | 24.0 | 8.0 | 0.4 |
| ENSTGUG00000010548 | WHSC2 | 0.179 | 0.032 | 24.0 | 12.0 | 0.4 |
| ENSTGUG00000007854 | UPP1 | 0.179 | 0.032 | 24.0 | 8.0 | 0.4 |
| ENSTGUG00000012587 | RIN3 | 0.179 | 0.032 | 24.0 | 13.0 | 0.4 |
| ENSTGUG00000013005 | GAB2 | 0.179 | 0.032 | 24.0 | 5.0 | 0.4 |
| ENSTGUG00000011779 | PDS5B | 0.179 | 0.032 | 24.0 | 12.0 | 0.4 |
| ENSTGUG00000017123 | AGMAT | 0.179 | 0.032 | 24.0 | 14.0 | 0.4 |
| ENSTGUG00000006452 | KCNT1 | 0.179 | 0.032 | 24.0 | 22.0 | 0.4 |
| ENSTGUG00000012237 | TGFB3 | 0.179 | 0.032 | 24.0 | 8.0 | 0.4 |
| ENSTGUG00000008720 | LMTK2 | 0.179 | 0.032 | 24.0 | 10.0 | 0.4 |
| ENSTGUG00000011995 | RBM43 | 0.179 | 0.032 | 24.0 | 8.0 | 0.4 |
| ENSTGUG00000013215 | GPR116 | 0.179 | 0.032 | 24.0 | 13.0 | 0.4 |
| ENSTGUG00000011729 |  | 0.179 | 0.032 | 24.0 | 4.0 | 0.4 |
| ENSTGUG00000004574 | CCR8 | 0.179 | 0.032 | 24.0 | 14.0 | 0.4 |
| ENSTGUG00000008551 | TSHZ2 | 0.179 | 0.032 | 24.0 | 0.0 | 0.4 |
| ENSTGUG00000005785 |  | 0.179 | 0.032 | 24.0 | 8.0 | 0.4 |
| ENSTGUG00000002705 | DUSP10 | 0.179 | 0.032 | 24.0 | 9.0 | 0.4 |
| ENSTGUG00000005541 | ABCG1 | 0.179 | 0.032 | 24.0 | 9.0 | 0.4 |
| ENSTGUG00000012202 | PKP4 | 0.179 | 0.032 | 24.0 | 8.0 | 0.4 |
| ENSTGUG00000001700 | PCDHA10 | 0.179 | 0.032 | 24.0 | 10.0 | 0.4 |
| ENSTGUG00000015593 | KIAA1310 | 0.179 | 0.032 | 24.0 | 4.0 | 0.4 |
| ENSTGUG00000003953 | SPNS3 | 0.179 | 0.032 | 24.0 | 14.0 | 0.4 |
| ENSTGUG00000000053 | MYO5B | 0.179 | 0.032 | 24.0 | 22.0 | 0.4 |
| ENSTGUG00000017236 |  | 0.179 | 0.032 | 25.3 | 9.3 | 0.4 |
| ENSTGUG00000012648 | COL12A1 | 0.179 | 0.032 | 24.0 | 6.0 | 0.4 |
| ENSTGUG00000010730 | FOXRED2 | 0.179 | 0.032 | 24.0 | 2.0 | 0.4 |
| ENSTGUG00000009276 |  | 0.179 | 0.032 | 24.0 | 13.0 | 0.4 |
| ENSTGUG00000016768 |  | 0.179 | 0.032 | 24.0 | 2.0 | 0.4 |
| ENSTGUG00000001064 | KLHL26 | 0.179 | 0.032 | 24.0 | 8.0 | 0.4 |
| ENSTGUG00000018200 |  | 0.179 | 0.032 | 24.0 | 1.0 | 0.4 |
| ENSTGUG00000016497 |  | 0.179 | 0.032 | 24.0 | 5.0 | 0.4 |
| ENSTGUG00000008714 | TNFAIP1 | 0.179 | 0.032 | 28.0 | 0.0 | 0.4 |
| ENSTGUG00000007715 | PLK3 | 0.179 | 0.032 | 24.0 | 1.0 | 0.4 |
| ENSTGUG00000007476 | CHD9 | 0.179 | 0.032 | 24.0 | 4.0 | 0.4 |
| ENSTGUG00000001528 | DOCK2 | 0.179 | 0.032 | 24.0 | 13.0 | 0.4 |
| ENSTGUG00000005272 |  | 0.179 | 0.032 | 24.0 | 14.0 | 0.4 |
| ENSTGUG00000012029 |  | 0.179 | 0.032 | 28.0 | 0.0 | 0.4 |
| ENSTGUG00000004916 | CYCB3 | 0.179 | 0.032 | 24.0 | 12.0 | 0.4 |
| ENSTGUG00000009965 | COG2 | 0.179 | 0.032 | 24.0 | 0.0 | 0.4 |
| ENSTGUG00000008593 | KSR1 | 0.179 | 0.032 | 24.0 | 2.0 | 0.3 |
| ENSTGUG00000009822 | CPEB2 | 0.179 | 0.032 | 24.0 | 1.0 | 0.3 |
| ENSTGUG00000015563 | GATAD2B | 0.179 | 0.032 | 24.0 | 13.0 | 0.3 |
| ENSTGUG00000013226 | FANCM | 0.179 | 0.032 | 24.0 | 22.0 | 0.3 |
| ENSTGUG00000007759 | SH3GL3 | 0.179 | 0.032 | 24.0 | 12.0 | 0.3 |
| ENSTGUG00000003945 | HK1 | 0.179 | 0.032 | 24.0 | 13.0 | 0.3 |
| ENSTGUG00000004899 | FAM78A | 0.179 | 0.032 | 24.0 | 9.0 | 0.3 |
| ENSTGUG00000009427 | CA10 | 0.179 | 0.032 | 24.0 | 14.0 | 0.3 |
| ENSTGUG00000010682 | SASH1 | 0.179 | 0.032 | 24.0 | 10.0 | 0.3 |
| ENSTGUG00000010832 | JMJD1A | 0.179 | 0.032 | 24.0 | 1.0 | 0.3 |
| ENSTGUG00000012041 | CKAP2 | 0.179 | 0.032 | 24.0 | 17.0 | 0.3 |
| ENSTGUG00000011644 | JAKMIP3 | 0.179 | 0.032 | 24.0 | 17.0 | 0.3 |
| ENSTGUG00000005477 |  | 0.179 | 0.032 | 24.0 | 9.0 | 0.3 |
| ENSTGUG00000003151 | KIAA1219 | 0.179 | 0.032 | 24.0 | 20.0 | 0.3 |
| ENSTGUG00000009444 | C10orf33 | 0.179 | 0.032 | 24.0 | 6.0 | 0.3 |
| ENSTGUG00000007071 |  | 0.179 | 0.032 | 25.3 | 8.0 | 0.3 |
| ENSTGUG00000017419 | ASH1L | 0.179 | 0.032 | 25.3 | 9.3 | 0.3 |
| ENSTGUG00000009574 | C10orf28 | 0.179 | 0.032 | 24.0 | 18.0 | 0.3 |
| ENSTGUG00000004541 | C20orf95 | 0.179 | 0.032 | 24.0 | 16.0 | 0.3 |
| ENSTGUG00000006692 | RECK | 0.179 | 0.032 | 24.0 | 13.0 | 0.3 |
| ENSTGUG00000013243 | RCAN2 | 0.179 | 0.032 | 25.3 | 4.0 | 0.3 |
| ENSTGUG00000002599 | RGS10-1 | 0.179 | 0.032 | 25.3 | 12.7 | 0.3 |
| ENSTGUG00000011809 | RNF217 | 0.179 | 0.032 | 28.0 | 2.0 | 0.3 |
| ENSTGUG00000004534 | KPNA2 | 0.179 | 0.032 | 24.0 | 18.0 | 0.3 |
| ENSTGUG00000003188 | ASB1 | 0.179 | 0.032 | 28.0 | 0.0 | 0.3 |
| ENSTGUG00000000085 | RBM27 | 0.179 | 0.032 | 24.0 | 14.0 | 0.3 |
| ENSTGUG00000006377 | ZNRF1 | 0.179 | 0.032 | 24.0 | 4.0 | 0.3 |
| ENSTGUG00000008547 | TRRAP | 0.179 | 0.032 | 26.0 | 15.0 | 0.3 |
| ENSTGUG00000015642 | GAD1-3 | 0.179 | 0.032 | 24.0 | 2.0 | 0.3 |
| ENSTGUG00000017405 | GOLPH3L | 0.179 | 0.032 | 28.0 | 0.0 | 0.3 |
| ENSTGUG00000011029 | HSPA12A | 0.179 | 0.032 | 24.0 | 12.0 | 0.3 |
| ENSTGUG00000016726 |  | 0.179 | 0.032 | 24.0 | 13.0 | 0.3 |
| ENSTGUG00000015197 |  | 0.179 | 0.032 | 24.0 | 13.0 | 0.3 |
| ENSTGUG00000016192 | GALNTL4 | 0.179 | 0.032 | 24.0 | 0.0 | 0.3 |
| ENSTGUG00000008498 | XRN1 | 0.179 | 0.032 | 24.0 | 18.0 | 0.3 |
| ENSTGUG00000002314 | SERTAD2 | 0.179 | 0.032 | 24.0 | 2.0 | 0.3 |
| ENSTGUG00000011362 | P2RY14 | 0.179 | 0.032 | 24.0 | 13.0 | 0.3 |
| ENSTGUG00000010200 | CRBN | 0.179 | 0.032 | 24.0 | 8.0 | 0.3 |
| ENSTGUG00000005467 | ADIPOQ | 0.179 | 0.032 | 25.3 | 4.0 | 0.3 |
| ENSTGUG00000005025 |  | 0.179 | 0.032 | 24.0 | 12.0 | 0.3 |
| ENSTGUG00000012639 | TRAPPC9 | 0.179 | 0.032 | 24.0 | 14.0 | 0.3 |
| ENSTGUG00000010614 | PHF16 | 0.179 | 0.032 | 24.0 | 10.0 | 0.3 |
| ENSTGUG00000004168 | DPYSL2 | 0.179 | 0.032 | 24.0 | 2.0 | 0.3 |
| ENSTGUG00000013157 | TULP3 | 0.179 | 0.032 | 24.0 | 20.0 | 0.3 |
| ENSTGUG00000000160 | SPRY4 | 0.179 | 0.032 | 28.0 | 0.0 | 0.3 |
| ENSTGUG00000007608 | G6PC2 | 0.179 | 0.032 | 24.0 | 0.0 | 0.3 |
| ENSTGUG00000009885 | USP6NL-2 | 0.179 | 0.032 | 26.0 | 15.0 | 0.3 |
| ENSTGUG00000017291 | RHOBTB2 | 0.179 | 0.032 | 24.0 | 4.0 | 0.3 |
| ENSTGUG00000006100 | SH3RF1 | 0.179 | 0.032 | 24.0 | 9.0 | 0.3 |
| ENSTGUG00000002846 | ZC3H12B | 0.179 | 0.032 | 24.0 | 0.0 | 0.3 |
| ENSTGUG00000012697 | DYNC2H1 | 0.179 | 0.032 | 24.0 | 16.0 | 0.3 |
| ENSTGUG00000011108 | ZFP106 | 0.179 | 0.032 | 24.0 | 13.0 | 0.3 |
| ENSTGUG00000002349 | SPRED2 | 0.179 | 0.032 | 24.0 | 6.0 | 0.3 |
| ENSTGUG00000010716 | TMEFF2 | 0.179 | 0.032 | 28.0 | 0.0 | 0.3 |
| ENSTGUG00000010412 | KCNJ4 | 0.179 | 0.032 | 24.0 | 13.0 | 0.3 |
| ENSTGUG00000006733 | FANCD2 | 0.179 | 0.032 | 24.0 | 13.0 | 0.3 |
| ENSTGUG00000010567 | ANKRD44 | 0.179 | 0.032 | 24.0 | 13.0 | 0.3 |
| ENSTGUG00000009770 | PARD3B | 0.179 | 0.032 | 24.0 | 6.0 | 0.3 |
| ENSTGUG00000012690 | DENND3 | 0.179 | 0.032 | 24.0 | 21.0 | 0.3 |
| ENSTGUG00000006906 | FRS2 | 0.179 | 0.032 | 24.0 | 22.0 | 0.3 |
| ENSTGUG00000015416 | ALMS1-1 | 0.179 | 0.032 | 24.0 | 4.0 | 0.2 |
| ENSTGUG00000007739 | INTS2 | 0.179 | 0.032 | 24.0 | 17.0 | 0.2 |
| ENSTGUG00000009343 | TNNT3 | 0.179 | 0.032 | 24.0 | 12.0 | 0.2 |
| ENSTGUG00000011533 | ZFYVE26-1 | 0.179 | 0.032 | 24.0 | 14.0 | 0.2 |
| ENSTGUG00000012646 | SLC6A13 | 0.179 | 0.032 | 24.0 | 13.0 | 0.2 |
| ENSTGUG00000010923 | AFAP1L2 | 0.179 | 0.032 | 24.0 | 5.0 | 0.2 |
| ENSTGUG00000012492 | DACH1 | 0.179 | 0.032 | 24.0 | 9.0 | 0.2 |
| ENSTGUG00000003617 | HMCN1 | 0.179 | 0.032 | 24.0 | 17.0 | 0.2 |
| ENSTGUG00000002101 | SERPINB1 | 0.179 | 0.032 | 24.0 | 13.0 | 0.2 |
| ENSTGUG00000013006 | ASAP2 | 0.179 | 0.032 | 24.0 | 8.0 | 0.2 |
| ENSTGUG00000012962 | PXDN | 0.179 | 0.032 | 24.0 | 8.0 | 0.2 |
| ENSTGUG00000014695 |  | 0.179 | 0.032 | 24.0 | 0.0 | 0.2 |
| ENSTGUG00000011790 | MCM6 | 0.179 | 0.032 | 24.0 | 10.0 | 0.2 |
| ENSTGUG00000013255 |  | 0.179 | 0.032 | 24.0 | 8.0 | 0.2 |
| ENSTGUG00000004624 | FAM149B1 | 0.179 | 0.032 | 24.0 | 14.0 | 0.2 |
| ENSTGUG00000000975 | HMGXB3 | 0.179 | 0.032 | 24.0 | 6.0 | 0.2 |
| ENSTGUG00000011488 | AHI1 | 0.179 | 0.032 | 24.0 | 22.0 | 0.2 |
| ENSTGUG00000017219 |  | 0.179 | 0.032 | 24.0 | 9.0 | 0.2 |
| ENSTGUG00000010956 |  | 0.179 | 0.032 | 24.0 | 2.0 | 0.2 |
| ENSTGUG00000015645 | BAX | 0.179 | 0.032 | 24.0 | 2.0 | 0.2 |
| ENSTGUG00000004794 |  | 0.179 | 0.032 | 24.0 | 8.0 | 0.2 |
| ENSTGUG00000007247 | MYO1H | 0.179 | 0.032 | 24.0 | 9.0 | 0.2 |
| ENSTGUG00000000589 | FLI1 | 0.179 | 0.032 | 24.0 | 8.0 | 0.2 |
| ENSTGUG00000008128 | WWP2 | 0.179 | 0.032 | 24.0 | 2.0 | 0.2 |
| ENSTGUG00000008522 | SOX6 | 0.179 | 0.032 | 24.0 | 5.0 | 0.2 |
| ENSTGUG00000014268 |  | 0.179 | 0.032 | 24.0 | 2.0 | 0.2 |
| ENSTGUG00000000167 | TBXA2R | 0.179 | 0.032 | 24.0 | 10.0 | 0.2 |
| ENSTGUG00000012509 | KIAA1009 | 0.179 | 0.032 | 24.0 | 16.0 | 0.2 |
| ENSTGUG00000012377 | DGKH | 0.179 | 0.032 | 24.0 | 12.0 | 0.2 |
| ENSTGUG00000012411 | HAS2 | 0.179 | 0.032 | 24.0 | 13.0 | 0.2 |
| ENSTGUG00000002523 | SUPT7L | 0.179 | 0.032 | 24.0 | 2.0 | 0.2 |
| ENSTGUG00000004569 | SMURF2 | 0.179 | 0.032 | 24.0 | 21.0 | 0.2 |
| ENSTGUG00000004199 | LRIT3 | 0.179 | 0.032 | 24.0 | 10.0 | 0.2 |
| ENSTGUG00000010019 | CYTSA | 0.179 | 0.032 | 24.0 | 14.0 | 0.2 |
| ENSTGUG00000012011 | POP1 | 0.179 | 0.032 | 24.0 | 22.0 | 0.2 |
| ENSTGUG00000001954 | TRPC3 | 0.179 | 0.032 | 24.0 | 6.0 | 0.2 |
| ENSTGUG00000003072 | SLC31A2 | 0.179 | 0.032 | 24.0 | 14.0 | 0.2 |
| ENSTGUG00000002081 | DDI2 | 0.179 | 0.032 | 24.0 | 13.0 | 0.2 |
| ENSTGUG00000008637 | OGG1 | 0.179 | 0.032 | 24.0 | 2.0 | 0.2 |
| ENSTGUG00000000783 | DIP2C | 0.179 | 0.032 | 24.0 | 9.0 | 0.2 |
| ENSTGUG00000002879 | RASGEF1B | 0.179 | 0.032 | 24.0 | 8.0 | 0.2 |
| ENSTGUG00000007055 | NFX1 | 0.179 | 0.032 | 24.0 | 22.0 | 0.2 |
| ENSTGUG00000007132 | NLGN3 | 0.179 | 0.032 | 24.0 | 20.0 | 0.2 |
| ENSTGUG00000017393 | POGZ | 0.179 | 0.032 | 24.0 | 5.0 | 0.2 |
| ENSTGUG00000014492 |  | 0.179 | 0.032 | 24.0 | 13.0 | 0.2 |
| ENSTGUG00000011289 | MYBL1 | 0.179 | 0.032 | 24.0 | 12.0 | 0.2 |
| ENSTGUG00000004851 | UNC45B | 0.179 | 0.032 | 24.0 | 12.0 | 0.2 |
| ENSTGUG00000005286 | PIK3CB | 0.179 | 0.032 | 24.0 | 2.0 | 0.2 |
| ENSTGUG00000001745 | ZNF76 | 0.179 | 0.032 | 24.0 | 18.0 | 0.2 |
| ENSTGUG00000002522 | DGKB | 0.179 | 0.032 | 26.0 | 5.0 | 0.2 |
| ENSTGUG00000009267 |  | 0.179 | 0.032 | 24.0 | 13.0 | 0.2 |
| ENSTGUG00000013826 |  | 0.179 | 0.032 | 24.0 | 12.0 | 0.2 |
| ENSTGUG00000008444 | RECQL5 | 0.179 | 0.032 | 24.0 | 0.0 | 0.2 |
| ENSTGUG00000007668 | FSCN1 | 0.179 | 0.032 | 24.0 | 6.0 | 0.2 |
| ENSTGUG00000009411 | TMC7 | 0.179 | 0.032 | 24.0 | 13.0 | 0.2 |
| ENSTGUG00000005347 | PSKH1 | 0.179 | 0.032 | 24.0 | 2.0 | 0.2 |
| ENSTGUG00000007001 | CCDC88A | 0.179 | 0.032 | 24.0 | 9.0 | 0.2 |
| ENSTGUG00000000661 | GRIN3A | 0.179 | 0.032 | 24.0 | 8.0 | 0.2 |
| ENSTGUG00000000782 | SLC36A1 | 0.179 | 0.032 | 24.0 | 6.0 | 0.2 |
| ENSTGUG00000012407 | ANKRD6 | 0.179 | 0.032 | 24.0 | 13.0 | 0.2 |
| ENSTGUG00000005376 | EXOC3L | 0.179 | 0.032 | 24.0 | 1.0 | 0.2 |
| ENSTGUG00000007663 | LRP2 | 0.179 | 0.032 | 25.3 | 20.7 | 0.2 |
| ENSTGUG00000010902 | KIAA1244 | 0.179 | 0.032 | 24.0 | 9.0 | 0.2 |
| ENSTGUG00000016909 | CDK5RAP3 | 0.179 | 0.032 | 24.0 | 9.0 | 0.2 |
| ENSTGUG00000002076 | DHX57 | 0.179 | 0.032 | 24.0 | 16.0 | 0.2 |
| ENSTGUG00000003940 | TFAP4 | 0.179 | 0.032 | 24.0 | 9.0 | 0.2 |
| ENSTGUG00000012450 | KCNK13 | 0.179 | 0.032 | 24.0 | 9.0 | 0.2 |
| ENSTGUG00000013516 | IGSF2 | 0.179 | 0.032 | 24.0 | 5.0 | 0.2 |
| ENSTGUG00000002229 | OXCT1 | 0.179 | 0.032 | 24.0 | 12.0 | 0.2 |
| ENSTGUG00000010247 | CKMT1A | 0.179 | 0.032 | 24.0 | 2.0 | 0.2 |
| ENSTGUG00000003355 | CLDN5 | 0.179 | 0.032 | 24.0 | 10.0 | 0.1 |
| ENSTGUG00000013496 | EPHA6 | 0.179 | 0.032 | 28.0 | 0.0 | 0.1 |
| ENSTGUG00000005789 | MYOCD | 0.179 | 0.032 | 28.0 | 0.0 | 0.1 |
| ENSTGUG00000013024 | KIAA0586 | 0.179 | 0.032 | 24.0 | 16.0 | 0.1 |
| ENSTGUG00000003985 | LAMC1 | 0.179 | 0.032 | 24.0 | 9.0 | 0.1 |
| ENSTGUG00000011552 | EYA4 | 0.179 | 0.032 | 24.0 | 17.0 | 0.1 |
| ENSTGUG00000016528 |  | 0.179 | 0.032 | 25.3 | 4.0 | 0.1 |
| ENSTGUG00000008401 | RIC3 | 0.179 | 0.032 | 24.0 | 21.0 | 0.1 |
| ENSTGUG00000007734 | PITPNM1 | 0.179 | 0.032 | 28.0 | 2.0 | 0.1 |
| ENSTGUG00000013137 | C3AR1 | 0.179 | 0.032 | 24.0 | 4.0 | 0.1 |
| ENSTGUG00000008799 | C17orf28 | 0.179 | 0.032 | 24.0 | 6.0 | 0.1 |
| ENSTGUG00000003782 |  | 0.179 | 0.032 | 24.0 | 2.0 | 0.1 |
| ENSTGUG00000000602 | TRIM3 | 0.179 | 0.032 | 24.0 | 9.0 | 0.1 |
| ENSTGUG00000006716 | HSF4 | 0.179 | 0.032 | 24.0 | 5.0 | 0.1 |
| ENSTGUG00000002698 | FAM155B | 0.179 | 0.032 | 24.0 | 0.0 | 0.1 |
| ENSTGUG00000004684 | SETDB1 | 0.179 | 0.032 | 24.0 | 2.0 | 0.1 |
| ENSTGUG00000011607 | FLT3 | 0.179 | 0.032 | 24.0 | 10.0 | 0.1 |
| ENSTGUG00000005701 |  | 0.179 | 0.032 | 24.0 | 12.0 | 0.1 |
| ENSTGUG00000017516 | ELK4 | 0.179 | 0.032 | 24.0 | 2.0 | 0.1 |
| ENSTGUG00000004517 | AP3B2 | 0.179 | 0.032 | 24.0 | 13.0 | 0.1 |
| ENSTGUG00000010831 | MCM5 | 0.179 | 0.032 | 24.0 | 9.0 | 0.1 |
| ENSTGUG00000003521 | TRPM8 | 0.179 | 0.032 | 24.0 | 9.0 | 0.1 |
| ENSTGUG00000002570 |  | 0.179 | 0.032 | 26.0 | 2.0 | 0.1 |
| ENSTGUG00000015456 | EVPLL | 0.179 | 0.032 | 24.0 | 6.0 | 0.1 |
| ENSTGUG00000007429 | SAG | 0.179 | 0.032 | 24.0 | 8.0 | 0.1 |
| ENSTGUG00000010189 | AFAP1 | 0.179 | 0.032 | 24.0 | 9.0 | 0.1 |
| ENSTGUG00000001207 | TRPC7 | 0.179 | 0.032 | 28.0 | 0.0 | 0.1 |
| ENSTGUG00000002536 | CACNA2D1 | 0.179 | 0.032 | 24.0 | 10.0 | 0.1 |
| ENSTGUG00000012592 | PHIP | 0.179 | 0.032 | 24.0 | 9.0 | 0.1 |
| ENSTGUG00000007273 | GALNT3 | 0.179 | 0.032 | 24.0 | 12.0 | 0.1 |
| ENSTGUG00000002672 | MAGI2 | 0.179 | 0.032 | 24.0 | 2.0 | 0.1 |
| ENSTGUG00000015343 |  | 0.179 | 0.032 | 24.0 | 12.0 | 0.1 |
| ENSTGUG00000011195 | SYNE1 | 0.179 | 0.032 | 24.0 | 12.0 | 0.1 |
| ENSTGUG00000011433 | SULF1 | 0.179 | 0.032 | 24.0 | 4.0 | 0.1 |
| ENSTGUG00000011503 | TRPA1 | 0.179 | 0.032 | 24.0 | 12.0 | 0.1 |
| ENSTGUG00000004249 | ASPM | 0.179 | 0.032 | 24.0 | 13.0 | 0.1 |
| ENSTGUG00000015149 |  | 0.179 | 0.032 | 24.0 | 12.0 | 0.1 |
| ENSTGUG00000004156 |  | 0.179 | 0.032 | 24.0 | 12.0 | 0.1 |
| ENSTGUG00000002743 | TP73 | 0.179 | 0.032 | 24.0 | 2.0 | 0.1 |
| ENSTGUG00000006382 | COL24A1 | 0.179 | 0.032 | 24.0 | 12.0 | 0.1 |
| ENSTGUG00000000246 | GJC2 | 0.179 | 0.032 | 24.0 | 2.0 | 0.1 |
| ENSTGUG00000012642 | PPP4R4 | 0.179 | 0.032 | 24.0 | 12.0 | 0.1 |
| ENSTGUG00000001100 | ZNF608 | 0.179 | 0.032 | 24.0 | 2.0 | 0.1 |
| ENSTGUG00000013839 | THBS3 | 0.179 | 0.032 | 24.0 | 12.0 | 0.1 |
| ENSTGUG00000004092 | CHRNB2 | 0.179 | 0.032 | 24.0 | 12.0 | 0.1 |
| ENSTGUG00000006469 | C20orf112 | 0.179 | 0.032 | 24.0 | 13.0 | 0.0 |
| ENSTGUG00000010791 |  | 0.179 | 0.032 | 24.0 | 13.0 | 0.0 |
| ENSTGUG00000000659 | C9orf125 | 0.179 | 0.032 | 24.0 | 12.0 | 0.0 |
| ENSTGUG00000016061 | FLNC | 0.179 | 0.032 | 24.0 | 10.0 | 0.0 |
| ENSTGUG00000003164 | BMPR1B | 0.179 | 0.032 | 24.0 | 13.0 | 0.0 |
| ENSTGUG00000010375 | CBY1 | 0.179 | 0.032 | 24.0 | 12.0 | 0.0 |
| ENSTGUG00000011716 |  | 0.179 | 0.032 | 24.0 | 2.0 | 0.0 |
| ENSTGUG00000004923 | PRAGMIN | 0.179 | 0.032 | 24.0 | 2.0 | 0.0 |
| ENSTGUG00000003635 | WNK2 | 0.179 | 0.032 | 24.0 | 9.0 | 0.0 |
| ENSTGUG00000007999 | FHOD3 | 0.179 | 0.032 | 24.0 | 8.0 | 0.0 |
| ENSTGUG00000000593 | EBF1 | 0.179 | 0.032 | 24.0 | 13.0 | 0.0 |
| ENSTGUG00000011605 | ZFHX4 | 0.179 | 0.032 | 24.0 | 0.0 | 0.0 |
| ENSTGUG00000010364 | NEGR1 | 0.179 | 0.032 | 24.0 | 14.0 | 0.0 |
| ENSTGUG00000007230 | DNHD2 | 0.179 | 0.032 | 24.0 | 10.0 | 0.0 |
| ENSTGUG00000012890 | MCPH1 | 0.179 | 0.032 | 24.0 | 2.0 | 0.0 |
| ENSTGUG00000010491 | RYR2 | 0.179 | 0.032 | 24.0 | 6.0 | 0.0 |
| ENSTGUG00000007778 | PPFIA2 | 0.179 | 0.032 | 24.0 | 4.0 | 0.0 |
| ENSTGUG00000005071 | TRAIP | 0.179 | 0.032 | 24.0 | 12.0 | 0.0 |
|  |  |  |  |  |  |  |

Common cycling genes

| **EnsemblGeneID** | **GeneSymbol** | **M_ BHQ** | **M_ ADJP** | **M_ PERIOD** | **M_ LAG** | **M_ AMP** | **nM_ BHQ** | **nM_ ADJP** | **nM_ PERIOD** | **nM_ LAG** | **nM_ AMP** |  |
| --- | --- | --- | --- | --- | --- | --- | --- | --- | --- | --- | --- | --- |
| ENSTGUG00000017542 | AP3S1 | 0.003 | 0.000 | 24.0 | 18.0 | 13.6 | 0.18 | 0.03 | 24.0 | 14.0 | 5.8 | |
| ENSTGUG00000009654 | SERHL2 | 0.003 | 0.000 | 24.0 | 6.0 | 11.6 | 0.18 | 0.03 | 24.0 | 4.0 | 6.9 | |
| ENSTGUG00000012423 | PNRC1 | 0.003 | 0.000 | 24.0 | 10.0 | 9.7 | 0.10 | 0.01 | 28.0 | 1.0 | 38.3 | |
| ENSTGUG00000004459 | DNAJB12 | 0.003 | 0.000 | 24.0 | 14.0 | 9.2 | 0.03 | 0.00 | 24.0 | 1.0 | 4.6 | |
| ENSTGUG00000005460 | SMARCA2 | 0.003 | 0.000 | 24.0 | 2.0 | 7.8 | 0.10 | 0.01 | 28.0 | 17.0 | 2.5 | |
| ENSTGUG00000009473 |  | 0.003 | 0.000 | 24.0 | 18.0 | 5.7 | 0.00 | 0.00 | 24.0 | 14.0 | 2.3 | |
| ENSTGUG00000010236 | SH3TC1 | 0.003 | 0.000 | 24.0 | 4.0 | 4.9 | 0.03 | 0.00 | 24.0 | 22.0 | 2.4 | |
| ENSTGUG00000009204 | ANKRD40-1 | 0.003 | 0.000 | 24.0 | 18.0 | 4.8 | 0.10 | 0.01 | 28.0 | 17.0 | 1.6 | |
| ENSTGUG00000015317 |  | 0.003 | 0.000 | 24.0 | 8.0 | 4.5 | 0.18 | 0.03 | 25.3 | 8.0 | 4.6 | |
| ENSTGUG00000010331 | TP53BP1 | 0.003 | 0.000 | 24.0 | 16.0 | 2.8 | 0.18 | 0.03 | 25.3 | 16.7 | 2.4 | |
| ENSTGUG00000017057 | ATF6 | 0.003 | 0.000 | 24.0 | 4.0 | 2.6 | 0.00 | 0.00 | 24.0 | 2.0 | 4.0 | |
| ENSTGUG00000010264 | TBCE | 0.003 | 0.000 | 24.0 | 18.0 | 2.4 | 0.03 | 0.00 | 26.0 | 18.0 | 2.3 | |
| ENSTGUG00000010530 | MXD4 | 0.003 | 0.000 | 24.0 | 4.0 | 2.1 | 0.18 | 0.03 | 24.0 | 10.0 | 10.0 | |
| ENSTGUG00000002216 | CAMK1D | 0.003 | 0.000 | 24.0 | 2.0 | 1.7 | 0.18 | 0.03 | 24.0 | 4.0 | 1.7 | |
| ENSTGUG00000011929 | ORC4L | 0.003 | 0.000 | 24.0 | 14.0 | 1.6 | 0.03 | 0.00 | 26.0 | 15.0 | 2.4 | |
| ENSTGUG00000011050 | TGS1 | 0.003 | 0.000 | 24.0 | 16.0 | 1.4 | 0.18 | 0.03 | 25.3 | 20.7 | 0.4 | |
| ENSTGUG00000008456 | HECTD2 | 0.003 | 0.000 | 24.0 | 10.0 | 1.3 | 0.00 | 0.00 | 24.0 | 14.0 | 2.2 | |
| ENSTGUG00000010415 | SRGAP3 | 0.003 | 0.000 | 24.0 | 6.0 | 1.1 | 0.03 | 0.00 | 24.0 | 8.0 | 1.6 | |
| ENSTGUG00000000335 | SIK2 | 0.003 | 0.000 | 24.0 | 8.0 | 0.9 | 0.18 | 0.03 | 26.0 | 5.0 | 1.5 | |
| ENSTGUG00000007348 | C15orf48 | 0.003 | 0.000 | 24.0 | 4.0 | 0.9 | 0.18 | 0.03 | 24.0 | 17.0 | 0.4 | |
| ENSTGUG00000008737 | SGSM2 | 0.003 | 0.000 | 24.0 | 10.0 | 0.8 | 0.03 | 0.00 | 24.0 | 12.0 | 1.1 | |
| ENSTGUG00000009812 | DOCK7 | 0.003 | 0.000 | 24.0 | 4.0 | 0.7 | 0.03 | 0.00 | 25.3 | 4.0 | 1.3 | |
| ENSTGUG00000013523 | BCL9 | 0.003 | 0.000 | 24.0 | 6.0 | 0.6 | 0.10 | 0.01 | 28.0 | 1.0 | 0.3 | |
| ENSTGUG00000007964 | ZRSR2 | 0.003 | 0.000 | 24.0 | 18.0 | 0.6 | 0.18 | 0.03 | 24.0 | 17.0 | 0.3 | |
| ENSTGUG00000005884 | SIK1 | 0.003 | 0.000 | 24.0 | 12.0 | 0.6 | 0.18 | 0.03 | 28.0 | 2.0 | 2.5 | |
| ENSTGUG00000007307 | TTC21B | 0.003 | 0.000 | 24.0 | 18.0 | 0.5 | 0.18 | 0.03 | 25.3 | 16.7 | 1.2 | |
| ENSTGUG00000017498 |  | 0.003 | 0.000 | 24.0 | 6.0 | 0.4 | 0.03 | 0.00 | 24.0 | 2.0 | 0.8 | |
| ENSTGUG00000004706 | CUL9 | 0.003 | 0.000 | 24.0 | 14.0 | 0.4 | 0.18 | 0.03 | 24.0 | 4.0 | 0.4 | |
| ENSTGUG00000006057 | ANXA10-2 | 0.003 | 0.000 | 24.0 | 22.0 | 0.4 | 0.18 | 0.03 | 24.0 | 14.0 | 0.7 | |
| ENSTGUG00000018287 |  | 0.003 | 0.000 | 24.0 | 18.0 | 0.3 | 0.10 | 0.01 | 24.0 | 13.0 | 0.2 | |
| ENSTGUG00000010524 | ZFYVE28 | 0.003 | 0.000 | 24.0 | 16.0 | 0.3 | 0.03 | 0.00 | 24.0 | 14.0 | 0.4 | |
| ENSTGUG00000003345 | SOCS3 | 0.003 | 0.000 | 24.0 | 18.0 | 0.3 | 0.00 | 0.00 | 24.0 | 2.0 | 0.6 | |
| ENSTGUG00000005605 | GPR141 | 0.003 | 0.000 | 24.0 | 4.0 | 0.1 | 0.10 | 0.01 | 24.0 | 11.0 | 0.2 | |
| ENSTGUG00000003637 | ACCN3 | 0.029 | 0.000 | 24.0 | 8.0 | 0.4 | 0.03 | 0.00 | 24.0 | 12.0 | 0.5 | |
| ENSTGUG00000002773 | IGF2BP3 | 0.029 | 0.000 | 24.0 | 2.0 | 0.2 | 0.03 | 0.00 | 24.0 | 8.0 | 0.1 | |
| ENSTGUG00000002396 | PCDH11X | 0.029 | 0.000 | 24.0 | 8.0 | 0.1 | 0.18 | 0.03 | 24.0 | 12.0 | 0.0 | |
| ENSTGUG00000003719 | FASN | 0.035 | 0.002 | 24.0 | 2.0 | 1335.1 | 0.18 | 0.03 | 25.3 | 4.0 | 903.6 | |
| ENSTGUG00000009166 | CYB5A | 0.035 | 0.002 | 24.0 | 5.0 | 714.7 | 0.18 | 0.03 | 24.0 | 6.0 | 373.8 | |
| ENSTGUG00000011254 | PAH | 0.035 | 0.002 | 24.0 | 4.0 | 459.2 | 0.03 | 0.00 | 24.0 | 2.0 | 507.5 | |
| ENSTGUG00000005403 | TDO2 | 0.035 | 0.002 | 24.0 | 4.0 | 455.6 | 0.18 | 0.03 | 24.0 | 4.0 | 359.8 | |
| ENSTGUG00000005767 | ELOVL2 | 0.035 | 0.002 | 24.0 | 16.0 | 257.7 | 0.03 | 0.00 | 24.0 | 14.0 | 332.8 | |
| ENSTGUG00000011476 | LPA-2 | 0.035 | 0.002 | 24.0 | 5.0 | 257.4 | 0.10 | 0.01 | 28.0 | 1.0 | 613.3 | |
| ENSTGUG00000016716 |  | 0.035 | 0.002 | 26.0 | 2.0 | 249.1 | 0.18 | 0.03 | 24.0 | 4.0 | 65.8 | |
| ENSTGUG00000001620 | SLC25A13 | 0.035 | 0.002 | 26.0 | 2.0 | 213.8 | 0.18 | 0.03 | 24.0 | 4.0 | 13.1 | |
| ENSTGUG00000004622 | DDX5 | 0.035 | 0.002 | 24.0 | 2.0 | 177.0 | 0.18 | 0.03 | 24.0 | 21.0 | 77.4 | |
| ENSTGUG00000005750 | GLUD1 | 0.035 | 0.002 | 24.0 | 20.0 | 142.7 | 0.03 | 0.00 | 25.3 | 16.7 | 95.6 | |
| ENSTGUG00000005248 | FGB | 0.003 | 0.000 | 24.0 | 2.0 | 945.6 | 0.03 | 0.00 | 24.0 | 2.0 | 951.7 | |
| ENSTGUG00000001195 | HMGCL | 0.003 | 0.000 | 24.0 | 18.0 | 387.3 | 0.00 | 0.00 | 24.0 | 20.0 | 464.1 | |
| ENSTGUG00000010450 | HRG-2 | 0.003 | 0.000 | 24.0 | 4.0 | 230.3 | 0.03 | 0.00 | 25.3 | 4.0 | 1115.5 | |
| ENSTGUG00000004004 | HADH | 0.003 | 0.000 | 24.0 | 16.0 | 101.6 | 0.03 | 0.00 | 24.0 | 14.0 | 221.1 | |
| ENSTGUG00000011577 | GPHN | 0.003 | 0.000 | 24.0 | 6.0 | 68.1 | 0.03 | 0.00 | 25.3 | 4.0 | 44.3 | |
| ENSTGUG00000000086 | ANGPTL4 | 0.003 | 0.000 | 24.0 | 2.0 | 60.7 | 0.18 | 0.03 | 24.0 | 6.0 | 62.5 | |
| ENSTGUG00000004611 | ABAT | 0.003 | 0.000 | 24.0 | 4.0 | 55.6 | 0.03 | 0.00 | 24.0 | 2.0 | 63.6 | |
| ENSTGUG00000004414 | UGT2A1 | 0.003 | 0.000 | 24.0 | 4.0 | 52.8 | 0.18 | 0.03 | 24.0 | 4.0 | 15.8 | |
| ENSTGUG00000005008 | PECR | 0.003 | 0.000 | 24.0 | 4.0 | 30.6 | 0.00 | 0.00 | 24.0 | 2.0 | 12.9 | |
| ENSTGUG00000012033 | TSPO | 0.003 | 0.000 | 24.0 | 0.0 | 25.5 | 0.18 | 0.03 | 24.0 | 0.0 | 16.7 | |
| ENSTGUG00000013629 | TMEM45A | 0.003 | 0.000 | 24.0 | 6.0 | 19.1 | 0.03 | 0.00 | 24.0 | 0.0 | 26.6 | |
| ENSTGUG00000017326 | ALDH9A1 | 0.035 | 0.002 | 24.0 | 8.0 | 116.1 | 0.18 | 0.03 | 24.0 | 6.0 | 24.0 | |
| ENSTGUG00000013166 | APOB | 0.035 | 0.002 | 24.0 | 2.0 | 108.4 | 0.03 | 0.00 | 24.0 | 20.0 | 119.6 | |
| ENSTGUG00000002175 | DHTKD1 | 0.035 | 0.002 | 24.0 | 8.0 | 103.3 | 0.18 | 0.03 | 24.0 | 6.0 | 39.2 | |
| ENSTGUG00000003592 | GPD1-1 | 0.035 | 0.002 | 24.0 | 10.0 | 99.1 | 0.18 | 0.03 | 25.3 | 8.0 | 518.7 | |
| ENSTGUG00000003373 | PSAT1 | 0.035 | 0.002 | 24.0 | 8.0 | 85.0 | 0.03 | 0.00 | 24.0 | 4.0 | 202.8 | |
| ENSTGUG00000002850 | HNRPDL | 0.035 | 0.002 | 26.0 | 15.0 | 78.2 | 0.03 | 0.00 | 24.0 | 17.0 | 11.3 | |
| ENSTGUG00000002978 | FN1 | 0.035 | 0.002 | 24.0 | 8.0 | 71.0 | 0.03 | 0.00 | 26.0 | 2.0 | 87.1 | |
| ENSTGUG00000011436 | OAT | 0.035 | 0.002 | 24.0 | 6.0 | 64.9 | 0.00 | 0.00 | 24.0 | 6.0 | 94.1 | |
| ENSTGUG00000013083 | GCH1 | 0.035 | 0.002 | 24.0 | 4.0 | 59.6 | 0.18 | 0.03 | 25.3 | 4.0 | 165.1 | |
| ENSTGUG00000012570 | BCKDHB | 0.035 | 0.002 | 24.0 | 4.0 | 58.7 | 0.18 | 0.03 | 24.0 | 1.0 | 52.2 | |
| ENSTGUG00000012876 | B5G293_TAEGU | 0.035 | 0.002 | 26.0 | 2.0 | 57.9 | 0.00 | 0.00 | 24.0 | 2.0 | 79.6 | |
| ENSTGUG00000009993 | CPT1A | 0.035 | 0.002 | 24.0 | 18.0 | 57.5 | 0.00 | 0.00 | 24.0 | 20.0 | 113.5 | |
| ENSTGUG00000011202 | RARRES1 | 0.035 | 0.002 | 24.0 | 16.0 | 54.9 | 0.03 | 0.00 | 24.0 | 9.0 | 88.9 | |
| ENSTGUG00000003568 | PYCR1 | 0.035 | 0.002 | 24.0 | 13.0 | 47.4 | 0.03 | 0.00 | 24.0 | 6.0 | 11.3 | |
| ENSTGUG00000008808 | UGDH | 0.035 | 0.002 | 26.0 | 2.0 | 45.5 | 0.03 | 0.00 | 24.0 | 1.0 | 19.5 | |
| ENSTGUG00000017238 | C1QC | 0.035 | 0.002 | 26.0 | 15.0 | 42.8 | 0.18 | 0.03 | 24.0 | 9.0 | 13.6 | |
| ENSTGUG00000008400 | KARS | 0.035 | 0.002 | 24.0 | 18.0 | 40.2 | 0.18 | 0.03 | 25.3 | 20.7 | 42.5 | |
| ENSTGUG00000012094 | ENTPD5 | 0.035 | 0.002 | 24.0 | 5.0 | 40.1 | 0.03 | 0.00 | 25.3 | 4.0 | 23.8 | |
| ENSTGUG00000009656 |  | 0.035 | 0.002 | 24.0 | 10.0 | 38.2 | 0.03 | 0.00 | 25.3 | 4.0 | 31.5 | |
| ENSTGUG00000010684 | MPST | 0.035 | 0.002 | 24.0 | 6.0 | 37.9 | 0.03 | 0.00 | 24.0 | 2.0 | 34.2 | |
| ENSTGUG00000014010 |  | 0.035 | 0.002 | 26.0 | 15.0 | 35.2 | 0.18 | 0.03 | 24.0 | 12.0 | 5.7 | |
| ENSTGUG00000010999 | CRY1 | 0.035 | 0.002 | 24.0 | 10.0 | 34.7 | 0.03 | 0.00 | 24.0 | 10.0 | 40.3 | |
| ENSTGUG00000005505 | SURF4 | 0.035 | 0.002 | 24.0 | 6.0 | 33.2 | 0.10 | 0.01 | 28.0 | 1.0 | 42.4 | |
| ENSTGUG00000016377 |  | 0.035 | 0.002 | 24.0 | 18.0 | 29.8 | 0.18 | 0.03 | 24.0 | 1.0 | 56.8 | |
| ENSTGUG00000009552 | PPAP2B | 0.035 | 0.002 | 26.0 | 2.0 | 28.1 | 0.03 | 0.00 | 24.0 | 2.0 | 14.3 | |
| ENSTGUG00000001688 | HARSL | 0.035 | 0.002 | 24.0 | 14.0 | 27.8 | 0.03 | 0.00 | 26.0 | 5.0 | 1.4 | |
| ENSTGUG00000008555 | C11orf58 | 0.035 | 0.002 | 24.0 | 18.0 | 26.6 | 0.18 | 0.03 | 24.0 | 1.0 | 9.8 | |
| ENSTGUG00000012997 | PPM1A | 0.035 | 0.002 | 25.3 | 4.0 | 25.3 | 0.18 | 0.03 | 25.3 | 4.0 | 19.5 | |
| ENSTGUG00000002795 | TRA2A | 0.035 | 0.002 | 25.3 | 4.0 | 24.2 | 0.18 | 0.03 | 25.3 | 4.0 | 20.9 | |
| ENSTGUG00000006411 | GPLD1 | 0.035 | 0.002 | 24.0 | 6.0 | 22.9 | 0.18 | 0.03 | 24.0 | 12.0 | 13.3 | |
| ENSTGUG00000011102 |  | 0.035 | 0.002 | 25.3 | 4.0 | 21.8 | 0.00 | 0.00 | 24.0 | 6.0 | 26.4 | |
| ENSTGUG00000004988 | SCARB1 | 0.035 | 0.002 | 26.0 | 15.0 | 21.7 | 0.18 | 0.03 | 24.0 | 13.0 | 12.0 | |
| ENSTGUG00000010520 | MOBKL3 | 0.035 | 0.002 | 24.0 | 20.0 | 21.0 | 0.18 | 0.03 | 26.0 | 18.0 | 18.5 | |
| ENSTGUG00000003668 | TSPAN3 | 0.035 | 0.002 | 24.0 | 5.0 | 20.8 | 0.00 | 0.00 | 24.0 | 4.0 | 12.3 | |
| ENSTGUG00000010553 | SF3B1 | 0.035 | 0.002 | 24.0 | 0.0 | 20.7 | 0.18 | 0.03 | 25.3 | 9.3 | 24.5 | |
| ENSTGUG00000012193 | UPP2 | 0.035 | 0.002 | 24.0 | 2.0 | 20.6 | 0.03 | 0.00 | 26.0 | 2.0 | 71.6 | |
| ENSTGUG00000002012 | ENDOD1 | 0.035 | 0.002 | 26.0 | 15.0 | 20.5 | 0.18 | 0.03 | 24.0 | 8.0 | 6.6 | |
| ENSTGUG00000012759 | C14orf68 | 0.035 | 0.002 | 24.0 | 20.0 | 19.1 | 0.03 | 0.00 | 24.0 | 22.0 | 17.9 | |
| ENSTGUG00000014760 | HSPA9B | 0.035 | 0.002 | 24.0 | 10.0 | 18.3 | 0.03 | 0.00 | 24.0 | 4.0 | 48.1 | |
| ENSTGUG00000017392 | SELENBP1-2 | 0.035 | 0.002 | 24.0 | 5.0 | 17.9 | 0.18 | 0.03 | 24.0 | 2.0 | 6.5 | |
| ENSTGUG00000006500 | GNAI2 | 0.035 | 0.002 | 26.0 | 15.0 | 17.7 | 0.18 | 0.03 | 24.0 | 8.0 | 5.3 | |
| ENSTGUG00000005242 | SERINC3 | 0.035 | 0.002 | 24.0 | 5.0 | 17.5 | 0.18 | 0.03 | 24.0 | 0.0 | 32.9 | |
| ENSTGUG00000000258 | HIGD1A | 0.035 | 0.002 | 24.0 | 20.0 | 17.5 | 0.03 | 0.00 | 24.0 | 22.0 | 16.6 | |
| ENSTGUG00000012961 | TMEM126B | 0.035 | 0.002 | 24.0 | 20.0 | 16.4 | 0.03 | 0.00 | 26.0 | 18.0 | 17.1 | |
| ENSTGUG00000011111 | SFXN4 | 0.035 | 0.002 | 24.0 | 18.0 | 15.5 | 0.00 | 0.00 | 24.0 | 20.0 | 0.6 | |
| ENSTGUG00000003665 | RFNG | 0.035 | 0.002 | 26.0 | 2.0 | 15.3 | 0.03 | 0.00 | 24.0 | 8.0 | 6.1 | |
| ENSTGUG00000005075 |  | 0.035 | 0.002 | 24.0 | 2.0 | 15.2 | 0.18 | 0.03 | 24.0 | 14.0 | 56.9 | |
| ENSTGUG00000008535 | FAM20C | 0.035 | 0.002 | 26.0 | 2.0 | 14.9 | 0.18 | 0.03 | 24.0 | 0.0 | 11.5 | |
| ENSTGUG00000013712 |  | 0.035 | 0.002 | 24.0 | 6.0 | 14.4 | 0.18 | 0.03 | 24.0 | 12.0 | 12.2 | |
| ENSTGUG00000005103 | GSPT1 | 0.035 | 0.002 | 24.0 | 20.0 | 14.2 | 0.18 | 0.03 | 25.3 | 20.7 | 9.9 | |
| ENSTGUG00000002766 | TPRG1L | 0.035 | 0.002 | 24.0 | 9.0 | 12.8 | 0.18 | 0.03 | 24.0 | 2.0 | 9.0 | |
| ENSTGUG00000003332 | IREB2 | 0.035 | 0.002 | 24.0 | 20.0 | 12.6 | 0.03 | 0.00 | 24.0 | 20.0 | 14.0 | |
| ENSTGUG00000003915 | SCYE1 | 0.035 | 0.002 | 24.0 | 20.0 | 12.3 | 0.03 | 0.00 | 24.0 | 20.0 | 6.8 | |
| ENSTGUG00000009417 | SEPSECS | 0.035 | 0.002 | 24.0 | 2.0 | 12.2 | 0.18 | 0.03 | 24.0 | 14.0 | 4.6 | |
| ENSTGUG00000000237 | THAP1 | 0.035 | 0.002 | 24.0 | 8.0 | 12.2 | 0.18 | 0.03 | 24.0 | 10.0 | 13.4 | |
| ENSTGUG00000003286 | COMMD5 | 0.035 | 0.002 | 26.0 | 15.0 | 11.8 | 0.03 | 0.00 | 25.3 | 16.7 | 11.8 | |
| ENSTGUG00000001313 | CSRP1 | 0.035 | 0.002 | 26.0 | 15.0 | 11.8 | 0.18 | 0.03 | 25.3 | 8.0 | 2.1 | |
| ENSTGUG00000002711 | TMEM60 | 0.035 | 0.002 | 26.0 | 15.0 | 11.5 | 0.18 | 0.03 | 24.0 | 20.0 | 2.4 | |
| ENSTGUG00000000678 | TIMD4 | 0.035 | 0.002 | 24.0 | 9.0 | 11.0 | 0.18 | 0.03 | 24.0 | 8.0 | 4.5 | |
| ENSTGUG00000015918 | DDA1 | 0.035 | 0.002 | 24.0 | 14.0 | 10.5 | 0.18 | 0.03 | 24.0 | 22.0 | 12.6 | |
| ENSTGUG00000007310 | NGEF | 0.035 | 0.002 | 26.0 | 2.0 | 10.4 | 0.03 | 0.00 | 24.0 | 5.0 | 12.3 | |
| ENSTGUG00000007768 | COPG | 0.035 | 0.002 | 26.0 | 15.0 | 10.3 | 0.00 | 0.00 | 24.0 | 2.0 | 3.8 | |
| ENSTGUG00000016349 |  | 0.035 | 0.002 | 24.0 | 8.0 | 10.2 | 0.18 | 0.03 | 24.0 | 2.0 | 7.7 | |
| ENSTGUG00000011838 | HSF2 | 0.035 | 0.002 | 24.0 | 0.0 | 10.1 | 0.18 | 0.03 | 25.3 | 9.3 | 8.9 | |
| ENSTGUG00000001365 | PDLIM4 | 0.035 | 0.002 | 24.0 | 6.0 | 10.0 | 0.18 | 0.03 | 28.0 | 2.0 | 13.8 | |
| ENSTGUG00000004947 | DSCR3 | 0.035 | 0.002 | 24.0 | 17.0 | 9.9 | 0.03 | 0.00 | 24.0 | 1.0 | 11.5 | |
| ENSTGUG00000003098 | SH3BP4 | 0.035 | 0.002 | 24.0 | 13.0 | 9.8 | 0.10 | 0.01 | 28.0 | 1.0 | 3.5 | |
| ENSTGUG00000010325 |  | 0.035 | 0.002 | 24.0 | 12.0 | 9.8 | 0.18 | 0.03 | 24.0 | 16.0 | 0.7 | |
| ENSTGUG00000014613 |  | 0.035 | 0.002 | 26.0 | 5.0 | 9.5 | 0.18 | 0.03 | 24.0 | 5.0 | 9.3 | |
| ENSTGUG00000000730 | TAF12 | 0.035 | 0.002 | 24.0 | 14.0 | 9.0 | 0.03 | 0.00 | 24.0 | 16.0 | 4.7 | |
| ENSTGUG00000007705 | BTBD1 | 0.035 | 0.002 | 24.0 | 18.0 | 9.0 | 0.03 | 0.00 | 24.0 | 14.0 | 15.0 | |
| ENSTGUG00000004496 | AASS | 0.035 | 0.002 | 24.0 | 2.0 | 8.9 | 0.18 | 0.03 | 24.0 | 1.0 | 7.1 | |
| ENSTGUG00000007546 | TMEM165 | 0.035 | 0.002 | 24.0 | 2.0 | 8.8 | 0.18 | 0.03 | 28.0 | 16.0 | 12.8 | |
| ENSTGUG00000001126 | SYF2 | 0.035 | 0.002 | 24.0 | 14.0 | 8.8 | 0.18 | 0.03 | 24.0 | 4.0 | 4.9 | |
| ENSTGUG00000010301 | BHLHE40 | 0.035 | 0.002 | 24.0 | 2.0 | 8.7 | 0.18 | 0.03 | 26.0 | 2.0 | 10.3 | |
| ENSTGUG00000013185 | UBXN2A | 0.035 | 0.002 | 24.0 | 2.0 | 8.7 | 0.03 | 0.00 | 24.0 | 14.0 | 2.5 | |
| ENSTGUG00000007195 |  | 0.035 | 0.002 | 24.0 | 2.0 | 8.7 | 0.18 | 0.03 | 24.0 | 5.0 | 10.4 | |
| ENSTGUG00000007788 | FARSB | 0.035 | 0.002 | 24.0 | 17.0 | 8.6 | 0.03 | 0.00 | 26.0 | 18.0 | 7.0 | |
| ENSTGUG00000012503 | BID | 0.035 | 0.002 | 24.0 | 8.0 | 8.6 | 0.18 | 0.03 | 24.0 | 10.0 | 9.4 | |
| ENSTGUG00000017394 | TOR3A | 0.035 | 0.002 | 24.0 | 12.0 | 8.5 | 0.00 | 0.00 | 24.0 | 12.0 | 2.6 | |
| ENSTGUG00000010389 | EDARADD | 0.035 | 0.002 | 26.0 | 2.0 | 8.4 | 0.03 | 0.00 | 24.0 | 1.0 | 7.5 | |
| ENSTGUG00000009784 | ARL6IP5 | 0.035 | 0.002 | 26.0 | 15.0 | 8.3 | 0.03 | 0.00 | 24.0 | 10.0 | 12.1 | |
| ENSTGUG00000010190 | SFXN2 | 0.035 | 0.002 | 24.0 | 10.0 | 8.3 | 0.18 | 0.03 | 24.0 | 4.0 | 4.4 | |
| ENSTGUG00000003191 | HERC1 | 0.035 | 0.002 | 24.0 | 14.0 | 8.2 | 0.18 | 0.03 | 24.0 | 14.0 | 5.8 | |
| ENSTGUG00000017032 | ART1 | 0.035 | 0.002 | 26.0 | 15.0 | 8.2 | 0.03 | 0.00 | 24.0 | 10.0 | 1.5 | |
| ENSTGUG00000010262 | ACOX3 | 0.035 | 0.002 | 24.0 | 18.0 | 8.0 | 0.18 | 0.03 | 26.0 | 18.0 | 4.3 | |
| ENSTGUG00000003339 | NPEPPS | 0.035 | 0.002 | 24.0 | 4.0 | 8.0 | 0.03 | 0.00 | 24.0 | 9.0 | 5.9 | |
| ENSTGUG00000003335 | NR1D2 | 0.035 | 0.002 | 24.0 | 1.0 | 7.9 | 0.03 | 0.00 | 24.0 | 0.0 | 20.8 | |
| ENSTGUG00000012452 | PSMC1 | 0.035 | 0.002 | 24.0 | 18.0 | 7.8 | 0.18 | 0.03 | 26.0 | 18.0 | 12.8 | |
| ENSTGUG00000002218 | B3GNT1 | 0.035 | 0.002 | 24.0 | 2.0 | 7.6 | 0.00 | 0.00 | 24.0 | 0.0 | 2.6 | |
| ENSTGUG00000008982 | 5-Mar | 0.035 | 0.002 | 24.0 | 17.0 | 7.6 | 0.03 | 0.00 | 24.0 | 16.0 | 2.2 | |
| ENSTGUG00000009186 | ABCC2 | 0.035 | 0.002 | 24.0 | 5.0 | 7.4 | 0.03 | 0.00 | 24.0 | 22.0 | 7.3 | |
| ENSTGUG00000008105 | TMEM63B | 0.035 | 0.002 | 24.0 | 8.0 | 7.2 | 0.18 | 0.03 | 28.0 | 2.0 | 17.7 | |
| ENSTGUG00000001895 | KLHDC10 | 0.035 | 0.002 | 26.0 | 15.0 | 7.2 | 0.03 | 0.00 | 24.0 | 20.0 | 4.4 | |
| ENSTGUG00000005747 | SLC35C2 | 0.035 | 0.002 | 24.0 | 8.0 | 7.1 | 0.18 | 0.03 | 24.0 | 6.0 | 7.1 | |
| ENSTGUG00000009442 | PSMD6 | 0.035 | 0.002 | 24.0 | 18.0 | 7.1 | 0.03 | 0.00 | 24.0 | 20.0 | 8.7 | |
| ENSTGUG00000011279 | IGF1 | 0.035 | 0.002 | 24.0 | 0.0 | 6.8 | 0.18 | 0.03 | 24.0 | 22.0 | 12.2 | |
| ENSTGUG00000010968 | PLA2G4D | 0.035 | 0.002 | 24.0 | 14.0 | 6.7 | 0.03 | 0.00 | 24.0 | 17.0 | 6.6 | |
| ENSTGUG00000011296 | NUP37 | 0.035 | 0.002 | 26.0 | 15.0 | 6.4 | 0.18 | 0.03 | 24.0 | 18.0 | 0.4 | |
| ENSTGUG00000004186 | KCNT2 | 0.035 | 0.002 | 24.0 | 2.0 | 6.3 | 0.18 | 0.03 | 24.0 | 9.0 | 13.3 | |
| ENSTGUG00000011575 | INSIG2 | 0.035 | 0.002 | 24.0 | 2.0 | 6.0 | 0.18 | 0.03 | 24.0 | 13.0 | 14.7 | |
| ENSTGUG00000011319 | HTRA1 | 0.035 | 0.002 | 24.0 | 16.0 | 5.9 | 0.00 | 0.00 | 24.0 | 16.0 | 37.0 | |
| ENSTGUG00000001283 |  | 0.035 | 0.002 | 24.0 | 5.0 | 5.9 | 0.03 | 0.00 | 24.0 | 2.0 | 3.8 | |
| ENSTGUG00000009558 | TRIAP1 | 0.035 | 0.002 | 24.0 | 18.0 | 5.7 | 0.18 | 0.03 | 24.0 | 20.0 | 3.7 | |
| ENSTGUG00000014399 |  | 0.035 | 0.002 | 24.0 | 18.0 | 5.7 | 0.18 | 0.03 | 24.0 | 20.0 | 2.2 | |
| ENSTGUG00000008119 | NPEPL1 | 0.035 | 0.002 | 26.0 | 2.0 | 5.6 | 0.00 | 0.00 | 24.0 | 10.0 | 3.6 | |
| ENSTGUG00000004948 | BRI3BP | 0.035 | 0.002 | 24.0 | 2.0 | 5.3 | 0.18 | 0.03 | 24.0 | 4.0 | 4.5 | |
| ENSTGUG00000009648 | TUBB6 | 0.035 | 0.002 | 25.3 | 4.0 | 5.2 | 0.18 | 0.03 | 28.0 | 2.0 | 2.3 | |
| ENSTGUG00000005812 | C4orf43 | 0.035 | 0.002 | 25.3 | 16.7 | 5.1 | 0.03 | 0.00 | 26.0 | 18.0 | 5.1 | |
| ENSTGUG00000012999 | KIDINS220 | 0.035 | 0.002 | 26.0 | 2.0 | 4.9 | 0.18 | 0.03 | 24.0 | 2.0 | 6.2 | |
| ENSTGUG00000007930 | DDC | 0.035 | 0.002 | 24.0 | 2.0 | 4.9 | 0.03 | 0.00 | 24.0 | 22.0 | 7.7 | |
| ENSTGUG00000002687 | ERCC8 | 0.035 | 0.002 | 25.3 | 16.7 | 4.8 | 0.18 | 0.03 | 26.0 | 18.0 | 2.0 | |
| ENSTGUG00000006486 | CCDC55 | 0.035 | 0.002 | 24.0 | 20.0 | 4.7 | 0.18 | 0.03 | 24.0 | 18.0 | 1.3 | |
| ENSTGUG00000002842 | ENOPH1 | 0.035 | 0.002 | 24.0 | 12.0 | 4.6 | 0.18 | 0.03 | 24.0 | 14.0 | 2.1 | |
| ENSTGUG00000009787 | PPP4R1 | 0.035 | 0.002 | 26.0 | 2.0 | 4.5 | 0.03 | 0.00 | 24.0 | 13.0 | 3.4 | |
| ENSTGUG00000007144 | DTX4 | 0.035 | 0.002 | 24.0 | 8.0 | 4.5 | 0.03 | 0.00 | 26.0 | 5.0 | 5.6 | |
| ENSTGUG00000006677 | BCL2L1 | 0.035 | 0.002 | 26.0 | 2.0 | 4.5 | 0.03 | 0.00 | 24.0 | 1.0 | 9.2 | |
| ENSTGUG00000010722 | CRY2 | 0.035 | 0.002 | 25.3 | 4.0 | 4.5 | 0.18 | 0.03 | 24.0 | 8.0 | 1.7 | |
| ENSTGUG00000003997 | TP53BP2 | 0.035 | 0.002 | 24.0 | 6.0 | 4.5 | 0.10 | 0.01 | 28.0 | 1.0 | 4.6 | |
| ENSTGUG00000014914 |  | 0.035 | 0.002 | 24.0 | 14.0 | 4.3 | 0.18 | 0.03 | 24.0 | 2.0 | 1.8 | |
| ENSTGUG00000015629 | PDE4C | 0.035 | 0.002 | 24.0 | 6.0 | 4.3 | 0.18 | 0.03 | 24.0 | 2.0 | 2.0 | |
| ENSTGUG00000005357 | PER2 | 0.035 | 0.002 | 25.3 | 4.0 | 4.2 | 0.03 | 0.00 | 24.0 | 5.0 | 5.2 | |
| ENSTGUG00000005140 | NMNAT3 | 0.035 | 0.002 | 24.0 | 20.0 | 4.2 | 0.18 | 0.03 | 24.0 | 18.0 | 5.5 | |
| ENSTGUG00000001445 | ZMPSTE24 | 0.035 | 0.002 | 24.0 | 6.0 | 4.1 | 0.18 | 0.03 | 24.0 | 16.0 | 4.4 | |
| ENSTGUG00000005277 | RNF123 | 0.035 | 0.002 | 26.0 | 15.0 | 4.1 | 0.00 | 0.00 | 24.0 | 14.0 | 4.7 | |
| ENSTGUG00000000092 |  | 0.035 | 0.002 | 25.3 | 16.7 | 4.1 | 0.18 | 0.03 | 24.0 | 22.0 | 1.4 | |
| ENSTGUG00000005990 | GMPR | 0.035 | 0.002 | 24.0 | 18.0 | 4.0 | 0.03 | 0.00 | 24.0 | 14.0 | 3.7 | |
| ENSTGUG00000001014 | ITGB1BP3 | 0.035 | 0.002 | 24.0 | 14.0 | 3.9 | 0.00 | 0.00 | 24.0 | 8.0 | 0.4 | |
| ENSTGUG00000005969 | ZNF335 | 0.035 | 0.002 | 24.0 | 12.0 | 3.8 | 0.03 | 0.00 | 26.0 | 5.0 | 5.4 | |
| ENSTGUG00000004623 | SFRS8 | 0.035 | 0.002 | 24.0 | 20.0 | 3.7 | 0.03 | 0.00 | 26.0 | 18.0 | 3.5 | |
| ENSTGUG00000015753 | RCC1-2 | 0.035 | 0.002 | 24.0 | 12.0 | 3.7 | 0.18 | 0.03 | 24.0 | 4.0 | 1.7 | |
| ENSTGUG00000017347 | YTHDF2 | 0.035 | 0.002 | 24.0 | 6.0 | 3.7 | 0.18 | 0.03 | 28.0 | 2.0 | 9.2 | |
| ENSTGUG00000010995 | COL3A1 | 0.035 | 0.002 | 24.0 | 8.0 | 3.6 | 0.18 | 0.03 | 25.3 | 8.0 | 4.7 | |
| ENSTGUG00000016588 |  | 0.035 | 0.002 | 24.0 | 14.0 | 3.5 | 0.18 | 0.03 | 24.0 | 0.0 | 2.2 | |
| ENSTGUG00000001945 | NET1 | 0.035 | 0.002 | 24.0 | 2.0 | 3.5 | 0.03 | 0.00 | 24.0 | 2.0 | 6.3 | |
| ENSTGUG00000006761 | NUP107 | 0.035 | 0.002 | 26.0 | 15.0 | 3.4 | 0.03 | 0.00 | 24.0 | 16.0 | 1.7 | |
| ENSTGUG00000004567 | GPR107 | 0.035 | 0.002 | 25.3 | 16.7 | 3.3 | 0.03 | 0.00 | 24.0 | 22.0 | 2.2 | |
| ENSTGUG00000005130 | UTP14A | 0.035 | 0.002 | 26.0 | 15.0 | 3.3 | 0.18 | 0.03 | 24.0 | 1.0 | 1.4 | |
| ENSTGUG00000007536 | GABPB1 | 0.035 | 0.002 | 26.0 | 2.0 | 3.3 | 0.18 | 0.03 | 24.0 | 22.0 | 0.6 | |
| ENSTGUG00000011230 | PAPLN | 0.035 | 0.002 | 24.0 | 2.0 | 3.2 | 0.03 | 0.00 | 24.0 | 0.0 | 1.8 | |
| ENSTGUG00000000813 | UBXN6 | 0.035 | 0.002 | 26.0 | 15.0 | 3.2 | 0.18 | 0.03 | 24.0 | 14.0 | 0.5 | |
| ENSTGUG00000004501 | RASA4 | 0.035 | 0.002 | 26.0 | 15.0 | 3.1 | 0.00 | 0.00 | 24.0 | 18.0 | 2.0 | |
| ENSTGUG00000017178 |  | 0.035 | 0.002 | 26.0 | 2.0 | 2.9 | 0.18 | 0.03 | 28.0 | 2.0 | 3.3 | |
| ENSTGUG00000009688 |  | 0.035 | 0.002 | 24.0 | 18.0 | 2.9 | 0.18 | 0.03 | 24.0 | 20.0 | 3.3 | |
| ENSTGUG00000013595 | ABHD10 | 0.035 | 0.002 | 24.0 | 20.0 | 2.8 | 0.03 | 0.00 | 24.0 | 17.0 | 2.4 | |
| ENSTGUG00000001417 | PTTG1 | 0.035 | 0.002 | 24.0 | 16.0 | 2.8 | 0.18 | 0.03 | 24.0 | 16.0 | 4.1 | |
| ENSTGUG00000004752 | ERN1 | 0.035 | 0.002 | 25.3 | 4.0 | 2.7 | 0.10 | 0.01 | 28.0 | 1.0 | 7.4 | |
| ENSTGUG00000012628 | CCDC77 | 0.035 | 0.002 | 24.0 | 14.0 | 2.7 | 0.18 | 0.03 | 24.0 | 14.0 | 1.0 | |
| ENSTGUG00000013497 | ARL6 | 0.035 | 0.002 | 26.0 | 18.0 | 2.7 | 0.10 | 0.01 | 28.0 | 17.0 | 2.3 | |
| ENSTGUG00000013635 | C3orf26 | 0.035 | 0.002 | 24.0 | 16.0 | 2.7 | 0.18 | 0.03 | 24.0 | 22.0 | 2.5 | |
| ENSTGUG00000005982 | CCNB2 | 0.035 | 0.002 | 24.0 | 14.0 | 2.4 | 0.18 | 0.03 | 24.0 | 10.0 | 0.8 | |
| ENSTGUG00000008484 | HEATR2 | 0.035 | 0.002 | 24.0 | 22.0 | 2.3 | 0.00 | 0.00 | 24.0 | 18.0 | 6.7 | |
| ENSTGUG00000007159 | CDC20 | 0.035 | 0.002 | 24.0 | 14.0 | 2.3 | 0.18 | 0.03 | 24.0 | 13.0 | 0.4 | |
| ENSTGUG00000002410 | JUP-1 | 0.035 | 0.002 | 24.0 | 10.0 | 2.3 | 0.18 | 0.03 | 26.0 | 5.0 | 1.6 | |
| ENSTGUG00000015144 |  | 0.035 | 0.002 | 24.0 | 18.0 | 2.2 | 0.18 | 0.03 | 24.0 | 18.0 | 1.1 | |
| ENSTGUG00000009615 | SLMO1 | 0.035 | 0.002 | 24.0 | 13.0 | 2.2 | 0.18 | 0.03 | 24.0 | 22.0 | 1.6 | |
| ENSTGUG00000005633 | LMO2 | 0.035 | 0.002 | 24.0 | 14.0 | 2.2 | 0.18 | 0.03 | 26.0 | 5.0 | 1.6 | |
| ENSTGUG00000002209 | CRKRS | 0.035 | 0.002 | 24.0 | 12.0 | 2.2 | 0.10 | 0.01 | 28.0 | 3.0 | 2.2 | |
| ENSTGUG00000007666 | FSD2 | 0.035 | 0.002 | 24.0 | 20.0 | 2.2 | 0.03 | 0.00 | 24.0 | 20.0 | 7.0 | |
| ENSTGUG00000015375 | RPUSD1 | 0.035 | 0.002 | 24.0 | 10.0 | 2.1 | 0.18 | 0.03 | 24.0 | 2.0 | 0.7 | |
| ENSTGUG00000007330 | C15orf57 | 0.035 | 0.002 | 24.0 | 6.0 | 2.1 | 0.03 | 0.00 | 25.3 | 4.0 | 0.9 | |
| ENSTGUG00000003836 | GSTCD | 0.035 | 0.002 | 26.0 | 15.0 | 2.1 | 0.18 | 0.03 | 24.0 | 16.0 | 1.1 | |
| ENSTGUG00000010003 | NPAS2 | 0.035 | 0.002 | 24.0 | 14.0 | 2.1 | 0.03 | 0.00 | 26.0 | 15.0 | 1.2 | |
| ENSTGUG00000014351 | PRKDC | 0.035 | 0.002 | 24.0 | 8.0 | 2.0 | 0.03 | 0.00 | 24.0 | 13.0 | 6.7 | |
| ENSTGUG00000004664 | CXorf40A | 0.035 | 0.002 | 26.0 | 15.0 | 2.0 | 0.03 | 0.00 | 24.0 | 16.0 | 3.8 | |
| ENSTGUG00000007517 | CYLD | 0.035 | 0.002 | 24.0 | 4.0 | 1.9 | 0.18 | 0.03 | 24.0 | 1.0 | 3.7 | |
| ENSTGUG00000010938 | RPL22L1-1 | 0.035 | 0.002 | 24.0 | 17.0 | 1.8 | 0.03 | 0.00 | 24.0 | 14.0 | 0.6 | |
| ENSTGUG00000014409 |  | 0.035 | 0.002 | 24.0 | 2.0 | 1.8 | 0.00 | 0.00 | 24.0 | 8.0 | 2.2 | |
| ENSTGUG00000004896 | MFHAS1 | 0.035 | 0.002 | 24.0 | 8.0 | 1.8 | 0.18 | 0.03 | 28.0 | 2.0 | 1.4 | |
| ENSTGUG00000007824 | TTC28 | 0.035 | 0.002 | 24.0 | 9.0 | 1.7 | 0.18 | 0.03 | 24.0 | 8.0 | 4.4 | |
| ENSTGUG00000009112 | RGS9BP | 0.035 | 0.002 | 24.0 | 6.0 | 1.7 | 0.18 | 0.03 | 24.0 | 5.0 | 1.2 | |
| ENSTGUG00000010090 | TSNAX | 0.035 | 0.002 | 24.0 | 2.0 | 1.7 | 0.18 | 0.03 | 24.0 | 21.0 | 2.9 | |
| ENSTGUG00000003386 | COL6A2 | 0.035 | 0.002 | 24.0 | 10.0 | 1.7 | 0.00 | 0.00 | 24.0 | 8.0 | 1.9 | |
| ENSTGUG00000005547 | ADAMTS13-1 | 0.035 | 0.002 | 24.0 | 2.0 | 1.6 | 0.00 | 0.00 | 24.0 | 4.0 | 2.3 | |
| ENSTGUG00000002594 | ARHGAP10 | 0.035 | 0.002 | 24.0 | 5.0 | 1.6 | 0.03 | 0.00 | 24.0 | 12.0 | 1.2 | |
| ENSTGUG00000000329 | CPEB4 | 0.035 | 0.002 | 24.0 | 10.0 | 1.6 | 0.18 | 0.03 | 24.0 | 22.0 | 3.7 | |
| ENSTGUG00000013094 | CDKN3 | 0.035 | 0.002 | 24.0 | 16.0 | 1.6 | 0.18 | 0.03 | 24.0 | 13.0 | 0.2 | |
| ENSTGUG00000012040 | GTF3C6 | 0.035 | 0.002 | 24.0 | 18.0 | 1.6 | 0.03 | 0.00 | 24.0 | 21.0 | 0.9 | |
| ENSTGUG00000001563 | ZC3H12A | 0.035 | 0.002 | 24.0 | 6.0 | 1.5 | 0.18 | 0.03 | 24.0 | 2.0 | 1.8 | |
| ENSTGUG00000007004 | C12orf28 | 0.035 | 0.002 | 24.0 | 18.0 | 1.5 | 0.10 | 0.01 | 24.0 | 16.0 | 3.1 | |
| ENSTGUG00000006237 |  | 0.035 | 0.002 | 24.0 | 2.0 | 1.4 | 0.03 | 0.00 | 24.0 | 22.0 | 5.7 | |
| ENSTGUG00000010282 | TP53I11-1 | 0.035 | 0.002 | 24.0 | 14.0 | 1.4 | 0.18 | 0.03 | 24.0 | 2.0 | 0.4 | |
| ENSTGUG00000005136 | MET | 0.035 | 0.002 | 26.0 | 2.0 | 1.4 | 0.18 | 0.03 | 26.0 | 2.0 | 2.8 | |
| ENSTGUG00000004027 | PIK3R4 | 0.035 | 0.002 | 24.0 | 17.0 | 1.4 | 0.03 | 0.00 | 24.0 | 16.0 | 1.8 | |
| ENSTGUG00000017157 |  | 0.035 | 0.002 | 24.0 | 14.0 | 1.4 | 0.03 | 0.00 | 24.0 | 13.0 | 2.3 | |
| ENSTGUG00000012511 | PIBF1 | 0.035 | 0.002 | 24.0 | 22.0 | 1.4 | 0.18 | 0.03 | 24.0 | 16.0 | 1.2 | |
| ENSTGUG00000017520 | MAGI3 | 0.035 | 0.002 | 24.0 | 2.0 | 1.3 | 0.18 | 0.03 | 24.0 | 5.0 | 1.2 | |
| ENSTGUG00000002075 | PLEKHM2 | 0.035 | 0.002 | 24.0 | 10.0 | 1.3 | 0.18 | 0.03 | 25.3 | 8.0 | 1.5 | |
| ENSTGUG00000003922 |  | 0.035 | 0.002 | 24.0 | 10.0 | 1.2 | 0.03 | 0.00 | 24.0 | 8.0 | 0.6 | |
| ENSTGUG00000011337 | N6AMT2 | 0.035 | 0.002 | 24.0 | 20.0 | 1.2 | 0.03 | 0.00 | 24.0 | 0.0 | 0.4 | |
| ENSTGUG00000015720 | CPN2 | 0.035 | 0.002 | 24.0 | 12.0 | 1.2 | 0.18 | 0.03 | 24.0 | 22.0 | 0.5 | |
| ENSTGUG00000000978 | C1orf172 | 0.035 | 0.002 | 24.0 | 6.0 | 1.1 | 0.03 | 0.00 | 24.0 | 4.0 | 1.0 | |
| ENSTGUG00000004731 | SETD4 | 0.035 | 0.002 | 25.3 | 16.7 | 1.1 | 0.18 | 0.03 | 24.0 | 22.0 | 1.9 | |
| ENSTGUG00000000298 |  | 0.035 | 0.002 | 24.0 | 8.0 | 1.1 | 0.03 | 0.00 | 24.0 | 8.0 | 0.6 | |
| ENSTGUG00000010713 | PVALB-2 | 0.035 | 0.002 | 26.0 | 15.0 | 1.1 | 0.18 | 0.03 | 24.0 | 13.0 | 0.3 | |
| ENSTGUG00000004738 | SRF | 0.035 | 0.002 | 24.0 | 14.0 | 1.1 | 0.18 | 0.03 | 24.0 | 2.0 | 1.4 | |
| ENSTGUG00000016365 |  | 0.035 | 0.002 | 24.0 | 4.0 | 1.1 | 0.03 | 0.00 | 24.0 | 0.0 | 0.9 | |
| ENSTGUG00000005251 | TCTN2 | 0.035 | 0.002 | 24.0 | 14.0 | 1.1 | 0.03 | 0.00 | 24.0 | 12.0 | 1.1 | |
| ENSTGUG00000004134 | CCDC109B | 0.035 | 0.002 | 25.3 | 16.7 | 1.1 | 0.03 | 0.00 | 26.0 | 5.0 | 0.7 | |
| ENSTGUG00000010458 | MTR | 0.035 | 0.002 | 24.0 | 13.0 | 1.1 | 0.18 | 0.03 | 28.0 | 16.0 | 2.0 | |
| ENSTGUG00000002443 | MACROD2 | 0.035 | 0.002 | 26.0 | 2.0 | 1.1 | 0.18 | 0.03 | 24.0 | 0.0 | 0.8 | |
| ENSTGUG00000011321 | IFT88 | 0.035 | 0.002 | 25.3 | 16.7 | 1.1 | 0.00 | 0.00 | 24.0 | 16.0 | 2.2 | |
| ENSTGUG00000006626 | ABR | 0.035 | 0.002 | 24.0 | 13.0 | 1.1 | 0.00 | 0.00 | 24.0 | 12.0 | 1.7 | |
| ENSTGUG00000000264 |  | 0.035 | 0.002 | 24.0 | 13.0 | 1.0 | 0.03 | 0.00 | 24.0 | 14.0 | 1.5 | |
| ENSTGUG00000011075 | GANC | 0.035 | 0.002 | 24.0 | 9.0 | 1.0 | 0.03 | 0.00 | 24.0 | 14.0 | 2.3 | |
| ENSTGUG00000004783 | RPGRIP1L | 0.035 | 0.002 | 24.0 | 20.0 | 1.0 | 0.03 | 0.00 | 25.3 | 16.7 | 3.0 | |
| ENSTGUG00000007337 | MMP2 | 0.035 | 0.002 | 25.3 | 4.0 | 1.0 | 0.03 | 0.00 | 24.0 | 10.0 | 4.6 | |
| ENSTGUG00000010181 |  | 0.035 | 0.002 | 24.0 | 5.0 | 1.0 | 0.18 | 0.03 | 24.0 | 5.0 | 2.3 | |
| ENSTGUG00000017102 | LMBR1L | 0.035 | 0.002 | 25.3 | 4.0 | 1.0 | 0.10 | 0.01 | 24.0 | 0.0 | 0.4 | |
| ENSTGUG00000011459 | NCOA2 | 0.035 | 0.002 | 24.0 | 2.0 | 0.9 | 0.18 | 0.03 | 25.3 | 9.3 | 1.2 | |
| ENSTGUG00000011101 | ITGAV | 0.035 | 0.002 | 25.3 | 4.0 | 0.9 | 0.18 | 0.03 | 24.0 | 5.0 | 1.0 | |
| ENSTGUG00000010119 | MIER1 | 0.035 | 0.002 | 24.0 | 6.0 | 0.9 | 0.03 | 0.00 | 24.0 | 8.0 | 2.2 | |
| ENSTGUG00000014829 |  | 0.035 | 0.002 | 24.0 | 12.0 | 0.9 | 0.18 | 0.03 | 24.0 | 8.0 | 0.2 | |
| ENSTGUG00000002074 |  | 0.035 | 0.002 | 24.0 | 20.0 | 0.9 | 0.00 | 0.00 | 24.0 | 8.0 | 1.4 | |
| ENSTGUG00000001612 | BRPF3 | 0.035 | 0.002 | 24.0 | 10.0 | 0.9 | 0.03 | 0.00 | 24.0 | 12.0 | 1.5 | |
| ENSTGUG00000014344 |  | 0.035 | 0.002 | 24.0 | 5.0 | 0.9 | 0.10 | 0.01 | 24.0 | 11.0 | 0.9 | |
| ENSTGUG00000000940 | RCC1-1 | 0.035 | 0.002 | 24.0 | 10.0 | 0.9 | 0.18 | 0.03 | 24.0 | 1.0 | 0.6 | |
| ENSTGUG00000008063 | WDR51B | 0.035 | 0.002 | 24.0 | 13.0 | 0.9 | 0.18 | 0.03 | 24.0 | 14.0 | 0.3 | |
| ENSTGUG00000008296 | CCDC127 | 0.035 | 0.002 | 24.0 | 6.0 | 0.9 | 0.03 | 0.00 | 24.0 | 8.0 | 1.1 | |
| ENSTGUG00000005738 | MMRN2 | 0.035 | 0.002 | 24.0 | 6.0 | 0.8 | 0.03 | 0.00 | 26.0 | 5.0 | 1.1 | |
| ENSTGUG00000007179 | IGFBP3 | 0.035 | 0.002 | 24.0 | 0.0 | 0.8 | 0.18 | 0.03 | 24.0 | 13.0 | 0.3 | |
| ENSTGUG00000011847 | GJA1 | 0.035 | 0.002 | 26.0 | 2.0 | 0.8 | 0.00 | 0.00 | 24.0 | 6.0 | 1.0 | |
| ENSTGUG00000009110 | TTC30B | 0.035 | 0.002 | 24.0 | 16.0 | 0.8 | 0.18 | 0.03 | 24.0 | 20.0 | 0.8 | |
| ENSTGUG00000013390 | CASQ2 | 0.035 | 0.002 | 26.0 | 15.0 | 0.7 | 0.03 | 0.00 | 24.0 | 16.0 | 0.5 | |
| ENSTGUG00000001558 | SGCE | 0.035 | 0.002 | 24.0 | 2.0 | 0.7 | 0.03 | 0.00 | 24.0 | 6.0 | 0.8 | |
| ENSTGUG00000001885 | SLC4A1-2 | 0.035 | 0.002 | 24.0 | 12.0 | 0.7 | 0.18 | 0.03 | 24.0 | 8.0 | 0.2 | |
| ENSTGUG00000010720 | SDPR | 0.035 | 0.002 | 24.0 | 2.0 | 0.7 | 0.18 | 0.03 | 24.0 | 4.0 | 0.9 | |
| ENSTGUG00000013136 | WDR35 | 0.035 | 0.002 | 24.0 | 18.0 | 0.7 | 0.03 | 0.00 | 24.0 | 16.0 | 1.0 | |
| ENSTGUG00000006098 | BOK | 0.035 | 0.002 | 24.0 | 14.0 | 0.7 | 0.02 | 0.00 | 24.0 | 12.0 | 0.3 | |
| ENSTGUG00000001503 |  | 0.035 | 0.002 | 24.0 | 13.0 | 0.7 | 0.02 | 0.00 | 24.0 | 14.0 | 0.2 | |
| ENSTGUG00000000700 | KLF2 | 0.035 | 0.002 | 25.3 | 16.7 | 0.6 | 0.18 | 0.03 | 24.0 | 2.0 | 0.8 | |
| ENSTGUG00000003043 | MRC2 | 0.035 | 0.002 | 24.0 | 9.0 | 0.6 | 0.18 | 0.03 | 24.0 | 6.0 | 0.8 | |
| ENSTGUG00000009040 | WDR6 | 0.035 | 0.002 | 24.0 | 13.0 | 0.6 | 0.03 | 0.00 | 24.0 | 10.0 | 0.5 | |
| ENSTGUG00000006059 | ETV5 | 0.035 | 0.002 | 24.0 | 12.0 | 0.6 | 0.18 | 0.03 | 24.0 | 12.0 | 0.2 | |
| ENSTGUG00000013281 | TRAM2 | 0.035 | 0.002 | 24.0 | 12.0 | 0.6 | 0.03 | 0.00 | 24.0 | 8.0 | 1.1 | |
| ENSTGUG00000013464 | SFRS15 | 0.035 | 0.002 | 24.0 | 5.0 | 0.6 | 0.03 | 0.00 | 24.0 | 1.0 | 0.8 | |
| ENSTGUG00000002147 | SLC8A1 | 0.035 | 0.002 | 24.0 | 4.0 | 0.5 | 0.18 | 0.03 | 24.0 | 6.0 | 0.4 | |
| ENSTGUG00000009234 | MGP | 0.035 | 0.002 | 25.3 | 16.7 | 0.5 | 0.03 | 0.00 | 25.3 | 4.0 | 0.8 | |
| ENSTGUG00000014685 | IFT172 | 0.035 | 0.002 | 25.3 | 4.0 | 0.5 | 0.03 | 0.00 | 24.0 | 10.0 | 0.1 | |
| ENSTGUG00000012791 | SCRIB | 0.035 | 0.002 | 24.0 | 8.0 | 0.5 | 0.18 | 0.03 | 24.0 | 4.0 | 0.9 | |
| ENSTGUG00000014388 | PIWIL2 | 0.035 | 0.002 | 24.0 | 12.0 | 0.5 | 0.18 | 0.03 | 24.0 | 2.0 | 0.3 | |
| ENSTGUG00000012217 | FOS | 0.035 | 0.002 | 24.0 | 14.0 | 0.5 | 0.18 | 0.03 | 28.0 | 2.0 | 0.5 | |
| ENSTGUG00000003195 | IFT140 | 0.035 | 0.002 | 24.0 | 16.0 | 0.5 | 0.18 | 0.03 | 24.0 | 16.0 | 0.5 | |
| ENSTGUG00000004465 |  | 0.035 | 0.002 | 24.0 | 10.0 | 0.5 | 0.18 | 0.03 | 24.0 | 13.0 | 1.0 | |
| ENSTGUG00000001478 | PEX1 | 0.035 | 0.002 | 24.0 | 18.0 | 0.5 | 0.03 | 0.00 | 24.0 | 20.0 | 2.6 | |
| ENSTGUG00000000390 | USP2 | 0.035 | 0.002 | 24.0 | 10.0 | 0.4 | 0.18 | 0.03 | 24.0 | 8.0 | 0.4 | |
| ENSTGUG00000006326 | KIF1A | 0.035 | 0.002 | 24.0 | 12.0 | 0.4 | 0.03 | 0.00 | 24.0 | 10.0 | 0.4 | |
| ENSTGUG00000010953 | SKIL | 0.035 | 0.002 | 24.0 | 6.0 | 0.4 | 0.18 | 0.03 | 24.0 | 10.0 | 0.0 | |
| ENSTGUG00000017443 | USP49 | 0.035 | 0.002 | 24.0 | 16.0 | 0.4 | 0.18 | 0.03 | 24.0 | 0.0 | 1.0 | |
| ENSTGUG00000004590 | RPRD2 | 0.035 | 0.002 | 24.0 | 10.0 | 0.4 | 0.18 | 0.03 | 26.0 | 2.0 | 2.6 | |
| ENSTGUG00000010096 | DISC1 | 0.035 | 0.002 | 24.0 | 6.0 | 0.4 | 0.03 | 0.00 | 24.0 | 10.0 | 1.1 | |
| ENSTGUG00000014266 | GLCE | 0.035 | 0.002 | 24.0 | 2.0 | 0.3 | 0.18 | 0.03 | 24.0 | 1.0 | 1.4 | |
| ENSTGUG00000015115 |  | 0.035 | 0.002 | 25.3 | 16.7 | 0.3 | 0.18 | 0.03 | 24.0 | 0.0 | 0.2 | |
| ENSTGUG00000003678 | CELSR1 | 0.035 | 0.002 | 24.0 | 6.0 | 0.3 | 0.18 | 0.03 | 24.0 | 22.0 | 0.4 | |
| ENSTGUG00000014158 |  | 0.035 | 0.002 | 24.0 | 10.0 | 0.3 | 0.18 | 0.03 | 24.0 | 13.0 | 0.7 | |
| ENSTGUG00000000061 | LIPG | 0.035 | 0.002 | 26.0 | 15.0 | 0.3 | 0.03 | 0.00 | 24.0 | 0.0 | 0.3 | |
| ENSTGUG00000011144 |  | 0.035 | 0.002 | 24.0 | 21.0 | 0.3 | 0.00 | 0.00 | 24.0 | 12.0 | 0.1 | |
| ENSTGUG00000004976 | KIAA2026-2 | 0.035 | 0.002 | 24.0 | 8.0 | 0.3 | 0.18 | 0.03 | 24.0 | 22.0 | 0.1 | |
| ENSTGUG00000007431 | MBTPS2 | 0.035 | 0.002 | 25.3 | 16.7 | 0.3 | 0.18 | 0.03 | 24.0 | 22.0 | 0.4 | |
| ENSTGUG00000009506 | GUCY2C | 0.035 | 0.002 | 24.0 | 14.0 | 0.2 | 0.18 | 0.03 | 24.0 | 13.0 | 0.3 | |
| ENSTGUG00000014077 |  | 0.035 | 0.002 | 24.0 | 12.0 | 0.2 | 0.18 | 0.03 | 24.0 | 10.0 | 0.2 | |
| ENSTGUG00000007977 | CA4 | 0.035 | 0.002 | 24.0 | 2.0 | 0.2 | 0.10 | 0.01 | 24.0 | 9.0 | 0.6 | |
| ENSTGUG00000009167 | KCNQ1 | 0.035 | 0.002 | 24.0 | 8.0 | 0.2 | 0.03 | 0.00 | 25.3 | 4.0 | 0.5 | |
| ENSTGUG00000015557 | DYSF | 0.035 | 0.002 | 24.0 | 8.0 | 0.2 | 0.18 | 0.03 | 25.3 | 9.3 | 0.5 | |
| ENSTGUG00000003743 | SPSB1 | 0.035 | 0.002 | 24.0 | 6.0 | 0.2 | 0.18 | 0.03 | 28.0 | 2.0 | 0.8 | |
| ENSTGUG00000015074 | ARAP3 | 0.035 | 0.002 | 24.0 | 9.0 | 0.2 | 0.03 | 0.00 | 24.0 | 2.0 | 0.4 | |
| ENSTGUG00000000191 | CEP164 | 0.035 | 0.002 | 24.0 | 13.0 | 0.2 | 0.03 | 0.00 | 24.0 | 1.0 | 0.1 | |
| ENSTGUG00000014986 | EIF2C3 | 0.035 | 0.002 | 24.0 | 13.0 | 0.1 | 0.18 | 0.03 | 24.0 | 9.0 | 0.1 | |
| ENSTGUG00000013278 | EFHC1 | 0.035 | 0.002 | 25.3 | 16.7 | 0.1 | 0.18 | 0.03 | 24.0 | 13.0 | 0.0 | |
| ENSTGUG00000010025 | JAKMIP1 | 0.035 | 0.002 | 26.0 | 18.0 | 0.0 | 0.18 | 0.03 | 24.0 | 14.0 | 0.2 | |
| ENSTGUG00000006881 | CACNA1H | 0.035 | 0.002 | 24.0 | 6.0 | 0.0 | 0.18 | 0.03 | 24.0 | 4.0 | 0.0 | |
| ENSTGUG00000010452 | EIF4A2 | 0.130 | 0.009 | 28.0 | 1.0 | 447.1 | 0.18 | 0.03 | 24.0 | 10.0 | 197.3 | |
| ENSTGUG00000006924 | CYP4V2 | 0.130 | 0.009 | 28.0 | 1.0 | 232.9 | 0.18 | 0.03 | 24.0 | 6.0 | 29.7 | |
| ENSTGUG00000001663 | PSMB2 | 0.130 | 0.009 | 28.0 | 15.0 | 68.5 | 0.18 | 0.03 | 24.0 | 21.0 | 44.7 | |
| ENSTGUG00000009881 | IL1R1 | 0.130 | 0.009 | 28.0 | 1.0 | 50.6 | 0.18 | 0.03 | 24.0 | 0.0 | 35.7 | |
| ENSTGUG00000012591 | ADIPOR2 | 0.130 | 0.009 | 28.0 | 1.0 | 45.5 | 0.00 | 0.00 | 24.0 | 6.0 | 23.8 | |
| ENSTGUG00000016409 |  | 0.130 | 0.009 | 28.0 | 15.0 | 30.5 | 0.18 | 0.03 | 24.0 | 22.0 | 23.7 | |
| ENSTGUG00000000884 | CIRBP | 0.130 | 0.009 | 28.0 | 15.0 | 29.2 | 0.18 | 0.03 | 24.0 | 20.0 | 77.4 | |
| ENSTGUG00000012010 | MRPS31 | 0.130 | 0.009 | 28.0 | 15.0 | 23.9 | 0.03 | 0.00 | 24.0 | 16.0 | 18.6 | |
| ENSTGUG00000004034 | UBL7 | 0.130 | 0.009 | 28.0 | 15.0 | 19.9 | 0.03 | 0.00 | 24.0 | 22.0 | 4.5 | |
| ENSTGUG00000011810 | DARS | 0.130 | 0.009 | 28.0 | 15.0 | 19.2 | 0.18 | 0.03 | 24.0 | 20.0 | 7.0 | |
| ENSTGUG00000012861 | CKB | 0.130 | 0.009 | 28.0 | 15.0 | 16.3 | 0.03 | 0.00 | 24.0 | 9.0 | 4.6 | |
| ENSTGUG00000006942 | NSFL1C | 0.130 | 0.009 | 28.0 | 15.0 | 15.3 | 0.18 | 0.03 | 24.0 | 0.0 | 2.4 | |
| ENSTGUG00000014326 |  | 0.130 | 0.009 | 28.0 | 1.0 | 15.0 | 0.18 | 0.03 | 24.0 | 8.0 | 8.3 | |
| ENSTGUG00000003338 | MCTS1 | 0.130 | 0.009 | 28.0 | 15.0 | 14.2 | 0.18 | 0.03 | 24.0 | 16.0 | 12.0 | |
| ENSTGUG00000002784 | BECN1 | 0.130 | 0.009 | 28.0 | 15.0 | 10.9 | 0.18 | 0.03 | 25.3 | 20.7 | 10.2 | |
| ENSTGUG00000005542 | C16orf63 | 0.130 | 0.009 | 28.0 | 15.0 | 10.7 | 0.03 | 0.00 | 24.0 | 16.0 | 3.4 | |
| ENSTGUG00000017963 |  | 0.130 | 0.009 | 24.0 | 0.0 | 10.3 | 0.18 | 0.03 | 24.0 | 12.0 | 10.9 | |
| ENSTGUG00000000003 | EGR1 | 0.130 | 0.009 | 28.0 | 15.0 | 9.6 | 0.18 | 0.03 | 24.0 | 21.0 | 9.0 | |
| ENSTGUG00000007210 |  | 0.130 | 0.009 | 28.0 | 1.0 | 7.4 | 0.18 | 0.03 | 24.0 | 6.0 | 4.1 | |
| ENSTGUG00000003705 | 12-Sep | 0.130 | 0.009 | 28.0 | 15.0 | 7.0 | 0.18 | 0.03 | 24.0 | 8.0 | 1.5 | |
| ENSTGUG00000007249 | RAB21 | 0.130 | 0.009 | 28.0 | 1.0 | 6.6 | 0.03 | 0.00 | 24.0 | 14.0 | 10.0 | |
| ENSTGUG00000013378 | LRRC58 | 0.130 | 0.009 | 28.0 | 1.0 | 5.6 | 0.10 | 0.01 | 28.0 | 3.0 | 12.6 | |
| ENSTGUG00000007969 | SLC35B2 | 0.130 | 0.009 | 28.0 | 15.0 | 5.3 | 0.03 | 0.00 | 24.0 | 1.0 | 1.4 | |
| ENSTGUG00000007861 | SCFD2-2 | 0.130 | 0.009 | 28.0 | 1.0 | 4.8 | 0.03 | 0.00 | 24.0 | 5.0 | 5.9 | |
| ENSTGUG00000007061 | PRPSAP2 | 0.130 | 0.009 | 28.0 | 1.0 | 4.0 | 0.18 | 0.03 | 24.0 | 12.0 | 1.8 | |
| ENSTGUG00000006515 | B5G1J6_TAEGU | 0.130 | 0.009 | 28.0 | 15.0 | 3.8 | 0.18 | 0.03 | 24.0 | 14.0 | 3.1 | |
| ENSTGUG00000010228 | C10orf32 | 0.130 | 0.009 | 28.0 | 15.0 | 3.5 | 0.18 | 0.03 | 26.0 | 15.0 | 4.3 | |
| ENSTGUG00000010273 | PCGF6 | 0.130 | 0.009 | 28.0 | 1.0 | 3.3 | 0.18 | 0.03 | 24.0 | 1.0 | 0.9 | |
| ENSTGUG00000004726 | TMEM177 | 0.130 | 0.009 | 28.0 | 15.0 | 3.2 | 0.18 | 0.03 | 24.0 | 20.0 | 2.1 | |
| ENSTGUG00000013844 | SKAP1 | 0.130 | 0.009 | 28.0 | 15.0 | 3.1 | 0.03 | 0.00 | 24.0 | 14.0 | 1.3 | |
| ENSTGUG00000007406 | AKTIP | 0.130 | 0.009 | 28.0 | 17.0 | 2.6 | 0.00 | 0.00 | 24.0 | 18.0 | 2.5 | |
| ENSTGUG00000007397 | RAD51C | 0.130 | 0.009 | 28.0 | 15.0 | 2.6 | 0.18 | 0.03 | 24.0 | 4.0 | 0.7 | |
| ENSTGUG00000005592 | CD59 | 0.130 | 0.009 | 28.0 | 1.0 | 2.2 | 0.03 | 0.00 | 24.0 | 12.0 | 1.7 | |
| ENSTGUG00000004179 | ADPGK | 0.130 | 0.009 | 28.0 | 15.0 | 1.9 | 0.00 | 0.00 | 24.0 | 8.0 | 0.9 | |
| ENSTGUG00000002093 | GEMIN6 | 0.130 | 0.009 | 28.0 | 15.0 | 1.8 | 0.03 | 0.00 | 24.0 | 13.0 | 2.5 | |
| ENSTGUG00000004816 | ELP4 | 0.130 | 0.009 | 28.0 | 15.0 | 1.3 | 0.18 | 0.03 | 24.0 | 21.0 | 3.9 | |
| ENSTGUG00000000827 | FAM118B | 0.130 | 0.009 | 28.0 | 15.0 | 1.2 | 0.03 | 0.00 | 24.0 | 17.0 | 0.8 | |
| ENSTGUG00000010703 | IL3RA-2 | 0.130 | 0.009 | 28.0 | 15.0 | 1.1 | 0.18 | 0.03 | 24.0 | 21.0 | 1.1 | |
| ENSTGUG00000012350 | ADCK1 | 0.130 | 0.009 | 28.0 | 15.0 | 0.9 | 0.03 | 0.00 | 24.0 | 20.0 | 0.6 | |
| ENSTGUG00000015887 |  | 0.130 | 0.009 | 28.0 | 1.0 | 0.7 | 0.18 | 0.03 | 24.0 | 2.0 | 0.2 | |
| ENSTGUG00000012028 | KIAA1919 | 0.130 | 0.009 | 24.0 | 9.0 | 0.6 | 0.03 | 0.00 | 24.0 | 22.0 | 0.6 | |
| ENSTGUG00000015511 |  | 0.130 | 0.009 | 28.0 | 1.0 | 0.6 | 0.03 | 0.00 | 24.0 | 9.0 | 0.6 | |
| ENSTGUG00000000662 | JAM3 | 0.130 | 0.009 | 28.0 | 1.0 | 0.5 | 0.00 | 0.00 | 24.0 | 10.0 | 1.3 | |
| ENSTGUG00000016627 |  | 0.130 | 0.009 | 24.0 | 7.0 | 0.5 | 0.03 | 0.00 | 24.0 | 10.0 | 0.6 | |
| ENSTGUG00000002451 | HSPB3 | 0.130 | 0.009 | 24.0 | 2.0 | 0.5 | 0.18 | 0.03 | 24.0 | 13.0 | 0.6 | |
| ENSTGUG00000007448 | CEP135 | 0.130 | 0.009 | 28.0 | 15.0 | 0.4 | 0.00 | 0.00 | 24.0 | 18.0 | 0.2 | |
| ENSTGUG00000017436 | PGC | 0.130 | 0.009 | 24.0 | 10.0 | 0.3 | 0.18 | 0.03 | 28.0 | 0.0 | 1.0 | |
| ENSTGUG00000007060 | ST6GALNAC3 | 0.130 | 0.009 | 24.0 | 5.0 | 0.2 | 0.18 | 0.03 | 24.0 | 21.0 | 0.3 | |
| ENSTGUG00000014323 |  | 0.130 | 0.009 | 24.0 | 4.0 | 0.2 | 0.18 | 0.03 | 24.0 | 10.0 | 0.2 | |
| ENSTGUG00000016456 |  | 0.130 | 0.009 | 24.0 | 6.0 | 0.2 | 0.00 | 0.00 | 24.0 | 0.0 | 0.7 | |
| ENSTGUG00000000157 | KIF9 | 0.130 | 0.009 | 28.0 | 15.0 | 0.1 | 0.18 | 0.03 | 24.0 | 18.0 | 0.2 | |
| ENSTGUG00000013598 | PLCXD2 | 0.130 | 0.009 | 24.0 | 4.0 | 0.1 | 0.18 | 0.03 | 24.0 | 9.0 | 0.5 | |
| ENSTGUG00000011100 | PLAG1 | 0.130 | 0.009 | 24.0 | 0.0 | 0.1 | 0.18 | 0.03 | 24.0 | 5.0 | 0.3 | |
| ENSTGUG00000001367 | ACBD7 | 0.130 | 0.009 | 24.0 | 6.0 | 0.1 | 0.18 | 0.03 | 24.0 | 17.0 | 0.3 | |
| ENSTGUG00000004435 | ADAMTSL1-2 | 0.130 | 0.009 | 24.0 | 17.0 | 0.1 | 0.03 | 0.00 | 24.0 | 14.0 | 0.2 | |
| ENSTGUG00000006544 | VIT2 | 0.130 | 0.009 | 24.0 | 16.0 | 0.0 | 0.10 | 0.01 | 24.0 | 14.0 | 0.0 | |
| ENSTGUG00000010921 | C8orf22 | 0.234 | 0.032 | 25.3 | 9.3 | 1026.8 | 0.18 | 0.03 | 24.0 | 0.0 | 422.8 | |
| ENSTGUG00000002447 | ACLY | 0.234 | 0.032 | 24.0 | 21.0 | 703.6 | 0.03 | 0.00 | 24.0 | 4.0 | 909.4 | |
| ENSTGUG00000007597 | GATM | 0.234 | 0.032 | 24.0 | 6.0 | 598.0 | 0.10 | 0.01 | 28.0 | 15.0 | 416.7 | |
| ENSTGUG00000008374 | TAT | 0.234 | 0.032 | 24.0 | 9.0 | 590.0 | 0.00 | 0.00 | 24.0 | 4.0 | 1950.2 | |
| ENSTGUG00000003698 | IVNS1ABP | 0.234 | 0.032 | 24.0 | 22.0 | 575.6 | 0.03 | 0.00 | 24.0 | 20.0 | 546.1 | |
| ENSTGUG00000003933 | EPAS1 | 0.234 | 0.032 | 26.0 | 2.0 | 330.1 | 0.18 | 0.03 | 24.0 | 2.0 | 183.7 | |
| ENSTGUG00000010441 | FETUB | 0.234 | 0.032 | 25.3 | 4.0 | 300.2 | 0.18 | 0.03 | 25.3 | 4.0 | 285.7 | |
| ENSTGUG00000007393 | PAICS | 0.234 | 0.032 | 24.0 | 6.0 | 282.7 | 0.00 | 0.00 | 24.0 | 4.0 | 509.9 | |
| ENSTGUG00000005973 | GOT2 | 0.234 | 0.032 | 24.0 | 9.0 | 212.3 | 0.18 | 0.03 | 24.0 | 4.0 | 242.7 | |
| ENSTGUG00000008930 | LDHA | 0.234 | 0.032 | 24.0 | 4.0 | 191.4 | 0.18 | 0.03 | 24.0 | 10.0 | 734.9 | |
| ENSTGUG00000015865 | SHMT2 | 0.234 | 0.032 | 25.3 | 12.7 | 172.6 | 0.03 | 0.00 | 26.0 | 5.0 | 44.9 | |
| ENSTGUG00000009087 |  | 0.234 | 0.032 | 24.0 | 21.0 | 162.6 | 0.00 | 0.00 | 24.0 | 0.0 | 46.3 | |
| ENSTGUG00000006154 | SLC38A4 | 0.234 | 0.032 | 25.3 | 9.3 | 161.2 | 0.18 | 0.03 | 25.3 | 9.3 | 297.2 | |
| ENSTGUG00000005650 | ITIH1 | 0.234 | 0.032 | 24.0 | 2.0 | 160.0 | 0.18 | 0.03 | 26.0 | 2.0 | 303.3 | |
| ENSTGUG00000000472 | GART | 0.234 | 0.032 | 25.3 | 4.0 | 92.7 | 0.03 | 0.00 | 24.0 | 4.0 | 179.8 | |
| ENSTGUG00000006842 | OSGIN1 | 0.234 | 0.032 | 25.3 | 9.3 | 91.9 | 0.03 | 0.00 | 25.3 | 16.7 | 65.6 | |
| ENSTGUG00000006167 | MAT1A | 0.234 | 0.032 | 24.0 | 4.0 | 90.3 | 0.03 | 0.00 | 24.0 | 1.0 | 297.5 | |
| ENSTGUG00000008524 | CYP4B1 | 0.234 | 0.032 | 25.3 | 8.0 | 85.1 | 0.03 | 0.00 | 24.0 | 12.0 | 15.6 | |
| ENSTGUG00000000363 | ACSBG2 | 0.234 | 0.032 | 24.0 | 4.0 | 81.1 | 0.00 | 0.00 | 24.0 | 8.0 | 200.1 | |
| ENSTGUG00000003777 | SLC2A7 | 0.234 | 0.032 | 25.3 | 8.0 | 68.7 | 0.03 | 0.00 | 24.0 | 9.0 | 64.5 | |
| ENSTGUG00000009032 | SPG21 | 0.234 | 0.032 | 24.0 | 9.0 | 63.8 | 0.03 | 0.00 | 24.0 | 13.0 | 98.5 | |
| ENSTGUG00000006153 | AQP9 | 0.234 | 0.032 | 24.0 | 9.0 | 58.6 | 0.18 | 0.03 | 24.0 | 5.0 | 61.7 | |
| ENSTGUG00000000528 | C21orf55 | 0.234 | 0.032 | 25.3 | 12.7 | 54.0 | 0.18 | 0.03 | 25.3 | 12.7 | 65.0 | |
| ENSTGUG00000000341 | SFXN1 | 0.234 | 0.032 | 26.0 | 15.0 | 48.2 | 0.18 | 0.03 | 24.0 | 12.0 | 29.6 | |
| ENSTGUG00000004786 | CTNNB1 | 0.234 | 0.032 | 26.0 | 2.0 | 46.8 | 0.00 | 0.00 | 24.0 | 2.0 | 19.7 | |
| ENSTGUG00000013059 | TSKU | 0.234 | 0.032 | 24.0 | 8.0 | 46.6 | 0.18 | 0.03 | 24.0 | 8.0 | 6.1 | |
| ENSTGUG00000003676 | ABCG5 | 0.234 | 0.032 | 25.3 | 9.3 | 44.7 | 0.18 | 0.03 | 24.0 | 0.0 | 13.5 | |
| ENSTGUG00000016642 |  | 0.234 | 0.032 | 24.0 | 2.0 | 44.6 | 0.18 | 0.03 | 24.0 | 2.0 | 16.4 | |
| ENSTGUG00000008694 | UBE2L3 | 0.234 | 0.032 | 24.0 | 21.0 | 42.6 | 0.18 | 0.03 | 24.0 | 20.0 | 31.0 | |
| ENSTGUG00000008978 | MTX2 | 0.234 | 0.032 | 24.0 | 18.0 | 41.5 | 0.18 | 0.03 | 24.0 | 14.0 | 30.2 | |
| ENSTGUG00000004985 | ABHD12 | 0.234 | 0.032 | 25.3 | 12.7 | 41.0 | 0.03 | 0.00 | 24.0 | 13.0 | 23.1 | |
| ENSTGUG00000003730 | BDH2 | 0.234 | 0.032 | 24.0 | 0.0 | 36.8 | 0.18 | 0.03 | 24.0 | 0.0 | 7.3 | |
| ENSTGUG00000002555 | NSBP1 | 0.234 | 0.032 | 24.0 | 21.0 | 36.1 | 0.10 | 0.01 | 28.0 | 17.0 | 9.6 | |
| ENSTGUG00000015311 | FKBP11 | 0.234 | 0.032 | 25.3 | 12.7 | 35.8 | 0.18 | 0.03 | 24.0 | 10.0 | 1.0 | |
| ENSTGUG00000008817 | DNAJA2 | 0.234 | 0.032 | 24.0 | 17.0 | 35.5 | 0.03 | 0.00 | 24.0 | 14.0 | 60.1 | |
| ENSTGUG00000014947 | ARF5 | 0.234 | 0.032 | 24.0 | 14.0 | 34.9 | 0.03 | 0.00 | 24.0 | 1.0 | 19.4 | |
| ENSTGUG00000010226 | TOMM20 | 0.234 | 0.032 | 24.0 | 18.0 | 33.8 | 0.18 | 0.03 | 24.0 | 20.0 | 13.5 | |
| ENSTGUG00000006197 | AMIGO2 | 0.234 | 0.032 | 24.0 | 14.0 | 31.2 | 0.00 | 0.00 | 24.0 | 14.0 | 10.2 | |
| ENSTGUG00000003420 | PSMA4-1 | 0.234 | 0.032 | 25.3 | 16.7 | 30.1 | 0.18 | 0.03 | 24.0 | 20.0 | 9.6 | |
| ENSTGUG00000002920 | MOSPD1 | 0.234 | 0.032 | 24.0 | 21.0 | 27.8 | 0.18 | 0.03 | 24.0 | 20.0 | 6.8 | |
| ENSTGUG00000007213 | EPS8L2 | 0.234 | 0.032 | 25.3 | 9.3 | 26.3 | 0.03 | 0.00 | 24.0 | 5.0 | 7.4 | |
| ENSTGUG00000006493 |  | 0.234 | 0.032 | 24.0 | 18.0 | 25.9 | 0.18 | 0.03 | 24.0 | 2.0 | 6.2 | |
| ENSTGUG00000009157 | RPP14 | 0.234 | 0.032 | 24.0 | 17.0 | 25.7 | 0.10 | 0.01 | 28.0 | 15.0 | 10.1 | |
| ENSTGUG00000004568 | HMGB3 | 0.234 | 0.032 | 24.0 | 18.0 | 24.5 | 0.18 | 0.03 | 25.3 | 20.7 | 34.5 | |
| ENSTGUG00000004449 | PSMD12 | 0.234 | 0.032 | 24.0 | 21.0 | 24.4 | 0.18 | 0.03 | 26.0 | 18.0 | 31.9 | |
| ENSTGUG00000007052 | ASAH1 | 0.234 | 0.032 | 24.0 | 2.0 | 24.3 | 0.18 | 0.03 | 24.0 | 12.0 | 30.4 | |
| ENSTGUG00000014485 |  | 0.234 | 0.032 | 24.0 | 20.0 | 24.3 | 0.03 | 0.00 | 24.0 | 21.0 | 15.6 | |
| ENSTGUG00000008372 | MARVELD3-2 | 0.234 | 0.032 | 24.0 | 9.0 | 23.9 | 0.00 | 0.00 | 24.0 | 8.0 | 23.6 | |
| ENSTGUG00000014836 |  | 0.234 | 0.032 | 24.0 | 2.0 | 23.5 | 0.18 | 0.03 | 24.0 | 6.0 | 2.5 | |
| ENSTGUG00000010402 | CRYZ | 0.234 | 0.032 | 24.0 | 17.0 | 22.6 | 0.03 | 0.00 | 24.0 | 14.0 | 17.1 | |
| ENSTGUG00000015160 | PA2G4 | 0.234 | 0.032 | 24.0 | 12.0 | 22.5 | 0.18 | 0.03 | 24.0 | 4.0 | 17.1 | |
| ENSTGUG00000010978 | SEC62 | 0.234 | 0.032 | 24.0 | 2.0 | 22.5 | 0.03 | 0.00 | 24.0 | 21.0 | 19.8 | |
| ENSTGUG00000006138 | GMPPA | 0.234 | 0.032 | 26.0 | 15.0 | 22.0 | 0.03 | 0.00 | 24.0 | 2.0 | 16.2 | |
| ENSTGUG00000017148 | UHMK1 | 0.234 | 0.032 | 26.0 | 2.0 | 22.0 | 0.00 | 0.00 | 24.0 | 6.0 | 7.5 | |
| ENSTGUG00000015954 | ARNTL | 0.234 | 0.032 | 25.3 | 12.7 | 21.5 | 0.03 | 0.00 | 24.0 | 13.0 | 10.4 | |
| ENSTGUG00000002413 | NPDC1-2 | 0.234 | 0.032 | 25.3 | 12.7 | 20.2 | 0.03 | 0.00 | 26.0 | 5.0 | 19.4 | |
| ENSTGUG00000007154 | ALDH3A1 | 0.234 | 0.032 | 24.0 | 4.0 | 19.8 | 0.03 | 0.00 | 24.0 | 5.0 | 22.2 | |
| ENSTGUG00000003640 | RAC3 | 0.234 | 0.032 | 24.0 | 8.0 | 19.3 | 0.18 | 0.03 | 24.0 | 9.0 | 19.6 | |
| ENSTGUG00000007511 | DEPDC6-1 | 0.234 | 0.032 | 24.0 | 4.0 | 18.7 | 0.00 | 0.00 | 24.0 | 2.0 | 14.4 | |
| ENSTGUG00000002492 | DHRS3 | 0.234 | 0.032 | 24.0 | 10.0 | 18.6 | 0.18 | 0.03 | 24.0 | 9.0 | 10.0 | |
| ENSTGUG00000010503 | NIPA2 | 0.234 | 0.032 | 24.0 | 0.0 | 17.5 | 0.18 | 0.03 | 24.0 | 21.0 | 39.5 | |
| ENSTGUG00000004630 | TMEM120A | 0.234 | 0.032 | 25.3 | 16.7 | 16.8 | 0.03 | 0.00 | 24.0 | 22.0 | 16.5 | |
| ENSTGUG00000001961 | BXDC2 | 0.234 | 0.032 | 24.0 | 21.0 | 16.7 | 0.18 | 0.03 | 24.0 | 18.0 | 34.4 | |
| ENSTGUG00000009525 | SEH1L | 0.234 | 0.032 | 24.0 | 22.0 | 16.0 | 0.18 | 0.03 | 24.0 | 21.0 | 16.3 | |
| ENSTGUG00000010920 | SLC2A2 | 0.234 | 0.032 | 24.0 | 6.0 | 14.9 | 0.03 | 0.00 | 24.0 | 1.0 | 17.9 | |
| ENSTGUG00000002774 | RAMP2 | 0.234 | 0.032 | 25.3 | 12.7 | 13.8 | 0.18 | 0.03 | 24.0 | 4.0 | 9.0 | |
| ENSTGUG00000011660 | SLC46A3 | 0.234 | 0.032 | 26.0 | 2.0 | 13.6 | 0.18 | 0.03 | 24.0 | 9.0 | 5.5 | |
| ENSTGUG00000011864 |  | 0.234 | 0.032 | 25.3 | 9.3 | 13.0 | 0.18 | 0.03 | 24.0 | 18.0 | 10.5 | |
| ENSTGUG00000000281 | MOBKL2B | 0.234 | 0.032 | 26.0 | 15.0 | 12.7 | 0.18 | 0.03 | 24.0 | 2.0 | 3.6 | |
| ENSTGUG00000011728 | CA2 | 0.234 | 0.032 | 24.0 | 6.0 | 12.6 | 0.03 | 0.00 | 24.0 | 12.0 | 4.3 | |
| ENSTGUG00000004980 |  | 0.234 | 0.032 | 26.0 | 15.0 | 12.6 | 0.03 | 0.00 | 24.0 | 12.0 | 5.5 | |
| ENSTGUG00000009147 | CNDP2 | 0.234 | 0.032 | 25.3 | 16.7 | 12.5 | 0.00 | 0.00 | 24.0 | 12.0 | 7.1 | |
| ENSTGUG00000009076 | PDCD5 | 0.234 | 0.032 | 24.0 | 18.0 | 11.9 | 0.10 | 0.01 | 28.0 | 17.0 | 6.9 | |
| ENSTGUG00000012311 | C6orf66 | 0.234 | 0.032 | 24.0 | 18.0 | 11.7 | 0.18 | 0.03 | 24.0 | 14.0 | 7.3 | |
| ENSTGUG00000002995 | ANGEL2 | 0.234 | 0.032 | 24.0 | 21.0 | 11.7 | 0.18 | 0.03 | 24.0 | 14.0 | 3.2 | |
| ENSTGUG00000005479 | NSDHL | 0.234 | 0.032 | 24.0 | 18.0 | 11.6 | 0.18 | 0.03 | 24.0 | 1.0 | 39.9 | |
| ENSTGUG00000009710 | RNF185 | 0.234 | 0.032 | 24.0 | 18.0 | 11.3 | 0.00 | 0.00 | 24.0 | 12.0 | 9.8 | |
| ENSTGUG00000010055 | CCNYL1 | 0.234 | 0.032 | 25.3 | 9.3 | 10.6 | 0.18 | 0.03 | 24.0 | 4.0 | 2.2 | |
| ENSTGUG00000001151 | SRFBP1 | 0.234 | 0.032 | 25.3 | 9.3 | 10.5 | 0.18 | 0.03 | 24.0 | 18.0 | 3.4 | |
| ENSTGUG00000006131 | DCTN5 | 0.234 | 0.032 | 26.0 | 15.0 | 10.3 | 0.18 | 0.03 | 24.0 | 2.0 | 1.5 | |
| ENSTGUG00000017188 |  | 0.234 | 0.032 | 24.0 | 8.0 | 10.2 | 0.00 | 0.00 | 24.0 | 16.0 | 12.4 | |
| ENSTGUG00000009634 | ACADS | 0.234 | 0.032 | 24.0 | 6.0 | 10.1 | 0.18 | 0.03 | 24.0 | 2.0 | 14.3 | |
| ENSTGUG00000007567 | DAP | 0.234 | 0.032 | 24.0 | 4.0 | 10.1 | 0.03 | 0.00 | 26.0 | 2.0 | 32.1 | |
| ENSTGUG00000011745 | WWP1 | 0.234 | 0.032 | 24.0 | 2.0 | 9.8 | 0.10 | 0.01 | 28.0 | 15.0 | 8.8 | |
| ENSTGUG00000011257 | MTFR1 | 0.234 | 0.032 | 24.0 | 21.0 | 9.8 | 0.18 | 0.03 | 24.0 | 21.0 | 28.1 | |
| ENSTGUG00000009896 | RNH1 | 0.234 | 0.032 | 25.3 | 9.3 | 9.7 | 0.18 | 0.03 | 24.0 | 18.0 | 9.1 | |
| ENSTGUG00000002873 | NBR1 | 0.234 | 0.032 | 25.3 | 9.3 | 9.6 | 0.00 | 0.00 | 24.0 | 22.0 | 11.1 | |
| ENSTGUG00000005440 | C12orf65 | 0.234 | 0.032 | 25.3 | 16.7 | 9.5 | 0.03 | 0.00 | 24.0 | 14.0 | 9.3 | |
| ENSTGUG00000000684 | MRPL22 | 0.234 | 0.032 | 26.0 | 15.0 | 9.1 | 0.10 | 0.01 | 28.0 | 15.0 | 2.5 | |
| ENSTGUG00000006979 | GRB14 | 0.234 | 0.032 | 24.0 | 2.0 | 9.0 | 0.18 | 0.03 | 24.0 | 22.0 | 6.1 | |
| ENSTGUG00000000924 | CUL2 | 0.234 | 0.032 | 24.0 | 20.0 | 8.8 | 0.03 | 0.00 | 24.0 | 20.0 | 10.7 | |
| ENSTGUG00000000631 |  | 0.234 | 0.032 | 24.0 | 6.0 | 8.6 | 0.18 | 0.03 | 24.0 | 12.0 | 6.8 | |
| ENSTGUG00000011561 | TCEB1 | 0.234 | 0.032 | 24.0 | 18.0 | 8.6 | 0.18 | 0.03 | 24.0 | 20.0 | 17.5 | |
| ENSTGUG00000014953 | CAPRIN1 | 0.234 | 0.032 | 26.0 | 2.0 | 8.1 | 0.03 | 0.00 | 24.0 | 6.0 | 3.5 | |
| ENSTGUG00000001918 | SUB1 | 0.234 | 0.032 | 24.0 | 20.0 | 8.1 | 0.18 | 0.03 | 26.0 | 18.0 | 12.5 | |
| ENSTGUG00000004558 | VMA21 | 0.234 | 0.032 | 26.0 | 15.0 | 8.0 | 0.00 | 0.00 | 24.0 | 16.0 | 5.4 | |
| ENSTGUG00000008223 | MRPL38 | 0.234 | 0.032 | 26.0 | 15.0 | 7.9 | 0.18 | 0.03 | 24.0 | 1.0 | 3.0 | |
| ENSTGUG00000001248 | C9orf80 | 0.234 | 0.032 | 24.0 | 18.0 | 7.9 | 0.00 | 0.00 | 24.0 | 16.0 | 3.2 | |
| ENSTGUG00000001790 | CUL1 | 0.234 | 0.032 | 24.0 | 21.0 | 7.9 | 0.03 | 0.00 | 26.0 | 18.0 | 13.8 | |
| ENSTGUG00000002337 | MRPS30 | 0.234 | 0.032 | 24.0 | 18.0 | 7.8 | 0.18 | 0.03 | 25.3 | 20.7 | 4.4 | |
| ENSTGUG00000005077 | CAPZA2 | 0.234 | 0.032 | 24.0 | 21.0 | 7.8 | 0.18 | 0.03 | 24.0 | 17.0 | 7.7 | |
| ENSTGUG00000003045 | MIB2 | 0.234 | 0.032 | 24.0 | 8.0 | 7.6 | 0.10 | 0.01 | 28.0 | 3.0 | 23.5 | |
| ENSTGUG00000007981 |  | 0.234 | 0.032 | 26.0 | 2.0 | 7.3 | 0.18 | 0.03 | 24.0 | 4.0 | 5.1 | |
| ENSTGUG00000001087 | MAN1C1 | 0.234 | 0.032 | 24.0 | 1.0 | 7.1 | 0.18 | 0.03 | 24.0 | 0.0 | 12.8 | |
| ENSTGUG00000010387 | C18orf45 | 0.234 | 0.032 | 24.0 | 12.0 | 7.0 | 0.03 | 0.00 | 24.0 | 9.0 | 5.8 | |
| ENSTGUG00000002480 | SKIV2L2 | 0.234 | 0.032 | 24.0 | 20.0 | 6.9 | 0.18 | 0.03 | 25.3 | 20.7 | 8.3 | |
| ENSTGUG00000004237 |  | 0.234 | 0.032 | 24.0 | 12.0 | 6.8 | 0.18 | 0.03 | 26.0 | 5.0 | 2.6 | |
| ENSTGUG00000000964 |  | 0.234 | 0.032 | 24.0 | 0.0 | 6.8 | 0.03 | 0.00 | 24.0 | 22.0 | 17.8 | |
| ENSTGUG00000001547 | GNL2 | 0.234 | 0.032 | 24.0 | 21.0 | 6.7 | 0.18 | 0.03 | 24.0 | 22.0 | 1.9 | |
| ENSTGUG00000000055 | AGAP3 | 0.234 | 0.032 | 24.0 | 10.0 | 6.6 | 0.18 | 0.03 | 25.3 | 8.0 | 7.0 | |
| ENSTGUG00000002557 | ANKMY2 | 0.234 | 0.032 | 25.3 | 9.3 | 6.6 | 0.18 | 0.03 | 24.0 | 13.0 | 1.7 | |
| ENSTGUG00000011803 | STARD13 | 0.234 | 0.032 | 26.0 | 2.0 | 6.6 | 0.18 | 0.03 | 24.0 | 12.0 | 4.4 | |
| ENSTGUG00000006691 | CAND1 | 0.234 | 0.032 | 24.0 | 2.0 | 6.6 | 0.18 | 0.03 | 24.0 | 21.0 | 4.1 | |
| ENSTGUG00000005651 | NDEL1 | 0.234 | 0.032 | 24.0 | 18.0 | 6.5 | 0.03 | 0.00 | 24.0 | 22.0 | 7.4 | |
| ENSTGUG00000008754 | ITFG1 | 0.234 | 0.032 | 24.0 | 18.0 | 6.4 | 0.03 | 0.00 | 24.0 | 21.0 | 1.5 | |
| ENSTGUG00000003917 | GGCT | 0.234 | 0.032 | 24.0 | 2.0 | 6.3 | 0.03 | 0.00 | 24.0 | 16.0 | 6.6 | |
| ENSTGUG00000004676 | TOP1 | 0.234 | 0.032 | 25.3 | 9.3 | 6.3 | 0.03 | 0.00 | 24.0 | 4.0 | 6.7 | |
| ENSTGUG00000006397 | MRS2L | 0.234 | 0.032 | 24.0 | 2.0 | 6.2 | 0.03 | 0.00 | 24.0 | 1.0 | 7.5 | |
| ENSTGUG00000004996 | POLR2C | 0.234 | 0.032 | 24.0 | 14.0 | 6.2 | 0.03 | 0.00 | 24.0 | 2.0 | 3.4 | |
| ENSTGUG00000005880 | TMEM14C | 0.234 | 0.032 | 24.0 | 17.0 | 5.9 | 0.18 | 0.03 | 24.0 | 14.0 | 7.9 | |
| ENSTGUG00000009936 | PHF5A | 0.234 | 0.032 | 24.0 | 18.0 | 5.8 | 0.18 | 0.03 | 24.0 | 0.0 | 15.9 | |
| ENSTGUG00000012137 | ATP6V1C1 | 0.234 | 0.032 | 24.0 | 21.0 | 5.8 | 0.18 | 0.03 | 24.0 | 20.0 | 4.6 | |
| ENSTGUG00000001629 | C5orf32 | 0.234 | 0.032 | 24.0 | 18.0 | 5.8 | 0.18 | 0.03 | 24.0 | 1.0 | 4.4 | |
| ENSTGUG00000000932 | PARD3 | 0.234 | 0.032 | 24.0 | 9.0 | 5.8 | 0.03 | 0.00 | 24.0 | 12.0 | 5.0 | |
| ENSTGUG00000014329 | CSK | 0.234 | 0.032 | 25.3 | 12.7 | 5.8 | 0.18 | 0.03 | 24.0 | 8.0 | 1.2 | |
| ENSTGUG00000008071 | INO80C | 0.234 | 0.032 | 24.0 | 5.0 | 5.7 | 0.18 | 0.03 | 24.0 | 17.0 | 5.4 | |
| ENSTGUG00000001714 | PDCD6IP | 0.234 | 0.032 | 26.0 | 2.0 | 5.4 | 0.18 | 0.03 | 24.0 | 20.0 | 1.6 | |
| ENSTGUG00000006322 | FKTN | 0.234 | 0.032 | 24.0 | 2.0 | 5.3 | 0.18 | 0.03 | 24.0 | 14.0 | 3.1 | |
| ENSTGUG00000002097 | WDR70 | 0.234 | 0.032 | 24.0 | 18.0 | 5.3 | 0.18 | 0.03 | 24.0 | 20.0 | 3.7 | |
| ENSTGUG00000008972 | PCSK6 | 0.234 | 0.032 | 24.0 | 9.0 | 5.3 | 0.18 | 0.03 | 28.0 | 2.0 | 8.6 | |
| ENSTGUG00000002047 |  | 0.234 | 0.032 | 25.3 | 9.3 | 5.2 | 0.10 | 0.01 | 28.0 | 17.0 | 3.5 | |
| ENSTGUG00000008228 |  | 0.234 | 0.032 | 24.0 | 20.0 | 5.2 | 0.03 | 0.00 | 25.3 | 16.7 | 4.7 | |
| ENSTGUG00000009472 | CEBPA | 0.234 | 0.032 | 24.0 | 6.0 | 5.2 | 0.18 | 0.03 | 28.0 | 2.0 | 9.6 | |
| ENSTGUG00000001031 | ELL | 0.234 | 0.032 | 24.0 | 0.0 | 5.1 | 0.03 | 0.00 | 24.0 | 2.0 | 11.0 | |
| ENSTGUG00000007507 | USP8 | 0.234 | 0.032 | 24.0 | 2.0 | 5.1 | 0.03 | 0.00 | 24.0 | 14.0 | 4.0 | |
| ENSTGUG00000005678 | ABCC6 | 0.234 | 0.032 | 24.0 | 6.0 | 5.0 | 0.18 | 0.03 | 24.0 | 14.0 | 1.6 | |
| ENSTGUG00000009219 | LYRM1-2 | 0.234 | 0.032 | 24.0 | 21.0 | 5.0 | 0.03 | 0.00 | 24.0 | 20.0 | 2.4 | |
| ENSTGUG00000008807 | EIF2AK1 | 0.234 | 0.032 | 26.0 | 15.0 | 4.9 | 0.18 | 0.03 | 24.0 | 13.0 | 0.9 | |
| ENSTGUG00000004898 | ERI1 | 0.234 | 0.032 | 24.0 | 17.0 | 4.9 | 0.03 | 0.00 | 24.0 | 0.0 | 2.5 | |
| ENSTGUG00000006205 | ZNF326 | 0.234 | 0.032 | 25.3 | 9.3 | 4.8 | 0.18 | 0.03 | 24.0 | 16.0 | 2.2 | |
| ENSTGUG00000003907 | SUPV3L1 | 0.234 | 0.032 | 26.0 | 15.0 | 4.7 | 0.03 | 0.00 | 24.0 | 21.0 | 4.4 | |
| ENSTGUG00000003317 | METAP1 | 0.234 | 0.032 | 26.0 | 2.0 | 4.7 | 0.03 | 0.00 | 24.0 | 12.0 | 2.2 | |
| ENSTGUG00000000254 | CYP8B1 | 0.234 | 0.032 | 24.0 | 21.0 | 4.7 | 0.03 | 0.00 | 24.0 | 22.0 | 6.1 | |
| ENSTGUG00000004697 | EXOSC2 | 0.234 | 0.032 | 26.0 | 15.0 | 4.6 | 0.18 | 0.03 | 25.3 | 16.7 | 2.9 | |
| ENSTGUG00000018515 |  | 0.234 | 0.032 | 25.3 | 12.7 | 4.6 | 0.18 | 0.03 | 26.0 | 5.0 | 1.4 | |
| ENSTGUG00000003693 | CMTM6 | 0.234 | 0.032 | 24.0 | 17.0 | 4.6 | 0.03 | 0.00 | 24.0 | 13.0 | 11.4 | |
| ENSTGUG00000003199 | C9orf103 | 0.234 | 0.032 | 26.0 | 18.0 | 4.5 | 0.18 | 0.03 | 24.0 | 10.0 | 3.4 | |
| ENSTGUG00000007229 | ALKBH5 | 0.234 | 0.032 | 24.0 | 6.0 | 4.5 | 0.10 | 0.01 | 28.0 | 3.0 | 5.7 | |
| ENSTGUG00000005032 | HS3ST3B1 | 0.234 | 0.032 | 26.0 | 15.0 | 4.4 | 0.18 | 0.03 | 24.0 | 2.0 | 1.4 | |
| ENSTGUG00000006503 |  | 0.234 | 0.032 | 24.0 | 8.0 | 4.3 | 0.03 | 0.00 | 24.0 | 9.0 | 4.1 | |
| ENSTGUG00000001136 | PDSS1 | 0.234 | 0.032 | 24.0 | 20.0 | 4.2 | 0.18 | 0.03 | 24.0 | 20.0 | 5.6 | |
| ENSTGUG00000008898 | PARD6G | 0.234 | 0.032 | 24.0 | 2.0 | 4.2 | 0.18 | 0.03 | 24.0 | 10.0 | 15.9 | |
| ENSTGUG00000009164 | SLC22A18 | 0.234 | 0.032 | 24.0 | 21.0 | 4.0 | 0.18 | 0.03 | 24.0 | 18.0 | 2.0 | |
| ENSTGUG00000004506 | UBA6 | 0.234 | 0.032 | 24.0 | 0.0 | 3.9 | 0.18 | 0.03 | 26.0 | 15.0 | 1.9 | |
| ENSTGUG00000014601 | SLC23A1 | 0.234 | 0.032 | 26.0 | 15.0 | 3.8 | 0.18 | 0.03 | 24.0 | 0.0 | 0.8 | |
| ENSTGUG00000002477 | APOOL | 0.234 | 0.032 | 24.0 | 17.0 | 3.8 | 0.18 | 0.03 | 24.0 | 16.0 | 4.7 | |
| ENSTGUG00000007151 | DNAJC5 | 0.234 | 0.032 | 26.0 | 15.0 | 3.7 | 0.18 | 0.03 | 24.0 | 9.0 | 1.1 | |
| ENSTGUG00000015756 | RHOG | 0.234 | 0.032 | 25.3 | 12.7 | 3.7 | 0.03 | 0.00 | 24.0 | 8.0 | 1.1 | |
| ENSTGUG00000012044 | TRAPPC6B | 0.234 | 0.032 | 24.0 | 17.0 | 3.6 | 0.18 | 0.03 | 24.0 | 14.0 | 2.8 | |
| ENSTGUG00000001827 | PSMA2 | 0.234 | 0.032 | 24.0 | 21.0 | 3.6 | 0.03 | 0.00 | 24.0 | 20.0 | 3.7 | |
| ENSTGUG00000000636 | SLC30A5 | 0.234 | 0.032 | 24.0 | 18.0 | 3.5 | 0.10 | 0.01 | 28.0 | 17.0 | 7.6 | |
| ENSTGUG00000006937 | ACRC | 0.234 | 0.032 | 25.3 | 12.7 | 3.5 | 0.03 | 0.00 | 24.0 | 8.0 | 1.7 | |
| ENSTGUG00000014560 |  | 0.234 | 0.032 | 24.0 | 14.0 | 3.5 | 0.18 | 0.03 | 28.0 | 2.0 | 3.1 | |
| ENSTGUG00000009944 | TEF | 0.234 | 0.032 | 24.0 | 1.0 | 3.4 | 0.03 | 0.00 | 24.0 | 0.0 | 6.5 | |
| ENSTGUG00000007847 | SCFD2-1 | 0.234 | 0.032 | 26.0 | 2.0 | 3.4 | 0.00 | 0.00 | 24.0 | 6.0 | 2.9 | |
| ENSTGUG00000006764 | C21orf2 | 0.234 | 0.032 | 28.0 | 16.0 | 3.4 | 0.18 | 0.03 | 24.0 | 0.0 | 0.7 | |
| ENSTGUG00000006419 | ZNHIT6 | 0.234 | 0.032 | 24.0 | 21.0 | 3.3 | 0.18 | 0.03 | 28.0 | 16.0 | 2.8 | |
| ENSTGUG00000008820 | CDC42EP3 | 0.234 | 0.032 | 26.0 | 2.0 | 3.3 | 0.03 | 0.00 | 24.0 | 12.0 | 3.2 | |
| ENSTGUG00000009979 | JAK1 | 0.234 | 0.032 | 24.0 | 9.0 | 3.2 | 0.18 | 0.03 | 25.3 | 8.0 | 7.6 | |
| ENSTGUG00000009460 | SFT2D1 | 0.234 | 0.032 | 24.0 | 18.0 | 3.2 | 0.18 | 0.03 | 28.0 | 16.0 | 7.0 | |
| ENSTGUG00000000565 | C9orf3 | 0.234 | 0.032 | 24.0 | 4.0 | 3.2 | 0.18 | 0.03 | 24.0 | 0.0 | 3.1 | |
| ENSTGUG00000010356 | GATA6 | 0.234 | 0.032 | 26.0 | 5.0 | 3.1 | 0.03 | 0.00 | 24.0 | 5.0 | 0.6 | |
| ENSTGUG00000014461 | SPAG7 | 0.234 | 0.032 | 25.3 | 12.7 | 3.1 | 0.18 | 0.03 | 24.0 | 2.0 | 0.2 | |
| ENSTGUG00000000845 | DCTN4 | 0.234 | 0.032 | 25.3 | 9.3 | 3.1 | 0.18 | 0.03 | 24.0 | 2.0 | 1.3 | |
| ENSTGUG00000014718 | NRBP1 | 0.234 | 0.032 | 26.0 | 15.0 | 3.1 | 0.18 | 0.03 | 24.0 | 1.0 | 1.3 | |
| ENSTGUG00000006746 | HS3ST6 | 0.234 | 0.032 | 26.0 | 15.0 | 3.0 | 0.18 | 0.03 | 24.0 | 1.0 | 1.1 | |
| ENSTGUG00000012037 | TTLL1 | 0.234 | 0.032 | 25.3 | 4.0 | 3.0 | 0.18 | 0.03 | 24.0 | 14.0 | 2.0 | |
| ENSTGUG00000005458 | MYO1C | 0.234 | 0.032 | 24.0 | 4.0 | 2.9 | 0.18 | 0.03 | 28.0 | 2.0 | 6.3 | |
| ENSTGUG00000006285 | FBXO8 | 0.234 | 0.032 | 24.0 | 22.0 | 2.9 | 0.18 | 0.03 | 24.0 | 17.0 | 7.4 | |
| ENSTGUG00000000977 | FAM46B | 0.234 | 0.032 | 24.0 | 10.0 | 2.9 | 0.03 | 0.00 | 24.0 | 10.0 | 1.3 | |
| ENSTGUG00000003292 | LONRF3 | 0.234 | 0.032 | 24.0 | 10.0 | 2.8 | 0.18 | 0.03 | 24.0 | 13.0 | 6.9 | |
| ENSTGUG00000012151 | PPFIBP1 | 0.234 | 0.032 | 24.0 | 14.0 | 2.8 | 0.00 | 0.00 | 24.0 | 16.0 | 2.8 | |
| ENSTGUG00000016832 |  | 0.234 | 0.032 | 25.3 | 12.7 | 2.8 | 0.18 | 0.03 | 24.0 | 2.0 | 2.4 | |
| ENSTGUG00000013370 | METT10D | 0.234 | 0.032 | 24.0 | 6.0 | 2.8 | 0.03 | 0.00 | 24.0 | 12.0 | 2.4 | |
| ENSTGUG00000001599 | TMEM173 | 0.234 | 0.032 | 26.0 | 15.0 | 2.8 | 0.00 | 0.00 | 24.0 | 10.0 | 1.0 | |
| ENSTGUG00000005653 | TJP1 | 0.234 | 0.032 | 26.0 | 2.0 | 2.8 | 0.18 | 0.03 | 24.0 | 22.0 | 1.6 | |
| ENSTGUG00000008935 | TSG101 | 0.234 | 0.032 | 24.0 | 18.0 | 2.7 | 0.18 | 0.03 | 24.0 | 20.0 | 5.1 | |
| ENSTGUG00000003346 | ABCA3 | 0.234 | 0.032 | 24.0 | 12.0 | 2.7 | 0.18 | 0.03 | 24.0 | 5.0 | 1.0 | |
| ENSTGUG00000007581 | NUP160 | 0.234 | 0.032 | 25.3 | 12.7 | 2.6 | 0.18 | 0.03 | 26.0 | 5.0 | 1.9 | |
| ENSTGUG00000011067 | SERPINI1 | 0.234 | 0.032 | 24.0 | 18.0 | 2.6 | 0.18 | 0.03 | 24.0 | 14.0 | 3.1 | |
| ENSTGUG00000003413 | UPF3B | 0.234 | 0.032 | 24.0 | 18.0 | 2.6 | 0.18 | 0.03 | 24.0 | 20.0 | 5.5 | |
| ENSTGUG00000004493 | VPS45 | 0.234 | 0.032 | 24.0 | 14.0 | 2.6 | 0.18 | 0.03 | 24.0 | 17.0 | 0.9 | |
| ENSTGUG00000005745 | PKNOX1 | 0.234 | 0.032 | 26.0 | 2.0 | 2.5 | 0.18 | 0.03 | 24.0 | 9.0 | 1.2 | |
| ENSTGUG00000001051 | C19orf50 | 0.234 | 0.032 | 24.0 | 14.0 | 2.5 | 0.18 | 0.03 | 26.0 | 2.0 | 20.1 | |
| ENSTGUG00000003821 | TSEN15 | 0.234 | 0.032 | 25.3 | 12.7 | 2.5 | 0.18 | 0.03 | 28.0 | 2.0 | 0.9 | |
| ENSTGUG00000012454 | BPGM | 0.234 | 0.032 | 26.0 | 2.0 | 2.5 | 0.18 | 0.03 | 24.0 | 12.0 | 0.5 | |
| ENSTGUG00000005722 |  | 0.234 | 0.032 | 24.0 | 5.0 | 2.4 | 0.03 | 0.00 | 24.0 | 5.0 | 4.1 | |
| ENSTGUG00000008286 | MSL3L1 | 0.234 | 0.032 | 24.0 | 2.0 | 2.4 | 0.03 | 0.00 | 24.0 | 16.0 | 6.2 | |
| ENSTGUG00000009820 |  | 0.234 | 0.032 | 25.3 | 9.3 | 2.4 | 0.03 | 0.00 | 24.0 | 22.0 | 1.4 | |
| ENSTGUG00000001689 | UBAP2 | 0.234 | 0.032 | 25.3 | 12.7 | 2.4 | 0.03 | 0.00 | 24.0 | 6.0 | 1.1 | |
| ENSTGUG00000000153 |  | 0.234 | 0.032 | 24.0 | 12.0 | 2.3 | 0.03 | 0.00 | 26.0 | 5.0 | 4.4 | |
| ENSTGUG00000006752 | DAB2IP | 0.234 | 0.032 | 24.0 | 6.0 | 2.3 | 0.10 | 0.01 | 28.0 | 1.0 | 10.3 | |
| ENSTGUG00000009586 | ZFYVE27 | 0.234 | 0.032 | 26.0 | 15.0 | 2.3 | 0.03 | 0.00 | 24.0 | 0.0 | 1.1 | |
| ENSTGUG00000003610 | CLSTN1 | 0.234 | 0.032 | 24.0 | 6.0 | 2.3 | 0.18 | 0.03 | 24.0 | 5.0 | 2.3 | |
| ENSTGUG00000003149 | CBX4 | 0.234 | 0.032 | 24.0 | 13.0 | 2.3 | 0.18 | 0.03 | 24.0 | 22.0 | 2.6 | |
| ENSTGUG00000005354 | CRCP | 0.234 | 0.032 | 24.0 | 16.0 | 2.2 | 0.18 | 0.03 | 24.0 | 20.0 | 2.3 | |
| ENSTGUG00000018247 |  | 0.234 | 0.032 | 24.0 | 10.0 | 2.1 | 0.18 | 0.03 | 26.0 | 5.0 | 1.7 | |
| ENSTGUG00000004953 | DHX37 | 0.234 | 0.032 | 24.0 | 1.0 | 2.1 | 0.03 | 0.00 | 24.0 | 5.0 | 0.2 | |
| ENSTGUG00000000573 |  | 0.234 | 0.032 | 26.0 | 15.0 | 2.1 | 0.03 | 0.00 | 24.0 | 10.0 | 0.7 | |
| ENSTGUG00000011351 | KATNA1 | 0.234 | 0.032 | 26.0 | 15.0 | 2.1 | 0.18 | 0.03 | 24.0 | 16.0 | 0.8 | |
| ENSTGUG00000003367 | KIAA0240 | 0.234 | 0.032 | 26.0 | 2.0 | 2.1 | 0.18 | 0.03 | 25.3 | 9.3 | 1.6 | |
| ENSTGUG00000003107 |  | 0.234 | 0.032 | 24.0 | 16.0 | 2.1 | 0.03 | 0.00 | 24.0 | 12.0 | 1.2 | |
| ENSTGUG00000000515 | ST14 | 0.234 | 0.032 | 24.0 | 8.0 | 1.9 | 0.18 | 0.03 | 24.0 | 0.0 | 0.6 | |
| ENSTGUG00000002031 | RANBP3L | 0.234 | 0.032 | 26.0 | 2.0 | 1.9 | 0.03 | 0.00 | 24.0 | 4.0 | 0.5 | |
| ENSTGUG00000007484 | FBXW2 | 0.234 | 0.032 | 25.3 | 4.0 | 1.9 | 0.00 | 0.00 | 24.0 | 16.0 | 1.7 | |
| ENSTGUG00000013016 | CPSF3 | 0.234 | 0.032 | 24.0 | 18.0 | 1.9 | 0.03 | 0.00 | 24.0 | 20.0 | 4.2 | |
| ENSTGUG00000005039 | TLN2 | 0.234 | 0.032 | 26.0 | 5.0 | 1.9 | 0.03 | 0.00 | 24.0 | 13.0 | 3.0 | |
| ENSTGUG00000004030 | COL13A1 | 0.234 | 0.032 | 24.0 | 13.0 | 1.9 | 0.18 | 0.03 | 24.0 | 2.0 | 2.0 | |
| ENSTGUG00000009476 | TCTEX1D2 | 0.234 | 0.032 | 25.3 | 12.7 | 1.9 | 0.18 | 0.03 | 24.0 | 13.0 | 5.8 | |
| ENSTGUG00000002534 | SLC38A9 | 0.234 | 0.032 | 24.0 | 2.0 | 1.8 | 0.03 | 0.00 | 24.0 | 18.0 | 2.2 | |
| ENSTGUG00000012165 | YLPM1 | 0.234 | 0.032 | 24.0 | 20.0 | 1.8 | 0.18 | 0.03 | 25.3 | 20.7 | 1.9 | |
| ENSTGUG00000005243 | NTAN1 | 0.234 | 0.032 | 24.0 | 20.0 | 1.7 | 0.18 | 0.03 | 24.0 | 9.0 | 9.5 | |
| ENSTGUG00000010592 | TTC14 | 0.234 | 0.032 | 24.0 | 2.0 | 1.7 | 0.10 | 0.01 | 28.0 | 15.0 | 7.4 | |
| ENSTGUG00000004728 |  | 0.234 | 0.032 | 28.0 | 2.0 | 1.7 | 0.18 | 0.03 | 24.0 | 10.0 | 1.7 | |
| ENSTGUG00000010342 | GTPBP1 | 0.234 | 0.032 | 24.0 | 9.0 | 1.7 | 0.18 | 0.03 | 28.0 | 2.0 | 3.7 | |
| ENSTGUG00000000247 | C1orf69 | 0.234 | 0.032 | 24.0 | 6.0 | 1.7 | 0.18 | 0.03 | 24.0 | 9.0 | 1.3 | |
| ENSTGUG00000015810 |  | 0.234 | 0.032 | 24.0 | 18.0 | 1.7 | 0.03 | 0.00 | 24.0 | 18.0 | 0.8 | |
| ENSTGUG00000001845 | GBA2 | 0.234 | 0.032 | 24.0 | 14.0 | 1.6 | 0.03 | 0.00 | 24.0 | 2.0 | 1.0 | |
| ENSTGUG00000000188 | PTH1R | 0.234 | 0.032 | 24.0 | 2.0 | 1.6 | 0.18 | 0.03 | 24.0 | 22.0 | 4.4 | |
| ENSTGUG00000000841 | TMEM2 | 0.234 | 0.032 | 26.0 | 2.0 | 1.6 | 0.03 | 0.00 | 24.0 | 1.0 | 2.0 | |
| ENSTGUG00000002556 | FOSL2 | 0.234 | 0.032 | 25.3 | 9.3 | 1.6 | 0.18 | 0.03 | 24.0 | 2.0 | 0.9 | |
| ENSTGUG00000003761 | SIN3A | 0.234 | 0.032 | 25.3 | 12.7 | 1.5 | 0.18 | 0.03 | 24.0 | 22.0 | 0.9 | |
| ENSTGUG00000000392 | PIK3R2 | 0.234 | 0.032 | 24.0 | 9.0 | 1.5 | 0.18 | 0.03 | 24.0 | 5.0 | 1.5 | |
| ENSTGUG00000007689 | RIN2 | 0.234 | 0.032 | 24.0 | 9.0 | 1.5 | 0.03 | 0.00 | 24.0 | 12.0 | 2.1 | |
| ENSTGUG00000009799 | USP1 | 0.234 | 0.032 | 25.3 | 4.0 | 1.5 | 0.03 | 0.00 | 24.0 | 13.0 | 2.2 | |
| ENSTGUG00000004884 | SLC28A1 | 0.234 | 0.032 | 25.3 | 12.7 | 1.5 | 0.18 | 0.03 | 24.0 | 10.0 | 0.8 | |
| ENSTGUG00000000867 | TCOF1 | 0.234 | 0.032 | 25.3 | 12.7 | 1.5 | 0.18 | 0.03 | 24.0 | 2.0 | 0.4 | |
| ENSTGUG00000010304 | ESCO1 | 0.234 | 0.032 | 24.0 | 2.0 | 1.5 | 0.18 | 0.03 | 24.0 | 21.0 | 0.5 | |
| ENSTGUG00000005744 | SERPINF1 | 0.234 | 0.032 | 26.0 | 15.0 | 1.5 | 0.18 | 0.03 | 24.0 | 10.0 | 2.1 | |
| ENSTGUG00000006871 | SORBS2 | 0.234 | 0.032 | 26.0 | 2.0 | 1.4 | 0.03 | 0.00 | 24.0 | 2.0 | 0.7 | |
| ENSTGUG00000010955 | JMJD7 | 0.234 | 0.032 | 24.0 | 9.0 | 1.4 | 0.18 | 0.03 | 24.0 | 13.0 | 0.6 | |
| ENSTGUG00000010461 | CAND2 | 0.234 | 0.032 | 25.3 | 9.3 | 1.4 | 0.03 | 0.00 | 24.0 | 8.0 | 1.4 | |
| ENSTGUG00000012605 | DDX10 | 0.234 | 0.032 | 24.0 | 0.0 | 1.4 | 0.18 | 0.03 | 24.0 | 20.0 | 0.8 | |
| ENSTGUG00000010563 | OSBP2 | 0.234 | 0.032 | 24.0 | 13.0 | 1.4 | 0.18 | 0.03 | 24.0 | 2.0 | 0.7 | |
| ENSTGUG00000000584 | RNF145 | 0.234 | 0.032 | 24.0 | 9.0 | 1.4 | 0.18 | 0.03 | 24.0 | 16.0 | 2.7 | |
| ENSTGUG00000012889 | AGPAT5 | 0.234 | 0.032 | 24.0 | 17.0 | 1.3 | 0.18 | 0.03 | 24.0 | 14.0 | 2.3 | |
| ENSTGUG00000000112 | DHX30 | 0.234 | 0.032 | 25.3 | 12.7 | 1.3 | 0.18 | 0.03 | 24.0 | 1.0 | 0.8 | |
| ENSTGUG00000008557 | PLEKHA7 | 0.234 | 0.032 | 26.0 | 2.0 | 1.3 | 0.18 | 0.03 | 25.3 | 9.3 | 3.1 | |
| ENSTGUG00000009870 | ATG4C | 0.234 | 0.032 | 24.0 | 2.0 | 1.3 | 0.18 | 0.03 | 24.0 | 12.0 | 1.3 | |
| ENSTGUG00000013194 | C14orf138 | 0.234 | 0.032 | 26.0 | 15.0 | 1.3 | 0.03 | 0.00 | 24.0 | 21.0 | 1.7 | |
| ENSTGUG00000012069 | ATP7B | 0.234 | 0.032 | 24.0 | 9.0 | 1.3 | 0.00 | 0.00 | 24.0 | 16.0 | 3.9 | |
| ENSTGUG00000001501 | CCDC132 | 0.234 | 0.032 | 24.0 | 18.0 | 1.3 | 0.18 | 0.03 | 28.0 | 16.0 | 4.4 | |
| ENSTGUG00000002863 | FHL1 | 0.234 | 0.032 | 24.0 | 6.0 | 1.2 | 0.03 | 0.00 | 24.0 | 12.0 | 1.7 | |
| ENSTGUG00000008641 | TRPC1 | 0.234 | 0.032 | 24.0 | 8.0 | 1.2 | 0.18 | 0.03 | 24.0 | 14.0 | 1.0 | |
| ENSTGUG00000010258 | DEPDC1 | 0.234 | 0.032 | 25.3 | 12.7 | 1.2 | 0.03 | 0.00 | 24.0 | 18.0 | 0.7 | |
| ENSTGUG00000007786 | SRD5A1 | 0.234 | 0.032 | 24.0 | 4.0 | 1.2 | 0.18 | 0.03 | 24.0 | 14.0 | 1.1 | |
| ENSTGUG00000004920 | ERCC3 | 0.234 | 0.032 | 24.0 | 5.0 | 1.2 | 0.18 | 0.03 | 25.3 | 20.7 | 2.7 | |
| ENSTGUG00000010824 | FNDC3B | 0.234 | 0.032 | 25.3 | 9.3 | 1.1 | 0.03 | 0.00 | 24.0 | 2.0 | 0.6 | |
| ENSTGUG00000001678 | RPA3 | 0.234 | 0.032 | 24.0 | 18.0 | 1.1 | 0.00 | 0.00 | 24.0 | 16.0 | 2.2 | |
| ENSTGUG00000009951 | CACHD1 | 0.234 | 0.032 | 26.0 | 2.0 | 1.1 | 0.00 | 0.00 | 24.0 | 4.0 | 0.3 | |
| ENSTGUG00000010479 | C2orf69 | 0.234 | 0.032 | 25.3 | 4.0 | 1.1 | 0.00 | 0.00 | 24.0 | 12.0 | 1.5 | |
| ENSTGUG00000003083 | PHF20 | 0.234 | 0.032 | 24.0 | 0.0 | 1.1 | 0.03 | 0.00 | 26.0 | 18.0 | 5.0 | |
| ENSTGUG00000004678 | IDS | 0.234 | 0.032 | 24.0 | 18.0 | 1.1 | 0.18 | 0.03 | 26.0 | 18.0 | 5.9 | |
| ENSTGUG00000007986 | ULK1 | 0.234 | 0.032 | 25.3 | 9.3 | 1.1 | 0.03 | 0.00 | 24.0 | 0.0 | 5.5 | |
| ENSTGUG00000017014 |  | 0.234 | 0.032 | 26.0 | 2.0 | 1.0 | 0.18 | 0.03 | 24.0 | 8.0 | 0.4 | |
| ENSTGUG00000008405 | KLHL25 | 0.234 | 0.032 | 24.0 | 9.0 | 1.0 | 0.03 | 0.00 | 24.0 | 9.0 | 0.6 | |
| ENSTGUG00000005362 | ASPN | 0.234 | 0.032 | 24.0 | 9.0 | 1.0 | 0.18 | 0.03 | 28.0 | 2.0 | 1.5 | |
| ENSTGUG00000002716 | ZSWIM6 | 0.234 | 0.032 | 26.0 | 2.0 | 1.0 | 0.18 | 0.03 | 24.0 | 2.0 | 0.8 | |
| ENSTGUG00000013258 | CENPQ | 0.234 | 0.032 | 24.0 | 20.0 | 1.0 | 0.00 | 0.00 | 24.0 | 18.0 | 2.0 | |
| ENSTGUG00000010365 | RGS12 | 0.234 | 0.032 | 25.3 | 9.3 | 0.9 | 0.18 | 0.03 | 24.0 | 13.0 | 0.6 | |
| ENSTGUG00000011467 | FAM60A | 0.234 | 0.032 | 24.0 | 2.0 | 0.9 | 0.18 | 0.03 | 26.0 | 2.0 | 3.8 | |
| ENSTGUG00000007053 | FAM83G | 0.234 | 0.032 | 25.3 | 12.7 | 0.9 | 0.18 | 0.03 | 24.0 | 2.0 | 0.2 | |
| ENSTGUG00000007582 | KIAA0513 | 0.234 | 0.032 | 24.0 | 5.0 | 0.9 | 0.18 | 0.03 | 24.0 | 1.0 | 0.7 | |
| ENSTGUG00000002066 | FBLIM1 | 0.234 | 0.032 | 24.0 | 14.0 | 0.9 | 0.18 | 0.03 | 24.0 | 6.0 | 0.2 | |
| ENSTGUG00000004038 |  | 0.234 | 0.032 | 25.3 | 4.0 | 0.9 | 0.10 | 0.01 | 28.0 | 3.0 | 0.6 | |
| ENSTGUG00000003480 | SIRT1 | 0.234 | 0.032 | 24.0 | 18.0 | 0.9 | 0.18 | 0.03 | 24.0 | 1.0 | 2.0 | |
| ENSTGUG00000012796 | ZNF451 | 0.234 | 0.032 | 24.0 | 5.0 | 0.9 | 0.10 | 0.01 | 28.0 | 17.0 | 2.6 | |
| ENSTGUG00000005926 | RRP1B | 0.234 | 0.032 | 25.3 | 9.3 | 0.9 | 0.03 | 0.00 | 24.0 | 18.0 | 1.8 | |
| ENSTGUG00000003341 | COL6A1 | 0.234 | 0.032 | 24.0 | 9.0 | 0.9 | 0.18 | 0.03 | 25.3 | 8.0 | 1.0 | |
| ENSTGUG00000006112 | PLK1 | 0.234 | 0.032 | 24.0 | 14.0 | 0.9 | 0.02 | 0.00 | 24.0 | 0.0 | 0.2 | |
| ENSTGUG00000011887 | RAD54B-2 | 0.234 | 0.032 | 24.0 | 9.0 | 0.9 | 0.18 | 0.03 | 24.0 | 10.0 | 1.0 | |
| ENSTGUG00000015025 | PPP3CC | 0.234 | 0.032 | 24.0 | 16.0 | 0.8 | 0.18 | 0.03 | 25.3 | 4.0 | 2.0 | |
| ENSTGUG00000011715 | CCNT2 | 0.234 | 0.032 | 26.0 | 2.0 | 0.8 | 0.00 | 0.00 | 24.0 | 10.0 | 0.6 | |
| ENSTGUG00000003872 | MREG | 0.234 | 0.032 | 24.0 | 4.0 | 0.8 | 0.18 | 0.03 | 24.0 | 12.0 | 1.0 | |
| ENSTGUG00000005538 | MUSTN1-1 | 0.234 | 0.032 | 24.0 | 6.0 | 0.8 | 0.18 | 0.03 | 24.0 | 12.0 | 19.9 | |
| ENSTGUG00000012097 | C6orf224 | 0.234 | 0.032 | 26.0 | 5.0 | 0.8 | 0.18 | 0.03 | 24.0 | 17.0 | 0.5 | |
| ENSTGUG00000015879 |  | 0.234 | 0.032 | 24.0 | 8.0 | 0.8 | 0.00 | 0.00 | 24.0 | 20.0 | 1.7 | |
| ENSTGUG00000015156 | NPR1 | 0.234 | 0.032 | 26.0 | 15.0 | 0.8 | 0.18 | 0.03 | 26.0 | 5.0 | 0.5 | |
| ENSTGUG00000001321 |  | 0.234 | 0.032 | 24.0 | 9.0 | 0.8 | 0.18 | 0.03 | 24.0 | 12.0 | 1.4 | |
| ENSTGUG00000016447 |  | 0.234 | 0.032 | 26.0 | 15.0 | 0.8 | 0.03 | 0.00 | 24.0 | 22.0 | 1.0 | |
| ENSTGUG00000010696 | ST3GAL5 | 0.234 | 0.032 | 24.0 | 6.0 | 0.8 | 0.18 | 0.03 | 24.0 | 9.0 | 0.9 | |
| ENSTGUG00000006522 | USP9X | 0.234 | 0.032 | 24.0 | 0.0 | 0.8 | 0.18 | 0.03 | 24.0 | 20.0 | 3.4 | |
| ENSTGUG00000004125 | KCNN3 | 0.234 | 0.032 | 24.0 | 1.0 | 0.7 | 0.03 | 0.00 | 24.0 | 1.0 | 5.6 | |
| ENSTGUG00000013445 | SYNJ1 | 0.234 | 0.032 | 26.0 | 2.0 | 0.7 | 0.03 | 0.00 | 24.0 | 22.0 | 0.7 | |
| ENSTGUG00000008466 | NTN4 | 0.234 | 0.032 | 24.0 | 9.0 | 0.7 | 0.03 | 0.00 | 24.0 | 10.0 | 0.7 | |
| ENSTGUG00000017299 | SCYL1BP1 | 0.234 | 0.032 | 24.0 | 8.0 | 0.7 | 0.03 | 0.00 | 24.0 | 22.0 | 1.1 | |
| ENSTGUG00000006431 | GPR82 | 0.234 | 0.032 | 24.0 | 17.0 | 0.7 | 0.18 | 0.03 | 24.0 | 12.0 | 0.5 | |
| ENSTGUG00000001104 | CEP120 | 0.234 | 0.032 | 24.0 | 6.0 | 0.7 | 0.03 | 0.00 | 24.0 | 14.0 | 0.5 | |
| ENSTGUG00000004337 | NCSTN | 0.234 | 0.032 | 26.0 | 15.0 | 0.7 | 0.18 | 0.03 | 24.0 | 5.0 | 0.3 | |
| ENSTGUG00000008985 |  | 0.234 | 0.032 | 24.0 | 2.0 | 0.7 | 0.03 | 0.00 | 24.0 | 22.0 | 2.3 | |
| ENSTGUG00000002651 |  | 0.234 | 0.032 | 24.0 | 6.0 | 0.7 | 0.18 | 0.03 | 24.0 | 4.0 | 0.1 | |
| ENSTGUG00000010478 | MBD4 | 0.234 | 0.032 | 24.0 | 14.0 | 0.7 | 0.18 | 0.03 | 24.0 | 17.0 | 2.8 | |
| ENSTGUG00000006455 | BCL10 | 0.234 | 0.032 | 24.0 | 13.0 | 0.7 | 0.03 | 0.00 | 24.0 | 8.0 | 0.9 | |
| ENSTGUG00000012673 | DDX11 | 0.234 | 0.032 | 24.0 | 13.0 | 0.7 | 0.18 | 0.03 | 25.3 | 16.7 | 0.7 | |
| ENSTGUG00000018374 |  | 0.234 | 0.032 | 24.0 | 17.0 | 0.7 | 0.18 | 0.03 | 24.0 | 2.0 | 0.4 | |
| ENSTGUG00000018173 |  | 0.234 | 0.032 | 24.0 | 5.0 | 0.7 | 0.03 | 0.00 | 24.0 | 21.0 | 0.7 | |
| ENSTGUG00000008425 | GRK7 | 0.234 | 0.032 | 24.0 | 4.0 | 0.6 | 0.03 | 0.00 | 25.3 | 4.0 | 1.5 | |
| ENSTGUG00000000729 | PTPRS | 0.234 | 0.032 | 24.0 | 9.0 | 0.6 | 0.00 | 0.00 | 24.0 | 8.0 | 0.4 | |
| ENSTGUG00000012839 |  | 0.234 | 0.032 | 24.0 | 2.0 | 0.6 | 0.18 | 0.03 | 24.0 | 6.0 | 1.9 | |
| ENSTGUG00000010043 | ADORA2A | 0.234 | 0.032 | 25.3 | 4.0 | 0.6 | 0.18 | 0.03 | 24.0 | 13.0 | 0.7 | |
| ENSTGUG00000011531 |  | 0.234 | 0.032 | 24.0 | 2.0 | 0.6 | 0.03 | 0.00 | 24.0 | 13.0 | 2.1 | |
| ENSTGUG00000015489 | SOLH | 0.234 | 0.032 | 24.0 | 13.0 | 0.6 | 0.03 | 0.00 | 24.0 | 8.0 | 0.2 | |
| ENSTGUG00000011534 | CDK8 | 0.234 | 0.032 | 24.0 | 6.0 | 0.5 | 0.03 | 0.00 | 24.0 | 20.0 | 1.4 | |
| ENSTGUG00000015063 | SEMA4C | 0.234 | 0.032 | 28.0 | 16.0 | 0.5 | 0.18 | 0.03 | 24.0 | 2.0 | 0.1 | |
| ENSTGUG00000012341 | TNFRSF11B | 0.234 | 0.032 | 26.0 | 18.0 | 0.5 | 0.03 | 0.00 | 24.0 | 8.0 | 1.4 | |
| ENSTGUG00000003067 |  | 0.234 | 0.032 | 24.0 | 2.0 | 0.5 | 0.18 | 0.03 | 24.0 | 8.0 | 0.5 | |
| ENSTGUG00000003274 |  | 0.234 | 0.032 | 24.0 | 6.0 | 0.5 | 0.03 | 0.00 | 24.0 | 16.0 | 0.4 | |
| ENSTGUG00000009310 | GPRC5B | 0.234 | 0.032 | 26.0 | 2.0 | 0.5 | 0.18 | 0.03 | 24.0 | 10.0 | 0.6 | |
| ENSTGUG00000012685 | C14orf129 | 0.234 | 0.032 | 24.0 | 4.0 | 0.5 | 0.18 | 0.03 | 24.0 | 16.0 | 3.9 | |
| ENSTGUG00000004615 | SLC25A24 | 0.234 | 0.032 | 24.0 | 9.0 | 0.5 | 0.18 | 0.03 | 24.0 | 9.0 | 0.4 | |
| ENSTGUG00000014687 |  | 0.234 | 0.032 | 24.0 | 4.0 | 0.5 | 0.18 | 0.03 | 24.0 | 16.0 | 0.8 | |
| ENSTGUG00000006655 | CYTSB | 0.234 | 0.032 | 28.0 | 2.0 | 0.4 | 0.18 | 0.03 | 24.0 | 10.0 | 0.3 | |
| ENSTGUG00000006279 | PASK | 0.234 | 0.032 | 25.3 | 16.7 | 0.4 | 0.18 | 0.03 | 24.0 | 21.0 | 0.2 | |
| ENSTGUG00000004407 | SNX27 | 0.234 | 0.032 | 26.0 | 15.0 | 0.4 | 0.03 | 0.00 | 24.0 | 0.0 | 0.6 | |
| ENSTGUG00000003238 | RAP1GDS1 | 0.234 | 0.032 | 24.0 | 16.0 | 0.4 | 0.03 | 0.00 | 24.0 | 10.0 | 2.2 | |
| ENSTGUG00000010534 | PLXND1 | 0.234 | 0.032 | 24.0 | 6.0 | 0.4 | 0.18 | 0.03 | 24.0 | 5.0 | 1.0 | |
| ENSTGUG00000011761 | ST8SIA1 | 0.234 | 0.032 | 24.0 | 5.0 | 0.4 | 0.18 | 0.03 | 24.0 | 4.0 | 0.3 | |
| ENSTGUG00000003642 | RUFY2 | 0.234 | 0.032 | 24.0 | 6.0 | 0.4 | 0.18 | 0.03 | 24.0 | 16.0 | 0.1 | |
| ENSTGUG00000015714 |  | 0.234 | 0.032 | 24.0 | 20.0 | 0.4 | 0.18 | 0.03 | 25.3 | 20.7 | 0.7 | |
| ENSTGUG00000000513 | ZNF367 | 0.234 | 0.032 | 24.0 | 9.0 | 0.4 | 0.18 | 0.03 | 24.0 | 13.0 | 0.7 | |
| ENSTGUG00000003540 | UBN1 | 0.234 | 0.032 | 24.0 | 17.0 | 0.4 | 0.18 | 0.03 | 24.0 | 20.0 | 0.7 | |
| ENSTGUG00000013473 | FAM168A | 0.234 | 0.032 | 24.0 | 18.0 | 0.4 | 0.18 | 0.03 | 24.0 | 1.0 | 3.5 | |
| ENSTGUG00000000840 | ST3GAL4 | 0.234 | 0.032 | 24.0 | 14.0 | 0.4 | 0.18 | 0.03 | 24.0 | 9.0 | 0.3 | |
| ENSTGUG00000016834 |  | 0.234 | 0.032 | 25.3 | 4.0 | 0.4 | 0.03 | 0.00 | 24.0 | 13.0 | 0.4 | |
| ENSTGUG00000006039 | ERN2 | 0.234 | 0.032 | 24.0 | 12.0 | 0.4 | 0.18 | 0.03 | 24.0 | 14.0 | 0.8 | |
| ENSTGUG00000012233 | BAZ2B | 0.234 | 0.032 | 25.3 | 9.3 | 0.4 | 0.03 | 0.00 | 24.0 | 20.0 | 0.4 | |
| ENSTGUG00000001164 | C10orf63 | 0.234 | 0.032 | 24.0 | 16.0 | 0.3 | 0.10 | 0.01 | 24.0 | 11.0 | 0.3 | |
| ENSTGUG00000013816 |  | 0.234 | 0.032 | 25.3 | 12.7 | 0.3 | 0.10 | 0.01 | 24.0 | 21.0 | 0.5 | |
| ENSTGUG00000015201 |  | 0.234 | 0.032 | 24.0 | 13.0 | 0.3 | 0.18 | 0.03 | 24.0 | 17.0 | 0.3 | |
| ENSTGUG00000006756 | TRIP13 | 0.234 | 0.032 | 25.3 | 16.7 | 0.3 | 0.03 | 0.00 | 24.0 | 14.0 | 0.5 | |
| ENSTGUG00000004638 | CSNK1G1 | 0.234 | 0.032 | 25.3 | 12.7 | 0.3 | 0.18 | 0.03 | 24.0 | 14.0 | 0.4 | |
| ENSTGUG00000009640 |  | 0.234 | 0.032 | 25.3 | 9.3 | 0.3 | 0.18 | 0.03 | 24.0 | 14.0 | 0.9 | |
| ENSTGUG00000009020 | ZNF507 | 0.234 | 0.032 | 24.0 | 6.0 | 0.3 | 0.03 | 0.00 | 24.0 | 2.0 | 0.5 | |
| ENSTGUG00000014777 |  | 0.234 | 0.032 | 24.0 | 9.0 | 0.3 | 0.10 | 0.01 | 24.0 | 4.0 | 0.2 | |
| ENSTGUG00000006729 | LRRC50 | 0.234 | 0.032 | 24.0 | 13.0 | 0.3 | 0.03 | 0.00 | 24.0 | 20.0 | 1.1 | |
| ENSTGUG00000017063 |  | 0.234 | 0.032 | 24.0 | 8.0 | 0.3 | 0.03 | 0.00 | 24.0 | 0.0 | 1.3 | |
| ENSTGUG00000012658 | DICER1 | 0.234 | 0.032 | 26.0 | 2.0 | 0.3 | 0.18 | 0.03 | 24.0 | 20.0 | 0.3 | |
| ENSTGUG00000010260 | VWA3B | 0.234 | 0.032 | 24.0 | 0.0 | 0.3 | 0.03 | 0.00 | 24.0 | 20.0 | 0.5 | |
| ENSTGUG00000007487 | NAPB | 0.234 | 0.032 | 24.0 | 12.0 | 0.3 | 0.03 | 0.00 | 24.0 | 9.0 | 0.5 | |
| ENSTGUG00000002577 | NR3C2 | 0.234 | 0.032 | 24.0 | 2.0 | 0.2 | 0.18 | 0.03 | 25.3 | 9.3 | 2.0 | |
| ENSTGUG00000016665 |  | 0.234 | 0.032 | 24.0 | 20.0 | 0.2 | 0.18 | 0.03 | 24.0 | 14.0 | 0.4 | |
| ENSTGUG00000015990 |  | 0.234 | 0.032 | 24.0 | 1.0 | 0.2 | 0.18 | 0.03 | 24.0 | 2.0 | 0.2 | |
| ENSTGUG00000006713 | B3GNT4 | 0.234 | 0.032 | 24.0 | 8.0 | 0.2 | 0.18 | 0.03 | 24.0 | 2.0 | 0.0 | |
| ENSTGUG00000011763 | BRCA2 | 0.234 | 0.032 | 25.3 | 12.7 | 0.2 | 0.00 | 0.00 | 24.0 | 14.0 | 0.4 | |
| ENSTGUG00000005827 | GPSN2 | 0.234 | 0.032 | 24.0 | 13.0 | 0.2 | 0.03 | 0.00 | 24.0 | 10.0 | 2.0 | |
| ENSTGUG00000009125 | LRRC15 | 0.234 | 0.032 | 26.0 | 15.0 | 0.2 | 0.03 | 0.00 | 24.0 | 2.0 | 0.2 | |
| ENSTGUG00000006182 | PAK7 | 0.234 | 0.032 | 24.0 | 6.0 | 0.2 | 0.18 | 0.03 | 24.0 | 8.0 | 0.3 | |
| ENSTGUG00000005481 | GPC1 | 0.234 | 0.032 | 24.0 | 10.0 | 0.1 | 0.00 | 0.00 | 24.0 | 14.0 | 0.1 | |
| ENSTGUG00000012562 | TG | 0.234 | 0.032 | 24.0 | 22.0 | 0.1 | 0.18 | 0.03 | 26.0 | 5.0 | 0.1 | |
| ENSTGUG00000007046 | RTEL1 | 0.234 | 0.032 | 24.0 | 12.0 | 0.1 | 0.18 | 0.03 | 24.0 | 13.0 | 0.1 | |
| ENSTGUG00000014544 | LAMB2 | 0.234 | 0.032 | 24.0 | 9.0 | 0.1 | 0.18 | 0.03 | 24.0 | 0.0 | 0.1 | |
| ENSTGUG00000001333 | ITGA8 | 0.234 | 0.032 | 24.0 | 2.0 | 0.1 | 0.03 | 0.00 | 24.0 | 8.0 | 0.4 | |
| ENSTGUG00000010382 |  | 0.234 | 0.032 | 24.0 | 13.0 | 0.1 | 0.18 | 0.03 | 24.0 | 16.0 | 0.3 | |
| ENSTGUG00000004495 | GAN | 0.234 | 0.032 | 24.0 | 12.0 | 0.1 | 0.18 | 0.03 | 24.0 | 20.0 | 0.7 | |
| ENSTGUG00000012517 | CECR2 | 0.234 | 0.032 | 24.0 | 2.0 | 0.1 | 0.00 | 0.00 | 24.0 | 8.0 | 0.2 | |
| ENSTGUG00000008125 | C15orf42 | 0.234 | 0.032 | 25.3 | 9.3 | 0.1 | 0.18 | 0.03 | 24.0 | 13.0 | 0.1 | |
| ENSTGUG00000009094 | KNDC1 | 0.234 | 0.032 | 24.0 | 2.0 | 0.1 | 0.18 | 0.03 | 26.0 | 5.0 | 0.2 | |
| ENSTGUG00000004135 | CHST3 | 0.234 | 0.032 | 24.0 | 17.0 | 0.1 | 0.10 | 0.01 | 24.0 | 2.0 | 0.1 | |
| ENSTGUG00000006798 | B4GALNT4 | 0.234 | 0.032 | 24.0 | 14.0 | 0.1 | 0.18 | 0.03 | 24.0 | 10.0 | 0.1 | |
| ENSTGUG00000008763 | ADAMTS17 | 0.234 | 0.032 | 26.0 | 2.0 | 0.1 | 0.18 | 0.03 | 25.3 | 8.0 | 0.1 | |
| ENSTGUG00000010423 | C10orf78 | 0.234 | 0.032 | 24.0 | 4.0 | 0.1 | 0.10 | 0.01 | 24.0 | 10.0 | 0.1 | |
| ENSTGUG00000017323 | TET3 | 0.234 | 0.032 | 25.3 | 16.7 | 0.1 | 0.18 | 0.03 | 25.3 | 4.0 | 0.0 | |
| ENSTGUG00000000377 | VILL | 0.234 | 0.032 | 24.0 | 21.0 | 0.0 | 0.18 | 0.03 | 25.3 | 4.0 | 0.1 | |
| ENSTGUG00000004543 | PTPRZ1 | 0.234 | 0.032 | 25.3 | 4.0 | 0.0 | 0.03 | 0.00 | 24.0 | 13.0 | 0.0 | |
|  |  |  |  |  |  |  |  |  |  |  |  | |
