# Supplementary material for: Transcriptional Signatures in Liver Reveal Metabolic Adaptations to Seasons in Migratory Blackheaded Buntings
Source: Front Physiol. 2018 Nov 27;9:1568. doi: 10.3389/fphys.2018.01568 (PMC6277527; doi:10.3389/fphys.2018.01568)
Supplement: Supplementary file 6 [file Data_Sheet_1.DOCX]

**Supplementary information**

**Methods**

**Monitoring phenotype and measurement of physiological measures**

Each individual was handled several times during the light and dark periods to get acclimatize and avoid handling or neophobia-induced stress. Food intake (FI) was measured consecutively for 2 days. After 24 h from the time food was given previous day, food bowl and spillage along with feces were removed. After removal of feces, this was weighed and subtracted from the food given which when averaged for 2 days gave FI in g bird-1 day -1. From this, the mean (± SE) for the group was calculated. We considered the change in body mass as an index of body fattening, since most, if not all, of the photostimulated fat deposition accounts for weight gain in migratory songbirds including blackheaded buntings (King and Farner, 1959; Misra et al., 2004; Rani et al., 2006, Mishra et al., 2017). To record body mass, birds were weighed on a top-pan balance with an accuracy of 0.1 g. We recorded surface body temperature using thermoscan (Quick shot Infra-red thermometer; model Exp-01B), which measures body temperature in 32 - 42^o^C range. For this, a gentle air blow exposed the keel region skin, and the temperature was recorded as average of 4 - 5 readings in a quick succession with thermoscan placed at about 2 cm distance.

To examine differences at the histological level, a liver piece was fixed in x% formaldehyde tissue, cryosectioned at 8 μm thickness, and stained with Hematoxyline-Eosin (H-E). H-E stained sections were passed through ascending grades of alcohol and cover-slipped. AxioCam ICc1 Rev.4 camera attached to the Zeiss Axio Imager M2 microscope then digitally imaged them. Another 2X2mm liver piece was prepared for the transmission electron microscopy (TEM) to show a better resolution of differences in the fat droplets and vacuoles in liver tissue between two states.

*Measurements of serum glucose and triglycerides.* We measured glucose and triglycerides levels in blood serum collected at 4 h intervals, beginning 1 h after light on, i.e. ZT 1, 5, 9, 13, 17 and 21. A bird was bled only once, and each time, 100 -150 µl of blood was collected by puncturing the wing vein into a capillary tube, and allowed to rest at the room temperature before it was centrifuged \at 3000 rpm for 15 min at 4 ^o^C. The serum was harvested and stored at -20°C until assayed for the hormone concentration. QuantiChromTM Glucose (DIGL-100) and EnzyChromTM Triglyceride (ETGA-200) Assay Kits were used to measure glucose and triglyceride levels in 5 and 10 μl serum samples, respectively, as per the manufacturer’s protocol. Briefly, serum, standards and reagents were thawed on ice. For glucose, standards were diluted in distilled water at the final concentration of 300, 200, 100, 50 mg/dl; distilled water was taken as a blank sample and served as control. A 5 μl of neat serum and diluted standards aliquot were mixed each with 500μl of reagent in 1.5ml tubes. These tubes were then kept in boiling water bath for 8min and cooled afterwards on a water bath for 4min. A 200 μl aliquot was transferred to separate wells of a 96 well plate and OD was measured at 630 nm light wavelength. Glucose concentration in sample was calculated as follows: Concentration= OD sample- OD blank (mg/dl)/ Slope.

For trigyclerides assay, standards were diluted in distilled water to get a concentration of 1, 0.6, 0.3 mmol/l; the distilled water was taken as a blank sample and served as control. A 10 μl serum aliquot diluted 5-fold in distilled water was used for the assay. A working reagent was prepared by mixing 100 μl assay buffer, 2μl Enzyme mix, 5 μl lipase, 1μl ATP and 1μl dye in a clean tube. A 100 μl working buffer aliquot was added, and tap-mixed with sample/ standard. This mixture was incubated for 30 min at the room temperature, and OD was measured on a microplate reader at 550-585 nm light wavelengths. Triglyceride concentration of sample was calculated as follows: Concentration= (OD sample- OD water (mmol/ L)/ Slope) x N (where, N is the serum dilution factor).

**Network analysis**

To examine gene expression patterns across the day as represented by 6 time-point samples (ZT1, ZT5, ZT9, ZT13, ZT17, ZT21) in LDM and LDR states, we estimated a mean expression value for each physiological states using individual ANOVA models. This grouped 12 samples into 2 physiological states, which we used in principal component analysis (PCA) to show relationships between them. The PCA showed that gene expression patterns were most strongly affected by the state. However, as determined, the differences in gene sequences as compared to zebra finch genome may not actually represent expression differences, and the amplification number may be a technical artifact that can affect the expression measurements. Also, the 4 h expression patterns may create uncertain more subtle patterns in comparison between LDM and LDR states that in fact we wanted to assess in this experiment. Therefore, we considered temporal samples as 6 biological replicates for the specific state. The PCA was done again on the batch-corrected group values to ascertain that the batch effects were removed and that what the next biggest factors affecting expression might be.

We performed Weighted Gene Coexpression Network Analysis (WGCNA) to show association of gene expression with the physiological state. WGCNA can be considered a step-wise data reduction technique, which (a) starts from the level of thousands of variables (e.g., gene expression profiles), (b) identifies biologically interesting modules based on a node significance measure, (c) represents the modules by their centroids (e.g., eigenvectors or intramodular hubs), (d) uses intramodular connectivity (kIM or kME) as quantitative measures of module membership, and (e) combines node significance and module membership measures for identifying significant hub nodes. The module centric analysis alleviates the multiple testing problems inherent in high dimensional data, e.g., gene expression data. Thus, Thus, although similar to other clustering methods in the sense that it also calculates a distance metric between the expression patterns of all genes, WGCNA is a better analysis as it takes into the complexity of the distance metric. WGCNA starts with simple correlation values (usually Pearson’s) between all pairs of genes. Then, the correlations are transformed into an adjacency matrix by raising the correlations to a soft-thresholding power function, β. The parameter β is chosen based on the data set to achieve an approximate scale-free network (Zhang and Horvath 2005) and favors strong correlations over weak correlations. The adjacencies are next transformed into a topological overlap matrix (Zhao et al., 2010), which as a similarity measure can be subtracted from 1 to give a distance measure. These distance measures are then used in traditional hierarchical clustering to represent the relationships among genes in a familiar dendrogram. The next step in a WGCNA analysis is to break genes into clusters or “modules”. There are many different methods of cutting a dendrogram and WGCNA suggests a computational approach called Dynamic Branch Cut (Langfedler et al., 2008a). Here, we performed WGCNA in R package (Langfedler et al., 2008b) on the number of annotated transcripts that had a p-value < 0.001 in at least one of the experimental contrasts. There are many different parameter choices at each step in the process. After assessing a range of soft-thresholding values, we chose power β = 8. We calculated Pearson correlation coefficients between all pairs of probes in one block on a laptop computer with 64-bit Windows and 4 GB of RAM. We chose to use a signed adjacency and signed topological overlap matrix to preserve differences between the positive and negative correlations. Average linkage hierarchical clustering was used and modules were determined using the Dynamic Hybrid method with deepSplit = 2 and a minimum module size = 30. A second Partitioning Around Mediods-like stage of module detection was done with pamRespectsDendro = TRUE. At the end, modules with similar expression patterns were merged at mergeCutHeight = 0.2. Otherwise, the default values of the blockwise module functions were used. Once modules have been defined, an average expression profile of all genes in the model can be determined by calculating an eigengene value for each treatment group by taking the first principal component of gene expression values in the module. Relatively higher and lower expression values are represented by positive and eigengene values, respectively. The set of eigengene values can be taken as a proxy for an average expression pattern of all genes in a module (Fig 5 A). The total number of annotated transcripts x 12 samples data matrix has now been reduced down to 6 modules x 2 groups.
